# The quality of prison primary care: cross-sectional cluster-level analyses of prison healthcare data in the North of England

**DOI:** 10.1016/j.eclinm.2023.102171

**Published:** 2023-08-31

**Authors:** Kate McLintock, Robbie Foy, Krysia Canvin, Sue Bellass, Philippa Hearty, Nat Wright, Marie Cunningham, Nicola Seanor, Laura Sheard, Tracey Farragher

**Affiliations:** a Leeds Institute of Health Sciences (LIHS), University of Leeds, Level 10, Worsley Building, Clarendon Way, Leeds, LS2 9NL, UK; b School of Medicine, Keele University, David Weatherall Building, Staffordshire, ST5 5BG, UK; c Faculty of Science and Engineering, Institute of Sport, Manchester Metropolitan University, 99 Oxford Road, Manchester, M1 7EL, UK; d Spectrum Community Health CIC, Hebble Wharf, Wakefield, WF1 5RH, UK; e North of England Commissioning Support (NECS), John Snow House, Durham, DH1 3YG, UK; f Department of Health Sciences, University of York, Seebohm Rowntree Building, York, YO10 5DD, UK; g Division of Population Health, Health Services Research and Primary Care, University of Manchester, Room 2.544, Stopford Building, Oxford Road, Manchester, M13 9PT, UK

**Keywords:** Prisons, Primary health care, Quality of health care, Inequalities

## Abstract

**Background:**

Prisoners have significant health needs, are relatively high users of healthcare, and often die prematurely. Strong primary care systems are associated with better population health outcomes. We investigated the quality of primary care delivered to prisoners.

**Methods:**

We assessed achievement against 30 quality indicators spanning different domains of care in 13 prisons in the North of England. We conducted repeated cross-sectional analyses of routinely recorded data from electronic health records over 2017–20. Multi-level mixed effects logistic regression models explored associations between indicator achievement and prison and prisoner characteristics.

**Findings:**

Achievement varied markedly between indicators, prisons and over time. Achieved processes of care ranged from 1% for annual epilepsy reviews to 94% for blood pressure checks in diabetes. Intermediate outcomes of care ranged from only 0.2% of people with epilepsy being seizure-free in the preceding year to 34% with diabetes having sufficient blood pressure control. Achievement improved over three years for 11 indicators and worsened for six, including declining antipsychotic monitoring and rising opioid prescribing. Achievement varied between prisons, e.g., 1.93-fold for gabapentinoid prescribing without coded neuropathic pain (odds ratio [OR] range 0.67–1.29) and 169-fold for dried blood spot testing (OR range 0.05–8.45). Shorter lengths of stay were frequently associated with lower achievement. Ethnicity was associated with some indicators achievement, although the associations differed (both positive and negative) with indicators.

**Interpretation:**

We found substantial scope for improvement and marked variations in quality, which were largely unaltered after adjustment for prison and prisoner characteristics.

**Funding:**

10.13039/501100001809National Institute for Health and Care Research Health and 10.13039/501100022224Social Care and Delivery Research Programme: 17/05/26.

**Research in context**

**Evidence before this study**

We searched six databases (CINAHL, Criminal Justice Abstracts, MEDLINE, PsycInfo, Embase and Scopus) from January 2004 to April 2021. We chose 2004 as the start date as it marked the beginning of the prison healthcare governance transition from the Home Office to the National Health Service in the UK. Search terms were constructed around three concepts: quality indicators or performance measurement, primary care, and prison healthcare. We included research papers, commentaries, editorials, and grey literature from international sources. We updated the search using the same terms in PubMed in January 2023.

We found limited work on measurement of care quality, with nine studies describing indicator development. One article described a managed care programme in a US state prison healthcare system over 1994–2003, which summarised improvements in clinical performance for six long-term conditions.

**Added value of this study**

We assessed the quality of primary care across a range of indicators for 13 prisons in the North of England. There was substantial scope for improvement and marked variations in quality which were largely unaltered after adjustment for prison and prisoner characteristics. Whilst we found encouraging trends suggesting improvement over a three-year period for several indicators, such as increasing hepatitis B vaccination and decreasing gabapentinoid prescribing, we identified areas of concern, notably decreasing antipsychotic monitoring and increasing opioid prescribing. Shorter lengths of stay were frequently associated with lower achievement. Ethnicity was associated with some indicator achievement, but this differed with indicators. Unmatched comparisons in achievement from community settings were unfavourable for 22 out of 24 relevant indicators.

**Implications of all the available evidence**

Prisoners generally receive worse primary care than that delivered in the community. Concerted efforts are needed to move towards equivalence of healthcare and outcomes between incarcerated and community populations, as well as tackle inequalities in healthcare delivery amongst prisons. Our methods offer a foundation for scalable, data-driven improvement.

## Introduction

Over 10 million people are held in prisons worldwide.^1^ Prisoners have significant health needs, including high levels of long-term physical and mental illness, blood-borne virus infections and substance misuse.^2^^,^^3^ Older people, often with more complex health needs, are the fastest-growing group in the prison population in many countries; the number of prisoners aged 55 years or older in the United States quadrupled between 1993 and 2013.^4^ Prisoners are relatively high users of both primary care and inpatient healthcare,^5^ and face long waits for assessment and treatment.^6^ The standardised mortality rate for prisoners in England is 50% higher than that of the general population; the average age of death is 56 compared with almost 81 years in England.^7^

Strong primary care systems are associated with efficient and equitable population healthcare and health.^8^ However, prison healthcare faces challenges in providing a standard of care at least equivalent to that available in the wider community.^2^ Concerns raised about access and quality of prison healthcare suggest equivalence is not always achieved.^7^ Neglecting the health needs of prisoners has negative consequences for both individuals and wider society.^9^

Previous research into prison healthcare has tended to focus on specific problems, such as substance misuse,^10^ with less attention paid to the quality of ‘routine’ primary care. We examined the quality of primary care for a broad range of indicators in a sample of English prisons.

## Methods

### Study design and setting

We conducted repeated cross-sectional analyses of anonymised routinely collected electronic primary care data from 13 prisons in the North of England, measuring achievement against 30 quality indicators over a three-year period.

In England, prisoners are assigned to the lowest security category appropriate to manage their risks. Adult males are typically categorised A–D; category A for those whose escape would be highly dangerous, B for those who do not require maximum security but for whom escape needs to be made very difficult, C for those who cannot be trusted in open conditions but who are unlikely to try to escape, and category D open prisons for those who can be reasonably trusted not to attempt escape.^11^ Women are managed in open or closed conditions.^12^ Young Offender Institutions (YOIs) house prisoners aged 18–21 years. Of the 13 prisons we sampled, 10 housed adult males aged 21 years and over (two category A, three category B, three category C, and two category D open prisons), two were closed prisons (females aged 18 years and over), and one a YOI for males.

Spectrum Community Health Community Interest Company (Spectrum) delivered primary care in all prisons at the time of data extraction. The study population was determined by the provider and included around 30% of all English prisoners in June 2020.^13^ We followed STROBE guidance in reporting our results.^14^

### Variables

We identified and defined 371 potential indicators to assess the quality of prison primary care from existing guidance and literature.^15^, ^16^, ^17^, ^18^ We excluded 217 indicators that had been retired or superseded, were duplicates or were irrelevant to primary care. A stakeholder panel of eight healthcare professionals and academics from a range of criminal justice, health, and mental health backgrounds independently rated and re-rated the remaining indicators following feedback and discussion. The panel prioritised 60 indicators according to relevance to primary care, scope for measurement using routinely coded data, and potential for individual or population-level benefit based on existing clinical guidance. Out of these, we selected 36 indicators with the highest potential for patient or population benefit. Feasibility work demonstrated that six of these could not be reliably operationalised. Our final set of 30 indicators comprised 15 on long-term physical conditions, five on prevention and screening, four on mental illness, three on communicable disease, one on opioid prescribing and two on prison-specific procedures. Three of the 30 indicators had sub-indicators (one sub-indicator for hepatitis B vaccination and polypharmacy, and four for opioid and gabapentinoid prescribing). Four indicators were composite (combined) indicators. We pragmatically defined achievement for these: hepatitis B vaccination was achieved if at least one vaccination was administered, and antipsychotic monitoring, dementia diagnoses and diabetes care achieved if over 60% of recommended monitoring tests or care processes were completed.

Prison-level explanatory variables comprised prison name and category. Patient-level explanatory variables included age of individual at study census date (in decades, to protect anonymity), gender (as stated in the medical record), months of stay at census date (as categories) and Office for National Statistics coded ethnicity.

### Data sources

All English prisons use the SystmOne electronic health record. This clinical system includes prisoner demographic data via integration with the Prison National Offender Management Information System (NOMIS), health screening data from reception assessments, and data related to ongoing care including diagnoses (clinical codes), pathology results and prescribing.

We extracted these anonymised data remotely via Spectrum during April–November 2020, covering 1 April to 31 March across 2017–18, 2018–19 and 2019–20. We reviewed and iteratively refined each search.

### Statistical analysis

Indicators generally comprised a defined eligible population (e.g., people with diabetes) and whether they received a desired process of care or achieved a desired outcome within a given timeframe (e.g., blood pressure 140/80 mmHg or less within the preceding 12 months), in their current prison, or during time spent in other prisons. Higher percentage achievement was generally desirable for indicators. For indicators examining psychotropic, opioid and gabapentinoid prescribing, there was no specific criterion to compare against; generally, lower prescribing levels were desirable.

Multi-level mixed effects logistic regression models explored whether explanatory variables (both prison and patient specific) were associated with indicator achievement, with each indicator modelled separately.^19^ The unit of analysis was the patient. Each indicator model included year as both a random and fixed effect to account for the correlation between years and explore changes in achievement over time. The models had two levels (person identifier and year), as there are repeated measures for people across and within years (e.g., someone could have attended multiple prisons in the same year and over years). Each explanatory variable was included as fixed effects individually in each indicator model to explore association with achievement of that indicator. Modelling was not feasible for seven indicators where prisoner numbers were too small for ORs to be estimated.

We included the explanatory variables in multivariable multi-level mixed effects logistic regression models for each indicator as fixed effect covariates to explore whether variation in indicator achievement altered after adjustment for other factors. We present both the univariate and multivariable model results as ORs with 95% confidence intervals (CIs) and probability of achievement of the indicator (and 95% CI) for the multivariable models. All appropriate assumptions were checked (multicollinearity, residual normality, and homoscedasticity) and met in each of the multivariable indicator models; prison category was excluded from these models given the close correlation between it and prison identity. Statistical analyses used Stata 16 software.^20^

### Ethical approval

Ethical approval was granted by the University of Leeds (reference 18-093). HM Prison and Probation Service National Research Committee confirmed that as we used remotely collected, anonymised data the project did not require their approval.

### Role of the funding source

The study funder had no role in study design, collection, analysis, and interpretation of data, the writing of the report or the decision to submit the paper for publication.

## Results

### Study population

The total number of prisoners increased from 21,677 to 25,811 over 2017–20 (Table 1), 92% were male and 43% were located in category B prisons, 65% were aged 20-40 years and 58% had prison sentences of less than six months. Ethnicity data were missing for 18%; the majority of people included were White (72%).

**Table 1:** Study population characteristics.

Explanatory variables	Year and study population (%)^a^		
	2017–18	2018–19	2019–20
**Total study population**	21,677	22,099	25,811
**Gender**			
Male	19,977 (92.2%)	20,295 (91.8%)	23,570 (91.3%)
Female	1699 (7.8%)	1802 (8.1%)	1376 (5.3%)
Missing	<10 (<0.05%)^b^	<10 (<0.05%)^b^	865 (3.4%)
**Prison category**			
A	1664 (7.7%)	1670 (7.6%)	1838 (7.1%)
B	9254 (42.7%)	9442 (42.7%)	11,904 (46.1%)
C	6035 (27.8%)	6204 (28.1%)	6870 (26.7%)
Closed	1720 (7.9%)	1802 (8.2%)	2245 (8.7%)
D	2189 (10.1%)	2189 (9.9%)	2149 (8.3%)
Young Offenders Institution	815 (3.8%)	792 (3.6%)	805 (3.1%)
**Age (years)**			
10–<20	468 (2.2%)	436 (2.0%)	404 (1.6%)
20–<30	6994 (32.3%)	7163 (32.4%)	8064 (31.2%)
30–<40	7051 (32.5%)	7381 (33.4%)	9125 (35.4%)
40–<50	4114 (19.0%)	4180 (18.9%)	4948 (19.2%)
50–<60	2107 (9.7%)	1978 (9.0%)	2224 (8.6%)
60–<70	684 (3.2%)	701 (3.2%)	751 (2.9%)
70–<80	213 (1.0%)	209 (1.0%)	238 (0.9%)
80–<90	40 (0.2%)	45 (0.2%)	53 (0.2%)
90–<100	<10 (<0.05%)^b^	<10 (<0.05%)^b^	<10 (<0.05%)^b^
100–<110	<10 (<0.05%)^b^	c	c
**Length of stay (months)**			
<1	4474 (20.6%)	4801 (21.7%)	6764 (26.2%)
1–<6	8075 (37.3%)	7742 (35.0%)	10,802 (41.9%)
6–<12	3672 (16.9%)	3616 (16.4%)	3893 (15.1%)
12–<24	2832 (13.1%)	3752 (17.0%)	2600 (10.1%)
24+	2624 (12.1%)	2188 (9.9%)	1752 (6.8%)
**Ethnic group**			
White	15,638 (72.1%)	14,911 (67.5%)	16,606 (64.3%)
Mixed	431 (2.0%)	371 (1.7%)	409 (1.6%)
Asian/Asian British	813 (3.8%)	726 (3.3%)	755 (2.9%)
Black/Black British	404 (2.0%)	364 (1.6%)	451 (1.7%)
Chinese/Other	214 (1.0%)	167 (0.8%)	163 (0.6%)
Unclassified	372 (1.7%)	409 (1.9%)	387 (1.5%)
Missing	3805 (17.6%)	5151 (23.3%)	7040 (27.3%)

a Percentages may not total 100 due to rounding.

b Very small numbers suppressed (<10) to avoid disclosure.

c No data available.

### Results by quality indicator

**Fig. 1:**
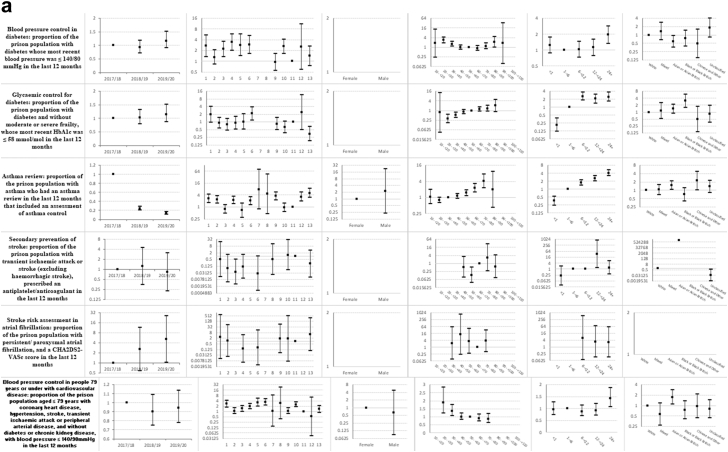

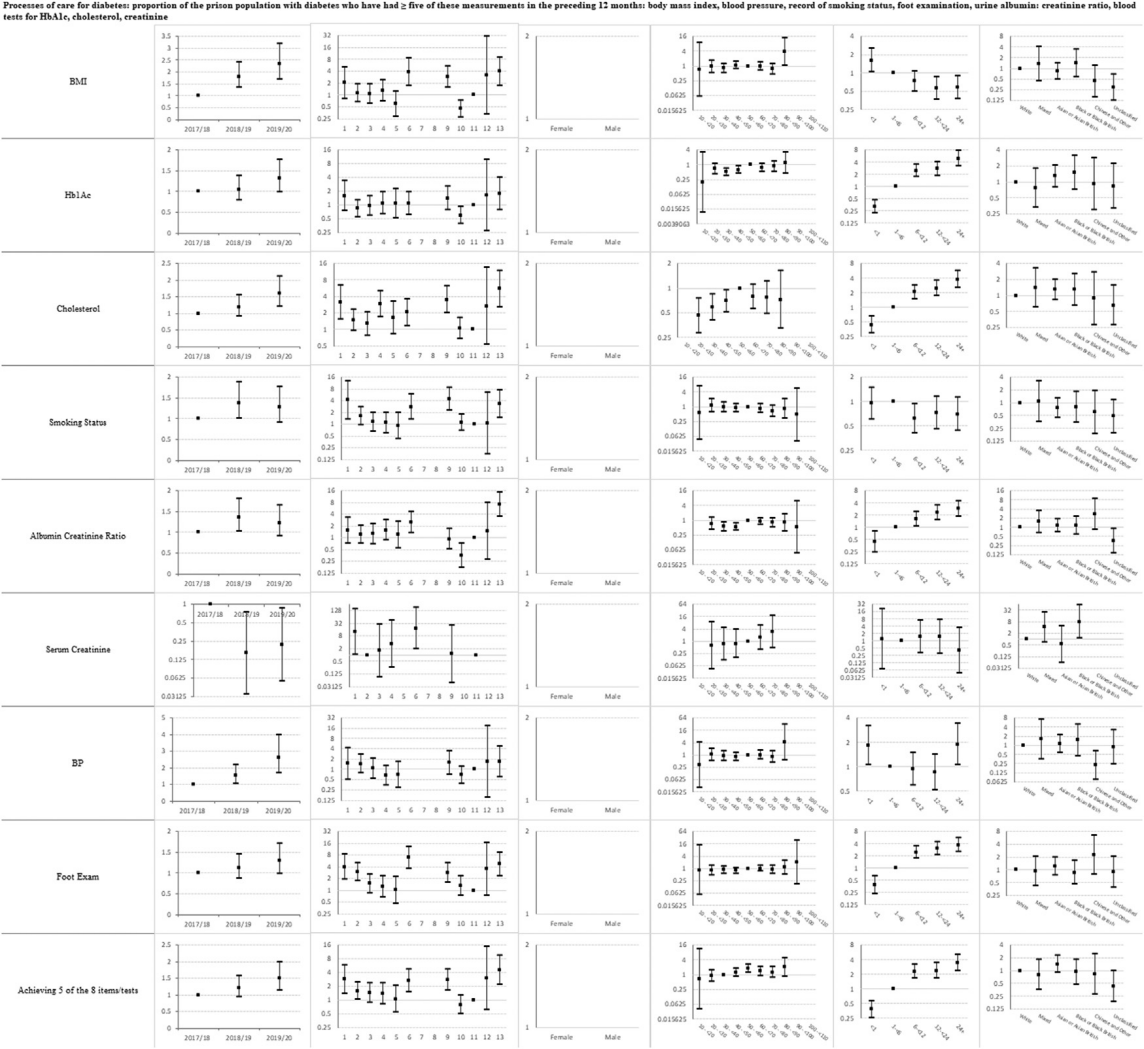

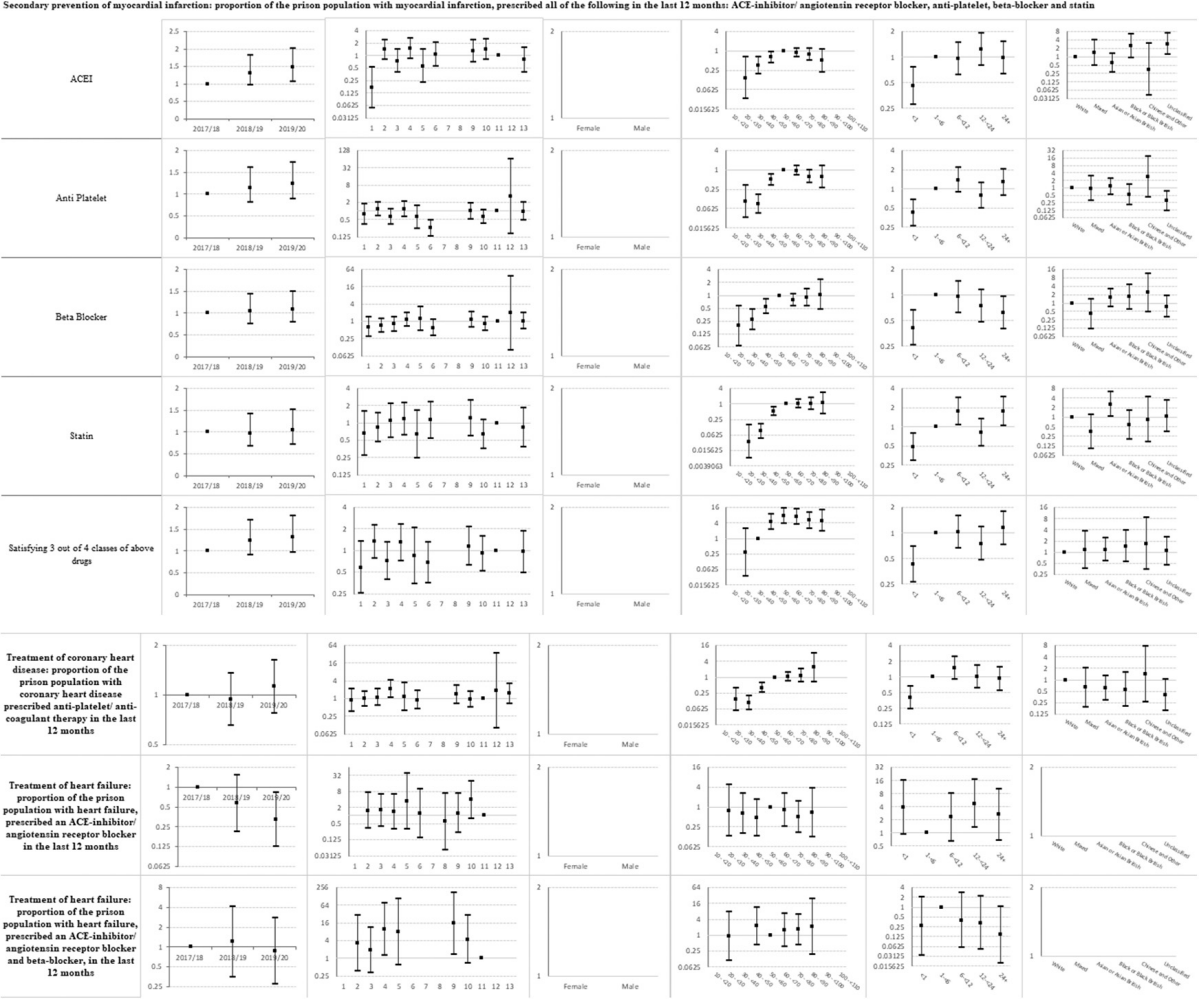

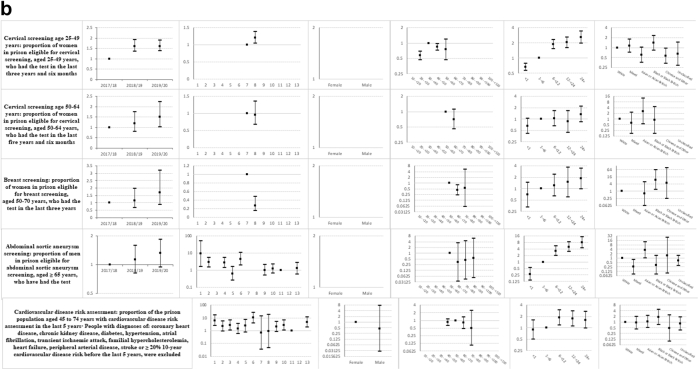

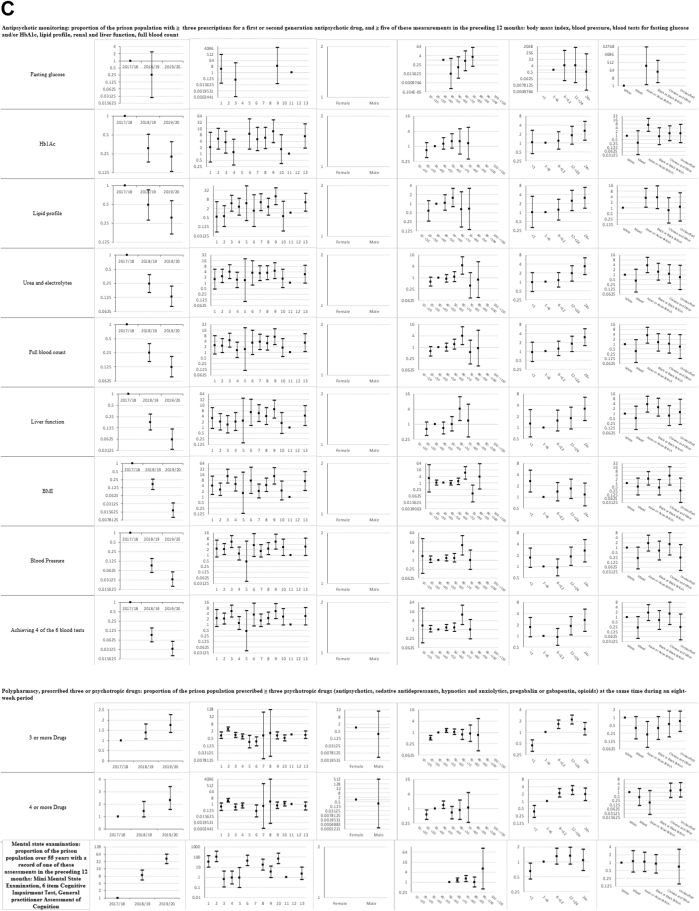

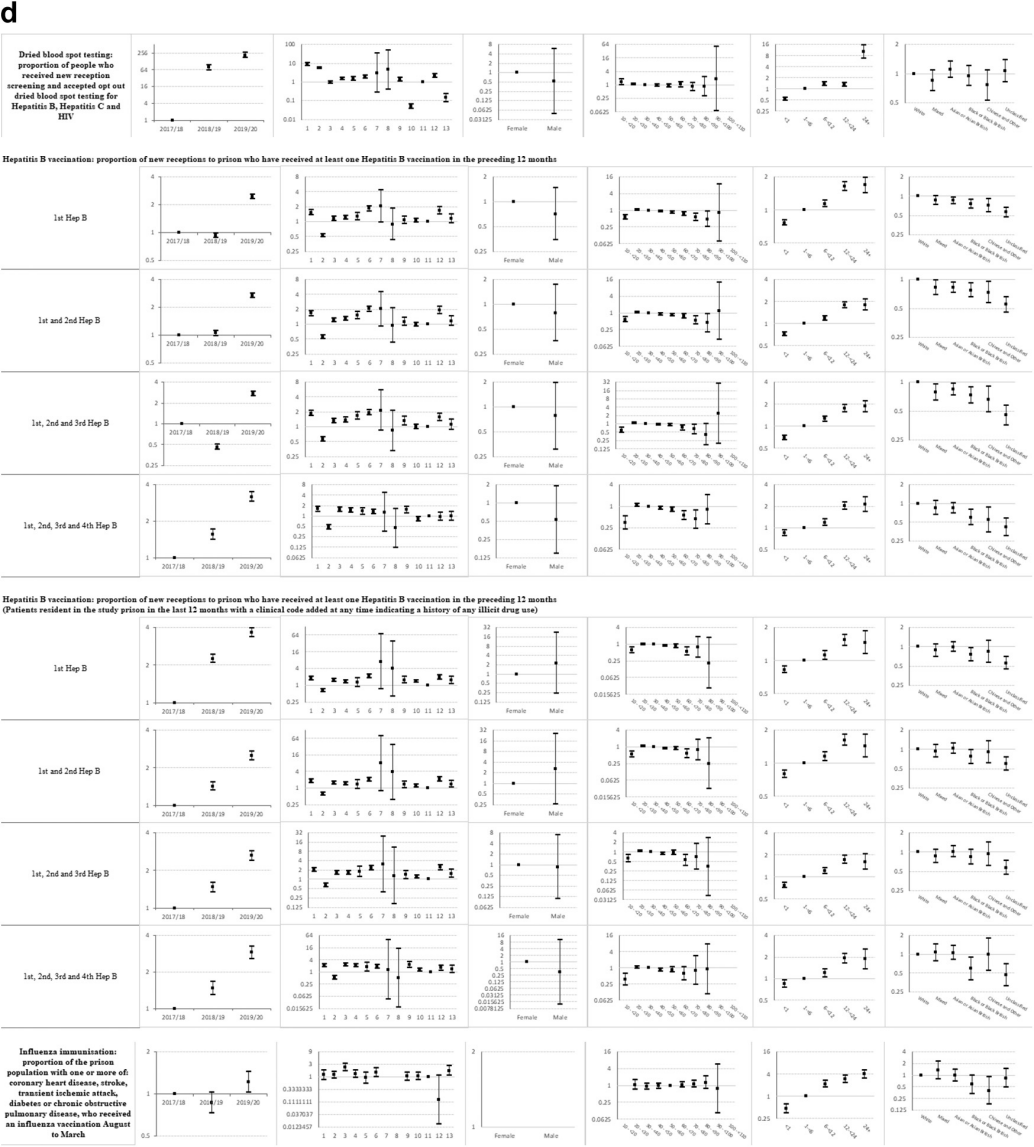

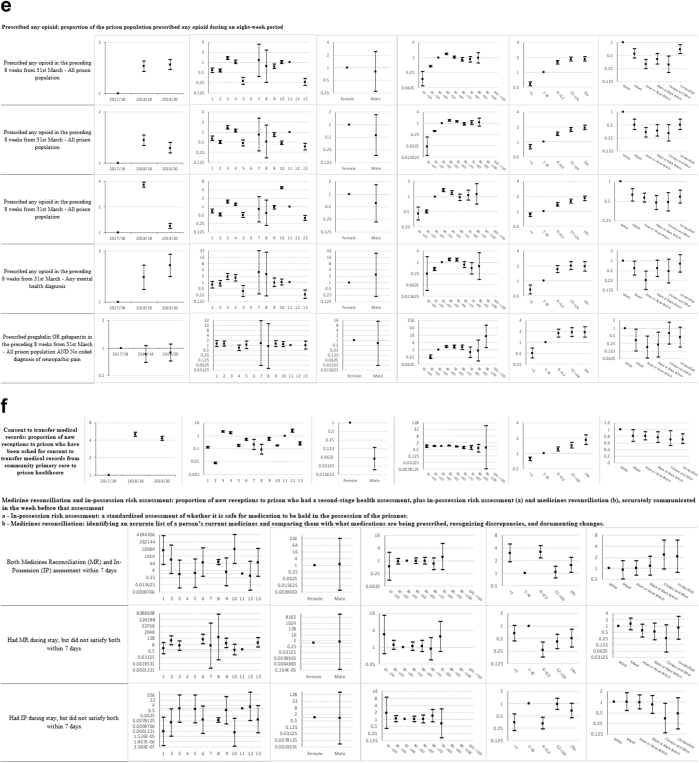
**a-f) Multi-level mixed effects logistic regression model results for each indicator by domains of care: Multivariable Odds Ratios (95% confidence intervals)**^**1**^. ^1^ Blank figures indicate insufficient data for OR estimates in multivariable models and where no year estimates then the indicator only available for 2019–20. a) Long-term conditions. b) Screening. c) Mental illness. d) Communicable disease. e) Opioid and gabapentinoid prescribing. f) Prison specific.

Descriptive statistics and multi-level mixed effects logistic regression model results for each indicator are provided in Supplementary sections 2 and 3 respectively. Supplementary section 1 (Table 2a–f) summarise indicator achievement by domains of care, based upon a study population of 25,811 people in 2019–20 unless otherwise stated. These summarises collated variation in percentage achievement of all indicators by domains of care and year, ORs trends and patterns by the explanatory variables and domains of care (irrespective of ‘significance’) as well as those statistically significant (at 5%) associations between achievement and the explanatory variables from the multivariable multi-level mixed effects logistic regression models. Fig. 1a–f shows the ORs with 95% CIs from the multivariable models for all indicators by domains of care.

### Long-term conditions (Table 2a in Supplementary section 1 and Fig. 1a)

Indicator achievement ranged from 0% for secondary prevention of myocardial infarction (MI), to 83% for anticoagulation for atrial fibrillation. Achievement was below 50% for six of 15 indicators in this clinical domain: secondary prevention of MI, epilepsy review and control, asthma review, blood pressure control in diabetes, glycaemic control for diabetes, and blood pressure control in people 79 years or under with cardiovascular disease (CVD). We observed mixed trends over 2017–20. Achievement improved for two indicators (processes of care for diabetes (OR 1.51; 95% CI 1.15, 1.99) and stroke assessment in atrial fibrillation (5.17; 1.02, 26.2)), and fell for three indicators (asthma review (0.14; 0.11, 0.17), treatment of heart failure with an angiotensin-converting enzyme (ACE) inhibitor or angiotensin receptor blocker (ARB) (0.32; 0.12, 0.82), and treatment of heart failure with both an ACE-inhibitor or ARB and a beta-blocker (0.87; 0.27, 2.76)). Variations in achievement amongst prisons ranged from over two-fold for treatment of coronary heart disease (ORs range 0.86–2.10) to 43-fold for secondary prevention of stroke (ORs range 0.03–1.29).

Achievement varied between prison categories, with no clear pattern by category or indicator. Achievement generally increased with length of stay. Compared to people staying one to six months, those with a stay of less than one month were less likely to have asthma reviews (0.36; 0.24, 0.53) whilst those staying over 24 months were more likely to receive diabetes processes of care (3.41; 2.32, 5.03).

Achievement generally improved with increasing age. Compared to those aged 30–39 years, people aged 50–59 years were more likely to receive diabetes processes of care (1.76; 1.23, 2.54) and asthma reviews. Patterns varied by ethnicity; compared to White people, glycaemic control of diabetes was more likely for Black or Black British people (3.08; 1.6, 5.91) whilst blood pressure control in diabetes was less likely for Asian or Asian British people (0.58; 0.36, 0.95).

### Screening (Table 2b in Supplementary section 1 and Fig. 1b)

Indicator achievement ranged from 30% for CVD risk assessment to 63.8% for cervical screening for ages 25–49 years. The likelihood of cervical screening increased over 2017–20 for those aged 25–49 years (1.61; 1.37, 1.89) and 50–64 years (1.5; 1.01, 2.24), but did not improve for other screening programmes. The likelihood of abdominal aortic aneurysm screening (ORs 0.63–9.12) and CVD risk assessment (ORs 0.69–10.04) varied over 14-fold between prisons.

Achievement generally increased with length of stay. People staying more than 24 months (8.04; 4.53, 14.26) were almost 30 times more likely to undergo abdominal aortic aneurysm screening (0.27; 0.14, 0.54), than people staying less than a month. Compared to White women, Chinese or Other women aged 25–49 years were less likely to have an adequate cervical screening test (0.6; 0.33, 0.95), and people of Mixed ethnicity were almost four times less likely to undergo abdominal aortic aneurysm screening (0.26; 0.08, 0.81).

### Mental illness (Table 2c in Supplementary section 1 and Fig. 1c)

Indicator achievement ranged from 5% for antipsychotic monitoring to 46% for diagnosis of dementia. The likelihood of mental state examination for people over 55 years increased 40-fold over 2017–20 (40.5; 25.3, 64.6), whilst antipsychotic monitoring fell over 80% (0.13; 0.07, 0.24). We found that 0.8% of prisoners were prescribed three or more and 0.4% prescribed four or more psychotropic drugs over the preceding eight weeks, with around two-fold increases in the likelihood of such prescribing over 2017–20 (OR for three or more 1.76; 1.37, 2.25 and OR for four or more 2.30; 1.56, 3.39). Variations in achievement amongst prisons ranged from 12-fold for antipsychotic monitoring (ORs 0.68–8.55) to 169-fold for mental state examination (ORs 0.65–109.76).

Antipsychotic monitoring was less likely in category B, C and closed prisons compared to category A prisons. Monitoring increased for people staying over 24 months (3.48; 1.66, 7.31). The likelihood of being prescribed three or more and four or more psychotropic drugs rose with increasing length of stay. Compared to people staying one to six months, those staying over 24 months were around twice as likely to be prescribed four or more psychotropics (1.92; 1.07, 3.42).

We observed variations by age and ethnic group. Compared to those aged 30–39 years, people aged 20–29 years were less likely to be prescribed at least three or four psychotropics (ORs 0.51; 0.38, 0.69 and 0.56; 0.36, 0.87 respectively). Compared to White people, Asian or Asian British and Black or Black British people were more likely to receive antipsychotic monitoring (ORs 5.67; 1.84, 17.46 and 4.04; 1.12, 14.54 respectively). Asian or Asian British people were also less likely to be prescribed three or more psychotropic drugs (0.22; 0.07, 0.69).

### Communicable disease (Table 2d in Supplementary section 1 and Fig. 1d)

Indicator achievement ranged from 45% for dried blood spot testing (DBST) for hepatitis B, hepatitis C and HIV to 50% for receipt of at least one hepatitis B vaccination for people with a history of illicit drug use. The likelihood of achievement in this domain generally increased over 2017–20, ranging from a 1.2-fold increase for influenza immunisation (OR 1.22; 1.02, 1.45) to 200-fold for DBST (212.13; 170.37, 264.13). Variations in achievement between prisons ranged from four-fold for hepatitis B vaccination (ORs 0.52–2.04) to 169-fold for DBST (ORs 0.05–8.45).

Compared to category A prisons, uptake of DBST was higher in all other categories. Achievement generally increased with length of stay. Compared to people staying one to six months, those staying less than one month were half as likely to accept DBST (0.53; 0.48, 0.58) and those staying over 24 months were 10 times as likely to accept testing (10.15; 6.73, 15.31). We observed variations by ethnicity. Compared to White people, Chinese or Other people were less likely to receive one hepatitis B vaccination (0.72; 0.57, 0.92).

### Opioid and gabapentinoid prescribing (Table 2e in Supplementary section 1 and Fig. 1e)

Of the total study population, 12% had been prescribed any opioid, 9% strong opioids, and 0.9% gabapentinoids (with no coded diagnosis of neuropathic pain) in the preceding eight weeks. Opioids were co-prescribed with benzodiazepines in 9%, and in 19% of people with a coded mental illness. The likelihood of any opioid prescribing increased over 2017–20 (1.47; 1.38, 1.58). Variations in prescribing between prisons ranged from two-fold for prescribing of gabapentinoids (ORs 0.67–1.29) to 12-fold for co-prescribed opioids and benzodiazepines (ORs 0.39–4.68).

Patterns of prescribing by age were broadly similar across all opioid and gabapentinoid sub-indicators, with lower rates of prescribing for people aged under 30 years (e.g., OR for 20–29 years prescribed any opioid 0.44; 0.41, 0.48) and generally higher for people over 40 years (e.g., OR for 40–49 years prescribed any opioid 1.38; 1.29, 1.48), compared to people aged 30–39 years.

Compared to White people, all other ethnic groups were less likely to be prescribed any opioid, any strong opioid, or any opioid with benzodiazepines. Likelihoods of any opioid prescribing were lower in people of Mixed ethnicity (0.55; 0.43, 0.71), Asian or Asian British people (0.32; 0.25, 0.4), Black or Black British people (0.41; 0.31, 0.54) and Chinese or Other people (0.31; 0.2, 0.48).

### Prison specific (Table 2f in Supplementary section 1 and Fig. 1f)

Indicator achievement ranged from 38% for completion of medicines reconciliation and in-possession risk assessment, to 70% for consent to transfer medical records from community general practice to the prison healthcare service. The likelihood of consent to transfer medical records increased four-fold over 2017–20 (4.28; 3.96, 4.62). Variations in achievement amongst prisons ranged from 337-fold variation for consent to transfer medical records (ORs 0.007–2.36) to 21,600-fold in the likelihood of receiving medicines reconciliation assessments (ORs 0.45–9724.5).

Compared to those staying one to six months, people were more likely to receive medicines reconciliation and in-possession risk assessment if they stayed less than a month (3.02; 1.86, 4.89), six to 12 months (3.17; 2.26, 4.44), or over 24 months (1.54; 1.0, 2.33).

Men were ten times less likely to be asked for consent to transfer medical records than women (0.1; 0.02, 0.14). Compared to people aged 30–39 years, those aged 50–69 years were less likely to be asked for consent to transfer medical records (e.g., OR for 60–69 years 0.72; 0.58, 0.89). Compared to White people, all other ethnic groups were less likely to be asked for consent to transfer medical records; Mixed ethnicity (0.80; 0.65, 0.99), Asian or Asian British people (0.80; 0.69, 0.92), Black or Black British people (0.75; 0.61, 0.93) and Chinese or Other people (0.70; 0.52, 0.96).

## Discussion

We found variations in the quality of primary care across a range of indicators in multiple prisons and identified substantial scope for improvement. Gaps and variations in care reflected both broad primary care needs (e.g., diabetes care) and recognised priorities in this population (e.g., mental illness). Variations between prisons were largely unexplained by available population characteristics, suggesting that, within the context of one provider system, they are likely to be attributable to local differences in healthcare organisation and delivery.

We found encouraging trends suggesting improvement over time for several indicators, such as an increase in hepatitis B vaccination and a reduction in gabapentinoid prescribing, and strengths in performance, such as secondary prevention of stroke. However, we identified areas of concern where overall achievement had declined over a three-year period, notably decreasing antipsychotic monitoring, and increasing opioid prescribing, having excluded opioid substitutes specifically prescribed for drug dependence.

Relatively short lengths of stay were frequently associated with lower achievement across prison specific, long-term conditions, and screening domains. Shorter stays could represent missed opportunities for health intervention and may accompany recidivism, reflecting the negative health impact of repeated incarceration.^21^ Rapid population turnover significantly challenges healthcare delivery to the many people passing through prisons each year, estimated to exceed 30 million worldwide.^22^

We observed no consistent patterns in achievement by gender, age, or prison category. Associations between ethnic group and indicator attainment varied. For example, compared to White people, those from other ethnic minorities were less likely to be vaccinated against hepatitis B, but also less likely to be prescribed opioids or gabapentinoids. Asian or Asian British people were less likely to achieve blood pressure control in diabetes, but more likely to achieve blood pressure control in cardiovascular disease.

To contextualise our findings, we compared indicator achievement from community settings, albeit without any adjustment for demographic differences. Comparisons were unfavourable for 22 out of 24 relevant indicators and one sub-indicator (Supplementary section 4). For example, less than half of eligible prisoners (45%) received influenza vaccination, compared with over 70% of eligible people in the community during 2019–20. Strong opioid prescribing was much higher for prisoners, although this may also be partly explained by demographic differences and the exclusion of people with coded substance misuse from the community study.^23^ Our work is consistent with the limited international literature measuring inequities in prison settings, specifically in cancer screening and cardiovascular risk assessment.^24^

The shortcomings in care we found could be anticipated given the multiple challenges prison healthcare services face. These services often operate in dated buildings and care environments, amidst security constraints and with chronic understaffing affecting both prison and prison healthcare workforces.^2^^,^^25^^,^^26^ These challenges, combined with the significant health needs of prisoners, highlight a disparity between available resources and need, which may exacerbate recognised health inequalities. Furthermore, prison healthcare is not financially incentivised as community general practice in England is. For example, the same Quality of Outcomes Framework (QOF) indicators are used in prison and community, but achievement of targets is not linked to payment in prison healthcare. QOF has led to improvements in care quality, reduced inequalities in delivery of care and advanced infrastructure and data collection in community general practice.^27^

We highlight five study limitations. First, our analysis used data from only one prison healthcare provider in Northern England. Our study population gender, age and length of stay were broadly consistent with national profiles,^28^^,^^29^ except that percentages of coded Black and Minority Ethnic groups were lower at around 7% compared to 27% from criminal justice statistics.^30^ Second, clinical coding is relatively poor in prison healthcare,^31^ partly because of the absence of incentives that are available to community primary care. We selected indicators where we considered coding sufficiently reliable to enable comparisons. Third, whilst using routinely collected electronic data allowed an efficient and scalable assessment of care, it cannot capture all important facets of quality, such as prisoners’ experiences. Fourth, with so many comparisons, some associations may be spurious. Five, we could not assess the contributions of care delivered in community general practice before or after incarceration given restrictions on data sharing. This is particularly relevant for short lengths of stay, where we may have under-estimated care delivered within any given 12-month period. Future research and initiatives to address continuity of care would be strengthened by data sharing across prison and community systems.

Whilst not a limitation we should be mindful of interpreting our ORs as risks. ORs can strongly overestimate the prevalence ratio, particularly when prevalence is high (or indicator achievement in this case). With our varying indicator achievement, we have not interpreted are results as risks but discussed as less/more likely to avoid this misconception.^32^^,^^33^ We did not employ any selection methods to reduce the number of explanatory variables in each of the multivariable indicator models, as the aim of the analysis was fundamentally descriptive, rather than inferential or predictive.^34^ However, as logistic regression is event-driven, we were mindful to balance the number of explanatory variables included.^35^ Therefore, we pre-defined, based on discussions within the team, the relatively small number of explanatory factors that would be potentially important and measurable to include.

Improvement in the quality of primary care in prisons is likely to require coordinated action across system, organisational and team levels. At the system level, improved levels of healthcare staffing and linkage of community and prison records may enhance continuity and safety.^2^^,^^36^, ^37^, ^38^ Innovations such as telemedicine may improve access to and cost-effectiveness of care.^39^ At organisational and team levels, actions to mitigate the impact of short sentences and restrictions inherent in prison regimes whilst tailored support specific to minority groups (e.g., for uptake of screening, interpretation services) may help address inequalities in access to care. Overall, the gaps and variations in quality of primary care we identified suggest that prisons be a key focus of public health programmes to reduce health inequalities.

The next challenge is to move beyond description, to developing and evaluating improvement strategies. Our demonstrated use of a suite of indicators spanning different domains of care could represent foundational work for an evidence-based data-driven approach, such as cyclical audit and feedback.^40^ Routine data capture and reporting may also enhance understanding of the health of prison populations and inform policies for improvement at national and international levels.^2^

## Contribution

TF, RF, NW and LS conceived the study. TF, LS, RF, NW, KM and NS designed the study and obtained funding. KC, SB, PH, KM, MC, NW and RF contributed to indicator development and data collection. TF and PH accessed and verified the data. TF was responsible for statistical analyses and all authors were involved in data interpretation. KM, TF and RF drafted the manuscript. All authors commented on further revisions and were responsible for the decision to submit the manuscript for publication. TF is guarantor of the paper.

## Data sharing statement

The anonymised data was provided by Spectrum Community Health Community Interest Company via a Data Sharing Agreement (DSA). As part of the DSA these data cannot be shared outside the DSA signatories and so further access would have to be arranged directly with Spectrum after appropriate ethical approval and signing of data sharing agreements. A data dictionary of the anonymised data extracts is available on request from the corresponding author.

A study protocol including statistical plan is provided with publication.

## Declaration of interests

KM reports grants from National Institute for Health and Care Research, during the conduct of the study; other from Spectrum Community Health CIC, outside the submitted work.

PH was employed by Spectrum Community Health CIC between July 2016 and July 2023. NW was employed by Spectrum Community Health CIC between 2015 and 2022. Spectrum received funding from the NIHR for work on this project. Spectrum also received funding from UKRI to fund work on a study exploring the impact of COVID-19 on prison healthcare.

KC, RF and TF report grants from National Institute for Health and Care Research, during the conduct of the study.

## Appendix A Supplementary data

Supplementary sections

HSDR170226_Full_20180912_clean
