# Supplementary material for: The quality of prison primary care: cross-sectional cluster-level analyses of prison healthcare data in the North of England
Source: eClinicalMedicine. 2023 Aug 31;63:102171. doi: 10.1016/j.eclinm.2023.102171 (PMC10484963; doi:10.1016/j.eclinm.2023.102171)
Supplement: Supplementary sections [file mmc1.pdf]

Table of Contents of Supplementary Sections

Supplementary Section 1. Summaries of Multi-level mixed effects logistic models of indicator achievement by domains of care	Pages 2-17
Supplementary Section 2: Descriptive statistics for each indicator	Pages 18-127
Supplementary Section 3: Multi-level mixed effects logistic regression model results for each indicator	Pages 128-164
Supplementary Section 4. Comparisons with achievement in community settings	Pages 165-168

Supplementary Section 1. Summaries of Multi-level mixed effects logistic models of indicator achievement by domains of care

Table 2a. Long-term conditions

Indicator	Achievement		Variation in ORs between prisons (range of ORs)	Association between explanatory variables and achievement (OR; 95% CI)									
	2019-20 (%)	2019-20 compared to 2017-18 (OR; 95% CI)		Prison category[1] (comparator category A)		Gender[2] (comparator female)		Age[2] (comparator 30-39 years)		Length of stay (months)[2] (comparator 1-<6 months)		Ethnic group[2] (comparator White people)	
				Higher	Lower	Higher	Lower	Higher	Lower	Higher	Lower	Higher	Lower
Blood pressure control in diabetes: proportion of the prison population with diabetes whose most recent blood pressure was ≤ 140/80 mmHg in the last 12 months	261 of 770 eligible people (33·9%)	No change (1·17; 0·9, 1·51)	3·6-fold (0·95 to 3·42)	C (1·94; 1·47, 2·57)<br><br>Closed (2·94; 2·05, 4·22)<br><br>D (1·61; 1·14, 2·27)	··	··	··	20-29 (2·82; 1·86526, 4·3)<br><br>30-39 (1·59; 1·13148, 2·2)<br><br>80-89 (2·15; 1·00321, 4·6)<br><br>(comparator 50-59 years)	··	> 24 (1·95; 1·34, 2·85)	··	··	Asian/Asian British (0·58; 0·36, 0·95)
Processes of care for diabetes: proportion of the prison population with diabetes who have had ≥ five of these measurements in the preceding 12 months: body mass index, blood pressure, record of smoking status, foot examination, urine albumin: creatinine ratio, blood tests for HbA1c, cholesterol, creatinine	426 of 770 eligible people (55·3%)	Increase (1·51; 1·15, 1·99)	5·7-fold (0·78 to 4·46)	··	B (0·10; 0·07, 0·13)<br><br>C (0·30; 0·22, 0·4)<br><br>Closed (0·12; 0·08, 0·18)<br><br>D (0·37; 0·26, 0·54)	··	··	50- 59 (1·76; 1·23, 2·54)	··	6-12 (2·29; 1·65, 3·19)<br><br>12-24 (2·39; 1·67, 3·42)<br><br>> 24 (3·41; 2·32, 5·03)	< 1 (0·38; 0·25, 0·58)	··	··
Glycaemic control for diabetes: proportion of the prison population with diabetes and without moderate or severe frailty, whose most recent HbA1c was ≤ 58 mmol/mol in the last 12 months	260 of 756 eligible people (34·4%)	No change (1·15; 0·88, 1·51)	7-fold (0·34 to 2·39)	··	B (0·36; 0·28, 0·47)<br><br>Closed (0·60;	··	··	··	20-29 (0·31; 0·16, 0·58)<br><br>30-39 (0·52; 0·35, 0·76)<br><br>(comparator 50-59 years)	6-12 (2·58; 1·83, 3·64)<br><br>12-24 (2·07;	< 1 (0·21; 0·12, 0·38)	Black/Black British (3·08; 1·6, 5·91)	··

Indicator	Achievement		Variation in ORs between prisons (range of ORs)	Association between explanatory variables and achievement (OR; 95% CI)									
	2019-20 (%)	2019-20 compared to 2017-18 (OR; 95% CI)		Prison category[1] (comparator category A)		Gender[2] (comparator female)		Age[2] (comparator 30-39 years)		Length of stay (months)[2] (comparator 1-<6 months)		Ethnic group[2] (comparator White people)	
				Higher	Lower	Higher	Lower	Higher	Lower	Higher	Lower	Higher	Lower
					0·41, 0·86)					1·42, 3·01)<br><br>> 24 (2·47; 1·68, 3·65)			
Glycaemic control for diabetes with frailty: proportion of the prison population with diabetes and moderate or severe frailty, whose most recent HbA1c was ≤ 75 mmol/mol in the last 12 months	9 of 14 eligible people (64·3%)	[3]	[3]	[3]	[3]	[3]	[3]	[3]	[3]	[3]	[3]	[3]	[3]
Asthma review: proportion of the prison population with asthma who had an asthma review in the last 12 months that included an assessment of asthma control	136 of 4,459 eligible people (3·1%)	Decrease (0·14; 0·11, 0·17)	11·2-fold (0·68 to 7·62)	··	B (0·09; 0·07, 0·12)<br><br>C (0·20; 0·16, 0·25)<br><br>Closed (0·25; 0·19, 0·32)<br><br>D (0·18; 0·14, 0·24)<br><br>YOI (0·31; 0·22, 0·43)	··	··	50-59 (1·48; 1·15, 1·9)<br><br>60-69 (2·26; 1·57, 3·25)<br><br>70-79 (4·11; 2·26, 7·46)	··	6-12 (1·78; 1·4, 2·25)<br><br>12-24 (2·58; 2·04, 3·27)<br><br>> 24 (4·06; 3·14, 5·24)	< 1 (0·36; 0·24, 0·53)	Asian/Asian British (1·49; 1·04, 2·14)<br><br>Chinese/Other (2·21; 1·03, 4·75)	··
Epilepsy review and control: proportion of the prison population with epilepsy and prescribed antiepileptic drug(s), that had an annual	Annual review: 5 of 419 eligible people (1·2%)	[3]	[3]	[3]	[3]	[3]	[3]	[3]	[3]	[3]	[3]	[3]	[3]

Indicator	Achievement		Variation in ORs between prisons (range of ORs)	Association between explanatory variables and achievement (OR; 95% CI)									
	2019-20 (%)	2019-20 compared to 2017-18 (OR; 95% CI)		Prison category[1] (comparator category A)		Gender[2] (comparator female)		Age[2] (comparator 30-39 years)		Length of stay (months)[2] (comparator 1-<6 months)		Ethnic group[2] (comparator White people)	
				Higher	Lower	Higher	Lower	Higher	Lower	Higher	Lower	Higher	Lower
review and were seizure free in the last 12 months	Seizure free: 1 of 419 eligible people (<1·00%)												
Secondary prevention of stroke: proportion of the prison population with transient ischaemic attack or stroke (excluding haemorrhagic stroke), prescribed an antiplatelet/anticoagulant in the last 12 months	51 of 86 eligible people (59·3%)	No change (0·84; 0·22, 3·16)	43-fold (0·03 to 1·29)	B (14·65; 5·32, 40·38)<br><br>C (8·23; 3·94, 17·2)<br><br>D (23·96; 5·08, 113·02)	··	··	··	··	··	··	··	··	··
Anticoagulation for atrial fibrillation: proportion of the prison population with persistent/ paroxysmal atrial fibrillation and CHA2DS2-VASc score ≥ two, with a prescription for warfarin/direct-acting oral anticoagulant in the last 12 months	45 of 54 eligible people (83·3%)	[3]	[3]	[3]	[3]	[3]	[3]	[3]	[3]	[3]	[3]	[3]	[3]
Stroke risk assessment in atrial fibrillation: proportion of the prison population with persistent/ paroxysmal atrial fibrillation, and a CHA2DS2-VASc score in the last 12 months	15 of 25 eligible people (60%)	Increase (5·17; 1·02, 26·2)	34·3-fold (0·15 to 5·14)	··	C (0·19; 0·05, 0·74)	··	··	··	··	··	··	··	··
Blood pressure control in people 79 years or under with cardiovascular disease: proportion of the prison population aged ≤ 79 years with coronary heart disease, hypertension, stroke, transient	586 of 1,311 eligible people (44·7%)	No change (0·94; 0·78, 1·13)	6·6-fold (0·52 to 3·42)	C (1·32; 1·08, 1·62)<br><br>Closed (2·31;	··	··	··	20-29 (1·88; 1·23, 2·86)<br><br>30-39 (1·36; 1·06, 1·74)	··	> 24 (1·42; 1·08, 1·87)	··	Asian/Asian British (1·76; 1·09, 2·84)	··

Indicator	Achievement		Variation in ORs between prisons (range of ORs)	Association between explanatory variables and achievement (OR; 95% CI)									
	2019-20 (%)	2019-20 compared to 2017-18 (OR; 95% CI)		Prison category[1] (comparator category A)		Gender[2] (comparator female)		Age[2] (comparator 30-39 years)		Length of stay (months)[2] (comparator 1-<6 months)		Ethnic group[2] (comparator White people)	
				Higher	Lower	Higher	Lower	Higher	Lower	Higher	Lower	Higher	Lower
ischaemic attack or peripheral arterial disease, and without diabetes or chronic kidney disease, with blood pressure ≤ 140/90mmHg in the last 12 months				1·74, 3·06)<br><br>D (2·06; 1·58, 2·68)				(comparator 50-59 years)					
Blood pressure control in people 80 years or over with cardiovascular disease: proportion of the prison population aged ≥ 80 years with coronary heart disease, hypertension, stroke, transient ischaemic attack or peripheral arterial disease, and without diabetes or chronic kidney disease, with blood pressure ≤ 150/90mmHg in the last 12 months	10 of 19 eligible people (52·6%)	[3]	[3]	[3]	[3]	[3]	[3]	[3]	[3]	[3]	[3]	[3]	[3]
Secondary prevention of myocardial infarction: proportion of the prison population with myocardial infarction, prescribed all of the following in the last 12 months: ACE-inhibitor/ angiotensin receptor blocker, anti-platelet, beta-blocker and statin	0 of 492 eligible people (0%)	··	··	··	··	··	··	··	··	··	··	··	··
Treatment of coronary heart disease: proportion of the prison population with coronary heart disease prescribed anti-platelet/ anti-coagulant therapy in the last 12 months	360 of 494 eligible people (72·9%)	No change (1·13; 0·78, 1·64)	2·4-fold (0·86 to 2·10)	··	B (0·46; 0·32, 0·66)<br><br>Closed (0·23; 0·14, 0·4)	··	··	··	20-29 (0·15; 0·05, 0·4)<br><br>30-39 (0·11; 0·06, 0·19)<br><br>40-49 (0·40; 0·26, 0·6)<br><br>(comparator 50-59 years)	··	< 1 (0·41; 0·25, 0·67)	··	··
Treatment of heart failure: proportion of the prison population with heart failure, prescribed an ACE-inhibitor/	62 of 98 eligible people (63·3%)	Decrease (0·32; 0·12, 0·82)	6·6-fold (0·6 to 3·94)	B (2·36; 1·02, 5·49)	··	··	··	··	··	··	··	··	··

Indicator	Achievement		Variation in ORs between prisons (range of ORs)	Association between explanatory variables and achievement (OR; 95% CI)									
	2019-20 (%)	2019-20 compared to 2017-18 (OR; 95% CI)		Prison category[1] (comparator category A)		Gender[2] (comparator female)		Age[2] (comparator 30-39 years)		Length of stay (months)[2] (comparator 1-<6 months)		Ethnic group[2] (comparator White people)	
				Higher	Lower	Higher	Lower	Higher	Lower	Higher	Lower	Higher	Lower
angiotensin receptor blocker in the last 12 months				C (3·21; 1·4, 7·36)<br><br>D (5·74; 1·42, 23·28)									
Treatment of heart failure: proportion of the prison population with heart failure, prescribed an ACE-inhibitor/ angiotensin receptor blocker and beta-blocker, in the last 12 months	49 of 61 eligible people (80·3%)	Decrease (0·87; 0·27, 2·76)	··	··	··	··	··	··	··	··	··	··	··

1 Univariate analysis

2 Multivariable analysis

3 Insufficient data for multivariable analysis

·· No statistically significant result (at 5%)

Table 2b. Screening

Indicator	Achievement		Variation in ORs between prisons (range of ORs)	Association between explanatory variables and achievement (OR; 95% CI)									
	2019-20 (%)	2019-20 compared to 2017-18 (OR; 95% CI)		Prison category[1] (comparator category A)		Gender[2] (comparator female)		Age[2] (comparator 30-39 years)		Length of stay (months)[2] (comparator 1-<6 months)		Ethnic group[2] (comparator White people)	
				**Higher**	**Lower**	**Higher**	**Lower**	**Higher**	**Lower**	**Higher**	**Lower**	**Higher**	**Lower**
Cervical screening age 25-49 years: proportion of women in prison eligible for cervical screening, aged 25-49 years, who had the test in the last three years and six months	1,145 of 1,796 eligible people (63·8%)	Increase (1·61; 1·37, 1·89)	··	··	··	··	··	··	20-29 (0·6; 0·47, 0·68)<br><br>40-49 (0·8; 0·72, 0·97	6-12 (1·9; 1·52, 2·29)<br><br>12-24 (2·0; 1·61, 2·58)<br><br>> 24 (2·6; 1·97, 3·43)	< 1 (0·7; 0·56, 0·78)	··	Chinese/Other (0·6; 0·33, 0·95)
Cervical screening age 50-64 years: proportion of women in prison eligible for cervical screening, aged 50-64 years, who had the test in the last five years and six months	159 of 259 eligible people (61·4%)	Increase (1·5; 1·01, 2·24)	··	··	··	··	··	··	··	··	··	··	··
Breast screening: proportion of women in prison eligible for breast screening, aged 50-70 years, who had the test in the last three years	59 of 179 eligible people (33%)	Increase (1·69; 0·88, 3·21)	··	··	··	··	··	··	··	··	··	··	··
Abdominal aortic aneurysm screening: proportion of men in prison eligible for abdominal aortic aneurysm screening, aged ≥ 65 years, who have had the test	241 of 540 eligible people (44·6%)	No change (1·32; 0·94, 1·85)	14·5-fold (0·63 to 9·12)	··	B (0·29; 0·22, 0·38)<br><br>C (0·51; 0·39, 0·68)<br><br>D (0·66; 0·44, 0·99)	··	··	··	··	12-24 (5·04; 2·98, 8·54)<br><br>> 24 (8·04; 4·53, 14·26)	< 1 (0·27; 0·14, 0·54)	Mixed (3·59; 1, 12·89)	··

Indicator	Achievement		Variation in ORs between prisons (range of ORs)	Association between explanatory variables and achievement (OR; 95% CI)									
	2019-20 (%)	2019-20 compared to 2017-18 (OR; 95% CI)		Prison category[1] (comparator category A)		Gender[2] (comparator female)		Age[2] (comparator 30-39 years)		Length of stay (months)[2] (comparator 1-<6 months)		Ethnic group[2] (comparator White people)	
				Higher	Lower	Higher	Lower	Higher	Lower	Higher	Lower	Higher	Lower
Cardiovascular disease risk assessment: proportion of the prison population aged 45 to 74 years with cardiovascular disease risk assessment in the last 5 years· People with diagnoses of: coronary heart disease, chronic kidney disease, diabetes, hypertension, atrial fibrillation, transient ischaemic attack, familial hypercholesterolemia, heart failure, peripheral arterial disease, stroke or ≥ 20% 10-year cardiovascular disease risk before the last 5 years, were excluded	353 of 1,183 eligible people (29·8%)	[4]	14·6-fold (0·69 to 10·04)	D (1·82; 1·17, 2·84)	B (0·44; 0·3, 0·63)<br><br>C (0·56; 0·41, 0·78)<br><br>Closed (0·48; 0·29, 0·8)	··	··	··	··	6-12 (1·88; 1·19, 2·97)<br><br>12-24 (1·78; 1·12, 2·82)	··	··	··

[1] Univariate analysis

[2] Multivariable analysis

[4] Data only available for 2019-20

·· No statistically significant result (at 5%)

Table 2c. Mental illness

Indicator	Achievement		Variation in ORs between prisons (range of ORs)	Association between explanatory variables and achievement (OR; 95% CI)									
	2019-20 (%)	2019-20 compared to 2017-18 (OR; 95% CI)		Prison category[1] (comparator category A)		Gender[2] (comparator female)		Age (comparator 30-39 years)[2]		Length of stay (months)[2] (comparator 1-<6 months)		Ethnic group[2] (comparator White people)	
				Higher	Lower	Higher	Lower	Higher	Lower	Higher	Lower	Higher	Lower
Antipsychotic monitoring: proportion of the prison population with ≥ three prescriptions for a first or second generation antipsychotic drug, and ≥ five of these measurements in the preceding 12 months: body mass index, blood pressure, blood tests for fasting glucose and/or HbA1c, lipid profile, renal and liver function, full blood count	24 of 463 eligible people (5·2%)	Decrease (0·13; 0·07, 0·24)	12·57-fold (0·68 to 8·55)	··	B (0·07; 0·04, 0·13)<br><br>C (0·14; 0·08, 0·24)<br><br>Closed (0·21; 0·12, 0·36)	··	··	··	··	> 24 (3·48; 1·66, 7·31)	··	Asian/Asian British (5·67; 1·84, 17·46)<br><br>Black/Black British (4·04; 1·12, 14·54)	··
Polypharmacy, prescribed three or psychotropic drugs: proportion of the prison population prescribed ≥ three psychotropic drugs (antipsychotics, sedative antidepressants, hypnotics and anxiolytics, pregabalin or gabapentin, opioids) at the same time during an eight-week period	216 of 25,811 eligible people (0·8%)	Increase (1·76; 1·37, 2·25)	11·9-fold (0·25 to 2·94)	Closed (3·33; 2·3, 4·82)	D (0·35; 0·2, 0·64)	··	··	··	20-29 (0·51; 0·38, 0·69)	6-12 (1·64; 1·26, 2·14)<br><br>12-24 (2·16; 1·64, 2·85)	< 1 (0·43; 0·31, 0·62)	··	Asian/Asian British (0·22; 0·07, 0·69)

Indicator	Achievement		Variation in ORs between prisons (range of ORs)	Association between explanatory variables and achievement (OR; 95% CI)									
	2019-20 (%)	2019-20 compared to 2017-18 (OR; 95% CI)		Prison category[1] (comparator category A)		Gender[2] (comparator female)		Age (comparator 30-39 years)[2]		Length of stay (months)[2] (comparator 1-<6 months)		Ethnic group[2] (comparator White people)	
				**Higher**	**Lower**	**Higher**	**Lower**	**Higher**	**Lower**	**Higher**	**Lower**	**Higher**	**Lower**
Polypharmacy, prescribed four or psychotropic drugs: proportion of the prison population prescribed ≥ four psychotropic drugs at the same time during an eight-week period	103 of 25,811 eligible people (0·4%)	Increase (2·30; 1·56, 3·39)	33-fold (0·10 to 3·30)	Closed (6·22; 3·3, 11·72)	D (0·07; 0·01, 0·57)	··	··	··	20-29 (0·56; 0·36, 0·87)	6-12 (2·19; 1·48, 3·24)<br><br>12-24 (2·82; 1·84, 4·32)<br><br>> 24 (1·92; 1·07, 3·42)	< 1 (0·38; 0·21, 0·67)	··	··
Mental state examination: proportion of the prison population over 55 years with a record of one of these assessments in the preceding 12 months: Mini Mental State Examination, 6 item Cognitive Impairment Test, General practitioner Assessment of Cognition	370 of 1,884 eligible people (19·6%)	Increase (40·47; 25·34, 64·64)	168·9-fold (0·65 to 109·76)	B (8·41; 5·79, 12·2)<br><br>Closed (2·52; 1·48, 4·3)<br><br>D (6·87; 4·44, 10·64)	··	··	··	··	··	··	··	··	··
Diagnosis of dementia: proportion of the prison population with a new diagnosis of any form of dementia, and a record of ≥ five of these blood tests 12 months before to 6 months after date of	6 of 13 eligible people (46·2%)	[3]	[3]	[3]	[3]	[3]	[3]	[3]	[3]	[3]	[3]	[3]	[3]

Indicator	Achievement		Variation in ORs between prisons (range of ORs)	Association between explanatory variables and achievement (OR; 95% CI)									
	2019-20 (%)	2019-20 compared to 2017-18 (OR; 95% CI)		Prison category[1] (comparator category A)		Gender[2] (comparator female)		Age (comparator 30-39 years)[2]		Length of stay (months)[2] (comparator 1-<6 months)		Ethnic group[2] (comparator White people)	
				Higher	Lower	Higher	Lower	Higher	Lower	Higher	Lower	Higher	Lower
diagnosis: full blood count, calcium, glucose and/or HbA1C, renal and liver function, thyroid function, vitamin B12, folate													

[1] Univariate analysis

[2] Multivariable analysis

[3] Insufficient data for multivariable analysis

.. No statistically significant result (at 5%)

Table 2d. Communicable disease

Indicator	Achievement		Variation in ORs between prisons (range of ORs)	Association between explanatory variables and achievement (OR; 95% CI)									
	2019-20 (%)	2019-20 compared to 2017-18 (OR; 95% CI)		Prison category[1] (comparator category A)		Gender[2] (comparator female)		Age (comparator 30-39 years)[2]		Length of stay (months)[2] (comparator 1-<6 months)		Ethnic group[2] (comparator White people)	
				Higher	Lower	Higher	Lower	Higher	Lower	Higher	Lower	Higher	Lower
Dried blood spot testing: proportion of people who received new reception screening and accepted opt out dried blood spot testing for Hepatitis B, Hepatitis C and HIV	5,653 of 12,606 (44·8%)	Increase (212·13; 170·37, 264·13)	169-fold (0·05 to 8·45)	B (1·31; 1·1, 1·55)<br><br>C (2·95; 2·48, 3·51)<br><br>Closed (8·09; 6·68, 9·79)<br><br>D (2·94; 2·45, 3·54)<br><br>YOI (3·87; 3·1, 4·83)	··	··	··	··	··	6-12 (1·36; 1·22, 1·51)<br><br>12-24 (1·30; 1·14, 1·5)<br><br>> 24 (10·15; 6·73, 15·31)	< 1 (0·53; 0·48, 0·58)	··	··
Hepatitis B vaccination: proportion of new receptions to prison who have received at least one Hepatitis B vaccination in the preceding 12 months	8,210 of 17,957 eligible people (45·7%)	Increase (2·45; 2·32, 2·6)	3·9-fold (0·52 to 2·04)	Closed (1·47; 1·29, 1·67)<br><br>D (1·20; 1·05, 1·36)<br><br>YOI (1·63; 1·4, 1·91)	B (0·63; 0·56, 0·7)	··	··	··	10-19 (0·55; 0·46, 0·66)<br><br>40-49 (0·92; 0·86, 0·98)<br><br>50 -59 (0·84; 0·76, 0·91)<br><br>60-69 (0·75; 0·64, 0·88)<br><br>70-79 (0·58; 0·42, 0·79)<br><br>80-89 (0·48; 0·25, 0·92)	6–12 (1·14; 1·06, 1·22)<br><br>12-24 (1·65; 1·5, 1·8)<br><br>> 24 (1·68; 1·43, 1·98)	< 1 (0·76; 0·72, 0·81)	··	Asian/Asian British (0·86; 0·77, 0·97)<br><br>Black/Black British (0·76; 0·65, 0·9)<br><br>Chinese/Other (0·72; 0·57, 0·92)
Hepatitis B vaccination for people with a history of illicit drug use: proportion of	5,117 of 10,207 eligible	Increase (3·64; 3·37, 3·93)	10·9-fold (0·64 to 6·99)	Closed (1·76;	B (0·67;	··	··	··	10-19 (0·6; 0·47, 0·76)	6-12 (1·12;	< 1 (0·83;	··	Black/Black British (0·75; 0·59, 0·96)

Indicator	Achievement		Variation in ORs between prisons (range of ORs)	Association between explanatory variables and achievement (OR; 95% CI)									
	2019-20 (%)	2019-20 compared to 2017-18 (OR; 95% CI)		Prison category[1] (comparator category A)		Gender[2] (comparator female)		Age (comparator 30-39 years)[2]		Length of stay (months)[2] (comparator 1-<6 months)		Ethnic group[2] (comparator White people)	
				Higher	Lower	Higher	Lower	Higher	Lower	Higher	Lower	Higher	Lower
new receptions to prison with a history of illicit drug use who have received at least one Hepatitis B vaccination in the preceding 12 months	people (50·1%)			1·47, 2·12)<br><br>YOI (1·50; 1·21, 1·86)	0·57, 0·8)				40-49 (0·89; 0·82, 0·96)<br><br>50-59 (0·86; 0·76, 0·99)<br><br>60-69 (0·55; 0·39, 0·78)	1·03, 1·23)<br><br>12-24 (1·53; 1·36, 1·73)<br><br>> 24 (1·46; 1·15, 1·85)	0·77, 0·89)		
Influenza immunisation: proportion of the prison population with one or more of: coronary heart disease, stroke, transient ischemic attack, diabetes or chronic obstructive pulmonary disease, who received an influenza vaccination August to March	787 of 1,752 eligible people (44·9%)	Increase (1·22; 1·02, 1·45)	18·2-fold (0·13 to 2·36)	··	B (0·26; 0·22, 0·31)<br><br>C (0·82; 0·68, 0·99)<br><br>Closed (0·42; 0·34, 0·52)<br><br>D (0·60; 0·47, 0·76)<br><br>YOI (0·12; 0·03, 0·56)	··	··	··	··	6-12 (2·16; 1·74, 2·69)<br><br>12-24 (2·89; 2·29, 3·65)<br><br>> 24 (3·96; 3·09, 5·07)	< 1 (0·46; 0·35, 0·6)	··	Black/Black British (0·58; 0·34, 0·99)<br><br>Chinese/Other (0·40; 0·17, 0·92)

1 Univariate analysis

2 Multivariable analysis

·· No statistically significant result (at 5%)

Table 2e. Opioid and gabapentinoid prescribing

Indicator	Achievement		Variation in ORs between prisons (range of ORs)	Association between explanatory variables and achievement (OR; 95% CI)									
	2019-20 (%)	2019-20 compared to 2017-18 (OR; 95% CI)		Prison category[1] (comparator category A)		Gender [2] (comparator female)		Age[2] (comparator 30-39 years)		Length of stay (months)[2] (comparator 1-<6 months)		Ethnic group[2] (comparator White people)	
				Higher	Lower	Higher	Lower	Higher	Lower	Higher	Lower	Higher	Lower
Prescribed any opioid: proportion of the prison population prescribed any opioid during an eight-week period	2,957 of 25,811 eligible people (11·5%)	Increase (1·47; 1·38, 1·58)	5-fold (0·26 to 1·31)	C (1·30; 1·19, 1·43)<br><br>Closed (1·47; 1·32, 1·64)	B (0·73; 0·66, 0·8)<br><br>D (0·14; 0·11, 0·17)	··	··	40-49 (1·38; 1·29, 1·48)	10-19 (0·14; 0·07, 0·27)<br><br>20-29 (0·44; 0·41, 0·48)<br><br>60-69 (0·83; 0·71, 0·97)	6-12 (1·59; 1·47, 1·72)<br><br>12-24 (1·82; 1·67, 1·99)<br><br>> 24 (1·85; 1·67, 2·05)	< 1 (0·58; 0·53, 0·64)	··	Mixed (0·55; 0·43, 0·71)<br><br>Asian/Asian British (0·32; 0·25, 0·4)<br><br>Black/Black British (0·41; 0·31, 0·54)<br><br>Chinese/Other (0·31; 0·2, 0·48)
Prescribed any strong opioid: proportion of the prison population prescribed any strong opioid during an eight-week period	2,257 of 25,811 eligible people (8·7%)	Increase (1·22; 1·14, 1·32)	3·8 fold (0·36 to 1·36)	Closed (1·21; 1·09, 1·35)	B (0·47; 0·43, 0·52)<br><br>D (0·14; 0·11, 0·17)	··	··	40-49 (1·39; 1·3, 1·49)<br><br>50-59 (1·22; 1·11, 1·34)	10-19 (0·06; 0·02, 0·18)<br><br>20-29 (0·38; 0·34, 0·41)	6-12 (1·44; 1·32, 1·57)<br><br>12-24 (1·75; 1·59, 1·91)<br><br>> 24 (1·92; 1·73, 2·13)	< 1 (0·79; 0·72, 0·87)	··	Mixed (0·51; 0·39, 0·67)<br><br>Asian/Asian British (0·34; 0·26, 0·43)<br><br>Black/Black British (0·37; 0·28, 0·5)<br><br>Chinese/Other (0·33; 0·21, 0·51)
Prescribed any opioid with benzodiazepines: proportion of the prison population prescribed any opioid and benzodiazepines during an eight-week period	2,257 of 25,811 eligible people (8·7%)	Increase (1·18; 1·1, 1·27)	12-fold (0·39 to 4·68)	B (1·20; 1·1, 1·32)<br><br>Closed (1·24; 1·11, 1·38)	D (0·13; 0·11, 0·16)	··	··	40-49 (1·32; 1·24, 1·42)<br><br>50-59 (1·19; 1·09, 1·31)	10-19 (0·46; 0·34, 0·61)<br><br>20-29 (0·5; 0·47, 0·54)	6-12 (1·36; 1·26, 1·47)<br><br>12-24 (1·6; 1·47, 1·75)<br><br>> 24	< 1 (0·85; 0·78, 0·92)	··	Mixed (0·62; 0·5, 0·78)<br><br>Asian/Asian British (0·56; 0·48, 0·65)<br><br>Black/Black British (0·47; 0·37, 0·6)<br><br>Chinese/Other (0·48; 0·35, 0·66)

Indicator	Achievement		Variation in ORs between prisons (range of ORs)	Association between explanatory variables and achievement (OR; 95% CI)									
	2019-20 (%)	2019-20 compared to 2017-18 (OR; 95% CI)		Prison category[1] (comparator category A)		Gender [2] (comparator female)		Age[2] (comparator 30-39 years)		Length of stay (months)[2] (comparator 1-<6 months)		Ethnic group[2] (comparator White people)	
				Higher	Lower	Higher	Lower	Higher	Lower	Higher	Lower	Higher	Lower
										(1·82;<br>1·64,<br>2·02)			
People with any mental illness prescribed any opioid: proportion of the prison population with any mental illness prescribed any opioid<br>during an eight-week period	770 of 4,054 eligible people (19·0%)	Increase (1·64; 1·41, 1·91)	11·1-fold (0·27 to 3·0)	C (2·04; 1·62, 2·58)<br><br>Closed (1·60; 1·26, 2·03)	··	··	··	40-49 (1·4; 1·21, 1·61)<br><br>50-59 (1·35; 1·11, 1·63)	10-19 (0·24; 0·03, 1·89)<br><br>20-29 (0·42; 0·35, 0·51)<br><br>70-79 (0·47; 0·23, 0·95)	6-12 (1·71; 1·46, 2·02)<br><br>12-24 (2·01; 1·67, 2·42)<br><br>> 24 (1·95; 1·56, 2·43)	< 1 (0·66; 0·54, 0·81)	··	Mixed (0·58; 0·36, 0·95)<br><br>Asian/Asian British (0·25; 0·13, 0·48)
No diagnosis of neuropathic pain and prescribed a gabapentinoid: proportion of the prison population with no coded diagnosis of neuropathic pain prescribed pregabalin or gabapentin<br>during an eight-week period	204 of 24,388 eligible people (0·8%)	Decrease (0·89; 0·71, 1·1)	1·93-fold (0·67 to 1·29)	··	B (0·40; 0·32, 0·5)<br><br>C (0·27; 0·21, 0·35)<br><br>D (0·18; 0·12, 0·28)	··	··	40-49 (1·77; 1·43, 2·19)<br><br>50-59 (1·7; 1·31, 2·21)<br><br>60-69 (1·54; 1·02, 2·31)<br><br>90-99 (12·22; 1·41, 105·56)	20-29 (0·25; 0·17, 0·36)	6-12 (1·77; 1·38, 2·29)<br><br>12-24 (1·91; 1·43, 2·54)<br><br>> 24 (1·91; 1·36, 2·67)	< 1 (0·51; 0·37, 0·7)	··	Mixed (0·33; 0·12, 0·9)<br><br>Asian/Asian British (0·18; 0·07, 0·48)<br><br>Black/Black British (0·22; 0·07, 0·69)

1 Univariate analysis

2 Multivariable analysis

·· No statistically significant result (at 5%)

Table 2f. Prison specific

Indicator	Achievement		Variation in ORs between prisons (range of Ors)	Association between explanatory variables and achievement (OR; 95% CI)									
	2019-20 (%)	2019-20 compared to 2017-18 (OR; 95% CI)		Prison category[1] (comparator category A)		Gender[2] (comparator female)		Age[2] (comparator 30-39 years)		Length of stay (months)[2] (comparator 1-<6 months)		Ethnic group[2] (comparator White people)	
				Higher	Lower	Higher	Lower	Higher	Lower	Higher	Lower	Higher	Lower
Consent to transfer medical records: proportion of new receptions to prison who have been asked for consent to transfer medical records from community primary care to prison healthcare	12,639 of 17,957 eligible people (70·4%)	Increase (4·28; 3·96, 4·62)	337·1-fold (0·007 to 2·36)	C (1·43; 1·26, 1·64)<br><br>Closed (2·86; 2·44, 3·35)<br><br>YOI (3·88; 3·08, 4·89)	B (0·38; 0·33, 0·43)	··	Men (0·1; 0·02, 0·14)	··	50-59 (0·86; 0·76, 0·97)<br><br>60-69 (0·72; 0·58, 0·89)	6 -12 (1·22; 1·11, 1·34)<br><br>12-24 (1·43; 1·25, 1·63)<br><br>> 24 (1·85; 1·47, 2·31)	< 1 (0·77; 0·71, 0·83)	··	Mixed (0·80; 0·65, 0·99)<br><br>Asian/Asian British (0·80; 0·69, 0·92)<br><br>Black/Black British (0·75; 0·61, 0·93)<br><br>Chinese/Other (0·70; 0·52, 0·96)
Medicine reconciliation and in-possession risk assessment: proportion of new receptions to prison who had a second-stage health assessment, plus in-possession risk assessment [a] and medicines reconciliation [b], accurately communicated in the week before that assessment<br><br>[a] In-possession risk assessment: a standardised assessment of whether it is safe for medication to be held in the	2,359 of 6,173 eligible people (38·2%)	[4]	21,610-fold (0·45 to 9724·5)	B (5·91; 4·81, 7·25)<br><br>D (1·70; 1·33, 2·16)	C (0·43; 0·35, 0·52)<br><br>YOI (0·02; 0·01, 0·06)	··	··	··	··	< 1 (3·02; 1·86, 4·89)<br><br>6-12 (3·17; 2·26, 4·44)<br><br>> 24 (1·54; 1·01, 2·33)	··	··	··

possession of the prisoner.<br>[b] Medicines reconciliation: identifying a n accurate list of a person's current medicines and comparing them with what medications are being prescribed, recognising discrepancies, and documenting changes.													

[1] Univariate analysis

[2] Multivariable analysis

[4] Data only available for 2019-20

.. No statistically significant result (at 5%)

Supplementary section 2: Descriptive statistics for each indicator

Indicator ***The proportion of people with diabetes in whom the last blood pressure reading (measured in the preceding 12 months) is 140/80 mmHg or less***

Group: Diabetes, Asthma & Epilepsy Care

Community achievement 2019/20: 68.01% (QOF 19/20 - for patients without moderate or severe frailty only)

	Variable	Population	Eligible[1] (% popln)	Satisfy[2] (% eligible)	Elsewhere[3] (% eligible)	Achieve[4] (% eligible)
**Year**						
	2017/18	21,677	675 (3.1)	230 (34.1)	<10 [6]	232 (34.4)
	2018/19	22,099	713 (3.2)	237 (33.2)	<10 [6]	240 (33.7)
	2019/20	25,811	770 (3.0)	256 (33.2)	<10 [6]	261 (33.9)
**Prison**						
2017/18	Prison 1	1,323	25 (1.9)	4 (16.0)	<10 [6]	<10 [6]
	Prison 2	3,261	96 (2.9)	35 (36.5)	<10 [6]	36 (37.5)
	Prison 3	2,623	70 (2.7)	20 (28.6)	<10 [6]	20 (28.6)
	Prison 4	2,089	68 (3.3)	41 (60.3)	<10 [6]	41 (60.3)
	Prison 5	637	22 (3.5)	<10 [6]	<10 [6]	<10 [6]
	Prison 6	1,552	54 (3.5)	23 (42.6)	<10 [6]	23 (42.6)
	Prison 7	635	21 (3.3)	<10 [6]	<10 [6]	<10 [6]
	Prison 8	1,085	29 (2.7)	14 (48.3)	<10 [6]	14 (48.3)
	Prison 9	981	86 (8.8)	22 (25.6)	<10 [6]	22 (25.6)
	Prison 10	2,523	75 (3.0)	24 (32.0)	<10 [6]	24 (32.0)
	Prison 11	3,470	83 (2.4)	14 (16.9)	<10 [6]	14 (16.9)
	Prison 12	815	<10 [6]	<10 [6]	<10 [6]	<10 [6]
	Prison 13	683	42 (6.1)	15 (35.7)	<10 [6]	15 (35.7)
2018/19	Prison 1	1,333	19 (1.4)	11 (57.9)	<10 [6]	11 (57.9)
	Prison 2	2,705	73 (2.7)	11 (15.1)	<10 [6]	11 (15.1)
	Prison 3	2,522	85 (3.4)	33 (38.8)	<10 [6]	33 (38.8)
	Prison 4	2,349	76 (3.2)	28 (36.8)	<10 [6]	28 (36.8)
	Prison 5	676	22 (3.3)	8 (36.4)	<10 [6]	<10 [6]
	Prison 6	1,513	48 (3.2)	22 (45.8)	<10 [6]	22 (45.8)
	Prison 7	654	19 (2.9)	<10 [6]	<10 [6]	<10 [6]
	Prison 8	1,148	34 (3.0)	23 (67.6)	<10 [6]	23 (67.6)
	Prison 9	996	105 (10.5)	25 (23.8)	<10 [6]	25 (23.8)
	Prison 10	2,717	77 (2.8)	32 (41.6)	<10 [6]	34 (44.2)
	Prison 11	4,020	113 (2.8)	21 (18.6)	<10 [6]	22 (19.5)
	Prison 12	792	<10 [6]	<10 [6]	<10 [6]	<10 [6]
	Prison 13	674	39 (5.8)	15 (38.5)	<10 [6]	15 (38.5)
2019/20	Prison 1	1,410	37 (2.6)	18 (48.6)	<10 [6]	18 (48.6)
	Prison 2	2,979	77 (2.6)	19 (24.7)	<10 [6]	19 (24.7)
	Prison 3	2,809	88 (3.1)	40 (45.5)	<10 [6]	40 (45.5)
	Prison 4	2,651	<10 [6]	<10 [6]	<10 [6]	<10 [6]
	Prison 5	616	25 (4.1)	<10 [6]	<10 [6]	<10 [6]
	Prison 6	1,533	51 (3.3)	11 (21.6)	<10 [6]	11 (21.6)
	Prison 7	860	29 (3.4)	12 (41.4)	<10 [6]	12 (41.4)
	Prison 8	1,385	44 (3.2)	29 (65.9)	<10 [6]	29 (65.9)
	Prison 9	1,092	128 (11.7)	26 (20.3)	<10 [6]	29 (22.7)
	Prison 10	3,577	97 (2.7)	42 (43.3)	<10 [6]	42 (43.3)
	Prison 11	5,348	138 (2.6)	38 (27.5)	<10 [6]	38 (27.5)
	Prison 12	805	<10 [6]	<10 [6]	<10 [6]	<10 [6]
	Prison 13	746	46 (6.2)	12 (26.1)	<10 [6]	13 (28.3)
**Prison category**						
2017/18	A	1,664	128 (7.7)	37 (28.9)	<10 [6]	37 (28.9)
	B	9,254	254 (2.7)	73 (28.7)	<10 [6]	74 (29.1)
	C	6,035	163 (2.7)	65 (39.9)	<10 [6]	65 (39.9)
	Closed	1,720	50 (2.9)	21 (42.0)	<10 [6]	21 (42.0)
	D	2,189	76 (3.5)	31 (40.8)	<10 [6]	32 (42.1)
	YOI	815	<10 [6]	<10 [6]	<10 [6]	<10 [6]
2018/19	A	1,670	144 (8.6)	40 (27.8)	<10 [6]	40 (27.8)
	B	9,442	263 (2.8)	64 (24.3)	<10 [6]	67 (25.5)
	C	6,204	180 (2.9)	72 (40.0)	<10 [6]	72 (40.0)
	Closed	1,802	53 (2.9)	29 (54.7)	<10 [6]	29 (54.7)
	D	2,189	70 (3.2)	30 (42.9)	<10 [6]	30 (42.9)
	YOI	792	<10 [6]	<10 [6]	<10 [6]	<10 [6]
2019/20	A	1,838	174 (9.5)	38 (21.8)	<10 [6]	42 (24.1)
	B	11,904	312 (2.6)	99 (31.7)	<10 [6]	99 (31.7)
	C	6,870	131 (1.9)	59 (45.0)	<10 [6]	59 (45.0)
	Closed	2,245	73 (3.3)	41 (56.2)	<10 [6]	41 (56.2)
	D	2,149	76 (3.5)	19 (25.0)	<10 [6]	20 (26.3)
	YOI	805	<10 [6]	<10 [6]	<10 [6]	<10 [6]
**Gender**						
2017/18	F	1,699	50 (2.9)	21 (42.0)	<10 [6]	21 (42.0)
	M	19,977	625 (3.1)	209 (33.4)	<10 [6]	211 (33.8)
2018/19	F	1,802	52 (2.9)	29 (55.8)	<10 [6]	29 (55.8)
	M	20,295	661 (3.3)	208 (31.5)	<10 [6]	211 (31.9)
2019/20	F	2,240	73 (3.3)	41 (56.2)	<10 [6]	41 (56.2)
	M	23,570	697 (3.0)	215 (30.8)	<10 [6]	220 (31.6)

[1]Eligible for indicator; [2]Achieve in current prison; [3]Achieve in previous prison; [4]Overall achievement - either current or previous prison; [5]Declined indicator; [6]Suppressed (<10) to avoid disclosure

Supplementary section 2: Descriptive statistics for each indicator

Indicator ***The proportion of people with diabetes in whom the last blood pressure reading (measured in the preceding 12 months) is 140/80 mmHg or less***

Group: Diabetes, Asthma & Epilepsy Care

Community achievement 2019/20: 68.01% (QOF 19/20 - for patients without moderate or severe frailty only)

	Variable	Population	Eligible[1] (% popln)	Satisfy[2] (% eligible)	Elsewhere[3] (% eligible)	Achieve[4] (% eligible)
**Sentence Status**						
2017/18	.	81	<10 [6]	<10 [6]	<10 [6]	<10 [6]
	Absconded	<10 [6]	<10 [6]	<10 [6]	<10 [6]	<10 [6]
	Active In	15,872	500 (3.2)	175 (35.0)	<10 [6]	176 (35.2)
	Active Out	1,052	30 (2.9)	<10 [6]	<10 [6]	<10 [6]
	Convicted Sentence	2,125	59 (2.8)	21 (35.6)	<10 [6]	21 (35.6)
	Downgrade in security category	<10 [6]	<10 [6]	<10 [6]	<10 [6]	<10 [6]
	Internal Cell Move	<10 [6]	<10 [6]	<10 [6]	<10 [6]	<10 [6]
	Judges Remand	92	<10 [6]	<10 [6]	<10 [6]	<10 [6]
	Licence Revoke	52	<10 [6]	<10 [6]	<10 [6]	<10 [6]
	On Remand	1,492	47 (3.2)	10 (21.3)	<10 [6]	11 (23.4)
	Transfer	910	31 (3.4)	13 (41.9)	<10 [6]	13 (41.9)
	Upgrade in security category	<10 [6]	<10 [6]	<10 [6]	<10 [6]	<10 [6]
2018/19	.	88	<10 [6]	<10 [6]	<10 [6]	<10 [6]
	Absconded	<10 [6]	<10 [6]	<10 [6]	<10 [6]	<10 [6]
	Active In	18,145	589 (3.2)	204 (34.6)	<10 [6]	207 (35.1)
	Active Out	835	34 (4.1)	14 (41.2)	<10 [6]	14 (41.2)
	Convicted Sentence	1,320	38 (2.9)	<10 [6]	<10 [6]	<10 [6]
	Downgrade in security category	<10 [6]	<10 [6]	<10 [6]	<10 [6]	<10 [6]
	Internal Cell Move	<10 [6]	<10 [6]	<10 [6]	<10 [6]	<10 [6]
	Judges Remand	<10 [6]	<10 [6]	<10 [6]	<10 [6]	<10 [6]
	Licence Revoke	125	<10 [6]	<10 [6]	<10 [6]	<10 [6]
	On Remand	1,059	25 (2.4)	<10 [6]	<10 [6]	<10 [6]
	Transfer	518	20 (3.9)	<10 [6]	<10 [6]	<10 [6]
	Upgrade in security category	<10 [6]	<10 [6]	<10 [6]	<10 [6]	<10 [6]
2019/20	.	69	<10 [6]	<10 [6]	<10 [6]	<10 [6]
	Absconded	<10 [6]	<10 [6]	<10 [6]	<10 [6]	<10 [6]
	Active In	22,424	674 (3.0)	226 (33.5)	<10 [6]	231 (34.3)
	Active Out	625	26 (4.2)	13 (50.0)	<10 [6]	13 (50.0)
	Convicted Sentence	1,361	36 (2.6)	<10 [6]	<10 [6]	<10 [6]
	Downgrade in security category	<10 [6]	<10 [6]	<10 [6]	<10 [6]	<10 [6]
	Internal Cell Move	<10 [6]	<10 [6]	<10 [6]	<10 [6]	<10 [6]
	Judges Remand	19	<10 [6]	<10 [6]	<10 [6]	<10 [6]
	Licence Revoke	178	<10 [6]	<10 [6]	<10 [6]	<10 [6]
	On Remand	1,031	23 (2.2)	<10 [6]	<10 [6]	<10 [6]
	Transfer	101	<10 [6]	<10 [6]	<10 [6]	<10 [6]
	Upgrade in security category	<10 [6]	<10 [6]	<10 [6]	<10 [6]	<10 [6]
**Age - years**						
2017/18	10 - <20	468	<10 [6]	<10 [6]	<10 [6]	<10 [6]
	20 - <30	6,994	71 (1.0)	40 (56.3)	<10 [6]	41 (57.7)
	30 - <40	7,051	101 (1.4)	40 (39.6)	<10 [6]	40 (39.6)
	40 - <50	4,114	140 (3.4)	38 (27.1)	<10 [6]	38 (27.1)
	50 - <60	2,107	194 (9.2)	66 (34.0)	<10 [6]	66 (34.0)
	60 - <70	684	109 (15.9)	27 (24.8)	<10 [6]	28 (25.7)
	70 - <80	213	47 (22.1)	15 (31.9)	<10 [6]	15 (31.9)
	80 - <90	40	10 (25.0)	<10 [6]	<10 [6]	<10 [6]
	90 - <100	<10 [6]	<10 [6]	<10 [6]	<10 [6]	<10 [6]
	100 - <110	<10 [6]	<10 [6]	<10 [6]	<10 [6]	<10 [6]
2018/19	10 - <20	436	<10 [6]	<10 [6]	<10 [6]	<10 [6]
	20 - <30	7,163	53 (0.7)	24 (45.3)	<10 [6]	26 (49.1)
	30 - <40	7,381	108 (1.5)	43 (39.8)	<10 [6]	43 (39.8)
	40 - <50	4,180	148 (3.5)	40 (27.0)	<10 [6]	40 (27.0)
	50 - <60	1,978	212 (10.7)	63 (29.7)	<10 [6]	63 (29.7)
	60 - <70	701	131 (18.7)	42 (32.1)	<10 [6]	43 (32.8)
	70 - <80	209	47 (22.5)	15 (31.9)	<10 [6]	15 (31.9)
	80 - <90	45	13 (28.9)	<10 [6]	<10 [6]	<10 [6]
	90 - <100	<10 [6]	<10 [6]	<10 [6]	<10 [6]	<10 [6]
	100 - <110	<10 [6]	<10 [6]	<10 [6]	<10 [6]	<10 [6]
2019/20	10 - <20	404	<10 [6]	<10 [6]	<10 [6]	<10 [6]
	20 - <30	8,064	60 (0.7)	30 (50.0)	<10 [6]	30 (50.0)
	30 - <40	9,125	100 (1.1)	40 (40.0)	<10 [6]	40 (40.0)
	40 - <50	4,948	195 (3.9)	65 (33.3)	<10 [6]	67 (34.4)
	50 - <60	2,224	193 (8.7)	56 (29.0)	<10 [6]	57 (29.5)
	60 - <70	751	152 (20.2)	39 (25.7)	<10 [6]	40 (26.3)
	70 - <80	238	57 (23.9)	21 (36.8)	<10 [6]	22 (38.6)
	80 - <90	53	11 (20.8)	<10 [6]	<10 [6]	<10 [6]
	90 - <100	<10 [6]	<10 [6]	<10 [6]	<10 [6]	<10 [6]
	100 - <110	<10 [6]	<10 [6]	<10 [6]	<10 [6]	<10 [6]

[1]Eligible for indicator; [2]Achieve in current prison; [3]Achieve in previous prison; [4]Overall achievement - either current or previous prison; [5]Declined indicator; [6]Suppressed (<10) to avoid disclosure

Supplementary section 2: Descriptive statistics for each indicator

Indicator ***The proportion of people with diabetes in whom the last blood pressure reading (measured in the preceding 12 months) is 140/80 mmHg or less***

Group: Diabetes, Asthma & Epilepsy Care

Community achievement 2019/20: 68.01% (QOF 19/20 - for patients without moderate or severe frailty only)

	Variable	Population	Eligible[1] (% popln)	Satisfy[2] (% eligible)	Elsewhere[3] (% eligible)	Achieve[4] (% eligible)
**Length of Stay (months)**						
2017/18	<1	4,474	100 (2.2)	31 (31.0)	<10 [6]	32 (32.0)
	1-<6	8,075	187 (2.3)	58 (31.0)	<10 [6]	59 (31.6)
	6-<12	3,672	107 (2.9)	37 (34.6)	<10 [6]	37 (34.6)
	12-<24	2,832	98 (3.5)	38 (38.8)	<10 [6]	38 (38.8)
	24+	2,624	183 (7.0)	66 (36.1)	<10 [6]	66 (36.1)
2018/19	<1	4,801	98 (2.0)	22 (22.4)	<10 [6]	23 (23.5)
	1-<6	7,742	184 (2.4)	63 (34.2)	<10 [6]	65 (35.3)
	6-<12	3,616	104 (2.9)	35 (33.7)	<10 [6]	35 (33.7)
	12-<24	3,752	138 (3.7)	41 (29.7)	<10 [6]	41 (29.7)
	24+	2,188	189 (8.6)	76 (40.2)	<10 [6]	76 (40.2)
2019/20	<1	6,764	164 (2.4)	65 (39.6)	<10 [6]	65 (39.6)
	1-<6	10,802	254 (2.4)	86 (33.9)	<10 [6]	88 (34.6)
	6-<12	3,893	106 (2.7)	30 (28.3)	<10 [6]	33 (31.1)
	12-<24	2,600	96 (3.7)	31 (32.3)	<10 [6]	31 (32.3)
	24+	1,752	150 (8.6)	44 (29.3)	<10 [6]	44 (29.3)
**Ethnic Group**						
2017/18	White	15,638	529 (3.4)	185 (35.0)	<10 [6]	187 (35.3)
	Mixed	431	15 (3.5)	<10 [6]	<10 [6]	<10 [6]
	Asian or Asian British	813	28 (3.4)	<10 [6]	<10 [6]	<10 [6]
	Black or Black British	404	15 (3.7)	<10 [6]	<10 [6]	<10 [6]
	Chinese and Other	214	<10 [6]	<10 [6]	<10 [6]	<10 [6]
	Unclassified	372	13 (3.5)	<10 [6]	<10 [6]	<10 [6]
2018/19	White	14,911	510 (3.4)	162 (31.8)	<10 [6]	165 (32.4)
	Mixed	371	10 (2.7)	<10 [6]	<10 [6]	<10 [6]
	Asian or Asian British	726	31 (4.3)	<10 [6]	<10 [6]	<10 [6]
	Black or Black British	364	15 (4.1)	<10 [6]	<10 [6]	<10 [6]
	Chinese and Other	167	<10 [6]	<10 [6]	<10 [6]	<10 [6]
	Unclassified	409	<10 [6]	<10 [6]	<10 [6]	<10 [6]
2019/20	White	16,606	499 (3.0)	171 (34.3)	<10 [6]	175 (35.1)
	Mixed	409	11 (2.7)	<10 [6]	<10 [6]	<10 [6]
	Asian or Asian British	755	46 (6.1)	14 (30.4)	<10 [6]	14 (30.4)
	Black or Black British	451	21 (4.7)	<10 [6]	<10 [6]	<10 [6]
	Chinese and Other	163	<10 [6]	<10 [6]	<10 [6]	<10 [6]
	Unclassified	387	<10 [6]	<10 [6]	<10 [6]	<10 [6]

[1]Eligible for indicator; [2]Achieve in current prison; [3]Achieve in previous prison; [4]Overall achievement - either current or previous prison; [5]Declined indicator; [6]Suppressed (<10) to avoid disclosure

Indicator	The proportion of people with diabetes who have had the following in the preceding 12 months: body mass index, blood pressure, record of smoking status, foot examination, urine albumin: creatinine ratio blood tests for HbA1c, cholesterol and serum creatinine
	Group: Diabetes, Asthma & Epilepsy Care
	68.01% of those without moderate or severe frailty have BP of 140/80 or less, 56.66% of those without moderate or severe frailty have HbA1c of 58 or less, 83.34% of those with moderate or severe frailty have HbA1c of 75 or less, 78.04% had foot examination with risk classification. No comparisons for other examinations.

				BMI (% eligible)		BP (% eligible)			Hb1Ac (% eligible)		Chol (% eligible)	
	Variable	Population	Eligible[1] (% popln)	Satisfy[2]	Elsewhere[3]	Satisfy[2]	Elsewhere[3]	Declined[5]	Satisfy[2]	Elsewhere[3]	Satisfy[2]	Elsewhere[3]
**Year**												
	2017/18	21,677	675 (3.1)	490 (72.6)	<10 [6]	548 (81.2)	25 (3.7)	<10 [6]	388 (57.5)	43 (6.4)	313 (46.4)	36 (5.3)
	2018/19	22,099	713 (3.2)	589 (82.6)	<10 [6]	624 (87.5)	12 (1.7)	<10 [6]	429 (60.2)	30 (4.2)	363 (50.9)	27 (3.8)
	2019/20	25,811	770 (3.0)	658 (85.5)	<10 [6]	718 (93.2)	<10 [6]	<10 [6]	470 (61.0)	32 (4.2)	408 (53.0)	21 (2.7)
**Prison**												
2017/18	Prison 1	1,323	25 (1.9)	22 (88.0)	<10 [6]	21 (84.0)	<10 [6]	<10 [6]	16 (64.0)	<10 [6]	13 (52.0)	<10 [6]
	Prison 2	3,261	96 (2.9)	68 (70.8)	<10 [6]	82 (85.4)	<10 [6]	<10 [6]	50 (52.1)	<10 [6]	38 (39.6)	<10 [6]
	Prison 3	2,623	70 (2.7)	57 (81.4)	<10 [6]	60 (85.7)	<10 [6]	<10 [6]	34 (48.6)	17 (24.3)	18 (25.7)	13 (18.6)
	Prison 4	2,089	68 (3.3)	51 (75.0)	<10 [6]	54 (79.4)	<10 [6]	<10 [6]	48 (70.6)	<10 [6]	44 (64.7)	<10 [6]
	Prison 5	637	22 (3.5)	15 (68.2)	<10 [6]	16 (72.7)	<10 [6]	<10 [6]	12 (54.5)	<10 [6]	<10 [6]	<10 [6]
	Prison 6	1,552	54 (3.5)	46 (85.2)	<10 [6]	53 (98.1)	<10 [6]	<10 [6]	41 (75.9)	<10 [6]	36 (66.7)	<10 [6]
	Prison 7	635	21 (3.3)	19 (90.5)	<10 [6]	19 (90.5)	<10 [6]	<10 [6]	8 (38.1)	<10 [6]	11 (52.4)	<10 [6]
	Prison 8	1,085	29 (2.7)	19 (65.5)	<10 [6]	24 (82.8)	<10 [6]	<10 [6]	15 (51.7)	<10 [6]	10 (34.5)	<10 [6]
	Prison 9	981	86 (8.8)	62 (72.1)	<10 [6]	73 (84.9)	<10 [6]	<10 [6]	73 (84.9)	<10 [6]	66 (76.7)	<10 [6]
	Prison 10	2,523	75 (3.0)	39 (52.0)	<10 [6]	62 (82.7)	<10 [6]	<10 [6]	25 (33.3)	<10 [6]	19 (25.3)	<10 [6]
	Prison 11	3,470	83 (2.4)	54 (65.1)	<10 [6]	43 (51.8)	<10 [6]	<10 [6]	27 (32.5)	<10 [6]	13 (15.7)	<10 [6]
	Prison 12	815	<10 [6]	<10 [6]	<10 [6]	<10 [6]	<10 [6]	<10 [6]	<10 [6]	<10 [6]	<10 [6]	<10 [6]
	Prison 13	683	42 (6.1)	34 (81.0)	<10 [6]	37 (88.1)	<10 [6]	<10 [6]	35 (83.3)	<10 [6]	35 (83.3)	<10 [6]
2018/19	Prison 1	1,333	19 (1.4)	16 (84.2)	<10 [6]	17 (89.5)	<10 [6]	<10 [6]	17 (89.5)	<10 [6]	16 (84.2)	<10 [6]
	Prison 2	2,705	73 (2.7)	60 (82.2)	<10 [6]	66 (90.4)	<10 [6]	<10 [6]	34 (46.6)	<10 [6]	26 (35.6)	<10 [6]
	Prison 3	2,522	85 (3.4)	69 (81.2)	<10 [6]	74 (87.1)	<10 [6]	<10 [6]	52 (61.2)	<10 [6]	45 (52.9)	<10 [6]
	Prison 4	2,349	76 (3.2)	58 (76.3)	<10 [6]	43 (56.6)	<10 [6]	<10 [6]	54 (71.1)	<10 [6]	54 (71.1)	<10 [6]
	Prison 5	676	22 (3.3)	12 (54.5)	<10 [6]	17 (77.3)	<10 [6]	<10 [6]	12 (54.5)	<10 [6]	11 (50.0)	<10 [6]
	Prison 6	1,513	48 (3.2)	43 (89.6)	<10 [6]	48 (100.0)	<10 [6]	<10 [6]	32 (66.7)	<10 [6]	29 (60.4)	<10 [6]
	Prison 7	654	19 (2.9)	16 (84.2)	<10 [6]	18 (94.7)	<10 [6]	<10 [6]	14 (73.7)	<10 [6]	10 (52.6)	<10 [6]
	Prison 8	1,148	34 (3.0)	24 (70.6)	<10 [6]	32 (94.1)	<10 [6]	<10 [6]	17 (50.0)	<10 [6]	<10 [6]	<10 [6]
	Prison 9	996	105 (10.5)	97 (92.4)	<10 [6]	100 (95.2)	<10 [6]	<10 [6]	95 (90.5)	<10 [6]	90 (85.7)	<10 [6]
	Prison 10	2,717	77 (2.8)	54 (70.1)	<10 [6]	62 (80.5)	<10 [6]	<10 [6]	33 (42.9)	<10 [6]	23 (29.9)	<10 [6]
	Prison 11	4,020	113 (2.8)	99 (87.6)	<10 [6]	106 (93.8)	<10 [6]	<10 [6]	30 (26.5)	<10 [6]	15 (13.3)	<10 [6]
	Prison 12	792	<10 [6]	<10 [6]	<10 [6]	<10 [6]	<10 [6]	<10 [6]	<10 [6]	12 (10.6)	<10 [6]	<10 [6]
	Prison 13	674	39 (5.8)	38 (97.4)	<10 [6]	38 (97.4)	<10 [6]	<10 [6]	36 (92.3)	<10 [6]	36 (92.3)	<10 [6]
2019/20	Prison 1	1,410	37 (2.6)	31 (83.8)	<10 [6]	33 (89.2)	<10 [6]	<10 [6]	24 (64.9)	<10 [6]	21 (56.8)	<10 [6]
	Prison 2	2,979	77 (2.6)	69 (89.6)	<10 [6]	72 (93.5)	<10 [6]	<10 [6]	50 (64.9)	<10 [6]	43 (55.8)	<10 [6]
	Prison 3	2,809	88 (3.1)	64 (72.7)	<10 [6]	84 (95.5)	<10 [6]	<10 [6]	58 (65.9)	<10 [6]	53 (60.2)	<10 [6]
	Prison 4	2,651	<10 [6]	<10 [6]	<10 [6]	<10 [6]	<10 [6]	<10 [6]	<10 [6]	<10 [6]	<10 [6]	<10 [6]
	Prison 5	616	25 (4.1)	21 (84.0)	<10 [6]	22 (88.0)	<10 [6]	<10 [6]	17 (68.0)	<10 [6]	11 (44.0)	<10 [6]
	Prison 6	1,533	51 (3.3)	49 (96.1)	<10 [6]	51 (100.0)	<10 [6]	<10 [6]	32 (62.7)	<10 [6]	28 (54.9)	<10 [6]
	Prison 7	860	29 (3.4)	25 (86.2)	<10 [6]	26 (89.7)	<10 [6]	<10 [6]	19 (65.5)	<10 [6]	19 (65.5)	<10 [6]
	Prison 8	1,385	44 (3.2)	35 (79.5)	<10 [6]	41 (93.2)	<10 [6]	<10 [6]	21 (47.7)	<10 [6]	12 (27.3)	<10 [6]
	Prison 9	1,092	128 (11.7)	121 (94.5)	<10 [6]	126 (98.4)	<10 [6]	<10 [6]	108 (84.4)	<10 [6]	105 (82.0)	<10 [6]
	Prison 10	3,577	97 (2.7)	68 (70.1)	<10 [6]	81 (83.5)	<10 [6]	<10 [6]	52 (53.6)	<10 [6]	42 (43.3)	<10 [6]
	Prison 11	5,348	138 (2.6)	125 (90.6)	<10 [6]	128 (92.8)	<10 [6]	<10 [6]	41 (29.7)	<10 [6]	27 (19.6)	<10 [6]
	Prison 12	805	<10 [6]	<10 [6]	<10 [6]	<10 [6]	<10 [6]	<10 [6]	<10 [6]	<10 [6]	<10 [6]	<10 [6]
	Prison 13	746	46 (6.2)	41 (89.1)	<10 [6]	45 (97.8)	<10 [6]	<10 [6]	42 (91.3)	<10 [6]	40 (87.0)	<10 [6]
**Prison category**												
2017/18	A	1,664	128 (7.7)	96 (75.0)	<10 [6]	110 (85.9)	<10 [6]	<10 [6]	108 (84.4)	<10 [6]	101 (78.9)	<10 [6]
	B	9,254	254 (2.7)	161 (63.4)	<10 [6]	187 (73.6)	<10 [6]	<10 [6]	102 (40.2)	13 (5.1)	70 (27.6)	10 (3.9)
	C	6,035	163 (2.7)	130 (79.8)	<10 [6]	135 (82.8)	<10 [6]	<10 [6]	98 (60.1)	22 (13.5)	75 (46.0)	17 (10.4)
	Closed	1,720	50 (2.9)	38 (76.0)	<10 [6]	43 (86.0)	<10 [6]	<10 [6]	23 (46.0)	<10 [6]	21 (42.0)	<10 [6]
	D	2,189	76 (3.5)	61 (80.3)	<10 [6]	69 (90.8)	<10 [6]	<10 [6]	53 (69.7)	<10 [6]	45 (59.2)	<10 [6]
	YOI	815	<10 [6]	<10 [6]	<10 [6]	<10 [6]	<10 [6]	<10 [6]	<10 [6]	<10 [6]	<10 [6]	<10 [6]
2018/19	A	1,670	144 (8.6)	135 (93.8)	<10 [6]	138 (95.8)	<10 [6]	<10 [6]	131 (91.0)	<10 [6]	126 (87.5)	<10 [6]
	B	9,442	263 (2.8)	213 (81.0)	<10 [6]	234 (89.0)	<10 [6]	<10 [6]	97 (36.9)	15 (5.7)	64 (24.3)	12 (4.6)
	C	6,204	180 (2.9)	143 (79.4)	<10 [6]	134 (74.4)	<10 [6]	<10 [6]	123 (68.3)	<10 [6]	115 (63.9)	<10 [6]
	Closed	1,802	53 (2.9)	40 (75.5)	<10 [6]	50 (94.3)	<10 [6]	<10 [6]	31 (58.5)	<10 [6]	16 (30.2)	<10 [6]
	D	2,189	70 (3.2)	55 (78.6)	<10 [6]	65 (92.9)	<10 [6]	<10 [6]	44 (62.9)	<10 [6]	40 (57.1)	<10 [6]
	YOI	792	<10 [6]	<10 [6]	<10 [6]	<10 [6]	<10 [6]	<10 [6]	<10 [6]	<10 [6]	<10 [6]	<10 [6]
2019/20	A	1,838	174 (9.5)	162 (93.1)	<10 [6]	171 (98.3)	<10 [6]	<10 [6]	150 (86.2)	<10 [6]	145 (83.3)	<10 [6]
	B	11,904	312 (2.6)	262 (84.0)	<10 [6]	281 (90.1)	<10 [6]	<10 [6]	143 (45.8)	<10 [6]	112 (35.9)	<10 [6]
	C	6,870	131 (1.9)	101 (77.1)	<10 [6]	123 (93.9)	<10 [6]	<10 [6]	87 (66.4)	10 (7.6)	80 (61.1)	<10 [6]
	Closed	2,245	73 (3.3)	60 (82.2)	<10 [6]	67 (91.8)	<10 [6]	<10 [6]	40 (54.8)	<10 [6]	31 (42.5)	<10 [6]
	D	2,149	76 (3.5)	70 (92.1)	<10 [6]	73 (96.1)	<10 [6]	<10 [6]	49 (64.5)	<10 [6]	39 (51.3)	<10 [6]
	YOI	805	<10 [6]	<10 [6]	<10 [6]	<10 [6]	<10 [6]	<10 [6]	<10 [6]	<10 [6]	<10 [6]	<10 [6]

[1]Eligible for indicator; [2]Achieve in current prison; [3]Achieve in previous prison; [4]Overall achievement - either current or previous prison; [5]Declined indicator; [6]Suppressed (<10) to avoid disclosure

Indicator	The proportion of people with diabetes who have had the following in the preceding 12 months: body mass index, blood pressure, record of smoking status, foot examination, urine albumin: creatinine ratio blood tests for HbA1c, cholesterol and serum creatinine<br>Group: Diabetes, Asthma & Epilepsy Care	*68.01% of those without moderate or severe frailty have BP of 140/80 or less, 56.66% of those without moderate or severe frailty have HbA1c of 58 or less, 83.34% of those with moderate or severe frailty have HbA1c of 75 or less, 78.04% had foot examination with risk classification. No comparisons for other examinations.*

			Smoke Status (% eligible)		Foot Exam (% eligible)			lbumin Creatinine Ratio (% eligible)		Serum Creatinine (% eligible)	
	Variable	Eligible[1]	Satisfy[2]	Elsewhere[3]	Satisfy[2]	Elsewhere[3]	Declined[5]	Satisfy[2]	Elsewhere[3]	Satisfy[2]	Elsewhere[3]
Year											
	2017/18	675	535 (79.3)	<10 [6]	235 (34.8)	23 (3.4)	<10 [6]	154 (22.8)	19 (2.8)	12 (1.8)	<10 [6]
	2018/19	713	595 (83.5)	16 (2.2)	250 (35.1)	26 (3.6)	<10 [6]	183 (25.7)	23 (3.2)	<10 [6]	<10 [6]
	2019/20	770	629 (81.7)	13 (1.7)	278 (36.1)	34 (4.4)	<10 [6]	172 (22.3)	22 (2.9)	<10 [6]	<10 [6]
**Prison**											
2017/18	Prison 1	25	20 (80.0)	<10 [6]	11 (44.0)	<10 [6]	<10 [6]	<10 [6]	<10 [6]	<10 [6]	<10 [6]
	Prison 2	96	72 (75.0)	<10 [6]	34 (35.4)	<10 [6]	<10 [6]	19 (19.8)	<10 [6]	<10 [6]	<10 [6]
	Prison 3	70	56 (80.0)	<10 [6]	22 (31.4)	<10 [6]	<10 [6]	24 (34.3)	<10 [6]	<10 [6]	<10 [6]
	Prison 4	68	50 (73.5)	<10 [6]	18 (26.5)	<10 [6]	<10 [6]	13 (19.1)	<10 [6]	<10 [6]	<10 [6]
	Prison 5	22	14 (63.6)	<10 [6]	<10 [6]	<10 [6]	<10 [6]	<10 [6]	<10 [6]	<10 [6]	<10 [6]
	Prison 6	54	47 (87.0)	<10 [6]	36 (66.7)	<10 [6]	<10 [6]	24 (44.4)	<10 [6]	<10 [6]	<10 [6]
	Prison 7	21	17 (81.0)	<10 [6]	<10 [6]	<10 [6]	<10 [6]	<10 [6]	<10 [6]	<10 [6]	<10 [6]
	Prison 8	29	25 (86.2)	<10 [6]	12 (41.4)	<10 [6]	<10 [6]	<10 [6]	<10 [6]	<10 [6]	<10 [6]
	Prison 9	86	74 (86.0)	<10 [6]	40 (46.5)	<10 [6]	<10 [6]	26 (30.2)	<10 [6]	<10 [6]	<10 [6]
	Prison 10	75	62 (82.7)	<10 [6]	16 (21.3)	<10 [6]	<10 [6]	<10 [6]	<10 [6]	<10 [6]	<10 [6]
	Prison 11	83	56 (67.5)	<10 [6]	<10 [6]	<10 [6]	<10 [6]	<10 [6]	<10 [6]	<10 [6]	<10 [6]
	Prison 12	<10 [6]	<10 [6]	<10 [6]	<10 [6]	<10 [6]	<10 [6]	<10 [6]	<10 [6]	<10 [6]	<10 [6]
	Prison 13	42	38 (90.5)	<10 [6]	34 (81.0)	<10 [6]	<10 [6]	32 (76.2)	<10 [6]	<10 [6]	<10 [6]
2018/19	Prison 1	19	15 (78.9)	<10 [6]	13 (68.4)	<10 [6]	<10 [6]	<10 [6]	<10 [6]	<10 [6]	<10 [6]
	Prison 2	73	65 (89.0)	<10 [6]	30 (41.1)	<10 [6]	<10 [6]	10 (13.7)	<10 [6]	<10 [6]	<10 [6]
	Prison 3	85	60 (70.6)	<10 [6]	26 (30.6)	<10 [6]	<10 [6]	17 (20.0)	<10 [6]	<10 [6]	<10 [6]
	Prison 4	76	55 (72.4)	<10 [6]	23 (30.3)	<10 [6]	<10 [6]	39 (51.3)	<10 [6]	<10 [6]	<10 [6]
	Prison 5	22	17 (77.3)	<10 [6]	<10 [6]	<10 [6]	<10 [6]	<10 [6]	<10 [6]	<10 [6]	<10 [6]
	Prison 6	48	41 (85.4)	<10 [6]	29 (60.4)	<10 [6]	<10 [6]	16 (33.3)	<10 [6]	<10 [6]	<10 [6]
	Prison 7	19	17 (89.5)	<10 [6]	<10 [6]	<10 [6]	<10 [6]	<10 [6]	<10 [6]	<10 [6]	<10 [6]
	Prison 8	34	25 (73.5)	<10 [6]	<10 [6]	<10 [6]	<10 [6]	<10 [6]	<10 [6]	<10 [6]	<10 [6]
	Prison 9	105	100 (95.2)	<10 [6]	68 (64.8)	<10 [6]	<10 [6]	40 (38.1)	<10 [6]	<10 [6]	<10 [6]
	Prison 10	77	61 (79.2)	<10 [6]	<10 [6]	<10 [6]	<10 [6]	<10 [6]	<10 [6]	<10 [6]	<10 [6]
	Prison 11	113	98 (86.7)	<10 [6]	<10 [6]	<10 [6]	<10 [6]	<10 [6]	<10 [6]	<10 [6]	<10 [6]
	Prison 12	<10 [6]	<10 [6]	<10 [6]	<10 [6]	<10 [6]	<10 [6]	<10 [6]	<10 [6]	<10 [6]	<10 [6]
	Prison 13	39	38 (97.4)	<10 [6]	24 (61.5)	<10 [6]	<10 [6]	32 (82.1)	<10 [6]	<10 [6]	<10 [6]
2019/20	Prison 1	37	31 (83.8)	<10 [6]	17 (45.9)	<10 [6]	<10 [6]	<10 [6]	<10 [6]	<10 [6]	<10 [6]
	Prison 2	77	72 (93.5)	<10 [6]	30 (39.0)	<10 [6]	<10 [6]	23 (29.9)	<10 [6]	<10 [6]	<10 [6]
	Prison 3	88	62 (70.5)	<10 [6]	27 (30.7)	<10 [6]	<10 [6]	22 (25.0)	<10 [6]	<10 [6]	<10 [6]
	Prison 4	<10 [6]	<10 [6]	<10 [6]	<10 [6]	<10 [6]	<10 [6]	<10 [6]	<10 [6]	<10 [6]	<10 [6]
	Prison 5	25	23 (92.0)	<10 [6]	<10 [6]	<10 [6]	<10 [6]	<10 [6]	<10 [6]	<10 [6]	<10 [6]
	Prison 6	51	46 (90.2)	<10 [6]	30 (58.8)	<10 [6]	<10 [6]	20 (39.2)	<10 [6]	<10 [6]	<10 [6]
	Prison 7	29	24 (82.8)	<10 [6]	10 (34.5)	<10 [6]	<10 [6]	<10 [6]	<10 [6]	<10 [6]	<10 [6]
	Prison 8	44	40 (90.9)	<10 [6]	12 (27.3)	<10 [6]	<10 [6]	<10 [6]	<10 [6]	<10 [6]	<10 [6]
	Prison 9	128	118 (92.2)	<10 [6]	85 (66.4)	<10 [6]	<10 [6]	37 (28.9)	<10 [6]	<10 [6]	<10 [6]
	Prison 10	97	80 (82.5)	<10 [6]	24 (24.7)	<10 [6]	<10 [6]	<10 [6]	<10 [6]	<10 [6]	<10 [6]
	Prison 11	138	92 (66.7)	<10 [6]	<10 [6]	<10 [6]	<10 [6]	10 (7.2)	<10 [6]	<10 [6]	<10 [6]
	Prison 12	<10 [6]	<10 [6]	<10 [6]	<10 [6]	<10 [6]	<10 [6]	<10 [6]	<10 [6]	<10 [6]	<10 [6]
	Prison 13	46	34 (73.9)	<10 [6]	30 (65.2)	<10 [6]	<10 [6]	30 (65.2)	<10 [6]	<10 [6]	<10 [6]
**Prison category**											
2017/18	A	128	112 (87.5)	<10 [6]	74 (57.8)	<10 [6]	<10 [6]	58 (45.3)	<10 [6]	<10 [6]	<10 [6]
	B	254	190 (74.8)	<10 [6]	52 (20.5)	<10 [6]	<10 [6]	29 (11.4)	<10 [6]	<10 [6]	<10 [6]
	C	163	126 (77.3)	<10 [6]	51 (31.3)	10 (6.1)	<10 [6]	39 (23.9)	<10 [6]	<10 [6]	<10 [6]
	Closed	50	42 (84.0)	<10 [6]	19 (38.0)	<10 [6]	<10 [6]	<10 [6]	<10 [6]	<10 [6]	<10 [6]
	D	76	61 (80.3)	<10 [6]	37 (48.7)	<10 [6]	<10 [6]	26 (34.2)	<10 [6]	<10 [6]	<10 [6]
	YOI	<10 [6]	<10 [6]	<10 [6]	<10 [6]	<10 [6]	<10 [6]	<10 [6]	<10 [6]	<10 [6]	<10 [6]
2018/19	A	144	138 (95.8)	<10 [6]	92 (63.9)	<10 [6]	<10 [6]	72 (50.0)	<10 [6]	<10 [6]	<10 [6]
	B	263	224 (85.2)	<10 [6]	43 (16.3)	10 (3.8)	<10 [6]	17 (6.5)	11 (4.2)	<10 [6]	<10 [6]
	C	180	130 (72.2)	<10 [6]	62 (34.4)	<10 [6]	<10 [6]	61 (33.9)	<10 [6]	<10 [6]	<10 [6]
	Closed	53	42 (79.2)	<10 [6]	15 (28.3)	<10 [6]	<10 [6]	<10 [6]	<10 [6]	<10 [6]	<10 [6]
	D	70	58 (82.9)	<10 [6]	35 (50.0)	<10 [6]	<10 [6]	24 (34.3)	<10 [6]	<10 [6]	<10 [6]
	YOI	<10 [6]	<10 [6]	<10 [6]	<10 [6]	<10 [6]	<10 [6]	<10 [6]	<10 [6]	<10 [6]	<10 [6]
2019/20	A	174	152 (87.4)	<10 [6]	115 (66.1)	<10 [6]	<10 [6]	67 (38.5)	<10 [6]	<10 [6]	<10 [6]
	B	312	244 (78.2)	<10 [6]	57 (18.3)	10 (3.2)	<10 [6]	40 (12.8)	<10 [6]	<10 [6]	<10 [6]
	C	131	98 (74.8)	<10 [6]	46 (35.1)	<10 [6]	<10 [6]	33 (25.2)	<10 [6]	<10 [6]	<10 [6]
	Closed	73	64 (87.7)	<10 [6]	22 (30.1)	<10 [6]	<10 [6]	<10 [6]	<10 [6]	<10 [6]	<10 [6]
	D	76	69 (90.8)	<10 [6]	37 (48.7)	11 (14.5)	<10 [6]	28 (36.8)	<10 [6]	<10 [6]	<10 [6]
	YOI	<10 [6]	<10 [6]	<10 [6]	<10 [6]	<10 [6]	<10 [6]	<10 [6]	<10 [6]	<10 [6]	<10 [6]

[1]Eligible for indicator; [2]Achieve in current prison; [3]Achieve in previous prison; [4]Overall achievement - either current or previous prison; [5]Declined indicator; [6]Suppressed (<10) to avoid disclosure

Indicator	The proportion of people with diabetes who have had the following in the preceding 12 months: body mass index, blood pressure, record of smoking status, foot examination, urine albumin: creatinine ratio blood tests for HbA1c, cholesterol and serum creatinine											
	Group: Diabetes, Asthma & Epilepsy Care											
	68.01% of those without moderate or severe frailty have BP of 140/80 or less, 56.66% of those without moderate or severe frailty have HbA1c of 58 or less, 83.34% of those with moderate or severe frailty have HbA1c of 75 or less, 78.04% had foot examination with risk classification. No comparisons for other examinations.											
			Eligible[1]	BMI (% eligible)		BP (% eligible)			Hb1Ac (% eligible)		Chol (% eligible)	
	Variable	Population	(% popln)	Satisfy[2]	Elsewhere[3]	Satisfy[2]	Elsewhere[3]	Declined[5]	Satisfy[2]	Elsewhere[3]	Satisfy[2]	Elsewhere[3]
**Gender**												
2017/18	F	1,699	50 (2.9)	38 (76.0)	<10 [6]	43 (86.0)	<10 [6]	<10 [6]	23 (46.0)	<10 [6]	21 (42.0)	<10 [6]
	M	19,977	625 (3.1)	452 (72.3)	<10 [6]	505 (80.8)	25 (4.0)	<10 [6]	365 (58.4)	40 (6.4)	292 (46.7)	34 (5.4)
2018/19	F	1,802	52 (2.9)	40 (76.9)	<10 [6]	50 (96.2)	<10 [6]	<10 [6]	31 (59.6)	<10 [6]	16 (30.8)	<10 [6]
	M	20,295	661 (3.3)	549 (83.1)	<10 [6]	574 (86.8)	12 (1.8)	<10 [6]	398 (60.2)	29 (4.4)	347 (52.5)	26 (3.9)
2019/20	F	1,376	44 (3.2)	35 (79.5)	<10 [6]	41 (93.2)	<10 [6]	<10 [6]	21 (47.7)	<10 [6]	12 (27.3)	<10 [6]
	M	23,570	697 (3.0)	598 (85.8)	<10 [6]	651 (93.4)	<10 [6]	<10 [6]	430 (61.7)	30 (4.3)	377 (54.1)	20 (2.9)
**Sentence Status**												
2017/18	.	81	<10 [6]	<10 [6]	<10 [6]	<10 [6]	<10 [6]	<10 [6]	<10 [6]	<10 [6]	<10 [6]	<10 [6]
	Absconded	<10 [6]	<10 [6]	<10 [6]	<10 [6]	<10 [6]	<10 [6]	<10 [6]	<10 [6]	<10 [6]	<10 [6]	<10 [6]
	Active In	15,872	495 (3.1)	371 (74.9)	<10 [6]	402 (81.2)	24 (4.8)	<10 [6]	292 (59.0)	42 (8.5)	238 (48.1)	35 (7.1)
	Active Out	1,052	33 (3.1)	16 (48.5)	<10 [6]	24 (72.7)	<10 [6]	<10 [6]	17 (51.5)	<10 [6]	15 (45.5)	<10 [6]
	Convicted Sentence	2,125	68 (3.2)	47 (69.1)	<10 [6]	56 (82.4)	<10 [6]	<10 [6]	38 (55.9)	<10 [6]	28 (41.2)	<10 [6]
	Downgrade in security category	<10 [6]	<10 [6]	<10 [6]	<10 [6]	<10 [6]	<10 [6]	<10 [6]	<10 [6]	<10 [6]	<10 [6]	<10 [6]
	Internal Cell Move	<10 [6]	<10 [6]	<10 [6]	<10 [6]	<10 [6]	<10 [6]	<10 [6]	<10 [6]	<10 [6]	<10 [6]	<10 [6]
	Judges Remand	92	<10 [6]	<10 [6]	<10 [6]	<10 [6]	<10 [6]	<10 [6]	<10 [6]	<10 [6]	<10 [6]	<10 [6]
	Licence Revoke	52	<10 [6]	<10 [6]	<10 [6]	<10 [6]	<10 [6]	<10 [6]	<10 [6]	<10 [6]	<10 [6]	<10 [6]
	On Remand	1,492	38 (2.5)	25 (65.8)	<10 [6]	29 (76.3)	<10 [6]	<10 [6]	20 (52.6)	<10 [6]	16 (42.1)	<10 [6]
	Transfer	910	31 (3.4)	25 (80.6)	<10 [6]	30 (96.8)	<10 [6]	<10 [6]	20 (64.5)	<10 [6]	15 (48.4)	<10 [6]
	Upgrade in security category	<10 [6]	<10 [6]	<10 [6]	<10 [6]	<10 [6]	<10 [6]	<10 [6]	<10 [6]	<10 [6]	<10 [6]	<10 [6]
2018/19	.	88	<10 [6]	<10 [6]	<10 [6]	<10 [6]	<10 [6]	<10 [6]	<10 [6]	<10 [6]	<10 [6]	<10 [6]
	Absconded	<10 [6]	<10 [6]	<10 [6]	<10 [6]	<10 [6]	<10 [6]	<10 [6]	<10 [6]	<10 [6]	<10 [6]	<10 [6]
	Active In	18,145	589 (3.2)	492 (83.5)	<10 [6]	516 (87.6)	<10 [6]	<10 [6]	366 (62.1)	29 (4.9)	318 (54.0)	26 (4.4)
	Active Out	835	34 (4.1)	26 (76.5)	<10 [6]	30 (88.2)	<10 [6]	<10 [6]	17 (50.0)	<10 [6]	13 (38.2)	<10 [6]
	Convicted Sentence	1,320	38 (2.9)	29 (76.3)	<10 [6]	31 (81.6)	<10 [6]	<10 [6]	20 (52.6)	<10 [6]	14 (36.8)	<10 [6]
	Downgrade in security category	<10 [6]	<10 [6]	<10 [6]	<10 [6]	<10 [6]	<10 [6]	<10 [6]	<10 [6]	<10 [6]	<10 [6]	<10 [6]
	Internal Cell Move	<10 [6]	<10 [6]	<10 [6]	<10 [6]	<10 [6]	<10 [6]	<10 [6]	<10 [6]	<10 [6]	<10 [6]	<10 [6]
	Judges Remand	<10 [6]	<10 [6]	<10 [6]	<10 [6]	<10 [6]	<10 [6]	<10 [6]	<10 [6]	<10 [6]	<10 [6]	<10 [6]
	Licence Revoke	125	<10 [6]	<10 [6]	<10 [6]	<10 [6]	<10 [6]	<10 [6]	<10 [6]	<10 [6]	<10 [6]	<10 [6]
	On Remand	1,059	27 (2.5)	23 (85.2)	<10 [6]	26 (96.3)	<10 [6]	<10 [6]	14 (51.9)	<10 [6]	<10 [6]	<10 [6]
	Transfer	518	19 (3.7)	17 (89.5)	<10 [6]	19 (100.0)	<10 [6]	<10 [6]	11 (57.9)	<10 [6]	<10 [6]	<10 [6]
	Upgrade in security category	<10 [6]	<10 [6]	<10 [6]	<10 [6]	<10 [6]	<10 [6]	<10 [6]	<10 [6]	<10 [6]	<10 [6]	<10 [6]
2019/20	.	69	<10 [6]	<10 [6]	<10 [6]	<10 [6]	<10 [6]	<10 [6]	<10 [6]	<10 [6]	<10 [6]	<10 [6]
	Absconded	<10 [6]	<10 [6]	<10 [6]	<10 [6]	<10 [6]	<10 [6]	<10 [6]	<10 [6]	<10 [6]	<10 [6]	<10 [6]
	Active In	22,424	674 (3.0)	578 (85.8)	<10 [6]	629 (93.3)	<10 [6]	<10 [6]	418 (62.0)	30 (4.5)	364 (54.0)	21 (3.1)
	Active Out	625	26 (4.2)	18 (69.2)	<10 [6]	25 (96.2)	<10 [6]	<10 [6]	<10 [6]	<10 [6]	<10 [6]	<10 [6]
	Convicted Sentence	1,361	36 (2.6)	35 (97.2)	<10 [6]	35 (97.2)	<10 [6]	<10 [6]	25 (69.4)	<10 [6]	21 (58.3)	<10 [6]
	Downgrade in security category	<10 [6]	<10 [6]	<10 [6]	<10 [6]	<10 [6]	<10 [6]	<10 [6]	<10 [6]	<10 [6]	<10 [6]	<10 [6]
	Internal Cell Move	<10 [6]	<10 [6]	<10 [6]	<10 [6]	<10 [6]	<10 [6]	<10 [6]	<10 [6]	<10 [6]	<10 [6]	<10 [6]
	Judges Remand	19	<10 [6]	<10 [6]	<10 [6]	<10 [6]	<10 [6]	<10 [6]	<10 [6]	<10 [6]	<10 [6]	<10 [6]
	Licence Revoke	178	<10 [6]	<10 [6]	<10 [6]	<10 [6]	<10 [6]	<10 [6]	<10 [6]	<10 [6]	<10 [6]	<10 [6]
	On Remand	1,031	23 (2.2)	20 (87.0)	<10 [6]	21 (91.3)	<10 [6]	<10 [6]	15 (65.2)	<10 [6]	14 (60.9)	<10 [6]
	Transfer	101	<10 [6]	<10 [6]	<10 [6]	<10 [6]	<10 [6]	<10 [6]	<10 [6]	<10 [6]	<10 [6]	<10 [6]
	Upgrade in security category	<10 [6]	<10 [6]	<10 [6]	<10 [6]	<10 [6]	<10 [6]	<10 [6]	<10 [6]	<10 [6]	<10 [6]	<10 [6]
**Age - years**												
2017/18	10 - <20	468	<10 [6]	<10 [6]	<10 [6]	<10 [6]	<10 [6]	<10 [6]	<10 [6]	<10 [6]	<10 [6]	<10 [6]
	20 - <30	6,994	38 (0.5)	26 (68.4)	<10 [6]	33 (86.8)	<10 [6]	<10 [6]	18 (47.4)	<10 [6]	<10 [6]	<10 [6]
	30 - <40	7,051	77 (1.1)	54 (70.1)	<10 [6]	60 (77.9)	<10 [6]	<10 [6]	32 (41.6)	<10 [6]	29 (37.7)	<10 [6]
	40 - <50	4,114	150 (3.6)	107 (71.3)	<10 [6]	115 (76.7)	<10 [6]	<10 [6]	78 (52.0)	<10 [6]	67 (44.7)	<10 [6]
	50 - <60	2,107	218 (10.3)	165 (75.7)	<10 [6]	186 (85.3)	<10 [6]	<10 [6]	134 (61.5)	18 (8.3)	110 (50.5)	16 (7.3)
	60 - <70	684	124 (18.1)	91 (73.4)	<10 [6]	99 (79.8)	<10 [6]	<10 [6]	78 (62.9)	<10 [6]	61 (49.2)	<10 [6]
	70 - <80	213	52 (24.4)	35 (67.3)	<10 [6]	43 (82.7)	<10 [6]	<10 [6]	36 (69.2)	<10 [6]	29 (55.8)	<10 [6]
	80 - <90	40	13 (32.5)	10 (76.9)	<10 [6]	10 (76.9)	<10 [6]	<10 [6]	10 (76.9)	<10 [6]	<10 [6]	<10 [6]
	90 - <100	<10 [6]	<10 [6]	<10 [6]	<10 [6]	<10 [6]	<10 [6]	<10 [6]	<10 [6]	<10 [6]	<10 [6]	<10 [6]
	100 - <110	<10 [6]	<10 [6]	<10 [6]	<10 [6]	<10 [6]	<10 [6]	<10 [6]	<10 [6]	<10 [6]	<10 [6]	<10 [6]

[1]Eligible for indicator; [2]Achieve in current prison; [3]Achieve in previous prison; [4]Overall achievement - either current or previous prison; [5]Declined indicator; [6]Suppressed (<10) to avoid disclosure

Indicator	*The proportion of people with diabetes who have had the following in the preceding 12 months: body mass index, blood pressure, record of smoking status, foot examination, urine albumin: creatinine ratio blood tests for HbA1c, cholesterol and serum creatinine*										*68.01% of those without moderate or severe frailty have BP of 140/80 or less, 56.66% of those without moderate or severe frailty have HbA1c of 58 or less, 83.34% of those with moderate or severe frailty have HbA1c of 75 or less, 78.04% had foot examination with risk classification. No comparisons for other examinations.*
	**Group: Diabetes, Asthma & Epilepsy Care**										
			**Smoke Status (% eligible)**		**Foot Exam (% eligible)**			**lbumin Creatinine Ratio (% eligible)**		**Serum Creatinine (% eligible)**	
	**Variable**	**Eligible[1]**	**Satisfy[2]**	**Elsewhere[3]**	**Satisfy[2]**	**Elsewhere[3]**	**Declined[5]**	**Satisfy[2]**	**Elsewhere[3]**	**Satisfy[2]**	**Elsewhere[3]**
Gender											
2017/18	F	50	42 (84.0)	<10 [6]	19 (38.0)	<10 [6]	<10 [6]	<10 [6]	<10 [6]	<10 [6]	<10 [6]
	M	625	493 (78.9)	<10 [6]	216 (34.6)	20 (3.2)	<10 [6]	153 (24.5)	18 (2.9)	12 (1.9)	<10 [6]
2018/19	F	52	42 (80.8)	<10 [6]	15 (28.8)	<10 [6]	<10 [6]	<10 [6]	<10 [6]	<10 [6]	<10 [6]
	M	661	553 (83.7)	16 (2.4)	235 (35.6)	24 (3.6)	<10 [6]	175 (26.5)	23 (3.5)	<10 [6]	<10 [6]
2019/20	F	44	40 (90.9)	<10 [6]	12 (27.3)	<10 [6]	<10 [6]	<10 [6]	<10 [6]	<10 [6]	<10 [6]
	M	697	565 (81.1)	13 (1.9)	256 (36.7)	33 (4.7)	<10 [6]	170 (24.4)	21 (3.0)	<10 [6]	<10 [6]
**Sentence Status**											
2017/18	.	<10 [6]	<10 [6]	<10 [6]	<10 [6]	<10 [6]	<10 [6]	<10 [6]	<10 [6]	<10 [6]	<10 [6]
	Absconded	<10 [6]	<10 [6]	<10 [6]	<10 [6]	<10 [6]	<10 [6]	<10 [6]	<10 [6]	<10 [6]	<10 [6]
	Active In	495	402 (81.2)	<10 [6]	175 (35.4)	20 (4.0)	<10 [6]	119 (24.0)	17 (3.4)	<10 [6]	<10 [6]
	Active Out	33	23 (69.7)	<10 [6]	10 (30.3)	<10 [6]	<10 [6]	<10 [6]	<10 [6]	<10 [6]	<10 [6]
	Convicted Sentence	68	52 (76.5)	<10 [6]	21 (30.9)	<10 [6]	<10 [6]	11 (16.2)	<10 [6]	<10 [6]	<10 [6]
	Downgrade in security category	<10 [6]	<10 [6]	<10 [6]	<10 [6]	<10 [6]	<10 [6]	<10 [6]	<10 [6]	<10 [6]	<10 [6]
	Internal Cell Move	<10 [6]	<10 [6]	<10 [6]	<10 [6]	<10 [6]	<10 [6]	<10 [6]	<10 [6]	<10 [6]	<10 [6]
	Judges Remand	<10 [6]	<10 [6]	<10 [6]	<10 [6]	<10 [6]	<10 [6]	<10 [6]	<10 [6]	<10 [6]	<10 [6]
	Licence Revoke	<10 [6]	<10 [6]	<10 [6]	<10 [6]	<10 [6]	<10 [6]	<10 [6]	<10 [6]	<10 [6]	<10 [6]
	On Remand	38	25 (65.8)	<10 [6]	14 (36.8)	<10 [6]	<10 [6]	<10 [6]	<10 [6]	<10 [6]	<10 [6]
	Transfer	31	27 (87.1)	<10 [6]	15 (48.4)	<10 [6]	<10 [6]	11 (35.5)	<10 [6]	<10 [6]	<10 [6]
	Upgrade in security category	<10 [6]	<10 [6]	<10 [6]	<10 [6]	<10 [6]	<10 [6]	<10 [6]	<10 [6]	<10 [6]	<10 [6]
2018/19	.	<10 [6]	<10 [6]	<10 [6]	<10 [6]	<10 [6]	<10 [6]	<10 [6]	<10 [6]	<10 [6]	<10 [6]
	Absconded	<10 [6]	<10 [6]	<10 [6]	<10 [6]	<10 [6]	<10 [6]	<10 [6]	<10 [6]	<10 [6]	<10 [6]
	Active In	589	493 (83.7)	16 (2.7)	202 (34.3)	25 (4.2)	<10 [6]	161 (27.3)	22 (3.7)	<10 [6]	<10 [6]
	Active Out	34	31 (91.2)	<10 [6]	10 (29.4)	<10 [6]	<10 [6]	<10 [6]	<10 [6]	<10 [6]	<10 [6]
	Convicted Sentence	38	29 (76.3)	<10 [6]	13 (34.2)	<10 [6]	<10 [6]	<10 [6]	<10 [6]	<10 [6]	<10 [6]
	Downgrade in security category	<10 [6]	<10 [6]	<10 [6]	<10 [6]	<10 [6]	<10 [6]	<10 [6]	<10 [6]	<10 [6]	<10 [6]
	Internal Cell Move	<10 [6]	<10 [6]	<10 [6]	<10 [6]	<10 [6]	<10 [6]	<10 [6]	<10 [6]	<10 [6]	<10 [6]
	Judges Remand	<10 [6]	<10 [6]	<10 [6]	<10 [6]	<10 [6]	<10 [6]	<10 [6]	<10 [6]	<10 [6]	<10 [6]
	Licence Revoke	<10 [6]	<10 [6]	<10 [6]	<10 [6]	<10 [6]	<10 [6]	<10 [6]	<10 [6]	<10 [6]	<10 [6]
	On Remand	27	26 (96.3)	<10 [6]	13 (48.1)	<10 [6]	<10 [6]	<10 [6]	<10 [6]	<10 [6]	<10 [6]
	Transfer	19	13 (68.4)	<10 [6]	11 (57.9)	<10 [6]	<10 [6]	<10 [6]	<10 [6]	<10 [6]	<10 [6]
	Upgrade in security category	<10 [6]	<10 [6]	<10 [6]	<10 [6]	<10 [6]	<10 [6]	<10 [6]	<10 [6]	<10 [6]	<10 [6]
2019/20	.	<10 [6]	<10 [6]	<10 [6]	<10 [6]	<10 [6]	<10 [6]	<10 [6]	<10 [6]	<10 [6]	<10 [6]
	Absconded	<10 [6]	<10 [6]	<10 [6]	<10 [6]	<10 [6]	<10 [6]	<10 [6]	<10 [6]	<10 [6]	<10 [6]
	Active In	674	545 (80.9)	12 (1.8)	245 (36.4)	33 (4.9)	<10 [6]	153 (22.7)	22 (3.3)	<10 [6]	<10 [6]
	Active Out	26	20 (76.9)	<10 [6]	<10 [6]	<10 [6]	<10 [6]	<10 [6]	<10 [6]	<10 [6]	<10 [6]
	Convicted Sentence	36	34 (94.4)	<10 [6]	15 (41.7)	<10 [6]	<10 [6]	11 (30.6)	<10 [6]	<10 [6]	<10 [6]
	Downgrade in security category	<10 [6]	<10 [6]	<10 [6]	<10 [6]	<10 [6]	<10 [6]	<10 [6]	<10 [6]	<10 [6]	<10 [6]
	Internal Cell Move	<10 [6]	<10 [6]	<10 [6]	<10 [6]	<10 [6]	<10 [6]	<10 [6]	<10 [6]	<10 [6]	<10 [6]
	Judges Remand	<10 [6]	<10 [6]	<10 [6]	<10 [6]	<10 [6]	<10 [6]	<10 [6]	<10 [6]	<10 [6]	<10 [6]
	Licence Revoke	<10 [6]	<10 [6]	<10 [6]	<10 [6]	<10 [6]	<10 [6]	<10 [6]	<10 [6]	<10 [6]	<10 [6]
	On Remand	23	21 (91.3)	<10 [6]	11 (47.8)	<10 [6]	<10 [6]	<10 [6]	<10 [6]	<10 [6]	<10 [6]
	Transfer	<10 [6]	<10 [6]	<10 [6]	<10 [6]	<10 [6]	<10 [6]	<10 [6]	<10 [6]	<10 [6]	<10 [6]
	Upgrade in security category	<10 [6]	<10 [6]	<10 [6]	<10 [6]	<10 [6]	<10 [6]	<10 [6]	<10 [6]	<10 [6]	<10 [6]
Age - years											
2017/18	10 - <20	<10 [6]	<10 [6]	<10 [6]	<10 [6]	<10 [6]	<10 [6]	<10 [6]	<10 [6]	<10 [6]	<10 [6]
	20 - <30	38	34 (89.5)	<10 [6]	11 (28.9)	<10 [6]	<10 [6]	<10 [6]	<10 [6]	<10 [6]	<10 [6]
	30 - <40	77	58 (75.3)	<10 [6]	23 (29.9)	<10 [6]	<10 [6]	14 (18.2)	<10 [6]	<10 [6]	<10 [6]
	40 - <50	150	117 (78.0)	<10 [6]	43 (28.7)	<10 [6]	<10 [6]	24 (16.0)	<10 [6]	<10 [6]	<10 [6]
	50 - <60	218	175 (80.3)	<10 [6]	78 (35.8)	<10 [6]	<10 [6]	52 (23.9)	<10 [6]	<10 [6]	<10 [6]
	60 - <70	124	98 (79.0)	<10 [6]	54 (43.5)	<10 [6]	<10 [6]	37 (29.8)	<10 [6]	<10 [6]	<10 [6]
	70 - <80	52	41 (78.8)	<10 [6]	22 (42.3)	<10 [6]	<10 [6]	18 (34.6)	<10 [6]	<10 [6]	<10 [6]
	80 - <90	13	10 (76.9)	<10 [6]	<10 [6]	<10 [6]	<10 [6]	<10 [6]	<10 [6]	<10 [6]	<10 [6]
	90 - <100	<10 [6]	<10 [6]	<10 [6]	<10 [6]	<10 [6]	<10 [6]	<10 [6]	<10 [6]	<10 [6]	<10 [6]
	100 - <110	<10 [6]	<10 [6]	<10 [6]	<10 [6]	<10 [6]	<10 [6]	<10 [6]	<10 [6]	<10 [6]	<10 [6]

[1]Eligible for indicator; [2]Achieve in current prison; [3]Achieve in previous prison; [4]Overall achievement - either current or previous prison; [5]Declined indicator; [6]Suppressed (<10) to avoid disclosure

Indicator	The proportion of people with diabetes who have had the following in the preceding 12 months: body mass index, blood pressure, record of smoking status, foot examination, urine albumin: creatinine ratio blood tests for HbA1c, cholesterol and serum creatinine									68.01% of those without moderate or severe frailty have BP of 140/80 or less, 56.66% of those without moderate or severe frailty have HbA1c of 58 or less, 83.34% of those with moderate or severe frailty have HbA1c of 75 or less, 78.04% had foot examination with risk classification. No comparisons for other examinations.		
	Group: Diabetes, Asthma & Epilepsy Care											
			Eligible[1]	BMI (% eligible)		BP (% eligible)			Hb1Ac (% eligible)		Chol (% eligible)	
	Variable	Population	(% popln)	Satisfy[2]	Elsewhere[3]	Satisfy[2]	Elsewhere[3]	Declined[5]	Satisfy[2]	Elsewhere[3]	Satisfy[2]	Elsewhere[3]
2018/19	10 - <20	436	<10 [6]	<10 [6]	<10 [6]	<10 [6]	<10 [6]	<10 [6]	<10 [6]	<10 [6]	<10 [6]	<10 [6]
	20 - <30	7,163	50 (0.7)	40 (80.0)	<10 [6]	39 (78.0)	<10 [6]	<10 [6]	23 (46.0)	<10 [6]	12 (24.0)	<10 [6]
	30 - <40	7,381	106 (1.4)	84 (79.2)	<10 [6]	94 (88.7)	<10 [6]	<10 [6]	49 (46.2)	<10 [6]	38 (35.8)	<10 [6]
	40 - <50	4,180	148 (3.5)	128 (86.5)	<10 [6]	128 (86.5)	<10 [6]	<10 [6]	70 (47.3)	<10 [6]	59 (39.9)	<10 [6]
	50 - <60	1,978	213 (10.8)	168 (78.9)	<10 [6]	184 (86.4)	<10 [6]	<10 [6]	146 (68.5)	<10 [6]	123 (57.7)	<10 [6]
	60 - <70	701	133 (19.0)	114 (85.7)	<10 [6]	121 (91.0)	<10 [6]	<10 [6]	95 (71.4)	<10 [6]	91 (68.4)	<10 [6]
	70 - <80	209	49 (23.4)	42 (85.7)	<10 [6]	44 (89.8)	<10 [6]	<10 [6]	35 (71.4)	<10 [6]	33 (67.3)	<10 [6]
	80 - <90	45	13 (28.9)	12 (92.3)	<10 [6]	13 (100.0)	<10 [6]	<10 [6]	10 (76.9)	<10 [6]	<10 [6]	<10 [6]
	90 - <100	<10 [6]	<10 [6]	<10 [6]	<10 [6]	<10 [6]	<10 [6]	<10 [6]	<10 [6]	<10 [6]	<10 [6]	<10 [6]
	100 - <110	<10 [6]	<10 [6]	<10 [6]	<10 [6]	<10 [6]	<10 [6]	<10 [6]	<10 [6]	<10 [6]	<10 [6]	<10 [6]
2019/20	10 - <20	404	<10 [6]	<10 [6]	<10 [6]	<10 [6]	<10 [6]	<10 [6]	<10 [6]	<10 [6]	<10 [6]	<10 [6]
	20 - <30	8,064	60 (0.7)	50 (83.3)	<10 [6]	54 (90.0)	<10 [6]	<10 [6]	29 (48.3)	<10 [6]	18 (30.0)	<10 [6]
	30 - <40	9,125	100 (1.1)	82 (82.0)	<10 [6]	92 (92.0)	<10 [6]	<10 [6]	50 (50.0)	<10 [6]	43 (43.0)	<10 [6]
	40 - <50	4,948	195 (3.9)	168 (86.2)	<10 [6]	179 (91.8)	<10 [6]	<10 [6]	107 (54.9)	15 (7.7)	92 (47.2)	10 (5.1)
	50 - <60	2,224	193 (8.7)	170 (88.1)	<10 [6]	180 (93.3)	<10 [6]	<10 [6]	128 (66.3)	<10 [6]	113 (58.5)	<10 [6]
	60 - <70	751	152 (20.2)	129 (84.9)	<10 [6]	146 (96.1)	<10 [6]	<10 [6]	110 (72.4)	<10 [6]	101 (66.4)	<10 [6]
	70 - <80	238	57 (23.9)	46 (80.7)	<10 [6]	54 (94.7)	<10 [6]	<10 [6]	37 (64.9)	<10 [6]	32 (56.1)	<10 [6]
	80 - <90	53	11 (20.8)	11 (100.0)	<10 [6]	11 (100.0)	<10 [6]	<10 [6]	<10 [6]	<10 [6]	<10 [6]	<10 [6]
	90 - <100	<10 [6]	<10 [6]	<10 [6]	<10 [6]	<10 [6]	<10 [6]	<10 [6]	<10 [6]	<10 [6]	<10 [6]	<10 [6]
	100 - <110	<10 [6]	<10 [6]	<10 [6]	<10 [6]	<10 [6]	<10 [6]	<10 [6]	<10 [6]	<10 [6]	<10 [6]	<10 [6]
**Length of Stay (months)**												
2017/18	<1	4,474	94 (2.1)	68 (72.3)	<10 [6]	66 (70.2)	12 (12.8)	<10 [6]	11 (11.7)	14 (14.9)	<10 [6]	<10 [6]
	1-<6	8,075	179 (2.2)	129 (72.1)	<10 [6]	141 (78.8)	<10 [6]	<10 [6]	68 (38.0)	15 (8.4)	48 (26.8)	10 (5.6)
	6-<12	3,672	106 (2.9)	75 (70.8)	<10 [6]	85 (80.2)	<10 [6]	<10 [6]	72 (67.9)	<10 [6]	56 (52.8)	<10 [6]
	12-<24	2,832	94 (3.3)	65 (69.1)	<10 [6]	77 (81.9)	<10 [6]	<10 [6]	64 (68.1)	<10 [6]	53 (56.4)	<10 [6]
	24+	2,624	202 (7.7)	153 (75.7)	<10 [6]	179 (88.6)	<10 [6]	<10 [6]	173 (85.6)	<10 [6]	147 (72.8)	<10 [6]
2018/19	<1	4,801	97 (2.0)	87 (89.7)	<10 [6]	90 (92.8)	<10 [6]	<10 [6]	<10 [6]	<10 [6]	<10 [6]	<10 [6]
	1-<6	7,742	185 (2.4)	149 (80.5)	<10 [6]	153 (82.7)	<10 [6]	<10 [6]	78 (42.2)	<10 [6]	56 (30.3)	<10 [6]
	6-<12	3,616	104 (2.9)	75 (72.1)	<10 [6]	85 (81.7)	<10 [6]	<10 [6]	74 (71.2)	<10 [6]	51 (49.0)	<10 [6]
	12-<24	3,752	138 (3.7)	112 (81.2)	<10 [6]	117 (84.8)	<10 [6]	<10 [6]	94 (68.1)	12 (8.7)	85 (61.6)	11 (8.0)
	24+	2,188	189 (8.6)	166 (87.8)	<10 [6]	179 (94.7)	<10 [6]	<10 [6]	174 (92.1)	<10 [6]	164 (86.8)	<10 [6]
2019/20	<1	5,745	136 (2.4)	125 (91.9)	<10 [6]	132 (97.1)	<10 [6]	<10 [6]	21 (15.4)	10 (7.4)	20 (14.7)	<10 [6]
	1-<6	9,697	216 (2.2)	181 (83.8)	<10 [6]	194 (89.8)	<10 [6]	<10 [6]	110 (50.9)	12 (5.6)	81 (37.5)	<10 [6]
	6-<12	5,090	144 (2.8)	124 (86.1)	<10 [6]	129 (89.6)	<10 [6]	<10 [6]	104 (72.2)	<10 [6]	86 (59.7)	<10 [6]
	12-<24	3,244	104 (3.2)	88 (84.6)	<10 [6]	99 (95.2)	<10 [6]	<10 [6]	86 (82.7)	<10 [6]	77 (74.0)	<10 [6]
	24+	2,035	170 (8.4)	140 (82.4)	<10 [6]	164 (96.5)	<10 [6]	<10 [6]	149 (87.6)	<10 [6]	144 (84.7)	<10 [6]

[1]Eligible for indicator; [2]Achieve in current prison; [3]Achieve in previous prison; [4]Overall achievement - either current or previous prison; [5]Declined indicator; [6]Suppressed (<10) to avoid disclosure

Indicator	*The proportion of people with diabetes who have had the following in the preceding 12 months: body mass index, blood pressure, record of smoking status, foot examination, urine albumin: creatinine ratio blood tests for HbA1c, cholesterol and serum creatinine*								*68.01% of those without moderate or severe frailty have BP of 140/80 or less, 56.66% of those without moderate or severe frailty have HbA1c of 58 or less, 83.34% of those with moderate or severe frailty have HbA1c of 75 or less, 78.04% had foot examination with risk classification. No comparisons for other examinations.*		
	**Group: Diabetes, Asthma & Epilepsy Care**										
			**Smoke Status (% eligible)**			**Foot Exam (% eligible)**		**lbumin Creatinine Ratio (% eligible)**		**Serum Creatinine (% eligible)**	
	**Variable**	**Eligible[1]**	**Satisfy[2]**	**Elsewhere[3]**	**Satisfy[2]**	**Elsewhere[3]**	**Declined[5]**	**Satisfy[2]**	**Elsewhere[3]**	**Satisfy[2]**	**Elsewhere[3]**
2018/19	10 - <20	<10 [6]	<10 [6]	<10 [6]	<10 [6]	<10 [6]	<10 [6]	<10 [6]	<10 [6]	<10 [6]	<10 [6]
	20 - <30	50	42 (84.0)	<10 [6]	<10 [6]	<10 [6]	<10 [6]	<10 [6]	<10 [6]	<10 [6]	<10 [6]
	30 - <40	106	86 (81.1)	<10 [6]	28 (26.4)	<10 [6]	<10 [6]	17 (16.0)	<10 [6]	<10 [6]	<10 [6]
	40 - <50	148	123 (83.1)	<10 [6]	42 (28.4)	<10 [6]	<10 [6]	27 (18.2)	<10 [6]	<10 [6]	<10 [6]
	50 - <60	213	178 (83.6)	<10 [6]	82 (38.5)	10 (4.7)	<10 [6]	67 (31.5)	<10 [6]	<10 [6]	<10 [6]
	60 - <70	133	116 (87.2)	<10 [6]	60 (45.1)	<10 [6]	<10 [6]	49 (36.8)	<10 [6]	<10 [6]	<10 [6]
	70 - <80	49	40 (81.6)	<10 [6]	22 (44.9)	<10 [6]	<10 [6]	16 (32.7)	<10 [6]	<10 [6]	<10 [6]
	80 - <90	13	10 (76.9)	<10 [6]	<10 [6]	<10 [6]	<10 [6]	<10 [6]	<10 [6]	<10 [6]	<10 [6]
	90 - <100	<10 [6]	<10 [6]	<10 [6]	<10 [6]	<10 [6]	<10 [6]	<10 [6]	<10 [6]	<10 [6]	<10 [6]
	100 - <110	<10 [6]	<10 [6]	<10 [6]	<10 [6]	<10 [6]	<10 [6]	<10 [6]	<10 [6]	<10 [6]	<10 [6]
2019/20	10 - <20	<10 [6]	<10 [6]	<10 [6]	<10 [6]	<10 [6]	<10 [6]	<10 [6]	<10 [6]	<10 [6]	<10 [6]
	20 - <30	60	50 (83.3)	<10 [6]	17 (28.3)	<10 [6]	<10 [6]	12 (20.0)	<10 [6]	<10 [6]	<10 [6]
	30 - <40	100	80 (80.0)	<10 [6]	28 (28.0)	<10 [6]	<10 [6]	11 (11.0)	<10 [6]	<10 [6]	<10 [6]
	40 - <50	195	164 (84.1)	<10 [6]	53 (27.2)	<10 [6]	<10 [6]	31 (15.9)	<10 [6]	<10 [6]	<10 [6]
	50 - <60	193	159 (82.4)	<10 [6]	81 (42.0)	15 (7.8)	<10 [6]	58 (30.1)	<10 [6]	<10 [6]	<10 [6]
	60 - <70	152	119 (78.3)	<10 [6]	70 (46.1)	<10 [6]	<10 [6]	40 (26.3)	<10 [6]	<10 [6]	<10 [6]
	70 - <80	57	47 (82.5)	<10 [6]	23 (40.4)	<10 [6]	<10 [6]	15 (26.3)	<10 [6]	<10 [6]	<10 [6]
	80 - <90	11	<10 [6]	<10 [6]	<10 [6]	<10 [6]	<10 [6]	<10 [6]	<10 [6]	<10 [6]	<10 [6]
	90 - <100	<10 [6]	<10 [6]	<10 [6]	<10 [6]	<10 [6]	<10 [6]	<10 [6]	<10 [6]	<10 [6]	<10 [6]
	100 - <110	<10 [6]	<10 [6]	<10 [6]	<10 [6]	<10 [6]	<10 [6]	<10 [6]	<10 [6]	<10 [6]	<10 [6]
**Length of Stay (months)**											
2017/18	<1	94	65 (69.1)	<10 [6]	<10 [6]	<10 [6]	<10 [6]	<10 [6]	<10 [6]	<10 [6]	<10 [6]
	1-<6	179	141 (78.8)	<10 [6]	32 (17.9)	<10 [6]	<10 [6]	21 (11.7)	<10 [6]	<10 [6]	<10 [6]
	6-<12	106	79 (74.5)	<10 [6]	45 (42.5)	<10 [6]	<10 [6]	15 (14.2)	<10 [6]	<10 [6]	<10 [6]
	12-<24	94	76 (80.9)	<10 [6]	46 (48.9)	<10 [6]	<10 [6]	30 (31.9)	<10 [6]	<10 [6]	<10 [6]
	24+	202	174 (86.1)	<10 [6]	109 (54.0)	<10 [6]	<10 [6]	86 (42.6)	<10 [6]	<10 [6]	<10 [6]
2018/19	<1	97	88 (90.7)	<10 [6]	<10 [6]	<10 [6]	<10 [6]	<10 [6]	<10 [6]	<10 [6]	<10 [6]
	1-<6	185	152 (82.2)	<10 [6]	29 (15.7)	<10 [6]	<10 [6]	23 (12.4)	<10 [6]	<10 [6]	<10 [6]
	6-<12	104	75 (72.1)	<10 [6]	44 (42.3)	<10 [6]	<10 [6]	17 (16.3)	<10 [6]	<10 [6]	<10 [6]
	12-<24	138	112 (81.2)	<10 [6]	52 (37.7)	<10 [6]	<10 [6]	46 (33.3)	<10 [6]	<10 [6]	<10 [6]
	24+	189	168 (88.9)	<10 [6]	121 (64.0)	<10 [6]	<10 [6]	95 (50.3)	<10 [6]	<10 [6]	<10 [6]
2019/20	<1	136	112 (82.4)	<10 [6]	<10 [6]	<10 [6]	<10 [6]	<10 [6]	<10 [6]	<10 [6]	<10 [6]
	1-<6	216	176 (81.5)	<10 [6]	41 (19.0)	12 (5.6)	<10 [6]	27 (12.5)	<10 [6]	<10 [6]	<10 [6]
	6-<12	144	120 (83.3)	<10 [6]	56 (38.9)	12 (8.3)	<10 [6]	35 (24.3)	11 (7.6)	<10 [6]	<10 [6]
	12-<24	104	80 (76.9)	<10 [6]	66 (63.5)	<10 [6]	<10 [6]	42 (40.4)	<10 [6]	<10 [6]	<10 [6]
	24+	170	141 (82.9)	<10 [6]	111 (65.3)	<10 [6]	<10 [6]	65 (38.2)	<10 [6]	<10 [6]	<10 [6]

[1]Eligible for indicator; [2]Achieve in current prison; [3]Achieve in previous prison; [4]Overall achievement - either current or previous prison; [5]Declined indicator; [6]Suppressed (<10) to avoid disclosure

Indicator	The proportion of people with diabetes who have had the following in the preceding 12 months: body mass index, blood pressure, record of smoking status, foot examination, urine albumin: creatinine ratio blood tests for HbA1c, cholesterol and serum creatinine<br>Group: Diabetes, Asthma & Epilepsy Care		Community achievement 2019/20 (%): 68.01% of those without moderate or severe frailty have BP of 140/80 or less, 56.66% of those without moderate or severe frailty have HbA1c of 58 or less, 83.34% of those with moderate or severe frailty have HbA1c of 75 or less, 78.04% had foot examination with risk classification. No comparisons for other examinations.	
	**Variable**	**Population**	**Eligible[1] (% population)**	**Achieved[4] 5 of the 8 items/tests (% eligible)**
**Year**				
	2017/18	21,677	675 (3.1)	341 (50.5)
	2018/19	22,099	713 (3.2)	381 (53.4)
	2019/20	25,811	770 (3.0)	426 (55.3)
**Prison**				
2017/18	Prison 1	1,323	25 (1.9)	14 (56.0)
	Prison 2	3,261	96 (2.9)	43 (44.8)
	Prison 3	2,623	70 (2.7)	44 (62.9)
	Prison 4	2,089	68 (3.3)	37 (54.4)
	Prison 5	637	22 (3.5)	10 (45.5)
	Prison 6	1,552	54 (3.5)	37 (68.5)
	Prison 7	635	21 (3.3)	<10 [6]
	Prison 8	1,085	29 (2.7)	11 (37.9)
	Prison 9	981	86 (8.8)	63 (73.3)
	Prison 10	2,523	75 (3.0)	15 (20.0)
	Prison 11	3,470	83 (2.4)	20 (24.1)
	Prison 12	815	<10 [6]	<10 [6]
	Prison 13	683	42 (6.1)	35 (83.3)
2018/19	Prison 1	1,333	19 (1.4)	15 (78.9)
	Prison 2	2,705	73 (2.7)	30 (41.1)
	Prison 3	2,522	85 (3.4)	48 (56.5)
	Prison 4	2,349	76 (3.2)	45 (59.2)
	Prison 5	676	22 (3.3)	<10 [6]
	Prison 6	1,513	48 (3.2)	33 (68.8)
	Prison 7	654	19 (2.9)	12 (63.2)
	Prison 8	1,148	34 (3.0)	9 (26.5)
	Prison 9	996	105 (10.5)	92 (87.6)
	Prison 10	2,717	77 (2.8)	21 (27.3)
	Prison 11	4,020	113 (2.8)	26 (23.0)
	Prison 12	792	<10 [6]	<10 [6]
	Prison 13	674	39 (5.8)	38 (97.4)
2019/20	Prison 1	1,410	37 (2.6)	25 (67.6)
	Prison 2	2,979	77 (2.6)	45 (58.4)
	Prison 3	2,809	88 (3.1)	49 (55.7)
	Prison 4	2,651	<10 [6]	<10 [6]
	Prison 5	616	25 (4.1)	19 (76.0)
	Prison 6	1,533	51 (3.3)	37 (72.5)
	Prison 7	860	29 (3.4)	15 (51.7)
	Prison 8	1,385	44 (3.2)	11 (25.0)
	Prison 9	1,092	128 (11.7)	107 (83.6)
	Prison 10	3,577	97 (2.7)	41 (42.3)
	Prison 11	5,348	138 (2.6)	32 (23.2)
	Prison 12	805	<10 [6]	<10 [6]
	Prison 13	746	46 (6.2)	38 (82.6)
**Prison category**				
2017/18	A	1,664	128 (7.7)	98 (76.6)
	B	9,254	254 (2.7)	78 (30.7)
	C	6,035	163 (2.7)	95 (58.3)
	Closed	1,720	50 (2.9)	20 (40.0)
	D	2,189	76 (3.5)	47 (61.8)
	YOI	815	<10 [6]	<10 [6]
2018/19	A	1,670	144 (8.6)	130 (90.3)
	B	9,442	263 (2.8)	77 (29.3)
	C	6,204	180 (2.9)	108 (60.0)
	Closed	1,802	53 (2.9)	21 (39.6)
	D	2,189	70 (3.2)	42 (60.0)
	YOI	792	<10 [6]	<10 [6]
2019/20	A	1,838	174 (9.5)	145 (83.3)
	B	11,904	312 (2.6)	118 (37.8)
	C	6,870	131 (1.9)	80 (61.1)
	Closed	2,245	73 (3.3)	26 (35.6)
	D	2,149	76 (3.5)	56 (73.7)
	YOI	805	<10 [6]	<10 [6]
**Gender**				
2017/18	F	1,699	50 (2.9)	20 (40.0)
	M	19,977	625 (3.1)	321 (51.4)
2018/19	F	1,802	52 (2.9)	21 (40.4)
	M	20,295	661 (3.3)	360 (54.5)
2019/20	F	1,376	44 (3.2)	11 (25.0)
	M	23,570	697 (3.0)	400 (57.4)

[1]Eligible for indicator; [2]Achieve in current prison; [3]Achieve in previous prison; [4]Overall achievement - either current or previous prison; [5]Declined indicator; [6]Suppressed (<10) to avoid disclosure

Indicator	*The proportion of people with diabetes who have had the following in the preceding 12 months: body mass index, blood pressure, record of smoking status, foot examination, urine albumin: creatinine ratio blood tests for HbA1c, cholesterol and serum creatinine*	*Community achievement 2019/20 (%): 68.01% of those without moderate or severe frailty have BP of 140/80 or less, 56.66% of those without moderate or severe frailty have HbA1c of 58 or less, 83.34% of those with moderate or severe frailty have HbA1c of 75 or less, 78.04% had foot examination with risk classification. No comparisons for other examinations.*
	**Group: Diabetes, Asthma & Epilepsy Care**	

	**Variable**	**Population**	**Eligible[1] (% population)**	**Achieved[4] 5 of the 8 items/tests (% eligible)**
**Sentence Status**				
2017/18	.	81	<10 [6]	<10 [6]
	Absconded	<10 [6]	<10 [6]	<10 [6]
	Active In	15,872	495 (3.1)	267 (53.9)
	Active Out	1,052	33 (3.1)	13 (39.4)
	Convicted Sentence	2,125	68 (3.2)	29 (42.6)
	Downgrade in security category	<10 [6]	<10 [6]	<10 [6]
	Internal Cell Move	<10 [6]	<10 [6]	<10 [6]
	Judges Remand	92	<10 [6]	<10 [6]
	Licence Revoke	52	<10 [6]	<10 [6]
	On Remand	1,492	38 (2.5)	16 (42.1)
	Transfer	910	31 (3.4)	16 (51.6)
	Upgrade in security category	<10 [6]	<10 [6]	<10 [6]
2018/19	.	88	<10 [6]	<10 [6]
	Absconded	<10 [6]	<10 [6]	<10 [6]
	Active In	18,145	589 (3.2)	330 (56.0)
	Active Out	835	34 (4.1)	12 (35.3)
	Convicted Sentence	1,320	38 (2.9)	14 (36.8)
	Downgrade in security category	<10 [6]	<10 [6]	<10 [6]
	Internal Cell Move	<10 [6]	<10 [6]	<10 [6]
	Judges Remand	<10 [6]	<10 [6]	<10 [6]
	Licence Revoke	125	<10 [6]	<10 [6]
	On Remand	1,059	27 (2.5)	14 (51.9)
	Transfer	518	19 (3.7)	10 (52.6)
	Upgrade in security category	<10 [6]	<10 [6]	<10 [6]
2019/20	.	69	<10 [6]	<10 [6]
	Absconded	<10 [6]	<10 [6]	<10 [6]
	Active In	22,424	674 (3.0)	381 (56.5)
	Active Out	625	26 (4.2)	<10 [6]
	Convicted Sentence	1,361	36 (2.6)	22 (61.1)
	Downgrade in security category	<10 [6]	<10 [6]	<10 [6]
	Internal Cell Move	<10 [6]	<10 [6]	<10 [6]
	Judges Remand	19	<10 [6]	<10 [6]
	Licence Revoke	178	<10 [6]	<10 [6]
	On Remand	1,031	23 (2.2)	14 (60.9)
	Transfer	101	<10 [6]	<10 [6]
	Upgrade in security category	<10 [6]	<10 [6]	<10 [6]
**Age - years**				
2017/18	10 - <20	468	<10 [6]	<10 [6]
	20 - <30	6,994	38 (0.5)	13 (34.2)
	30 - <40	7,051	77 (1.1)	32 (41.6)
	40 - <50	4,114	150 (3.6)	68 (45.3)
	50 - <60	2,107	218 (10.3)	121 (55.5)
	60 - <70	684	124 (18.1)	65 (52.4)
	70 - <80	213	52 (24.4)	31 (59.6)
	80 - <90	40	13 (32.5)	<10 [6]
	90 - <100	<10 [6]	<10 [6]	<10 [6]
	100 - <110	<10 [6]	<10 [6]	<10 [6]
2018/19	10 - <20	436	<10 [6]	<10 [6]
	20 - <30	7,163	50 (0.7)	18 (36.0)
	30 - <40	7,381	106 (1.4)	43 (40.6)
	40 - <50	4,180	148 (3.5)	59 (39.9)
	50 - <60	1,978	213 (10.8)	125 (58.7)
	60 - <70	701	133 (19.0)	97 (72.9)
	70 - <80	209	49 (23.4)	30 (61.2)
	80 - <90	45	13 (28.9)	<10 [6]
	90 - <100	<10 [6]	<10 [6]	<10 [6]
	100 - <110	<10 [6]	<10 [6]	<10 [6]
2019/20	10 - <20	404	<10 [6]	<10 [6]
	20 - <30	8,064	60 (0.7)	20 (33.3)
	30 - <40	9,125	100 (1.1)	41 (41.0)
	40 - <50	4,948	195 (3.9)	101 (51.8)
	50 - <60	2,224	193 (8.7)	124 (64.2)
	60 - <70	751	152 (20.2)	95 (62.5)
	70 - <80	238	57 (23.9)	36 (63.2)
	80 - <90	53	11 (20.8)	<10 [6]
	90 - <100	<10 [6]	<10 [6]	<10 [6]
	100 - <110	<10 [6]	<10 [6]	<10 [6]

[1]Eligible for indicator; [2]Achieve in current prison; [3]Achieve in previous prison; [4]Overall achievement - either current or previous prison; [5]Declined indicator; [6]Suppressed (<10) to avoid disclosure

Indicator	The proportion of people with diabetes who have had the following in the preceding 12 months: body mass index, blood pressure, record of smoking status, foot examination, urine albumin: creatinine ratio blood tests for HbA1c, cholesterol and serum creatinine		Community achievement 2019/20 (%): 68.01% of those without moderate or severe frailty have BP of 140/80 or less, 56.66% of those without moderate or severe frailty have HbA1c of 58 or less, 83.34% of those with moderate or severe frailty have HbA1c of 75 or less, 78.04% had foot examination with risk classification. No comparisons for other examinations.
	Group: Diabetes, Asthma & Epilepsy Care			
	**Variable**	**Population**	**Eligible[1] (% population)**	**Achieved[4] 5 of the 8 items/tests (% eligible)**
**Length of Stay (months)**				
2017/18	<1	4,474	94 (2.1)	17 (18.1)
	1-<6	8,075	179 (2.2)	57 (31.8)
	6-<12	3,672	106 (2.9)	58 (54.7)
	12-<24	2,832	94 (3.3)	57 (60.6)
	24+	2,624	202 (7.7)	152 (75.2)
2018/19	<1	4,801	97 (2.0)	12 (12.4)
	1-<6	7,742	185 (2.4)	58 (31.4)
	6-<12	3,616	104 (2.9)	59 (56.7)
	12-<24	3,752	138 (3.7)	94 (68.1)
	24+	2,188	189 (8.6)	158 (83.6)
2019/20	<1	5,745	136 (2.4)	22 (16.2)
	1-<6	9,697	216 (2.2)	91 (42.1)
	6-<12	5,090	144 (2.8)	100 (69.4)
	12-<24	3,244	104 (3.2)	78 (75.0)
	24+	2,035	170 (8.4)	135 (79.4)
**Ethnic Group**				
2017/18	White	15,638	524 (3.4)	274 (52.3)
	Mixed	431	14 (3.2)	<10 [6]
	Asian or Asian British	813	32 (3.9)	17 (53.1)
	Black or Black British	404	13 (3.2)	<10 [6]
	Chinese and Other	214	10 (4.7)	<10 [6]
	Unclassified	372	15 (4.0)	<10 [6]
2018/19	White	14,911	509 (3.4)	281 (55.2)
	Mixed	371	10 (2.7)	<10 [6]
	Asian or Asian British	726	31 (4.3)	17 (54.8)
	Black or Black British	364	16 (4.4)	<10 [6]
	Chinese and Other	167	<10 [6]	<10 [6]
	Unclassified	409	<10 [6]	<10 [6]
2019/20	White	16,606	499 (3.0)	294 (58.9)
	Mixed	409	11 (2.7)	<10 [6]
	Asian or Asian British	755	46 (6.1)	29 (63.0)
	Black or Black British	451	21 (4.7)	12 (57.1)
	Chinese and Other	163	<10 [6]	<10 [6]
	Unclassified	387	<10 [6]	<10 [6]

[1]Eligible for indicator; [2]Achieve in current prison; [3]Achieve in previous prison; [4]Overall achievement - either current or previous prison; [5]Declined indicator; [6]Suppressed (<10) to avoid disclosure

Indicator	The proportion of people with diabetes without moderate or severe frailty, in whom the last HbA1c is 58 mmol/mol or less in the preceding 12 months					Community achievement 2019/20: 56.66% (QOF 19/20)
	Group: Diabetes, Asthma & Epilepsy Care					
	**Variable**	**Population**	**Eligible[1] (% popln)**	**Satisfy[2] (% eligible)**	**Elsewhere[3] (% eligible)**	**Achieve[4] (% eligible)**
**Year**						
	2017/18	21,677	666 (3.1)	215 (32.3)	16 (2.4)	231 (34.7)
	2018/19	22,099	706 (3.2)	233 (33.0)	17 (2.4)	250 (35.4)
	2019/20	25,811	756 (2.9)	242 (32.0)	18 (2.4)	260 (34.4)
**Prison**						
2017/18	Prison 1	1,323	25 (1.9)	10 (40.0)	<10 [6]	11 (44.0)
	Prison 2	3,261	96 (2.9)	26 (27.1)	<10 [6]	26 (27.1)
	Prison 3	2,623	65 (2.5)	19 (29.2)	<10 [6]	26 (40.0)
	Prison 4	2,089	67 (3.2)	25 (37.3)	<10 [6]	26 (38.8)
	Prison 5	637	22 (3.5)	<10 [6]	<10 [6]	<10 [6]
	Prison 6	1,552	54 (3.5)	31 (57.4)	<10 [6]	31 (57.4)
	Prison 7	635	21 (3.3)	<10 [6]	<10 [6]	<10 [6]
	Prison 8	1,085	29 (2.7)	<10 [6]	<10 [6]	<10 [6]
	Prison 9	981	86 (8.8)	38 (44.2)	<10 [6]	40 (46.5)
	Prison 10	2,523	75 (3.0)	15 (20.0)	<10 [6]	15 (20.0)
	Prison 11	3,470	80 (2.3)	11 (13.8)	<10 [6]	15 (18.8)
	Prison 12	815	<10 [6]	<10 [6]	<10 [6]	<10 [6]
	Prison 13	683	42 (6.1)	16 (38.1)	<10 [6]	16 (38.1)
2018/19	Prison 1	1,333	19 (1.4)	10 (52.6)	<10 [6]	10 (52.6)
	Prison 2	2,705	73 (2.7)	19 (26.0)	<10 [6]	21 (28.8)
	Prison 3	2,522	81 (3.2)	26 (32.1)	<10 [6]	28 (34.6)
	Prison 4	2,349	75 (3.2)	28 (37.3)	<10 [6]	29 (38.7)
	Prison 5	676	22 (3.3)	<10 [6]	<10 [6]	<10 [6]
	Prison 6	1,513	48 (3.2)	23 (47.9)	<10 [6]	25 (52.1)
	Prison 7	654	19 (2.9)	<10 [6]	<10 [6]	<10 [6]
	Prison 8	1,148	34 (3.0)	<10 [6]	<10 [6]	<10 [6]
	Prison 9	996	105 (10.5)	53 (50.5)	<10 [6]	55 (52.4)
	Prison 10	2,717	77 (2.8)	14 (18.2)	<10 [6]	15 (19.5)
	Prison 11	4,020	111 (2.8)	20 (18.0)	<10 [6]	24 (21.6)
	Prison 12	792	<10 [6]	<10 [6]	<10 [6]	<10 [6]
	Prison 13	674	39 (5.8)	13 (33.3)	<10 [6]	15 (38.5)
2019/20	Prison 1	1,410	37 (2.6)	15 (40.5)	<10 [6]	16 (43.2)
	Prison 2	2,979	74 (2.5)	27 (36.5)	<10 [6]	28 (37.8)
	Prison 3	2,809	83 (3.0)	33 (39.8)	<10 [6]	36 (43.4)
	Prison 4	2,651	<10 [6]	<10 [6]	<10 [6]	<10 [6]
	Prison 5	616	25 (4.1)	<10 [6]	<10 [6]	13 (52.0)
	Prison 6	1,533	51 (3.3)	28 (54.9)	<10 [6]	30 (58.8)
	Prison 7	860	29 (3.4)	12 (41.4)	<10 [6]	12 (41.4)
	Prison 8	1,385	44 (3.2)	10 (22.7)	<10 [6]	11 (25.0)
	Prison 9	1,092	125 (11.4)	58 (46.4)	<10 [6]	61 (48.8)
	Prison 10	3,577	97 (2.7)	22 (22.7)	<10 [6]	22 (22.7)
	Prison 11	5,348	135 (2.5)	15 (11.1)	<10 [6]	18 (13.3)
	Prison 12	805	<10 [6]	<10 [6]	<10 [6]	<10 [6]
	Prison 13	746	46 (6.2)	10 (21.7)	<10 [6]	10 (21.7)
**Prison category**						
2017/18	A	1,664	128 (7.7)	54 (42.2)	<10 [6]	56 (43.8)
	B	9,254	251 (2.7)	52 (20.7)	<10 [6]	56 (22.3)
	C	6,035	157 (2.6)	54 (34.4)	<10 [6]	63 (40.1)
	Closed	1,720	50 (2.9)	15 (30.0)	<10 [6]	16 (32.0)
	D	2,189	76 (3.5)	38 (50.0)	<10 [6]	38 (50.0)
	YOI	815	<10 [6]	<10 [6]	<10 [6]	<10 [6]
2018/19	A	1,670	144 (8.6)	66 (45.8)	<10 [6]	70 (48.6)
	B	9,442	261 (2.8)	53 (20.3)	<10 [6]	60 (23.0)
	C	6,204	175 (2.8)	64 (36.6)	<10 [6]	67 (38.3)
	Closed	1,802	53 (2.9)	17 (32.1)	<10 [6]	18 (34.0)
	D	2,189	70 (3.2)	31 (44.3)	<10 [6]	33 (47.1)
	YOI	792	<10 [6]	<10 [6]	<10 [6]	<10 [6]
2019/20	A	1,838	171 (9.3)	68 (39.8)	<10 [6]	71 (41.5)
	B	11,904	306 (2.6)	64 (20.9)	<10 [6]	68 (22.2)
	C	6,870	126 (1.8)	51 (40.5)	<10 [6]	55 (43.7)
	Closed	2,245	73 (3.3)	22 (30.1)	<10 [6]	23 (31.5)
	D	2,149	76 (3.5)	37 (48.7)	<10 [6]	43 (56.6)
	YOI	805	<10 [6]	<10 [6]	<10 [6]	<10 [6]
**Gender**						
2017/18	F	1,699	50 (2.9)	15 (30.0)	<10 [6]	16 (32.0)
	M	19,977	616 (3.1)	200 (32.5)	15 (2.4)	215 (34.9)
2018/19	F	1,802	52 (2.9)	17 (32.7)	<10 [6]	18 (34.6)
	M	20,295	654 (3.2)	216 (33.0)	16 (2.4)	232 (35.5)
2019/20	F	1,376	44 (3.2)	10 (22.7)	<10 [6]	11 (25.0)
	M	23,570	683 (2.9)	220 (32.2)	17 (2.5)	237 (34.7)

[1]Eligible for indicator; [2]Achieve in current prison; [3]Achieve in previous prison; [4]Overall achievement - either current or previous prison; [5]Declined indicator; [6]Suppressed (<10) to avoid disclosure

Indicator	*The proportion of people with diabetes without moderate or severe frailty, in whom the last HbA1c is 58 mmol/mol or less in the preceding 12 months*					*Community achievement 2019/20: 56.66% (QOF 19/20)*
	**Group: Diabetes, Asthma & Epilepsy Care**					

	**Variable**	**Population**	**Eligible[1] (% popln)**	**Satisfy[2] (% eligible)**	**Elsewhere[3] (% eligible)**	**Achieve[4] (% eligible)**
**Sentence Status**						
2017/18	.	81	<10 [6]	<10 [6]	<10 [6]	<10 [6]
	Absconded	<10 [6]	<10 [6]	<10 [6]	<10 [6]	<10 [6]
	Active In	15,872	486 (3.1)	159 (32.7)	16 (3.3)	175 (36.0)
	Active Out	1,052	33 (3.1)	13 (39.4)	<10 [6]	13 (39.4)
	Convicted Sentence	2,125	68 (3.2)	19 (27.9)	<10 [6]	19 (27.9)
	Downgrade in security category	<10 [6]	<10 [6]	<10 [6]	<10 [6]	<10 [6]
	Internal Cell Move	<10 [6]	<10 [6]	<10 [6]	<10 [6]	<10 [6]
	Judges Remand	92	<10 [6]	<10 [6]	<10 [6]	<10 [6]
	Licence Revoke	52	<10 [6]	<10 [6]	<10 [6]	<10 [6]
	On Remand	1,492	38 (2.5)	<10 [6]	<10 [6]	<10 [6]
	Transfer	910	31 (3.4)	15 (48.4)	<10 [6]	15 (48.4)
	Upgrade in security category	<10 [6]	<10 [6]	<10 [6]	<10 [6]	<10 [6]
2018/19	.	<10 [6]	<10 [6]	<10 [6]	<10 [6]	<10 [6]
	Absconded	<10 [6]	<10 [6]	<10 [6]	<10 [6]	<10 [6]
	Active In	18,145	583 (3.2)	197 (33.8)	16 (2.7)	213 (36.5)
	Active Out	835	33 (4.0)	11 (33.3)	<10 [6]	11 (33.3)
	Convicted Sentence	1,320	38 (2.9)	12 (31.6)	<10 [6]	12 (31.6)
	Downgrade in security category	<10 [6]	<10 [6]	<10 [6]	<10 [6]	<10 [6]
	Internal Cell Move	<10 [6]	<10 [6]	<10 [6]	<10 [6]	<10 [6]
	Judges Remand	<10 [6]	<10 [6]	<10 [6]	<10 [6]	<10 [6]
	Licence Revoke	125	<10 [6]	<10 [6]	<10 [6]	<10 [6]
	On Remand	1,059	27 (2.5)	<10 [6]	<10 [6]	<10 [6]
	Transfer	518	19 (3.7)	<10 [6]	<10 [6]	<10 [6]
	Upgrade in security category	<10 [6]	<10 [6]	<10 [6]	<10 [6]	<10 [6]
2019/20	.	69	<10 [6]	<10 [6]	<10 [6]	<10 [6]
	Absconded	<10 [6]	<10 [6]	<10 [6]	<10 [6]	<10 [6]
	Active In	22,424	664 (3.0)	212 (31.9)	18 (2.7)	230 (34.6)
	Active Out	625	25 (4.0)	<10 [6]	<10 [6]	<10 [6]
	Convicted Sentence	1,361	33 (2.4)	12 (36.4)	<10 [6]	12 (36.4)
	Downgrade in security category	<10 [6]	<10 [6]	<10 [6]	<10 [6]	<10 [6]
	Internal Cell Move	<10 [6]	<10 [6]	<10 [6]	<10 [6]	<10 [6]
	Judges Remand	19	<10 [6]	<10 [6]	<10 [6]	<10 [6]
	Licence Revoke	178	<10 [6]	<10 [6]	<10 [6]	<10 [6]
	On Remand	1,031	23 (2.2)	10 (43.5)	<10 [6]	10 (43.5)
	Transfer	101	<10 [6]	<10 [6]	<10 [6]	<10 [6]
	Upgrade in security category	<10 [6]	<10 [6]	<10 [6]	<10 [6]	<10 [6]
**Age - years**						
2017/18	10 - <20	468	<10 [6]	<10 [6]	<10 [6]	<10 [6]
	20 - <30	6,994	38 (0.5)	<10 [6]	<10 [6]	<10 [6]
	30 - <40	7,051	77 (1.1)	15 (19.5)	<10 [6]	16 (20.8)
	40 - <50	4,114	150 (3.6)	42 (28.0)	<10 [6]	43 (28.7)
	50 - <60	2,107	217 (10.3)	77 (35.5)	11 (5.1)	88 (40.6)
	60 - <70	684	123 (18.0)	46 (37.4)	<10 [6]	48 (39.0)
	70 - <80	213	48 (22.5)	24 (50.0)	<10 [6]	25 (52.1)
	80 - <90	40	10 (25.0)	<10 [6]	<10 [6]	<10 [6]
	90 - <100	<10 [6]	<10 [6]	<10 [6]	<10 [6]	<10 [6]
	100 - <110	<10 [6]	<10 [6]	<10 [6]	<10 [6]	<10 [6]
2018/19	10 - <20	436	<10 [6]	<10 [6]	<10 [6]	<10 [6]
	20 - <30	7,163	50 (0.7)	10 (20.0)	<10 [6]	10 (20.0)
	30 - <40	7,381	106 (1.4)	22 (20.8)	<10 [6]	27 (25.5)
	40 - <50	4,180	148 (3.5)	41 (27.7)	<10 [6]	42 (28.4)
	50 - <60	1,978	212 (10.7)	78 (36.8)	<10 [6]	83 (39.2)
	60 - <70	701	132 (18.8)	53 (40.2)	<10 [6]	57 (43.2)
	70 - <80	209	48 (23.0)	23 (47.9)	<10 [6]	25 (52.1)
	80 - <90	45	<10 [6]	<10 [6]	<10 [6]	<10 [6]
	90 - <100	<10 [6]	<10 [6]	<10 [6]	<10 [6]	<10 [6]
	100 - <110	<10 [6]	<10 [6]	<10 [6]	<10 [6]	<10 [6]
2019/20	10 - <20	404	<10 [6]	<10 [6]	<10 [6]	<10 [6]
	20 - <30	8,064	60 (0.7)	<10 [6]	<10 [6]	<10 [6]
	30 - <40	9,125	100 (1.1)	19 (19.0)	<10 [6]	20 (20.0)
	40 - <50	4,948	194 (3.9)	60 (30.9)	10 (5.2)	70 (36.1)
	50 - <60	2,224	192 (8.6)	66 (34.4)	<10 [6]	70 (36.5)
	60 - <70	751	148 (19.7)	67 (45.3)	<10 [6]	69 (46.6)
	70 - <80	238	53 (22.3)	18 (34.0)	<10 [6]	19 (35.8)
	80 - <90	53	<10 [6]	<10 [6]	<10 [6]	<10 [6]
	90 - <100	<10 [6]	<10 [6]	<10 [6]	<10 [6]	<10 [6]
	100 - <110	<10 [6]	<10 [6]	<10 [6]	<10 [6]	<10 [6]

[1]Eligible for indicator; [2]Achieve in current prison; [3]Achieve in previous prison; [4]Overall achievement - either current or previous prison; [5]Declined indicator; [6]Suppressed (<10) to avoid disclosure

Indicator	The proportion of people with diabetes without moderate or severe frailty, in whom the last HbA1c is 58 mmol/mol or less in the preceding 12 months	Community achievement 2019/20: 56.66% (QOF 19/20)

Group: Diabetes, Asthma & Epilepsy Care

	Variable	Population	Eligible[1] (% popln)	Satisfy[2] (% eligible)	Elsewhere[3] (% eligible)	Achieve[4] (% eligible)
**Length of Stay (months)**						
2017/18	<1	4,474	92 (2.1)	<10 [6]	<10 [6]	10 (10.9)
	1-<6	8,075	177 (2.2)	26 (14.7)	<10 [6]	30 (16.9)
	6-<12	3,672	104 (2.8)	49 (47.1)	<10 [6]	52 (50.0)
	12-<24	2,832	93 (3.3)	41 (44.1)	<10 [6]	42 (45.2)
	24+	2,624	200 (7.6)	94 (47.0)	<10 [6]	97 (48.5)
2018/19	<1	4,801	97 (2.0)	<10 [6]	<10 [6]	<10 [6]
	1-<6	7,742	183 (2.4)	40 (21.9)	<10 [6]	48 (26.2)
	6-<12	3,616	103 (2.8)	42 (40.8)	<10 [6]	42 (40.8)
	12-<24	3,447	115 (3.3)	50 (43.5)	<10 [6]	57 (49.6)
	24+	2,493	208 (8.3)	99 (47.6)	<10 [6]	99 (47.6)
2019/20	<1	5,745	134 (2.3)	<10 [6]	<10 [6]	<10 [6]
	1-<6	9,697	214 (2.2)	57 (26.6)	<10 [6]	65 (30.4)
	6-<12	5,090	143 (2.8)	66 (46.2)	<10 [6]	72 (50.3)
	12-<24	3,244	100 (3.1)	45 (45.0)	<10 [6]	46 (46.0)
	24+	2,035	165 (8.1)	73 (44.2)	<10 [6]	73 (44.2)
Ethnic Group						
2017/18	White	15,638	518 (3.3)	167 (32.2)	12 (2.3)	179 (34.6)
	Mixed	431	14 (3.2)	<10 [6]	<10 [6]	<10 [6]
	Asian or Asian British	813	32 (3.9)	11 (34.4)	<10 [6]	14 (43.8)
	Black or Black British	404	13 (3.2)	<10 [6]	<10 [6]	10 (76.9)
	Chinese and Other	214	10 (4.7)	<10 [6]	<10 [6]	<10 [6]
	Unclassified	372	14 (3.8)	<10 [6]	<10 [6]	<10 [6]
2018/19	White	14,911	503 (3.4)	162 (32.2)	13 (2.6)	175 (34.8)
	Mixed	371	10 (2.7)	<10 [6]	<10 [6]	<10 [6]
	Asian or Asian British	726	30 (4.1)	11 (36.7)	<10 [6]	11 (36.7)
	Black or Black British	364	16 (4.4)	<10 [6]	<10 [6]	<10 [6]
	Chinese and Other	167	<10 [6]	<10 [6]	<10 [6]	<10 [6]
	Unclassified	409	<10 [6]	<10 [6]	<10 [6]	<10 [6]
2019/20	White	16,606	486 (2.9)	168 (34.6)	10 (2.1)	178 (36.6)
	Mixed	409	11 (2.7)	<10 [6]	<10 [6]	<10 [6]
	Asian or Asian British	755	45 (6.0)	16 (35.6)	<10 [6]	18 (40.0)
	Black or Black British	451	21 (4.7)	10 (47.6)	<10 [6]	10 (47.6)
	Chinese and Other	163	<10 [6]	<10 [6]	<10 [6]	<10 [6]
	Unclassified	387	<10 [6]	<10 [6]	<10 [6]	<10 [6]

[1]Eligible for indicator; [2]Achieve in current prison; [3]Achieve in previous prison; [4]Overall achievement - either current or previous prison; [5]Declined indicator; [6]Suppressed (<10) to avoid disclosure

Indicator	*The proportion of people with diabetes and moderate or severe frailty, in whom the last HbA1c is 75 mmol/mol or less in the preceding 12 months*					*Community achievement 2019/20: 83.34% (QOF 19/20)*
	**Group: Diabetes, Asthma & Epilepsy Care**					

	**Variable**	**Population**	**Eligible[1] (% popln)**	**Satisfy[2] (% eligible)**	**Elsewhere[3] (% eligible)**	**Achieve[4] (% eligible)**
**Year**						
	2017/18	21,677	<10 [6]	<10 [6]	<10 [6]	<10 [6]
	2018/19	22,099	<10 [6]	<10 [6]	<10 [6]	<10 [6]
	2019/20	25,811	14 (0.1)	<10 [6]	<10 [6]	<10 [6]
**Prison**						
2017/18	Prison 1	1,323	<10 [6]	<10 [6]	<10 [6]	<10 [6]
	Prison 2	3,261	<10 [6]	<10 [6]	<10 [6]	<10 [6]
	Prison 3	2,623	<10 [6]	<10 [6]	<10 [6]	<10 [6]
	Prison 4	2,089	<10 [6]	<10 [6]	<10 [6]	<10 [6]
	Prison 5	637	<10 [6]	<10 [6]	<10 [6]	<10 [6]
	Prison 6	1,552	<10 [6]	<10 [6]	<10 [6]	<10 [6]
	Prison 7	635	<10 [6]	<10 [6]	<10 [6]	<10 [6]
	Prison 8	1,085	<10 [6]	<10 [6]	<10 [6]	<10 [6]
	Prison 9	981	<10 [6]	<10 [6]	<10 [6]	<10 [6]
	Prison 10	2,523	<10 [6]	<10 [6]	<10 [6]	<10 [6]
	Prison 11	3,470	<10 [6]	<10 [6]	<10 [6]	<10 [6]
	Prison 12	815	<10 [6]	<10 [6]	<10 [6]	<10 [6]
	Prison 13	683	<10 [6]	<10 [6]	<10 [6]	<10 [6]
2018/19	Prison 1	1,333	<10 [6]	<10 [6]	<10 [6]	<10 [6]
	Prison 2	2,705	<10 [6]	<10 [6]	<10 [6]	<10 [6]
	Prison 3	2,522	<10 [6]	<10 [6]	<10 [6]	<10 [6]
	Prison 4	2,349	<10 [6]	<10 [6]	<10 [6]	<10 [6]
	Prison 5	676	<10 [6]	<10 [6]	<10 [6]	<10 [6]
	Prison 6	1,513	<10 [6]	<10 [6]	<10 [6]	<10 [6]
	Prison 7	654	<10 [6]	<10 [6]	<10 [6]	<10 [6]
	Prison 8	1,148	<10 [6]	<10 [6]	<10 [6]	<10 [6]
	Prison 9	996	<10 [6]	<10 [6]	<10 [6]	<10 [6]
	Prison 10	2,717	<10 [6]	<10 [6]	<10 [6]	<10 [6]
	Prison 11	4,020	<10 [6]	<10 [6]	<10 [6]	<10 [6]
	Prison 12	792	<10 [6]	<10 [6]	<10 [6]	<10 [6]
	Prison 13	674	<10 [6]	<10 [6]	<10 [6]	<10 [6]
2019/20	Prison 1	1,410	<10 [6]	<10 [6]	<10 [6]	<10 [6]
	Prison 2	2,979	<10 [6]	<10 [6]	<10 [6]	<10 [6]
	Prison 3	2,809	<10 [6]	<10 [6]	<10 [6]	<10 [6]
	Prison 4	2,651	<10 [6]	<10 [6]	<10 [6]	<10 [6]
	Prison 5	616	<10 [6]	<10 [6]	<10 [6]	<10 [6]
	Prison 6	1,533	<10 [6]	<10 [6]	<10 [6]	<10 [6]
	Prison 7	860	<10 [6]	<10 [6]	<10 [6]	<10 [6]
	Prison 8	1,385	<10 [6]	<10 [6]	<10 [6]	<10 [6]
	Prison 9	1,092	<10 [6]	<10 [6]	<10 [6]	<10 [6]
	Prison 10	3,577	<10 [6]	<10 [6]	<10 [6]	<10 [6]
	Prison 11	5,348	<10 [6]	<10 [6]	<10 [6]	<10 [6]
	Prison 12	805	<10 [6]	<10 [6]	<10 [6]	<10 [6]
	Prison 13	746	<10 [6]	<10 [6]	<10 [6]	<10 [6]
**Prison category**						
2017/18	A	1,664	<10 [6]	<10 [6]	<10 [6]	<10 [6]
	B	9,254	<10 [6]	<10 [6]	<10 [6]	<10 [6]
	C	6,035	<10 [6]	<10 [6]	<10 [6]	<10 [6]
	Closed	1,720	<10 [6]	<10 [6]	<10 [6]	<10 [6]
	D	2,189	<10 [6]	<10 [6]	<10 [6]	<10 [6]
	YOI	815	<10 [6]	<10 [6]	<10 [6]	<10 [6]
2018/19	A	1,670	<10 [6]	<10 [6]	<10 [6]	<10 [6]
	B	9,442	<10 [6]	<10 [6]	<10 [6]	<10 [6]
	C	6,204	<10 [6]	<10 [6]	<10 [6]	<10 [6]
	Closed	1,802	<10 [6]	<10 [6]	<10 [6]	<10 [6]
	D	2,189	<10 [6]	<10 [6]	<10 [6]	<10 [6]
	YOI	792	<10 [6]	<10 [6]	<10 [6]	<10 [6]
2019/20	A	1,838	<10 [6]	<10 [6]	<10 [6]	<10 [6]
	B	11,904	<10 [6]	<10 [6]	<10 [6]	<10 [6]
	C	6,870	<10 [6]	<10 [6]	<10 [6]	<10 [6]
	Closed	2,245	<10 [6]	<10 [6]	<10 [6]	<10 [6]
	D	2,149	<10 [6]	<10 [6]	<10 [6]	<10 [6]
	YOI	805	<10 [6]	<10 [6]	<10 [6]	<10 [6]
**Gender**						
2017/18	F	1,699	<10 [6]	<10 [6]	<10 [6]	<10 [6]
	M	19,977	<10 [6]	<10 [6]	<10 [6]	<10 [6]
2018/19	F	1,802	<10 [6]	<10 [6]	<10 [6]	<10 [6]
	M	20,295	<10 [6]	<10 [6]	<10 [6]	<10 [6]
2019/20	F	1,376	<10 [6]	<10 [6]	<10 [6]	<10 [6]
	M	23,570	14 (0.1)	<10 [6]	<10 [6]	<10 [6]

[1]Eligible for indicator; [2]Achieve in current prison; [3]Achieve in previous prison; [4]Overall achievement - either current or previous prison; [5]Declined indicator; [6]Suppressed (<10) to avoid disclosure

Indicator *The proportion of people with diabetes and moderate or severe frailty, in whom the last HbA1c is 75 mmol/mol or less in the preceding 12 months*
Group: Diabetes, Asthma & Epilepsy Care

Community achievement 2019/20: 83.34% (QOF 19/20)

	Variable	Population	Eligible[1] (% popln)	Satisfy[2] (% eligible)	Elsewhere[3] (% eligible)	Achieve[4] (% eligible)
**Sentence Status**						
2017/18	.	81	<10 [6]	<10 [6]	<10 [6]	<10 [6]
	Absconded	<10 [6]	<10 [6]	<10 [6]	<10 [6]	<10 [6]
	Active In	15,872	<10 [6]	<10 [6]	<10 [6]	<10 [6]
	Active Out	1,052	<10 [6]	<10 [6]	<10 [6]	<10 [6]
	Convicted Sentence	2,125	<10 [6]	<10 [6]	<10 [6]	<10 [6]
	Downgrade in security category	<10 [6]	<10 [6]	<10 [6]	<10 [6]	<10 [6]
	Internal Cell Move	<10 [6]	<10 [6]	<10 [6]	<10 [6]	<10 [6]
	Judges Remand	92	<10 [6]	<10 [6]	<10 [6]	<10 [6]
	Licence Revoke	52	<10 [6]	<10 [6]	<10 [6]	<10 [6]
	On Remand	1,492	<10 [6]	<10 [6]	<10 [6]	<10 [6]
	Transfer	910	<10 [6]	<10 [6]	<10 [6]	<10 [6]
	Upgrade in security category	<10 [6]	<10 [6]	<10 [6]	<10 [6]	<10 [6]
2018/19	.	88	<10 [6]	<10 [6]	<10 [6]	<10 [6]
	Absconded	<10 [6]	<10 [6]	<10 [6]	<10 [6]	<10 [6]
	Active In	18,145	<10 [6]	<10 [6]	<10 [6]	<10 [6]
	Active Out	835	<10 [6]	<10 [6]	<10 [6]	<10 [6]
	Convicted Sentence	1,320	<10 [6]	<10 [6]	<10 [6]	<10 [6]
	Downgrade in security category	<10 [6]	<10 [6]	<10 [6]	<10 [6]	<10 [6]
	Internal Cell Move	<10 [6]	<10 [6]	<10 [6]	<10 [6]	<10 [6]
	Judges Remand	<10 [6]	<10 [6]	<10 [6]	<10 [6]	<10 [6]
	Licence Revoke	125	<10 [6]	<10 [6]	<10 [6]	<10 [6]
	On Remand	1,059	<10 [6]	<10 [6]	<10 [6]	<10 [6]
	Transfer	518	<10 [6]	<10 [6]	<10 [6]	<10 [6]
	Upgrade in security category	<10 [6]	<10 [6]	<10 [6]	<10 [6]	<10 [6]
2019/20	.	69	<10 [6]	<10 [6]	<10 [6]	<10 [6]
	Absconded	<10 [6]	<10 [6]	<10 [6]	<10 [6]	<10 [6]
	Active In	22,424	<10 [6]	<10 [6]	<10 [6]	<10 [6]
	Active Out	625	<10 [6]	<10 [6]	<10 [6]	<10 [6]
	Convicted Sentence	1,361	<10 [6]	<10 [6]	<10 [6]	<10 [6]
	Downgrade in security category	<10 [6]	<10 [6]	<10 [6]	<10 [6]	<10 [6]
	Internal Cell Move	<10 [6]	<10 [6]	<10 [6]	<10 [6]	<10 [6]
	Judges Remand	19	<10 [6]	<10 [6]	<10 [6]	<10 [6]
	Licence Revoke	178	<10 [6]	<10 [6]	<10 [6]	<10 [6]
	On Remand	1,031	<10 [6]	<10 [6]	<10 [6]	<10 [6]
	Transfer	101	<10 [6]	<10 [6]	<10 [6]	<10 [6]
	Upgrade in security category	<10 [6]	<10 [6]	<10 [6]	<10 [6]	<10 [6]
**Age - years**						
2017/18	10 - <20	468	<10 [6]	<10 [6]	<10 [6]	<10 [6]
	20 - <30	6,994	<10 [6]	<10 [6]	<10 [6]	<10 [6]
	30 - <40	7,051	<10 [6]	<10 [6]	<10 [6]	<10 [6]
	40 - <50	4,114	<10 [6]	<10 [6]	<10 [6]	<10 [6]
	50 - <60	2,107	<10 [6]	<10 [6]	<10 [6]	<10 [6]
	60 - <70	684	<10 [6]	<10 [6]	<10 [6]	<10 [6]
	70 - <80	213	<10 [6]	<10 [6]	<10 [6]	<10 [6]
	80 - <90	40	<10 [6]	<10 [6]	<10 [6]	<10 [6]
	90 - <100	<10 [6]	<10 [6]	<10 [6]	<10 [6]	<10 [6]
	100 - <110	<10 [6]	<10 [6]	<10 [6]	<10 [6]	<10 [6]
2018/19	10 - <20	436	<10 [6]	<10 [6]	<10 [6]	<10 [6]
	20 - <30	7,163	<10 [6]	<10 [6]	<10 [6]	<10 [6]
	30 - <40	7,381	<10 [6]	<10 [6]	<10 [6]	<10 [6]
	40 - <50	4,180	<10 [6]	<10 [6]	<10 [6]	<10 [6]
	50 - <60	1,978	<10 [6]	<10 [6]	<10 [6]	<10 [6]
	60 - <70	701	<10 [6]	<10 [6]	<10 [6]	<10 [6]
	70 - <80	209	<10 [6]	<10 [6]	<10 [6]	<10 [6]
	80 - <90	45	<10 [6]	<10 [6]	<10 [6]	<10 [6]
	90 - <100	<10 [6]	<10 [6]	<10 [6]	<10 [6]	<10 [6]
	100 - <110	<10 [6]	<10 [6]	<10 [6]	<10 [6]	<10 [6]
2019/20	10 - <20	404	<10 [6]	<10 [6]	<10 [6]	<10 [6]
	20 - <30	8,064	<10 [6]	<10 [6]	<10 [6]	<10 [6]
	30 - <40	9,125	<10 [6]	<10 [6]	<10 [6]	<10 [6]
	40 - <50	4,948	<10 [6]	<10 [6]	<10 [6]	<10 [6]
	50 - <60	2,224	<10 [6]	<10 [6]	<10 [6]	<10 [6]
	60 - <70	751	<10 [6]	<10 [6]	<10 [6]	<10 [6]
	70 - <80	238	<10 [6]	<10 [6]	<10 [6]	<10 [6]
	80 - <90	53	<10 [6]	<10 [6]	<10 [6]	<10 [6]
	90 - <100	<10 [6]	<10 [6]	<10 [6]	<10 [6]	<10 [6]
	100 - <110	<10 [6]	<10 [6]	<10 [6]	<10 [6]	<10 [6]

[1]Eligible for indicator; [2]Achieve in current prison; [3]Achieve in previous prison; [4]Overall achievement - either current or previous prison; [5]Declined indicator; [6]Suppressed (<10) to avoid disclosure

Indicator	The proportion of people with diabetes and moderate or severe frailty, in whom the last HbA1c is 75 mmol/mol or less in the preceding 12 months
	**Group: Diabetes, Asthma & Epilepsy Care**

Community achievement 2019/20: 83.34% (QOF 19/20)

	Variable	Population	Eligible[1] (% popln)	Satisfy[2] (% eligible)	Elsewhere[3] (% eligible)	Achieve[4] (% eligible)
**Length of Stay (months)**						
2017/18	<1	4,474	<10 [6]	<10 [6]	<10 [6]	<10 [6]
	1-<6	8,075	<10 [6]	<10 [6]	<10 [6]	<10 [6]
	6-<12	3,672	<10 [6]	<10 [6]	<10 [6]	<10 [6]
	12-<24	2,832	<10 [6]	<10 [6]	<10 [6]	<10 [6]
	24+	2,624	<10 [6]	<10 [6]	<10 [6]	<10 [6]
2018/19	<1	4,801	<10 [6]	<10 [6]	<10 [6]	<10 [6]
	1-<6	7,742	<10 [6]	<10 [6]	<10 [6]	<10 [6]
	6-<12	3,616	<10 [6]	<10 [6]	<10 [6]	<10 [6]
	12-<24	3,447	<10 [6]	<10 [6]	<10 [6]	<10 [6]
	24+	2,493	<10 [6]	<10 [6]	<10 [6]	<10 [6]
2019/20	<1	5,745	<10 [6]	<10 [6]	<10 [6]	<10 [6]
	1-<6	9,697	<10 [6]	<10 [6]	<10 [6]	<10 [6]
	6-<12	5,090	<10 [6]	<10 [6]	<10 [6]	<10 [6]
	12-<24	3,244	<10 [6]	<10 [6]	<10 [6]	<10 [6]
	24+	2,035	<10 [6]	<10 [6]	<10 [6]	<10 [6]
**Ethnic Group**						
2017/18	White	15,638	<10 [6]	<10 [6]	<10 [6]	<10 [6]
	Mixed	431	<10 [6]	<10 [6]	<10 [6]	<10 [6]
	Asian or Asian British	813	<10 [6]	<10 [6]	<10 [6]	<10 [6]
	Black or Black British	404	<10 [6]	<10 [6]	<10 [6]	<10 [6]
	Chinese and Other	214	<10 [6]	<10 [6]	<10 [6]	<10 [6]
	Unclassified	372	<10 [6]	<10 [6]	<10 [6]	<10 [6]
2018/19	White	14,911	<10 [6]	<10 [6]	<10 [6]	<10 [6]
	Mixed	371	<10 [6]	<10 [6]	<10 [6]	<10 [6]
	Asian or Asian British	726	<10 [6]	<10 [6]	<10 [6]	<10 [6]
	Black or Black British	364	<10 [6]	<10 [6]	<10 [6]	<10 [6]
	Chinese and Other	167	<10 [6]	<10 [6]	<10 [6]	<10 [6]
	Unclassified	409	<10 [6]	<10 [6]	<10 [6]	<10 [6]
2019/20	White	16,606	13 (0.1)	<10 [6]	<10 [6]	<10 [6]
	Mixed	409	<10 [6]	<10 [6]	<10 [6]	<10 [6]
	Asian or Asian British	755	<10 [6]	<10 [6]	<10 [6]	<10 [6]
	Black or Black British	451	<10 [6]	<10 [6]	<10 [6]	<10 [6]
	Chinese and Other	163	<10 [6]	<10 [6]	<10 [6]	<10 [6]
	Unclassified	387	<10 [6]	<10 [6]	<10 [6]	<10 [6]

[1]Eligible for indicator; [2]Achieve in current prison; [3]Achieve in previous prison; [4]Overall achievement - either current or previous prison; [5]Declined indicator; [6]Suppressed (<10) to avoid disclosure

**Indicator**	*The proportion of people with asthma who have had an asthma review in the preceding 12 months that includes an assessment of asthma control*						*Community achievement 2019/20: 67.14% (QOF 19/20)*
	**Group: Diabetes, Asthma & Epilepsy Care**						
	**Variable**	**Population**	**Eligible[1] (% popln)**	**Satisfy[2] (% eligible)**	**Elsewhere[3] (% eligible)**	**Achieve[4] (% eligible)**	
**Year**							
	2017/18	21,677	3,227 (14.9)	530 (16.4)	73 (2.3)	599 (18.6)	
	2018/19	22,099	3,720 (16.8)	175 (4.7)	34 (0.9)	209 (5.6)	
	2019/20	25,811	4,459 (17.3)	101 (2.3)	35 (0.8)	136 (3.1)	
**Prison**							
2017/18	Prison 1	1,323	243 (18.4)	45 (18.5)	15 (6.2)	59 (24.3)	
	Prison 2	3,261	465 (14.3)	91 (19.6)	<10 [6]	95 (20.4)	
	Prison 3	2,623	297 (11.3)	15 (5.1)	<10 [6]	23 (7.7)	
	Prison 4	2,089	256 (12.3)	61 (23.8)	<10 [6]	65 (25.4)	
	Prison 5	637	60 (9.4)	<10 [6]	<10 [6]	<10 [6]	
	Prison 6	1,552	280 (18.0)	52 (18.6)	11 (3.9)	63 (22.5)	
	Prison 7	635	175 (27.6)	47 (26.9)	<10 [6]	50 (28.6)	
	Prison 8	1,085	232 (21.4)	39 (16.8)	<10 [6]	42 (18.1)	
	Prison 9	981	125 (12.7)	51 (40.8)	<10 [6]	54 (43.2)	
	Prison 10	2,523	482 (19.1)	36 (7.5)	<10 [6]	36 (7.5)	
	Prison 11	3,470	383 (11.0)	<10 [6]	17 (4.4)	17 (4.4)	
	Prison 12	815	155 (19.0)	46 (29.7)	<10 [6]	46 (29.7)	
	Prison 13	683	74 (10.8)	44 (59.5)	<10 [6]	44 (59.5)	
2018/19	Prison 1	1,333	240 (18.0)	<10 [6]	<10 [6]	<10 [6]	
	Prison 2	2,705	390 (14.4)	17 (4.4)	<10 [6]	19 (4.9)	
	Prison 3	2,522	374 (14.8)	<10 [6]	<10 [6]	<10 [6]	
	Prison 4	2,349	354 (15.1)	37 (10.5)	<10 [6]	39 (11.0)	
	Prison 5	676	72 (10.7)	<10 [6]	<10 [6]	<10 [6]	
	Prison 6	1,513	240 (15.9)	<10 [6]	<10 [6]	12 (5.0)	
	Prison 7	654	204 (31.2)	21 (10.3)	<10 [6]	24 (11.8)	
	Prison 8	1,148	340 (29.6)	14 (4.1)	<10 [6]	14 (4.1)	
	Prison 9	996	147 (14.8)	34 (23.1)	<10 [6]	35 (23.8)	
	Prison 10	2,717	486 (17.9)	<10 [6]	<10 [6]	<10 [6]	
	Prison 11	4,020	623 (15.5)	<10 [6]	11 (1.8)	11 (1.8)	
	Prison 12	792	153 (19.3)	<10 [6]	<10 [6]	<10 [6]	
	Prison 13	674	97 (14.4)	28 (28.9)	<10 [6]	29 (29.9)	
2019/20	Prison 1	1,410	256 (18.2)	<10 [6]	<10 [6]	<10 [6]	
	Prison 2	2,979	515 (17.3)	<10 [6]	<10 [6]	<10 [6]	
	Prison 3	2,809	441 (15.7)	<10 [6]	<10 [6]	11 (2.5)	
	Prison 4	2,651	465 (17.5)	24 (5.2)	<10 [6]	24 (5.2)	
	Prison 5	616	82 (13.3)	<10 [6]	<10 [6]	<10 [6]	
	Prison 6	1,533	281 (18.3)	<10 [6]	<10 [6]	<10 [6]	
	Prison 7	860	280 (32.6)	13 (4.6)	<10 [6]	15 (5.4)	
	Prison 8	1,385	372 (26.9)	<10 [6]	<10 [6]	<10 [6]	
	Prison 9	1,092	166 (15.2)	20 (12.0)	<10 [6]	21 (12.7)	
	Prison 10	3,577	577 (16.1)	<10 [6]	<10 [6]	<10 [6]	
	Prison 11	5,348	765 (14.3)	<10 [6]	<10 [6]	<10 [6]	
	Prison 12	805	134 (16.6)	<10 [6]	<10 [6]	<10 [6]	
	Prison 13	746	125 (16.8)	18 (14.4)	<10 [6]	18 (14.4)	
**Prison category**							
2017/18	A	1,664	199 (12.0)	95 (47.7)	<10 [6]	98 (49.2)	
	B	9,254	1,330 (14.4)	127 (9.5)	21 (1.6)	148 (11.1)	
	C	6,035	796 (13.2)	121 (15.2)	27 (3.4)	147 (18.5)	
	Closed	1,720	407 (23.7)	86 (21.1)	<10 [6]	92 (22.6)	
	D	2,189	340 (15.5)	55 (16.2)	13 (3.8)	68 (20.0)	
	YOI	815	155 (19.0)	46 (29.7)	<10 [6]	46 (29.7)	
2018/19	A	1,670	244 (14.6)	62 (25.4)	<10 [6]	64 (26.2)	
	B	9,442	1,499 (15.9)	18 (1.2)	13 (0.9)	31 (2.1)	
	C	6,204	968 (15.6)	46 (4.8)	<10 [6]	54 (5.6)	
	Closed	1,802	544 (30.2)	35 (6.4)	<10 [6]	38 (7.0)	
	D	2,189	312 (14.3)	<10 [6]	<10 [6]	13 (4.2)	
	YOI	792	153 (19.3)	<10 [6]	<10 [6]	<10 [6]	
2019/20	A	1,838	291 (15.8)	38 (13.1)	<10 [6]	39 (13.4)	
	B	11,904	1,857 (15.6)	<10 [6]	11 (0.6)	20 (1.1)	
	C	6,870	1,162 (16.9)	27 (2.3)	15 (1.3)	42 (3.6)	
	Closed	2,245	652 (29.0)	22 (3.4)	<10 [6]	24 (3.7)	
	D	2,149	363 (16.9)	<10 [6]	<10 [6]	<10 [6]	
	YOI	805	134 (16.6)	<10 [6]	<10 [6]	<10 [6]	
**Gender**							
2017/18	F	1,699	406 (23.9)	86 (21.2)	<10 [6]	92 (22.7)	
	M	19,977	2,821 (14.1)	444 (15.7)	67 (2.4)	507 (18.0)	
2018/19	F	1,802	544 (30.2)	35 (6.4)	<10 [6]	38 (7.0)	
	M	20,295	3,176 (15.6)	140 (4.4)	31 (1.0)	171 (5.4)	
2019/20	F	1,376	370 (26.9)	<10 [6]	<10 [6]	<10 [6]	
	M	23,570	3,809 (16.2)	79 (2.1)	33 (0.9)	112 (2.9)	

[1]Eligible for indicator; [2]Achieve in current prison; [3]Achieve in previous prison; [4]Overall achievement - either current or previous prison; [5]Declined indicator; [6]Suppressed (<10) to avoid disclosure

Indicator	*The proportion of people with asthma who have had an asthma review in the preceding 12 months that includes an assessment of asthma control*	*Community achievement 2019/20: 67.14% (QOF 19/20)*
	Group: Diabetes, Asthma & Epilepsy Care	

	Variable	Population	Eligible[1] (% popln)	Satisfy[2] (% eligible)	Elsewhere[3] (% eligible)	Achieve[4] (% eligible)
**Sentence Status**						
2017/18	.	81	12 (14.8)	<10 [6]	<10 [6]	<10 [6]
	Absconded	<10 [6]	<10 [6]	<10 [6]	<10 [6]	<10 [6]
	Active In	15,872	2,316 (14.6)	395 (17.1)	65 (2.8)	456 (19.7)
	Active Out	1,052	166 (15.8)	17 (10.2)	<10 [6]	21 (12.7)
	Convicted Sentence	2,125	336 (15.8)	41 (12.2)	<10 [6]	42 (12.5)
	Downgrade in security category	<10 [6]	<10 [6]	<10 [6]	<10 [6]	<10 [6]
	Internal Cell Move	<10 [6]	<10 [6]	<10 [6]	<10 [6]	<10 [6]
	Judges Remand	92	20 (21.7)	<10 [6]	<10 [6]	<10 [6]
	Licence Revoke	52	<10 [6]	<10 [6]	<10 [6]	<10 [6]
	On Remand	1,492	209 (14.0)	40 (19.1)	<10 [6]	42 (20.1)
	Transfer	910	160 (17.6)	34 (21.3)	<10 [6]	35 (21.9)
	Upgrade in security category	<10 [6]	<10 [6]	<10 [6]	<10 [6]	<10 [6]
2018/19	.	88	<10 [6]	<10 [6]	<10 [6]	<10 [6]
	Absconded	<10 [6]	<10 [6]	<10 [6]	<10 [6]	<10 [6]
	Active In	18,145	3,112 (17.2)	158 (5.1)	31 (1.0)	189 (6.1)
	Active Out	835	147 (17.6)	<10 [6]	<10 [6]	<10 [6]
	Convicted Sentence	1,320	198 (15.0)	11 (5.6)	<10 [6]	12 (6.1)
	Downgrade in security category	<10 [6]	<10 [6]	<10 [6]	<10 [6]	<10 [6]
	Internal Cell Move	<10 [6]	<10 [6]	<10 [6]	<10 [6]	<10 [6]
	Judges Remand	<10 [6]	<10 [6]	<10 [6]	<10 [6]	<10 [6]
	Licence Revoke	125	30 (24.0)	<10 [6]	<10 [6]	<10 [6]
	On Remand	1,059	148 (14.0)	<10 [6]	<10 [6]	<10 [6]
	Transfer	518	77 (14.9)	<10 [6]	<10 [6]	<10 [6]
	Upgrade in security category	<10 [6]	<10 [6]	<10 [6]	<10 [6]	<10 [6]
2019/20	.	69	<10 [6]	<10 [6]	<10 [6]	<10 [6]
	Absconded	<10 [6]	<10 [6]	<10 [6]	<10 [6]	<10 [6]
	Active In	22,424	3,875 (17.3)	94 (2.4)	35 (0.9)	129 (3.3)
	Active Out	625	113 (18.1)	<10 [6]	<10 [6]	<10 [6]
	Convicted Sentence	1,361	235 (17.3)	<10 [6]	<10 [6]	<10 [6]
	Downgrade in security category	<10 [6]	<10 [6]	<10 [6]	<10 [6]	<10 [6]
	Internal Cell Move	<10 [6]	<10 [6]	<10 [6]	<10 [6]	<10 [6]
	Judges Remand	19	<10 [6]	<10 [6]	<10 [6]	<10 [6]
	Licence Revoke	178	35 (19.7)	<10 [6]	<10 [6]	<10 [6]
	On Remand	1,031	174 (16.9)	<10 [6]	<10 [6]	<10 [6]
	Transfer	101	19 (18.8)	<10 [6]	<10 [6]	<10 [6]
	Upgrade in security category	<10 [6]	<10 [6]	<10 [6]	<10 [6]	<10 [6]
**Age - years**						
2017/18	10 - <20	468	68 (14.5)	17 (25.0)	<10 [6]	18 (26.5)
	20 - <30	6,994	1,023 (14.6)	126 (12.3)	19 (1.9)	142 (13.9)
	30 - <40	7,051	980 (13.9)	137 (14.0)	26 (2.7)	162 (16.5)
	40 - <50	4,114	705 (17.1)	136 (19.3)	16 (2.3)	152 (21.6)
	50 - <60	2,107	332 (15.8)	71 (21.4)	<10 [6]	79 (23.8)
	60 - <70	684	92 (13.5)	32 (34.8)	<10 [6]	35 (38.0)
	70 - <80	213	24 (11.3)	11 (45.8)	<10 [6]	11 (45.8)
	80 - <90	40	<10 [6]	<10 [6]	<10 [6]	<10 [6]
	90 - <100	<10 [6]	<10 [6]	<10 [6]	<10 [6]	<10 [6]
	100 - <110	<10 [6]	<10 [6]	<10 [6]	<10 [6]	<10 [6]
2018/19	10 - <20	436	64 (14.7)	<10 [6]	<10 [6]	<10 [6]
	20 - <30	7,163	1,143 (16.0)	22 (1.9)	13 (1.1)	35 (3.1)
	30 - <40	7,381	1,242 (16.8)	52 (4.2)	12 (1.0)	64 (5.2)
	40 - <50	4,180	784 (18.8)	42 (5.4)	<10 [6]	48 (6.1)
	50 - <60	1,978	347 (17.5)	32 (9.2)	<10 [6]	34 (9.8)
	60 - <70	701	105 (15.0)	16 (15.2)	<10 [6]	17 (16.2)
	70 - <80	209	27 (12.9)	<10 [6]	<10 [6]	<10 [6]
	80 - <90	45	<10 [6]	<10 [6]	<10 [6]	<10 [6]
	90 - <100	<10 [6]	<10 [6]	<10 [6]	<10 [6]	<10 [6]
	100 - <110	<10 [6]	<10 [6]	<10 [6]	<10 [6]	<10 [6]
2019/20	10 - <20	404	42 (10.4)	<10 [6]	<10 [6]	<10 [6]
	20 - <30	8,064	1,275 (15.8)	14 (1.1)	<10 [6]	23 (1.8)
	30 - <40	9,125	1,633 (17.9)	26 (1.6)	14 (0.9)	40 (2.4)
	40 - <50	4,948	940 (19.0)	22 (2.3)	<10 [6]	27 (2.9)
	50 - <60	2,224	396 (17.8)	21 (5.3)	<10 [6]	26 (6.6)
	60 - <70	751	117 (15.6)	13 (11.1)	<10 [6]	14 (12.0)
	70 - <80	238	43 (18.1)	<10 [6]	<10 [6]	<10 [6]
	80 - <90	53	11 (20.8)	<10 [6]	<10 [6]	<10 [6]
	90 - <100	<10 [6]	<10 [6]	<10 [6]	<10 [6]	<10 [6]
	100 - <110	<10 [6]	<10 [6]	<10 [6]	<10 [6]	<10 [6]

[1]Eligible for indicator; [2]Achieve in current prison; [3]Achieve in previous prison; [4]Overall achievement - either current or previous prison; [5]Declined indicator; [6]Suppressed (<10) to avoid disclosure

**Indicator**	***The proportion of people with asthma who have had an asthma review in the preceding 12 months that includes an assessment of asthma control***					*Community achievement 2019/20: 67.14% (QOF 19/20)*
	**Group: Diabetes, Asthma & Epilepsy Care**					
	**Variable**	**Population**	**Eligible[1] (% popln)**	**Satisfy[2] (% eligible)**	**Elsewhere[3] (% eligible)**	**Achieve[4] (% eligible)**
**Length of Stay (months)**						
2017/18	<1	4,474	588 (13.1)	12 (2.0)	10 (1.7)	22 (3.7)
	1-<6	8,075	1,193 (14.8)	116 (9.7)	28 (2.3)	141 (11.8)
	6-<12	3,672	566 (15.4)	114 (20.1)	16 (2.8)	130 (23.0)
	12-<24	2,832	447 (15.8)	127 (28.4)	12 (2.7)	138 (30.9)
	24+	2,624	433 (16.5)	161 (37.2)	<10 [6]	168 (38.8)
2018/19	<1	4,801	731 (15.2)	<10 [6]	<10 [6]	<10 [6]
	1-<6	7,742	1,332 (17.2)	13 (1.0)	11 (0.8)	24 (1.8)
	6-<12	3,616	609 (16.8)	11 (1.8)	<10 [6]	16 (2.6)
	12-<24	3,447	620 (18.0)	48 (7.7)	13 (2.1)	61 (9.8)
	24+	2,493	428 (17.2)	102 (23.8)	<10 [6]	102 (23.8)
2019/20	<1	5,745	854 (14.9)	<10 [6]	<10 [6]	<10 [6]
	1-<6	9,697	1,667 (17.2)	<10 [6]	12 (0.7)	21 (1.3)
	6-<12	5,090	974 (19.1)	11 (1.1)	16 (1.6)	27 (2.8)
	12-<24	3,244	614 (18.9)	16 (2.6)	<10 [6]	18 (2.9)
	24+	2,035	350 (17.2)	63 (18.0)	<10 [6]	63 (18.0)
**Ethnic Group**						
2017/18	White	15,638	2,619 (16.7)	427 (16.3)	59 (2.3)	484 (18.5)
	Mixed	431	78 (18.1)	15 (19.2)	<10 [6]	17 (21.8)
	Asian or Asian British	813	117 (14.4)	26 (22.2)	<10 [6]	28 (23.9)
	Black or Black British	404	58 (14.4)	<10 [6]	<10 [6]	<10 [6]
	Chinese and Other	214	14 (6.5)	<10 [6]	<10 [6]	<10 [6]
	Unclassified	372	43 (11.6)	14 (32.6)	<10 [6]	15 (34.9)
2018/19	White	14,911	2,770 (18.6)	140 (5.1)	22 (0.8)	162 (5.8)
	Mixed	371	73 (19.7)	<10 [6]	<10 [6]	<10 [6]
	Asian or Asian British	726	113 (15.6)	<10 [6]	<10 [6]	13 (11.5)
	Black or Black British	364	63 (17.3)	<10 [6]	<10 [6]	<10 [6]
	Chinese and Other	167	23 (13.8)	<10 [6]	<10 [6]	<10 [6]
	Unclassified	409	67 (16.4)	<10 [6]	<10 [6]	<10 [6]
2019/20	White	16,606	3,352 (20.2)	84 (2.5)	29 (0.9)	113 (3.4)
	Mixed	409	101 (24.7)	<10 [6]	<10 [6]	<10 [6]
	Asian or Asian British	755	129 (17.1)	<10 [6]	<10 [6]	<10 [6]
	Black or Black British	451	88 (19.5)	<10 [6]	<10 [6]	<10 [6]
	Chinese and Other	163	22 (13.5)	<10 [6]	<10 [6]	<10 [6]
	Unclassified	387	66 (17.1)	<10 [6]	<10 [6]	<10 [6]

[1]Eligible for indicator; [2]Achieve in current prison; [3]Achieve in previous prison; [4]Overall achievement - either current or previous prison; [5]Declined indicator; [6]Suppressed (<10) to avoid disclosure

Indicator	*The proportion of people on drug treatment for epilepsy who have had an annual review and recorded as seizure free in the preceding 12 months*					***no comparable data in QOF 2019/20. Most recent QOF was 2013/14 which was 57.50%***
	**Group: Diabetes, Asthma & Epilepsy Care**					
	**Variable**	**Population**	**Eligible[1] (% popln)**	**Satisfy Annual Review[4] (% eligible)**	**Satisfy Seizure Free[4] (% eligible)**	
**Year**						
	2017/18	21,677	290 (1.3)	<10 [6]	<10 [6]	
	2018/19	22,099	300 (1.4)	13 (4.3)	<10 [6]	
	2019/20	25,811	419 (1.6)	<10 [6]	<10 [6]	
**Prison**						
2017/18	Prison 1	1,323	19 (1.4)	<10 [6]	<10 [6]	
	Prison 2	3,261	40 (1.2)	<10 [6]	<10 [6]	
	Prison 3	2,623	35 (1.3)	<10 [6]	<10 [6]	
	Prison 4	2,089	30 (1.4)	<10 [6]	<10 [6]	
	Prison 5	637	<10 [6]	<10 [6]	<10 [6]	
	Prison 6	1,552	<10 [6]	<10 [6]	<10 [6]	
	Prison 7	635	19 (3.0)	<10 [6]	<10 [6]	
	Prison 8	1,085	21 (1.9)	<10 [6]	<10 [6]	
	Prison 9	981	18 (1.8)	<10 [6]	<10 [6]	
	Prison 10	2,523	40 (1.6)	<10 [6]	<10 [6]	
	Prison 11	3,470	43 (1.2)	<10 [6]	<10 [6]	
	Prison 12	815	<10 [6]	<10 [6]	<10 [6]	
	Prison 13	683	<10 [6]	<10 [6]	<10 [6]	
2018/19	Prison 1	1,333	17 (1.3)	<10 [6]	<10 [6]	
	Prison 2	2,705	31 (1.1)	<10 [6]	<10 [6]	
	Prison 3	2,522	38 (1.5)	<10 [6]	<10 [6]	
	Prison 4	2,349	42 (1.8)	<10 [6]	<10 [6]	
	Prison 5	676	<10 [6]	<10 [6]	<10 [6]	
	Prison 6	1,513	<10 [6]	<10 [6]	<10 [6]	
	Prison 7	654	17 (2.6)	<10 [6]	<10 [6]	
	Prison 8	1,148	28 (2.4)	<10 [6]	<10 [6]	
	Prison 9	996	17 (1.7)	<10 [6]	<10 [6]	
	Prison 10	2,717	39 (1.4)	<10 [6]	<10 [6]	
	Prison 11	4,020	53 (1.3)	<10 [6]	<10 [6]	
	Prison 12	792	<10 [6]	<10 [6]	<10 [6]	
	Prison 13	674	<10 [6]	<10 [6]	<10 [6]	
2019/20	Prison 1	1,410	20 (1.4)	<10 [6]	<10 [6]	
	Prison 2	2,979	42 (1.4)	<10 [6]	<10 [6]	
	Prison 3	2,809	59 (2.1)	<10 [6]	<10 [6]	
	Prison 4	2,651	58 (2.2)	<10 [6]	<10 [6]	
	Prison 5	616	<10 [6]	<10 [6]	<10 [6]	
	Prison 6	1,533	<10 [6]	<10 [6]	<10 [6]	
	Prison 7	860	25 (2.9)	<10 [6]	<10 [6]	
	Prison 8	1,385	34 (2.5)	<10 [6]	<10 [6]	
	Prison 9	1,092	21 (1.9)	<10 [6]	<10 [6]	
	Prison 10	3,577	51 (1.4)	<10 [6]	<10 [6]	
	Prison 11	5,348	85 (1.6)	<10 [6]	<10 [6]	
	Prison 12	805	<10 [6]	<10 [6]	<10 [6]	
	Prison 13	746	<10 [6]	<10 [6]	<10 [6]	
**Prison category**						
2017/18	A	1,664	25 (1.5)	<10 [6]	<10 [6]	
	B	9,254	123 (1.3)	<10 [6]	<10 [6]	
	C	6,035	84 (1.4)	<10 [6]	<10 [6]	
	Closed	1,720	40 (2.3)	<10 [6]	<10 [6]	
	D	2,189	11 (0.5)	<10 [6]	<10 [6]	
	YOI	815	<10 [6]	<10 [6]	<10 [6]	
2018/19	A	1,670	22 (1.3)	<10 [6]	<10 [6]	
	B	9,442	123 (1.3)	<10 [6]	<10 [6]	
	C	6,204	97 (1.6)	<10 [6]	<10 [6]	
	Closed	1,802	45 (2.5)	<10 [6]	<10 [6]	
	D	2,189	<10 [6]	<10 [6]	<10 [6]	
	YOI	792	<10 [6]	<10 [6]	<10 [6]	
2019/20	A	1,838	28 (1.5)	<10 [6]	<10 [6]	
	B	11,904	178 (1.5)	<10 [6]	<10 [6]	
	C	6,870	137 (2.0)	<10 [6]	<10 [6]	
	Closed	2,245	59 (2.6)	<10 [6]	<10 [6]	
	D	2,149	11 (0.5)	<10 [6]	<10 [6]	
	YOI	805	<10 [6]	<10 [6]	<10 [6]	

[1]Eligible for indicator; [2]Achieve in current prison; [3]Achieve in previous prison; [4]Overall achievement - either current or previous prison; [5]Declined indicator; [6]Suppressed (<10) to avoid disclosure

Indicator	*The proportion of people on drug treatment for epilepsy who have had an annual review and recorded as seizure free in the preceding 12 months*					***no comparable data in QOF 2019/20. Most recent QOF was 2013/14 which was 57.50%***
	**Group: Diabetes, Asthma & Epilepsy Care**					
	**Variable**	**Population**	**Eligible[1] (% popln)**	**Satisfy Annual Review[4] (% eligible)**	**Satisfy Seizure Free[4] (% eligible)**	
**Gender**						
2017/18	F	1,699	40 (2.4)	<10 [6]	<10 [6]	
	M	19,977	250 (1.3)	<10 [6]	<10 [6]	
2018/19	F	1,802	45 (2.5)	<10 [6]	<10 [6]	
	M	20,295	255 (1.3)	13 (5.1)	<10 [6]	
2019/20	F	1,376	34 (2.5)	<10 [6]	<10 [6]	
	M	23,570	360 (1.5)	<10 [6]	<10 [6]	
**Sentence Status**						
2017/18	.	81	<10 [6]	<10 [6]	<10 [6]	
	Absconded	<10 [6]	<10 [6]	<10 [6]	<10 [6]	
	Active In	15,872	227 (1.4)	<10 [6]	<10 [6]	
	Active Out	1,052	12 (1.1)	<10 [6]	<10 [6]	
	Convicted Sentence	2,125	29 (1.4)	<10 [6]	<10 [6]	
	Downgrade in security category	<10 [6]	<10 [6]	<10 [6]	<10 [6]	
	Internal Cell Move	<10 [6]	<10 [6]	<10 [6]	<10 [6]	
	Judges Remand	92	<10 [6]	<10 [6]	<10 [6]	
	Licence Revoke	52	<10 [6]	<10 [6]	<10 [6]	
	On Remand	1,492	16 (1.1)	<10 [6]	<10 [6]	
	Transfer	910	<10 [6]	<10 [6]	<10 [6]	
	Upgrade in security category	<10 [6]	<10 [6]	<10 [6]	<10 [6]	
2018/19	.	88	<10 [6]	<10 [6]	<10 [6]	
	Absconded	<10 [6]	<10 [6]	<10 [6]	<10 [6]	
	Active In	18,145	259 (1.4)	10 (3.9)	<10 [6]	
	Active Out	835	<10 [6]	<10 [6]	<10 [6]	
	Convicted Sentence	1,320	<10 [6]	<10 [6]	<10 [6]	
	Downgrade in security category	<10 [6]	<10 [6]	<10 [6]	<10 [6]	
	Internal Cell Move	<10 [6]	<10 [6]	<10 [6]	<10 [6]	
	Judges Remand	<10 [6]	<10 [6]	<10 [6]	<10 [6]	
	Licence Revoke	125	<10 [6]	<10 [6]	<10 [6]	
	On Remand	1,059	20 (1.9)	<10 [6]	<10 [6]	
	Transfer	518	<10 [6]	<10 [6]	<10 [6]	
	Upgrade in security category	<10 [6]	<10 [6]	<10 [6]	<10 [6]	
2019/20	.	69	<10 [6]	<10 [6]	<10 [6]	
	Absconded	<10 [6]	<10 [6]	<10 [6]	<10 [6]	
	Active In	22,424	372 (1.7)	<10 [6]	<10 [6]	
	Active Out	625	<10 [6]	<10 [6]	<10 [6]	
	Convicted Sentence	1,361	18 (1.3)	<10 [6]	<10 [6]	
	Downgrade in security category	<10 [6]	<10 [6]	<10 [6]	<10 [6]	
	Internal Cell Move	<10 [6]	<10 [6]	<10 [6]	<10 [6]	
	Judges Remand	19	<10 [6]	<10 [6]	<10 [6]	
	Licence Revoke	178	<10 [6]	<10 [6]	<10 [6]	
	On Remand	1,031	18 (1.7)	<10 [6]	<10 [6]	
	Transfer	101	<10 [6]	<10 [6]	<10 [6]	
	Upgrade in security category	<10 [6]	<10 [6]	<10 [6]	<10 [6]	
**Age - years**						
2017/18	10 - <20	468	<10 [6]	<10 [6]	<10 [6]	
	20 - <30	6,994	55 (0.8)	<10 [6]	<10 [6]	
	30 - <40	7,051	96 (1.4)	<10 [6]	<10 [6]	
	40 - <50	4,114	85 (2.1)	<10 [6]	<10 [6]	
	50 - <60	2,107	38 (1.8)	<10 [6]	<10 [6]	
	60 - <70	684	11 (1.6)	<10 [6]	<10 [6]	
	70 - <80	213	<10 [6]	<10 [6]	<10 [6]	
	80 - <90	40	<10 [6]	<10 [6]	<10 [6]	
	90 - <100	<10 [6]	<10 [6]	<10 [6]	<10 [6]	
	100 - <110	<10 [6]	<10 [6]	<10 [6]	<10 [6]	
2018/19	10 - <20	436	<10 [6]	<10 [6]	<10 [6]	
	20 - <30	7,163	72 (1.0)	<10 [6]	<10 [6]	
	30 - <40	7,381	104 (1.4)	<10 [6]	<10 [6]	
	40 - <50	4,180	72 (1.7)	<10 [6]	<10 [6]	
	50 - <60	1,978	30 (1.5)	<10 [6]	<10 [6]	
	60 - <70	701	18 (2.6)	<10 [6]	<10 [6]	
	70 - <80	209	<10 [6]	<10 [6]	<10 [6]	
	80 - <90	45	<10 [6]	<10 [6]	<10 [6]	
	90 - <100	<10 [6]	<10 [6]	<10 [6]	<10 [6]	
	100 - <110	<10 [6]	<10 [6]	<10 [6]	<10 [6]	

[1]Eligible for indicator; [2]Achieve in current prison; [3]Achieve in previous prison; [4]Overall achievement - either current or previous prison; [5]Declined indicator; [6]Suppressed (<10) to avoid disclosure

Indicator	*The proportion of people on drug treatment for epilepsy who have had an annual review and recorded as seizure free in the preceding 12 months*					***no comparable data in QOF 2019/20. Most recent QOF was 2013/14 which was 57.50%***
	**Group: Diabetes, Asthma & Epilepsy Care**					
	**Variable**	**Population**	**Eligible[1] (% popln)**	**Satisfy Annual Review[4] (% eligible)**	**Satisfy Seizure Free[4] (% eligible)**	
2019/20	10 - <20	404	<10 [6]	<10 [6]	<10 [6]	
	20 - <30	8,064	85 (1.1)	<10 [6]	<10 [6]	
	30 - <40	9,125	151 (1.7)	<10 [6]	<10 [6]	
	40 - <50	4,948	131 (2.6)	<10 [6]	<10 [6]	
	50 - <60	2,224	32 (1.4)	<10 [6]	<10 [6]	
	60 - <70	751	17 (2.3)	<10 [6]	<10 [6]	
	70 - <80	238	<10 [6]	<10 [6]	<10 [6]	
	80 - <90	53	<10 [6]	<10 [6]	<10 [6]	
	90 - <100	<10 [6]	<10 [6]	<10 [6]	<10 [6]	
	100 - <110	<10 [6]	<10 [6]	<10 [6]	<10 [6]	
**Length of Stay (months)**						
2017/18	<1	4,474	55 (1.2)	<10 [6]	<10 [6]	
	1-<6	8,075	104 (1.3)	<10 [6]	<10 [6]	
	6-<12	3,672	51 (1.4)	<10 [6]	<10 [6]	
	12-<24	2,832	36 (1.3)	<10 [6]	<10 [6]	
	24+	2,624	44 (1.7)	<10 [6]	<10 [6]	
2017/18	<1	4,801	56 (1.2)	<10 [6]	<10 [6]	
	1-<6	7,742	87 (1.1)	<10 [6]	<10 [6]	
	6-<12	3,616	63 (1.7)	<10 [6]	<10 [6]	
	12-<24	3,752	62 (1.7)	<10 [6]	<10 [6]	
	24+	2,188	32 (1.5)	<10 [6]	<10 [6]	
2018/19	<1	5,745	85 (1.5)	<10 [6]	<10 [6]	
	1-<6	9,697	138 (1.4)	<10 [6]	<10 [6]	
	6-<12	5,090	97 (1.9)	<10 [6]	<10 [6]	
	12-<24	3,244	63 (1.9)	<10 [6]	<10 [6]	
	24+	2,035	36 (1.8)	<10 [6]	<10 [6]	
**Ethnic Group**						
2017/18	White	15,638	243 (1.6)	<10 [6]	<10 [6]	
	Mixed	431	<10 [6]	<10 [6]	<10 [6]	
	Asian or Asian British	813	<10 [6]	<10 [6]	<10 [6]	
	Black or Black British	404	<10 [6]	<10 [6]	<10 [6]	
	Chinese and Other	214	<10 [6]	<10 [6]	<10 [6]	
	Unclassified	372	<10 [6]	<10 [6]	<10 [6]	
2018/19	White	14,911	242 (1.6)	12 (5.0)	<10 [6]	
	Mixed	371	<10 [6]	<10 [6]	<10 [6]	
	Asian or Asian British	726	<10 [6]	<10 [6]	<10 [6]	
	Black or Black British	364	<10 [6]	<10 [6]	<10 [6]	
	Chinese and Other	167	<10 [6]	<10 [6]	<10 [6]	
	Unclassified	409	<10 [6]	<10 [6]	<10 [6]	
2019/20	White	16,606	325 (2.0)	<10 [6]	<10 [6]	
	Mixed	409	<10 [6]	<10 [6]	<10 [6]	
	Asian or Asian British	755	<10 [6]	<10 [6]	<10 [6]	
	Black or Black British	451	<10 [6]	<10 [6]	<10 [6]	
	Chinese and Other	163	<10 [6]	<10 [6]	<10 [6]	
	Unclassified	387	<10 [6]	<10 [6]	<10 [6]	

[1]Eligible for indicator; [2]Achieve in current prison; [3]Achieve in previous prison; [4]Overall achievement - either current or previous prison; [5]Declined indicator; [6]Suppressed (<10) to avoid disclosure

Indicator	*The proportion of people with transient ischaemic attack or stroke, excluding those with haemorrhagic stroke, who have been prescribed an antiplatelet or anticoagulant in the preceding 12 months*	*Community achievement 2019/20: 91.01% (QOF 19/20)*
	**Group: Cardiovascular Disease**	

	**Variable**	**Population**	**Eligible[1] (% popln)**	**Satisfy[2] (% eligible)**	**Elsewhere[3] (% eligible)**	**Achieve[4] (% eligible)**
**Year**						
	2017/18	21,677	72 (0.3)	50 (69.4)	<10 [6]	51 (70.8)
	2018/19	22,099	73 (0.3)	49 (67.1)	<10 [6]	50 (68.5)
	2019/20	25,811	86 (0.3)	51 (59.3)	<10 [6]	51 (59.3)
**Prison**						
2017/18	Prison 1	1,323	<10 [6]	<10 [6]	<10 [6]	<10 [6]
	Prison 2	3,261	<10 [6]	<10 [6]	<10 [6]	<10 [6]
	Prison 3	2,623	13 (0.5)	10 (76.9)	<10 [6]	10 (76.9)
	Prison 4	2,089	11 (0.5)	<10 [6]	<10 [6]	10 (90.9)
	Prison 5	637	<10 [6]	<10 [6]	<10 [6]	<10 [6]
	Prison 6	1,552	<10 [6]	<10 [6]	<10 [6]	<10 [6]
	Prison 7	635	<10 [6]	<10 [6]	<10 [6]	<10 [6]
	Prison 8	1,085	<10 [6]	<10 [6]	<10 [6]	<10 [6]
	Prison 9	981	<10 [6]	<10 [6]	<10 [6]	<10 [6]
	Prison 10	2,523	<10 [6]	<10 [6]	<10 [6]	<10 [6]
	Prison 11	3,470	<10 [6]	<10 [6]	<10 [6]	<10 [6]
	Prison 12	815	<10 [6]	<10 [6]	<10 [6]	<10 [6]
	Prison 13	683	<10 [6]	<10 [6]	<10 [6]	<10 [6]
2018/19	Prison 1	1,333	<10 [6]	<10 [6]	<10 [6]	<10 [6]
	Prison 2	2,705	<10 [6]	<10 [6]	<10 [6]	<10 [6]
	Prison 3	2,522	<10 [6]	<10 [6]	<10 [6]	<10 [6]
	Prison 4	2,349	14 (0.6)	11 (78.6)	<10 [6]	11 (78.6)
	Prison 5	676	<10 [6]	<10 [6]	<10 [6]	<10 [6]
	Prison 6	1,513	<10 [6]	<10 [6]	<10 [6]	<10 [6]
	Prison 7	654	<10 [6]	<10 [6]	<10 [6]	<10 [6]
	Prison 8	1,148	<10 [6]	<10 [6]	<10 [6]	<10 [6]
	Prison 9	996	13 (1.3)	<10 [6]	<10 [6]	<10 [6]
	Prison 10	2,717	<10 [6]	<10 [6]	<10 [6]	<10 [6]
	Prison 11	4,020	<10 [6]	<10 [6]	<10 [6]	<10 [6]
	Prison 12	792	<10 [6]	<10 [6]	<10 [6]	<10 [6]
	Prison 13	674	<10 [6]	<10 [6]	<10 [6]	<10 [6]
2019/20	Prison 1	1,410	<10 [6]	<10 [6]	<10 [6]	<10 [6]
	Prison 2	2,979	<10 [6]	<10 [6]	<10 [6]	<10 [6]
	Prison 3	2,809	10 (0.4)	<10 [6]	<10 [6]	<10 [6]
	Prison 4	2,651	19 (0.7)	15 (78.9)	<10 [6]	15 (78.9)
	Prison 5	616	<10 [6]	<10 [6]	<10 [6]	<10 [6]
	Prison 6	1,533	<10 [6]	<10 [6]	<10 [6]	<10 [6]
	Prison 7	860	<10 [6]	<10 [6]	<10 [6]	<10 [6]
	Prison 8	1,385	<10 [6]	<10 [6]	<10 [6]	<10 [6]
	Prison 9	1,092	21 (1.9)	<10 [6]	<10 [6]	<10 [6]
	Prison 10	3,577	<10 [6]	<10 [6]	<10 [6]	<10 [6]
	Prison 11	5,348	<10 [6]	<10 [6]	<10 [6]	<10 [6]
	Prison 12	805	<10 [6]	<10 [6]	<10 [6]	<10 [6]
	Prison 13	746	<10 [6]	<10 [6]	<10 [6]	<10 [6]
**Prison category**						
2017/18	A	1,664	15 (0.9)	<10 [6]	<10 [6]	<10 [6]
	B	9,254	16 (0.2)	12 (75.0)	<10 [6]	12 (75.0)
	C	6,035	28 (0.5)	22 (78.6)	<10 [6]	23 (82.1)
	Closed	1,720	<10 [6]	<10 [6]	<10 [6]	<10 [6]
	D	2,189	10 (0.5)	<10 [6]	<10 [6]	<10 [6]
	YOI	815	<10 [6]	<10 [6]	<10 [6]	<10 [6]
2018/19	A	1,670	22 (1.3)	<10 [6]	<10 [6]	<10 [6]
	B	9,442	15 (0.2)	13 (86.7)	<10 [6]	14 (93.3)
	C	6,204	24 (0.4)	19 (79.2)	<10 [6]	19 (79.2)
	Closed	1,802	<10 [6]	<10 [6]	<10 [6]	<10 [6]
	D	2,189	<10 [6]	<10 [6]	<10 [6]	<10 [6]
	YOI	792	<10 [6]	<10 [6]	<10 [6]	<10 [6]
2019/20	A	1,838	30 (1.6)	<10 [6]	<10 [6]	<10 [6]
	B	11,904	13 (0.1)	12 (92.3)	<10 [6]	12 (92.3)
	C	6,870	33 (0.5)	24 (72.7)	<10 [6]	24 (72.7)
	Closed	2,245	<10 [6]	<10 [6]	<10 [6]	<10 [6]
	D	2,149	<10 [6]	<10 [6]	<10 [6]	<10 [6]
	YOI	805	<10 [6]	<10 [6]	<10 [6]	<10 [6]
**Gender**						
2017/18	F	1,699	<10 [6]	<10 [6]	<10 [6]	<10 [6]
	M	19,977	69 (0.3)	49 (71.0)	<10 [6]	50 (72.5)
2018/19	F	1,802	<10 [6]	<10 [6]	<10 [6]	<10 [6]
	M	20,295	69 (0.3)	46 (66.7)	<10 [6]	47 (68.1)
2019/20	F	1,376	<10 [6]	<10 [6]	<10 [6]	<10 [6]
	M	23,570	81 (0.3)	48 (59.3)	<10 [6]	48 (59.3)

[1]Eligible for indicator; [2]Achieve in current prison; [3]Achieve in previous prison; [4]Overall achievement - either current or previous prison; [5]Declined indicator; [6]Suppressed (<10) to avoid disclosure

Indicator *The proportion of people with transient ischaemic attack or stroke, excluding those with haemorrhagic stroke, who have been prescribed an antiplatelet or anticoagulant in the preceding 12 months*

Group: Cardiovascular Disease

Community achievement 2019/20: 91.01% (QOF 19/20)

	Variable	Population	Eligible[1] (% popln)	Satisfy[2] (% eligible)	Elsewhere[3] (% eligible)	Achieve[4] (% eligible)
**Sentence Status**						
2017/18	.	81	<10 [6]	<10 [6]	<10 [6]	<10 [6]
	Absconded	<10 [6]	<10 [6]	<10 [6]	<10 [6]	<10 [6]
	Active In	15,872	57 (0.4)	39 (68.4)	<10 [6]	40 (70.2)
	Active Out	1,052	<10 [6]	<10 [6]	<10 [6]	<10 [6]
	Convicted Sentence	2,125	<10 [6]	<10 [6]	<10 [6]	<10 [6]
	Downgrade in security category	<10 [6]	<10 [6]	<10 [6]	<10 [6]	<10 [6]
	Internal Cell Move	<10 [6]	<10 [6]	<10 [6]	<10 [6]	<10 [6]
	Judges Remand	92	<10 [6]	<10 [6]	<10 [6]	<10 [6]
	Licence Revoke	52	<10 [6]	<10 [6]	<10 [6]	<10 [6]
	On Remand	1,492	<10 [6]	<10 [6]	<10 [6]	<10 [6]
	Transfer	910	<10 [6]	<10 [6]	<10 [6]	<10 [6]
	Upgrade in security category	<10 [6]	<10 [6]	<10 [6]	<10 [6]	<10 [6]
2018/19	.	88	<10 [6]	<10 [6]	<10 [6]	<10 [6]
	Absconded	<10 [6]	<10 [6]	<10 [6]	<10 [6]	<10 [6]
	Active In	18,145	66 (0.4)	43 (65.2)	<10 [6]	44 (66.7)
	Active Out	835	<10 [6]	<10 [6]	<10 [6]	<10 [6]
	Convicted Sentence	1,320	<10 [6]	<10 [6]	<10 [6]	<10 [6]
	Downgrade in security category	<10 [6]	<10 [6]	<10 [6]	<10 [6]	<10 [6]
	Internal Cell Move	<10 [6]	<10 [6]	<10 [6]	<10 [6]	<10 [6]
	Judges Remand	<10 [6]	<10 [6]	<10 [6]	<10 [6]	<10 [6]
	Licence Revoke	125	<10 [6]	<10 [6]	<10 [6]	<10 [6]
	On Remand	1,059	<10 [6]	<10 [6]	<10 [6]	<10 [6]
	Transfer	518	<10 [6]	<10 [6]	<10 [6]	<10 [6]
	Upgrade in security category	<10 [6]	<10 [6]	<10 [6]	<10 [6]	<10 [6]
2019/20	.	69	<10 [6]	<10 [6]	<10 [6]	<10 [6]
	Absconded	<10 [6]	<10 [6]	<10 [6]	<10 [6]	<10 [6]
	Active In	22,424	83 (0.4)	49 (59.0)	<10 [6]	49 (59.0)
	Active Out	625	<10 [6]	<10 [6]	<10 [6]	<10 [6]
	Convicted Sentence	1,361	<10 [6]	<10 [6]	<10 [6]	<10 [6]
	Downgrade in security category	<10 [6]	<10 [6]	<10 [6]	<10 [6]	<10 [6]
	Internal Cell Move	<10 [6]	<10 [6]	<10 [6]	<10 [6]	<10 [6]
	Judges Remand	19	<10 [6]	<10 [6]	<10 [6]	<10 [6]
	Licence Revoke	178	<10 [6]	<10 [6]	<10 [6]	<10 [6]
	On Remand	1,031	<10 [6]	<10 [6]	<10 [6]	<10 [6]
	Transfer	101	<10 [6]	<10 [6]	<10 [6]	<10 [6]
	Upgrade in security category	<10 [6]	<10 [6]	<10 [6]	<10 [6]	<10 [6]
**Age - years**						
2017/18	10 - <20	468	<10 [6]	<10 [6]	<10 [6]	<10 [6]
	20 - <30	6,994	<10 [6]	<10 [6]	<10 [6]	<10 [6]
	30 - <40	7,051	<10 [6]	<10 [6]	<10 [6]	<10 [6]
	40 - <50	4,114	<10 [6]	<10 [6]	<10 [6]	<10 [6]
	50 - <60	2,107	18 (0.9)	12 (66.7)	<10 [6]	12 (66.7)
	60 - <70	684	25 (3.7)	17 (68.0)	<10 [6]	17 (68.0)
	70 - <80	213	16 (7.5)	15 (93.8)	<10 [6]	15 (93.8)
	80 - <90	40	<10 [6]	<10 [6]	<10 [6]	<10 [6]
	90 - <100	<10 [6]	<10 [6]	<10 [6]	<10 [6]	<10 [6]
	100 - <110	<10 [6]	<10 [6]	<10 [6]	<10 [6]	<10 [6]
2018/19	10 - <20	436	<10 [6]	<10 [6]	<10 [6]	<10 [6]
	20 - <30	7,163	<10 [6]	<10 [6]	<10 [6]	<10 [6]
	30 - <40	7,381	<10 [6]	<10 [6]	<10 [6]	<10 [6]
	40 - <50	4,180	<10 [6]	<10 [6]	<10 [6]	<10 [6]
	50 - <60	1,978	22 (1.1)	10 (45.5)	<10 [6]	11 (50.0)
	60 - <70	701	24 (3.4)	19 (79.2)	<10 [6]	19 (79.2)
	70 - <80	209	15 (7.2)	<10 [6]	<10 [6]	<10 [6]
	80 - <90	45	<10 [6]	<10 [6]	<10 [6]	<10 [6]
	90 - <100	<10 [6]	<10 [6]	<10 [6]	<10 [6]	<10 [6]
	100 - <110	<10 [6]	<10 [6]	<10 [6]	<10 [6]	<10 [6]
2019/20	10 - <20	404	<10 [6]	<10 [6]	<10 [6]	<10 [6]
	20 - <30	8,064	<10 [6]	<10 [6]	<10 [6]	<10 [6]
	30 - <40	9,125	<10 [6]	<10 [6]	<10 [6]	<10 [6]
	40 - <50	4,948	<10 [6]	<10 [6]	<10 [6]	<10 [6]
	50 - <60	2,224	23 (1.0)	11 (47.8)	<10 [6]	11 (47.8)
	60 - <70	751	27 (3.6)	19 (70.4)	<10 [6]	19 (70.4)
	70 - <80	238	16 (6.7)	10 (62.5)	<10 [6]	10 (62.5)
	80 - <90	53	<10 [6]	<10 [6]	<10 [6]	<10 [6]
	90 - <100	<10 [6]	<10 [6]	<10 [6]	<10 [6]	<10 [6]
	100 - <110	<10 [6]	<10 [6]	<10 [6]	<10 [6]	<10 [6]

[1]Eligible for indicator; [2]Achieve in current prison; [3]Achieve in previous prison; [4]Overall achievement - either current or previous prison; [5]Declined indicator; [6]Suppressed (<10) to avoid disclosure

Indicator	*The proportion of people with transient ischaemic attack or stroke, excluding those with haemorrhagic stroke, who have been prescribed an antiplatelet or anticoagulant in the preceding 12 months*	*Community achievement 2019/20: 91.01% (QOF 19/20)*
	**Group: Cardiovascular Disease**	

	**Variable**	**Population**	**Eligible[1] (% popln)**	**Satisfy[2] (% eligible)**	**Elsewhere[3] (% eligible)**	**Achieve[4] (% eligible)**
**Length of Stay (months)**						
2017/18	<1	4,474	<10 [6]	<10 [6]	<10 [6]	<10 [6]
	1-<6	8,075	13 (0.2)	<10 [6]	<10 [6]	<10 [6]
	6-<12	3,672	11 (0.3)	11 (100.0)	<10 [6]	11 (100.0)
	12-<24	2,832	12 (0.4)	12 (100.0)	<10 [6]	12 (100.0)
	24+	2,624	29 (1.1)	14 (48.3)	<10 [6]	15 (51.7)
2018/19	<1	4,801	<10 [6]	<10 [6]	<10 [6]	<10 [6]
	1-<6	7,742	11 (0.1)	10 (90.9)	<10 [6]	10 (90.9)
	6-<12	3,616	<10 [6]	<10 [6]	<10 [6]	<10 [6]
	12-<24	3,447	15 (0.4)	11 (73.3)	<10 [6]	11 (73.3)
	24+	2,493	38 (1.5)	21 (55.3)	<10 [6]	21 (55.3)
2019/20	<1	5,745	<10 [6]	<10 [6]	<10 [6]	<10 [6]
	1-<6	9,697	17 (0.2)	10 (58.8)	<10 [6]	10 (58.8)
	6-<12	5,090	10 (0.2)	<10 [6]	<10 [6]	<10 [6]
	12-<24	3,244	15 (0.5)	11 (73.3)	<10 [6]	11 (73.3)
	24+	2,035	42 (2.1)	20 (47.6)	<10 [6]	20 (47.6)
**Ethnic Group**						
2017/18	White	15,638	62 (0.4)	43 (69.4)	<10 [6]	44 (71.0)
	Mixed	431	<10 [6]	<10 [6]	<10 [6]	<10 [6]
	Asian or Asian British	813	<10 [6]	<10 [6]	<10 [6]	<10 [6]
	Black or Black British	404	<10 [6]	<10 [6]	<10 [6]	<10 [6]
	Chinese and Other	214	<10 [6]	<10 [6]	<10 [6]	<10 [6]
	Unclassified	372	<10 [6]	<10 [6]	<10 [6]	<10 [6]
2018/19	White	14,911	69 (0.5)	46 (66.7)	<10 [6]	47 (68.1)
	Mixed	371	<10 [6]	<10 [6]	<10 [6]	<10 [6]
	Asian or Asian British	726	<10 [6]	<10 [6]	<10 [6]	<10 [6]
	Black or Black British	364	<10 [6]	<10 [6]	<10 [6]	<10 [6]
	Chinese and Other	167	<10 [6]	<10 [6]	<10 [6]	<10 [6]
	Unclassified	409	<10 [6]	<10 [6]	<10 [6]	<10 [6]
2019/20	White	16,606	76 (0.5)	46 (60.5)	<10 [6]	46 (60.5)
	Mixed	409	<10 [6]	<10 [6]	<10 [6]	<10 [6]
	Asian or Asian British	755	<10 [6]	<10 [6]	<10 [6]	<10 [6]
	Black or Black British	451	<10 [6]	<10 [6]	<10 [6]	<10 [6]
	Chinese and Other	163	<10 [6]	<10 [6]	<10 [6]	<10 [6]
	Unclassified	387	<10 [6]	<10 [6]	<10 [6]	<10 [6]

[1]Eligible for indicator; [2]Achieve in current prison; [3]Achieve in previous prison; [4]Overall achievement - either current or previous prison; [5]Declined indicator; [6]Suppressed (<10) to avoid disclosure

Indicator	The proportion of people with persistent or paroxysmal atrial fibrillation and a CHA2DS2-VASc score equal to two or more, with a prescription for warfarin or a direct-acting oral anticoagulant in the preceding 12 months					Community achievement 2019/20: 87.26% (QOF 19/20)
	Group: Cardiovascular Disease					
	**Variable**	**Population**	**Eligible[1] (% popln)**	**Satisfy[2] (% eligible)**	**Elsewhere[3] (% eligible)**	**Achieve[4] (% eligible)**
**Year**						
	2017/18	21,677	40 (0.2)	33 (82.5)	<10 [6]	34 (85.0)
	2018/19	22,099	53 (0.2)	41 (77.4)	<10 [6]	41 (77.4)
	2019/20	25,811	54 (0.2)	45 (83.3)	<10 [6]	45 (83.3)
**Prison**						
2017/18	Prison 1	1,323	<10 [6]	<10 [6]	<10 [6]	<10 [6]
	Prison 2	3,261	<10 [6]	<10 [6]	<10 [6]	<10 [6]
	Prison 3	2,623	<10 [6]	<10 [6]	<10 [6]	<10 [6]
	Prison 4	2,089	<10 [6]	<10 [6]	<10 [6]	<10 [6]
	Prison 5	637	<10 [6]	<10 [6]	<10 [6]	<10 [6]
	Prison 6	1,552	<10 [6]	<10 [6]	<10 [6]	<10 [6]
	Prison 7	635	<10 [6]	<10 [6]	<10 [6]	<10 [6]
	Prison 8	1,085	<10 [6]	<10 [6]	<10 [6]	<10 [6]
	Prison 9	981	<10 [6]	<10 [6]	<10 [6]	<10 [6]
	Prison 10	2,523	<10 [6]	<10 [6]	<10 [6]	<10 [6]
	Prison 11	3,470	<10 [6]	<10 [6]	<10 [6]	<10 [6]
	Prison 12	815	<10 [6]	<10 [6]	<10 [6]	<10 [6]
	Prison 13	683	<10 [6]	<10 [6]	<10 [6]	<10 [6]
2018/19	Prison 1	1,333	<10 [6]	<10 [6]	<10 [6]	<10 [6]
	Prison 2	2,705	<10 [6]	<10 [6]	<10 [6]	<10 [6]
	Prison 3	2,522	<10 [6]	<10 [6]	<10 [6]	<10 [6]
	Prison 4	2,349	11 (0.5)	<10 [6]	<10 [6]	<10 [6]
	Prison 5	676	<10 [6]	<10 [6]	<10 [6]	<10 [6]
	Prison 6	1,513	<10 [6]	<10 [6]	<10 [6]	<10 [6]
	Prison 7	654	<10 [6]	<10 [6]	<10 [6]	<10 [6]
	Prison 8	1,148	<10 [6]	<10 [6]	<10 [6]	<10 [6]
	Prison 9	996	12 (1.2)	<10 [6]	<10 [6]	<10 [6]
	Prison 10	2,717	<10 [6]	<10 [6]	<10 [6]	<10 [6]
	Prison 11	4,020	<10 [6]	<10 [6]	<10 [6]	<10 [6]
	Prison 12	792	<10 [6]	<10 [6]	<10 [6]	<10 [6]
	Prison 13	674	<10 [6]	<10 [6]	<10 [6]	<10 [6]
2019/20	Prison 1	1,410	<10 [6]	<10 [6]	<10 [6]	<10 [6]
	Prison 2	2,979	<10 [6]	<10 [6]	<10 [6]	<10 [6]
	Prison 3	2,809	<10 [6]	<10 [6]	<10 [6]	<10 [6]
	Prison 4	2,651	10 (0.4)	<10 [6]	<10 [6]	<10 [6]
	Prison 5	616	<10 [6]	<10 [6]	<10 [6]	<10 [6]
	Prison 6	1,533	<10 [6]	<10 [6]	<10 [6]	<10 [6]
	Prison 7	860	<10 [6]	<10 [6]	<10 [6]	<10 [6]
	Prison 8	1,385	<10 [6]	<10 [6]	<10 [6]	<10 [6]
	Prison 9	1,092	16 (1.5)	13 (81.3)	<10 [6]	13 (81.3)
	Prison 10	3,577	<10 [6]	<10 [6]	<10 [6]	<10 [6]
	Prison 11	5,348	<10 [6]	<10 [6]	<10 [6]	<10 [6]
	Prison 12	805	<10 [6]	<10 [6]	<10 [6]	<10 [6]
	Prison 13	746	<10 [6]	<10 [6]	<10 [6]	<10 [6]
**Prison category**						
2017/18	A	1,664	<10 [6]	<10 [6]	<10 [6]	<10 [6]
	B	9,254	10 (0.1)	<10 [6]	<10 [6]	<10 [6]
	C	6,035	12 (0.2)	11 (91.7)	<10 [6]	11 (91.7)
	Closed	1,720	<10 [6]	<10 [6]	<10 [6]	<10 [6]
	D	2,189	<10 [6]	<10 [6]	<10 [6]	<10 [6]
	YOI	815	<10 [6]	<10 [6]	<10 [6]	<10 [6]
2018/19	A	1,670	19 (1.1)	14 (73.7)	<10 [6]	14 (73.7)
	B	9,442	11 (0.1)	<10 [6]	<10 [6]	<10 [6]
	C	6,204	16 (0.3)	13 (81.3)	<10 [6]	13 (81.3)
	Closed	1,802	<10 [6]	<10 [6]	<10 [6]	<10 [6]
	D	2,189	<10 [6]	<10 [6]	<10 [6]	<10 [6]
	YOI	792	<10 [6]	<10 [6]	<10 [6]	<10 [6]
2019/20	A	1,838	22 (1.2)	18 (81.8)	<10 [6]	18 (81.8)
	B	11,904	12 (0.1)	<10 [6]	<10 [6]	<10 [6]
	C	6,870	15 (0.2)	14 (93.3)	<10 [6]	14 (93.3)
	Closed	2,245	<10 [6]	<10 [6]	<10 [6]	<10 [6]
	D	2,149	<10 [6]	<10 [6]	<10 [6]	<10 [6]
	YOI	805	<10 [6]	<10 [6]	<10 [6]	<10 [6]
**Gender**						
2017/18	F	1,699	<10 [6]	<10 [6]	<10 [6]	<10 [6]
	M	19,977	37 (0.2)	31 (83.8)	<10 [6]	31 (83.8)
2018/19	F	1,802	<10 [6]	<10 [6]	<10 [6]	<10 [6]
	M	20,295	51 (0.3)	40 (78.4)	<10 [6]	40 (78.4)
2019/20	F	1,376	<10 [6]	<10 [6]	<10 [6]	<10 [6]
	M	23,570	52 (0.2)	44 (84.6)	<10 [6]	44 (84.6)

[1]Eligible for indicator; [2]Achieve in current prison; [3]Achieve in previous prison; [4]Overall achievement - either current or previous prison; [5]Declined indicator; [6]Suppressed (<10) to avoid disclosure

Indicator ***The proportion of people with persistent or paroxysmal atrial fibrillation and a CHA2DS2-VASc score equal to two or more, with a prescription for warfarin or a direct-acting oral anticoagulant in the preceding 12 months***

Community achievement 2019/20: 87.26% (QOF 19/20)

Group: Cardiovascular Disease

	**Variable**	**Population**	**Eligible[1] (% popln)**	**Satisfy[2] (% eligible)**	**Elsewhere[3] (% eligible)**	**Achieve[4] (% eligible)**
**Sentence Status**						
2017/18	.	81	<10 [6]	<10 [6]	<10 [6]	<10 [6]
	Absconded	<10 [6]	<10 [6]	<10 [6]	<10 [6]	<10 [6]
	Active In	15,872	32 (0.2)	26 (81.3)	<10 [6]	27 (84.4)
	Active Out	1,052	<10 [6]	<10 [6]	<10 [6]	<10 [6]
	Convicted Sentence	2,125	<10 [6]	<10 [6]	<10 [6]	<10 [6]
	Downgrade in security category	<10 [6]	<10 [6]	<10 [6]	<10 [6]	<10 [6]
	Internal Cell Move	<10 [6]	<10 [6]	<10 [6]	<10 [6]	<10 [6]
	Judges Remand	92	<10 [6]	<10 [6]	<10 [6]	<10 [6]
	Licence Revoke	52	<10 [6]	<10 [6]	<10 [6]	<10 [6]
	On Remand	1,492	<10 [6]	<10 [6]	<10 [6]	<10 [6]
	Transfer	910	<10 [6]	<10 [6]	<10 [6]	<10 [6]
	Upgrade in security category	<10 [6]	<10 [6]	<10 [6]	<10 [6]	<10 [6]
2018/19	.	88	<10 [6]	<10 [6]	<10 [6]	<10 [6]
	Absconded	<10 [6]	<10 [6]	<10 [6]	<10 [6]	<10 [6]
	Active In	18,145	45 (0.2)	36 (80.0)	<10 [6]	36 (80.0)
	Active Out	835	<10 [6]	<10 [6]	<10 [6]	<10 [6]
	Convicted Sentence	1,320	<10 [6]	<10 [6]	<10 [6]	<10 [6]
	Downgrade in security category	<10 [6]	<10 [6]	<10 [6]	<10 [6]	<10 [6]
	Internal Cell Move	<10 [6]	<10 [6]	<10 [6]	<10 [6]	<10 [6]
	Judges Remand	<10 [6]	<10 [6]	<10 [6]	<10 [6]	<10 [6]
	Licence Revoke	125	<10 [6]	<10 [6]	<10 [6]	<10 [6]
	On Remand	1,059	<10 [6]	<10 [6]	<10 [6]	<10 [6]
	Transfer	518	<10 [6]	<10 [6]	<10 [6]	<10 [6]
	Upgrade in security category	<10 [6]	<10 [6]	<10 [6]	<10 [6]	<10 [6]
2019/20	.	69	<10 [6]	<10 [6]	<10 [6]	<10 [6]
	Absconded	<10 [6]	<10 [6]	<10 [6]	<10 [6]	<10 [6]
	Active In	22,424	46 (0.2)	40 (87.0)	<10 [6]	40 (87.0)
	Active Out	625	<10 [6]	<10 [6]	<10 [6]	<10 [6]
	Convicted Sentence	1,361	<10 [6]	<10 [6]	<10 [6]	<10 [6]
	Downgrade in security category	<10 [6]	<10 [6]	<10 [6]	<10 [6]	<10 [6]
	Internal Cell Move	<10 [6]	<10 [6]	<10 [6]	<10 [6]	<10 [6]
	Judges Remand	19	<10 [6]	<10 [6]	<10 [6]	<10 [6]
	Licence Revoke	178	<10 [6]	<10 [6]	<10 [6]	<10 [6]
	On Remand	1,031	<10 [6]	<10 [6]	<10 [6]	<10 [6]
	Transfer	101	<10 [6]	<10 [6]	<10 [6]	<10 [6]
	Upgrade in security category	<10 [6]	<10 [6]	<10 [6]	<10 [6]	<10 [6]
**Age - years**						
2017/18	10 - <20	468	<10 [6]	<10 [6]	<10 [6]	<10 [6]
	20 - <30	6,994	<10 [6]	<10 [6]	<10 [6]	<10 [6]
	30 - <40	7,051	<10 [6]	<10 [6]	<10 [6]	<10 [6]
	40 - <50	4,114	<10 [6]	<10 [6]	<10 [6]	<10 [6]
	50 - <60	2,107	11 (0.5)	8 (72.7)	<10 [6]	<10 [6]
	60 - <70	684	13 (1.9)	11 (84.6)	<10 [6]	11 (84.6)
	70 - <80	213	11 (5.2)	10 (90.9)	<10 [6]	10 (90.9)
	80 - <90	40	<10 [6]	<10 [6]	<10 [6]	<10 [6]
	90 - <100	<10 [6]	<10 [6]	<10 [6]	<10 [6]	<10 [6]
	100 - <110	<10 [6]	<10 [6]	<10 [6]	<10 [6]	<10 [6]
2018/19	10 - <20	436	<10 [6]	<10 [6]	<10 [6]	<10 [6]
	20 - <30	7,163	<10 [6]	<10 [6]	<10 [6]	<10 [6]
	30 - <40	7,381	<10 [6]	<10 [6]	<10 [6]	<10 [6]
	40 - <50	4,180	<10 [6]	<10 [6]	<10 [6]	<10 [6]
	50 - <60	1,978	10 (0.5)	<10 [6]	<10 [6]	<10 [6]
	60 - <70	701	21 (3.0)	17 (81.0)	<10 [6]	17 (81.0)
	70 - <80	209	13 (6.2)	11 (84.6)	<10 [6]	11 (84.6)
	80 - <90	45	<10 [6]	<10 [6]	<10 [6]	<10 [6]
	90 - <100	<10 [6]	<10 [6]	<10 [6]	<10 [6]	<10 [6]
	100 - <110	<10 [6]	<10 [6]	<10 [6]	<10 [6]	<10 [6]
2019/20	10 - <20	404	<10 [6]	<10 [6]	<10 [6]	<10 [6]
	20 - <30	8,064	<10 [6]	<10 [6]	<10 [6]	<10 [6]
	30 - <40	9,125	<10 [6]	<10 [6]	<10 [6]	<10 [6]
	40 - <50	4,948	<10 [6]	<10 [6]	<10 [6]	<10 [6]
	50 - <60	2,224	<10 [6]	<10 [6]	<10 [6]	<10 [6]
	60 - <70	751	22 (2.9)	19 (86.4)	<10 [6]	19 (86.4)
	70 - <80	238	15 (6.3)	12 (80.0)	<10 [6]	12 (80.0)
	80 - <90	53	<10 [6]	<10 [6]	<10 [6]	<10 [6]
	90 - <100	<10 [6]	<10 [6]	<10 [6]	<10 [6]	<10 [6]
	100 - <110	<10 [6]	<10 [6]	<10 [6]	<10 [6]	<10 [6]

[1]Eligible for indicator; [2]Achieve in current prison; [3]Achieve in previous prison; [4]Overall achievement - either current or previous prison; [5]Declined indicator; [6]Suppressed (<10) to avoid disclosure

Indicator *The proportion of people with persistent or paroxysmal atrial fibrillation and a CHA2DS2-VASc score equal to two or more, with a prescription for warfarin or a direct-acting oral anticoagulant in the preceding 12 months*

Community achievement 2019/20: 87.26% (QOF 19/20)

Group: Cardiovascular Disease

	Variable	Population	Eligible[1] (% popln)	Satisfy[2] (% eligible)	Elsewhere[3] (% eligible)	Achieve[4] (% eligible)
**Length of Stay (months)**						
2017/18	<1	4,474	<10 [6]	<10 [6]	<10 [6]	<10 [6]
	1-<6	8,075	<10 [6]	<10 [6]	<10 [6]	<10 [6]
	6-<12	3,672	<10 [6]	<10 [6]	<10 [6]	<10 [6]
	12-<24	2,832	<10 [6]	<10 [6]	<10 [6]	<10 [6]
	24+	2,624	18 (0.7)	13 (72.2)	<10 [6]	14 (77.8)
2018/19	<1	4,801	<10 [6]	<10 [6]	<10 [6]	<10 [6]
	1-<6	7,742	<10 [6]	<10 [6]	<10 [6]	<10 [6]
	6-<12	3,616	<10 [6]	<10 [6]	<10 [6]	<10 [6]
	12-<24	3,447	13 (0.4)	13 (100.0)	<10 [6]	13 (100.0)
	24+	2,493	27 (1.1)	18 (66.7)	<10 [6]	18 (66.7)
2019/20	<1	5,745	<10 [6]	<10 [6]	<10 [6]	<10 [6]
	1-<6	9,697	<10 [6]	<10 [6]	<10 [6]	<10 [6]
	6-<12	5,090	<10 [6]	<10 [6]	<10 [6]	<10 [6]
	12-<24	3,244	16 (0.5)	14 (87.5)	<10 [6]	14 (87.5)
	24+	2,035	26 (1.3)	19 (73.1)	<10 [6]	19 (73.1)
**Ethnic Group**						
2017/18	White	15,638	35 (0.2)	30 (85.7)	<10 [6]	31 (88.6)
	Mixed	431	<10 [6]	<10 [6]	<10 [6]	<10 [6]
	Asian or Asian British	813	<10 [6]	<10 [6]	<10 [6]	<10 [6]
	Black or Black British	404	<10 [6]	<10 [6]	<10 [6]	<10 [6]
	Chinese and Other	214	<10 [6]	<10 [6]	<10 [6]	<10 [6]
	Unclassified	372	<10 [6]	<10 [6]	<10 [6]	<10 [6]
2018/19	White	14,911	46 (0.3)	36 (78.3)	<10 [6]	36 (78.3)
	Mixed	371	<10 [6]	<10 [6]	<10 [6]	<10 [6]
	Asian or Asian British	726	<10 [6]	<10 [6]	<10 [6]	<10 [6]
	Black or Black British	364	<10 [6]	<10 [6]	<10 [6]	<10 [6]
	Chinese and Other	167	<10 [6]	<10 [6]	<10 [6]	<10 [6]
	Unclassified	409	<10 [6]	<10 [6]	<10 [6]	<10 [6]
2019/20	White	16,606	48 (0.3)	40 (83.3)	<10 [6]	40 (83.3)
	Mixed	409	<10 [6]	<10 [6]	<10 [6]	<10 [6]
	Asian or Asian British	755	<10 [6]	<10 [6]	<10 [6]	<10 [6]
	Black or Black British	451	<10 [6]	<10 [6]	<10 [6]	<10 [6]
	Chinese and Other	163	<10 [6]	<10 [6]	<10 [6]	<10 [6]
	Unclassified	387	<10 [6]	<10 [6]	<10 [6]	<10 [6]

[1]Eligible for indicator; [2]Achieve in current prison; [3]Achieve in previous prison; [4]Overall achievement - either current or previous prison; [5]Declined indicator; [6]Suppressed (<10) to avoid disclosure

Indicator	*The proportion of people with persistent or paroxysmal atrial fibrillation and a CHA2DS2-VASc score in the preceding 12 months*						*Community achievement 2019/20: 93.35% (QOF 19/20)*
	**Group: Cardiovascular Disease**						
	**Variable**	**Population**	**Eligible[1] (% popln)**	**Satisfy[2] (% eligible)**	**Elsewhere[3] (% eligible)**	**Achieve[4] (% eligible)**	
**Year**							
	2017/18	21,677	22 (0.1)	<10 [6]	<10 [6]	<10 [6]	
	2018/19	22,099	23 (0.1)	12 (52.2)	<10 [6]	12 (52.2)	
	2019/20	25,811	25 (0.1)	15 (60.0)	<10 [6]	15 (60.0)	
**Prison**							
2017/18	Prison 1	1,323	<10 [6]	<10 [6]	<10 [6]	<10 [6]	
	Prison 2	3,261	<10 [6]	<10 [6]	<10 [6]	<10 [6]	
	Prison 3	2,623	<10 [6]	<10 [6]	<10 [6]	<10 [6]	
	Prison 4	2,089	<10 [6]	<10 [6]	<10 [6]	<10 [6]	
	Prison 5	637	<10 [6]	<10 [6]	<10 [6]	<10 [6]	
	Prison 6	1,552	<10 [6]	<10 [6]	<10 [6]	<10 [6]	
	Prison 7	635	<10 [6]	<10 [6]	<10 [6]	<10 [6]	
	Prison 8	1,085	<10 [6]	<10 [6]	<10 [6]	<10 [6]	
	Prison 9	981	<10 [6]	<10 [6]	<10 [6]	<10 [6]	
	Prison 10	2,523	<10 [6]	<10 [6]	<10 [6]	<10 [6]	
	Prison 11	3,470	<10 [6]	<10 [6]	<10 [6]	<10 [6]	
	Prison 12	815	<10 [6]	<10 [6]	<10 [6]	<10 [6]	
	Prison 13	683	<10 [6]	<10 [6]	<10 [6]	<10 [6]	
2018/19	Prison 1	1,333	<10 [6]	<10 [6]	<10 [6]	<10 [6]	
	Prison 2	2,705	<10 [6]	<10 [6]	<10 [6]	<10 [6]	
	Prison 3	2,522	<10 [6]	<10 [6]	<10 [6]	<10 [6]	
	Prison 4	2,349	<10 [6]	<10 [6]	<10 [6]	<10 [6]	
	Prison 5	676	<10 [6]	<10 [6]	<10 [6]	<10 [6]	
	Prison 6	1,513	<10 [6]	<10 [6]	<10 [6]	<10 [6]	
	Prison 7	654	<10 [6]	<10 [6]	<10 [6]	<10 [6]	
	Prison 8	1,148	<10 [6]	<10 [6]	<10 [6]	<10 [6]	
	Prison 9	996	<10 [6]	<10 [6]	<10 [6]	<10 [6]	
	Prison 10	2,717	<10 [6]	<10 [6]	<10 [6]	<10 [6]	
	Prison 11	4,020	<10 [6]	<10 [6]	<10 [6]	<10 [6]	
	Prison 12	792	<10 [6]	<10 [6]	<10 [6]	<10 [6]	
	Prison 13	674	<10 [6]	<10 [6]	<10 [6]	<10 [6]	
2019/20	Prison 1	1,410	<10 [6]	<10 [6]	<10 [6]	<10 [6]	
	Prison 2	2,979	<10 [6]	<10 [6]	<10 [6]	<10 [6]	
	Prison 3	2,809	<10 [6]	<10 [6]	<10 [6]	<10 [6]	
	Prison 4	2,651	<10 [6]	<10 [6]	<10 [6]	<10 [6]	
	Prison 5	616	<10 [6]	<10 [6]	<10 [6]	<10 [6]	
	Prison 6	1,533	<10 [6]	<10 [6]	<10 [6]	<10 [6]	
	Prison 7	860	<10 [6]	<10 [6]	<10 [6]	<10 [6]	
	Prison 8	1,385	<10 [6]	<10 [6]	<10 [6]	<10 [6]	
	Prison 9	1,092	<10 [6]	<10 [6]	<10 [6]	<10 [6]	
	Prison 10	3,577	<10 [6]	<10 [6]	<10 [6]	<10 [6]	
	Prison 11	5,348	<10 [6]	<10 [6]	<10 [6]	<10 [6]	
	Prison 12	805	<10 [6]	<10 [6]	<10 [6]	<10 [6]	
	Prison 13	746	<10 [6]	<10 [6]	<10 [6]	<10 [6]	
**Prison category**							
2017/18	A	1,664	<10 [6]	<10 [6]	<10 [6]	<10 [6]	
	B	9,254	<10 [6]	<10 [6]	<10 [6]	<10 [6]	
	C	6,035	<10 [6]	<10 [6]	<10 [6]	<10 [6]	
	Closed	1,720	<10 [6]	<10 [6]	<10 [6]	<10 [6]	
	D	2,189	<10 [6]	<10 [6]	<10 [6]	<10 [6]	
	YOI	815	<10 [6]	<10 [6]	<10 [6]	<10 [6]	
2018/19	A	1,670	<10 [6]	<10 [6]	<10 [6]	<10 [6]	
	B	9,442	<10 [6]	<10 [6]	<10 [6]	<10 [6]	
	C	6,204	<10 [6]	<10 [6]	<10 [6]	<10 [6]	
	Closed	1,802	<10 [6]	<10 [6]	<10 [6]	<10 [6]	
	D	2,189	<10 [6]	<10 [6]	<10 [6]	<10 [6]	
	YOI	792	<10 [6]	<10 [6]	<10 [6]	<10 [6]	
2019/20	A	1,838	10 (0.5)	<10 [6]	<10 [6]	<10 [6]	
	B	11,904	<10 [6]	<10 [6]	<10 [6]	<10 [6]	
	C	6,870	<10 [6]	<10 [6]	<10 [6]	<10 [6]	
	Closed	2,245	<10 [6]	<10 [6]	<10 [6]	<10 [6]	
	D	2,149	<10 [6]	<10 [6]	<10 [6]	<10 [6]	
	YOI	805	<10 [6]	<10 [6]	<10 [6]	<10 [6]	
**Gender**							
2017/18	F	1,699	<10 [6]	<10 [6]	<10 [6]	<10 [6]	
	M	19,977	21 (0.1)	<10 [6]	<10 [6]	<10 [6]	
2018/19	F	1,802	<10 [6]	<10 [6]	<10 [6]	<10 [6]	
	M	20,295	22 (0.1)	12 (54.5)	<10 [6]	12 (54.5)	
2019/20	F	1,376	<10 [6]	<10 [6]	<10 [6]	<10 [6]	
	M	23,570	24 (0.1)	14 (58.3)	<10 [6]	14 (58.3)	

[1]Eligible for indicator; [2]Achieve in current prison; [3]Achieve in previous prison; [4]Overall achievement - either current or previous prison; [5]Declined indicator; [6]Suppressed (<10) to avoid disclosure

Indicator	*The proportion of people with persistent or paroxysmal atrial fibrillation and a CHA2DS2-VASc score in the preceding 12 months*						*Community achievement 2019/20: 93.35% (QOF 19/20)*
	**Group: Cardiovascular Disease**						
	**Variable**	**Population**	**Eligible[1] (% popln)**	**Satisfy[2] (% eligible)**	**Elsewhere[3] (% eligible)**	**Achieve[4] (% eligible)**	
**Sentence Status**							
2017/18	.	81	<10 [6]	<10 [6]	<10 [6]	<10 [6]	
	Absconded	<10 [6]	<10 [6]	<10 [6]	<10 [6]	<10 [6]	
	Active In	15,872	19 (0.1)	<10 [6]	<10 [6]	<10 [6]	
	Active Out	1,052	<10 [6]	<10 [6]	<10 [6]	<10 [6]	
	Convicted Sentence	2,125	<10 [6]	<10 [6]	<10 [6]	<10 [6]	
	Downgrade in security category	<10 [6]	<10 [6]	<10 [6]	<10 [6]	<10 [6]	
	Internal Cell Move	<10 [6]	<10 [6]	<10 [6]	<10 [6]	<10 [6]	
	Judges Remand	92	<10 [6]	<10 [6]	<10 [6]	<10 [6]	
	Licence Revoke	52	<10 [6]	<10 [6]	<10 [6]	<10 [6]	
	On Remand	1,492	<10 [6]	<10 [6]	<10 [6]	<10 [6]	
	Transfer	910	<10 [6]	<10 [6]	<10 [6]	<10 [6]	
	Upgrade in security category	<10 [6]	<10 [6]	<10 [6]	<10 [6]	<10 [6]	
2018/19	.	88	<10 [6]	<10 [6]	<10 [6]	<10 [6]	
	Absconded	<10 [6]	<10 [6]	<10 [6]	<10 [6]	<10 [6]	
	Active In	18,145	20 (0.1)	11 (55.0)	<10 [6]	11 (55.0)	
	Active Out	835	<10 [6]	<10 [6]	<10 [6]	<10 [6]	
	Convicted Sentence	1,320	<10 [6]	<10 [6]	<10 [6]	<10 [6]	
	Downgrade in security category	<10 [6]	<10 [6]	<10 [6]	<10 [6]	<10 [6]	
	Internal Cell Move	<10 [6]	<10 [6]	<10 [6]	<10 [6]	<10 [6]	
	Judges Remand	<10 [6]	<10 [6]	<10 [6]	<10 [6]	<10 [6]	
	Licence Revoke	125	<10 [6]	<10 [6]	<10 [6]	<10 [6]	
	On Remand	1,059	<10 [6]	<10 [6]	<10 [6]	<10 [6]	
	Transfer	518	<10 [6]	<10 [6]	<10 [6]	<10 [6]	
	Upgrade in security category	<10 [6]	<10 [6]	<10 [6]	<10 [6]	<10 [6]	
2019/20	.	69	<10 [6]	<10 [6]	<10 [6]	<10 [6]	
	Absconded	<10 [6]	<10 [6]	<10 [6]	<10 [6]	<10 [6]	
	Active In	22,424	21 (0.1)	12 (57.1)	<10 [6]	12 (57.1)	
	Active Out	625	<10 [6]	<10 [6]	<10 [6]	<10 [6]	
	Convicted Sentence	1,361	<10 [6]	<10 [6]	<10 [6]	<10 [6]	
	Downgrade in security category	<10 [6]	<10 [6]	<10 [6]	<10 [6]	<10 [6]	
	Internal Cell Move	<10 [6]	<10 [6]	<10 [6]	<10 [6]	<10 [6]	
	Judges Remand	19	<10 [6]	<10 [6]	<10 [6]	<10 [6]	
	Licence Revoke	178	<10 [6]	<10 [6]	<10 [6]	<10 [6]	
	On Remand	1,031	<10 [6]	<10 [6]	<10 [6]	<10 [6]	
	Transfer	101	<10 [6]	<10 [6]	<10 [6]	<10 [6]	
	Upgrade in security category	<10 [6]	<10 [6]	<10 [6]	<10 [6]	<10 [6]	
**Age - years**							
2017/18	10 - <20	468	<10 [6]	<10 [6]	<10 [6]	<10 [6]	
	20 - <30	6,994	<10 [6]	<10 [6]	<10 [6]	<10 [6]	
	30 - <40	7,051	<10 [6]	<10 [6]	<10 [6]	<10 [6]	
	40 - <50	4,114	<10 [6]	<10 [6]	<10 [6]	<10 [6]	
	50 - <60	2,107	<10 [6]	<10 [6]	<10 [6]	<10 [6]	
	60 - <70	684	<10 [6]	<10 [6]	<10 [6]	<10 [6]	
	70 - <80	213	<10 [6]	<10 [6]	<10 [6]	<10 [6]	
	80 - <90	40	<10 [6]	<10 [6]	<10 [6]	<10 [6]	
	90 - <100	<10 [6]	<10 [6]	<10 [6]	<10 [6]	<10 [6]	
	100 - <110	<10 [6]	<10 [6]	<10 [6]	<10 [6]	<10 [6]	
2018/19	10 - <20	436	<10 [6]	<10 [6]	<10 [6]	<10 [6]	
	20 - <30	7,163	<10 [6]	<10 [6]	<10 [6]	<10 [6]	
	30 - <40	7,381	<10 [6]	<10 [6]	<10 [6]	<10 [6]	
	40 - <50	4,180	<10 [6]	<10 [6]	<10 [6]	<10 [6]	
	50 - <60	1,978	<10 [6]	<10 [6]	<10 [6]	<10 [6]	
	60 - <70	701	<10 [6]	<10 [6]	<10 [6]	<10 [6]	
	70 - <80	209	<10 [6]	<10 [6]	<10 [6]	<10 [6]	
	80 - <90	45	<10 [6]	<10 [6]	<10 [6]	<10 [6]	
	90 - <100	<10 [6]	<10 [6]	<10 [6]	<10 [6]	<10 [6]	
	100 - <110	<10 [6]	<10 [6]	<10 [6]	<10 [6]	<10 [6]	
2019/20	10 - <20	404	<10 [6]	<10 [6]	<10 [6]	<10 [6]	
	20 - <30	8,064	<10 [6]	<10 [6]	<10 [6]	<10 [6]	
	30 - <40	9,125	<10 [6]	<10 [6]	<10 [6]	<10 [6]	
	40 - <50	4,948	<10 [6]	<10 [6]	<10 [6]	<10 [6]	
	50 - <60	2,224	<10 [6]	<10 [6]	<10 [6]	<10 [6]	
	60 - <70	751	<10 [6]	<10 [6]	<10 [6]	<10 [6]	
	70 - <80	238	<10 [6]	<10 [6]	<10 [6]	<10 [6]	
	80 - <90	53	<10 [6]	<10 [6]	<10 [6]	<10 [6]	
	90 - <100	<10 [6]	<10 [6]	<10 [6]	<10 [6]	<10 [6]	
	100 - <110	<10 [6]	<10 [6]	<10 [6]	<10 [6]	<10 [6]	

[1]Eligible for indicator; [2]Achieve in current prison; [3]Achieve in previous prison; [4]Overall achievement - either current or previous prison; [5]Declined indicator; [6]Suppressed (<10) to avoid disclosure

Indicator	*The proportion of people with persistent or paroxysmal atrial fibrillation and a CHA2DS2-VASc score in the preceding 12 months*					*Community achievement 2019/20: 93.35% (QOF 19/20)*
	**Group: Cardiovascular Disease**					
	**Variable**	**Population**	**Eligible[1] (% popln)**	**Satisfy[2] (% eligible)**	**Elsewhere[3] (% eligible)**	**Achieve[4] (% eligible)**
**Length of Stay (months)**						
2017/18	<1	4,474	<10 [6]	<10 [6]	<10 [6]	<10 [6]
	1-<6	8,075	<10 [6]	<10 [6]	<10 [6]	<10 [6]
	6-<12	3,672	<10 [6]	<10 [6]	<10 [6]	<10 [6]
	12-<24	2,832	<10 [6]	<10 [6]	<10 [6]	<10 [6]
	24+	2,624	11 (0.4)	<10 [6]	<10 [6]	<10 [6]
2018/19	<1	4,801	<10 [6]	<10 [6]	<10 [6]	<10 [6]
	1-<6	7,742	<10 [6]	<10 [6]	<10 [6]	<10 [6]
	6-<12	3,616	<10 [6]	<10 [6]	<10 [6]	<10 [6]
	12-<24	3,447	<10 [6]	<10 [6]	<10 [6]	<10 [6]
	24+	2,493	13 (0.5)	<10 [6]	<10 [6]	<10 [6]
2019/20	<1	5,745	<10 [6]	<10 [6]	<10 [6]	<10 [6]
	1-<6	9,697	<10 [6]	<10 [6]	<10 [6]	<10 [6]
	6-<12	5,090	<10 [6]	<10 [6]	<10 [6]	<10 [6]
	12-<24	3,244	<10 [6]	<10 [6]	<10 [6]	<10 [6]
	24+	2,035	12 (0.6)	<10 [6]	<10 [6]	<10 [6]
**Ethnic Group**						
2017/18	White	15,638	22 (0.1)	<10 [6]	<10 [6]	<10 [6]
	Mixed	431	<10 [6]	<10 [6]	<10 [6]	<10 [6]
	Asian or Asian British	813	<10 [6]	<10 [6]	<10 [6]	<10 [6]
	Black or Black British	404	<10 [6]	<10 [6]	<10 [6]	<10 [6]
	Chinese and Other	214	<10 [6]	<10 [6]	<10 [6]	<10 [6]
	Unclassified	372	<10 [6]	<10 [6]	<10 [6]	<10 [6]
2018/19	White	14,911	23 (0.2)	12 (52.2)	<10 [6]	12 (52.2)
	Mixed	371	<10 [6]	<10 [6]	<10 [6]	<10 [6]
	Asian or Asian British	726	<10 [6]	<10 [6]	<10 [6]	<10 [6]
	Black or Black British	364	<10 [6]	<10 [6]	<10 [6]	<10 [6]
	Chinese and Other	167	<10 [6]	<10 [6]	<10 [6]	<10 [6]
	Unclassified	409	<10 [6]	<10 [6]	<10 [6]	<10 [6]
2019/20	White	16,606	23 (0.1)	14 (60.9)	<10 [6]	14 (60.9)
	Mixed	409	<10 [6]	<10 [6]	<10 [6]	<10 [6]
	Asian or Asian British	755	<10 [6]	<10 [6]	<10 [6]	<10 [6]
	Black or Black British	451	<10 [6]	<10 [6]	<10 [6]	<10 [6]
	Chinese and Other	163	<10 [6]	<10 [6]	<10 [6]	<10 [6]
	Unclassified	387	<10 [6]	<10 [6]	<10 [6]	<10 [6]

[1]Eligible for indicator; [2]Achieve in current prison; [3]Achieve in previous prison; [4]Overall achievement - either current or previous prison; [5]Declined indicator; [6]Suppressed (<10) to avoid disclosure

Indicator	*The proportion of people aged 79 years or under, with coronary heart disease, hypertension, stroke, transient ischaemic attack or peripheral arterial disease, who have had a blood pressure of 140/90mmHg or less in the preceding 12 months*						*Community achievement 2019/20: CHD: 77.77%, Hypertension: 67.28%, Stroke/TIA: 73.92%, PAD not recorded*
	**Group: Cardiovascular Disease**						
	**Variable**	**Population**	**Eligible[1] (% popln)**	**Satisfy[2] (% eligible)**	**Elsewhere[3] (% eligible)**	**Achieve[4] (% eligible)**	
**Year**							
	2017/18	21,677	989 (4.6)	457 (46.2)	<10 [6]	465 (47.0)	
	2018/19	22,099	1,103 (5.0)	491 (44.5)	19 (1.7)	510 (46.2)	
	2019/20	25,811	1,311 (5.1)	543 (41.4)	43 (3.3)	586 (44.7)	
**Prison**							
2017/18	Prison 1	1,323	69 (5.2)	33 (47.8)	<10 [6]	34 (49.3)	
	Prison 2	3,261	114 (3.5)	42 (36.8)	<10 [6]	42 (36.8)	
	Prison 3	2,623	81 (3.1)	30 (37.0)	<10 [6]	31 (38.3)	
	Prison 4	2,089	113 (5.4)	57 (50.4)	<10 [6]	61 (54.0)	
	Prison 5	637	32 (5.0)	20 (62.5)	<10 [6]	20 (62.5)	
	Prison 6	1,552	85 (5.5)	54 (63.5)	<10 [6]	54 (63.5)	
	Prison 7	635	31 (4.9)	14 (45.2)	<10 [6]	15 (48.4)	
	Prison 8	1,085	49 (4.5)	31 (63.3)	<10 [6]	31 (63.3)	
	Prison 9	981	125 (12.7)	52 (41.6)	<10 [6]	52 (41.6)	
	Prison 10	2,523	126 (5.0)	64 (50.8)	<10 [6]	64 (50.8)	
	Prison 11	3,470	99 (2.9)	31 (31.3)	<10 [6]	31 (31.3)	
	Prison 12	815	<10 [6]	<10 [6]	<10 [6]	<10 [6]	
	Prison 13	683	65 (9.5)	29 (44.6)	<10 [6]	30 (46.2)	
2018/19	Prison 1	1,333	58 (4.4)	32 (55.2)	<10 [6]	33 (56.9)	
	Prison 2	2,705	89 (3.3)	30 (33.7)	<10 [6]	31 (34.8)	
	Prison 3	2,522	91 (3.6)	25 (27.5)	<10 [6]	26 (28.6)	
	Prison 4	2,349	141 (6.0)	78 (55.3)	<10 [6]	84 (59.6)	
	Prison 5	676	43 (6.4)	26 (60.5)	<10 [6]	27 (62.8)	
	Prison 6	1,513	82 (5.4)	51 (62.2)	<10 [6]	51 (62.2)	
	Prison 7	654	48 (7.3)	21 (43.8)	<10 [6]	21 (43.8)	
	Prison 8	1,148	60 (5.2)	43 (71.7)	<10 [6]	45 (75.0)	
	Prison 9	996	125 (12.6)	43 (34.4)	<10 [6]	47 (37.6)	
	Prison 10	2,717	133 (4.9)	70 (52.6)	<10 [6]	70 (52.6)	
	Prison 11	4,020	150 (3.7)	46 (30.7)	<10 [6]	46 (30.7)	
	Prison 12	792	<10 [6]	<10 [6]	<10 [6]	<10 [6]	
	Prison 13	674	80 (11.9)	25 (31.3)	<10 [6]	28 (35.0)	
2019/20	Prison 1	1,410	64 (4.5)	29 (45.3)	<10 [6]	32 (50.0)	
	Prison 2	2,979	100 (3.4)	26 (26.0)	<10 [6]	28 (28.0)	
	Prison 3	2,809	107 (3.8)	50 (46.7)	<10 [6]	53 (49.5)	
	Prison 4	2,651	239 (9.0)	88 (36.8)	15 (6.3)	103 (43.1)	
	Prison 5	616	39 (6.3)	18 (46.2)	<10 [6]	18 (46.2)	
	Prison 6	1,533	79 (5.2)	41 (51.9)	<10 [6]	41 (51.9)	
	Prison 7	860	47 (5.5)	29 (61.7)	<10 [6]	29 (61.7)	
	Prison 8	1,385	62 (4.5)	40 (64.5)	<10 [6]	41 (66.1)	
	Prison 9	1,092	155 (14.2)	52 (33.5)	<10 [6]	60 (38.7)	
	Prison 10	3,577	160 (4.5)	90 (56.3)	<10 [6]	92 (57.5)	
	Prison 11	5,348	174 (3.3)	45 (25.9)	<10 [6]	47 (27.0)	
	Prison 12	805	<10 [6]	<10 [6]	<10 [6]	<10 [6]	
	Prison 13	746	84 (11.3)	34 (40.5)	<10 [6]	41 (48.8)	
**Prison category**							
2017/18	A	1,664	190 (11.4)	81 (42.6)	<10 [6]	82 (43.2)	
	B	9,254	339 (3.7)	137 (40.4)	<10 [6]	137 (40.4)	
	C	6,035	263 (4.4)	120 (45.6)	<10 [6]	126 (47.9)	
	Closed	1,720	80 (4.7)	45 (56.3)	<10 [6]	46 (57.5)	
	D	2,189	117 (5.3)	74 (63.2)	<10 [6]	74 (63.2)	
	YOI	815	<10 [6]	<10 [6]	<10 [6]	<10 [6]	
2018/19	A	1,670	205 (12.3)	68 (33.2)	<10 [6]	75 (36.6)	
	B	9,442	372 (3.9)	146 (39.2)	<10 [6]	147 (39.5)	
	C	6,204	290 (4.7)	135 (46.6)	<10 [6]	143 (49.3)	
	Closed	1,802	108 (6.0)	64 (59.3)	<10 [6]	66 (61.1)	
	D	2,189	125 (5.7)	77 (61.6)	<10 [6]	78 (62.4)	
	YOI	792	<10 [6]	<10 [6]	<10 [6]	<10 [6]	
2019/20	A	1,838	239 (13.0)	86 (36.0)	15 (6.3)	101 (42.3)	
	B	11,904	434 (3.6)	161 (37.1)	<10 [6]	167 (38.5)	
	C	6,870	410 (6.0)	167 (40.7)	21 (5.1)	188 (45.9)	
	Closed	2,245	109 (4.9)	69 (63.3)	<10 [6]	70 (64.2)	
	D	2,149	118 (5.5)	59 (50.0)	<10 [6]	59 (50.0)	
	YOI	805	<10 [6]	<10 [6]	<10 [6]	<10 [6]	
**Gender**							
2017/18	F	1,699	78 (4.6)	45 (57.7)	<10 [6]	46 (59.0)	
	M	19,977	911 (4.6)	412 (45.2)	<10 [6]	419 (46.0)	
2018/19	F	1,802	107 (5.9)	63 (58.9)	<10 [6]	65 (60.7)	
	M	20,295	996 (4.9)	428 (43.0)	17 (1.7)	445 (44.7)	
2019/20	F	1,376	62 (4.5)	40 (64.5)	<10 [6]	41 (66.1)	
	M	23,570	1,202 (5.1)	475 (39.5)	42 (3.5)	517 (43.0)	

[1]Eligible for indicator; [2]Achieve in current prison; [3]Achieve in previous prison; [4]Overall achievement - either current or previous prison; [5]Declined indicator; [6]Suppressed (<10) to avoid disclosure

Indicator *The proportion of people aged 79 years or under, with coronary heart disease, hypertension, stroke, transient ischaemic attack or peripheral arterial disease, who have had a blood pressure of 140/90mmHg or less in the preceding 12 months*

Community achievement 2019/20: CHD: 77.77%, Hypertension: 67.28%, Stroke/TIA: 73.92%, PAD not recorded

Group: Cardiovascular Disease

	Variable	Population	Eligible[1] (% popln)	Satisfy[2] (% eligible)	Elsewhere[3] (% eligible)	Achieve[4] (% eligible)
**Sentence Status**						
2017/18	.	81	<10 [6]	<10 [6]	<10 [6]	<10 [6]
	Absconded	<10 [6]	<10 [6]	<10 [6]	<10 [6]	<10 [6]
	Active In	15,872	751 (4.7)	349 (46.5)	<10 [6]	357 (47.5)
	Active Out	1,052	47 (4.5)	23 (48.9)	<10 [6]	23 (48.9)
	Convicted Sentence	2,125	76 (3.6)	25 (32.9)	<10 [6]	25 (32.9)
	Downgrade in security category	<10 [6]	<10 [6]	<10 [6]	<10 [6]	<10 [6]
	Internal Cell Move	<10 [6]	<10 [6]	<10 [6]	<10 [6]	<10 [6]
	Judges Remand	92	<10 [6]	<10 [6]	<10 [6]	<10 [6]
	Licence Revoke	52	<10 [6]	<10 [6]	<10 [6]	<10 [6]
	On Remand	1,492	53 (3.6)	25 (47.2)	<10 [6]	25 (47.2)
	Transfer	910	52 (5.7)	32 (61.5)	<10 [6]	32 (61.5)
	Upgrade in security category	<10 [6]	<10 [6]	<10 [6]	<10 [6]	<10 [6]
2018/19	.	88	<10 [6]	<10 [6]	<10 [6]	<10 [6]
	Absconded	<10 [6]	<10 [6]	<10 [6]	<10 [6]	<10 [6]
	Active In	18,145	939 (5.2)	421 (44.8)	17 (1.8)	438 (46.6)
	Active Out	835	46 (5.5)	24 (52.2)	<10 [6]	25 (54.3)
	Convicted Sentence	1,320	38 (2.9)	12 (31.6)	<10 [6]	13 (34.2)
	Downgrade in security category	<10 [6]	<10 [6]	<10 [6]	<10 [6]	<10 [6]
	Internal Cell Move	<10 [6]	<10 [6]	<10 [6]	<10 [6]	<10 [6]
	Judges Remand	<10 [6]	<10 [6]	<10 [6]	<10 [6]	<10 [6]
	Licence Revoke	125	<10 [6]	<10 [6]	<10 [6]	<10 [6]
	On Remand	1,059	46 (4.3)	16 (34.8)	<10 [6]	16 (34.8)
	Transfer	518	28 (5.4)	17 (60.7)	<10 [6]	17 (60.7)
	Upgrade in security category	<10 [6]	<10 [6]	<10 [6]	<10 [6]	<10 [6]
2019/20	.	69	<10 [6]	<10 [6]	<10 [6]	<10 [6]
	Absconded	<10 [6]	<10 [6]	<10 [6]	<10 [6]	<10 [6]
	Active In	22,424	1,187 (5.3)	505 (42.5)	42 (3.5)	547 (46.1)
	Active Out	625	29 (4.6)	<10 [6]	<10 [6]	<10 [6]
	Convicted Sentence	1,361	45 (3.3)	12 (26.7)	<10 [6]	12 (26.7)
	Downgrade in security category	<10 [6]	<10 [6]	<10 [6]	<10 [6]	<10 [6]
	Internal Cell Move	<10 [6]	<10 [6]	<10 [6]	<10 [6]	<10 [6]
	Judges Remand	19	<10 [6]	<10 [6]	<10 [6]	<10 [6]
	Licence Revoke	178	<10 [6]	<10 [6]	<10 [6]	<10 [6]
	On Remand	1,031	37 (3.6)	11 (29.7)	<10 [6]	12 (32.4)
	Transfer	101	<10 [6]	<10 [6]	<10 [6]	<10 [6]
	Upgrade in security category	<10 [6]	<10 [6]	<10 [6]	<10 [6]	<10 [6]
**Age - years**						
2017/18	10 - <20	468	<10 [6]	<10 [6]	<10 [6]	<10 [6]
	20 - <30	6,994	30 (0.4)	19 (63.3)	<10 [6]	21 (70.0)
	30 - <40	7,051	120 (1.7)	57 (47.5)	<10 [6]	57 (47.5)
	40 - <50	4,114	264 (6.4)	115 (43.6)	<10 [6]	117 (44.3)
	50 - <60	2,107	323 (15.3)	149 (46.1)	<10 [6]	152 (47.1)
	60 - <70	684	180 (26.3)	88 (48.9)	<10 [6]	89 (49.4)
	70 - <80	213	71 (33.3)	28 (39.4)	<10 [6]	28 (39.4)
	80 - <90	40	<10 [6]	<10 [6]	<10 [6]	<10 [6]
	90 - <100	<10 [6]	<10 [6]	<10 [6]	<10 [6]	<10 [6]
	100 - <110	<10 [6]	<10 [6]	<10 [6]	<10 [6]	<10 [6]
2018/19	10 - <20	436	<10 [6]	<10 [6]	<10 [6]	<10 [6]
	20 - <30	7,163	46 (0.6)	22 (47.8)	<10 [6]	24 (52.2)
	30 - <40	7,381	157 (2.1)	77 (49.0)	<10 [6]	80 (51.0)
	40 - <50	4,180	284 (6.8)	126 (44.4)	<10 [6]	131 (46.1)
	50 - <60	1,978	344 (17.4)	158 (45.9)	<10 [6]	162 (47.1)
	60 - <70	701	196 (28.0)	78 (39.8)	<10 [6]	80 (40.8)
	70 - <80	209	76 (36.4)	30 (39.5)	<10 [6]	33 (43.4)
	80 - <90	45	<10 [6]	<10 [6]	<10 [6]	<10 [6]
	90 - <100	<10 [6]	<10 [6]	<10 [6]	<10 [6]	<10 [6]
	100 - <110	<10 [6]	<10 [6]	<10 [6]	<10 [6]	<10 [6]
2019/20	10 - <20	404	<10 [6]	<10 [6]	<10 [6]	<10 [6]
	20 - <30	8,064	56 (0.7)	25 (44.6)	<10 [6]	27 (48.2)
	30 - <40	9,125	207 (2.3)	101 (48.8)	<10 [6]	110 (53.1)
	40 - <50	4,948	310 (6.3)	130 (41.9)	<10 [6]	137 (44.2)
	50 - <60	2,224	423 (19.0)	166 (39.2)	15 (3.5)	181 (42.8)
	60 - <70	751	220 (29.3)	84 (38.2)	<10 [6]	89 (40.5)
	70 - <80	238	93 (39.1)	35 (37.6)	<10 [6]	40 (43.0)
	80 - <90	53	<10 [6]	<10 [6]	<10 [6]	<10 [6]
	90 - <100	<10 [6]	<10 [6]	<10 [6]	<10 [6]	<10 [6]
	100 - <110	<10 [6]	<10 [6]	<10 [6]	<10 [6]	<10 [6]

[1]Eligible for indicator; [2]Achieve in current prison; [3]Achieve in previous prison; [4]Overall achievement - either current or previous prison; [5]Declined indicator; [6]Suppressed (<10) to avoid disclosure

Indicator	The proportion of people aged 79 years or under, with coronary heart disease, hypertension, stroke, transient ischaemic attack or peripheral arterial disease, who have had a blood pressure of 140/90mmHg or less in the preceding 12 months					Community achievement 2019/20: CHD: 77.77%, Hypertension: 67.28%, Stroke/TIA: 73.92%, PAD not recorded
	Group: Cardiovascular Disease					
	Variable	Population	Eligible[1] (% popln)	Satisfy[2] (% eligible)	Elsewhere[3] (% eligible)	Achieve[4] (% eligible)
**Length of Stay (months)**						
2017/18	<1	4,474	114 (2.5)	46 (40.4)	<10 [6]	46 (40.4)
	1-<6	8,075	282 (3.5)	127 (45.0)	<10 [6]	134 (47.5)
	6-<12	3,672	159 (4.3)	79 (49.7)	<10 [6]	80 (50.3)
	12-<24	2,832	135 (4.8)	59 (43.7)	<10 [6]	59 (43.7)
	24+	2,624	299 (11.4)	146 (48.8)	<10 [6]	146 (48.8)
2018/19	<1	4,801	163 (3.4)	64 (39.3)	<10 [6]	64 (39.3)
	1-<6	7,742	313 (4.0)	148 (47.3)	<10 [6]	154 (49.2)
	6-<12	3,616	144 (4.0)	61 (42.4)	<10 [6]	61 (42.4)
	12-<24	3,447	191 (5.5)	94 (49.2)	<10 [6]	103 (53.9)
	24+	2,493	292 (11.7)	124 (42.5)	<10 [6]	128 (43.8)
2019/20	<1	5,745	160 (2.8)	65 (40.6)	<10 [6]	67 (41.9)
	1-<6	9,697	383 (3.9)	153 (39.9)	15 (3.9)	168 (43.9)
	6-<12	5,090	259 (5.1)	96 (37.1)	19 (7.3)	115 (44.4)
	12-<24	3,244	223 (6.9)	94 (42.2)	<10 [6]	97 (43.5)
	24+	2,035	286 (14.1)	135 (47.2)	<10 [6]	139 (48.6)
**Ethnic Group**						
2017/18	White	15,638	839 (5.4)	398 (47.4)	<10 [6]	404 (48.2)
	Mixed	431	<10 [6]	<10 [6]	<10 [6]	<10 [6]
	Asian or Asian British	813	23 (2.8)	13 (56.5)	<10 [6]	14 (60.9)
	Black or Black British	404	12 (3.0)	<10 [6]	<10 [6]	<10 [6]
	Chinese and Other	214	13 (6.1)	<10 [6]	<10 [6]	<10 [6]
	Unclassified	372	17 (4.6)	<10 [6]	<10 [6]	<10 [6]
2018/19	White	14,911	864 (5.8)	375 (43.4)	15 (1.7)	390 (45.1)
	Mixed	371	13 (3.5)	<10 [6]	<10 [6]	<10 [6]
	Asian or Asian British	726	25 (3.4)	16 (64.0)	<10 [6]	16 (64.0)
	Black or Black British	364	17 (4.7)	<10 [6]	<10 [6]	<10 [6]
	Chinese and Other	167	<10 [6]	<10 [6]	<10 [6]	<10 [6]
	Unclassified	409	17 (4.2)	<10 [6]	<10 [6]	<10 [6]
2019/20	White	16,606	956 (5.8)	416 (43.5)	30 (3.1)	446 (46.7)
	Mixed	409	12 (2.9)	<10 [6]	<10 [6]	<10 [6]
	Asian or Asian British	755	33 (4.4)	20 (60.6)	<10 [6]	22 (66.7)
	Black or Black British	451	21 (4.7)	<10 [6]	<10 [6]	<10 [6]
	Chinese and Other	163	<10 [6]	<10 [6]	<10 [6]	<10 [6]
	Unclassified	387	21 (5.4)	<10 [6]	<10 [6]	<10 [6]

[1]Eligible for indicator; [2]Achieve in current prison; [3]Achieve in previous prison; [4]Overall achievement - either current or previous prison; [5]Declined indicator; [6]Suppressed (<10) to avoid disclosure

Indicator *The proportion of people aged 80 years or over, with coronary heart disease, hypertension, stroke, transient ischaemic attack or peripheral arterial disease, who have had a blood pressure of 150/90mmHg or less in the preceding 12 months*

Community achievement 2019/20: CHD: 86.5%, Hypertension: 81.97%, Stoke/TIA: 84.9%, PAD not recorded

Group: Cardiovascular Disease

	**Variable**	**Population**	**Eligible[1] (% popln)**	**Satisfy[2] (% eligible)**	**Elsewhere[3] (% eligible)**	**Achieve[4] (% eligible)**
**Year**						
	2017/18	21,677	<10 [6]	<10 [6]	<10 [6]	<10 [6]
	2018/19	22,099	12 (0.1)	<10 [6]	<10 [6]	<10 [6]
	2019/20	25,811	19 (0.1)	<10 [6]	<10 [6]	10 (52.6)
**Prison**						
2017/18	Prison 1	1,323	<10 [6]	<10 [6]	<10 [6]	<10 [6]
	Prison 2	3,261	<10 [6]	<10 [6]	<10 [6]	<10 [6]
	Prison 3	2,623	<10 [6]	<10 [6]	<10 [6]	<10 [6]
	Prison 4	2,089	<10 [6]	<10 [6]	<10 [6]	<10 [6]
	Prison 5	637	<10 [6]	<10 [6]	<10 [6]	<10 [6]
	Prison 6	1,552	<10 [6]	<10 [6]	<10 [6]	<10 [6]
	Prison 7	635	<10 [6]	<10 [6]	<10 [6]	<10 [6]
	Prison 8	1,085	<10 [6]	<10 [6]	<10 [6]	<10 [6]
	Prison 9	981	<10 [6]	<10 [6]	<10 [6]	<10 [6]
	Prison 10	2,523	<10 [6]	<10 [6]	<10 [6]	<10 [6]
	Prison 11	3,470	<10 [6]	<10 [6]	<10 [6]	<10 [6]
	Prison 12	815	<10 [6]	<10 [6]	<10 [6]	<10 [6]
	Prison 13	683	<10 [6]	<10 [6]	<10 [6]	<10 [6]
2018/19	Prison 1	1,333	<10 [6]	<10 [6]	<10 [6]	<10 [6]
	Prison 2	2,705	<10 [6]	<10 [6]	<10 [6]	<10 [6]
	Prison 3	2,522	<10 [6]	<10 [6]	<10 [6]	<10 [6]
	Prison 4	2,349	<10 [6]	<10 [6]	<10 [6]	<10 [6]
	Prison 5	676	<10 [6]	<10 [6]	<10 [6]	<10 [6]
	Prison 6	1,513	<10 [6]	<10 [6]	<10 [6]	<10 [6]
	Prison 7	654	<10 [6]	<10 [6]	<10 [6]	<10 [6]
	Prison 8	1,148	<10 [6]	<10 [6]	<10 [6]	<10 [6]
	Prison 9	996	<10 [6]	<10 [6]	<10 [6]	<10 [6]
	Prison 10	2,717	<10 [6]	<10 [6]	<10 [6]	<10 [6]
	Prison 11	4,020	<10 [6]	<10 [6]	<10 [6]	<10 [6]
	Prison 12	792	<10 [6]	<10 [6]	<10 [6]	<10 [6]
	Prison 13	674	<10 [6]	<10 [6]	<10 [6]	<10 [6]
2019/20	Prison 1	1,410	<10 [6]	<10 [6]	<10 [6]	<10 [6]
	Prison 2	2,979	<10 [6]	<10 [6]	<10 [6]	<10 [6]
	Prison 3	2,809	<10 [6]	<10 [6]	<10 [6]	<10 [6]
	Prison 4	2,651	<10 [6]	<10 [6]	<10 [6]	<10 [6]
	Prison 5	616	<10 [6]	<10 [6]	<10 [6]	<10 [6]
	Prison 6	1,533	<10 [6]	<10 [6]	<10 [6]	<10 [6]
	Prison 7	860	<10 [6]	<10 [6]	<10 [6]	<10 [6]
	Prison 8	1,385	<10 [6]	<10 [6]	<10 [6]	<10 [6]
	Prison 9	1,092	<10 [6]	<10 [6]	<10 [6]	<10 [6]
	Prison 10	3,577	<10 [6]	<10 [6]	<10 [6]	<10 [6]
	Prison 11	5,348	<10 [6]	<10 [6]	<10 [6]	<10 [6]
	Prison 12	805	<10 [6]	<10 [6]	<10 [6]	<10 [6]
	Prison 13	746	<10 [6]	<10 [6]	<10 [6]	<10 [6]
**Prison category**						
2017/18	A	1,664	<10 [6]	<10 [6]	<10 [6]	<10 [6]
	B	9,254	<10 [6]	<10 [6]	<10 [6]	<10 [6]
	C	6,035	<10 [6]	<10 [6]	<10 [6]	<10 [6]
	Closed	1,720	<10 [6]	<10 [6]	<10 [6]	<10 [6]
	D	2,189	<10 [6]	<10 [6]	<10 [6]	<10 [6]
	YOI	815	<10 [6]	<10 [6]	<10 [6]	<10 [6]
2018/19	A	1,670	<10 [6]	<10 [6]	<10 [6]	<10 [6]
	B	9,442	<10 [6]	<10 [6]	<10 [6]	<10 [6]
	C	6,204	<10 [6]	<10 [6]	<10 [6]	<10 [6]
	Closed	1,802	<10 [6]	<10 [6]	<10 [6]	<10 [6]
	D	2,189	<10 [6]	<10 [6]	<10 [6]	<10 [6]
	YOI	792	<10 [6]	<10 [6]	<10 [6]	<10 [6]
2019/20	A	1,838	<10 [6]	<10 [6]	<10 [6]	<10 [6]
	B	11,904	<10 [6]	<10 [6]	<10 [6]	<10 [6]
	C	6,870	<10 [6]	<10 [6]	<10 [6]	<10 [6]
	Closed	2,245	<10 [6]	<10 [6]	<10 [6]	<10 [6]
	D	2,149	<10 [6]	<10 [6]	<10 [6]	<10 [6]
	YOI	805	<10 [6]	<10 [6]	<10 [6]	<10 [6]
**Gender**						
2017/18	F	1,699	<10 [6]	<10 [6]	<10 [6]	<10 [6]
	M	19,977	<10 [6]	<10 [6]	<10 [6]	<10 [6]
2018/19	F	1,802	<10 [6]	<10 [6]	<10 [6]	<10 [6]
	M	20,295	12 (0.1)	<10 [6]	<10 [6]	<10 [6]
2019/20	F	1,376	<10 [6]	<10 [6]	<10 [6]	<10 [6]
	M	23,570	19 (0.1)	<10 [6]	<10 [6]	10 (52.6)

[1]Eligible for indicator; [2]Achieve in current prison; [3]Achieve in previous prison; [4]Overall achievement - either current or previous prison; [5]Declined indicator; [6]Suppressed (<10) to avoid disclosure

Indicator *The proportion of people aged 80 years or over, with coronary heart disease, hypertension, stroke, transient ischaemic attack or peripheral arterial disease, who have had a blood pressure of 150/90mmHg or less in the preceding 12 months*

Community achievement 2019/20: CHD: 86.5%, Hypertension: 81.97%, Stoke/TIA: 84.9%, PAD not recorded

Group: Cardiovascular Disease

	Variable	Population	Eligible[1] (% popln)	Satisfy[2] (% eligible)	Elsewhere[3] (% eligible)	Achieve[4] (% eligible)
**Sentence Status**						
2017/18	.	81	<10 [6]	<10 [6]	<10 [6]	<10 [6]
	Absconded	<10 [6]	<10 [6]	<10 [6]	<10 [6]	<10 [6]
	Active In	15,872	<10 [6]	<10 [6]	<10 [6]	<10 [6]
	Active Out	1,052	<10 [6]	<10 [6]	<10 [6]	<10 [6]
	Convicted Sentence	2,125	<10 [6]	<10 [6]	<10 [6]	<10 [6]
	Downgrade in security category	<10 [6]	<10 [6]	<10 [6]	<10 [6]	<10 [6]
	Internal Cell Move	<10 [6]	<10 [6]	<10 [6]	<10 [6]	<10 [6]
	Judges Remand	92	<10 [6]	<10 [6]	<10 [6]	<10 [6]
	Licence Revoke	52	<10 [6]	<10 [6]	<10 [6]	<10 [6]
	On Remand	1,492	<10 [6]	<10 [6]	<10 [6]	<10 [6]
	Transfer	910	<10 [6]	<10 [6]	<10 [6]	<10 [6]
	Upgrade in security category	<10 [6]	<10 [6]	<10 [6]	<10 [6]	<10 [6]
2018/19	.	88	<10 [6]	<10 [6]	<10 [6]	<10 [6]
	Absconded	<10 [6]	<10 [6]	<10 [6]	<10 [6]	<10 [6]
	Active In	18,145	<10 [6]	<10 [6]	<10 [6]	<10 [6]
	Active Out	835	<10 [6]	<10 [6]	<10 [6]	<10 [6]
	Convicted Sentence	1,320	<10 [6]	<10 [6]	<10 [6]	<10 [6]
	Downgrade in security category	<10 [6]	<10 [6]	<10 [6]	<10 [6]	<10 [6]
	Internal Cell Move	<10 [6]	<10 [6]	<10 [6]	<10 [6]	<10 [6]
	Judges Remand	<10 [6]	<10 [6]	<10 [6]	<10 [6]	<10 [6]
	Licence Revoke	125	<10 [6]	<10 [6]	<10 [6]	<10 [6]
	On Remand	1,059	<10 [6]	<10 [6]	<10 [6]	<10 [6]
	Transfer	518	<10 [6]	<10 [6]	<10 [6]	<10 [6]
	Upgrade in security category	<10 [6]	<10 [6]	<10 [6]	<10 [6]	<10 [6]
2019/20	.	69	<10 [6]	<10 [6]	<10 [6]	<10 [6]
	Absconded	<10 [6]	<10 [6]	<10 [6]	<10 [6]	<10 [6]
	Active In	22,424	15 (0.1)	<10 [6]	<10 [6]	<10 [6]
	Active Out	625	<10 [6]	<10 [6]	<10 [6]	<10 [6]
	Convicted Sentence	1,361	<10 [6]	<10 [6]	<10 [6]	<10 [6]
	Downgrade in security category	<10 [6]	<10 [6]	<10 [6]	<10 [6]	<10 [6]
	Internal Cell Move	<10 [6]	<10 [6]	<10 [6]	<10 [6]	<10 [6]
	Judges Remand	19	<10 [6]	<10 [6]	<10 [6]	<10 [6]
	Licence Revoke	178	<10 [6]	<10 [6]	<10 [6]	<10 [6]
	On Remand	1,031	<10 [6]	<10 [6]	<10 [6]	<10 [6]
	Transfer	101	<10 [6]	<10 [6]	<10 [6]	<10 [6]
	Upgrade in security category	<10 [6]	<10 [6]	<10 [6]	<10 [6]	<10 [6]
**Age - years**						
2017/18	10 - <20	468	<10 [6]	<10 [6]	<10 [6]	<10 [6]
	20 - <30	6,994	<10 [6]	<10 [6]	<10 [6]	<10 [6]
	30 - <40	7,051	<10 [6]	<10 [6]	<10 [6]	<10 [6]
	40 - <50	4,114	<10 [6]	<10 [6]	<10 [6]	<10 [6]
	50 - <60	2,107	<10 [6]	<10 [6]	<10 [6]	<10 [6]
	60 - <70	684	<10 [6]	<10 [6]	<10 [6]	<10 [6]
	70 - <80	213	<10 [6]	<10 [6]	<10 [6]	<10 [6]
	80 - <90	40	<10 [6]	<10 [6]	<10 [6]	<10 [6]
	90 - <100	<10 [6]	<10 [6]	<10 [6]	<10 [6]	<10 [6]
	100 - <110	<10 [6]	<10 [6]	<10 [6]	<10 [6]	<10 [6]
2018/19	10 - <20	436	<10 [6]	<10 [6]	<10 [6]	<10 [6]
	20 - <30	7,163	<10 [6]	<10 [6]	<10 [6]	<10 [6]
	30 - <40	7,381	<10 [6]	<10 [6]	<10 [6]	<10 [6]
	40 - <50	4,180	<10 [6]	<10 [6]	<10 [6]	<10 [6]
	50 - <60	1,978	<10 [6]	<10 [6]	<10 [6]	<10 [6]
	60 - <70	701	<10 [6]	<10 [6]	<10 [6]	<10 [6]
	70 - <80	209	<10 [6]	<10 [6]	<10 [6]	<10 [6]
	80 - <90	45	10 (22.2)	<10 [6]	<10 [6]	<10 [6]
	90 - <100	<10 [6]	<10 [6]	<10 [6]	<10 [6]	<10 [6]
	100 - <110	<10 [6]	<10 [6]	<10 [6]	<10 [6]	<10 [6]
2019/20	10 - <20	404	<10 [6]	<10 [6]	<10 [6]	<10 [6]
	20 - <30	8,064	<10 [6]	<10 [6]	<10 [6]	<10 [6]
	30 - <40	9,125	<10 [6]	<10 [6]	<10 [6]	<10 [6]
	40 - <50	4,948	<10 [6]	<10 [6]	<10 [6]	<10 [6]
	50 - <60	2,224	<10 [6]	<10 [6]	<10 [6]	<10 [6]
	60 - <70	751	<10 [6]	<10 [6]	<10 [6]	<10 [6]
	70 - <80	238	<10 [6]	<10 [6]	<10 [6]	<10 [6]
	80 - <90	53	18 (34.0)	<10 [6]	<10 [6]	<10 [6]
	90 - <100	<10 [6]	<10 [6]	<10 [6]	<10 [6]	<10 [6]
	100 - <110	<10 [6]	<10 [6]	<10 [6]	<10 [6]	<10 [6]

[1]Eligible for indicator; [2]Achieve in current prison; [3]Achieve in previous prison; [4]Overall achievement - either current or previous prison; [5]Declined indicator; [6]Suppressed (<10) to avoid disclosure

Indicator	*The proportion of people aged 80 years or over, with coronary heart disease, hypertension, stroke, transient ischaemic attack or peripheral arterial disease, who have had a blood pressure of 150/90mmHg or less in the preceding 12 months*					*Community achievement 2019/20: CHD: 86.5%, Hypertension: 81.97%, Stoke/TIA: 84.9%, PAD not recorded*
	**Group: Cardiovascular Disease**					
	**Variable**	**Population**	**Eligible[1] (% popln)**	**Satisfy[2] (% eligible)**	**Elsewhere[3] (% eligible)**	**Achieve[4] (% eligible)**
**Length of Stay (months)**						
2017/18	<1	4,474	<10 [6]	<10 [6]	<10 [6]	<10 [6]
	1-<6	8,075	<10 [6]	<10 [6]	<10 [6]	<10 [6]
	6-<12	3,672	<10 [6]	<10 [6]	<10 [6]	<10 [6]
	12-<24	2,832	<10 [6]	<10 [6]	<10 [6]	<10 [6]
	24+	2,624	<10 [6]	<10 [6]	<10 [6]	<10 [6]
2018/19	<1	4,801	<10 [6]	<10 [6]	<10 [6]	<10 [6]
	1-<6	7,742	<10 [6]	<10 [6]	<10 [6]	<10 [6]
	6-<12	3,616	<10 [6]	<10 [6]	<10 [6]	<10 [6]
	12-<24	3,447	<10 [6]	<10 [6]	<10 [6]	<10 [6]
	24+	2,493	<10 [6]	<10 [6]	<10 [6]	<10 [6]
2019/20	<1	5,745	<10 [6]	<10 [6]	<10 [6]	<10 [6]
	1-<6	9,697	<10 [6]	<10 [6]	<10 [6]	<10 [6]
	6-<12	5,090	<10 [6]	<10 [6]	<10 [6]	<10 [6]
	12-<24	3,244	<10 [6]	<10 [6]	<10 [6]	<10 [6]
	24+	2,035	<10 [6]	<10 [6]	<10 [6]	<10 [6]
**Ethnic Group**						
2017/18	White	15,638	<10 [6]	<10 [6]	<10 [6]	<10 [6]
	Mixed	431	<10 [6]	<10 [6]	<10 [6]	<10 [6]
	Asian or Asian British	813	<10 [6]	<10 [6]	<10 [6]	<10 [6]
	Black or Black British	404	<10 [6]	<10 [6]	<10 [6]	<10 [6]
	Chinese and Other	214	<10 [6]	<10 [6]	<10 [6]	<10 [6]
	Unclassified	372	<10 [6]	<10 [6]	<10 [6]	<10 [6]
2018/19	White	14,911	10 (0.1)	<10 [6]	<10 [6]	<10 [6]
	Mixed	371	<10 [6]	<10 [6]	<10 [6]	<10 [6]
	Asian or Asian British	726	<10 [6]	<10 [6]	<10 [6]	<10 [6]
	Black or Black British	364	<10 [6]	<10 [6]	<10 [6]	<10 [6]
	Chinese and Other	167	<10 [6]	<10 [6]	<10 [6]	<10 [6]
	Unclassified	409	<10 [6]	<10 [6]	<10 [6]	<10 [6]
2019/20	White	16,606	16 (0.1)	<10 [6]	<10 [6]	<10 [6]
	Mixed	409	<10 [6]	<10 [6]	<10 [6]	<10 [6]
	Asian or Asian British	755	<10 [6]	<10 [6]	<10 [6]	<10 [6]
	Black or Black British	451	<10 [6]	<10 [6]	<10 [6]	<10 [6]
	Chinese and Other	163	<10 [6]	<10 [6]	<10 [6]	<10 [6]
	Unclassified	387	<10 [6]	<10 [6]	<10 [6]	<10 [6]

[1]Eligible for indicator; [2]Achieve in current prison; [3]Achieve in previous prison; [4]Overall achievement - either current or previous prison; [5]Declined indicator; [6]Suppressed (<10) to avoid disclosure

Indicator	The proportion of people who have had a myocardial infarction and are have received a prescription for an ACE-inhibitor or angiotensin receptor blocker, anti-platelet therapy, beta-blocker and a statin in the preceding 12 months<br>Group: Cardiovascular Disease	no comparable data in QOF 2019/20 data. Latest was QOF 2014/15 in which achievement was 70.57%

	Variable	Population	Eligible[1] (% popln)	Satisfy All[2] (% eligible)	Satisfy ACEI[2] (% eligible)	Satisfy ARB[2] (% eligible)	Satisfy Anti Platelet[2] (% eligible)	Satisfy Beta Blocker[2] (% eligible)	Satisfy Statin[2] (% eligible)
**Year**									
	2017/18	21,677	343 (1.6)	<10 [6]	130 (37.9)	<10 [6]	202 (58.9)	192 (56.0)	254 (74.1)
	2018/19	22,099	429 (1.9)	<10 [6]	172 (40.1)	<10 [6]	254 (59.2)	240 (55.9)	292 (68.1)
	2019/20	25,811	492 (1.9)	<10 [6]	207 (42.1)	<10 [6]	300 (61.0)	280 (56.9)	346 (70.3)
**Prison**									
2017/18	Prison 1	1,323	<10 [6]	<10 [6]	<10 [6]	<10 [6]	<10 [6]	<10 [6]	<10 [6]
	Prison 2	3,261	47 (1.4)	<10 [6]	15 (31.9)	<10 [6]	25 (53.2)	19 (40.4)	24 (51.1)
	Prison 3	2,623	35 (1.3)	<10 [6]	<10 [6]	<10 [6]	20 (57.1)	22 (62.9)	27 (77.1)
	Prison 4	2,089	42 (2.0)	<10 [6]	18 (42.9)	<10 [6]	32 (76.2)	28 (66.7)	33 (78.6)
	Prison 5	637	<10 [6]	<10 [6]	<10 [6]	<10 [6]	<10 [6]	<10 [6]	<10 [6]
	Prison 6	1,552	33 (2.1)	<10 [6]	15 (45.5)	<10 [6]	<10 [6]	17 (51.5)	23 (69.7)
	Prison 7	635	<10 [6]	<10 [6]	<10 [6]	<10 [6]	<10 [6]	<10 [6]	<10 [6]
	Prison 8	1,085	12 (1.1)	<10 [6]	<10 [6]	<10 [6]	<10 [6]	<10 [6]	10 (83.3)
	Prison 9	981	52 (5.3)	<10 [6]	23 (44.2)	<10 [6]	40 (76.9)	28 (53.8)	44 (84.6)
	Prison 10	2,523	16 (0.6)	<10 [6]	12 (75.0)	<10 [6]	11 (68.8)	11 (68.8)	14 (87.5)
	Prison 11	3,470	50 (1.4)	<10 [6]	15 (30.0)	<10 [6]	27 (54.0)	31 (62.0)	35 (70.0)
	Prison 12	815	<10 [6]	<10 [6]	<10 [6]	<10 [6]	<10 [6]	<10 [6]	<10 [6]
	Prison 13	683	34 (5.0)	<10 [6]	15 (44.1)	<10 [6]	26 (76.5)	19 (55.9)	28 (82.4)
2018/19	Prison 1	1,333	10 (0.8)	<10 [6]	<10 [6]	<10 [6]	<10 [6]	<10 [6]	<10 [6]
	Prison 2	2,705	42 (1.6)	<10 [6]	22 (52.4)	<10 [6]	29 (69.0)	24 (57.1)	28 (66.7)
	Prison 3	2,522	32 (1.3)	<10 [6]	<10 [6]	<10 [6]	20 (62.5)	19 (59.4)	24 (75.0)
	Prison 4	2,349	57 (2.4)	<10 [6]	26 (45.6)	<10 [6]	37 (64.9)	34 (59.6)	45 (78.9)
	Prison 5	676	14 (2.1)	<10 [6]	<10 [6]	<10 [6]	<10 [6]	<10 [6]	<10 [6]
	Prison 6	1,513	32 (2.1)	<10 [6]	12 (37.5)	<10 [6]	19 (59.4)	16 (50.0)	21 (65.6)
	Prison 7	654	<10 [6]	<10 [6]	<10 [6]	<10 [6]	<10 [6]	<10 [6]	<10 [6]
	Prison 8	1,148	23 (2.0)	<10 [6]	<10 [6]	<10 [6]	11 (47.8)	<10 [6]	12 (52.2)
	Prison 9	996	54 (5.4)	<10 [6]	26 (48.1)	<10 [6]	41 (75.9)	34 (63.0)	46 (85.2)
	Prison 10	2,717	55 (2.0)	<10 [6]	21 (38.2)	<10 [6]	26 (47.3)	28 (50.9)	30 (54.5)
	Prison 11	4,020	65 (1.6)	<10 [6]	21 (32.3)	<10 [6]	30 (46.2)	36 (55.4)	38 (58.5)
	Prison 12	792	<10 [6]	<10 [6]	<10 [6]	<10 [6]	<10 [6]	<10 [6]	<10 [6]
	Prison 13	674	37 (5.5)	<10 [6]	18 (48.6)	<10 [6]	23 (62.2)	24 (64.9)	30 (81.1)
2019/20	Prison 1	1,410	22 (1.6)	<10 [6]	<10 [6]	<10 [6]	12 (54.5)	11 (50.0)	12 (54.5)
	Prison 2	2,979	48 (1.6)	<10 [6]	24 (50.0)	<10 [6]	37 (77.1)	29 (60.4)	39 (81.3)
	Prison 3	2,809	40 (1.4)	<10 [6]	17 (42.5)	<10 [6]	20 (50.0)	21 (52.5)	29 (72.5)
	Prison 4	2,651	80 (3.0)	<10 [6]	44 (55.0)	<10 [6]	58 (72.5)	54 (67.5)	65 (81.3)
	Prison 5	616	10 (1.6)	<10 [6]	<10 [6]	<10 [6]	<10 [6]	<10 [6]	<10 [6]
	Prison 6	1,533	29 (1.9)	<10 [6]	12 (41.4)	<10 [6]	17 (58.6)	13 (44.8)	23 (79.3)
	Prison 7	860	12 (1.4)	<10 [6]	<10 [6]	<10 [6]	<10 [6]	<10 [6]	<10 [6]
	Prison 8	1,385	20 (1.4)	<10 [6]	<10 [6]	<10 [6]	10 (50.0)	<10 [6]	<10 [6]
	Prison 9	1,092	65 (6.0)	<10 [6]	33 (50.8)	<10 [6]	44 (67.7)	41 (63.1)	51 (78.5)
	Prison 10	3,577	58 (1.6)	<10 [6]	21 (36.2)	<10 [6]	26 (44.8)	32 (55.2)	31 (53.4)
	Prison 11	5,348	71 (1.3)	<10 [6]	31 (43.7)	<10 [6]	44 (62.0)	39 (54.9)	48 (67.6)
	Prison 12	805	<10 [6]	<10 [6]	<10 [6]	<10 [6]	<10 [6]	<10 [6]	<10 [6]
	Prison 13	746	37 (5.0)	<10 [6]	13 (35.1)	<10 [6]	22 (59.5)	24 (64.9)	29 (78.4)
**Prison category**									
2017/18	A	1,664	86 (5.2)	<10 [6]	38 (44.2)	<10 [6]	66 (76.7)	47 (54.7)	72 (83.7)
	B	9,254	113 (1.2)	<10 [6]	42 (37.2)	<10 [6]	63 (55.8)	61 (54.0)	73 (64.6)
	C	6,035	85 (1.4)	<10 [6]	26 (30.6)	<10 [6]	57 (67.1)	55 (64.7)	66 (77.6)
	Closed	1,720	18 (1.0)	<10 [6]	<10 [6]	<10 [6]	<10 [6]	<10 [6]	13 (72.2)
	D	2,189	41 (1.9)	<10 [6]	17 (41.5)	<10 [6]	<10 [6]	23 (56.1)	30 (73.2)
	YOI	815	<10 [6]	<10 [6]	<10 [6]	<10 [6]	<10 [6]	<10 [6]	<10 [6]
2018/19	A	1,670	91 (5.4)	<10 [6]	44 (48.4)	<10 [6]	64 (70.3)	58 (63.7)	76 (83.5)
	B	9,442	162 (1.7)	<10 [6]	64 (39.5)	<10 [6]	85 (52.5)	88 (54.3)	96 (59.3)
	C	6,204	99 (1.6)	<10 [6]	39 (39.4)	<10 [6]	63 (63.6)	57 (57.6)	75 (75.8)
	Closed	1,802	29 (1.6)	<10 [6]	<10 [6]	<10 [6]	14 (48.3)	11 (37.9)	15 (51.7)
	D	2,189	46 (2.1)	<10 [6]	16 (34.8)	<10 [6]	27 (58.7)	25 (54.3)	30 (65.2)
	YOI	792	<10 [6]	<10 [6]	<10 [6]	<10 [6]	<10 [6]	<10 [6]	<10 [6]
2019/20	A	1,838	102 (5.5)	<10 [6]	46 (45.1)	<10 [6]	66 (64.7)	65 (63.7)	80 (78.4)
	B	11,904	177 (1.5)	<10 [6]	76 (42.9)	<10 [6]	107 (60.5)	100 (56.5)	118 (66.7)
	C	6,870	142 (2.1)	<10 [6]	61 (43.0)	<10 [6]	90 (63.4)	86 (60.6)	106 (74.6)
	Closed	2,245	32 (1.4)	<10 [6]	<10 [6]	<10 [6]	15 (46.9)	10 (31.3)	13 (40.6)
	D	2,149	39 (1.8)	<10 [6]	16 (41.0)	<10 [6]	22 (56.4)	19 (48.7)	29 (74.4)
	YOI	805	<10 [6]	<10 [6]	<10 [6]	<10 [6]	<10 [6]	<10 [6]	<10 [6]
**Gender**									
2017/18	F	1,699	18 (1.1)	<10 [6]	<10 [6]	<10 [6]	<10 [6]	<10 [6]	13 (72.2)
	M	19,977	325 (1.6)	<10 [6]	123 (37.8)	<10 [6]	193 (59.4)	186 (57.2)	241 (74.2)
2018/19	F	1,802	28 (1.6)	<10 [6]	<10 [6]	<10 [6]	13 (46.4)	10 (35.7)	14 (50.0)
	M	20,295	401 (2.0)	<10 [6]	164 (40.9)	<10 [6]	241 (60.1)	230 (57.4)	278 (69.3)
2019/20	F	1,376	20 (1.5)	<10 [6]	<10 [6]	<10 [6]	10 (50.0)	<10 [6]	<10 [6]
	M	23,570	460 (2.0)	<10 [6]	199 (43.3)	<10 [6]	285 (62.0)	270 (58.7)	333 (72.4)

[1]Eligible for indicator; [2]Achieve in current prison; [3]Achieve in previous prison; [4]Overall achievement - either current or previous prison; [5]Declined indicator; [6]Suppressed (<10) to avoid disclosure

Indicator	*The proportion of people who have had a myocardial infarction and are have received a prescription for an ACE-inhibitor or angiotensin receptor blocker, anti-platelet therapy, beta-blocker and a statin in the preceding 12 months*	*no comparable data in QOF 2019/20 data. Latest was QOF 2014/15 in which achievement was 70.57%*
	**Group: Cardiovascular Disease**	

	Variable	Population	Eligible[1] (% popln)	Satisfy All[2] (% eligible)	Satisfy ACEI[2] (% eligible)	Satisfy ARB[2] (% eligible)	Satisfy Anti Platelet[2] (% eligible)	Satisfy Beta Blocker[2] (% eligible)	Satisfy Statin[2] (% eligible)
**Sentence Status**									
2017/18	.	81	<10 [6]	<10 [6]	<10 [6]	<10 [6]	<10 [6]	<10 [6]	<10 [6]
	Absconded	<10 [6]	<10 [6]	<10 [6]	<10 [6]	<10 [6]	<10 [6]	<10 [6]	<10 [6]
	Active In	15,872	267 (1.7)	<10 [6]	107 (40.1)	<10 [6]	168 (62.9)	157 (58.8)	211 (79.0)
	Active Out	1,052	13 (1.2)	<10 [6]	<10 [6]	<10 [6]	<10 [6]	<10 [6]	<10 [6]
	Convicted Sentence	2,125	27 (1.3)	<10 [6]	<10 [6]	<10 [6]	16 (59.3)	14 (51.9)	17 (63.0)
	Downgrade in security category	<10 [6]	<10 [6]	<10 [6]	<10 [6]	<10 [6]	<10 [6]	<10 [6]	<10 [6]
	Internal Cell Move	<10 [6]	<10 [6]	<10 [6]	<10 [6]	<10 [6]	<10 [6]	<10 [6]	<10 [6]
	Judges Remand	92	<10 [6]	<10 [6]	<10 [6]	<10 [6]	<10 [6]	<10 [6]	<10 [6]
	Licence Revoke	52	<10 [6]	<10 [6]	<10 [6]	<10 [6]	<10 [6]	<10 [6]	<10 [6]
	On Remand	1,492	17 (1.1)	<10 [6]	<10 [6]	<10 [6]	11 (64.7)	<10 [6]	<10 [6]
	Transfer	910	16 (1.8)	<10 [6]	<10 [6]	<10 [6]	<10 [6]	<10 [6]	11 (68.8)
	Upgrade in security category	<10 [6]	<10 [6]	<10 [6]	<10 [6]	<10 [6]	<10 [6]	<10 [6]	<10 [6]
2018/19	.	88	<10 [6]	<10 [6]	<10 [6]	<10 [6]	<10 [6]	<10 [6]	<10 [6]
	Absconded	<10 [6]	<10 [6]	<10 [6]	<10 [6]	<10 [6]	<10 [6]	<10 [6]	<10 [6]
	Active In	18,145	363 (2.0)	<10 [6]	144 (39.7)	<10 [6]	220 (60.6)	205 (56.5)	255 (70.2)
	Active Out	835	18 (2.2)	<10 [6]	<10 [6]	<10 [6]	<10 [6]	<10 [6]	<10 [6]
	Convicted Sentence	1,320	21 (1.6)	<10 [6]	10 (47.6)	<10 [6]	14 (66.7)	14 (66.7)	14 (66.7)
	Downgrade in security category	<10 [6]	<10 [6]	<10 [6]	<10 [6]	<10 [6]	<10 [6]	<10 [6]	<10 [6]
	Internal Cell Move	<10 [6]	<10 [6]	<10 [6]	<10 [6]	<10 [6]	<10 [6]	<10 [6]	<10 [6]
	Judges Remand	<10 [6]	<10 [6]	<10 [6]	<10 [6]	<10 [6]	<10 [6]	<10 [6]	<10 [6]
	Licence Revoke	125	<10 [6]	<10 [6]	<10 [6]	<10 [6]	<10 [6]	<10 [6]	<10 [6]
	On Remand	1,059	15 (1.4)	<10 [6]	<10 [6]	<10 [6]	<10 [6]	<10 [6]	<10 [6]
	Transfer	518	<10 [6]	<10 [6]	<10 [6]	<10 [6]	<10 [6]	<10 [6]	<10 [6]
	Upgrade in security category	<10 [6]	<10 [6]	<10 [6]	<10 [6]	<10 [6]	<10 [6]	<10 [6]	<10 [6]
2019/20	.	69	<10 [6]	<10 [6]	<10 [6]	<10 [6]	<10 [6]	<10 [6]	<10 [6]
	Absconded	<10 [6]	<10 [6]	<10 [6]	<10 [6]	<10 [6]	<10 [6]	<10 [6]	<10 [6]
	Active In	22,424	433 (1.9)	<10 [6]	185 (42.7)	<10 [6]	263 (60.7)	246 (56.8)	304 (70.2)
	Active Out	625	<10 [6]	<10 [6]	<10 [6]	<10 [6]	<10 [6]	<10 [6]	<10 [6]
	Convicted Sentence	1,361	26 (1.9)	<10 [6]	<10 [6]	<10 [6]	16 (61.5)	15 (57.7)	17 (65.4)
	Downgrade in security category	<10 [6]	<10 [6]	<10 [6]	<10 [6]	<10 [6]	<10 [6]	<10 [6]	<10 [6]
	Internal Cell Move	<10 [6]	<10 [6]	<10 [6]	<10 [6]	<10 [6]	<10 [6]	<10 [6]	<10 [6]
	Judges Remand	19	<10 [6]	<10 [6]	<10 [6]	<10 [6]	<10 [6]	<10 [6]	<10 [6]
	Licence Revoke	178	<10 [6]	<10 [6]	<10 [6]	<10 [6]	<10 [6]	<10 [6]	<10 [6]
	On Remand	1,031	16 (1.6)	<10 [6]	<10 [6]	<10 [6]	13 (81.3)	<10 [6]	13 (81.3)
	Transfer	101	<10 [6]	<10 [6]	<10 [6]	<10 [6]	<10 [6]	<10 [6]	<10 [6]
	Upgrade in security category	<10 [6]	<10 [6]	<10 [6]	<10 [6]	<10 [6]	<10 [6]	<10 [6]	<10 [6]
**Age - years**									
2017/18	10 - <20	468	<10 [6]	<10 [6]	<10 [6]	<10 [6]	<10 [6]	<10 [6]	<10 [6]
	20 - <30	6,994	<10 [6]	<10 [6]	<10 [6]	<10 [6]	<10 [6]	<10 [6]	<10 [6]
	30 - <40	7,051	18 (0.3)	<10 [6]	<10 [6]	<10 [6]	<10 [6]	<10 [6]	<10 [6]
	40 - <50	4,114	54 (1.3)	<10 [6]	13 (24.1)	<10 [6]	27 (50.0)	25 (46.3)	34 (63.0)
	50 - <60	2,107	123 (5.8)	<10 [6]	58 (47.2)	<10 [6]	76 (61.8)	75 (61.0)	95 (77.2)
	60 - <70	684	96 (14.0)	<10 [6]	34 (35.4)	<10 [6]	63 (65.6)	54 (56.3)	76 (79.2)
	70 - <80	213	41 (19.2)	<10 [6]	15 (36.6)	<10 [6]	26 (63.4)	25 (61.0)	35 (85.4)
	80 - <90	40	<10 [6]	<10 [6]	<10 [6]	<10 [6]	<10 [6]	<10 [6]	<10 [6]
	90 - <100	<10 [6]	<10 [6]	<10 [6]	<10 [6]	<10 [6]	<10 [6]	<10 [6]	<10 [6]
	100 - <110	<10 [6]	<10 [6]	<10 [6]	<10 [6]	<10 [6]	<10 [6]	<10 [6]	<10 [6]
2018/19	10 - <20	436	<10 [6]	<10 [6]	<10 [6]	<10 [6]	<10 [6]	<10 [6]	<10 [6]
	20 - <30	7,163	11 (0.2)	<10 [6]	<10 [6]	<10 [6]	<10 [6]	<10 [6]	<10 [6]
	30 - <40	7,381	38 (0.5)	<10 [6]	<10 [6]	<10 [6]	<10 [6]	11 (28.9)	<10 [6]
	40 - <50	4,180	69 (1.7)	<10 [6]	26 (37.7)	<10 [6]	36 (52.2)	35 (50.7)	39 (56.5)
	50 - <60	1,978	149 (7.5)	<10 [6]	66 (44.3)	<10 [6]	104 (69.8)	90 (60.4)	114 (76.5)
	60 - <70	701	109 (15.5)	<10 [6]	53 (48.6)	<10 [6]	75 (68.8)	64 (58.7)	85 (78.0)
	70 - <80	209	40 (19.1)	<10 [6]	18 (45.0)	<10 [6]	23 (57.5)	27 (67.5)	35 (87.5)
	80 - <90	45	13 (28.9)	<10 [6]	<10 [6]	<10 [6]	<10 [6]	<10 [6]	11 (84.6)
	90 - <100	<10 [6]	<10 [6]	<10 [6]	<10 [6]	<10 [6]	<10 [6]	<10 [6]	<10 [6]
	100 - <110	<10 [6]	<10 [6]	<10 [6]	<10 [6]	<10 [6]	<10 [6]	<10 [6]	<10 [6]
2019/20	10 - <20	404	<10 [6]	<10 [6]	<10 [6]	<10 [6]	<10 [6]	<10 [6]	<10 [6]
	20 - <30	8,064	<10 [6]	<10 [6]	<10 [6]	<10 [6]	<10 [6]	<10 [6]	<10 [6]
	30 - <40	9,125	37 (0.4)	<10 [6]	<10 [6]	<10 [6]	<10 [6]	12 (32.4)	<10 [6]
	40 - <50	4,948	84 (1.7)	<10 [6]	30 (35.7)	<10 [6]	40 (47.6)	41 (48.8)	52 (61.9)
	50 - <60	2,224	163 (7.3)	<10 [6]	74 (45.4)	<10 [6]	110 (67.5)	96 (58.9)	125 (76.7)
	60 - <70	751	137 (18.2)	<10 [6]	66 (48.2)	<10 [6]	101 (73.7)	91 (66.4)	115 (83.9)
	70 - <80	238	48 (20.2)	<10 [6]	22 (45.8)	<10 [6]	31 (64.6)	29 (60.4)	35 (72.9)
	80 - <90	53	13 (24.5)	<10 [6]	<10 [6]	<10 [6]	<10 [6]	<10 [6]	<10 [6]
	90 - <100	<10 [6]	<10 [6]	<10 [6]	<10 [6]	<10 [6]	<10 [6]	<10 [6]	<10 [6]
	100 - <110	<10 [6]	<10 [6]	<10 [6]	<10 [6]	<10 [6]	<10 [6]	<10 [6]	<10 [6]

[1]Eligible for indicator; [2]Achieve in current prison; [3]Achieve in previous prison; [4]Overall achievement - either current or previous prison; [5]Declined indicator; [6]Suppressed (<10) to avoid disclosure

Indicator	The proportion of people who have had a myocardial infarction and are have received a prescription for an ACE-inhibitor or angiotensin receptor blocker, anti-platelet therapy, beta-blocker and a statin in the preceding 12 months	*no comparable data in QOF 2019/20 data. Latest was QOF 2014/15 in which achievement was 70.57%*
	**Group: Cardiovascular Disease**	

	Variable	Population	Eligible[1] (% popln)	Satisfy All[2] (% eligible)	Satisfy ACEI[2] (% eligible)	Satisfy ARB[2] (% eligible)	Satisfy Anti Platelet[2] (% eligible)	Satisfy Beta Blocker[2] (% eligible)	Satisfy Statin[2] (% eligible)
**Length of Stay (months)**									
2017/18	<1	4,474	37 (0.8)	<10 [6]	<10 [6]	<10 [6]	14 (37.8)	12 (32.4)	18 (48.6)
	1-<6	8,075	78 (1.0)	<10 [6]	30 (38.5)	<10 [6]	43 (55.1)	51 (65.4)	55 (70.5)
	6-<12	3,672	54 (1.5)	<10 [6]	22 (40.7)	<10 [6]	35 (64.8)	37 (68.5)	43 (79.6)
	12-<24	2,832	50 (1.8)	<10 [6]	16 (32.0)	<10 [6]	20 (40.0)	26 (52.0)	36 (72.0)
	24+	2,624	124 (4.7)	<10 [6]	53 (42.7)	<10 [6]	90 (72.6)	66 (53.2)	102 (82.3)
2018/19	<1	4,801	60 (1.2)	<10 [6]	15 (25.0)	<10 [6]	23 (38.3)	29 (48.3)	29 (48.3)
	1-<6	7,742	107 (1.4)	<10 [6]	40 (37.4)	<10 [6]	64 (59.8)	58 (54.2)	65 (60.7)
	6-<12	3,616	60 (1.7)	<10 [6]	20 (33.3)	<10 [6]	34 (56.7)	33 (55.0)	38 (63.3)
	12-<24	3,447	68 (2.0)	<10 [6]	32 (47.1)	<10 [6]	38 (55.9)	43 (63.2)	46 (67.6)
	24+	2,493	134 (5.4)	<10 [6]	65 (48.5)	<10 [6]	95 (70.9)	77 (57.5)	114 (85.1)
2019/20	<1	5,745	69 (1.2)	<10 [6]	20 (29.0)	<10 [6]	33 (47.8)	30 (43.5)	40 (58.0)
	1-<6	9,697	117 (1.2)	<10 [6]	49 (41.9)	<10 [6]	66 (56.4)	66 (56.4)	72 (61.5)
	6-<12	5,090	97 (1.9)	<10 [6]	36 (37.1)	<10 [6]	56 (57.7)	54 (55.7)	70 (72.2)
	12-<24	3,244	83 (2.6)	<10 [6]	42 (50.6)	<10 [6]	54 (65.1)	49 (59.0)	57 (68.7)
	24+	2,035	126 (6.2)	<10 [6]	60 (47.6)	<10 [6]	91 (72.2)	81 (64.3)	107 (84.9)
**Ethnic Group**									
2017/18	White	15,638	290 (1.9)	<10 [6]	104 (35.9)	<10 [6]	174 (60.0)	161 (55.5)	213 (73.4)
	Mixed	431	<10 [6]	<10 [6]	<10 [6]	<10 [6]	<10 [6]	<10 [6]	<10 [6]
	Asian or Asian British	813	<10 [6]	<10 [6]	<10 [6]	<10 [6]	<10 [6]	<10 [6]	<10 [6]
	Black or Black British	404	<10 [6]	<10 [6]	<10 [6]	<10 [6]	<10 [6]	<10 [6]	<10 [6]
	Chinese and Other	214	<10 [6]	<10 [6]	<10 [6]	<10 [6]	<10 [6]	<10 [6]	<10 [6]
	Unclassified	372	12 (3.2)	<10 [6]	<10 [6]	<10 [6]	<10 [6]	<10 [6]	<10 [6]
2018/19	White	14,911	341 (2.3)	<10 [6]	137 (40.2)	<10 [6]	207 (60.7)	187 (54.8)	233 (68.3)
	Mixed	371	<10 [6]	<10 [6]	<10 [6]	<10 [6]	<10 [6]	<10 [6]	<10 [6]
	Asian or Asian British	726	13 (1.8)	<10 [6]	<10 [6]	<10 [6]	<10 [6]	<10 [6]	<10 [6]
	Black or Black British	364	<10 [6]	<10 [6]	<10 [6]	<10 [6]	<10 [6]	<10 [6]	<10 [6]
	Chinese and Other	167	<10 [6]	<10 [6]	<10 [6]	<10 [6]	<10 [6]	<10 [6]	<10 [6]
	Unclassified	409	<10 [6]	<10 [6]	<10 [6]	<10 [6]	<10 [6]	<10 [6]	<10 [6]
2019/20	White	16,606	376 (2.3)	<10 [6]	159 (42.3)	<10 [6]	235 (62.5)	211 (56.1)	263 (69.9)
	Mixed	409	<10 [6]	<10 [6]	<10 [6]	<10 [6]	<10 [6]	<10 [6]	<10 [6]
	Asian or Asian British	755	21 (2.8)	<10 [6]	<10 [6]	<10 [6]	12 (57.1)	11 (52.4)	16 (76.2)
	Black or Black British	451	<10 [6]	<10 [6]	<10 [6]	<10 [6]	<10 [6]	<10 [6]	<10 [6]
	Chinese and Other	163	<10 [6]	<10 [6]	<10 [6]	<10 [6]	<10 [6]	<10 [6]	<10 [6]
	Unclassified	387	<10 [6]	<10 [6]	<10 [6]	<10 [6]	<10 [6]	<10 [6]	<10 [6]

[1]Eligible for indicator; [2]Achieve in current prison; [3]Achieve in previous prison; [4]Overall achievement - either current or previous prison; [5]Declined indicator; [6]Suppressed (<10) to avoid disclosure

Indicator	*The proportion of people who have had a myocardial infarction and are have received a prescription for an ACE-inhibitor or angiotensin receptor blocker, anti-platelet therapy, beta-blocker and a statin in the preceding 12 months*<br>Group: Cardiovascular Disease		*Community achievement 2019/20 (%): no comparable data in QOF 2019/20 data. Latest was QOF 2014/15 in which achievement was 70.57%*	
	**Variable**	**Population**	**Eligible[1] (% population)**	**Satisfy 3 out of the 4 classes of drugs (% eligible)**
**Year**				
	2017/18	21,677	343 (1.6)	175 (51.0)
	2018/19	22,099	429 (1.9)	223 (52.0)
	2019/20	25,811	492 (1.9)	256 (52.0)
**Prison**				
2017/18	Prison 1	1,323	<10 [6]	<10 [6]
	Prison 2	3,261	47 (1.4)	20 (42.6)
	Prison 3	2,623	35 (1.3)	16 (45.7)
	Prison 4	2,089	42 (2.0)	25 (59.5)
	Prison 5	637	<10 [6]	<10 [6]
	Prison 6	1,552	33 (2.1)	12 (36.4)
	Prison 7	635	<10 [6]	<10 [6]
	Prison 8	1,085	12 (1.1)	<10 [6]
	Prison 9	981	52 (5.3)	32 (61.5)
	Prison 10	2,523	16 (0.6)	11 (68.8)
	Prison 11	3,470	50 (1.4)	22 (44.0)
	Prison 12	815	<10 [6]	<10 [6]
	Prison 13	683	34 (5.0)	21 (61.8)
2018/19	Prison 1	1,333	10 (0.8)	<10 [6]
	Prison 2	2,705	42 (1.6)	25 (59.5)
	Prison 3	2,522	32 (1.3)	17 (53.1)
	Prison 4	2,349	57 (2.4)	31 (54.4)
	Prison 5	676	14 (2.1)	<10 [6]
	Prison 6	1,513	32 (2.1)	15 (46.9)
	Prison 7	654	<10 [6]	<10 [6]
	Prison 8	1,148	23 (2.0)	<10 [6]
	Prison 9	996	54 (5.4)	36 (66.7)
	Prison 10	2,717	55 (2.0)	24 (43.6)
	Prison 11	4,020	65 (1.6)	28 (43.1)
	Prison 12	792	<10 [6]	<10 [6]
	Prison 13	674	37 (5.5)	24 (64.9)
2019/20	Prison 1	1,410	22 (1.6)	<10 [6]
	Prison 2	2,979	48 (1.6)	34 (70.8)
	Prison 3	2,809	40 (1.4)	18 (45.0)
	Prison 4	2,651	80 (3.0)	55 (68.8)
	Prison 5	616	10 (1.6)	<10 [6]
	Prison 6	1,533	29 (1.9)	14 (48.3)
	Prison 7	860	12 (1.4)	<10 [6]
	Prison 8	1,385	20 (1.4)	<10 [6]
	Prison 9	1,092	65 (6.0)	38 (58.5)
	Prison 10	3,577	58 (1.6)	24 (41.4)
	Prison 11	5,348	71 (1.3)	34 (47.9)
	Prison 12	805	<10 [6]	<10 [6]
	Prison 13	746	37 (5.0)	20 (54.1)
**Prison category**				
2017/18	A	1,664	86 (5.2)	53 (61.6)
	B	9,254	113 (1.2)	53 (46.9)
	C	6,035	85 (1.4)	45 (52.9)
	Closed	1,720	18 (1.0)	<10 [6]
	D	2,189	41 (1.9)	17 (41.5)
	YOI	815	<10 [6]	<10 [6]
2018/19	A	1,670	<10 [6]	60 (65.9)
	B	9,442	162 (1.7)	77 (47.5)
	C	6,204	99 (1.6)	53 (53.5)
	Closed	1,802	29 (1.6)	11 (37.9)
	D	2,189	46 (2.1)	22 (47.8)
	YOI	792	<10 [6]	<10 [6]
2019/20	A	1,838	102 (5.5)	58 (56.9)
	B	11,904	177 (1.5)	92 (52.0)
	C	6,870	142 (2.1)	79 (55.6)
	Closed	2,245	32 (1.4)	<10 [6]
	D	2,149	39 (1.8)	18 (46.2)
	YOI	805	<10 [6]	<10 [6]
**Gender**				
2017/18	F	1,699	18 (1.1)	<10 [6]
	M	19,977	325 (1.6)	168 (51.7)
2018/19	F	1,802	28 (1.6)	10 (35.7)
	M	20,295	401 (2.0)	213 (53.1)
2019/20	F	1,376	20 (1.5)	<10 [6]
	M	23,570	460 (2.0)	247 (53.7)

[1]Eligible for indicator; [2]Achieve in current prison; [3]Achieve in previous prison; [4]Overall achievement - either current or previous prison; [5]Declined indicator; [6]Suppressed (<10) to avoid disclosure

Indicator	*The proportion of people who have had a myocardial infarction and are have received a prescription for an ACE-inhibitor or angiotensin receptor blocker, anti-platelet therapy, beta-blocker and a statin in the preceding 12 months* **Group: Cardiovascular Disease**		***Community achievement 2019/20 (%): no comparable data in QOF 2019/20 data. Latest was QOF 2014/15 in which achievement was 70.57%***

	Variable	Population	Eligible[1] (% population)	Satisfy 3 out of the 4 classes of drugs (% eligible)
**Sentence Status**				
2017/18	.	81	<10 [6]	<10 [6]
	Absconded	<10 [6]	<10 [6]	<10 [6]
	Active In	15,872	267 (1.7)	143 (53.6)
	Active Out	1,052	13 (1.2)	<10 [6]
	Convicted Sentence	2,125	27 (1.3)	14 (51.9)
	Downgrade in security category	<10 [6]	<10 [6]	<10 [6]
	Internal Cell Move	<10 [6]	<10 [6]	<10 [6]
	Judges Remand	92	<10 [6]	<10 [6]
	Licence Revoke	52	<10 [6]	<10 [6]
	On Remand	1,492	17 (1.1)	<10 [6]
	Transfer	910	16 (1.8)	<10 [6]
	Upgrade in security category	<10 [6]	<10 [6]	<10 [6]
2018/19	.	88	<10 [6]	<10 [6]
	Absconded	<10 [6]	<10 [6]	<10 [6]
	Active In	18,145	363 (2.0)	192 (52.9)
	Active Out	835	18 (2.2)	<10 [6]
	Convicted Sentence	1,320	21 (1.6)	12 (57.1)
	Downgrade in security category	<10 [6]	<10 [6]	<10 [6]
	Internal Cell Move	<10 [6]	<10 [6]	<10 [6]
	Judges Remand	<10 [6]	<10 [6]	<10 [6]
	Licence Revoke	125	<10 [6]	<10 [6]
	On Remand	1,059	15 (1.4)	<10 [6]
	Transfer	518	<10 [6]	<10 [6]
	Upgrade in security category	<10 [6]	<10 [6]	<10 [6]
2019/20	.	69	<10 [6]	<10 [6]
	Absconded	<10 [6]	<10 [6]	<10 [6]
	Active In	22,424	433 (1.9)	223 (51.5)
	Active Out	625	<10 [6]	<10 [6]
	Convicted Sentence	1,361	26 (1.9)	15 (57.7)
	Downgrade in security category	<10 [6]	<10 [6]	<10 [6]
	Internal Cell Move	<10 [6]	<10 [6]	<10 [6]
	Judges Remand	19	<10 [6]	<10 [6]
	Licence Revoke	178	<10 [6]	<10 [6]
	On Remand	1,031	16 (1.6)	<10 [6]
	Transfer	101	<10 [6]	<10 [6]
	Upgrade in security category	<10 [6]	<10 [6]	<10 [6]
**Age - years**				
2017/18	10 - <20	468	<10 [6]	<10 [6]
	20 - <30	6,994	<10 [6]	<10 [6]
	30 - <40	7,051	18 (0.3)	<10 [6]
	40 - <50	4,114	54 (1.3)	22 (40.7)
	50 - <60	2,107	123 (5.8)	71 (57.7)
	60 - <70	684	96 (14.0)	52 (54.2)
	70 - <80	213	41 (19.2)	20 (48.8)
	80 - <90	40	<10 [6]	<10 [6]
	90 - <100	<10 [6]	<10 [6]	<10 [6]
	100 - <110	<10 [6]	<10 [6]	<10 [6]
2018/19	10 - <20	436	<10 [6]	<10 [6]
	20 - <30	7,163	11 (0.2)	<10 [6]
	30 - <40	7,381	38 (0.5)	<10 [6]
	40 - <50	4,180	69 (1.7)	30 (43.5)
	50 - <60	1,978	149 (7.5)	90 (60.4)
	60 - <70	701	109 (15.5)	69 (63.3)
	70 - <80	209	40 (19.1)	22 (55.0)
	80 - <90	45	13 (28.9)	<10 [6]
	90 - <100	<10 [6]	<10 [6]	<10 [6]
	100 - <110	<10 [6]	<10 [6]	<10 [6]
2019/20	10 - <20	404	<10 [6]	<10 [6]
	20 - <30	8,064	<10 [6]	<10 [6]
	30 - <40	9,125	37 (0.4)	<10 [6]
	40 - <50	4,948	84 (1.7)	37 (44.0)
	50 - <60	2,224	163 (7.3)	92 (56.4)
	60 - <70	751	137 (18.2)	88 (64.2)
	70 - <80	238	48 (20.2)	25 (52.1)
	80 - <90	53	13 (24.5)	<10 [6]
	90 - <100	<10 [6]	<10 [6]	<10 [6]
	100 - <110	<10 [6]	<10 [6]	<10 [6]

[1]Eligible for indicator; [2]Achieve in current prison; [3]Achieve in previous prison; [4]Overall achievement - either current or previous prison; [5]Declined indicator; [6]Suppressed (<10) to avoid disclosure

Indicator	*The proportion of people who have had a myocardial infarction and are have received a prescription for an ACE-inhibitor or angiotensin receptor blocker, anti-platelet therapy, beta-blocker and a statin in the preceding 12 months* Group: Cardiovascular Disease		*Community achievement 2019/20 (%): no comparable data in QOF 2019/20 data. Latest was QOF 2014/15 in which achievement was 70.57%*
	**Variable**	**Population**	**Eligible[1] (% population)**	**Satisfy 3 out of the 4 classes of drugs (% eligible)**

	Variable	Population	Eligible[1] (% population)	Satisfy 3 out of the 4 classes of drugs (% eligible)
**Length of Stay (months)**				
2017/18	<1	4,474	37 (0.8)	11 (29.7)
	1-<6	8,075	78 (1.0)	40 (51.3)
	6-<12	3,672	54 (1.5)	30 (55.6)
	12-<24	2,832	50 (1.8)	21 (42.0)
	24+	2,624	124 (4.7)	73 (58.9)
2018/19	<1	4,801	60 (1.2)	20 (33.3)
	1-<6	7,742	107 (1.4)	54 (50.5)
	6-<12	3,616	60 (1.7)	25 (41.7)
	12-<24	3,447	68 (2.0)	37 (54.4)
	24+	2,493	134 (5.4)	87 (64.9)
2019/20	<1	5,745	69 (1.2)	28 (40.6)
	1-<6	9,697	117 (1.2)	57 (48.7)
	6-<12	5,090	97 (1.9)	45 (46.4)
	12-<24	3,244	83 (2.6)	42 (50.6)
	24+	2,035	126 (6.2)	84 (66.7)
**Ethnic Group**				
2017/18	White	15,638	290 (1.9)	145 (50.0)
	Mixed	431	<10 [6]	<10 [6]
	Asian or Asian British	813	<10 [6]	<10 [6]
	Black or Black British	404	<10 [6]	<10 [6]
	Chinese and Other	214	<10 [6]	<10 [6]
	Unclassified	372	12 (3.2)	<10 [6]
2018/19	White	14,911	341 (2.3)	176 (51.6)
	Mixed	371	<10 [6]	<10 [6]
	Asian or Asian British	726	13 (1.8)	<10 [6]
	Black or Black British	364	<10 [6]	<10 [6]
	Chinese and Other	167	<10 [6]	<10 [6]
	Unclassified	409	<10 [6]	<10 [6]
2019/20	White	16,606	376 (2.3)	198 (52.7)
	Mixed	409	<10 [6]	<10 [6]
	Asian or Asian British	755	21 (2.8)	10 (47.6)
	Black or Black British	451	<10 [6]	<10 [6]
	Chinese and Other	163	<10 [6]	<10 [6]
	Unclassified	387	<10 [6]	<10 [6]

[1]Eligible for indicator; [2]Achieve in current prison; [3]Achieve in previous prison; [4]Overall achievement - either current or previous prison; [5]Declined indicator; [6]Suppressed (<10) to avoid disclosure

Indicator *The proportion of people with coronary heart disease who have received a prescription for anti-platelet or anti-coagulant therapy in the preceding 12 months*

Group: Cardiovascular Disease

Community achievement 2019/20: 90.87% (QOF 19/20)

	Variable	Population	Eligible[1] (% popln)	Satisfy[2] (% eligible)	Elsewhere[3] (% eligible)	Achieve[4] (% eligible)
**Year**						
	2017/18	21,677	355 (1.6)	262 (73.8)	<10 [6]	265 (74.6)
	2018/19	22,099	441 (2.0)	302 (68.5)	<10 [6]	308 (69.8)
	2019/20	25,811	494 (1.9)	351 (71.1)	<10 [6]	360 (72.9)
**Prison**						
2017/18	Prison 1	1,323	<10 [6]	<10 [6]	<10 [6]	<10 [6]
	Prison 2	3,261	47 (1.4)	27 (57.4)	<10 [6]	27 (57.4)
	Prison 3	2,623	36 (1.4)	26 (72.2)	<10 [6]	27 (75.0)
	Prison 4	2,089	52 (2.5)	44 (84.6)	<10 [6]	45 (86.5)
	Prison 5	637	<10 [6]	<10 [6]	<10 [6]	<10 [6]
	Prison 6	1,552	33 (2.1)	24 (72.7)	<10 [6]	25 (75.8)
	Prison 7	635	<10 [6]	<10 [6]	<10 [6]	<10 [6]
	Prison 8	1,085	12 (1.1)	<10 [6]	<10 [6]	<10 [6]
	Prison 9	981	52 (5.3)	43 (82.7)	<10 [6]	43 (82.7)
	Prison 10	2,523	16 (0.6)	13 (81.3)	<10 [6]	13 (81.3)
	Prison 11	3,470	50 (1.4)	33 (66.0)	<10 [6]	33 (66.0)
	Prison 12	815	<10 [6]	<10 [6]	<10 [6]	<10 [6]
	Prison 13	683	35 (5.1)	28 (80.0)	<10 [6]	28 (80.0)
2018/19	Prison 1	1,333	10 (0.8)	<10 [6]	<10 [6]	<10 [6]
	Prison 2	2,705	42 (1.6)	31 (73.8)	<10 [6]	31 (73.8)
	Prison 3	2,522	32 (1.3)	24 (75.0)	<10 [6]	27 (84.4)
	Prison 4	2,349	68 (2.9)	55 (80.9)	<10 [6]	56 (82.4)
	Prison 5	676	14 (2.1)	10 (71.4)	<10 [6]	10 (71.4)
	Prison 6	1,513	32 (2.1)	20 (62.5)	<10 [6]	20 (62.5)
	Prison 7	654	<10 [6]	<10 [6]	<10 [6]	<10 [6]
	Prison 8	1,148	23 (2.0)	<10 [6]	<10 [6]	<10 [6]
	Prison 9	996	54 (5.4)	44 (81.5)	<10 [6]	44 (81.5)
	Prison 10	2,717	55 (2.0)	32 (58.2)	<10 [6]	32 (58.2)
	Prison 11	4,020	65 (1.6)	37 (56.9)	<10 [6]	38 (58.5)
	Prison 12	792	<10 [6]	<10 [6]	<10 [6]	<10 [6]
	Prison 13	674	38 (5.6)	30 (78.9)	<10 [6]	31 (81.6)
2019/20	Prison 1	1,410	22 (1.6)	14 (63.6)	<10 [6]	15 (68.2)
	Prison 2	2,979	48 (1.6)	38 (79.2)	<10 [6]	38 (79.2)
	Prison 3	2,809	41 (1.5)	26 (63.4)	<10 [6]	26 (63.4)
	Prison 4	2,651	80 (3.0)	66 (82.5)	<10 [6]	67 (83.8)
	Prison 5	616	10 (1.6)	<10 [6]	<10 [6]	<10 [6]
	Prison 6	1,533	29 (1.9)	21 (72.4)	<10 [6]	21 (72.4)
	Prison 7	860	12 (1.4)	<10 [6]	<10 [6]	<10 [6]
	Prison 8	1,385	20 (1.4)	11 (55.0)	<10 [6]	11 (55.0)
	Prison 9	1,092	65 (6.0)	51 (78.5)	<10 [6]	52 (80.0)
	Prison 10	3,577	58 (1.6)	36 (62.1)	<10 [6]	38 (65.5)
	Prison 11	5,348	71 (1.3)	47 (66.2)	<10 [6]	48 (67.6)
	Prison 12	805	<10 [6]	<10 [6]	<10 [6]	<10 [6]
	Prison 13	746	38 (5.1)	29 (76.3)	<10 [6]	30 (78.9)
**Prison category**						
2017/18	A	1,664	87 (5.2)	71 (81.6)	<10 [6]	71 (81.6)
	B	9,254	113 (1.2)	73 (64.6)	<10 [6]	73 (64.6)
	C	6,035	96 (1.6)	76 (79.2)	<10 [6]	78 (81.3)
	Closed	1,720	18 (1.0)	10 (55.6)	<10 [6]	10 (55.6)
	D	2,189	41 (1.9)	32 (78.0)	<10 [6]	33 (80.5)
	YOI	815	<10 [6]	<10 [6]	<10 [6]	<10 [6]
2018/19	A	1,670	92 (5.5)	74 (80.4)	<10 [6]	75 (81.5)
	B	9,442	162 (1.7)	100 (61.7)	<10 [6]	101 (62.3)
	C	6,204	110 (1.8)	85 (77.3)	<10 [6]	89 (80.9)
	Closed	1,802	29 (1.6)	12 (41.4)	<10 [6]	12 (41.4)
	D	2,189	46 (2.1)	30 (65.2)	<10 [6]	30 (65.2)
	YOI	792	<10 [6]	<10 [6]	<10 [6]	<10 [6]
2019/20	A	1,838	103 (5.6)	80 (77.7)	<10 [6]	82 (79.6)
	B	11,904	177 (1.5)	121 (68.4)	<10 [6]	124 (70.1)
	C	6,870	143 (2.1)	106 (74.1)	<10 [6]	108 (75.5)
	Closed	2,245	32 (1.4)	15 (46.9)	<10 [6]	17 (53.1)
	D	2,149	39 (1.8)	29 (74.4)	<10 [6]	29 (74.4)
	YOI	805	<10 [6]	<10 [6]	<10 [6]	<10 [6]
**Gender**						
2017/18	F	1,699	18 (1.1)	10 (55.6)	<10 [6]	10 (55.6)
	M	19,977	337 (1.7)	252 (74.8)	<10 [6]	255 (75.7)
2018/19	F	1,802	28 (1.6)	12 (42.9)	<10 [6]	12 (42.9)
	M	20,295	413 (2.0)	290 (70.2)	<10 [6]	296 (71.7)
2019/20	F	1,376	20 (1.5)	11 (55.0)	<10 [6]	11 (55.0)
	M	23,570	462 (2.0)	336 (72.7)	<10 [6]	343 (74.2)

[1]Eligible for indicator; [2]Achieve in current prison; [3]Achieve in previous prison; [4]Overall achievement - either current or previous prison; [5]Declined indicator; [6]Suppressed (<10) to avoid disclosure

Indicator	*The proportion of people with coronary heart disease who have received a prescription for anti-platelet or anti-coagulant therapy in the preceding 12 months*						*Community achievement 2019/20: 90.87% (QOF 19/20)*
	**Group: Cardiovascular Disease**						
	**Variable**	**Population**	**Eligible[1] (% popln)**	**Satisfy[2] (% eligible)**	**Elsewhere[3] (% eligible)**	**Achieve[4] (% eligible)**	
**Sentence Status**							
2017/18	.	81	<10 [6]	<10 [6]	<10 [6]	<10 [6]	
	Absconded	<10 [6]	<10 [6]	<10 [6]	<10 [6]	<10 [6]	
	Active In	15,872	279 (1.8)	215 (77.1)	<10 [6]	217 (77.8)	
	Active Out	1,052	13 (1.2)	<10 [6]	<10 [6]	<10 [6]	
	Convicted Sentence	2,125	27 (1.3)	16 (59.3)	<10 [6]	16 (59.3)	
	Downgrade in security category	<10 [6]	<10 [6]	<10 [6]	<10 [6]	<10 [6]	
	Internal Cell Move	<10 [6]	<10 [6]	<10 [6]	<10 [6]	<10 [6]	
	Judges Remand	92	<10 [6]	<10 [6]	<10 [6]	<10 [6]	
	Licence Revoke	52	<10 [6]	<10 [6]	<10 [6]	<10 [6]	
	On Remand	1,492	17 (1.1)	14 (82.4)	<10 [6]	14 (82.4)	
	Transfer	910	16 (1.8)	10 (62.5)	<10 [6]	11 (68.8)	
	Upgrade in security category	<10 [6]	<10 [6]	<10 [6]	<10 [6]	<10 [6]	
2018/19	.	88	<10 [6]	<10 [6]	<10 [6]	<10 [6]	
	Absconded	<10 [6]	<10 [6]	<10 [6]	<10 [6]	<10 [6]	
	Active In	18,145	375 (2.1)	264 (70.4)	<10 [6]	270 (72.0)	
	Active Out	835	18 (2.2)	<10 [6]	<10 [6]	<10 [6]	
	Convicted Sentence	1,320	21 (1.6)	16 (76.2)	<10 [6]	16 (76.2)	
	Downgrade in security category	<10 [6]	<10 [6]	<10 [6]	<10 [6]	<10 [6]	
	Internal Cell Move	<10 [6]	<10 [6]	<10 [6]	<10 [6]	<10 [6]	
	Judges Remand	<10 [6]	<10 [6]	<10 [6]	<10 [6]	<10 [6]	
	Licence Revoke	125	<10 [6]	<10 [6]	<10 [6]	<10 [6]	
	On Remand	1,059	15 (1.4)	11 (73.3)	<10 [6]	11 (73.3)	
	Transfer	518	<10 [6]	<10 [6]	<10 [6]	<10 [6]	
	Upgrade in security category	<10 [6]	<10 [6]	<10 [6]	<10 [6]	<10 [6]	
2019/20	.	69	<10 [6]	<10 [6]	<10 [6]	<10 [6]	
	Absconded	<10 [6]	<10 [6]	<10 [6]	<10 [6]	<10 [6]	
	Active In	22,424	435 (1.9)	311 (71.5)	<10 [6]	320 (73.6)	
	Active Out	625	<10 [6]	<10 [6]	<10 [6]	<10 [6]	
	Convicted Sentence	1,361	26 (1.9)	17 (65.4)	<10 [6]	17 (65.4)	
	Downgrade in security category	<10 [6]	<10 [6]	<10 [6]	<10 [6]	<10 [6]	
	Internal Cell Move	<10 [6]	<10 [6]	<10 [6]	<10 [6]	<10 [6]	
	Judges Remand	19	<10 [6]	<10 [6]	<10 [6]	<10 [6]	
	Licence Revoke	178	<10 [6]	<10 [6]	<10 [6]	<10 [6]	
	On Remand	1,031	16 (1.6)	13 (81.3)	<10 [6]	13 (81.3)	
	Transfer	101	<10 [6]	<10 [6]	<10 [6]	<10 [6]	
	Upgrade in security category	<10 [6]	<10 [6]	<10 [6]	<10 [6]	<10 [6]	
**Age - years**							
2017/18	10 - <20	468	<10 [6]	<10 [6]	<10 [6]	<10 [6]	
	20 - <30	6,994	<10 [6]	<10 [6]	<10 [6]	<10 [6]	
	30 - <40	7,051	18 (0.3)	<10 [6]	<10 [6]	<10 [6]	
	40 - <50	4,114	56 (1.4)	34 (60.7)	<10 [6]	35 (62.5)	
	50 - <60	2,107	127 (6.0)	101 (79.5)	<10 [6]	101 (79.5)	
	60 - <70	684	101 (14.8)	79 (78.2)	<10 [6]	80 (79.2)	
	70 - <80	213	42 (19.7)	35 (83.3)	<10 [6]	36 (85.7)	
	80 - <90	40	<10 [6]	<10 [6]	<10 [6]	<10 [6]	
	90 - <100	<10 [6]	<10 [6]	<10 [6]	<10 [6]	<10 [6]	
	100 - <110	<10 [6]	<10 [6]	<10 [6]	<10 [6]	<10 [6]	
2018/19	10 - <20	436	<10 [6]	<10 [6]	<10 [6]	<10 [6]	
	20 - <30	7,163	11 (0.2)	<10 [6]	<10 [6]	<10 [6]	
	30 - <40	7,381	38 (0.5)	10 (26.3)	<10 [6]	10 (26.3)	
	40 - <50	4,180	70 (1.7)	37 (52.9)	<10 [6]	38 (54.3)	
	50 - <60	1,978	152 (7.7)	115 (75.7)	<10 [6]	116 (76.3)	
	60 - <70	701	116 (16.5)	90 (77.6)	<10 [6]	93 (80.2)	
	70 - <80	209	41 (19.6)	34 (82.9)	<10 [6]	34 (82.9)	
	80 - <90	45	13 (28.9)	11 (84.6)	<10 [6]	12 (92.3)	
	90 - <100	<10 [6]	<10 [6]	<10 [6]	<10 [6]	<10 [6]	
	100 - <110	<10 [6]	<10 [6]	<10 [6]	<10 [6]	<10 [6]	
2019/20	10 - <20	404	<10 [6]	<10 [6]	<10 [6]	<10 [6]	
	20 - <30	8,064	<10 [6]	<10 [6]	<10 [6]	<10 [6]	
	30 - <40	9,125	37 (0.4)	<10 [6]	<10 [6]	<10 [6]	
	40 - <50	4,948	84 (1.7)	44 (52.4)	<10 [6]	45 (53.6)	
	50 - <60	2,224	164 (7.4)	126 (76.8)	<10 [6]	129 (78.7)	
	60 - <70	751	137 (18.2)	113 (82.5)	<10 [6]	117 (85.4)	
	70 - <80	238	49 (20.6)	43 (87.8)	<10 [6]	43 (87.8)	
	80 - <90	53	13 (24.5)	12 (92.3)	<10 [6]	12 (92.3)	
	90 - <100	<10 [6]	<10 [6]	<10 [6]	<10 [6]	<10 [6]	
	100 - <110	<10 [6]	<10 [6]	<10 [6]	<10 [6]	<10 [6]	

[1]Eligible for indicator; [2]Achieve in current prison; [3]Achieve in previous prison; [4]Overall achievement - either current or previous prison; [5]Declined indicator; [6]Suppressed (<10) to avoid disclosure

Indicator *The proportion of people with coronary heart disease who have received a prescription for anti-platelet or anti-coagulant therapy in the preceding 12 months*

Group: Cardiovascular Disease

Community achievement 2019/20: 90.87% (QOF 19/20)

	Variable	Population	Eligible[1] (% popln)	Satisfy[2] (% eligible)	Elsewhere[3] (% eligible)	Achieve[4] (% eligible)
**Length of Stay (months)**						
2017/18	<1	4,474	37 (0.8)	16 (43.2)	<10 [6]	17 (45.9)
	1-<6	8,075	78 (1.0)	54 (69.2)	<10 [6]	55 (70.5)
	6-<12	3,672	54 (1.5)	46 (85.2)	<10 [6]	46 (85.2)
	12-<24	2,832	51 (1.8)	37 (72.5)	<10 [6]	37 (72.5)
	24+	2,624	135 (5.1)	109 (80.7)	<10 [6]	110 (81.5)
2018/19	<1	4,801	60 (1.2)	26 (43.3)	<10 [6]	29 (48.3)
	1-<6	7,742	107 (1.4)	69 (64.5)	<10 [6]	70 (65.4)
	6-<12	3,616	61 (1.7)	40 (65.6)	<10 [6]	41 (67.2)
	12-<24	3,447	73 (2.1)	55 (75.3)	<10 [6]	56 (76.7)
	24+	2,493	140 (5.6)	112 (80.0)	<10 [6]	112 (80.0)
2019/20	<1	5,745	69 (1.2)	37 (53.6)	<10 [6]	39 (56.5)
	1-<6	9,697	117 (1.2)	75 (64.1)	<10 [6]	79 (67.5)
	6-<12	5,090	97 (1.9)	67 (69.1)	<10 [6]	70 (72.2)
	12-<24	3,244	84 (2.6)	69 (82.1)	<10 [6]	69 (82.1)
	24+	2,035	127 (6.2)	103 (81.1)	<10 [6]	103 (81.1)
**Ethnic Group**						
2017/18	White	15,638	302 (1.9)	226 (74.8)	<10 [6]	229 (75.8)
	Mixed	431	<10 [6]	<10 [6]	<10 [6]	<10 [6]
	Asian or Asian British	813	<10 [6]	<10 [6]	<10 [6]	<10 [6]
	Black or Black British	404	<10 [6]	<10 [6]	<10 [6]	<10 [6]
	Chinese and Other	214	<10 [6]	<10 [6]	<10 [6]	<10 [6]
	Unclassified	372	12 (3.2)	<10 [6]	<10 [6]	<10 [6]
2018/19	White	14,911	351 (2.4)	247 (70.4)	<10 [6]	252 (71.8)
	Mixed	371	<10 [6]	<10 [6]	<10 [6]	<10 [6]
	Asian or Asian British	726	14 (1.9)	<10 [6]	<10 [6]	<10 [6]
	Black or Black British	364	<10 [6]	<10 [6]	<10 [6]	<10 [6]
	Chinese and Other	167	<10 [6]	<10 [6]	<10 [6]	<10 [6]
	Unclassified	409	<10 [6]	<10 [6]	<10 [6]	<10 [6]
2019/20	White	16,606	378 (2.3)	275 (72.8)	<10 [6]	283 (74.9)
	Mixed	409	<10 [6]	<10 [6]	<10 [6]	<10 [6]
	Asian or Asian British	755	21 (2.8)	12 (57.1)	<10 [6]	12 (57.1)
	Black or Black British	451	<10 [6]	<10 [6]	<10 [6]	<10 [6]
	Chinese and Other	163	<10 [6]	<10 [6]	<10 [6]	<10 [6]
	Unclassified	387	<10 [6]	<10 [6]	<10 [6]	<10 [6]

[1]Eligible for indicator; [2]Achieve in current prison; [3]Achieve in previous prison; [4]Overall achievement - either current or previous prison; [5]Declined indicator; [6]Suppressed (<10) to avoid disclosure

Indicator	*The proportion of people with heart failure who have been prescribed an ACE-inhibitor or angiotensin receptor blocker in the preceding 12 months*						*Community achievement 2019/20: 81.95% (QOF 19/20)*
	**Group: Cardiovascular Disease**						
	**Variable**	**Population**	**Eligible[1] (% popln)**	**Satisfy[2] (% eligible)**	**Elsewhere[3] (% eligible)**	**Achieve[4] (% eligible)**	
**Year**							
	2017/18	21,677	61 (0.3)	44 (72.1)	<10 [6]	46 (75.4)	
	2018/19	22,099	75 (0.3)	50 (66.7)	<10 [6]	52 (69.3)	
	2019/20	25,811	98 (0.4)	61 (62.2)	<10 [6]	62 (63.3)	
**Prison**							
2017/18	Prison 1	1,323	<10 [6]	<10 [6]	<10 [6]	<10 [6]	
	Prison 2	3,261	<10 [6]	<10 [6]	<10 [6]	<10 [6]	
	Prison 3	2,623	10 (0.4)	<10 [6]	<10 [6]	10 (100.0)	
	Prison 4	2,089	13 (0.6)	10 (76.9)	<10 [6]	10 (76.9)	
	Prison 5	637	<10 [6]	<10 [6]	<10 [6]	<10 [6]	
	Prison 6	1,552	<10 [6]	<10 [6]	<10 [6]	<10 [6]	
	Prison 7	635	<10 [6]	<10 [6]	<10 [6]	<10 [6]	
	Prison 8	1,085	<10 [6]	<10 [6]	<10 [6]	<10 [6]	
	Prison 9	981	<10 [6]	<10 [6]	<10 [6]	<10 [6]	
	Prison 10	2,523	<10 [6]	<10 [6]	<10 [6]	<10 [6]	
	Prison 11	3,470	11 (0.3)	<10 [6]	<10 [6]	<10 [6]	
	Prison 12	815	<10 [6]	<10 [6]	<10 [6]	<10 [6]	
	Prison 13	683	<10 [6]	<10 [6]	<10 [6]	<10 [6]	
2018/19	Prison 1	1,333	<10 [6]	<10 [6]	<10 [6]	<10 [6]	
	Prison 2	2,705	<10 [6]	<10 [6]	<10 [6]	<10 [6]	
	Prison 3	2,522	13 (0.5)	10 (76.9)	<10 [6]	11 (84.6)	
	Prison 4	2,349	19 (0.8)	13 (68.4)	<10 [6]	13 (68.4)	
	Prison 5	676	<10 [6]	<10 [6]	<10 [6]	<10 [6]	
	Prison 6	1,513	<10 [6]	<10 [6]	<10 [6]	<10 [6]	
	Prison 7	654	<10 [6]	<10 [6]	<10 [6]	<10 [6]	
	Prison 8	1,148	<10 [6]	<10 [6]	<10 [6]	<10 [6]	
	Prison 9	996	<10 [6]	<10 [6]	<10 [6]	<10 [6]	
	Prison 10	2,717	<10 [6]	<10 [6]	<10 [6]	<10 [6]	
	Prison 11	4,020	<10 [6]	<10 [6]	<10 [6]	<10 [6]	
	Prison 12	792	<10 [6]	<10 [6]	<10 [6]	<10 [6]	
	Prison 13	674	<10 [6]	<10 [6]	<10 [6]	<10 [6]	
2019/20	Prison 1	1,410	<10 [6]	<10 [6]	<10 [6]	<10 [6]	
	Prison 2	2,979	<10 [6]	<10 [6]	<10 [6]	<10 [6]	
	Prison 3	2,809	18 (0.6)	11 (61.1)	<10 [6]	11 (61.1)	
	Prison 4	2,651	16 (0.6)	12 (75.0)	<10 [6]	12 (75.0)	
	Prison 5	616	<10 [6]	<10 [6]	<10 [6]	<10 [6]	
	Prison 6	1,533	<10 [6]	<10 [6]	<10 [6]	<10 [6]	
	Prison 7	860	<10 [6]	<10 [6]	<10 [6]	<10 [6]	
	Prison 8	1,385	<10 [6]	<10 [6]	<10 [6]	<10 [6]	
	Prison 9	1,092	11 (1.0)	<10 [6]	<10 [6]	<10 [6]	
	Prison 10	3,577	13 (0.4)	11 (84.6)	<10 [6]	11 (84.6)	
	Prison 11	5,348	15 (0.3)	<10 [6]	<10 [6]	<10 [6]	
	Prison 12	805	<10 [6]	<10 [6]	<10 [6]	<10 [6]	
	Prison 13	746	<10 [6]	<10 [6]	<10 [6]	<10 [6]	
**Prison category**							
2017/18	A	1,664	<10 [6]	<10 [6]	<10 [6]	<10 [6]	
	B	9,254	19 (0.2)	14 (73.7)	<10 [6]	14 (73.7)	
	C	6,035	23 (0.4)	19 (82.6)	<10 [6]	20 (87.0)	
	Closed	1,720	<10 [6]	<10 [6]	<10 [6]	<10 [6]	
	D	2,189	<10 [6]	<10 [6]	<10 [6]	<10 [6]	
	YOI	815	<10 [6]	<10 [6]	<10 [6]	<10 [6]	
2018/19	A	1,670	11 (0.7)	<10 [6]	<10 [6]	<10 [6]	
	B	9,442	20 (0.2)	14 (70.0)	<10 [6]	14 (70.0)	
	C	6,204	33 (0.5)	24 (72.7)	<10 [6]	25 (75.8)	
	Closed	1,802	<10 [6]	<10 [6]	<10 [6]	<10 [6]	
	D	2,189	<10 [6]	<10 [6]	<10 [6]	<10 [6]	
	YOI	792	<10 [6]	<10 [6]	<10 [6]	<10 [6]	
2019/20	A	1,838	14 (0.8)	<10 [6]	<10 [6]	<10 [6]	
	B	11,904	34 (0.3)	23 (67.6)	<10 [6]	23 (67.6)	
	C	6,870	36 (0.5)	24 (66.7)	<10 [6]	25 (69.4)	
	Closed	2,245	<10 [6]	<10 [6]	<10 [6]	<10 [6]	
	D	2,149	<10 [6]	<10 [6]	<10 [6]	<10 [6]	
	YOI	805	<10 [6]	<10 [6]	<10 [6]	<10 [6]	
**Gender**							
2017/18	F	1,699	<10 [6]	<10 [6]	<10 [6]	<10 [6]	
	M	19,977	60 (0.3)	43 (71.7)	<10 [6]	45 (75.0)	
2018/19	F	1,802	<10 [6]	<10 [6]	<10 [6]	<10 [6]	
	M	20,295	72 (0.4)	49 (68.1)	<10 [6]	51 (70.8)	
2019/20	F	1,376	<10 [6]	<10 [6]	<10 [6]	<10 [6]	
	M	23,570	91 (0.4)	59 (64.8)	<10 [6]	60 (65.9)	

[1]Eligible for indicator; [2]Achieve in current prison; [3]Achieve in previous prison; [4]Overall achievement - either current or previous prison; [5]Declined indicator; [6]Suppressed (<10) to avoid disclosure

Indicator ***The proportion of people with heart failure who have been prescribed an ACE-inhibitor or angiotensin receptor blocker in the preceding 12 months***

Community achievement 2019/20: 81.95% (QOF 19/20)

Group: Cardiovascular Disease

	Variable	Population	Eligible[1] (% popln)	Satisfy[2] (% eligible)	Elsewhere[3] (% eligible)	Achieve[4] (% eligible)
**Sentence Status**						
2017/18	.	81	<10 [6]	<10 [6]	<10 [6]	<10 [6]
	Absconded	<10 [6]	<10 [6]	<10 [6]	<10 [6]	<10 [6]
	Active In	15,872	49 (0.3)	37 (75.5)	<10 [6]	38 (77.6)
	Active Out	1,052	<10 [6]	<10 [6]	<10 [6]	<10 [6]
	Convicted Sentence	2,125	<10 [6]	<10 [6]	<10 [6]	<10 [6]
	Downgrade in security category	<10 [6]	<10 [6]	<10 [6]	<10 [6]	<10 [6]
	Internal Cell Move	<10 [6]	<10 [6]	<10 [6]	<10 [6]	<10 [6]
	Judges Remand	92	<10 [6]	<10 [6]	<10 [6]	<10 [6]
	Licence Revoke	52	<10 [6]	<10 [6]	<10 [6]	<10 [6]
	On Remand	1,492	<10 [6]	<10 [6]	<10 [6]	<10 [6]
	Transfer	910	<10 [6]	<10 [6]	<10 [6]	<10 [6]
	Upgrade in security category	<10 [6]	<10 [6]	<10 [6]	<10 [6]	<10 [6]
2018/19	.	88	<10 [6]	<10 [6]	<10 [6]	<10 [6]
	Absconded	<10 [6]	<10 [6]	<10 [6]	<10 [6]	<10 [6]
	Active In	18,145	65 (0.4)	43 (66.2)	<10 [6]	45 (69.2)
	Active Out	835	<10 [6]	<10 [6]	<10 [6]	<10 [6]
	Convicted Sentence	1,320	<10 [6]	<10 [6]	<10 [6]	<10 [6]
	Downgrade in security category	<10 [6]	<10 [6]	<10 [6]	<10 [6]	<10 [6]
	Internal Cell Move	<10 [6]	<10 [6]	<10 [6]	<10 [6]	<10 [6]
	Judges Remand	<10 [6]	<10 [6]	<10 [6]	<10 [6]	<10 [6]
	Licence Revoke	125	<10 [6]	<10 [6]	<10 [6]	<10 [6]
	On Remand	1,059	<10 [6]	<10 [6]	<10 [6]	<10 [6]
	Transfer	518	<10 [6]	<10 [6]	<10 [6]	<10 [6]
	Upgrade in security category	<10 [6]	<10 [6]	<10 [6]	<10 [6]	<10 [6]
2019/20	.	69	<10 [6]	<10 [6]	<10 [6]	<10 [6]
	Absconded	<10 [6]	<10 [6]	<10 [6]	<10 [6]	<10 [6]
	Active In	22,424	88 (0.4)	55 (62.5)	<10 [6]	56 (63.6)
	Active Out	625	<10 [6]	<10 [6]	<10 [6]	<10 [6]
	Convicted Sentence	1,361	<10 [6]	<10 [6]	<10 [6]	<10 [6]
	Downgrade in security category	<10 [6]	<10 [6]	<10 [6]	<10 [6]	<10 [6]
	Internal Cell Move	<10 [6]	<10 [6]	<10 [6]	<10 [6]	<10 [6]
	Judges Remand	19	<10 [6]	<10 [6]	<10 [6]	<10 [6]
	Licence Revoke	178	<10 [6]	<10 [6]	<10 [6]	<10 [6]
	On Remand	1,031	<10 [6]	<10 [6]	<10 [6]	<10 [6]
	Transfer	101	<10 [6]	<10 [6]	<10 [6]	<10 [6]
	Upgrade in security category	<10 [6]	<10 [6]	<10 [6]	<10 [6]	<10 [6]
**Age - years**						
2017/18	10 - <20	468	<10 [6]	<10 [6]	<10 [6]	<10 [6]
	20 - <30	6,994	<10 [6]	<10 [6]	<10 [6]	<10 [6]
	30 - <40	7,051	<10 [6]	<10 [6]	<10 [6]	<10 [6]
	40 - <50	4,114	<10 [6]	<10 [6]	<10 [6]	<10 [6]
	50 - <60	2,107	15 (0.7)	14 (93.3)	<10 [6]	14 (93.3)
	60 - <70	684	16 (2.3)	12 (75.0)	<10 [6]	13 (81.3)
	70 - <80	213	15 (7.0)	<10 [6]	<10 [6]	10 (66.7)
	80 - <90	40	<10 [6]	<10 [6]	<10 [6]	<10 [6]
	90 - <100	<10 [6]	<10 [6]	<10 [6]	<10 [6]	<10 [6]
	100 - <110	<10 [6]	<10 [6]	<10 [6]	<10 [6]	<10 [6]
2018/19	10 - <20	436	<10 [6]	<10 [6]	<10 [6]	<10 [6]
	20 - <30	7,163	<10 [6]	<10 [6]	<10 [6]	<10 [6]
	30 - <40	7,381	<10 [6]	<10 [6]	<10 [6]	<10 [6]
	40 - <50	4,180	13 (0.3)	<10 [6]	<10 [6]	<10 [6]
	50 - <60	1,978	22 (1.1)	19 (86.4)	<10 [6]	19 (86.4)
	60 - <70	701	14 (2.0)	<10 [6]	<10 [6]	<10 [6]
	70 - <80	209	14 (6.7)	<10 [6]	<10 [6]	<10 [6]
	80 - <90	45	<10 [6]	<10 [6]	<10 [6]	<10 [6]
	90 - <100	<10 [6]	<10 [6]	<10 [6]	<10 [6]	<10 [6]
	100 - <110	<10 [6]	<10 [6]	<10 [6]	<10 [6]	<10 [6]
2019/20	10 - <20	404	<10 [6]	<10 [6]	<10 [6]	<10 [6]
	20 - <30	8,064	<10 [6]	<10 [6]	<10 [6]	<10 [6]
	30 - <40	9,125	<10 [6]	<10 [6]	<10 [6]	<10 [6]
	40 - <50	4,948	14 (0.3)	<10 [6]	<10 [6]	<10 [6]
	50 - <60	2,224	28 (1.3)	18 (64.3)	<10 [6]	18 (64.3)
	60 - <70	751	21 (2.8)	12 (57.1)	<10 [6]	13 (61.9)
	70 - <80	238	14 (5.9)	11 (78.6)	<10 [6]	11 (78.6)
	80 - <90	53	<10 [6]	<10 [6]	<10 [6]	<10 [6]
	90 - <100	<10 [6]	<10 [6]	<10 [6]	<10 [6]	<10 [6]
	100 - <110	<10 [6]	<10 [6]	<10 [6]	<10 [6]	<10 [6]

[1]Eligible for indicator; [2]Achieve in current prison; [3]Achieve in previous prison; [4]Overall achievement - either current or previous prison; [5]Declined indicator; [6]Suppressed (<10) to avoid disclosure

Indicator	*The proportion of people with heart failure who have been prescribed an ACE-inhibitor or angiotensin receptor blocker in the preceding 12 months*					
	**Group: Cardiovascular Disease**					
	**Variable**	**Population**	**Eligible[1] (% popln)**	**Satisfy[2] (% eligible)**	**Elsewhere[3] (% eligible)**	**Achieve[4] (% eligible)**
**Length of Stay (months)**						
2017/18	<1	4,474	<10 [6]	<10 [6]	<10 [6]	<10 [6]
	1-<6	8,075	15 (0.2)	11 (73.3)	<10 [6]	12 (80.0)
	6-<12	3,672	<10 [6]	<10 [6]	<10 [6]	<10 [6]
	12-<24	2,832	<10 [6]	<10 [6]	<10 [6]	<10 [6]
	24+	2,624	22 (0.8)	15 (68.2)	<10 [6]	15 (68.2)
2018/19	<1	4,801	<10 [6]	<10 [6]	<10 [6]	<10 [6]
	1-<6	7,742	16 (0.2)	<10 [6]	<10 [6]	<10 [6]
	6-<12	3,616	12 (0.3)	11 (91.7)	<10 [6]	11 (91.7)
	12-<24	3,447	15 (0.4)	12 (80.0)	<10 [6]	13 (86.7)
	24+	2,493	26 (1.0)	15 (57.7)	<10 [6]	15 (57.7)
2019/20	<1	5,745	<10 [6]	<10 [6]	<10 [6]	<10 [6]
	1-<6	9,697	26 (0.3)	11 (42.3)	<10 [6]	11 (42.3)
	6-<12	5,090	20 (0.4)	13 (65.0)	<10 [6]	14 (70.0)
	12-<24	3,244	24 (0.7)	18 (75.0)	<10 [6]	18 (75.0)
	24+	2,035	21 (1.0)	13 (61.9)	<10 [6]	13 (61.9)
**Ethnic group**						
2017/18	White	15,638	53 (0.3)	39 (73.6)	<10 [6]	41 (77.4)
	Mixed	431	<10 [6]	<10 [6]	<10 [6]	<10 [6]
	Asian or Asian British	813	<10 [6]	<10 [6]	<10 [6]	<10 [6]
	Black or Black British	404	<10 [6]	<10 [6]	<10 [6]	<10 [6]
	Chinese and Other	214	<10 [6]	<10 [6]	<10 [6]	<10 [6]
	Unclassified	372	<10 [6]	<10 [6]	<10 [6]	<10 [6]
2018/19	White	14,911	63 (0.4)	42 (66.7)	<10 [6]	44 (69.8)
	Mixed	371	<10 [6]	<10 [6]	<10 [6]	<10 [6]
	Asian or Asian British	726	<10 [6]	<10 [6]	<10 [6]	<10 [6]
	Black or Black British	364	<10 [6]	<10 [6]	<10 [6]	<10 [6]
	Chinese and Other	167	<10 [6]	<10 [6]	<10 [6]	<10 [6]
	Unclassified	409	<10 [6]	<10 [6]	<10 [6]	<10 [6]
2019/20	White	16,606	79 (0.5)	47 (59.5)	<10 [6]	48 (60.8)
	Mixed	409	<10 [6]	<10 [6]	<10 [6]	<10 [6]
	Asian or Asian British	755	<10 [6]	<10 [6]	<10 [6]	<10 [6]
	Black or Black British	451	<10 [6]	<10 [6]	<10 [6]	<10 [6]
	Chinese and Other	163	<10 [6]	<10 [6]	<10 [6]	<10 [6]
	Unclassified	387	<10 [6]	<10 [6]	<10 [6]	<10 [6]

Community achievement 2019/20: 81.95% (QOF 19/20)

[1]Eligible for indicator; [2]Achieve in current prison; [3]Achieve in previous prison; [4]Overall achievement - either current or previous prison; [5]Declined indicator; [6]Suppressed (<10) to avoid disclosure

Indicator	*The proportion of people with heart failure who have been prescribed an ACE-inhibitor or angiotensin receptor blocker, and a beta blocker, in the preceding 12 months*					*Community achievement 2019/20: 82.01% (QOF 19/20)*
	**Group: Cardiovascular Disease**					
	**Variable**	**Population**	**Eligible[1] (% popln)**	**Satisfy[2] (% eligible)**	**Elsewhere[3] (% eligible)**	**Achieve[4] (% eligible)**
**Year**						
	2017/18	21,677	44 (0.2)	35 (79.5)	<10 [6]	36 (81.8)
	2018/19	22,099	50 (0.2)	40 (80.0)	<10 [6]	42 (84.0)
	2019/20	25,811	61 (0.2)	45 (73.8)	<10 [6]	49 (80.3)
**Prison**						
2017/18	Prison 1	1,323	<10 [6]	<10 [6]	<10 [6]	<10 [6]
	Prison 2	3,261	<10 [6]	<10 [6]	<10 [6]	<10 [6]
	Prison 3	2,623	<10 [6]	<10 [6]	<10 [6]	<10 [6]
	Prison 4	2,089	10 (0.5)	<10 [6]	<10 [6]	<10 [6]
	Prison 5	637	<10 [6]	<10 [6]	<10 [6]	<10 [6]
	Prison 6	1,552	<10 [6]	<10 [6]	<10 [6]	<10 [6]
	Prison 7	635	<10 [6]	<10 [6]	<10 [6]	<10 [6]
	Prison 8	1,085	<10 [6]	<10 [6]	<10 [6]	<10 [6]
	Prison 9	981	<10 [6]	<10 [6]	<10 [6]	<10 [6]
	Prison 10	2,523	<10 [6]	<10 [6]	<10 [6]	<10 [6]
	Prison 11	3,470	<10 [6]	<10 [6]	<10 [6]	<10 [6]
	Prison 12	815	<10 [6]	<10 [6]	<10 [6]	<10 [6]
	Prison 13	683	<10 [6]	<10 [6]	<10 [6]	<10 [6]
2018/19	Prison 1	1,333	<10 [6]	<10 [6]	<10 [6]	<10 [6]
	Prison 2	2,705	<10 [6]	<10 [6]	<10 [6]	<10 [6]
	Prison 3	2,522	10 (0.4)	<10 [6]	<10 [6]	<10 [6]
	Prison 4	2,349	13 (0.6)	11 (84.6)	<10 [6]	11 (84.6)
	Prison 5	676	<10 [6]	<10 [6]	<10 [6]	<10 [6]
	Prison 6	1,513	<10 [6]	<10 [6]	<10 [6]	<10 [6]
	Prison 7	654	<10 [6]	<10 [6]	<10 [6]	<10 [6]
	Prison 8	1,148	<10 [6]	<10 [6]	<10 [6]	<10 [6]
	Prison 9	996	<10 [6]	<10 [6]	<10 [6]	<10 [6]
	Prison 10	2,717	<10 [6]	<10 [6]	<10 [6]	<10 [6]
	Prison 11	4,020	<10 [6]	<10 [6]	<10 [6]	<10 [6]
	Prison 12	792	<10 [6]	<10 [6]	<10 [6]	<10 [6]
	Prison 13	674	<10 [6]	<10 [6]	<10 [6]	<10 [6]
2019/20	Prison 1	1,410	<10 [6]	<10 [6]	<10 [6]	<10 [6]
	Prison 2	2,979	<10 [6]	<10 [6]	<10 [6]	<10 [6]
	Prison 3	2,809	11 (0.4)	<10 [6]	<10 [6]	<10 [6]
	Prison 4	2,651	12 (0.5)	11 (91.7)	<10 [6]	11 (91.7)
	Prison 5	616	<10 [6]	<10 [6]	<10 [6]	<10 [6]
	Prison 6	1,533	<10 [6]	<10 [6]	<10 [6]	<10 [6]
	Prison 7	860	<10 [6]	<10 [6]	<10 [6]	<10 [6]
	Prison 8	1,385	<10 [6]	<10 [6]	<10 [6]	<10 [6]
	Prison 9	1,092	<10 [6]	<10 [6]	<10 [6]	<10 [6]
	Prison 10	3,577	11 (0.3)	<10 [6]	<10 [6]	<10 [6]
	Prison 11	5,348	<10 [6]	<10 [6]	<10 [6]	<10 [6]
	Prison 12	805	<10 [6]	<10 [6]	<10 [6]	<10 [6]
	Prison 13	746	<10 [6]	<10 [6]	<10 [6]	<10 [6]
**Prison category**						
2017/18	A	1,664	<10 [6]	<10 [6]	<10 [6]	<10 [6]
	B	9,254	14 (0.2)	11 (78.6)	<10 [6]	11 (78.6)
	C	6,035	19 (0.3)	16 (84.2)	<10 [6]	16 (84.2)
	Closed	1,720	<10 [6]	<10 [6]	<10 [6]	<10 [6]
	D	2,189	<10 [6]	<10 [6]	<10 [6]	<10 [6]
	YOI	815	<10 [6]	<10 [6]	<10 [6]	<10 [6]
2018/19	A	1,670	<10 [6]	<10 [6]	<10 [6]	<10 [6]
	B	9,442	14 (0.1)	12 (85.7)	<10 [6]	12 (85.7)
	C	6,204	24 (0.4)	19 (79.2)	<10 [6]	19 (79.2)
	Closed	1,802	<10 [6]	<10 [6]	<10 [6]	<10 [6]
	D	2,189	<10 [6]	<10 [6]	<10 [6]	<10 [6]
	YOI	792	<10 [6]	<10 [6]	<10 [6]	<10 [6]
2019/20	A	1,838	<10 [6]	<10 [6]	<10 [6]	<10 [6]
	B	11,904	23 (0.2)	16 (69.6)	<10 [6]	18 (78.3)
	C	6,870	24 (0.3)	16 (66.7)	<10 [6]	17 (70.8)
	Closed	2,245	<10 [6]	<10 [6]	<10 [6]	<10 [6]
	D	2,149	<10 [6]	<10 [6]	<10 [6]	<10 [6]
	YOI	805	<10 [6]	<10 [6]	<10 [6]	<10 [6]
**Gender**						
2017/18	F	1,699	<10 [6]	<10 [6]	<10 [6]	<10 [6]
	M	19,977	43 (0.2)	34 (79.1)	<10 [6]	35 (81.4)
2018/19	F	1,802	<10 [6]	<10 [6]	<10 [6]	<10 [6]
	M	20,295	49 (0.2)	40 (81.6)	<10 [6]	41 (83.7)
2019/20	F	1,376	<10 [6]	<10 [6]	<10 [6]	<10 [6]
	M	23,570	59 (0.3)	43 (72.9)	<10 [6]	47 (79.7)

[1]Eligible for indicator; [2]Achieve in current prison; [3]Achieve in previous prison; [4]Overall achievement - either current or previous prison; [5]Declined indicator; [6]Suppressed (<10) to avoid disclosure

Indicator	*The proportion of people with heart failure who have been prescribed an ACE-inhibitor or angiotensin receptor blocker, and a beta blocker, in the preceding 12 months*					
	**Group: Cardiovascular Disease**					
	**Variable**	**Population**	**Eligible[1] (% popln)**	**Satisfy[2] (% eligible)**	**Elsewhere[3] (% eligible)**	**Achieve[4] (% eligible)**
**Sentence Status**						
2017/18	.	81	<10 [6]	<10 [6]	<10 [6]	<10 [6]
	Absconded	<10 [6]	<10 [6]	<10 [6]	<10 [6]	<10 [6]
	Active In	15,872	37 (0.2)	29 (78.4)	<10 [6]	30 (81.1)
	Active Out	1,052	<10 [6]	<10 [6]	<10 [6]	<10 [6]
	Convicted Sentence	2,125	<10 [6]	<10 [6]	<10 [6]	<10 [6]
	Downgrade in security category	<10 [6]	<10 [6]	<10 [6]	<10 [6]	<10 [6]
	Internal Cell Move	<10 [6]	<10 [6]	<10 [6]	<10 [6]	<10 [6]
	Judges Remand	92	<10 [6]	<10 [6]	<10 [6]	<10 [6]
	Licence Revoke	52	<10 [6]	<10 [6]	<10 [6]	<10 [6]
	On Remand	1,492	<10 [6]	<10 [6]	<10 [6]	<10 [6]
	Transfer	910	<10 [6]	<10 [6]	<10 [6]	<10 [6]
	Upgrade in security category	<10 [6]	<10 [6]	<10 [6]	<10 [6]	<10 [6]
2018/19	.	88	<10 [6]	<10 [6]	<10 [6]	<10 [6]
	Absconded	<10 [6]	<10 [6]	<10 [6]	<10 [6]	<10 [6]
	Active In	18,145	43 (0.2)	34 (79.1)	<10 [6]	36 (83.7)
	Active Out	835	<10 [6]	<10 [6]	<10 [6]	<10 [6]
	Convicted Sentence	1,320	<10 [6]	<10 [6]	<10 [6]	<10 [6]
	Downgrade in security category	<10 [6]	<10 [6]	<10 [6]	<10 [6]	<10 [6]
	Internal Cell Move	<10 [6]	<10 [6]	<10 [6]	<10 [6]	<10 [6]
	Judges Remand	<10 [6]	<10 [6]	<10 [6]	<10 [6]	<10 [6]
	Licence Revoke	125	<10 [6]	<10 [6]	<10 [6]	<10 [6]
	On Remand	1,059	<10 [6]	<10 [6]	<10 [6]	<10 [6]
	Transfer	518	<10 [6]	<10 [6]	<10 [6]	<10 [6]
	Upgrade in security category	<10 [6]	<10 [6]	<10 [6]	<10 [6]	<10 [6]
2019/20	.	69	<10 [6]	<10 [6]	<10 [6]	<10 [6]
	Absconded	<10 [6]	<10 [6]	<10 [6]	<10 [6]	<10 [6]
	Active In	22,424	55 (0.2)	39 (70.9)	<10 [6]	43 (78.2)
	Active Out	625	<10 [6]	<10 [6]	<10 [6]	<10 [6]
	Convicted Sentence	1,361	<10 [6]	<10 [6]	<10 [6]	<10 [6]
	Downgrade in security category	<10 [6]	<10 [6]	<10 [6]	<10 [6]	<10 [6]
	Internal Cell Move	<10 [6]	<10 [6]	<10 [6]	<10 [6]	<10 [6]
	Judges Remand	19	<10 [6]	<10 [6]	<10 [6]	<10 [6]
	Licence Revoke	178	<10 [6]	<10 [6]	<10 [6]	<10 [6]
	On Remand	1,031	<10 [6]	<10 [6]	<10 [6]	<10 [6]
	Transfer	101	<10 [6]	<10 [6]	<10 [6]	<10 [6]
	Upgrade in security category	<10 [6]	<10 [6]	<10 [6]	<10 [6]	<10 [6]
**Age - years**						
2017/18	10 - <20	468	<10 [6]	<10 [6]	<10 [6]	<10 [6]
	20 - <30	6,994	<10 [6]	<10 [6]	<10 [6]	<10 [6]
	30 - <40	7,051	<10 [6]	<10 [6]	<10 [6]	<10 [6]
	40 - <50	4,114	<10 [6]	<10 [6]	<10 [6]	<10 [6]
	50 - <60	2,107	14 (0.7)	<10 [6]	<10 [6]	10 (71.4)
	60 - <70	684	12 (1.8)	10 (83.3)	<10 [6]	10 (83.3)
	70 - <80	213	<10 [6]	<10 [6]	<10 [6]	<10 [6]
	80 - <90	40	<10 [6]	<10 [6]	<10 [6]	<10 [6]
	90 - <100	<10 [6]	<10 [6]	<10 [6]	<10 [6]	<10 [6]
	100 - <110	<10 [6]	<10 [6]	<10 [6]	<10 [6]	<10 [6]
2018/19	10 - <20	436	<10 [6]	<10 [6]	<10 [6]	<10 [6]
	20 - <30	7,163	<10 [6]	<10 [6]	<10 [6]	<10 [6]
	30 - <40	7,381	<10 [6]	<10 [6]	<10 [6]	<10 [6]
	40 - <50	4,180	<10 [6]	<10 [6]	<10 [6]	<10 [6]
	50 - <60	1,978	19 (1.0)	13 (68.4)	<10 [6]	14 (73.7)
	60 - <70	701	<10 [6]	<10 [6]	<10 [6]	<10 [6]
	70 - <80	209	<10 [6]	<10 [6]	<10 [6]	<10 [6]
	80 - <90	45	<10 [6]	<10 [6]	<10 [6]	<10 [6]
	90 - <100	<10 [6]	<10 [6]	<10 [6]	<10 [6]	<10 [6]
	100 - <110	<10 [6]	<10 [6]	<10 [6]	<10 [6]	<10 [6]
2019/20	10 - <20	404	<10 [6]	<10 [6]	<10 [6]	<10 [6]
	20 - <30	8,064	<10 [6]	<10 [6]	<10 [6]	<10 [6]
	30 - <40	9,125	<10 [6]	<10 [6]	<10 [6]	<10 [6]
	40 - <50	4,948	<10 [6]	<10 [6]	<10 [6]	<10 [6]
	50 - <60	2,224	18 (0.8)	15 (83.3)	<10 [6]	16 (88.9)
	60 - <70	751	12 (1.6)	<10 [6]	<10 [6]	10 (83.3)
	70 - <80	238	11 (4.6)	<10 [6]	<10 [6]	<10 [6]
	80 - <90	53	<10 [6]	<10 [6]	<10 [6]	<10 [6]
	90 - <100	<10 [6]	<10 [6]	<10 [6]	<10 [6]	<10 [6]
	100 - <110	<10 [6]	<10 [6]	<10 [6]	<10 [6]	<10 [6]

Community achievement 2019/20: 82.01% (QOF 19/20)

[1]Eligible for indicator; [2]Achieve in current prison; [3]Achieve in previous prison; [4]Overall achievement - either current or previous prison; [5]Declined indicator; [6]Suppressed (<10) to avoid disclosure

Indicator	*The proportion of people with heart failure who have been prescribed an ACE-inhibitor or angiotensin receptor blocker, and a beta blocker, in the preceding 12 months*					*Community achievement 2019/20: 82.01% (QOF 19/20)*
	**Group: Cardiovascular Disease**					
	**Variable**	**Population**	**Eligible[1] (% popln)**	**Satisfy[2] (% eligible)**	**Elsewhere[3] (% eligible)**	**Achieve[4] (% eligible)**
**Length of Stay (months)**						
2017/18	<1	4,474	<10 [6]	<10 [6]	<10 [6]	<10 [6]
	1-<6	8,075	11 (0.1)	11 (100.0)	<10 [6]	11 (100.0)
	6-<12	3,672	<10 [6]	<10 [6]	<10 [6]	<10 [6]
	12-<24	2,832	<10 [6]	<10 [6]	<10 [6]	<10 [6]
	24+	2,624	15 (0.6)	11 (73.3)	<10 [6]	11 (73.3)
2018/19	<1	4,801	<10 [6]	<10 [6]	<10 [6]	<10 [6]
	1-<6	7,742	<10 [6]	<10 [6]	<10 [6]	<10 [6]
	6-<12	3,616	11 (0.3)	10 (90.9)	<10 [6]	10 (90.9)
	12-<24	3,447	12 (0.3)	<10 [6]	<10 [6]	10 (83.3)
	24+	2,493	15 (0.6)	10 (66.7)	<10 [6]	10 (66.7)
2019/20	<1	5,745	<10 [6]	<10 [6]	<10 [6]	<10 [6]
	1-<6	9,697	11 (0.1)	<10 [6]	<10 [6]	<10 [6]
	6-<12	5,090	13 (0.3)	10 (76.9)	<10 [6]	11 (84.6)
	12-<24	3,244	18 (0.6)	14 (77.8)	<10 [6]	16 (88.9)
	24+	2,035	13 (0.6)	11 (84.6)	<10 [6]	11 (84.6)
**Ethnic Group**						
2017/18	White	15,638	39 (0.2)	32 (82.1)	<10 [6]	32 (82.1)
	Mixed	431	<10 [6]	<10 [6]	<10 [6]	<10 [6]
	Asian or Asian British	813	<10 [6]	<10 [6]	<10 [6]	<10 [6]
	Black or Black British	404	<10 [6]	<10 [6]	<10 [6]	<10 [6]
	Chinese and Other	214	<10 [6]	<10 [6]	<10 [6]	<10 [6]
	Unclassified	372	<10 [6]	<10 [6]	<10 [6]	<10 [6]
2018/19	White	14,911	42 (0.3)	33 (78.6)	<10 [6]	35 (83.3)
	Mixed	371	<10 [6]	<10 [6]	<10 [6]	<10 [6]
	Asian or Asian British	726	<10 [6]	<10 [6]	<10 [6]	<10 [6]
	Black or Black British	364	<10 [6]	<10 [6]	<10 [6]	<10 [6]
	Chinese and Other	167	<10 [6]	<10 [6]	<10 [6]	<10 [6]
	Unclassified	409	<10 [6]	<10 [6]	<10 [6]	<10 [6]
2019/20	White	16,606	47 (0.3)	34 (72.3)	<10 [6]	38 (80.9)
	Mixed	409	<10 [6]	<10 [6]	<10 [6]	<10 [6]
	Asian or Asian British	755	<10 [6]	<10 [6]	<10 [6]	<10 [6]
	Black or Black British	451	<10 [6]	<10 [6]	<10 [6]	<10 [6]
	Chinese and Other	163	<10 [6]	<10 [6]	<10 [6]	<10 [6]
	Unclassified	387	<10 [6]	<10 [6]	<10 [6]	<10 [6]

[1]Eligible for indicator; [2]Achieve in current prison; [3]Achieve in previous prison; [4]Overall achievement - either current or previous prison; [5]Declined indicator; [6]Suppressed (<10) to avoid disclosure

Indicator	*The proportion of women eligible for screening and aged 25-49 years who have had cervical screening in the preceding five years and six months*	*Community achievement 2019/20: 71.48% (QOF 19/20)*
	**Group: Screening**	

	**Variable**	**Population**	**Eligible[1] (% popln)**	**Declined[5] (% eligible)**	**Satisfy[2] (% eligible)**	**Elsewhere[3] (% eligible)**	**Achieve[4] (% eligible)**
**Year**							
	2017/18	21,677	1,294 (6.0)	85 (6.6)	629 (48.6)	90 (7.0)	719 (55.6)
	2018/19	22,099	1,411 (6.4)	81 (5.7)	835 (59.2)	85 (6.0)	920 (65.2)
	2019/20	25,811	1,796 (7.0)	80 (4.5)	972 (54.1)	173 (9.6)	1,145 (63.8)
**Prison**							
2017/18	Prison 1	1,323	<10 [6]	<10 [6]	<10 [6]	<10 [6]	<10 [6]
	Prison 2	3,261	<10 [6]	<10 [6]	<10 [6]	<10 [6]	<10 [6]
	Prison 3	2,623	<10 [6]	<10 [6]	<10 [6]	<10 [6]	<10 [6]
	Prison 4	2,089	<10 [6]	<10 [6]	<10 [6]	<10 [6]	<10 [6]
	Prison 5	637	<10 [6]	<10 [6]	<10 [6]	<10 [6]	<10 [6]
	Prison 6	1,552	<10 [6]	<10 [6]	<10 [6]	<10 [6]	<10 [6]
	Prison 7	635	491 (77.3)	40 (8.1)	252 (51.3)	52 (10.6)	304 (61.9)
	Prison 8	1,085	800 (73.7)	45 (5.6)	377 (47.1)	38 (4.8)	415 (51.9)
	Prison 9	981	<10 [6]	<10 [6]	<10 [6]	<10 [6]	<10 [6]
	Prison 10	2,523	<10 [6]	<10 [6]	<10 [6]	<10 [6]	<10 [6]
	Prison 11	3,470	<10 [6]	<10 [6]	<10 [6]	<10 [6]	<10 [6]
	Prison 12	815	<10 [6]	<10 [6]	<10 [6]	<10 [6]	<10 [6]
	Prison 13	683	<10 [6]	<10 [6]	<10 [6]	<10 [6]	<10 [6]
2018/19	Prison 1	1,333	<10 [6]	<10 [6]	<10 [6]	<10 [6]	<10 [6]
	Prison 2	2,705	<10 [6]	<10 [6]	<10 [6]	<10 [6]	<10 [6]
	Prison 3	2,522	<10 [6]	<10 [6]	<10 [6]	<10 [6]	<10 [6]
	Prison 4	2,349	<10 [6]	<10 [6]	<10 [6]	<10 [6]	<10 [6]
	Prison 5	676	<10 [6]	<10 [6]	<10 [6]	<10 [6]	<10 [6]
	Prison 6	1,513	<10 [6]	<10 [6]	<10 [6]	<10 [6]	<10 [6]
	Prison 7	654	515 (78.7)	37 (7.2)	280 (54.4)	59 (11.5)	339 (65.8)
	Prison 8	1,148	887 (77.3)	44 (5.0)	555 (62.6)	26 (2.9)	581 (65.5)
	Prison 9	996	<10 [6]	<10 [6]	<10 [6]	<10 [6]	<10 [6]
	Prison 10	2,717	<10 [6]	<10 [6]	<10 [6]	<10 [6]	<10 [6]
	Prison 11	4,020	<10 [6]	<10 [6]	<10 [6]	<10 [6]	<10 [6]
	Prison 12	792	<10 [6]	<10 [6]	<10 [6]	<10 [6]	<10 [6]
	Prison 13	674	<10 [6]	<10 [6]	<10 [6]	<10 [6]	<10 [6]
2019/20	Prison 1	1,410	<10 [6]	<10 [6]	<10 [6]	<10 [6]	<10 [6]
	Prison 2	2,979	<10 [6]	<10 [6]	<10 [6]	<10 [6]	<10 [6]
	Prison 3	2,809	<10 [6]	<10 [6]	<10 [6]	<10 [6]	<10 [6]
	Prison 4	2,651	<10 [6]	<10 [6]	<10 [6]	<10 [6]	<10 [6]
	Prison 5	616	<10 [6]	<10 [6]	<10 [6]	<10 [6]	<10 [6]
	Prison 6	1,533	<10 [6]	<10 [6]	<10 [6]	<10 [6]	<10 [6]
	Prison 7	860	688 (80.0)	35 (5.1)	262 (38.1)	128 (18.6)	390 (56.7)
	Prison 8	1,385	1,099 (79.4)	45 (4.1)	710 (64.6)	45 (4.1)	755 (68.7)
	Prison 9	1,092	<10 [6]	<10 [6]	<10 [6]	<10 [6]	<10 [6]
	Prison 10	3,577	<10 [6]	<10 [6]	<10 [6]	<10 [6]	<10 [6]
	Prison 11	5,348	<10 [6]	<10 [6]	<10 [6]	<10 [6]	<10 [6]
	Prison 12	805	<10 [6]	<10 [6]	<10 [6]	<10 [6]	<10 [6]
	Prison 13	746	<10 [6]	<10 [6]	<10 [6]	<10 [6]	<10 [6]
**Prison category**							
2017/18	A	1,664	<10 [6]	<10 [6]	<10 [6]	<10 [6]	<10 [6]
	B	9,254	<10 [6]	<10 [6]	<10 [6]	<10 [6]	<10 [6]
	C	6,035	<10 [6]	<10 [6]	<10 [6]	<10 [6]	<10 [6]
	Closed	1,720	1,291 (75.1)	85 (6.6)	629 (48.7)	90 (7.0)	719 (55.7)
	D	2,189	<10 [6]	<10 [6]	<10 [6]	<10 [6]	<10 [6]
	YOI	815	<10 [6]	<10 [6]	<10 [6]	<10 [6]	<10 [6]
2018/19	A	1,670	<10 [6]	<10 [6]	<10 [6]	<10 [6]	<10 [6]
	C	6,204	<10 [6]	<10 [6]	<10 [6]	<10 [6]	<10 [6]
	Closed	1,802	1,402 (77.8)	81 (5.8)	835 (59.6)	85 (6.1)	920 (65.6)
	D	2,189	<10 [6]	<10 [6]	<10 [6]	<10 [6]	<10 [6]
	YOI	792	<10 [6]	<10 [6]	<10 [6]	<10 [6]	<10 [6]
2019/20	A	1,838	<10 [6]	<10 [6]	<10 [6]	<10 [6]	<10 [6]
	C	6,870	<10 [6]	<10 [6]	<10 [6]	<10 [6]	<10 [6]
	Closed	2,245	1,787 (79.6)	80 (4.5)	972 (54.4)	173 (9.7)	1,145 (64.1)
	D	2,149	<10 [6]	<10 [6]	<10 [6]	<10 [6]	<10 [6]
	YOI	805	<10 [6]	<10 [6]	<10 [6]	<10 [6]	<10 [6]
**Gender**							
2017/18	F	1,699	1,294 (76.2)	85 (6.6)	629 (48.6)	90 (7.0)	719 (55.6)
	M	19,977	0 (0.0)	0 (0.0)	0 (0.0)	0 (0.0)	0 (0.0)
2018/19	F	1,802	1,411 (78.3)	81 (5.7)	835 (59.2)	85 (6.0)	920 (65.2)
	M	20,295	0 (0.0)	0 (0.0)	0 (0.0)	0 (0.0)	0 (0.0)
2019/20	F	1,376	1,100 (79.9)	45 (4.1)	710 (64.5)	45 (4.1)	755 (68.6)
	M	23,570	0 (0.0)	0 (0.0)	0 (0.0)	0 (0.0)	0 (0.0)

[1]Eligible for indicator; [2]Achieve in current prison; [3]Achieve in previous prison; [4]Overall achievement - either current or previous prison; [5]Declined indicator; [6]Suppressed (<10) to avoid disclosure

Indicator	*The proportion of women eligible for screening and aged 25-49 years who have had cervical screening in the preceding five years and six months*						*Community achievement 2019/20: 71.48% (QOF 19/20)*
	**Group: Screening**						
	**Variable**	**Population**	**Eligible[1] (% popln)**	**Declined[5] (% eligible)**	**Satisfy[2] (% eligible)**	**Elsewhere[3] (% eligible)**	**Achieve[4] (% eligible)**
**Sentence Status**							
2017/18	.	81	<10 [6]	<10 [6]	<10 [6]	<10 [6]	<10 [6]
	Absconded	<10 [6]	<10 [6]	<10 [6]	<10 [6]	<10 [6]	<10 [6]
	Active In	15,872	1,138 (7.2)	80 (7.0)	560 (49.2)	83 (7.3)	643 (56.5)
	Active Out	1,052	94 (8.9)	<10 [6]	45 (47.9)	<10 [6]	49 (52.1)
	Convicted Sentence	2,125	36 (1.7)	<10 [6]	13 (36.1)	<10 [6]	16 (44.4)
	Downgrade in security category	<10 [6]	<10 [6]	<10 [6]	<10 [6]	<10 [6]	<10 [6]
	Internal Cell Move	<10 [6]	<10 [6]	<10 [6]	<10 [6]	<10 [6]	<10 [6]
	Judges Remand	92	<10 [6]	<10 [6]	<10 [6]	<10 [6]	<10 [6]
	Licence Revoke	52	<10 [6]	<10 [6]	<10 [6]	<10 [6]	<10 [6]
	On Remand	1,492	21 (1.4)	<10 [6]	<10 [6]	<10 [6]	<10 [6]
	Transfer	910	<10 [6]	<10 [6]	<10 [6]	<10 [6]	<10 [6]
	Upgrade in security category	<10 [6]	<10 [6]	<10 [6]	<10 [6]	<10 [6]	<10 [6]
2018/19	.	88	<10 [6]	<10 [6]	<10 [6]	<10 [6]	<10 [6]
	Absconded	<10 [6]	<10 [6]	<10 [6]	<10 [6]	<10 [6]	<10 [6]
	Active In	18,145	1,262 (7.0)	75 (5.9)	765 (60.6)	81 (6.4)	846 (67.0)
	Active Out	835	90 (10.8)	<10 [6]	46 (51.1)	<10 [6]	49 (54.4)
	Convicted Sentence	1,320	36 (2.7)	<10 [6]	16 (44.4)	<10 [6]	17 (47.2)
	Downgrade in security category	<10 [6]	<10 [6]	<10 [6]	<10 [6]	<10 [6]	<10 [6]
	Internal Cell Move	<10 [6]	<10 [6]	<10 [6]	<10 [6]	<10 [6]	<10 [6]
	Judges Remand	<10 [6]	<10 [6]	<10 [6]	<10 [6]	<10 [6]	<10 [6]
	Licence Revoke	125	<10 [6]	<10 [6]	<10 [6]	<10 [6]	<10 [6]
	On Remand	1,059	14 (1.3)	<10 [6]	<10 [6]	<10 [6]	<10 [6]
	Transfer	518	<10 [6]	<10 [6]	<10 [6]	<10 [6]	<10 [6]
	Upgrade in security category	<10 [6]	<10 [6]	<10 [6]	<10 [6]	<10 [6]	<10 [6]
2019/20	.	69	<10 [6]	<10 [6]	<10 [6]	<10 [6]	<10 [6]
	Absconded	<10 [6]	<10 [6]	<10 [6]	<10 [6]	<10 [6]	<10 [6]
	Active In	22,424	1,650 (7.4)	76 (4.6)	910 (55.2)	167 (10.1)	1,077 (65.3)
	Active Out	625	104 (16.6)	<10 [6]	48 (46.2)	<10 [6]	52 (50.0)
	Convicted Sentence	1,361	16 (1.2)	<10 [6]	<10 [6]	<10 [6]	<10 [6]
	Downgrade in security category	<10 [6]	<10 [6]	<10 [6]	<10 [6]	<10 [6]	<10 [6]
	Internal Cell Move	<10 [6]	<10 [6]	<10 [6]	<10 [6]	<10 [6]	<10 [6]
	Judges Remand	19	<10 [6]	<10 [6]	<10 [6]	<10 [6]	<10 [6]
	Licence Revoke	178	<10 [6]	<10 [6]	<10 [6]	<10 [6]	<10 [6]
	On Remand	1,031	15 (1.5)	<10 [6]	<10 [6]	<10 [6]	<10 [6]
	Transfer	101	<10 [6]	<10 [6]	<10 [6]	<10 [6]	<10 [6]
	Upgrade in security category	<10 [6]	<10 [6]	<10 [6]	<10 [6]	<10 [6]	<10 [6]
**Age - years**							
2017/18	10 - <20	468	<10 [6]	<10 [6]	<10 [6]	<10 [6]	<10 [6]
	20 - <30	6,994	229 (3.3)	14 (6.1)	107 (46.7)	18 (7.9)	125 (54.6)
	30 - <40	7,051	615 (8.7)	35 (5.7)	306 (49.8)	49 (8.0)	355 (57.7)
	40 - <50	4,114	425 (10.3)	35 (8.2)	204 (48.0)	23 (5.4)	227 (53.4)
	50 - <60	2,107	25 (1.2)	<10 [6]	12 (48.0)	<10 [6]	12 (48.0)
	60 - <70	684	<10 [6]	<10 [6]	<10 [6]	<10 [6]	<10 [6]
	70 - <80	213	<10 [6]	<10 [6]	<10 [6]	<10 [6]	<10 [6]
	80 - <90	40	<10 [6]	<10 [6]	<10 [6]	<10 [6]	<10 [6]
	90 - <100	<10 [6]	<10 [6]	<10 [6]	<10 [6]	<10 [6]	<10 [6]
	100 - <110	<10 [6]	<10 [6]	<10 [6]	<10 [6]	<10 [6]	<10 [6]
2018/19	10 - <20	436	<10 [6]	<10 [6]	<10 [6]	<10 [6]	<10 [6]
	20 - <30	7,163	245 (3.4)	16 (6.5)	123 (50.2)	12 (4.9)	135 (55.1)
	30 - <40	7,381	650 (8.8)	30 (4.6)	404 (62.2)	41 (6.3)	445 (68.5)
	40 - <50	4,180	474 (11.3)	30 (6.3)	289 (61.0)	27 (5.7)	316 (66.7)
	50 - <60	1,978	42 (2.1)	<10 [6]	19 (45.2)	<10 [6]	24 (57.1)
	60 - <70	701	<10 [6]	<10 [6]	<10 [6]	<10 [6]	<10 [6]
	70 - <80	209	<10 [6]	<10 [6]	<10 [6]	<10 [6]	<10 [6]
	80 - <90	45	<10 [6]	<10 [6]	<10 [6]	<10 [6]	<10 [6]
	90 - <100	<10 [6]	<10 [6]	<10 [6]	<10 [6]	<10 [6]	<10 [6]
	100 - <110	<10 [6]	<10 [6]	<10 [6]	<10 [6]	<10 [6]	<10 [6]
2019/20	10 - <20	404	<10 [6]	<10 [6]	<10 [6]	<10 [6]	<10 [6]
	20 - <30	8,064	296 (3.7)	13 (4.4)	122 (41.2)	25 (8.4)	147 (49.7)
	30 - <40	9,125	894 (9.8)	32 (3.6)	512 (57.3)	83 (9.3)	595 (66.6)
	40 - <50	4,948	581 (11.7)	33 (5.7)	323 (55.6)	62 (10.7)	385 (66.3)
	50 - <60	2,224	25 (1.1)	<10 [6]	15 (60.0)	<10 [6]	18 (72.0)
	60 - <70	751	<10 [6]	<10 [6]	<10 [6]	<10 [6]	<10 [6]
	70 - <80	238	<10 [6]	<10 [6]	<10 [6]	<10 [6]	<10 [6]
	80 - <90	53	<10 [6]	<10 [6]	<10 [6]	<10 [6]	<10 [6]
	90 - <100	<10 [6]	<10 [6]	<10 [6]	<10 [6]	<10 [6]	<10 [6]
	100 - <110	<10 [6]	<10 [6]	<10 [6]	<10 [6]	<10 [6]	<10 [6]

[1]Eligible for indicator; [2]Achieve in current prison; [3]Achieve in previous prison; [4]Overall achievement - either current or previous prison; [5]Declined indicator; [6]Suppressed (<10) to avoid disclosure

Indicator	The proportion of women eligible for screening and aged 25-49 years who have had cervical screening in the preceding five years and six months						Community achievement 2019/20: 71.48% (QOF 19/20)
	Group: Screening						
	Variable	Population	Eligible[1] (% popln)	Declined[5] (% eligible)	Satisfy[2] (% eligible)	Elsewhere[3] (% eligible)	Achieve[4] (% eligible)
**Length of Stay (months)**							
2017/18	<1	4,474	282 (6.3)	18 (6.4)	96 (34.0)	12 (4.3)	108 (38.3)
	1-<6	8,075	535 (6.6)	40 (7.5)	231 (43.2)	44 (8.2)	275 (51.4)
	6-<12	3,672	181 (4.9)	12 (6.6)	110 (60.8)	13 (7.2)	123 (68.0)
	12-<24	2,832	158 (5.6)	<10 [6]	97 (61.4)	14 (8.9)	111 (70.3)
	24+	2,624	138 (5.3)	<10 [6]	95 (68.8)	<10 [6]	102 (73.9)
2018/19	<1	4,801	335 (7.0)	15 (4.5)	160 (47.8)	17 (5.1)	177 (52.8)
	1-<6	7,742	561 (7.2)	30 (5.3)	311 (55.4)	41 (7.3)	352 (62.7)
	6-<12	3,616	197 (5.4)	18 (9.1)	136 (69.0)	10 (5.1)	146 (74.1)
	12-<24	3,447	182 (5.3)	<10 [6]	126 (69.2)	14 (7.7)	140 (76.9)
	24+	2,493	136 (5.5)	<10 [6]	102 (75.0)	<10 [6]	105 (77.2)
2019/20	<1	5,745	436 (7.6)	18 (4.1)	200 (45.9)	28 (6.4)	228 (52.3)
	1-<6	9,697	751 (7.7)	33 (4.4)	377 (50.2)	73 (9.7)	450 (59.9)
	6-<12	5,090	326 (6.4)	14 (4.3)	185 (56.7)	54 (16.6)	239 (73.3)
	12-<24	3,244	171 (5.3)	<10 [6]	120 (70.2)	14 (8.2)	134 (78.4)
	24+	2,035	112 (5.5)	<10 [6]	90 (80.4)	<10 [6]	94 (83.9)
**Ethnic Group**							
2017/18	White	15,638	1,077 (6.9)	76 (7.1)	533 (49.5)	79 (7.3)	612 (56.8)
	Mixed	431	30 (7.0)	<10 [6]	19 (63.3)	<10 [6]	19 (63.3)
	Asian or Asian British	813	23 (2.8)	<10 [6]	<10 [6]	<10 [6]	<10 [6]
	Black or Black British	404	29 (7.2)	<10 [6]	<10 [6]	<10 [6]	16 (55.2)
	Chinese and Other	214	19 (8.9)	<10 [6]	<10 [6]	<10 [6]	11 (57.9)
	Unclassified	372	<10 [6]	<10 [6]	<10 [6]	<10 [6]	<10 [6]
2018/19	White	14,911	1,176 (7.9)	69 (5.9)	716 (60.9)	74 (6.3)	790 (67.2)
	Mixed	371	34 (9.2)	<10 [6]	17 (50.0)	<10 [6]	22 (64.7)
	Asian or Asian British	726	23 (3.2)	<10 [6]	13 (56.5)	<10 [6]	13 (56.5)
	Black or Black British	364	22 (6.0)	<10 [6]	15 (68.2)	<10 [6]	16 (72.7)
	Chinese and Other	167	19 (11.4)	<10 [6]	<10 [6]	<10 [6]	<10 [6]
	Unclassified	409	<10 [6]	<10 [6]	<10 [6]	<10 [6]	<10 [6]
2019/20	White	16,606	1,482 (8.9)	58 (3.9)	820 (55.3)	162 (10.9)	982 (66.3)
	Mixed	409	29 (7.1)	<10 [6]	18 (62.1)	<10 [6]	22 (75.9)
	Asian or Asian British	755	18 (2.4)	<10 [6]	14 (77.8)	<10 [6]	14 (77.8)
	Black or Black British	451	29 (6.4)	<10 [6]	18 (62.1)	<10 [6]	22 (75.9)
	Chinese and Other	163	20 (12.3)	<10 [6]	<10 [6]	<10 [6]	<10 [6]
	Unclassified	387	<10 [6]	<10 [6]	<10 [6]	<10 [6]	<10 [6]

[1]Eligible for indicator; [2]Achieve in current prison; [3]Achieve in previous prison; [4]Overall achievement - either current or previous prison; [5]Declined indicator; [6]Suppressed (<10) to avoid disclosure

Indicator ***The proportion of women eligible for screening and aged 50-64 years who have had cervical screening in the preceding five years and six months***

Community achievement 2019/20: 77.64% (QOF 19/20)

Group: Screening

	Variable	Population	Eligible[1] (% popln)	Declined[5] (% eligible)	Satisfy[2] (% eligible)	Elsewhere[3] (% eligible)	Achieve[4] (% eligible)
**Year**							
	2017/18	21,677	215 (1.0)	16 (7.4)	97 (45.1)	20 (9.3)	117 (54.4)
	2018/19	22,099	243 (1.1)	25 (10.3)	117 (48.1)	21 (8.6)	138 (56.8)
	2019/20	25,811	259 (1.0)	18 (6.9)	129 (49.8)	30 (11.6)	159 (61.4)
**Prison**							
2017/18	Prison 1	1,323	<10 [6]	<10 [6]	<10 [6]	<10 [6]	<10 [6]
	Prison 2	3,261	<10 [6]	<10 [6]	<10 [6]	<10 [6]	<10 [6]
	Prison 3	2,623	<10 [6]	<10 [6]	<10 [6]	<10 [6]	<10 [6]
	Prison 4	2,089	<10 [6]	<10 [6]	<10 [6]	<10 [6]	<10 [6]
	Prison 5	637	<10 [6]	<10 [6]	<10 [6]	<10 [6]	<10 [6]
	Prison 6	1,552	<10 [6]	<10 [6]	<10 [6]	<10 [6]	<10 [6]
	Prison 7	635	68 (10.7)	<10 [6]	30 (44.1)	10 (14.7)	40 (58.8)
	Prison 8	1,085	146 (13.5)	10 (6.8)	67 (45.9)	10 (6.8)	77 (52.7)
	Prison 9	981	<10 [6]	<10 [6]	<10 [6]	<10 [6]	<10 [6]
	Prison 10	2,523	<10 [6]	<10 [6]	<10 [6]	<10 [6]	<10 [6]
	Prison 11	3,470	<10 [6]	<10 [6]	<10 [6]	<10 [6]	<10 [6]
	Prison 12	815	<10 [6]	<10 [6]	<10 [6]	<10 [6]	<10 [6]
	Prison 13	683	<10 [6]	<10 [6]	<10 [6]	<10 [6]	<10 [6]
2018/19	Prison 1	1,333	<10 [6]	<10 [6]	<10 [6]	<10 [6]	<10 [6]
	Prison 2	2,705	<10 [6]	<10 [6]	<10 [6]	<10 [6]	<10 [6]
	Prison 3	2,522	<10 [6]	<10 [6]	<10 [6]	<10 [6]	<10 [6]
	Prison 4	2,349	<10 [6]	<10 [6]	<10 [6]	<10 [6]	<10 [6]
	Prison 5	676	<10 [6]	<10 [6]	<10 [6]	<10 [6]	<10 [6]
	Prison 6	1,513	<10 [6]	<10 [6]	<10 [6]	<10 [6]	<10 [6]
	Prison 7	654	85 (13.0)	11 (12.9)	34 (40.0)	15 (17.6)	49 (57.6)
	Prison 8	1,148	157 (13.7)	14 (8.9)	83 (52.9)	<10 [6]	89 (56.7)
	Prison 9	996	<10 [6]	<10 [6]	<10 [6]	<10 [6]	<10 [6]
	Prison 10	2,717	<10 [6]	<10 [6]	<10 [6]	<10 [6]	<10 [6]
	Prison 11	4,020	<10 [6]	<10 [6]	<10 [6]	<10 [6]	<10 [6]
	Prison 12	792	<10 [6]	<10 [6]	<10 [6]	<10 [6]	<10 [6]
	Prison 13	674	<10 [6]	<10 [6]	<10 [6]	<10 [6]	<10 [6]
2019/20	Prison 1	1,410	<10 [6]	<10 [6]	<10 [6]	<10 [6]	<10 [6]
	Prison 2	2,979	<10 [6]	<10 [6]	<10 [6]	<10 [6]	<10 [6]
	Prison 3	2,809	<10 [6]	<10 [6]	<10 [6]	<10 [6]	<10 [6]
	Prison 4	2,651	<10 [6]	<10 [6]	<10 [6]	<10 [6]	<10 [6]
	Prison 5	616	<10 [6]	<10 [6]	<10 [6]	<10 [6]	<10 [6]
	Prison 6	1,533	<10 [6]	<10 [6]	<10 [6]	<10 [6]	<10 [6]
	Prison 7	860	94 (10.9)	<10 [6]	28 (29.8)	25 (26.6)	53 (56.4)
	Prison 8	1,385	164 (11.8)	12 (7.3)	101 (61.6)	<10 [6]	106 (64.6)
	Prison 9	1,092	<10 [6]	<10 [6]	<10 [6]	<10 [6]	<10 [6]
	Prison 10	3,577	<10 [6]	<10 [6]	<10 [6]	<10 [6]	<10 [6]
	Prison 11	5,348	<10 [6]	<10 [6]	<10 [6]	<10 [6]	<10 [6]
	Prison 12	805	<10 [6]	<10 [6]	<10 [6]	<10 [6]	<10 [6]
	Prison 13	746	<10 [6]	<10 [6]	<10 [6]	<10 [6]	<10 [6]
**Prison category**							
2017/18	A	1,664	<10 [6]	<10 [6]	<10 [6]	<10 [6]	<10 [6]
	B	9,254	<10 [6]	<10 [6]	<10 [6]	<10 [6]	<10 [6]
	C	6,035	<10 [6]	<10 [6]	<10 [6]	<10 [6]	<10 [6]
	Closed	1,720	214 (12.4)	16 (7.5)	97 (45.3)	20 (9.3)	117 (54.7)
	D	2,189	<10 [6]	<10 [6]	<10 [6]	<10 [6]	<10 [6]
	YOI	815	<10 [6]	<10 [6]	<10 [6]	<10 [6]	<10 [6]
2018/19	A	1,670	<10 [6]	<10 [6]	<10 [6]	<10 [6]	<10 [6]
	B	9,442	<10 [6]	<10 [6]	<10 [6]	<10 [6]	<10 [6]
	C	6,204	<10 [6]	<10 [6]	<10 [6]	<10 [6]	<10 [6]
	Closed	1,802	242 (13.4)	25 (10.3)	117 (48.3)	21 (8.7)	138 (57.0)
	D	2,189	<10 [6]	<10 [6]	<10 [6]	<10 [6]	<10 [6]
	YOI	792	<10 [6]	<10 [6]	<10 [6]	<10 [6]	<10 [6]
2019/20	A	1,838	<10 [6]	<10 [6]	<10 [6]	<10 [6]	<10 [6]
	B	11,904	<10 [6]	<10 [6]	<10 [6]	<10 [6]	<10 [6]
	C	6,870	<10 [6]	<10 [6]	<10 [6]	<10 [6]	<10 [6]
	Closed	2,245	258 (11.5)	18 (7.0)	129 (50.0)	30 (11.6)	159 (61.6)
	D	2,149	<10 [6]	<10 [6]	<10 [6]	<10 [6]	<10 [6]
	YOI	805	<10 [6]	<10 [6]	<10 [6]	<10 [6]	<10 [6]
**Gender**							
2017/18	F	1,699	215 (12.7)	16 (7.4)	97 (45.1)	20 (9.3)	117 (54.4)
	M	19,977	0 (0.0)	0 (0.0)	0 (0.0)	0 (0.0)	0 (0.0)
2018/19	F	1,802	243 (13.5)	25 (10.3)	117 (48.1)	21 (8.6)	138 (56.8)
	M	20,295	0 (0.0)	0 (0.0)	0 (0.0)	0 (0.0)	0 (0.0)
2019/20	F	1,376	164 (11.9)	12 (7.3)	101 (61.6)	5 (3.0)	106 (64.6)
	M	23,570	0 (0.0)	0 (0.0)	0 (0.0)	0 (0.0)	0 (0.0)

[1]Eligible for indicator; [2]Achieve in current prison; [3]Achieve in previous prison; [4]Overall achievement - either current or previous prison; [5]Declined indicator; [6]Suppressed (<10) to avoid disclosure

Indicator	*The proportion of women eligible for screening and aged 50-64 years who have had cervical screening in the preceding five years and six months*						*Community achievement 2019/20: 77.64% (QOF 19/20)*
	**Group: Screening**						
	**Variable**	**Population**	**Eligible[1] (% popln)**	**Declined[5] (% eligible)**	**Satisfy[2] (% eligible)**	**Elsewhere[3] (% eligible)**	**Achieve[4] (% eligible)**
**Sentence Status**							
2017/18	.	81	<10 [6]	<10 [6]	<10 [6]	<10 [6]	<10 [6]
	Absconded	<10 [6]	<10 [6]	<10 [6]	<10 [6]	<10 [6]	<10 [6]
	Active In	15,872	192 (1.2)	15 (7.8)	86 (44.8)	19 (9.9)	105 (54.7)
	Active Out	1,052	17 (1.6)	<10 [6]	<10 [6]	<10 [6]	10 (58.8)
	Convicted Sentence	2,125	<10 [6]	<10 [6]	<10 [6]	<10 [6]	<10 [6]
	Downgrade in security category	<10 [6]	<10 [6]	<10 [6]	<10 [6]	<10 [6]	<10 [6]
	Internal Cell Move	<10 [6]	<10 [6]	<10 [6]	<10 [6]	<10 [6]	<10 [6]
	Judges Remand	92	<10 [6]	<10 [6]	<10 [6]	<10 [6]	<10 [6]
	Licence Revoke	52	<10 [6]	<10 [6]	<10 [6]	<10 [6]	<10 [6]
	On Remand	1,492	<10 [6]	<10 [6]	<10 [6]	<10 [6]	<10 [6]
	Transfer	910	<10 [6]	<10 [6]	<10 [6]	<10 [6]	<10 [6]
	Upgrade in security category	<10 [6]	<10 [6]	<10 [6]	<10 [6]	<10 [6]	<10 [6]
2018/19	.	88	<10 [6]	<10 [6]	<10 [6]	<10 [6]	<10 [6]
	Absconded	<10 [6]	<10 [6]	<10 [6]	<10 [6]	<10 [6]	<10 [6]
	Active In	18,145	207 (1.1)	22 (10.6)	101 (48.8)	15 (7.2)	116 (56.0)
	Active Out	835	22 (2.6)	<10 [6]	<10 [6]	<10 [6]	13 (59.1)
	Convicted Sentence	1,320	12 (0.9)	<10 [6]	<10 [6]	<10 [6]	<10 [6]
	Downgrade in security category	<10 [6]	<10 [6]	<10 [6]	<10 [6]	<10 [6]	<10 [6]
	Internal Cell Move	<10 [6]	<10 [6]	<10 [6]	<10 [6]	<10 [6]	<10 [6]
	Judges Remand	<10 [6]	<10 [6]	<10 [6]	<10 [6]	<10 [6]	<10 [6]
	Licence Revoke	125	<10 [6]	<10 [6]	<10 [6]	<10 [6]	<10 [6]
	On Remand	1,059	<10 [6]	<10 [6]	<10 [6]	<10 [6]	<10 [6]
	Transfer	518	<10 [6]	<10 [6]	<10 [6]	<10 [6]	<10 [6]
	Upgrade in security category	<10 [6]	<10 [6]	<10 [6]	<10 [6]	<10 [6]	<10 [6]
2019/20	.	69	<10 [6]	<10 [6]	<10 [6]	<10 [6]	<10 [6]
	Absconded	<10 [6]	<10 [6]	<10 [6]	<10 [6]	<10 [6]	<10 [6]
	Active In	22,424	243 (1.1)	16 (6.6)	123 (50.6)	29 (11.9)	152 (62.6)
	Active Out	625	13 (2.1)	<10 [6]	<10 [6]	<10 [6]	<10 [6]
	Convicted Sentence	1,361	<10 [6]	<10 [6]	<10 [6]	<10 [6]	<10 [6]
	Downgrade in security category	<10 [6]	<10 [6]	<10 [6]	<10 [6]	<10 [6]	<10 [6]
	Internal Cell Move	<10 [6]	<10 [6]	<10 [6]	<10 [6]	<10 [6]	<10 [6]
	Judges Remand	19	<10 [6]	<10 [6]	<10 [6]	<10 [6]	<10 [6]
	Licence Revoke	178	<10 [6]	<10 [6]	<10 [6]	<10 [6]	<10 [6]
	On Remand	1,031	<10 [6]	<10 [6]	<10 [6]	<10 [6]	<10 [6]
	Transfer	101	<10 [6]	<10 [6]	<10 [6]	<10 [6]	<10 [6]
	Upgrade in security category	<10 [6]	<10 [6]	<10 [6]	<10 [6]	<10 [6]	<10 [6]
**Age - years**							
2017/18	10 - <20	468	<10 [6]	<10 [6]	<10 [6]	<10 [6]	<10 [6]
	20 - <30	6,994	<10 [6]	<10 [6]	<10 [6]	<10 [6]	<10 [6]
	30 - <40	7,051	<10 [6]	<10 [6]	<10 [6]	<10 [6]	<10 [6]
	40 - <50	4,114	<10 [6]	<10 [6]	<10 [6]	<10 [6]	<10 [6]
	50 - <60	2,107	178 (8.4)	11 (6.2)	79 (44.4)	17 (9.6)	96 (53.9)
	60 - <70	684	37 (5.4)	<10 [6]	18 (48.6)	<10 [6]	21 (56.8)
	70 - <80	213	<10 [6]	<10 [6]	<10 [6]	<10 [6]	<10 [6]
	80 - <90	40	<10 [6]	<10 [6]	<10 [6]	<10 [6]	<10 [6]
	90 - <100	<10 [6]	<10 [6]	<10 [6]	<10 [6]	<10 [6]	<10 [6]
	100 - <110	<10 [6]	<10 [6]	<10 [6]	<10 [6]	<10 [6]	<10 [6]
2018/19	10 - <20	436	<10 [6]	<10 [6]	<10 [6]	<10 [6]	<10 [6]
	20 - <30	7,163	<10 [6]	<10 [6]	<10 [6]	<10 [6]	<10 [6]
	30 - <40	7,381	<10 [6]	<10 [6]	<10 [6]	<10 [6]	<10 [6]
	40 - <50	4,180	<10 [6]	<10 [6]	<10 [6]	<10 [6]	<10 [6]
	50 - <60	1,978	209 (10.6)	20 (9.6)	106 (50.7)	18 (8.6)	124 (59.3)
	60 - <70	701	34 (4.9)	<10 [6]	11 (32.4)	<10 [6]	14 (41.2)
	70 - <80	209	<10 [6]	<10 [6]	<10 [6]	<10 [6]	<10 [6]
	80 - <90	45	<10 [6]	<10 [6]	<10 [6]	<10 [6]	<10 [6]
	90 - <100	<10 [6]	<10 [6]	<10 [6]	<10 [6]	<10 [6]	<10 [6]
	100 - <110	<10 [6]	<10 [6]	<10 [6]	<10 [6]	<10 [6]	<10 [6]
2019/20	10 - <20	404	<10 [6]	<10 [6]	<10 [6]	<10 [6]	<10 [6]
	20 - <30	8,064	<10 [6]	<10 [6]	<10 [6]	<10 [6]	<10 [6]
	30 - <40	9,125	<10 [6]	<10 [6]	<10 [6]	<10 [6]	<10 [6]
	40 - <50	4,948	<10 [6]	<10 [6]	<10 [6]	<10 [6]	<10 [6]
	50 - <60	2,224	225 (10.1)	14 (6.2)	117 (52.0)	23 (10.2)	140 (62.2)
	60 - <70	751	34 (4.5)	<10 [6]	12 (35.3)	<10 [6]	19 (55.9)
	70 - <80	238	<10 [6]	<10 [6]	<10 [6]	<10 [6]	<10 [6]
	80 - <90	53	<10 [6]	<10 [6]	<10 [6]	<10 [6]	<10 [6]
	90 - <100	<10 [6]	<10 [6]	<10 [6]	<10 [6]	<10 [6]	<10 [6]
	100 - <110	<10 [6]	<10 [6]	<10 [6]	<10 [6]	<10 [6]	<10 [6]

[1]Eligible for indicator; [2]Achieve in current prison; [3]Achieve in previous prison; [4]Overall achievement - either current or previous prison; [5]Declined indicator; [6]Suppressed (<10) to avoid disclosure

Indicator	*The proportion of women eligible for screening and aged 50-64 years who have had cervical screening in the preceding five years and six months*						*Community achievement 2019/20: 77.64% (QOF 19/20)*
	**Group: Screening**						
	**Variable**	**Population**	**Eligible[1] (% popln)**	**Declined[5] (% eligible)**	**Satisfy[2] (% eligible)**	**Elsewhere[3] (% eligible)**	**Achieve[4] (% eligible)**
**Length of Stay (months)**							
2017/18	<1	4,474	32 (0.7)	<10 [6]	<10 [6]	<10 [6]	11 (34.4)
	1-<6	8,075	88 (1.1)	<10 [6]	45 (51.1)	<10 [6]	48 (54.5)
	6-<12	3,672	36 (1.0)	<10 [6]	12 (33.3)	<10 [6]	16 (44.4)
	12-<24	2,832	15 (0.5)	<10 [6]	11 (73.3)	<10 [6]	11 (73.3)
	24+	2,624	44 (1.7)	<10 [6]	21 (47.7)	10 (22.7)	31 (70.5)
2018/19	<1	4,801	41 (0.9)	<10 [6]	17 (41.5)	<10 [6]	18 (43.9)
	1-<6	7,742	98 (1.3)	10 (10.2)	51 (52.0)	<10 [6]	56 (57.1)
	6-<12	3,616	41 (1.1)	<10 [6]	20 (48.8)	<10 [6]	26 (63.4)
	12-<24	3,447	17 (0.5)	<10 [6]	<10 [6]	<10 [6]	<10 [6]
	24+	2,493	46 (1.8)	<10 [6]	22 (47.8)	<10 [6]	29 (63.0)
2019/20	<1	5,745	50 (0.9)	<10 [6]	24 (48.0)	<10 [6]	31 (62.0)
	1-<6	9,697	107 (1.1)	<10 [6]	55 (51.4)	<10 [6]	64 (59.8)
	6-<12	5,090	51 (1.0)	<10 [6]	25 (49.0)	<10 [6]	34 (66.7)
	12-<24	3,244	16 (0.5)	<10 [6]	<10 [6]	<10 [6]	<10 [6]
	24+	2,035	35 (1.7)	<10 [6]	19 (54.3)	<10 [6]	22 (62.9)
**Ethnic Group**							
2017/18	White	15,638	191 (1.2)	15 (7.9)	87 (45.5)	19 (9.9)	106 (55.5)
	Mixed	431	<10 [6]	<10 [6]	<10 [6]	<10 [6]	<10 [6]
	Asian or Asian British	813	<10 [6]	<10 [6]	<10 [6]	<10 [6]	<10 [6]
	Black or Black British	404	<10 [6]	<10 [6]	<10 [6]	<10 [6]	<10 [6]
	Chinese and Other	214	<10 [6]	<10 [6]	<10 [6]	<10 [6]	<10 [6]
	Unclassified	372	<10 [6]	<10 [6]	<10 [6]	<10 [6]	<10 [6]
2018/19	White	14,911	218 (1.5)	22 (10.1)	108 (49.5)	21 (9.6)	129 (59.2)
	Mixed	371	<10 [6]	<10 [6]	<10 [6]	<10 [6]	<10 [6]
	Asian or Asian British	726	<10 [6]	<10 [6]	<10 [6]	<10 [6]	<10 [6]
	Black or Black British	364	<10 [6]	<10 [6]	<10 [6]	<10 [6]	<10 [6]
	Chinese and Other	167	<10 [6]	<10 [6]	<10 [6]	<10 [6]	<10 [6]
	Unclassified	409	<10 [6]	<10 [6]	<10 [6]	<10 [6]	<10 [6]
2019/20	White	16,606	218 (1.3)	15 (6.9)	111 (50.9)	27 (12.4)	138 (63.3)
	Mixed	409	<10 [6]	<10 [6]	<10 [6]	<10 [6]	<10 [6]
	Asian or Asian British	755	<10 [6]	<10 [6]	<10 [6]	<10 [6]	<10 [6]
	Black or Black British	451	<10 [6]	<10 [6]	<10 [6]	<10 [6]	<10 [6]
	Chinese and Other	163	<10 [6]	<10 [6]	<10 [6]	<10 [6]	<10 [6]
	Unclassified	387	<10 [6]	<10 [6]	<10 [6]	<10 [6]	<10 [6]

[1]Eligible for indicator; [2]Achieve in current prison; [3]Achieve in previous prison; [4]Overall achievement - either current or previous prison; [5]Declined indicator; [6]Suppressed (<10) to avoid disclosure

Indicator ***The proportion of women eligible for screening and aged 50-70 years who have had breast screening in the preceding three years***

no comparable data in 2019/20. NHS Digital reports that proportion of women (50-70) taking up routine breast screening invitations was 70.5% in 2017-18

Group: Screening

	Variable	Population	Eligible[1] (% popln)	Declined[5] (% eligible)	Satisfy[2] (% eligible)	Elsewhere[3] (% eligible)	Achieve[4] (% eligible)
**Year**							
	2017/18	21,677	157 (0.7)	<10 [6]	42 (26.8)	<10 [6]	45 (28.7)
	2018/19	22,099	162 (0.7)	<10 [6]	48 (29.6)	<10 [6]	51 (31.5)
	2019/20	25,811	179 (0.7)	<10 [6]	53 (29.6)	<10 [6]	59 (33.0)
**Prison**							
2017/18	Prison 1	1,323	<10 [6]	<10 [6]	<10 [6]	<10 [6]	<10 [6]
	Prison 2	3,261	<10 [6]	<10 [6]	<10 [6]	<10 [6]	<10 [6]
	Prison 3	2,623	<10 [6]	<10 [6]	<10 [6]	<10 [6]	<10 [6]
	Prison 4	2,089	<10 [6]	<10 [6]	<10 [6]	<10 [6]	<10 [6]
	Prison 5	637	<10 [6]	<10 [6]	<10 [6]	<10 [6]	<10 [6]
	Prison 6	1,552	<10 [6]	<10 [6]	<10 [6]	<10 [6]	<10 [6]
	Prison 7	635	55 (8.7)	<10 [6]	27 (49.1)	<10 [6]	27 (49.1)
	Prison 8	1,085	102 (9.4)	<10 [6]	15 (14.7)	<10 [6]	18 (17.6)
	Prison 9	981	<10 [6]	<10 [6]	<10 [6]	<10 [6]	<10 [6]
	Prison 10	2,523	<10 [6]	<10 [6]	<10 [6]	<10 [6]	<10 [6]
	Prison 11	3,470	<10 [6]	<10 [6]	<10 [6]	<10 [6]	<10 [6]
	Prison 12	815	<10 [6]	<10 [6]	<10 [6]	<10 [6]	<10 [6]
	Prison 13	683	<10 [6]	<10 [6]	<10 [6]	<10 [6]	<10 [6]
2018/19	Prison 1	1,333	<10 [6]	<10 [6]	<10 [6]	<10 [6]	<10 [6]
	Prison 2	2,705	<10 [6]	<10 [6]	<10 [6]	<10 [6]	<10 [6]
	Prison 3	2,522	<10 [6]	<10 [6]	<10 [6]	<10 [6]	<10 [6]
	Prison 4	2,349	<10 [6]	<10 [6]	<10 [6]	<10 [6]	<10 [6]
	Prison 5	676	<10 [6]	<10 [6]	<10 [6]	<10 [6]	<10 [6]
	Prison 6	1,513	<10 [6]	<10 [6]	<10 [6]	<10 [6]	<10 [6]
	Prison 7	654	51 (7.8)	<10 [6]	22 (43.1)	<10 [6]	23 (45.1)
	Prison 8	1,148	110 (9.6)	<10 [6]	26 (23.6)	<10 [6]	28 (25.5)
	Prison 9	996	<10 [6]	<10 [6]	<10 [6]	<10 [6]	<10 [6]
	Prison 10	2,717	<10 [6]	<10 [6]	<10 [6]	<10 [6]	<10 [6]
	Prison 11	4,020	<10 [6]	<10 [6]	<10 [6]	<10 [6]	<10 [6]
	Prison 12	792	<10 [6]	<10 [6]	<10 [6]	<10 [6]	<10 [6]
	Prison 13	674	<10 [6]	<10 [6]	<10 [6]	<10 [6]	<10 [6]
2019/20	Prison 1	1,410	<10 [6]	<10 [6]	<10 [6]	<10 [6]	<10 [6]
	Prison 2	2,979	<10 [6]	<10 [6]	<10 [6]	<10 [6]	<10 [6]
	Prison 3	2,809	<10 [6]	<10 [6]	<10 [6]	<10 [6]	<10 [6]
	Prison 4	2,651	<10 [6]	<10 [6]	<10 [6]	<10 [6]	<10 [6]
	Prison 5	616	<10 [6]	<10 [6]	<10 [6]	<10 [6]	<10 [6]
	Prison 6	1,533	<10 [6]	<10 [6]	<10 [6]	<10 [6]	<10 [6]
	Prison 7	860	73 (8.5)	<10 [6]	23 (31.5)	<10 [6]	29 (39.7)
	Prison 8	1,385	106 (7.7)	<10 [6]	30 (28.3)	<10 [6]	30 (28.3)
	Prison 9	1,092	<10 [6]	<10 [6]	<10 [6]	<10 [6]	<10 [6]
	Prison 10	3,577	<10 [6]	<10 [6]	<10 [6]	<10 [6]	<10 [6]
	Prison 11	5,348	<10 [6]	<10 [6]	<10 [6]	<10 [6]	<10 [6]
	Prison 12	805	<10 [6]	<10 [6]	<10 [6]	<10 [6]	<10 [6]
	Prison 13	746	<10 [6]	<10 [6]	<10 [6]	<10 [6]	<10 [6]
**Prison category**							
2017/18	A	1,664	<10 [6]	<10 [6]	<10 [6]	<10 [6]	<10 [6]
	B	9,254	<10 [6]	<10 [6]	<10 [6]	<10 [6]	<10 [6]
	C	6,035	<10 [6]	<10 [6]	<10 [6]	<10 [6]	<10 [6]
	Closed	1,720	157 (9.1)	<10 [6]	42 (26.8)	<10 [6]	45 (28.7)
	D	2,189	<10 [6]	<10 [6]	<10 [6]	<10 [6]	<10 [6]
	YOI	815	<10 [6]	<10 [6]	<10 [6]	<10 [6]	<10 [6]
2018/19	A	1,670	<10 [6]	<10 [6]	<10 [6]	<10 [6]	<10 [6]
	B	9,442	<10 [6]	<10 [6]	<10 [6]	<10 [6]	<10 [6]
	C	6,204	<10 [6]	<10 [6]	<10 [6]	<10 [6]	<10 [6]
	Closed	1,802	161 (8.9)	<10 [6]	48 (29.8)	<10 [6]	51 (31.7)
	D	2,189	<10 [6]	<10 [6]	<10 [6]	<10 [6]	<10 [6]
	YOI	792	<10 [6]	<10 [6]	<10 [6]	<10 [6]	<10 [6]
2019/20	A	1,838	<10 [6]	<10 [6]	<10 [6]	<10 [6]	<10 [6]
	B	11,904	<10 [6]	<10 [6]	<10 [6]	<10 [6]	<10 [6]
	C	6,870	<10 [6]	<10 [6]	<10 [6]	<10 [6]	<10 [6]
	Closed	2,245	179 (8.0)	<10 [6]	53 (29.6)	<10 [6]	59 (33.0)
	D	2,149	<10 [6]	<10 [6]	<10 [6]	<10 [6]	<10 [6]
	YOI	805	<10 [6]	<10 [6]	<10 [6]	<10 [6]	<10 [6]
**Gender**							
2017/18	F	1,699	157 (9.2)	<10 [6]	42 (26.8)	<10 [6]	45 (28.7)
	M	19,977	0 (0.0)	<10 [6]	0 (0.0)	<10 [6]	0 (0.0)
2018/19	F	1,802	162 (9.0)	<10 [6]	48 (29.6)	<10 [6]	51 (31.5)
	M	20,295	0 (0.0)	<10 [6]	0 (0.0)	<10 [6]	0 (0.0)
2019/20	F	1,376	106 (7.7)	<10 [6]	30 (28.3)	<10 [6]	30 (28.3)
	M	23,570	0 (0.0)	<10 [6]	0 (0.0)	<10 [6]	0 (0.0)

[1]Eligible for indicator; [2]Achieve in current prison; [3]Achieve in previous prison; [4]Overall achievement - either current or previous prison; [5]Declined indicator; [6]Suppressed (<10) to avoid disclosure

Indicator		*The proportion of women eligible for screening and aged 50-70 years who have had breast screening in the preceding three years*					no comparable data in 2019/20. NHS Digital reports that proportion of women (50-70) taking up routine breast screening invitations was 70.5% in 2017-18
	**Group: Screening**						
	**Variable**	**Population**	**Eligible[1] (% popln)**	**Declined[5] (% eligible)**	**Satisfy[2] (% eligible)**	**Elsewhere[3] (% eligible)**	**Achieve[4] (% eligible)**
**Sentence Status**							
2017/18	.	81	<10 [6]	<10 [6]	<10 [6]	<10 [6]	<10 [6]
	Absconded	<10 [6]	<10 [6]	<10 [6]	<10 [6]	<10 [6]	<10 [6]
	Active In	15,872	146 (0.9)	<10 [6]	42 (28.8)	<10 [6]	45 (30.8)
	Active Out	1,052	<10 [6]	<10 [6]	<10 [6]	<10 [6]	<10 [6]
	Convicted Sentence	2,125	<10 [6]	<10 [6]	<10 [6]	<10 [6]	<10 [6]
	Downgrade in security category	<10 [6]	<10 [6]	<10 [6]	<10 [6]	<10 [6]	<10 [6]
	Internal Cell Move	<10 [6]	<10 [6]	<10 [6]	<10 [6]	<10 [6]	<10 [6]
	Judges Remand	92	<10 [6]	<10 [6]	<10 [6]	<10 [6]	<10 [6]
	Licence Revoke	52	<10 [6]	<10 [6]	<10 [6]	<10 [6]	<10 [6]
	On Remand	1,492	<10 [6]	<10 [6]	<10 [6]	<10 [6]	<10 [6]
	Transfer	910	<10 [6]	<10 [6]	<10 [6]	<10 [6]	<10 [6]
	Upgrade in security category	<10 [6]	<10 [6]	<10 [6]	<10 [6]	<10 [6]	<10 [6]
2018/19	.	88	<10 [6]	<10 [6]	<10 [6]	<10 [6]	<10 [6]
	Absconded	<10 [6]	<10 [6]	<10 [6]	<10 [6]	<10 [6]	<10 [6]
	Active In	18,145	142 (0.8)	<10 [6]	45 (31.7)	<10 [6]	48 (33.8)
	Active Out	835	13 (1.6)	<10 [6]	<10 [6]	<10 [6]	<10 [6]
	Convicted Sentence	1,320	<10 [6]	<10 [6]	<10 [6]	<10 [6]	<10 [6]
	Downgrade in security category	<10 [6]	<10 [6]	<10 [6]	<10 [6]	<10 [6]	<10 [6]
	Internal Cell Move	<10 [6]	<10 [6]	<10 [6]	<10 [6]	<10 [6]	<10 [6]
	Judges Remand	<10 [6]	<10 [6]	<10 [6]	<10 [6]	<10 [6]	<10 [6]
	Licence Revoke	125	<10 [6]	<10 [6]	<10 [6]	<10 [6]	<10 [6]
	On Remand	1,059	<10 [6]	<10 [6]	<10 [6]	<10 [6]	<10 [6]
	Transfer	518	<10 [6]	<10 [6]	<10 [6]	<10 [6]	<10 [6]
	Upgrade in security category	<10 [6]	<10 [6]	<10 [6]	<10 [6]	<10 [6]	<10 [6]
2019/20	.	69	<10 [6]	<10 [6]	<10 [6]	<10 [6]	<10 [6]
	Absconded	<10 [6]	<10 [6]	<10 [6]	<10 [6]	<10 [6]	<10 [6]
	Active In	22,424	168 (0.7)	<10 [6]	50 (29.8)	<10 [6]	56 (33.3)
	Active Out	625	<10 [6]	<10 [6]	<10 [6]	<10 [6]	<10 [6]
	Convicted Sentence	1,361	<10 [6]	<10 [6]	<10 [6]	<10 [6]	<10 [6]
	Downgrade in security category	<10 [6]	<10 [6]	<10 [6]	<10 [6]	<10 [6]	<10 [6]
	Internal Cell Move	<10 [6]	<10 [6]	<10 [6]	<10 [6]	<10 [6]	<10 [6]
	Judges Remand	19	<10 [6]	<10 [6]	<10 [6]	<10 [6]	<10 [6]
	Licence Revoke	178	<10 [6]	<10 [6]	<10 [6]	<10 [6]	<10 [6]
	On Remand	1,031	<10 [6]	<10 [6]	<10 [6]	<10 [6]	<10 [6]
	Transfer	101	<10 [6]	<10 [6]	<10 [6]	<10 [6]	<10 [6]
	Upgrade in security category	<10 [6]	<10 [6]	<10 [6]	<10 [6]	<10 [6]	<10 [6]
**Age - years**							
2017/18	10 - <20	468	<10 [6]	<10 [6]	<10 [6]	<10 [6]	<10 [6]
	20 - <30	6,994	<10 [6]	<10 [6]	<10 [6]	<10 [6]	<10 [6]
	30 - <40	7,051	<10 [6]	<10 [6]	<10 [6]	<10 [6]	<10 [6]
	40 - <50	4,114	<10 [6]	<10 [6]	<10 [6]	<10 [6]	<10 [6]
	50 - <60	2,107	111 (5.3)	<10 [6]	33 (29.7)	<10 [6]	36 (32.4)
	60 - <70	684	44 (6.4)	<10 [6]	<10 [6]	<10 [6]	<10 [6]
	70 - <80	213	<10 [6]	<10 [6]	<10 [6]	<10 [6]	<10 [6]
	80 - <90	40	<10 [6]	<10 [6]	<10 [6]	<10 [6]	<10 [6]
	90 - <100	<10 [6]	<10 [6]	<10 [6]	<10 [6]	<10 [6]	<10 [6]
	100 - <110	<10 [6]	<10 [6]	<10 [6]	<10 [6]	<10 [6]	<10 [6]
2018/19	10 - <20	436	<10 [6]	<10 [6]	<10 [6]	<10 [6]	<10 [6]
	20 - <30	7,163	<10 [6]	<10 [6]	<10 [6]	<10 [6]	<10 [6]
	30 - <40	7,381	<10 [6]	<10 [6]	<10 [6]	<10 [6]	<10 [6]
	40 - <50	4,180	<10 [6]	<10 [6]	<10 [6]	<10 [6]	<10 [6]
	50 - <60	1,978	120 (6.1)	<10 [6]	37 (30.8)	<10 [6]	40 (33.3)
	60 - <70	701	40 (5.7)	<10 [6]	11 (27.5)	<10 [6]	11 (27.5)
	70 - <80	209	<10 [6]	<10 [6]	<10 [6]	<10 [6]	<10 [6]
	80 - <90	45	<10 [6]	<10 [6]	<10 [6]	<10 [6]	<10 [6]
	90 - <100	<10 [6]	<10 [6]	<10 [6]	<10 [6]	<10 [6]	<10 [6]
	100 - <110	<10 [6]	<10 [6]	<10 [6]	<10 [6]	<10 [6]	<10 [6]
2019/20	10 - <20	404	<10 [6]	<10 [6]	<10 [6]	<10 [6]	<10 [6]
	20 - <30	8,064	<10 [6]	<10 [6]	<10 [6]	<10 [6]	<10 [6]
	30 - <40	9,125	<10 [6]	<10 [6]	<10 [6]	<10 [6]	<10 [6]
	40 - <50	4,948	<10 [6]	<10 [6]	<10 [6]	<10 [6]	<10 [6]
	50 - <60	2,224	131 (5.9)	<10 [6]	44 (33.6)	<10 [6]	48 (36.6)
	60 - <70	751	46 (6.1)	<10 [6]	<10 [6]	<10 [6]	11 (23.9)
	70 - <80	238	<10 [6]	<10 [6]	<10 [6]	<10 [6]	<10 [6]
	80 - <90	53	<10 [6]	<10 [6]	<10 [6]	<10 [6]	<10 [6]
	90 - <100	<10 [6]	<10 [6]	<10 [6]	<10 [6]	<10 [6]	<10 [6]
	100 - <110	<10 [6]	<10 [6]	<10 [6]	<10 [6]	<10 [6]	<10 [6]

[1]Eligible for indicator; [2]Achieve in current prison; [3]Achieve in previous prison; [4]Overall achievement - either current or previous prison; [5]Declined indicator; [6]Suppressed (<10) to avoid disclosure

Indicator		The proportion of women eligible for screening and aged 50-70 years who have had breast screening in the preceding three years					no comparable data in 2019/20. NHS Digital reports that proportion of women (50-70) taking up routine breast screening invitations was 70.5% in 2017-18
	**Group: Screening Variable**	**Population**	**Eligible[1] (% popln)**	**Declined[5] (% eligible)**	**Satisfy[2] (% eligible)**	**Elsewhere[3] (% eligible)**	**Achieve[4] (% eligible)**
**Length of Stay (months)**							
2017/18	<1	4,474	23 (0.5)	<10 [6]	<10 [6]	<10 [6]	<10 [6]
	1-<6	8,075	60 (0.7)	<10 [6]	12 (20.0)	<10 [6]	13 (21.7)
	6-<12	3,672	28 (0.8)	<10 [6]	<10 [6]	<10 [6]	10 (35.7)
	12-<24	2,832	10 (0.4)	<10 [6]	<10 [6]	<10 [6]	<10 [6]
	24+	2,624	36 (1.4)	<10 [6]	18 (50.0)	<10 [6]	19 (52.8)
2018/19	<1	4,801	27 (0.6)	<10 [6]	<10 [6]	<10 [6]	<10 [6]
	1-<6	7,742	58 (0.7)	<10 [6]	17 (29.3)	<10 [6]	17 (29.3)
	6-<12	3,616	31 (0.9)	<10 [6]	<10 [6]	<10 [6]	10 (32.3)
	12-<24	3,447	11 (0.3)	<10 [6]	<10 [6]	<10 [6]	<10 [6]
	24+	2,493	35 (1.4)	<10 [6]	14 (40.0)	<10 [6]	14 (40.0)
2019/20	<1	5,745	33 (0.6)	<10 [6]	<10 [6]	<10 [6]	10 (30.3)
	1-<6	9,697	69 (0.7)	<10 [6]	19 (27.5)	<10 [6]	21 (30.4)
	6-<12	5,090	34 (0.7)	<10 [6]	10 (29.4)	<10 [6]	13 (38.2)
	12-<24	3,244	14 (0.4)	<10 [6]	<10 [6]	<10 [6]	<10 [6]
	24+	2,035	29 (1.4)	<10 [6]	10 (34.5)	<10 [6]	10 (34.5)
**Ethnic Group**							
2017/18	White	15,638	141 (0.9)	<10 [6]	38 (27.0)	<10 [6]	41 (29.1)
	Mixed	431	<10 [6]	<10 [6]	<10 [6]	<10 [6]	<10 [6]
	Asian or Asian British	813	<10 [6]	<10 [6]	<10 [6]	<10 [6]	<10 [6]
	Black or Black British	404	<10 [6]	<10 [6]	<10 [6]	<10 [6]	<10 [6]
	Chinese and Other	214	<10 [6]	<10 [6]	<10 [6]	<10 [6]	<10 [6]
	Unclassified	372	<10 [6]	<10 [6]	<10 [6]	<10 [6]	<10 [6]
2018/19	White	14,911	144 (1.0)	<10 [6]	42 (29.2)	<10 [6]	45 (31.3)
	Mixed	371	<10 [6]	<10 [6]	<10 [6]	<10 [6]	<10 [6]
	Asian or Asian British	726	<10 [6]	<10 [6]	<10 [6]	<10 [6]	<10 [6]
	Black or Black British	364	<10 [6]	<10 [6]	<10 [6]	<10 [6]	<10 [6]
	Chinese and Other	167	<10 [6]	<10 [6]	<10 [6]	<10 [6]	<10 [6]
	Unclassified	409	<10 [6]	<10 [6]	<10 [6]	<10 [6]	<10 [6]
2019/20	White	16,606	156 (0.9)	<10 [6]	48 (30.8)	<10 [6]	52 (33.3)
	Mixed	409	<10 [6]	<10 [6]	<10 [6]	<10 [6]	<10 [6]
	Asian or Asian British	755	<10 [6]	<10 [6]	<10 [6]	<10 [6]	<10 [6]
	Black or Black British	451	<10 [6]	<10 [6]	<10 [6]	<10 [6]	<10 [6]
	Chinese and Other	163	<10 [6]	<10 [6]	<10 [6]	<10 [6]	<10 [6]
	Unclassified	387	<10 [6]	<10 [6]	<10 [6]	<10 [6]	<10 [6]

[1]Eligible for indicator; [2]Achieve in current prison; [3]Achieve in previous prison; [4]Overall achievement - either current or previous prison; [5]Declined indicator; [6]Suppressed (<10) to avoid disclosure

Indicator		The proportion of men eligible for screening and aged 65 years or over with a record of abdominal aortic aneurysm screening					no comparable data in QOF 2019/20. PHE source states initial uptake of screening in 2014/15 was 79.5%
**Group: Screening**	**Variable**	**Population**	**Eligible[1] (% popln)**	**Declined[5] (% eligible)**	**Satisfy[2] (% eligible)**	**Elsewhere[3] (% eligible)**	**Achieve[4] (% eligible)**
**Year**							
	2017/18	21,677	501 (2.3)	<10 [6]	195 (38.9)	23 (4.6)	218 (43.5)
	2018/19	22,099	478 (2.2)	<10 [6]	187 (39.1)	37 (7.7)	224 (46.9)
	2019/20	25,811	540 (2.1)	<10 [6]	187 (34.6)	54 (10.0)	241 (44.6)
**Prison**							
2017/18	Prison 1	1,323	<10 [6]	<10 [6]	<10 [6]	<10 [6]	<10 [6]
	Prison 2	3,261	64 (2.0)	<10 [6]	26 (40.6)	<10 [6]	27 (42.2)
	Prison 3	2,623	64 (2.4)	<10 [6]	<10 [6]	<10 [6]	<10 [6]
	Prison 4	2,089	82 (3.9)	<10 [6]	58 (70.7)	<10 [6]	64 (78.0)
	Prison 5	637	14 (2.2)	<10 [6]	<10 [6]	<10 [6]	<10 [6]
	Prison 6	1,552	28 (1.8)	<10 [6]	10 (35.7)	<10 [6]	14 (50.0)
	Prison 7	635	<10 [6]	<10 [6]	<10 [6]	<10 [6]	<10 [6]
	Prison 8	1,085	<10 [6]	<10 [6]	<10 [6]	<10 [6]	<10 [6]
	Prison 9	981	76 (7.7)	<10 [6]	45 (59.2)	<10 [6]	48 (63.2)
	Prison 10	2,523	54 (2.1)	<10 [6]	19 (35.2)	<10 [6]	19 (35.2)
	Prison 11	3,470	70 (2.0)	<10 [6]	<10 [6]	<10 [6]	13 (18.6)
	Prison 12	815	<10 [6]	<10 [6]	<10 [6]	<10 [6]	<10 [6]
	Prison 13	683	43 (6.3)	<10 [6]	27 (62.8)	<10 [6]	29 (67.4)
2018/19	Prison 1	1,333	<10 [6]	<10 [6]	<10 [6]	<10 [6]	<10 [6]
	Prison 2	2,705	54 (2.0)	<10 [6]	26 (48.1)	<10 [6]	27 (50.0)
	Prison 3	2,522	60 (2.4)	<10 [6]	<10 [6]	<10 [6]	<10 [6]
	Prison 4	2,349	76 (3.2)	<10 [6]	58 (76.3)	<10 [6]	65 (85.5)
	Prison 5	676	19 (2.8)	<10 [6]	<10 [6]	<10 [6]	<10 [6]
	Prison 6	1,513	27 (1.8)	<10 [6]	13 (48.1)	<10 [6]	18 (66.7)
	Prison 7	654	<10 [6]	<10 [6]	<10 [6]	<10 [6]	<10 [6]
	Prison 8	1,148	<10 [6]	<10 [6]	<10 [6]	<10 [6]	<10 [6]
	Prison 9	996	81 (8.1)	<10 [6]	42 (51.9)	<10 [6]	49 (60.5)
	Prison 10	2,717	54 (2.0)	<10 [6]	11 (20.4)	<10 [6]	14 (25.9)
	Prison 11	4,020	56 (1.4)	<10 [6]	<10 [6]	<10 [6]	11 (19.6)
	Prison 12	792	<10 [6]	<10 [6]	<10 [6]	<10 [6]	<10 [6]
	Prison 13	674	48 (7.1)	<10 [6]	27 (56.3)	<10 [6]	31 (64.6)
2019/20	Prison 1	1,410	<10 [6]	<10 [6]	<10 [6]	<10 [6]	<10 [6]
	Prison 2	2,979	60 (2.0)	<10 [6]	30 (50.0)	<10 [6]	36 (60.0)
	Prison 3	2,809	60 (2.1)	<10 [6]	<10 [6]	<10 [6]	<10 [6]
	Prison 4	2,651	91 (3.4)	<10 [6]	51 (56.0)	10 (11.0)	61 (67.0)
	Prison 5	616	13 (2.1)	<10 [6]	<10 [6]	<10 [6]	10 (76.9)
	Prison 6	1,533	22 (1.4)	<10 [6]	<10 [6]	<10 [6]	14 (63.6)
	Prison 7	860	<10 [6]	<10 [6]	<10 [6]	<10 [6]	<10 [6]
	Prison 8	1,385	<10 [6]	<10 [6]	<10 [6]	<10 [6]	<10 [6]
	Prison 9	1,092	95 (8.7)	<10 [6]	44 (46.3)	<10 [6]	55 (57.9)
	Prison 10	3,577	59 (1.6)	<10 [6]	14 (23.7)	<10 [6]	18 (30.5)
	Prison 11	5,348	78 (1.5)	<10 [6]	<10 [6]	<10 [6]	<10 [6]
	Prison 12	805	<10 [6]	<10 [6]	<10 [6]	<10 [6]	<10 [6]
	Prison 13	746	56 (7.5)	<10 [6]	30 (53.6)	<10 [6]	33 (58.9)
**Prison category**							
2017/18	A	1,664	119 (7.2)	<10 [6]	72 (60.5)	<10 [6]	77 (64.7)
	B	9,254	188 (2.0)	<10 [6]	53 (28.2)	<10 [6]	59 (31.4)
	C	6,035	151 (2.5)	<10 [6]	60 (39.7)	<10 [6]	67 (44.4)
	Closed	1,720	<10 [6]	<10 [6]	<10 [6]	<10 [6]	<10 [6]
	D	2,189	42 (1.9)	<10 [6]	10 (23.8)	<10 [6]	15 (35.7)
	YOI	815	<10 [6]	<10 [6]	<10 [6]	<10 [6]	<10 [6]
2018/19	A	1,670	129 (7.7)	<10 [6]	69 (53.5)	11 (8.5)	80 (62.0)
	B	9,442	164 (1.7)	<10 [6]	45 (27.4)	<10 [6]	52 (31.7)
	C	6,204	139 (2.2)	<10 [6]	59 (42.4)	<10 [6]	68 (48.9)
	Closed	1,802	<10 [6]	<10 [6]	<10 [6]	<10 [6]	<10 [6]
	D	2,189	46 (2.1)	<10 [6]	14 (30.4)	10 (21.7)	24 (52.2)
	YOI	792	<10 [6]	<10 [6]	<10 [6]	<10 [6]	<10 [6]
2019/20	A	1,838	151 (8.2)	<10 [6]	74 (49.0)	14 (9.3)	88 (58.3)
	B	11,904	197 (1.7)	<10 [6]	49 (24.9)	14 (7.1)	63 (32.0)
	C	6,870	157 (2.3)	<10 [6]	52 (33.1)	14 (8.9)	66 (42.0)
	Closed	2,245	<10 [6]	<10 [6]	<10 [6]	<10 [6]	<10 [6]
	D	2,149	35 (1.6)	<10 [6]	12 (34.3)	12 (34.3)	24 (68.6)
	YOI	805	<10 [6]	<10 [6]	<10 [6]	<10 [6]	<10 [6]
**Gender**							
2017/18	F	1,699	0 (0.0)	<10 [6]	0 (0.0)	0 (0.0)	0 (0.0)
	M	19,977	501 (2.5)	<10 [6]	195 (38.9)	23 (4.6)	218 (43.5)
2018/19	F	1,802	0 (0.0)	<10 [6]	0 (0.0)	0 (0.0)	0 (0.0)
	M	20,295	478 (2.4)	<10 [6]	187 (39.1)	37 (7.7)	224 (46.9)
2019/20	F	1,376	0 (0.0)	<10 [6]	0 (0.0)	0 (0.0)	0 (0.0)
	M	23,570	540 (2.3)	<10 [6]	187 (34.6)	54 (10.0)	241 (44.6)

[1]Eligible for indicator; [2]Achieve in current prison; [3]Achieve in previous prison; [4]Overall achievement - either current or previous prison; [5]Declined indicator; [6]Suppressed (<10) to avoid disclosure

Indicator	*The proportion of men eligible for screening and aged 65 years or over with a record of abdominal aortic aneurysm screening*						no comparable data in QOF 2019/20. PHE source states initial uptake of screening in 2014/15 was 79.5%
	**Group: Screening**						
	**Variable**	**Population**	**Eligible[1] (% popln)**	**Declined[5] (% eligible)**	**Satisfy[2] (% eligible)**	**Elsewhere[3] (% eligible)**	**Achieve[4] (% eligible)**
**Sentence Status**							
2017/18	.	81	<10 [6]	<10 [6]	<10 [6]	<10 [6]	<10 [6]
	Absconded	<10 [6]	<10 [6]	<10 [6]	<10 [6]	<10 [6]	<10 [6]
	Active In	15,872	380 (2.4)	<10 [6]	149 (39.2)	20 (5.3)	169 (44.5)
	Active Out	1,052	20 (1.9)	<10 [6]	<10 [6]	<10 [6]	<10 [6]
	Convicted Sentence	2,125	55 (2.6)	<10 [6]	23 (41.8)	<10 [6]	23 (41.8)
	Downgrade in security category	<10 [6]	<10 [6]	<10 [6]	<10 [6]	<10 [6]	<10 [6]
	Internal Cell Move	<10 [6]	<10 [6]	<10 [6]	<10 [6]	<10 [6]	<10 [6]
	Judges Remand	92	<10 [6]	<10 [6]	<10 [6]	<10 [6]	<10 [6]
	Licence Revoke	52	<10 [6]	<10 [6]	<10 [6]	<10 [6]	<10 [6]
	On Remand	1,492	17 (1.1)	<10 [6]	<10 [6]	<10 [6]	<10 [6]
	Transfer	910	21 (2.3)	<10 [6]	<10 [6]	<10 [6]	10 (47.6)
	Upgrade in security category	<10 [6]	<10 [6]	<10 [6]	<10 [6]	<10 [6]	<10 [6]
2018/19	.	88	<10 [6]	<10 [6]	<10 [6]	<10 [6]	<10 [6]
	Absconded	<10 [6]	<10 [6]	<10 [6]	<10 [6]	<10 [6]	<10 [6]
	Active In	18,145	403 (2.2)	<10 [6]	157 (39.0)	36 (8.9)	193 (47.9)
	Active Out	835	12 (1.4)	<10 [6]	<10 [6]	<10 [6]	<10 [6]
	Convicted Sentence	1,320	35 (2.7)	<10 [6]	15 (42.9)	<10 [6]	15 (42.9)
	Downgrade in security category	<10 [6]	<10 [6]	<10 [6]	<10 [6]	<10 [6]	<10 [6]
	Internal Cell Move	<10 [6]	<10 [6]	<10 [6]	<10 [6]	<10 [6]	<10 [6]
	Judges Remand	<10 [6]	<10 [6]	<10 [6]	<10 [6]	<10 [6]	<10 [6]
	Licence Revoke	125	<10 [6]	<10 [6]	<10 [6]	<10 [6]	<10 [6]
	On Remand	1,059	16 (1.5)	<10 [6]	<10 [6]	<10 [6]	<10 [6]
	Transfer	518	<10 [6]	<10 [6]	<10 [6]	<10 [6]	<10 [6]
	Upgrade in security category	<10 [6]	<10 [6]	<10 [6]	<10 [6]	<10 [6]	<10 [6]
2019/20	.	69	<10 [6]	<10 [6]	<10 [6]	<10 [6]	<10 [6]
	Absconded	<10 [6]	<10 [6]	<10 [6]	<10 [6]	<10 [6]	<10 [6]
	Active In	22,424	465 (2.1)	<10 [6]	156 (33.5)	49 (10.5)	205 (44.1)
	Active Out	625	14 (2.2)	<10 [6]	<10 [6]	<10 [6]	<10 [6]
	Convicted Sentence	1,361	35 (2.6)	<10 [6]	17 (48.6)	<10 [6]	18 (51.4)
	Downgrade in security category	<10 [6]	<10 [6]	<10 [6]	<10 [6]	<10 [6]	<10 [6]
	Internal Cell Move	<10 [6]	<10 [6]	<10 [6]	<10 [6]	<10 [6]	<10 [6]
	Judges Remand	19	<10 [6]	<10 [6]	<10 [6]	<10 [6]	<10 [6]
	Licence Revoke	178	<10 [6]	<10 [6]	<10 [6]	<10 [6]	<10 [6]
	On Remand	1,031	18 (1.7)	<10 [6]	<10 [6]	<10 [6]	11 (61.1)
	Transfer	101	<10 [6]	<10 [6]	<10 [6]	<10 [6]	<10 [6]
	Upgrade in security category	<10 [6]	<10 [6]	<10 [6]	<10 [6]	<10 [6]	<10 [6]
**Age - years**							
2017/18	10 - <20	468	<10 [6]	<10 [6]	<10 [6]	<10 [6]	<10 [6]
	20 - <30	6,994	<10 [6]	<10 [6]	<10 [6]	<10 [6]	<10 [6]
	30 - <40	7,051	<10 [6]	<10 [6]	<10 [6]	<10 [6]	<10 [6]
	40 - <50	4,114	<10 [6]	<10 [6]	<10 [6]	<10 [6]	<10 [6]
	50 - <60	2,107	<10 [6]	<10 [6]	<10 [6]	<10 [6]	<10 [6]
	60 - <70	213	211 (99.1)	<10 [6]	89 (42.2)	10 (4.7)	99 (46.9)
	70 - <80	40	40 (100.0)	<10 [6]	11 (27.5)	<10 [6]	11 (27.5)
	80 - <90	<10 [6]	<10 [6]	<10 [6]	<10 [6]	<10 [6]	<10 [6]
	90 - <100	<10 [6]	<10 [6]	<10 [6]	<10 [6]	<10 [6]	<10 [6]
	100 - <110	<10 [6]	<10 [6]	<10 [6]	<10 [6]	<10 [6]	<10 [6]
2018/19	10 - <20	436	<10 [6]	<10 [6]	<10 [6]	<10 [6]	<10 [6]
	20 - <30	7,163	<10 [6]	<10 [6]	<10 [6]	<10 [6]	<10 [6]
	30 - <40	7,381	<10 [6]	<10 [6]	<10 [6]	<10 [6]	<10 [6]
	40 - <50	4,180	<10 [6]	<10 [6]	<10 [6]	<10 [6]	<10 [6]
	50 - <60	1,978	<10 [6]	<10 [6]	<10 [6]	<10 [6]	<10 [6]
	60 - <70	701	227 (32.4)	<10 [6]	87 (38.3)	15 (6.6)	102 (44.9)
	70 - <80	209	204 (97.6)	<10 [6]	80 (39.2)	19 (9.3)	99 (48.5)
	80 - <90	45	44 (97.8)	<10 [6]	18 (40.9)	<10 [6]	21 (47.7)
	90 - <100	<10 [6]	<10 [6]	<10 [6]	<10 [6]	<10 [6]	<10 [6]
	100 - <110	<10 [6]	<10 [6]	<10 [6]	<10 [6]	<10 [6]	<10 [6]
2019/20	10 - <20	404	<10 [6]	<10 [6]	<10 [6]	<10 [6]	<10 [6]
	20 - <30	9,125	<10 [6]	<10 [6]	<10 [6]	<10 [6]	<10 [6]
	30 - <40	4,948	<10 [6]	<10 [6]	<10 [6]	<10 [6]	<10 [6]
	40 - <50	2,224	<10 [6]	<10 [6]	<10 [6]	<10 [6]	<10 [6]
	50 - <60	751	254 (33.8)	<10 [6]	91 (35.8)	20 (7.9)	111 (43.7)
	60 - <70	238	233 (97.9)	<10 [6]	74 (31.8)	32 (13.7)	106 (45.5)
	70 - <80	53	51 (96.2)	<10 [6]	20 (39.2)	<10 [6]	22 (43.1)
	80 - <90	<10 [6]	<10 [6]	<10 [6]	<10 [6]	<10 [6]	<10 [6]
	90 - <100	<10 [6]	<10 [6]	<10 [6]	<10 [6]	<10 [6]	<10 [6]
	100 - <110	<10 [6]	<10 [6]	<10 [6]	<10 [6]	<10 [6]	<10 [6]

[1]Eligible for indicator; [2]Achieve in current prison; [3]Achieve in previous prison; [4]Overall achievement - either current or previous prison; [5]Declined indicator; [6]Suppressed (<10) to avoid disclosure

Indicator		The proportion of men eligible for screening and aged 65 years or over with a record of abdominal aortic aneurysm screening					no comparable data in QOF 2019/20. PHE source states initial uptake of screening in 2014/15 was 79.5%
	**Group: Screening Variable**	**Population**	**Eligible[1] (% popln)**	**Declined[5] (% eligible)**	**Satisfy[2] (% eligible)**	**Elsewhere[3] (% eligible)**	**Achieve[4] (% eligible)**
**Length of Stay (months)**							
2017/18	<1	4,474	60 (1.3)	<10 [6]	<10 [6]	1 (1.7)	<10 [6]
	1-<6	8,075	92 (1.1)	<10 [6]	11 (12.0)	<10 [6]	15 (16.3)
	6-<12	3,672	67 (1.8)	<10 [6]	30 (44.8)	<10 [6]	33 (49.3)
	12-<24	2,832	80 (2.8)	<10 [6]	35 (43.8)	<10 [6]	39 (48.8)
	24+	2,624	202 (7.7)	<10 [6]	118 (58.4)	11 (5.4)	129 (63.9)
2018/19	<1	4,801	62 (1.3)	<10 [6]	<10 [6]	<10 [6]	<10 [6]
	1-<6	7,742	76 (1.0)	<10 [6]	12 (15.8)	<10 [6]	19 (25.0)
	6-<12	3,616	49 (1.4)	<10 [6]	18 (36.7)	<10 [6]	21 (42.9)
	12-<24	3,447	87 (2.5)	<10 [6]	39 (44.8)	<10 [6]	46 (52.9)
	24+	2,493	204 (8.2)	<10 [6]	117 (57.4)	15 (7.4)	132 (64.7)
2019/20	<1	5,745	78 (1.4)	<10 [6]	<10 [6]	<10 [6]	<10 [6]
	1-<6	9,697	112 (1.2)	<10 [6]	14 (12.5)	14 (12.5)	28 (25.0)
	6-<12	5,090	76 (1.5)	<10 [6]	32 (42.1)	10 (13.2)	42 (55.3)
	12-<24	3,244	83 (2.6)	<10 [6]	36 (43.4)	<10 [6]	45 (54.2)
	24+	2,035	191 (9.4)	<10 [6]	102 (53.4)	15 (7.9)	117 (61.3)
**Ethnic Group**							
2017/18	White	15,638	417 (2.7)	<10 [6]	177 (42.4)	22 (5.3)	199 (47.7)
	Mixed	431	<10 [6]	<10 [6]	<10 [6]	<10 [6]	<10 [6]
	Asian or Asian British	813	<10 [6]	<10 [6]	<10 [6]	<10 [6]	<10 [6]
	Black or Black British	404	<10 [6]	<10 [6]	<10 [6]	<10 [6]	<10 [6]
	Chinese and Other	214	<10 [6]	<10 [6]	<10 [6]	<10 [6]	<10 [6]
	Unclassified	372	13 (3.5)	<10 [6]	<10 [6]	<10 [6]	<10 [6]
2018/19	White	14,911	386 (2.6)	<10 [6]	166 (43.0)	30 (7.8)	196 (50.8)
	Mixed	371	<10 [6]	<10 [6]	<10 [6]	<10 [6]	<10 [6]
	Asian or Asian British	726	10 (1.4)	<10 [6]	<10 [6]	<10 [6]	<10 [6]
	Black or Black British	364	<10 [6]	<10 [6]	<10 [6]	<10 [6]	<10 [6]
	Chinese and Other	167	<10 [6]	<10 [6]	<10 [6]	<10 [6]	<10 [6]
	Unclassified	409	<10 [6]	<10 [6]	<10 [6]	<10 [6]	<10 [6]
2019/20	White	16,606	363 (2.2)	<10 [6]	160 (44.1)	39 (10.7)	199 (54.8)
	Mixed	409	<10 [6]	<10 [6]	<10 [6]	<10 [6]	<10 [6]
	Asian or Asian British	755	12 (1.6)	<10 [6]	<10 [6]	<10 [6]	<10 [6]
	Black or Black British	451	<10 [6]	<10 [6]	<10 [6]	<10 [6]	<10 [6]
	Chinese and Other	163	<10 [6]	<10 [6]	<10 [6]	<10 [6]	<10 [6]
	Unclassified	387	12 (3.1)	<10 [6]	<10 [6]	<10 [6]	<10 [6]

[1]Eligible for indicator; [2]Achieve in current prison; [3]Achieve in previous prison; [4]Overall achievement - either current or previous prison; [5]Declined indicator; [6]Suppressed (<10) to avoid disclosure

Indicator	*The proportion of people aged 45 to 74 years with a recorded cardiovascular disease risk assessment in the preceding 5 years, and who do NOT have a diagnosis of: coronary heart disease, chronic kidney disease, diabetes, hypertension, atrial fibrillation, transient ischaemic attack, familial hypercholesterolemia, heart failure, peripheral arterial disease, stroke or previously documented 20% or higher 10-year cardiovascular disease risk BEFORE the last 5 years*	no comparable data in QOF. PHE Fingertips states: % of people in England invited for NHS Health Check per year in 2019/20 was 17.7%, % of people in England receiving an NHS Health Check per year in 2019/20 was 7.7%, % of people in England taking up an NHS Health Check per year in 2019/20 was 43.7%

Group: Screening

	Variable	Population	Eligible[1] (% popln)	Declined[5] (% eligible)	Satisfy[2] (% eligible)	Elsewhere[3] (% eligible)	Achieve[4] (% eligible)
**Year**							
	2019/20	8,225	1,183 (14.4)	77 (6.5)	198 (16.7)	155 (13.1)	353 (29.8)
**Prison**							
2019/20	Prison 1	488	46 (9.4)	<10 [6]	<10 [6]	15 (32.6)	24 (52.2)
	Prison 2	756	114 (15.1)	<10 [6]	<10 [6]	22 (19.3)	30 (26.3)
	Prison 3	1,144	136 (11.9)	<10 [6]	20 (14.7)	21 (15.4)	41 (30.1)
	Prison 4	1,305	196 (15.0)	<10 [6]	<10 [6]	27 (13.8)	32 (16.3)
	Prison 5	205	38 (18.5)	<10 [6]	<10 [6]	<10 [6]	10 (26.3)
	Prison 6	462	72 (15.6)	<10 [6]	37 (51.4)	11 (15.3)	48 (66.7)
	Prison 7	265	41 (15.5)	<10 [6]	<10 [6]	<10 [6]	10 (24.4)
	Prison 8	339	65 (19.2)	<10 [6]	10 (15.4)	<10 [6]	14 (21.5)
	Prison 9	827	169 (20.4)	27 (16.0)	35 (20.7)	16 (9.5)	51 (30.2)
	Prison 10	657	88 (13.4)	<10 [6]	14 (15.9)	10 (11.4)	24 (27.3)
	Prison 11	892	92 (10.3)	<10 [6]	<10 [6]	<10 [6]	<10 [6]
	Prison 12	310	<10 [6]	<10 [6]	<10 [6]	<10 [6]	<10 [6]
	Prison 13	575	126 (21.9)	19 (15.1)	47 (37.3)	14 (11.1)	61 (48.4)
2019/20	A	1,402	295 (21.0)	46 (15.6)	82 (27.8)	30 (10.2)	112 (38.0)
	B	2,305	294 (12.8)	<10 [6]	24 (8.2)	38 (12.9)	62 (21.1)
	C	2,937	378 (12.9)	<10 [6]	34 (9.0)	63 (16.7)	97 (25.7)
	Closed	604	106 (17.5)	<10 [6]	18 (17.0)	<10 [6]	24 (22.6)
	D	667	110 (16.5)	17 (15.5)	40 (36.4)	18 (16.4)	58 (52.7)
	YOI	310	<10 [6]	<10 [6]	<10 [6]	<10 [6]	<10 [6]
**Gender**							
2019/20	F	604	108 (17.9)	<10 [6]	18 (16.7)	<10 [6]	25 (23.1)
	M	7,621	1,075 (14.1)	76 (7.1)	180 (16.7)	148 (13.8)	328 (30.5)
**Sentence Status**							
2019/20	.	<10 [6]	<10 [6]	<10 [6]	<10 [6]	<10 [6]	<10 [6]
	Active In	7,522	1,072 (14.3)	73 (6.8)	191 (17.8)	129 (12.0)	320 (29.9)
	Active Out	72	<10 [6]	<10 [6]	<10 [6]	<10 [6]	<10 [6]
	Convicted Sentence	277	43 (15.5)	<10 [6]	<10 [6]	10 (23.3)	12 (27.9)
	Judges Remand	<10 [6]	<10 [6]	<10 [6]	<10 [6]	<10 [6]	<10 [6]
	Licence Revoke	33	10 (30.3)	<10 [6]	<10 [6]	<10 [6]	<10 [6]
	On Remand	300	50 (16.7)	<10 [6]	<10 [6]	<10 [6]	10 (20.0)
	Transfer	16	<10 [6]	<10 [6]	<10 [6]	<10 [6]	<10 [6]
**Age - years**							
2019/20	10 - <20	150	<10 [6]	<10 [6]	<10 [6]	<10 [6]	<10 [6]
	20 - <30	2,307	<10 [6]	<10 [6]	<10 [6]	<10 [6]	<10 [6]
	30 - <40	2,802	<10 [6]	<10 [6]	<10 [6]	<10 [6]	<10 [6]
	40 - <50	1,682	522 (31.0)	24 (4.6)	72 (13.8)	78 (14.9)	150 (28.7)
	50 - <60	812	528 (65.0)	34 (6.4)	104 (19.7)	67 (12.7)	171 (32.4)
	60 - <70	339	118 (34.8)	17 (14.4)	21 (17.8)	<10 [6]	30 (25.4)
	70 - <80	113	15 (13.3)	<10 [6]	<10 [6]	<10 [6]	<10 [6]
	80 - <90	20	0 (0.0)	<10 [6]	<10 [6]	<10 [6]	<10 [6]
**Length of Stay (months)**							
	1-<6	2,686	293 (10.9)	<10 [6]	19 (6.5)	40 (13.7)	59 (20.1)
	6-<12	1,697	208 (12.3)	14 (6.7)	34 (16.3)	43 (20.7)	77 (37.0)
	12-<24	1,422	222 (15.6)	15 (6.8)	37 (16.7)	43 (19.4)	80 (36.0)
	24+	1,350	323 (23.9)	48 (14.9)	103 (31.9)	13 (4.0)	116 (35.9)
**Ethnic Group**							
	Mixed	188	34 (18.1)	<10 [6]	<10 [6]	<10 [6]	11 (32.4)
	Asian or Asian British	310	37 (11.9)	<10 [6]	<10 [6]	<10 [6]	14 (37.8)
	Black or Black British	211	22 (10.4)	<10 [6]	<10 [6]	<10 [6]	11 (50.0)
	Chinese and Other	85	11 (12.9)	<10 [6]	<10 [6]	<10 [6]	<10 [6]
	Unclassified	128	30 (23.4)	<10 [6]	10 (33.3)	<10 [6]	12 (40.0)

[1]Eligible for indicator; [2]Achieve in current prison; [3]Achieve in previous prison; [4]Overall achievement - either current or previous prison; [5]Declined indicator; [6]Suppressed (<10) to avoid disclosure

Indicator	*The proportion of people who have received three or more prescriptions for an antipsychotic in the preceding 12 months, and who have a record of blood testing (fasting glucose, HbA1c, lipid profile, urea and electrolytes, full blood count, liver function tests), body mass index and blood pressure*			***Community achievement 2019/20: No direct comparison in QOF - just percentage of patients with MH diagnosis of schizophrenia, bipolar affective disorder and other psychoses who have record of BP and BMI in the last 12 months (80.28% and 74.40% respectively)***							
	**Group: Mental health**										
			**Eligible[1]**	**Fasting glucose (% eligible)**		**HbA1c (% eligible)**		**Lipid profile (% eligible)**		**(% eligible)**	
	**Variable**	**Population**	**(% popln)**	**Satisfy[2]**	**Elsewhere[3]**	**Satisfy[2]**	**Elsewhere[3]**	**Satisfy[2]**	**Elsewhere[3]**	**Satisfy[2]**	**Elsewhere[3]**
**Year**											
	2017/18	21,677	244 (1.1)	<10 [6]	<10 [6]	75 (30.7)	<10 [6]	57 (23.4)	<10 [6]	112 (45.9)	12 (4.9)
	2018/19	22,099	326 (1.5)	<10 [6]	<10 [6]	31 (9.5)	11 (3.4)	28 (8.6)	13 (4.0)	46 (14.1)	21 (6.4)
	2019/20	25,811	463 (1.8)	<10 [6]	<10 [6]	29 (6.3)	<10 [6]	22 (4.8)	<10 [6]	35 (7.6)	<10 [6]
**Prison**											
2017/18	Prison 1	1,323	11 (0.8)	<10 [6]	<10 [6]	<10 [6]	<10 [6]	<10 [6]	<10 [6]	<10 [6]	<10 [6]
	Prison 2	3,261	36 (1.1)	<10 [6]	<10 [6]	11 (30.6)	<10 [6]	<10 [6]	<10 [6]	15 (41.7)	<10 [6]
	Prison 3	2,623	38 (1.4)	<10 [6]	<10 [6]	<10 [6]	<10 [6]	<10 [6]	<10 [6]	12 (31.6)	<10 [6]
	Prison 4	2,089	22 (1.1)	<10 [6]	<10 [6]	<10 [6]	<10 [6]	<10 [6]	<10 [6]	<10 [6]	<10 [6]
	Prison 5	637	<10 [6]	<10 [6]	<10 [6]	<10 [6]	<10 [6]	<10 [6]	<10 [6]	<10 [6]	<10 [6]
	Prison 6	1,552	<10 [6]	<10 [6]	<10 [6]	<10 [6]	<10 [6]	<10 [6]	<10 [6]	<10 [6]	<10 [6]
	Prison 7	635	15 (2.4)	<10 [6]	<10 [6]	<10 [6]	<10 [6]	<10 [6]	<10 [6]	11 (73.3)	<10 [6]
	Prison 8	1,085	27 (2.5)	<10 [6]	<10 [6]	12 (44.4)	<10 [6]	<10 [6]	<10 [6]	16 (59.3)	<10 [6]
	Prison 9	981	28 (2.9)	<10 [6]	<10 [6]	17 (60.7)	<10 [6]	16 (57.1)	<10 [6]	20 (71.4)	<10 [6]
	Prison 10	2,523	19 (0.8)	<10 [6]	<10 [6]	<10 [6]	<10 [6]	<10 [6]	<10 [6]	<10 [6]	<10 [6]
	Prison 11	3,470	23 (0.7)	<10 [6]	<10 [6]	<10 [6]	<10 [6]	<10 [6]	<10 [6]	<10 [6]	<10 [6]
	Prison 12	815	<10 [6]	<10 [6]	<10 [6]	<10 [6]	<10 [6]	<10 [6]	<10 [6]	<10 [6]	<10 [6]
	Prison 13	683	15 (2.2)	<10 [6]	<10 [6]	11 (73.3)	<10 [6]	<10 [6]	<10 [6]	11 (73.3)	<10 [6]
2018/19	Prison 1	1,333	16 (1.2)	<10 [6]	<10 [6]	<10 [6]	<10 [6]	<10 [6]	<10 [6]	<10 [6]	<10 [6]
	Prison 2	2,705	43 (1.6)	<10 [6]	<10 [6]	<10 [6]	<10 [6]	<10 [6]	<10 [6]	<10 [6]	<10 [6]
	Prison 3	2,522	34 (1.3)	<10 [6]	<10 [6]	<10 [6]	<10 [6]	<10 [6]	<10 [6]	<10 [6]	<10 [6]
	Prison 4	2,349	37 (1.6)	<10 [6]	<10 [6]	<10 [6]	<10 [6]	<10 [6]	<10 [6]	<10 [6]	<10 [6]
	Prison 5	676	<10 [6]	<10 [6]	<10 [6]	<10 [6]	<10 [6]	<10 [6]	<10 [6]	<10 [6]	<10 [6]
	Prison 6	1,513	<10 [6]	<10 [6]	<10 [6]	<10 [6]	<10 [6]	<10 [6]	<10 [6]	<10 [6]	<10 [6]
	Prison 7	654	45 (6.9)	<10 [6]	<10 [6]	<10 [6]	<10 [6]	<10 [6]	<10 [6]	<10 [6]	<10 [6]
	Prison 8	1,148	30 (2.6)	<10 [6]	<10 [6]	<10 [6]	<10 [6]	<10 [6]	<10 [6]	<10 [6]	<10 [6]
	Prison 9	996	28 (2.8)	<10 [6]	<10 [6]	<10 [6]	<10 [6]	<10 [6]	<10 [6]	<10 [6]	<10 [6]
	Prison 10	2,717	<10 [6]	<10 [6]	<10 [6]	<10 [6]	<10 [6]	<10 [6]	<10 [6]	<10 [6]	<10 [6]
	Prison 11	4,020	73 (1.8)	<10 [6]	<10 [6]	<10 [6]	<10 [6]	<10 [6]	<10 [6]	<10 [6]	<10 [6]
	Prison 12	792	<10 [6]	<10 [6]	<10 [6]	<10 [6]	<10 [6]	<10 [6]	<10 [6]	<10 [6]	<10 [6]
	Prison 13	674	17 (2.5)	<10 [6]	<10 [6]	<10 [6]	<10 [6]	<10 [6]	<10 [6]	<10 [6]	<10 [6]
2019/20	Prison 1	1,410	16 (1.1)	<10 [6]	<10 [6]	<10 [6]	<10 [6]	<10 [6]	<10 [6]	<10 [6]	<10 [6]
	Prison 2	2,979	43 (1.4)	<10 [6]	<10 [6]	<10 [6]	<10 [6]	<10 [6]	<10 [6]	<10 [6]	<10 [6]
	Prison 3	2,809	55 (2.0)	<10 [6]	<10 [6]	<10 [6]	<10 [6]	<10 [6]	<10 [6]	<10 [6]	<10 [6]
	Prison 4	2,651	35 (1.3)	<10 [6]	<10 [6]	<10 [6]	<10 [6]	<10 [6]	<10 [6]	<10 [6]	<10 [6]
	Prison 5	616	<10 [6]	<10 [6]	<10 [6]	<10 [6]	<10 [6]	<10 [6]	<10 [6]	<10 [6]	<10 [6]
	Prison 6	1,533	<10 [6]	<10 [6]	<10 [6]	<10 [6]	<10 [6]	<10 [6]	<10 [6]	<10 [6]	<10 [6]
	Prison 7	860	75 (8.7)	<10 [6]	<10 [6]	<10 [6]	<10 [6]	<10 [6]	<10 [6]	<10 [6]	<10 [6]
	Prison 8	1,385	38 (2.7)	<10 [6]	<10 [6]	<10 [6]	<10 [6]	<10 [6]	<10 [6]	<10 [6]	<10 [6]
	Prison 9	1,092	31 (2.8)	<10 [6]	<10 [6]	12 (38.7)	<10 [6]	12 (38.7)	<10 [6]	10 (32.3)	<10 [6]
	Prison 10	3,577	38 (1.1)	<10 [6]	<10 [6]	<10 [6]	<10 [6]	<10 [6]	<10 [6]	<10 [6]	<10 [6]
	Prison 11	5,348	97 (1.8)	<10 [6]	<10 [6]	<10 [6]	<10 [6]	<10 [6]	<10 [6]	<10 [6]	<10 [6]
	Prison 12	805	<10 [6]	<10 [6]	<10 [6]	<10 [6]	<10 [6]	<10 [6]	<10 [6]	<10 [6]	<10 [6]
	Prison 13	746	21 (2.8)	<10 [6]	<10 [6]	<10 [6]	<10 [6]	<10 [6]	<10 [6]	<10 [6]	<10 [6]
**Prison category**											
2017/18	A	1,664	43 (2.6)	<10 [6]	<10 [6]	28 (65.1)	<10 [6]	25 (58.1)	<10 [6]	31 (72.1)	<10 [6]
	B	9,254	78 (0.8)	<10 [6]	<10 [6]	14 (17.9)	<10 [6]	<10 [6]	<10 [6]	28 (35.9)	<10 [6]
	C	6,035	71 (1.2)	<10 [6]	<10 [6]	12 (16.9)	<10 [6]	13 (18.3)	<10 [6]	23 (32.4)	<10 [6]
	Closed	1,720	42 (2.4)	<10 [6]	<10 [6]	19 (45.2)	<10 [6]	12 (28.6)	<10 [6]	27 (64.3)	<10 [6]
	D	2,189	<10 [6]	<10 [6]	<10 [6]	<10 [6]	<10 [6]	<10 [6]	<10 [6]	<10 [6]	<10 [6]
	YOI	815	<10 [6]	<10 [6]	<10 [6]	<10 [6]	<10 [6]	<10 [6]	<10 [6]	<10 [6]	<10 [6]
2018/19	A	1,670	45 (2.7)	<10 [6]	<10 [6]	15 (33.3)	<10 [6]	14 (31.1)	<10 [6]	14 (31.1)	<10 [6]
	B	9,442	116 (1.2)	<10 [6]	<10 [6]	<10 [6]	<10 [6]	<10 [6]	<10 [6]	<10 [6]	<10 [6]
	C	6,204	87 (1.4)	<10 [6]	<10 [6]	<10 [6]	<10 [6]	<10 [6]	<10 [6]	15 (17.2)	<10 [6]
	Closed	1,802	75 (4.2)	<10 [6]	<10 [6]	<10 [6]	<10 [6]	<10 [6]	<10 [6]	12 (16.0)	<10 [6]
	D	2,189	<10 [6]	<10 [6]	<10 [6]	<10 [6]	<10 [6]	<10 [6]	<10 [6]	<10 [6]	<10 [6]
	YOI	792	<10 [6]	<10 [6]	<10 [6]	<10 [6]	<10 [6]	<10 [6]	<10 [6]	<10 [6]	<10 [6]
2019/20	A	1,838	52 (2.8)	<10 [6]	<10 [6]	14 (26.9)	<10 [6]	14 (26.9)	<10 [6]	13 (25.0)	<10 [6]
	B	11,904	178 (1.5)	<10 [6]	<10 [6]	<10 [6]	<10 [6]	<10 [6]	<10 [6]	<10 [6]	<10 [6]
	C	6,870	106 (1.5)	<10 [6]	<10 [6]	<10 [6]	<10 [6]	<10 [6]	<10 [6]	<10 [6]	<10 [6]
	Closed	2,245	113 (5.0)	<10 [6]	<10 [6]	<10 [6]	<10 [6]	<10 [6]	<10 [6]	<10 [6]	<10 [6]
	D	2,149	11 (0.5)	<10 [6]	<10 [6]	<10 [6]	<10 [6]	<10 [6]	<10 [6]	<10 [6]	<10 [6]
	YOI	805	<10 [6]	<10 [6]	<10 [6]	<10 [6]	<10 [6]	<10 [6]	<10 [6]	<10 [6]	<10 [6]

[1]Eligible for indicator; [2]Achieve in current prison; [3]Achieve in previous prison; [4]Overall achievement - either current or previous prison; [5]Declined indicator; [6]Suppressed (<10) to avoid disclosure

Indicator	*The proportion of people who have received three or more prescriptions for an antipsychotic in the preceding 12 months, and who have a record of blood testing (fasting glucose, HbA1c, lipid profile, urea and electrolytes, full blood count, liver function tests), body mass index and blood pressure*										
	**Group: Mental health**										
			**Full blood count (% eligible)**		**Liver function (% eligible)**		**BMI (% eligible)**			**Blood Pressure (% eligible)**	
	**Variable**	**Eligible**	**Satisfy[2]**	**Elsewhere[3]**	**Satisfy[2]**	**Elsewhere[3]**	**Satisfy[2]**	**Elsewhere[3]**	**Declined[5]**	**Satisfy[2]**	**Elsewhere[3]**
Year											
	2017/18	244	116 (47.5)	11 (4.5)	107 (43.9)	<10 [6]	179 (73.4)	<10 [6]	<10 [6]	203 (83.2)	<10 [6]
	2018/19	326	49 (15.0)	22 (6.7)	40 (12.3)	14 (4.3)	95 (29.1)	18 (5.5)	<10 [6]	101 (31.0)	18 (5.5)
	2019/20	463	37 (8.0)	<10 [6]	17 (3.7)	<10 [6]	20 (4.3)	11 (2.4)	<10 [6]	62 (13.4)	<10 [6]
**Prison**											
2017/18	Prison 1	11	<10 [6]	<10 [6]	<10 [6]	<10 [6]	10 (90.9)	<10 [6]	<10 [6]	11 (100.0)	<10 [6]
	Prison 2	36	14 (38.9)	<10 [6]	12 (33.3)	<10 [6]	27 (75.0)	<10 [6]	<10 [6]	31 (86.1)	<10 [6]
	Prison 3	38	14 (36.8)	<10 [6]	10 (26.3)	<10 [6]	30 (78.9)	<10 [6]	<10 [6]	34 (89.5)	<10 [6]
	Prison 4	22	<10 [6]	<10 [6]	<10 [6]	<10 [6]	17 (77.3)	<10 [6]	<10 [6]	10 (45.5)	<10 [6]
	Prison 5	<10 [6]	<10 [6]	<10 [6]	<10 [6]	<10 [6]	<10 [6]	<10 [6]	<10 [6]	<10 [6]	<10 [6]
	Prison 6	<10 [6]	<10 [6]	<10 [6]	<10 [6]	<10 [6]	<10 [6]	<10 [6]	<10 [6]	<10 [6]	<10 [6]
	Prison 7	15	11 (73.3)	<10 [6]	11 (73.3)	<10 [6]	10 (66.7)	<10 [6]	<10 [6]	15 (100.0)	<10 [6]
	Prison 8	27	15 (55.6)	<10 [6]	14 (51.9)	<10 [6]	24 (88.9)	<10 [6]	<10 [6]	25 (92.6)	<10 [6]
	Prison 9	28	22 (78.6)	<10 [6]	20 (71.4)	<10 [6]	15 (53.6)	<10 [6]	<10 [6]	24 (85.7)	<10 [6]
	Prison 10	19	<10 [6]	<10 [6]	<10 [6]	<10 [6]	12 (63.2)	<10 [6]	<10 [6]	15 (78.9)	<10 [6]
	Prison 11	23	<10 [6]	<10 [6]	<10 [6]	<10 [6]	16 (69.6)	<10 [6]	<10 [6]	18 (78.3)	<10 [6]
	Prison 12	<10 [6]	<10 [6]	<10 [6]	<10 [6]	<10 [6]	<10 [6]	<10 [6]	<10 [6]	<10 [6]	<10 [6]
	Prison 13	15	11 (73.3)	<10 [6]	11 (73.3)	<10 [6]	12 (80.0)	<10 [6]	<10 [6]	13 (86.7)	<10 [6]
2018/19	Prison 1	16	<10 [6]	<10 [6]	<10 [6]	<10 [6]	<10 [6]	<10 [6]	<10 [6]	<10 [6]	<10 [6]
	Prison 2	43	<10 [6]	<10 [6]	<10 [6]	<10 [6]	<10 [6]	<10 [6]	<10 [6]	<10 [6]	<10 [6]
	Prison 3	34	10 (29.4)	<10 [6]	<10 [6]	<10 [6]	28 (82.4)	<10 [6]	<10 [6]	29 (85.3)	<10 [6]
	Prison 4	37	<10 [6]	<10 [6]	<10 [6]	<10 [6]	10 (27.0)	<10 [6]	<10 [6]	11 (29.7)	<10 [6]
	Prison 5	<10 [6]	<10 [6]	<10 [6]	<10 [6]	<10 [6]	<10 [6]	<10 [6]	<10 [6]	<10 [6]	<10 [6]
	Prison 6	<10 [6]	<10 [6]	<10 [6]	<10 [6]	<10 [6]	<10 [6]	<10 [6]	<10 [6]	<10 [6]	<10 [6]
	Prison 7	45	<10 [6]	<10 [6]	<10 [6]	<10 [6]	11 (24.4)	<10 [6]	<10 [6]	12 (26.7)	<10 [6]
	Prison 8	30	<10 [6]	<10 [6]	<10 [6]	<10 [6]	<10 [6]	<10 [6]	<10 [6]	<10 [6]	<10 [6]
	Prison 9	28	<10 [6]	<10 [6]	<10 [6]	<10 [6]	10 (35.7)	<10 [6]	<10 [6]	12 (42.9)	<10 [6]
	Prison 10	<10 [6]	<10 [6]	<10 [6]	<10 [6]	<10 [6]	<10 [6]	<10 [6]	<10 [6]	<10 [6]	<10 [6]
	Prison 11	73	<10 [6]	<10 [6]	<10 [6]	<10 [6]	<10 [6]	<10 [6]	<10 [6]	<10 [6]	<10 [6]
	Prison 12	<10 [6]	<10 [6]	<10 [6]	<10 [6]	<10 [6]	<10 [6]	<10 [6]	<10 [6]	<10 [6]	<10 [6]
	Prison 13	17	<10 [6]	<10 [6]	<10 [6]	<10 [6]	<10 [6]	<10 [6]	<10 [6]	<10 [6]	<10 [6]
2019/20	Prison 1	16	<10 [6]	<10 [6]	<10 [6]	<10 [6]	<10 [6]	<10 [6]	<10 [6]	<10 [6]	<10 [6]
	Prison 2	43	<10 [6]	<10 [6]	<10 [6]	<10 [6]	<10 [6]	<10 [6]	<10 [6]	<10 [6]	<10 [6]
	Prison 3	55	<10 [6]	<10 [6]	<10 [6]	<10 [6]	<10 [6]	<10 [6]	<10 [6]	<10 [6]	<10 [6]
	Prison 4	35	<10 [6]	<10 [6]	<10 [6]	<10 [6]	<10 [6]	<10 [6]	<10 [6]	<10 [6]	<10 [6]
	Prison 5	<10 [6]	<10 [6]	<10 [6]	<10 [6]	<10 [6]	<10 [6]	<10 [6]	<10 [6]	<10 [6]	<10 [6]
	Prison 6	<10 [6]	<10 [6]	<10 [6]	<10 [6]	<10 [6]	<10 [6]	<10 [6]	<10 [6]	<10 [6]	<10 [6]
	Prison 7	75	<10 [6]	<10 [6]	<10 [6]	<10 [6]	<10 [6]	<10 [6]	<10 [6]	<10 [6]	<10 [6]
	Prison 8	38	<10 [6]	<10 [6]	<10 [6]	<10 [6]	<10 [6]	<10 [6]	<10 [6]	<10 [6]	<10 [6]
	Prison 9	31	12 (38.7)	<10 [6]	11 (35.5)	<10 [6]	13 (41.9)	<10 [6]	<10 [6]	14 (45.2)	<10 [6]
	Prison 10	38	<10 [6]	<10 [6]	<10 [6]	<10 [6]	<10 [6]	<10 [6]	<10 [6]	<10 [6]	<10 [6]
	Prison 11	97	<10 [6]	<10 [6]	<10 [6]	<10 [6]	<10 [6]	<10 [6]	<10 [6]	<10 [6]	<10 [6]
	Prison 12	<10 [6]	<10 [6]	<10 [6]	<10 [6]	<10 [6]	<10 [6]	<10 [6]	<10 [6]	<10 [6]	<10 [6]
	Prison 13	21	<10 [6]	<10 [6]	<10 [6]	<10 [6]	<10 [6]	<10 [6]	<10 [6]	<10 [6]	<10 [6]
Prison category											
2017/18	A	43	33 (76.7)	<10 [6]	31 (72.1)	<10 [6]	27 (62.8)	<10 [6]	<10 [6]	37 (86.0)	<10 [6]
	B	78	27 (34.6)	<10 [6]	25 (32.1)	<10 [6]	55 (70.5)	<10 [6]	<10 [6]	64 (82.1)	<10 [6]
	C	71	27 (38.0)	<10 [6]	23 (32.4)	<10 [6]	57 (80.3)	<10 [6]	<10 [6]	55 (77.5)	<10 [6]
	Closed	42	26 (61.9)	<10 [6]	25 (59.5)	<10 [6]	34 (81.0)	<10 [6]	<10 [6]	40 (95.2)	<10 [6]
	D	<10 [6]	<10 [6]	<10 [6]	<10 [6]	<10 [6]	<10 [6]	<10 [6]	<10 [6]	<10 [6]	<10 [6]
	YOI	<10 [6]	<10 [6]	<10 [6]	<10 [6]	<10 [6]	<10 [6]	<10 [6]	<10 [6]	<10 [6]	<10 [6]
2018/19	A	45	15 (33.3)	<10 [6]	16 (35.6)	<10 [6]	18 (40.0)	<10 [6]	<10 [6]	19 (42.2)	<10 [6]
	B	116	<10 [6]	<10 [6]	<10 [6]	<10 [6]	16 (13.8)	<10 [6]	<10 [6]	17 (14.7)	<10 [6]
	C	87	17 (19.5)	<10 [6]	<10 [6]	<10 [6]	42 (48.3)	<10 [6]	<10 [6]	45 (51.7)	<10 [6]
	Closed	75	12 (16.0)	<10 [6]	12 (16.0)	<10 [6]	19 (25.3)	<10 [6]	<10 [6]	20 (26.7)	<10 [6]
	D	<10 [6]	<10 [6]	<10 [6]	<10 [6]	<10 [6]	<10 [6]	<10 [6]	<10 [6]	<10 [6]	<10 [6]
	YOI	<10 [6]	<10 [6]	<10 [6]	<10 [6]	<10 [6]	<10 [6]	<10 [6]	<10 [6]	<10 [6]	<10 [6]
2019/20	A	52	15 (28.8)	<10 [6]	12 (23.1)	<10 [6]	14 (26.9)	<10 [6]	<10 [6]	18 (34.6)	<10 [6]
	B	178	<10 [6]	<10 [6]	<10 [6]	<10 [6]	<10 [6]	<10 [6]	<10 [6]	21 (11.8)	<10 [6]
	C	106	<10 [6]	<10 [6]	<10 [6]	<10 [6]	<10 [6]	<10 [6]	<10 [6]	<10 [6]	<10 [6]
	Closed	113	<10 [6]	<10 [6]	<10 [6]	<10 [6]	<10 [6]	<10 [6]	<10 [6]	13 (11.5)	<10 [6]
	D	11	<10 [6]	<10 [6]	<10 [6]	<10 [6]	<10 [6]	<10 [6]	<10 [6]	<10 [6]	<10 [6]
	YOI	<10 [6]	<10 [6]	<10 [6]	<10 [6]	<10 [6]	<10 [6]	<10 [6]	<10 [6]	<10 [6]	<10 [6]

Community achievement 2019/20: No direct comparison in QOF - just percentage of patients with MH diagnosis of schizophrenia, bipolar affective disorder and other psychoses who have record of BP and BMI in the last 12 months (80.28% and 74.40% respectively)

[1]Eligible for indicator; [2]Achieve in current prison; [3]Achieve in previous prison; [4]Overall achievement - either current or previous prison; [5]Declined indicator; [6]Suppressed (<10) to avoid disclosure

Indicator	*The proportion of people who have received three or more prescriptions for an antipsychotic in the preceding 12 months, and who have a record of blood testing (fasting glucose, HbA1c, lipid profile, urea and electrolytes, full blood count, liver function tests), body mass index and blood pressure*			***Community achievement 2019/20: No direct comparison in QOF - just percentage of patients with MH diagnosis of schizophrenia, bipolar affective disorder and other psychoses who have record of BP and BMI in the last 12 months (80.28% and 74.40% respectively)***							
	**Group: Mental health**										
				**Fasting glucose (% eligible)**		**HbA1c (% eligible)**		**Lipid profile (% eligible)**		**Urea and electrolytes (% eligible)**	
	**Variable**	**Population**	**Eligible (% popln)**	**Satisfy[2]**	**Elsewhere[3]**	**Satisfy[2]**	**Elsewhere[3]**	**Satisfy[2]**	**Elsewhere[3]**	**Satisfy[2]**	**Elsewhere[3]**
**Gender**											
2017/18	F	1,699	42 (2.5)	<10 [6]	<10 [6]	19 (45.2)	<10 [6]	12 (28.6)	<10 [6]	27 (64.3)	<10 [6]
	M	19,977	202 (1.0)	<10 [6]	<10 [6]	56 (27.7)	<10 [6]	45 (22.3)	<10 [6]	85 (42.1)	11 (5.4)
2018/19	F	1,802	75 (4.2)	<10 [6]	<10 [6]	<10 [6]	<10 [6]	<10 [6]	<10 [6]	12 (16.0)	<10 [6]
	M	20,295	251 (1.2)	<10 [6]	<10 [6]	25 (10.0)	10 (4.0)	24 (9.6)	11 (4.4)	34 (13.5)	20 (8.0)
2019/20	F	1,376	38 (2.8)	<10 [6]	<10 [6]	<10 [6]	<10 [6]	<10 [6]	<10 [6]	<10 [6]	<10 [6]
	M	23,570	350 (1.5)	<10 [6]	<10 [6]	25 (7.1)	<10 [6]	18 (5.1)	<10 [6]	28 (8.0)	<10 [6]
**Sentence Status**											
2017/18	.	81	<10 [6]	<10 [6]	<10 [6]	<10 [6]	<10 [6]	<10 [6]	<10 [6]	<10 [6]	<10 [6]
	Absconded	<10 [6]	<10 [6]	<10 [6]	<10 [6]	<10 [6]	<10 [6]	<10 [6]	<10 [6]	<10 [6]	<10 [6]
	Active In	15,872	187 (1.2)	<10 [6]	<10 [6]	62 (33.2)	<10 [6]	50 (26.7)	<10 [6]	90 (48.1)	12 (6.4)
	Active Out	1,052	13 (1.2)	<10 [6]	<10 [6]	<10 [6]	<10 [6]	<10 [6]	<10 [6]	<10 [6]	<10 [6]
	Convicted Sentence	2,125	22 (1.0)	<10 [6]	<10 [6]	<10 [6]	<10 [6]	<10 [6]	<10 [6]	<10 [6]	<10 [6]
	Downgrade in security category	<10 [6]	<10 [6]	<10 [6]	<10 [6]	<10 [6]	<10 [6]	<10 [6]	<10 [6]	<10 [6]	<10 [6]
	Internal Cell Move	<10 [6]	<10 [6]	<10 [6]	<10 [6]	<10 [6]	<10 [6]	<10 [6]	<10 [6]	<10 [6]	<10 [6]
	Judges Remand	92	<10 [6]	<10 [6]	<10 [6]	<10 [6]	<10 [6]	<10 [6]	<10 [6]	<10 [6]	<10 [6]
	Licence Revoke	52	<10 [6]	<10 [6]	<10 [6]	<10 [6]	<10 [6]	<10 [6]	<10 [6]	<10 [6]	<10 [6]
	On Remand	1,492	15 (1.0)	<10 [6]	<10 [6]	<10 [6]	<10 [6]	<10 [6]	<10 [6]	<10 [6]	<10 [6]
	Transfer	910	<10 [6]	<10 [6]	<10 [6]	<10 [6]	<10 [6]	<10 [6]	<10 [6]	<10 [6]	<10 [6]
	Upgrade in security category	<10 [6]	<10 [6]	<10 [6]	<10 [6]	<10 [6]	<10 [6]	<10 [6]	<10 [6]	<10 [6]	<10 [6]
2018/19	.	88	<10 [6]	<10 [6]	<10 [6]	<10 [6]	<10 [6]	<10 [6]	<10 [6]	<10 [6]	<10 [6]
	Absconded	<10 [6]	<10 [6]	<10 [6]	<10 [6]	<10 [6]	<10 [6]	<10 [6]	<10 [6]	<10 [6]	<10 [6]
	Active In	18,145	266 (1.5)	<10 [6]	<10 [6]	26 (9.8)	10 (3.8)	25 (9.4)	12 (4.5)	39 (14.7)	20 (7.5)
	Active Out	835	12 (1.4)	<10 [6]	<10 [6]	<10 [6]	<10 [6]	<10 [6]	<10 [6]	<10 [6]	<10 [6]
	Convicted Sentence	1,320	24 (1.8)	<10 [6]	<10 [6]	<10 [6]	<10 [6]	<10 [6]	<10 [6]	<10 [6]	<10 [6]
	Downgrade in security category	<10 [6]	<10 [6]	<10 [6]	<10 [6]	<10 [6]	<10 [6]	<10 [6]	<10 [6]	<10 [6]	<10 [6]
	Internal Cell Move	<10 [6]	<10 [6]	<10 [6]	<10 [6]	<10 [6]	<10 [6]	<10 [6]	<10 [6]	<10 [6]	<10 [6]
	Judges Remand	<10 [6]	<10 [6]	<10 [6]	<10 [6]	<10 [6]	<10 [6]	<10 [6]	<10 [6]	<10 [6]	<10 [6]
	Licence Revoke	125	<10 [6]	<10 [6]	<10 [6]	<10 [6]	<10 [6]	<10 [6]	<10 [6]	<10 [6]	<10 [6]
	On Remand	1,059	23 (2.2)	<10 [6]	<10 [6]	<10 [6]	<10 [6]	<10 [6]	<10 [6]	<10 [6]	<10 [6]
	Transfer	518	<10 [6]	<10 [6]	<10 [6]	<10 [6]	<10 [6]	<10 [6]	<10 [6]	<10 [6]	<10 [6]
	Upgrade in security category	<10 [6]	<10 [6]	<10 [6]	<10 [6]	<10 [6]	<10 [6]	<10 [6]	<10 [6]	<10 [6]	<10 [6]
2019/20	.	69	<10 [6]	<10 [6]	<10 [6]	<10 [6]	<10 [6]	<10 [6]	<10 [6]	<10 [6]	<10 [6]
	Absconded	<10 [6]	<10 [6]	<10 [6]	<10 [6]	<10 [6]	<10 [6]	<10 [6]	<10 [6]	<10 [6]	<10 [6]
	Active In	22,424	411 (1.8)	<10 [6]	<10 [6]	25 (6.1)	<10 [6]	21 (5.1)	<10 [6]	31 (7.5)	<10 [6]
	Active Out	625	10 (1.6)	<10 [6]	<10 [6]	<10 [6]	<10 [6]	<10 [6]	<10 [6]	<10 [6]	<10 [6]
	Convicted Sentence	1,361	13 (1.0)	<10 [6]	<10 [6]	<10 [6]	<10 [6]	<10 [6]	<10 [6]	<10 [6]	<10 [6]
	Downgrade in security category	<10 [6]	<10 [6]	<10 [6]	<10 [6]	<10 [6]	<10 [6]	<10 [6]	<10 [6]	<10 [6]	<10 [6]
	Internal Cell Move	<10 [6]	<10 [6]	<10 [6]	<10 [6]	<10 [6]	<10 [6]	<10 [6]	<10 [6]	<10 [6]	<10 [6]
	Judges Remand	19	<10 [6]	<10 [6]	<10 [6]	<10 [6]	<10 [6]	<10 [6]	<10 [6]	<10 [6]	<10 [6]
	Licence Revoke	178	<10 [6]	<10 [6]	<10 [6]	<10 [6]	<10 [6]	<10 [6]	<10 [6]	<10 [6]	<10 [6]
	On Remand	1,031	23 (2.2)	<10 [6]	<10 [6]	<10 [6]	<10 [6]	<10 [6]	<10 [6]	<10 [6]	<10 [6]
	Transfer	101	<10 [6]	<10 [6]	<10 [6]	<10 [6]	<10 [6]	<10 [6]	<10 [6]	<10 [6]	<10 [6]
	Upgrade in security category	<10 [6]	<10 [6]	<10 [6]	<10 [6]	<10 [6]	<10 [6]	<10 [6]	<10 [6]	<10 [6]	<10 [6]
**Age - years**											
2017/18	10 - <20	468	<10 [6]	<10 [6]	<10 [6]	<10 [6]	<10 [6]	<10 [6]	<10 [6]	<10 [6]	<10 [6]
	20 - <30	6,994	37 (0.5)	<10 [6]	<10 [6]	<10 [6]	<10 [6]	<10 [6]	<10 [6]	14 (37.8)	<10 [6]
	30 - <40	7,051	94 (1.3)	<10 [6]	<10 [6]	27 (28.7)	<10 [6]	21 (22.3)	<10 [6]	42 (44.7)	<10 [6]
	40 - <50	4,114	60 (1.5)	<10 [6]	<10 [6]	19 (31.7)	<10 [6]	16 (26.7)	<10 [6]	26 (43.3)	<10 [6]
	50 - <60	2,107	35 (1.7)	<10 [6]	<10 [6]	15 (42.9)	<10 [6]	13 (37.1)	<10 [6]	19 (54.3)	<10 [6]
	60 - <70	684	<10 [6]	<10 [6]	<10 [6]	<10 [6]	<10 [6]	<10 [6]	<10 [6]	<10 [6]	<10 [6]
	70 - <80	213	<10 [6]	<10 [6]	<10 [6]	<10 [6]	<10 [6]	<10 [6]	<10 [6]	<10 [6]	<10 [6]
	80 - <90	40	<10 [6]	<10 [6]	<10 [6]	<10 [6]	<10 [6]	<10 [6]	<10 [6]	<10 [6]	<10 [6]
	90 - <100	<10 [6]	<10 [6]	<10 [6]	<10 [6]	<10 [6]	<10 [6]	<10 [6]	<10 [6]	<10 [6]	<10 [6]
	100 - <110	<10 [6]	<10 [6]	<10 [6]	<10 [6]	<10 [6]	<10 [6]	<10 [6]	<10 [6]	<10 [6]	<10 [6]

[1]Eligible for indicator; [2]Achieve in current prison; [3]Achieve in previous prison; [4]Overall achievement - either current or previous prison; [5]Declined indicator; [6]Suppressed (<10) to avoid disclosure

Indicator	*The proportion of people who have received three or more prescriptions for an antipsychotic in the preceding 12 months, and who have a record of blood testing (fasting glucose, HbA1c, lipid profile, urea and electrolytes, full blood count, liver function tests), body mass index and blood pressure*										
	**Group: Mental health**		**Community achievement 2019/20: No direct comparison in QOF - just percentage of patients with MH diagnosis of schizophrenia, bipolar affective disorder and other psychoses who have record of BP and BMI in the last 12 months (80.28% and 74.40% respectively)**								
			**Full blood count (% eligible)**		**Liver function (% eligible)**		**BMI (% eligible)**			**Blood Pressure (% eligible)**	
	**Variable**	**Eligible**	**Satisfy[2]**	**Elsewhere[3]**	**Satisfy[2]**	**Elsewhere[3]**	**Satisfy[2]**	**Elsewhere[3]**	**Declined[5]**	**Satisfy[2]**	**Elsewhere[3]**
Gender											
2017/18	F	42	26 (61.9)	<10 [6]	25 (59.5)	<10 [6]	34 (81.0)	<10 [6]	<10 [6]	40 (95.2)	<10 [6]
	M	202	90 (44.6)	10 (5.0)	82 (40.6)	<10 [6]	145 (71.8)	<10 [6]	<10 [6]	163 (80.7)	<10 [6]
2018/19	F	75	12 (16.0)	<10 [6]	12 (16.0)	<10 [6]	19 (25.3)	<10 [6]	<10 [6]	20 (26.7)	<10 [6]
	M	251	37 (14.7)	20 (8.0)	28 (11.2)	12 (4.8)	76 (30.3)	16 (6.4)	<10 [6]	81 (32.3)	15 (6.0)
2019/20	F	38	<10 [6]	<10 [6]	<10 [6]	<10 [6]	<10 [6]	<10 [6]	<10 [6]	<10 [6]	<10 [6]
	M	350	30 (8.6)	6 (1.7)	16 (4.6)	<10 [6]	19 (5.4)	10 (2.9)	<10 [6]	49 (14.0)	<10 [6]
Sentence Status											
2017/18	.	<10 [6]	<10 [6]	<10 [6]	<10 [6]	<10 [6]	<10 [6]	<10 [6]	<10 [6]	<10 [6]	<10 [6]
	Absconded	<10 [6]	<10 [6]	<10 [6]	<10 [6]	<10 [6]	<10 [6]	<10 [6]	<10 [6]	<10 [6]	<10 [6]
	Active In	187	95 (50.8)	11 (5.9)	88 (47.1)	<10 [6]	139 (74.3)	<10 [6]	<10 [6]	157 (84.0)	<10 [6]
	Active Out	13	<10 [6]	<10 [6]	<10 [6]	<10 [6]	<10 [6]	<10 [6]	<10 [6]	<10 [6]	<10 [6]
	Convicted Sentence	22	<10 [6]	<10 [6]	<10 [6]	<10 [6]	17 (77.3)	<10 [6]	<10 [6]	20 (90.9)	<10 [6]
	Downgrade in security category	<10 [6]	<10 [6]	<10 [6]	<10 [6]	<10 [6]	<10 [6]	<10 [6]	<10 [6]	<10 [6]	<10 [6]
	Internal Cell Move	<10 [6]	<10 [6]	<10 [6]	<10 [6]	<10 [6]	<10 [6]	<10 [6]	<10 [6]	<10 [6]	<10 [6]
	Judges Remand	<10 [6]	<10 [6]	<10 [6]	<10 [6]	<10 [6]	<10 [6]	<10 [6]	<10 [6]	<10 [6]	<10 [6]
	Licence Revoke	<10 [6]	<10 [6]	<10 [6]	<10 [6]	<10 [6]	<10 [6]	<10 [6]	<10 [6]	<10 [6]	<10 [6]
	On Remand	15	<10 [6]	<10 [6]	<10 [6]	<10 [6]	12 (80.0)	<10 [6]	<10 [6]	12 (80.0)	<10 [6]
	Transfer	<10 [6]	<10 [6]	<10 [6]	<10 [6]	<10 [6]	<10 [6]	<10 [6]	<10 [6]	<10 [6]	<10 [6]
	Upgrade in security category	<10 [6]	<10 [6]	<10 [6]	<10 [6]	<10 [6]	<10 [6]	<10 [6]	<10 [6]	<10 [6]	<10 [6]
2018/19											
	.	<10 [6]	<10 [6]	<10 [6]	<10 [6]	<10 [6]	<10 [6]	<10 [6]	<10 [6]	<10 [6]	<10 [6]
	Absconded	<10 [6]	<10 [6]	<10 [6]	<10 [6]	<10 [6]	<10 [6]	<10 [6]	<10 [6]	<10 [6]	<10 [6]
	Active In	266	42 (15.8)	21 (7.9)	34 (12.8)	13 (4.9)	84 (31.6)	16 (6.0)	<10 [6]	88 (33.1)	16 (6.0)
	Active Out	12	<10 [6]	<10 [6]	<10 [6]	<10 [6]	<10 [6]	<10 [6]	<10 [6]	<10 [6]	<10 [6]
	Convicted Sentence	24	<10 [6]	<10 [6]	<10 [6]	<10 [6]	<10 [6]	<10 [6]	<10 [6]	<10 [6]	<10 [6]
	Downgrade in security category	<10 [6]	<10 [6]	<10 [6]	<10 [6]	<10 [6]	<10 [6]	<10 [6]	<10 [6]	<10 [6]	<10 [6]
	Internal Cell Move	<10 [6]	<10 [6]	<10 [6]	<10 [6]	<10 [6]	<10 [6]	<10 [6]	<10 [6]	<10 [6]	<10 [6]
	Judges Remand	<10 [6]	<10 [6]	<10 [6]	<10 [6]	<10 [6]	<10 [6]	<10 [6]	<10 [6]	<10 [6]	<10 [6]
	Licence Revoke	<10 [6]	<10 [6]	<10 [6]	<10 [6]	<10 [6]	<10 [6]	<10 [6]	<10 [6]	<10 [6]	<10 [6]
	On Remand	23	<10 [6]	<10 [6]	<10 [6]	<10 [6]	<10 [6]	<10 [6]	<10 [6]	<10 [6]	<10 [6]
	Transfer	<10 [6]	<10 [6]	<10 [6]	<10 [6]	<10 [6]	<10 [6]	<10 [6]	<10 [6]	<10 [6]	<10 [6]
	Upgrade in security category	<10 [6]	<10 [6]	<10 [6]	<10 [6]	<10 [6]	<10 [6]	<10 [6]	<10 [6]	<10 [6]	<10 [6]
2019/20	.	<10 [6]	<10 [6]	<10 [6]	<10 [6]	<10 [6]	<10 [6]	<10 [6]	<10 [6]	<10 [6]	<10 [6]
	Absconded	<10 [6]	<10 [6]	<10 [6]	<10 [6]	<10 [6]	<10 [6]	<10 [6]	<10 [6]	<10 [6]	<10 [6]
	Active In	411	32 (7.8)	<10 [6]	15 (3.6)	<10 [6]	20 (4.9)	11 (2.7)	<10 [6]	54 (13.1)	<10 [6]
	Active Out	10	<10 [6]	<10 [6]	<10 [6]	<10 [6]	<10 [6]	<10 [6]	<10 [6]	<10 [6]	<10 [6]
	Convicted Sentence	13	<10 [6]	<10 [6]	<10 [6]	<10 [6]	<10 [6]	<10 [6]	<10 [6]	<10 [6]	<10 [6]
	Downgrade in security category	<10 [6]	<10 [6]	<10 [6]	<10 [6]	<10 [6]	<10 [6]	<10 [6]	<10 [6]	<10 [6]	<10 [6]
	Internal Cell Move	<10 [6]	<10 [6]	<10 [6]	<10 [6]	<10 [6]	<10 [6]	<10 [6]	<10 [6]	<10 [6]	<10 [6]
	Judges Remand	<10 [6]	<10 [6]	<10 [6]	<10 [6]	<10 [6]	<10 [6]	<10 [6]	<10 [6]	<10 [6]	<10 [6]
	Licence Revoke	<10 [6]	<10 [6]	<10 [6]	<10 [6]	<10 [6]	<10 [6]	<10 [6]	<10 [6]	<10 [6]	<10 [6]
	On Remand	23	<10 [6]	<10 [6]	<10 [6]	<10 [6]	<10 [6]	<10 [6]	<10 [6]	<10 [6]	<10 [6]
	Transfer	<10 [6]	<10 [6]	<10 [6]	<10 [6]	<10 [6]	<10 [6]	<10 [6]	<10 [6]	<10 [6]	<10 [6]
	Upgrade in security category	<10 [6]	<10 [6]	<10 [6]	<10 [6]	<10 [6]	<10 [6]	<10 [6]	<10 [6]	<10 [6]	<10 [6]
Age - years											
2017/18	10 - <20	<10 [6]	<10 [6]	<10 [6]	<10 [6]	<10 [6]	<10 [6]	<10 [6]	<10 [6]	<10 [6]	<10 [6]
	20 - <30	37	14 (37.8)	<10 [6]	15 (40.5)	<10 [6]	31 (83.8)	<10 [6]	<10 [6]	32 (86.5)	<10 [6]
	30 - <40	94	43 (45.7)	<10 [6]	40 (42.6)	<10 [6]	65 (69.1)	<10 [6]	<10 [6]	74 (78.7)	<10 [6]
	40 - <50	60	28 (46.7)	<10 [6]	24 (40.0)	<10 [6]	45 (75.0)	<10 [6]	<10 [6]	52 (86.7)	<10 [6]
	50 - <60	35	21 (60.0)	<10 [6]	18 (51.4)	<10 [6]	26 (74.3)	<10 [6]	<10 [6]	29 (82.9)	<10 [6]
	60 - <70	<10 [6]	<10 [6]	<10 [6]	<10 [6]	<10 [6]	<10 [6]	<10 [6]	<10 [6]	<10 [6]	<10 [6]
	70 - <80	<10 [6]	<10 [6]	<10 [6]	<10 [6]	<10 [6]	<10 [6]	<10 [6]	<10 [6]	<10 [6]	<10 [6]
	80 - <90	<10 [6]	<10 [6]	<10 [6]	<10 [6]	<10 [6]	<10 [6]	<10 [6]	<10 [6]	<10 [6]	<10 [6]
	90 - <100	<10 [6]	<10 [6]	<10 [6]	<10 [6]	<10 [6]	<10 [6]	<10 [6]	<10 [6]	<10 [6]	<10 [6]
	100 - <110	<10 [6]	<10 [6]	<10 [6]	<10 [6]	<10 [6]	<10 [6]	<10 [6]	<10 [6]	<10 [6]	<10 [6]

[1]Eligible for indicator; [2]Achieve in current prison; [3]Achieve in previous prison; [4]Overall achievement - either current or previous prison; [5]Declined indicator; [6]Suppressed (<10) to avoid disclosure

Indicator	*The proportion of people who have received three or more prescriptions for an antipsychotic in the preceding 12 months, and who have a record of blood testing (fasting glucose, HbA1c, lipid profile, urea and electrolytes, full blood count, liver function tests), body mass index and blood pressure*			***Community achievement 2019/20: No direct comparison in QOF - just percentage of patients with MH diagnosis of schizophrenia, bipolar affective disorder and other psychoses who have record of BP and BMI in the last 12 months (80.28% and 74.40% respectively)***							
	**Group: Mental health**			**Fasting glucose (% eligible)**		**HbA1c (% eligible)**		**Lipid profile (% eligible)**		**Urea and electrolytes (% eligible)**	
	**Variable**	**Population**	**Eligible (% popln)**	**Satisfy[2]**	**Elsewhere[3]**	**Satisfy[2]**	**Elsewhere[3]**	**Satisfy[2]**	**Elsewhere[3]**	**Satisfy[2]**	**Elsewhere[3]**
2018/19	10 - <20	436	<10 [6]	<10 [6]	<10 [6]	<10 [6]	<10 [6]	<10 [6]	<10 [6]	<10 [6]	<10 [6]
	20 - <30	7,163	68 (0.9)	<10 [6]	<10 [6]	<10 [6]	<10 [6]	<10 [6]	<10 [6]	<10 [6]	<10 [6]
	30 - <40	7,381	124 (1.7)	<10 [6]	<10 [6]	11 (8.9)	<10 [6]	<10 [6]	<10 [6]	14 (11.3)	10 (8.1)
	40 - <50	4,180	85 (2.0)	<10 [6]	<10 [6]	<10 [6]	<10 [6]	<10 [6]	<10 [6]	14 (16.5)	<10 [6]
	50 - <60	1,978	31 (1.6)	<10 [6]	<10 [6]	<10 [6]	<10 [6]	<10 [6]	<10 [6]	<10 [6]	<10 [6]
	60 - <70	701	<10 [6]	<10 [6]	<10 [6]	<10 [6]	<10 [6]	<10 [6]	<10 [6]	<10 [6]	<10 [6]
	70 - <80	209	<10 [6]	<10 [6]	<10 [6]	<10 [6]	<10 [6]	<10 [6]	<10 [6]	<10 [6]	<10 [6]
	80 - <90	45	<10 [6]	<10 [6]	<10 [6]	<10 [6]	<10 [6]	<10 [6]	<10 [6]	<10 [6]	<10 [6]
	90 - <100	<10 [6]	<10 [6]	<10 [6]	<10 [6]	<10 [6]	<10 [6]	<10 [6]	<10 [6]	<10 [6]	<10 [6]
	100 - <110	<10 [6]	<10 [6]	<10 [6]	<10 [6]	<10 [6]	<10 [6]	<10 [6]	<10 [6]	<10 [6]	<10 [6]
2019/20	10 - <20	404	<10 [6]	<10 [6]	<10 [6]	<10 [6]	<10 [6]	<10 [6]	<10 [6]	<10 [6]	<10 [6]
	20 - <30	8,064	92 (1.1)	<10 [6]	<10 [6]	<10 [6]	<10 [6]	<10 [6]	<10 [6]	<10 [6]	<10 [6]
	30 - <40	9,125	188 (2.1)	<10 [6]	<10 [6]	<10 [6]	<10 [6]	<10 [6]	<10 [6]	14 (7.4)	<10 [6]
	40 - <50	4,948	130 (2.6)	<10 [6]	<10 [6]	<10 [6]	<10 [6]	<10 [6]	<10 [6]	<10 [6]	<10 [6]
	50 - <60	2,224	35 (1.6)	<10 [6]	<10 [6]	<10 [6]	<10 [6]	<10 [6]	<10 [6]	<10 [6]	<10 [6]
	60 - <70	751	<10 [6]	<10 [6]	<10 [6]	<10 [6]	<10 [6]	<10 [6]	<10 [6]	<10 [6]	<10 [6]
	70 - <80	238	<10 [6]	<10 [6]	<10 [6]	<10 [6]	<10 [6]	<10 [6]	<10 [6]	<10 [6]	<10 [6]
	80 - <90	53	<10 [6]	<10 [6]	<10 [6]	<10 [6]	<10 [6]	<10 [6]	<10 [6]	<10 [6]	<10 [6]
	90 - <100	<10 [6]	<10 [6]	<10 [6]	<10 [6]	<10 [6]	<10 [6]	<10 [6]	<10 [6]	<10 [6]	<10 [6]
	100 - <110	<10 [6]	<10 [6]	<10 [6]	<10 [6]	<10 [6]	<10 [6]	<10 [6]	<10 [6]	<10 [6]	<10 [6]
**Length of Stay (months)**											
2017/18	<1	4,474	19 (0.4)	<10 [6]	<10 [6]	<10 [6]	<10 [6]	<10 [6]	<10 [6]	<10 [6]	<10 [6]
	1-<6	8,075	65 (0.8)	<10 [6]	<10 [6]	13 (20.0)	<10 [6]	<10 [6]	<10 [6]	24 (36.9)	<10 [6]
	6-<12	3,672	51 (1.4)	<10 [6]	<10 [6]	14 (27.5)	<10 [6]	10 (19.6)	<10 [6]	23 (45.1)	<10 [6]
	12-<24	2,832	48 (1.7)	<10 [6]	<10 [6]	14 (29.2)	<10 [6]	12 (25.0)	<10 [6]	21 (43.8)	<10 [6]
	24+	2,624	61 (2.3)	<10 [6]	<10 [6]	32 (52.5)	<10 [6]	28 (45.9)	<10 [6]	40 (65.6)	<10 [6]
2018/19	<1	4,801	49 (1.0)	<10 [6]	<10 [6]	<10 [6]	<10 [6]	<10 [6]	<10 [6]	<10 [6]	<10 [6]
	1-<6	7,742	106 (1.4)	<10 [6]	<10 [6]	<10 [6]	<10 [6]	<10 [6]	<10 [6]	<10 [6]	<10 [6]
	6-<12	3,616	54 (1.5)	<10 [6]	<10 [6]	<10 [6]	<10 [6]	<10 [6]	<10 [6]	<10 [6]	<10 [6]
	12-<24	3,447	57 (1.7)	<10 [6]	<10 [6]	<10 [6]	<10 [6]	<10 [6]	<10 [6]	10 (17.5)	<10 [6]
	24+	2,493	60 (2.4)	<10 [6]	<10 [6]	13 (21.7)	<10 [6]	13 (21.7)	<10 [6]	17 (28.3)	<10 [6]
2019/20	<1	5,745	70 (1.2)	<10 [6]	<10 [6]	<10 [6]	<10 [6]	<10 [6]	<10 [6]	<10 [6]	<10 [6]
	1-<6	9,697	177 (1.8)	<10 [6]	<10 [6]	10 (5.6)	<10 [6]	<10 [6]	<10 [6]	12 (6.8)	<10 [6]
	6-<12	5,090	120 (2.4)	<10 [6]	<10 [6]	<10 [6]	<10 [6]	<10 [6]	<10 [6]	<10 [6]	<10 [6]
	12-<24	3,244	52 (1.6)	<10 [6]	<10 [6]	<10 [6]	<10 [6]	<10 [6]	<10 [6]	<10 [6]	<10 [6]
	24+	2,035	44 (2.2)	<10 [6]	<10 [6]	10 (22.7)	<10 [6]	11 (25.0)	<10 [6]	11 (25.0)	<10 [6]
**Ethnic Group**											
2017/18	White	15,638	192 (1.2)	<10 [6]	<10 [6]	56 (29.2)	<10 [6]	40 (20.8)	<10 [6]	86 (44.8)	11 (5.7)
	Mixed	431	<10 [6]	<10 [6]	<10 [6]	<10 [6]	<10 [6]	<10 [6]	<10 [6]	<10 [6]	<10 [6]
	Asian or Asian British	813	<10 [6]	<10 [6]	<10 [6]	<10 [6]	<10 [6]	<10 [6]	<10 [6]	<10 [6]	<10 [6]
	Black or Black British	404	<10 [6]	<10 [6]	<10 [6]	<10 [6]	<10 [6]	<10 [6]	<10 [6]	<10 [6]	<10 [6]
	Chinese and Other	214	<10 [6]	<10 [6]	<10 [6]	<10 [6]	<10 [6]	<10 [6]	<10 [6]	<10 [6]	<10 [6]
	Unclassified	372	<10 [6]	<10 [6]	<10 [6]	<10 [6]	<10 [6]	<10 [6]	<10 [6]	<10 [6]	<10 [6]
2018/19	White	14,911	266 (1.8)	<10 [6]	<10 [6]	23 (8.6)	<10 [6]	21 (7.9)	12 (4.5)	36 (13.5)	18 (6.8)
	Mixed	371	<10 [6]	<10 [6]	<10 [6]	<10 [6]	<10 [6]	<10 [6]	<10 [6]	<10 [6]	<10 [6]
	Asian or Asian British	726	<10 [6]	<10 [6]	<10 [6]	<10 [6]	<10 [6]	<10 [6]	<10 [6]	<10 [6]	<10 [6]
	Black or Black British	364	<10 [6]	<10 [6]	<10 [6]	<10 [6]	<10 [6]	<10 [6]	<10 [6]	<10 [6]	<10 [6]
	Chinese and Other	167	<10 [6]	<10 [6]	<10 [6]	<10 [6]	<10 [6]	<10 [6]	<10 [6]	<10 [6]	<10 [6]
	Unclassified	409	<10 [6]	<10 [6]	<10 [6]	<10 [6]	<10 [6]	<10 [6]	<10 [6]	<10 [6]	<10 [6]
2019/20	White	16,606	364 (2.2)	<10 [6]	<10 [6]	24 (6.6)	<10 [6]	17 (4.7)	<10 [6]	29 (8.0)	<10 [6]
	Mixed	409	<10 [6]	<10 [6]	<10 [6]	<10 [6]	<10 [6]	<10 [6]	<10 [6]	<10 [6]	<10 [6]
	Asian or Asian British	755	<10 [6]	<10 [6]	<10 [6]	<10 [6]	<10 [6]	<10 [6]	<10 [6]	<10 [6]	<10 [6]
	Black or Black British	451	<10 [6]	<10 [6]	<10 [6]	<10 [6]	<10 [6]	<10 [6]	<10 [6]	<10 [6]	<10 [6]
	Chinese and Other	163	<10 [6]	<10 [6]	<10 [6]	<10 [6]	<10 [6]	<10 [6]	<10 [6]	<10 [6]	<10 [6]
	Unclassified	387	11 (2.8)	<10 [6]	<10 [6]	<10 [6]	<10 [6]	<10 [6]	<10 [6]	<10 [6]	<10 [6]

[1]Eligible for indicator; [2]Achieve in current prison; [3]Achieve in previous prison; [4]Overall achievement - either current or previous prison; [5]Declined indicator; [6]Suppressed (<10) to avoid disclosure

Indicator — ***The proportion of people who have received three or more prescriptions for an antipsychotic in the preceding 12 months, and who have a record of blood testing (fasting glucose, HbA1c, lipid profile, urea and electrolytes, full blood count, liver function tests), body mass index and blood pressure***

Group: Mental health

Community achievement 2019/20: No direct comparison in QOF - just percentage of patients with MH diagnosis of schizophrenia, bipolar affective disorder and other psychoses who have record of BP and BMI in the last 12 months (80.28% and 74.40% respectively)

			Full blood count (% eligible)		Liver function (% eligible)		BMI (% eligible)			Blood Pressure (% eligible)	
	**Variable**	**Eligible**	**Satisfy[2]**	**Elsewhere[3]**	**Satisfy[2]**	**Elsewhere[3]**	**Satisfy[2]**	**Elsewhere[3]**	**Declined[5]**	**Satisfy[2]**	**Elsewhere[3]**
2018/19	10 - <20	<10 [6]	<10 [6]	<10 [6]	<10 [6]	<10 [6]	<10 [6]	<10 [6]	<10 [6]	<10 [6]	<10 [6]
	20 - <30	68	<10 [6]	<10 [6]	<10 [6]	<10 [6]	19 (27.9)	<10 [6]	<10 [6]	21 (30.9)	<10 [6]
	30 - <40	124	15 (12.1)	10 (8.1)	15 (12.1)	<10 [6]	32 (25.8)	<10 [6]	<10 [6]	32 (25.8)	<10 [6]
	40 - <50	85	14 (16.5)	<10 [6]	10 (11.8)	<10 [6]	24 (28.2)	<10 [6]	<10 [6]	25 (29.4)	<10 [6]
	50 - <60	31	<10 [6]	<10 [6]	<10 [6]	<10 [6]	<10 [6]	<10 [6]	<10 [6]	11 (35.5)	<10 [6]
	60 - <70	<10 [6]	<10 [6]	<10 [6]	<10 [6]	<10 [6]	<10 [6]	<10 [6]	<10 [6]	<10 [6]	<10 [6]
	70 - <80	<10 [6]	<10 [6]	<10 [6]	<10 [6]	<10 [6]	<10 [6]	<10 [6]	<10 [6]	<10 [6]	<10 [6]
	80 - <90	<10 [6]	<10 [6]	<10 [6]	<10 [6]	<10 [6]	<10 [6]	<10 [6]	<10 [6]	<10 [6]	<10 [6]
	90 - <100	<10 [6]	<10 [6]	<10 [6]	<10 [6]	<10 [6]	<10 [6]	<10 [6]	<10 [6]	<10 [6]	<10 [6]
	100 - <110	<10 [6]	<10 [6]	<10 [6]	<10 [6]	<10 [6]	<10 [6]	<10 [6]	<10 [6]	<10 [6]	<10 [6]
2019/20	10 - <20	<10 [6]	<10 [6]	<10 [6]	<10 [6]	<10 [6]	<10 [6]	<10 [6]	<10 [6]	<10 [6]	<10 [6]
	20 - <30	92	<10 [6]	<10 [6]	<10 [6]	<10 [6]	<10 [6]	<10 [6]	<10 [6]	<10 [6]	<10 [6]
	30 - <40	188	14 (7.4)	<10 [6]	<10 [6]	<10 [6]	<10 [6]	<10 [6]	<10 [6]	23 (12.2)	<10 [6]
	40 - <50	130	10 (7.7)	<10 [6]	<10 [6]	<10 [6]	<10 [6]	<10 [6]	<10 [6]	20 (15.4)	<10 [6]
	50 - <60	35	<10 [6]	<10 [6]	<10 [6]	<10 [6]	<10 [6]	<10 [6]	<10 [6]	<10 [6]	<10 [6]
	60 - <70	<10 [6]	<10 [6]	<10 [6]	<10 [6]	<10 [6]	<10 [6]	<10 [6]	<10 [6]	<10 [6]	<10 [6]
	70 - <80	<10 [6]	<10 [6]	<10 [6]	<10 [6]	<10 [6]	<10 [6]	<10 [6]	<10 [6]	<10 [6]	<10 [6]
	80 - <90	<10 [6]	<10 [6]	<10 [6]	<10 [6]	<10 [6]	<10 [6]	<10 [6]	<10 [6]	<10 [6]	<10 [6]
	90 - <100	<10 [6]	<10 [6]	<10 [6]	<10 [6]	<10 [6]	<10 [6]	<10 [6]	<10 [6]	<10 [6]	<10 [6]
	100 - <110	<10 [6]	<10 [6]	<10 [6]	<10 [6]	<10 [6]	<10 [6]	<10 [6]	<10 [6]	<10 [6]	<10 [6]
**Length of Stay (months)**											
2017/18	<1	19	<10 [6]	<10 [6]	<10 [6]	<10 [6]	18 (94.7)	<10 [6]	<10 [6]	18 (94.7)	<10 [6]
	1-<6	65	23 (35.4)	<10 [6]	22 (33.8)	<10 [6]	49 (75.4)	<10 [6]	<10 [6]	56 (86.2)	<10 [6]
	6-<12	51	24 (47.1)	<10 [6]	23 (45.1)	<10 [6]	41 (80.4)	<10 [6]	<10 [6]	42 (82.4)	<10 [6]
	12-<24	48	22 (45.8)	<10 [6]	19 (39.6)	<10 [6]	29 (60.4)	<10 [6]	<10 [6]	34 (70.8)	<10 [6]
	24+	61	43 (70.5)	<10 [6]	40 (65.6)	<10 [6]	42 (68.9)	<10 [6]	<10 [6]	53 (86.9)	<10 [6]
2018/19	<1	49	<10 [6]	<10 [6]	<10 [6]	<10 [6]	12 (24.5)	<10 [6]	<10 [6]	13 (26.5)	<10 [6]
	1-<6	106	<10 [6]	<10 [6]	<10 [6]	<10 [6]	20 (18.9)	<10 [6]	<10 [6]	20 (18.9)	<10 [6]
	6-<12	54	<10 [6]	<10 [6]	<10 [6]	<10 [6]	16 (29.6)	<10 [6]	<10 [6]	17 (31.5)	<10 [6]
	12-<24	57	10 (17.5)	<10 [6]	<10 [6]	<10 [6]	22 (38.6)	<10 [6]	<10 [6]	23 (40.4)	<10 [6]
	24+	60	19 (31.7)	<10 [6]	17 (28.3)	<10 [6]	25 (41.7)	<10 [6]	<10 [6]	28 (46.7)	<10 [6]
2019/20	<1	70	<10 [6]	<10 [6]	<10 [6]	<10 [6]	<10 [6]	<10 [6]	<10 [6]	<10 [6]	<10 [6]
	1-<6	177	12 (6.8)	<10 [6]	<10 [6]	<10 [6]	<10 [6]	<10 [6]	<10 [6]	18 (10.2)	<10 [6]
	6-<12	120	<10 [6]	<10 [6]	<10 [6]	<10 [6]	<10 [6]	<10 [6]	<10 [6]	11 (9.2)	<10 [6]
	12-<24	52	<10 [6]	<10 [6]	<10 [6]	<10 [6]	<10 [6]	<10 [6]	<10 [6]	<10 [6]	<10 [6]
	24+	44	12 (27.3)	<10 [6]	<10 [6]	<10 [6]	11 (25.0)	<10 [6]	<10 [6]	17 (38.6)	<10 [6]
**Ethnic Group**											
2017/18	White	192	89 (46.4)	10 (5.2)	82 (42.7)	<10 [6]	142 (74.0)	<10 [6]	<10 [6]	161 (83.9)	<10 [6]
	Mixed	<10 [6]	<10 [6]	<10 [6]	<10 [6]	<10 [6]	<10 [6]	<10 [6]	<10 [6]	<10 [6]	<10 [6]
	Asian or Asian British	<10 [6]	<10 [6]	<10 [6]	<10 [6]	<10 [6]	<10 [6]	<10 [6]	<10 [6]	<10 [6]	<10 [6]
	Black or Black British	<10 [6]	<10 [6]	<10 [6]	<10 [6]	<10 [6]	<10 [6]	<10 [6]	<10 [6]	<10 [6]	<10 [6]
	Chinese and Other	<10 [6]	<10 [6]	<10 [6]	<10 [6]	<10 [6]	<10 [6]	<10 [6]	<10 [6]	<10 [6]	<10 [6]
	Unclassified	<10 [6]	<10 [6]	<10 [6]	<10 [6]	<10 [6]	<10 [6]	<10 [6]	<10 [6]	<10 [6]	<10 [6]
2018/19	White	266	39 (14.7)	19 (7.1)	31 (11.7)	12 (4.5)	77 (28.9)	15 (5.6)	<10 [6]	84 (31.6)	14 (5.3)
	Mixed	<10 [6]	<10 [6]	<10 [6]	<10 [6]	<10 [6]	<10 [6]	<10 [6]	<10 [6]	<10 [6]	<10 [6]
	Asian or Asian British	<10 [6]	<10 [6]	<10 [6]	<10 [6]	<10 [6]	<10 [6]	<10 [6]	<10 [6]	<10 [6]	<10 [6]
	Black or Black British	<10 [6]	<10 [6]	<10 [6]	<10 [6]	<10 [6]	<10 [6]	<10 [6]	<10 [6]	<10 [6]	<10 [6]
	Chinese and Other	<10 [6]	<10 [6]	<10 [6]	<10 [6]	<10 [6]	<10 [6]	<10 [6]	<10 [6]	<10 [6]	<10 [6]
	Unclassified	<10 [6]	<10 [6]	<10 [6]	<10 [6]	<10 [6]	<10 [6]	<10 [6]	<10 [6]	<10 [6]	<10 [6]
2019/20	White	364	31 (8.5)	<10 [6]	12 (3.3)	<10 [6]	15 (4.1)	10 (2.7)	<10 [6]	55 (15.1)	<10 [6]
	Mixed	<10 [6]	<10 [6]	<10 [6]	<10 [6]	<10 [6]	<10 [6]	<10 [6]	<10 [6]	<10 [6]	<10 [6]
	Asian or Asian British	<10 [6]	<10 [6]	<10 [6]	<10 [6]	<10 [6]	<10 [6]	<10 [6]	<10 [6]	<10 [6]	<10 [6]
	Black or Black British	<10 [6]	<10 [6]	<10 [6]	<10 [6]	<10 [6]	<10 [6]	<10 [6]	<10 [6]	<10 [6]	<10 [6]
	Chinese and Other	<10 [6]	<10 [6]	<10 [6]	<10 [6]	<10 [6]	<10 [6]	<10 [6]	<10 [6]	<10 [6]	<10 [6]
	Unclassified	11	<10 [6]	<10 [6]	<10 [6]	<10 [6]	<10 [6]	<10 [6]	<10 [6]	<10 [6]	<10 [6]

[1]Eligible for indicator; [2]Achieve in current prison; [3]Achieve in previous prison; [4]Overall achievement - either current or previous prison; [5]Declined indicator; [6]Suppressed (<10) to avoid disclosure

Indicator	The proportion of people who have received three or more prescriptions for an antipsychotic in the preceding 12 months, and who have a record of blood testing (fasting glucose, HbA1c, lipid profile, urea and electrolytes, full blood count, liver function tests), body mass index and blood pressure<br>Group: Mental health		Community achievement 2019/20 (%): No direct comparison in QOF - just percentage of patients with MH diagnosis of schizophrenia, bipolar affective disorder and other psychoses who have record of BP and BMI in the last 12 months (80.28% and 74.40% respectively)
	**Variable**	**Population**	**Eligible[1] (% population)**	**Achieved[4] 4 of the 6 blood tests (% eligible)**
**Year**				
	2017/18	21,677	244 (1.1)	88 (36.1)
	2018/19	22,099	326 (1.5)	45 (13.8)
	2019/20	25,811	463 (1.8)	24 (5.2)
**Prison**				
2017/18	Prison 1	1,323	11 (0.8)	<10 [6]
	Prison 2	3,261	36 (1.1)	<10 [6]
	Prison 3	2,623	38 (1.4)	10 (26.3)
	Prison 4	2,089	22 (1.1)	<10 [6]
	Prison 5	637	<10 [6]	<10 [6]
	Prison 6	1,552	<10 [6]	<10 [6]
	Prison 7	635	15 (2.4)	<10 [6]
	Prison 8	1,085	27 (2.5)	12 (44.4)
	Prison 9	981	28 (2.9)	18 (64.3)
	Prison 10	2,523	19 (0.8)	<10 [6]
	Prison 11	3,470	23 (0.7)	<10 [6]
	Prison 12	815	<10 [6]	<10 [6]
	Prison 13	683	15 (2.2)	11 (73.3)
2018/19	Prison 1	1,333	16 (1.2)	<10 [6]
	Prison 2	2,705	43 (1.6)	<10 [6]
	Prison 3	2,522	34 (1.3)	<10 [6]
	Prison 4	2,349	37 (1.6)	<10 [6]
	Prison 5	676	<10 [6]	<10 [6]
	Prison 6	1,513	<10 [6]	<10 [6]
	Prison 7	654	45 (6.9)	<10 [6]
	Prison 8	1,148	30 (2.6)	<10 [6]
	Prison 9	996	28 (2.8)	13 (46.4)
	Prison 10	2,717	<10 [6]	<10 [6]
	Prison 11	4,020	73 (1.8)	<10 [6]
	Prison 12	792	<10 [6]	<10 [6]
	Prison 13	674	17 (2.5)	<10 [6]
2019/20	Prison 1	1,410	16 (1.1)	<10 [6]
	Prison 2	2,979	43 (1.4)	<10 [6]
	Prison 3	2,809	55 (2.0)	<10 [6]
	Prison 4	2,651	35 (1.3)	<10 [6]
	Prison 5	616	<10 [6]	<10 [6]
	Prison 6	1,533	<10 [6]	<10 [6]
	Prison 7	860	75 (8.7)	<10 [6]
	Prison 8	1,385	38 (2.7)	<10 [6]
	Prison 9	1,092	31 (2.8)	12 (38.7)
	Prison 10	3,577	38 (1.1)	<10 [6]
	Prison 11	5,348	97 (1.8)	<10 [6]
	Prison 12	805	<10 [6]	<10 [6]
	Prison 13	746	21 (2.8)	<10 [6]
**Prison category**				
2017/18	A	1,664	43 (2.6)	29 (67.4)
	B	9,254	78 (0.8)	15 (19.2)
	C	6,035	71 (1.2)	20 (28.2)
	Closed	1,720	42 (2.4)	20 (47.6)
	D	2,189	<10 [6]	<10 [6]
	YOI	815	<10 [6]	<10 [6]
2018/19	A	1,670	45 (2.7)	19 (42.2)
	B	9,442	116 (1.2)	<10 [6]
	C	6,204	87 (1.4)	11 (12.6)
	Closed	1,802	75 (4.2)	<10 [6]
	D	2,189	<10 [6]	<10 [6]
	YOI	792	<10 [6]	<10 [6]
2019/20	A	1,838	52 (2.8)	14 (26.9)
	B	11,904	178 (1.5)	<10 [6]
	C	6,870	106 (1.5)	<10 [6]
	Closed	2,245	113 (5.0)	<10 [6]
	D	2,149	11 (0.5)	<10 [6]
	YOI	805	<10 [6]	<10 [6]
**Gender**				
2017/18	F	1,699	42 (2.5)	20 (47.6)
	M	19,977	202 (1.0)	68 (33.7)
2018/19	F	1,802	75 (4.2)	<10 [6]
	M	20,295	251 (1.2)	37 (14.7)
2019/20	F	1,376	38 (2.8)	<10 [6]
	M	23,570	350 (1.5)	20 (5.7)

[1]Eligible for indicator; [2]Achieve in current prison; [3]Achieve in previous prison; [4]Overall achievement - either current or previous prison; [5]Declined indicator; [6]Suppressed (<10) to avoid disclosure

Indicator

The proportion of people who have received three or more prescriptions for an antipsychotic in the preceding 12 months, and who have a record of blood testing (fasting glucose, HbA1c, lipid profile, urea and electrolytes, full blood count, liver function tests), body mass index and blood pressure

Group: Mental health

Community achievement 2019/20 (%): No direct comparison in QOF - just percentage of patients with MH diagnosis of schizophrenia, bipolar affective disorder and other psychoses who have record of BP and BMI in the last 12 months (80.28% and 74.40% respectively)

	Variable	Population	Eligible[1] (% population)	Achieved[4] 4 of the 6 blood tests (% eligible)
**Sentence Status**				
2017/18	.	81	<10 [6]	<10 [6]
	Absconded	<10 [6]	<10 [6]	<10 [6]
	Active In	15,872	187 (1.2)	75 (40.1)
	Active Out	1,052	13 (1.2)	<10 [6]
	Convicted Sentence	2,125	22 (1.0)	<10 [6]
	Downgrade in security category	<10 [6]	<10 [6]	<10 [6]
	Internal Cell Move	<10 [6]	<10 [6]	<10 [6]
	Judges Remand	92	<10 [6]	<10 [6]
	Licence Revoke	52	<10 [6]	<10 [6]
	On Remand	1,492	15 (1.0)	<10 [6]
	Transfer	910	<10 [6]	<10 [6]
	Upgrade in security category	<10 [6]	<10 [6]	<10 [6]
2018/19	.	88	<10 [6]	<10 [6]
	Absconded	<10 [6]	<10 [6]	<10 [6]
	Active In	18,145	266 (1.5)	39 (14.7)
	Active Out	835	12 (1.4)	<10 [6]
	Convicted Sentence	1,320	24 (1.8)	<10 [6]
	Downgrade in security category	<10 [6]	<10 [6]	<10 [6]
	Internal Cell Move	<10 [6]	<10 [6]	<10 [6]
	Judges Remand	<10 [6]	<10 [6]	<10 [6]
	Licence Revoke	125	<10 [6]	<10 [6]
	On Remand	1,059	23 (2.2)	<10 [6]
	Transfer	518	<10 [6]	<10 [6]
	Upgrade in security category	<10 [6]	<10 [6]	<10 [6]
2019/20	.	69	<10 [6]	<10 [6]
	Absconded	<10 [6]	<10 [6]	<10 [6]
	Active In	22,424	411 (1.8)	22 (5.4)
	Active Out	625	10 (1.6)	<10 [6]
	Convicted Sentence	1,361	13 (1.0)	<10 [6]
	Downgrade in security category	<10 [6]	<10 [6]	<10 [6]
	Internal Cell Move	<10 [6]	<10 [6]	<10 [6]
	Judges Remand	19	<10 [6]	<10 [6]
	Licence Revoke	178	<10 [6]	<10 [6]
	On Remand	1,031	23 (2.2)	<10 [6]
	Transfer	101	<10 [6]	<10 [6]
	Upgrade in security category	<10 [6]	<10 [6]	<10 [6]
**Age - years**				
2017/18	10 - <20	468	<10 [6]	<10 [6]
	20 - <30	6,994	37 (0.5)	10 (27.0)
	30 - <40	7,051	94 (1.3)	32 (34.0)
	40 - <50	4,114	60 (1.5)	22 (36.7)
	50 - <60	2,107	35 (1.7)	19 (54.3)
	60 - <70	684	<10 [6]	<10 [6]
	70 - <80	213	<10 [6]	<10 [6]
	80 - <90	40	<10 [6]	<10 [6]
	90 - <100	<10 [6]	<10 [6]	<10 [6]
	100 - <110	<10 [6]	<10 [6]	<10 [6]
2018/19	10 - <20	436	<10 [6]	<10 [6]
	20 - <30	7,163	68 (0.9)	<10 [6]
	30 - <40	7,381	124 (1.7)	17 (13.7)
	40 - <50	4,180	85 (2.0)	12 (14.1)
	50 - <60	1,978	31 (1.6)	<10 [6]
	60 - <70	701	<10 [6]	<10 [6]
	70 - <80	209	<10 [6]	<10 [6]
	80 - <90	45	<10 [6]	<10 [6]
	90 - <100	<10 [6]	<10 [6]	<10 [6]
	100 - <110	<10 [6]	<10 [6]	<10 [6]
2019/20	10 - <20	404	<10 [6]	<10 [6]
	20 - <30	8,064	92 (1.1)	<10 [6]
	30 - <40	9,125	188 (2.1)	<10 [6]
	40 - <50	4,948	130 (2.6)	<10 [6]
	50 - <60	2,224	35 (1.6)	<10 [6]
	60 - <70	751	<10 [6]	<10 [6]
	70 - <80	238	<10 [6]	<10 [6]
	80 - <90	53	<10 [6]	<10 [6]
	90 - <100	<10 [6]	<10 [6]	<10 [6]
	100 - <110	<10 [6]	<10 [6]	<10 [6]

[1]Eligible for indicator; [2]Achieve in current prison; [3]Achieve in previous prison; [4]Overall achievement - either current or previous prison; [5]Declined indicator; [6]Suppressed (<10) to avoid disclosure

Indicator	*The proportion of people who have received three or more prescriptions for an antipsychotic in the preceding 12 months, and who have a record of blood testing (fasting glucose, HbA1c, lipid profile, urea and electrolytes, full blood count, liver function tests), body mass index and blood pressure*<br>**Group: Mental health**		*Community achievement 2019/20 (%): No direct comparison in QOF - just percentage of patients with MH diagnosis of schizophrenia, bipolar affective disorder and other psychoses who have record of BP and BMI in the last 12 months (80.28% and 74.40% respectively)*
	**Variable**	**Population**	**Eligible[1] (% population)**	**Achieved[4] 4 of the 6 blood tests (% eligible)**
**Length of Stay (months)**				
2017/18	<1	4,474	19 (0.4)	<10 [6]
	1-<6	8,075	65 (0.8)	16 (24.6)
	6-<12	3,672	51 (1.4)	18 (35.3)
	12-<24	2,832	48 (1.7)	16 (33.3)
	24+	2,624	61 (2.3)	37 (60.7)
2018/19	<1	4,801	49 (1.0)	<10 [6]
	1-<6	7,742	106 (1.4)	<10 [6]
	6-<12	3,616	54 (1.5)	<10 [6]
	12-<24	3,447	57 (1.7)	10 (17.5)
	24+	2,493	60 (2.4)	19 (31.7)
2019/20	<1	5,745	70 (1.2)	<10 [6]
	1-<6	9,697	177 (1.8)	<10 [6]
	6-<12	5,090	120 (2.4)	<10 [6]
	12-<24	3,244	52 (1.6)	<10 [6]
	24+	2,035	44 (2.2)	11 (25.0)
**Ethnic Group**				
2017/18	White	15,638	192 (1.2)	64 (33.3)
	Mixed	431	<10 [6]	<10 [6]
	Asian or Asian British	813	<10 [6]	<10 [6]
	Black or Black British	404	<10 [6]	<10 [6]
	Chinese and Other	214	<10 [6]	<10 [6]
	Unclassified	372	<10 [6]	<10 [6]
2018/19	White	14,911	266 (1.8)	35 (13.2)
	Mixed	371	<10 [6]	<10 [6]
	Asian or Asian British	726	<10 [6]	<10 [6]
	Black or Black British	364	<10 [6]	<10 [6]
	Chinese and Other	167	<10 [6]	<10 [6]
	Unclassified	409	<10 [6]	<10 [6]
2019/20	White	16,606	364 (2.2)	19 (5.2)
	Mixed	409	<10 [6]	<10 [6]
	Asian or Asian British	755	<10 [6]	<10 [6]
	Black or Black British	451	<10 [6]	<10 [6]
	Chinese and Other	163	<10 [6]	<10 [6]
	Unclassified	387	11 (2.8)	<10 [6]

[1]Eligible for indicator; [2]Achieve in current prison; [3]Achieve in previous prison; [4]Overall achievement - either current or previous prison; [5]Declined indicator; [6]Suppressed (<10) to avoid disclosure

Indicator	*The proportion of the prison population prescribed three or more psychotropic drugs (antipsychotics, sedative anti-depressants, hypnotics and anxiolytics, pregabalin or gabapentin, opioids) at the same time during an eight-week period*								Community achievement 2019/20 (%): No comparable data in QOF 2019/20

Group: Mental health

	Variable	Population	Satisfy 3 or more[2] (% popln)	Elsewhere 3 or more[3] (% popln)	Achieve 3 or more[4] (% popln)	Satisfy 4 or more[2] (% popln)	Elsewhere 4 or more[3] (% popln)	Achieve 4 or more[4] (% popln)
**Year**								
	2017/18	21,677	97 (0.4)	17 (0.1)	114 (0.5)	33 (0.2)	<10 [6]	39 (0.2)
	2018/19	22,099	145 (0.7)	13 (0.1)	158 (0.7)	53 (0.2)	<10 [6]	58 (0.3)
	2019/20	25,811	196 (0.8)	21 (0.1)	216 (0.8)	97 (0.4)	<10 [6]	103 (0.4)
**Prison**								
2017/18	Prison 1	1,323	<10 [6]	<10 [6]	<10 [6]	<10 [6]	<10 [6]	1 (0.1)
	Prison 2	3,261	43 (1.3)	<10 [6]	45 (1.4)	16 (0.5)	<10 [6]	18 (0.6)
	Prison 3	2,623	<10 [6]	<10 [6]	16 (0.6)	<10 [6]	<10 [6]	3 (0.1)
	Prison 4	2,089	<10 [6]	<10 [6]	<10 [6]	<10 [6]	<10 [6]	1 (0.0)
	Prison 5	637	<10 [6]	<10 [6]	<10 [6]	<10 [6]	<10 [6]	0 (0.0)
	Prison 6	1,552	<10 [6]	<10 [6]	<10 [6]	<10 [6]	<10 [6]	0 (0.0)
	Prison 7	635	<10 [6]	<10 [6]	<10 [6]	<10 [6]	<10 [6]	1 (0.2)
	Prison 8	1,085	<10 [6]	<10 [6]	<10 [6]	<10 [6]	<10 [6]	4 (0.4)
	Prison 9	981	<10 [6]	<10 [6]	<10 [6]	<10 [6]	<10 [6]	1 (0.1)
	Prison 10	2,523	<10 [6]	<10 [6]	<10 [6]	<10 [6]	<10 [6]	5 (0.2)
	Prison 11	3,470	<10 [6]	<10 [6]	<10 [6]	<10 [6]	<10 [6]	2 (0.1)
	Prison 12	815	<10 [6]	<10 [6]	<10 [6]	<10 [6]	<10 [6]	0 (0.0)
	Prison 13	683	11 (1.6)	<10 [6]	12 (1.8)	<10 [6]	<10 [6]	3 (0.4)
2018/19	Prison 1	1,333	<10 [6]	<10 [6]	<10 [6]	<10 [6]	<10 [6]	1 (0.1)
	Prison 2	2,705	46 (1.7)	<10 [6]	46 (1.7)	20 (0.7)	<10 [6]	20 (0.7)
	Prison 3	2,522	15 (0.6)	<10 [6]	18 (0.7)	<10 [6]	<10 [6]	3 (0.1)
	Prison 4	2,349	10 (0.4)	<10 [6]	13 (0.6)	<10 [6]	<10 [6]	6 (0.3)
	Prison 5	676	<10 [6]	<10 [6]	<10 [6]	<10 [6]	<10 [6]	0 (0.0)
	Prison 6	1,513	<10 [6]	<10 [6]	<10 [6]	<10 [6]	<10 [6]	0 (0.0)
	Prison 7	654	15 (2.3)	<10 [6]	15 (2.3)	<10 [6]	<10 [6]	4 (0.6)
	Prison 8	1,148	20 (1.7)	<10 [6]	20 (1.7)	12 (1.0)	<10 [6]	12 (1.0)
	Prison 9	996	<10 [6]	<10 [6]	<10 [6]	<10 [6]	<10 [6]	4 (0.4)
	Prison 10	2,717	<10 [6]	<10 [6]	<10 [6]	<10 [6]	<10 [6]	3 (0.1)
	Prison 11	4,020	<10 [6]	<10 [6]	13 (0.3)	<10 [6]	<10 [6]	3 (0.1)
	Prison 12	792	<10 [6]	<10 [6]	<10 [6]	<10 [6]	<10 [6]	0 (0.0)
	Prison 13	674	<10 [6]	<10 [6]	<10 [6]	<10 [6]	<10 [6]	2 (0.3)
2019/20	Prison 1	1,410	<10 [6]	<10 [6]	<10 [6]	<10 [6]	<10 [6]	2 (0.1)
	Prison 2	2,979	41 (1.4)	<10 [6]	43 (1.4)	17 (0.6)	<10 [6]	17 (0.6)
	Prison 3	2,809	<10 [6]	<10 [6]	12 (0.4)	<10 [6]	<10 [6]	1 (0.0)
	Prison 4	2,651	17 (0.6)	<10 [6]	21 (0.8)	<10 [6]	<10 [6]	7 (0.3)
	Prison 5	616	<10 [6]	<10 [6]	<10 [6]	<10 [6]	<10 [6]	0 (0.0)
	Prison 6	1,533	<10 [6]	<10 [6]	<10 [6]	<10 [6]	<10 [6]	1 (0.1)
	Prison 7	860	11 (1.3)	<10 [6]	12 (1.4)	<10 [6]	<10 [6]	3 (0.3)
	Prison 8	1,385	71 (5.1)	<10 [6]	74 (5.3)	52 (3.8)	<10 [6]	53 (3.8)
	Prison 9	1,092	<10 [6]	<10 [6]	<10 [6]	<10 [6]	<10 [6]	1 (0.1)
	Prison 10	3,577	<10 [6]	<10 [6]	<10 [6]	<10 [6]	<10 [6]	6 (0.2)
	Prison 11	5,348	22 (0.4)	<10 [6]	24 (0.4)	12 (0.2)	<10 [6]	12 (0.2)
	Prison 12	805	<10 [6]	<10 [6]	<10 [6]	<10 [6]	<10 [6]	0 (0.0)
	Prison 13	746	<10 [6]	<10 [6]	<10 [6]	<10 [6]	<10 [6]	0 (0.0)
**Prison category**								
2017/18	A	1,664	15 (0.9)	<10 [6]	17 (1.0)	<10 [6]	<10 [6]	<10 [6]
	B	9,254	50 (0.5)	<10 [6]	55 (0.6)	21 (0.2)	<10 [6]	25 (0.3)
	C	6,035	19 (0.3)	<10 [6]	27 (0.4)	<10 [6]	<10 [6]	<10 [6]
	Closed	1,720	10 (0.6)	<10 [6]	12 (0.7)	<10 [6]	<10 [6]	<10 [6]
	D	2,189	<10 [6]	<10 [6]	<10 [6]	<10 [6]	<10 [6]	<10 [6]
	YOI	815	<10 [6]	<10 [6]	<10 [6]	<10 [6]	<10 [6]	<10 [6]
2018/19	A	1,670	10 (0.6)	<10 [6]	12 (0.7)	<10 [6]	<10 [6]	<10 [6]
	B	9,442	65 (0.7)	<10 [6]	68 (0.7)	25 (0.3)	<10 [6]	26 (0.3)
	C	6,204	31 (0.5)	<10 [6]	37 (0.6)	<10 [6]	<10 [6]	10 (0.2)
	Closed	1,802	35 (1.9)	<10 [6]	35 (1.9)	16 (0.9)	<10 [6]	16 (0.9)
	D	2,189	<10 [6]	<10 [6]	<10 [6]	<10 [6]	<10 [6]	<10 [6]
	YOI	792	<10 [6]	<10 [6]	<10 [6]	<10 [6]	<10 [6]	<10 [6]
2019/20	A	1,838	<10 [6]	<10 [6]	<10 [6]	<10 [6]	<10 [6]	<10 [6]
	B	11,904	71 (0.6)	<10 [6]	76 (0.6)	34 (0.3)	<10 [6]	35 (0.3)
	C	6,870	29 (0.4)	11 (0.2)	40 (0.6)	<10 [6]	<10 [6]	10 (0.1)
	Closed	2,245	82 (3.7)	<10 [6]	86 (3.8)	55 (2.4)	<10 [6]	56 (2.5)
	D	2,149	<10 [6]	<10 [6]	<10 [6]	<10 [6]	<10 [6]	<10 [6]
	YOI	805	<10 [6]	<10 [6]	<10 [6]	<10 [6]	<10 [6]	<10 [6]
**Gender**								
2017/18	F	1,699	10 (0.6)	<10 [6]	12 (0.7)	<10 [6]	<10 [6]	<10 [6]
	M	19,977	87 (0.4)	15 (0.1)	102 (0.5)	28 (0.1)	<10 [6]	34 (0.2)
2018/19	F	1,802	35 (1.9)	<10 [6]	35 (1.9)	16 (0.9)	<10 [6]	16 (0.9)
	M	20,295	110 (0.5)	13 (0.1)	123 (0.6)	37 (0.2)	<10 [6]	42 (0.2)
2019/20	F	1,376	71 (5.2)	<10 [6]	74 (5.4)	52 (3.8)	<10 [6]	53 (3.9)
	M	23,570	114 (0.5)	16 (0.1)	130 (0.6)	42 (0.2)	<10 [6]	47 (0.2)

[1]Eligible for indicator; [2]Achieve in current prison; [3]Achieve in previous prison; [4]Overall achievement - either current or previous prison; [5]Declined indicator; [6]Suppressed (<10) to avoid disclosure

Indicator	The proportion of the prison population prescribed three or more psychotropic drugs (antipsychotics, sedative anti-depressants, hypnotics and anxiolytics, pregabalin or gabapentin, opioids) at the same time during an eight-week period							Community achievement 2019/20 (%): No comparable data in QOF 2019/20
	Group: Mental health							

	Variable	Population	Satisfy 3 or more[2] (% popln)	Elsewhere 3 or more[3] (% popln)	Achieve 3 or more[4] (% popln)	Satisfy 4 or more[2] (% popln)	Elsewhere 4 or more[3] (% popln)	Achieve 4 or more[4] (% popln)
**Sentence Status**								
2017/18	.	81	<10 [6]	<10 [6]	<10 [6]	<10 [6]	<10 [6]	<10 [6]
	Absconded	<10 [6]	<10 [6]	<10 [6]	<10 [6]	<10 [6]	<10 [6]	<10 [6]
	Active In	15,872	60 (0.4)	15 (0.1)	75 (0.5)	17 (0.1)	<10 [6]	21 (0.1)
	Active Out	1,052	<10 [6]	<10 [6]	<10 [6]	<10 [6]	<10 [6]	<10 [6]
	Convicted Sentence	2,125	12 (0.6)	<10 [6]	14 (0.7)	<10 [6]	<10 [6]	<10 [6]
	Downgrade in security category	<10 [6]	<10 [6]	<10 [6]	<10 [6]	<10 [6]	<10 [6]	<10 [6]
	Internal Cell Move	<10 [6]	<10 [6]	<10 [6]	<10 [6]	<10 [6]	<10 [6]	<10 [6]
	Judges Remand	92	<10 [6]	<10 [6]	<10 [6]	<10 [6]	<10 [6]	<10 [6]
	Licence Revoke	52	<10 [6]	<10 [6]	<10 [6]	<10 [6]	<10 [6]	<10 [6]
	On Remand	1,492	21 (1.4)	<10 [6]	21 (1.4)	<10 [6]	<10 [6]	<10 [6]
	Transfer	910	<10 [6]	<10 [6]	<10 [6]	<10 [6]	<10 [6]	<10 [6]
	Upgrade in security category	<10 [6]	<10 [6]	<10 [6]	<10 [6]	<10 [6]	<10 [6]	<10 [6]
2018/19	.	88	<10 [6]	<10 [6]	<10 [6]	<10 [6]	<10 [6]	<10 [6]
	Absconded	<10 [6]	<10 [6]	<10 [6]	<10 [6]	<10 [6]	<10 [6]	<10 [6]
	Active In	18,145	103 (0.6)	13 (0.1)	116 (0.6)	35 (0.2)	<10 [6]	40 (0.2)
	Active Out	835	<10 [6]	<10 [6]	<10 [6]	<10 [6]	<10 [6]	<10 [6]
	Convicted Sentence	1,320	25 (1.9)	<10 [6]	25 (1.9)	<10 [6]	<10 [6]	<10 [6]
	Downgrade in security category	<10 [6]	<10 [6]	<10 [6]	<10 [6]	<10 [6]	<10 [6]	<10 [6]
	Internal Cell Move	<10 [6]	<10 [6]	<10 [6]	<10 [6]	<10 [6]	<10 [6]	<10 [6]
	Judges Remand	<10 [6]	<10 [6]	<10 [6]	<10 [6]	<10 [6]	<10 [6]	<10 [6]
	Licence Revoke	125	<10 [6]	<10 [6]	<10 [6]	<10 [6]	<10 [6]	<10 [6]
	On Remand	1,059	12 (1.1)	<10 [6]	12 (1.1)	<10 [6]	<10 [6]	<10 [6]
	Transfer	518	<10 [6]	<10 [6]	<10 [6]	<10 [6]	<10 [6]	<10 [6]
	Upgrade in security category	<10 [6]	<10 [6]	<10 [6]	<10 [6]	<10 [6]	<10 [6]	<10 [6]
2019/20	.	69	<10 [6]	<10 [6]	<10 [6]	<10 [6]	<10 [6]	<10 [6]
	Absconded	<10 [6]	<10 [6]	<10 [6]	<10 [6]	<10 [6]	<10 [6]	<10 [6]
	Active In	22,424	162 (0.7)	20 (0.1)	181 (0.8)	82 (0.4)	<10 [6]	88 (0.4)
	Active Out	625	<10 [6]	<10 [6]	<10 [6]	<10 [6]	<10 [6]	<10 [6]
	Convicted Sentence	1,361	23 (1.7)	<10 [6]	23 (1.7)	<10 [6]	<10 [6]	<10 [6]
	Downgrade in security category	<10 [6]	<10 [6]	<10 [6]	<10 [6]	<10 [6]	<10 [6]	<10 [6]
	Internal Cell Move	<10 [6]	<10 [6]	<10 [6]	<10 [6]	<10 [6]	<10 [6]	<10 [6]
	Judges Remand	19	<10 [6]	<10 [6]	<10 [6]	<10 [6]	<10 [6]	<10 [6]
	Licence Revoke	178	<10 [6]	<10 [6]	<10 [6]	<10 [6]	<10 [6]	<10 [6]
	Transfer	101	<10 [6]	<10 [6]	<10 [6]	<10 [6]	<10 [6]	<10 [6]
	Upgrade in security category	<10 [6]	<10 [6]	<10 [6]	<10 [6]	<10 [6]	<10 [6]	<10 [6]
**Age - years**								
2017/18	10 - <20	468	<10 [6]	<10 [6]	<10 [6]	<10 [6]	<10 [6]	<10 [6]
	20 - <30	6,994	<10 [6]	<10 [6]	10 (0.1)	<10 [6]	<10 [6]	<10 [6]
	30 - <40	7,051	28 (0.4)	<10 [6]	37 (0.5)	<10 [6]	<10 [6]	11 (0.2)
	40 - <50	4,114	33 (0.8)	<10 [6]	38 (0.9)	13 (0.3)	<10 [6]	16 (0.4)
	50 - <60	2,107	20 (0.9)	<10 [6]	22 (1.0)	<10 [6]	<10 [6]	<10 [6]
	60 - <70	684	<10 [6]	<10 [6]	<10 [6]	<10 [6]	<10 [6]	<10 [6]
	70 - <80	213	<10 [6]	<10 [6]	<10 [6]	<10 [6]	<10 [6]	<10 [6]
	80 - <90	40	<10 [6]	<10 [6]	<10 [6]	<10 [6]	<10 [6]	<10 [6]
	90 - <100	<10 [6]	<10 [6]	<10 [6]	<10 [6]	<10 [6]	<10 [6]	<10 [6]
	100 - <110	<10 [6]	<10 [6]	<10 [6]	<10 [6]	<10 [6]	<10 [6]	<10 [6]
2018/19	10 - <20	436	<10 [6]	<10 [6]	<10 [6]	<10 [6]	<10 [6]	<10 [6]
	20 - <30	7,163	25 (0.3)	<10 [6]	29 (0.4)	<10 [6]	<10 [6]	10 (0.1)
	30 - <40	7,381	53 (0.7)	<10 [6]	57 (0.8)	20 (0.3)	<10 [6]	23 (0.3)
	40 - <50	4,180	41 (1.0)	<10 [6]	43 (1.0)	17 (0.4)	<10 [6]	17 (0.4)
	50 - <60	1,978	14 (0.7)	<10 [6]	17 (0.9)	<10 [6]	<10 [6]	<10 [6]
	60 - <70	701	<10 [6]	<10 [6]	<10 [6]	<10 [6]	<10 [6]	<10 [6]
	70 - <80	209	<10 [6]	<10 [6]	<10 [6]	<10 [6]	<10 [6]	<10 [6]
	80 - <90	45	<10 [6]	<10 [6]	<10 [6]	<10 [6]	<10 [6]	<10 [6]
	90 - <100	<10 [6]	<10 [6]	<10 [6]	<10 [6]	<10 [6]	<10 [6]	<10 [6]
	100 - <110	<10 [6]	<10 [6]	<10 [6]	<10 [6]	<10 [6]	<10 [6]	<10 [6]
2019/20	10 - <20	404	<10 [6]	<10 [6]	<10 [6]	<10 [6]	<10 [6]	<10 [6]
	20 - <30	8,064	29 (0.4)	<10 [6]	34 (0.4)	16 (0.2)	<10 [6]	17 (0.2)
	30 - <40	9,125	90 (1.0)	<10 [6]	101 (1.1)	44 (0.5)	<10 [6]	47 (0.5)
	40 - <50	4,948	58 (1.2)	<10 [6]	61 (1.2)	31 (0.6)	<10 [6]	33 (0.7)
	50 - <60	2,224	19 (0.9)	<10 [6]	20 (0.9)	<10 [6]	<10 [6]	<10 [6]
	60 - <70	751	<10 [6]	<10 [6]	<10 [6]	<10 [6]	<10 [6]	<10 [6]
	70 - <80	238	<10 [6]	<10 [6]	<10 [6]	<10 [6]	<10 [6]	<10 [6]
	80 - <90	53	<10 [6]	<10 [6]	<10 [6]	<10 [6]	<10 [6]	<10 [6]
	90 - <100	<10 [6]	<10 [6]	<10 [6]	<10 [6]	<10 [6]	<10 [6]	<10 [6]
	100 - <110	<10 [6]	<10 [6]	<10 [6]	<10 [6]	<10 [6]	<10 [6]	<10 [6]

[1]Eligible for indicator; [2]Achieve in current prison; [3]Achieve in previous prison; [4]Overall achievement - either current or previous prison; [5]Declined indicator; [6]Suppressed (<10) to avoid disclosure

Indicator	*The proportion of the prison population prescribed three or more psychotropic drugs (antipsychotics, sedative anti-depressants, hypnotics and anxiolytics, pregabalin or gabapentin, opioids) at the same time during an eight-week period*								Community achievement 2019/20 (%): No comparable data in QOF 2019/20
	**Group: Mental health**								

	**Variable**	**Population**	**Satisfy 3 or more[2] (% popln)**	**Elsewhere 3 or more[3] (% popln)**	**Achieve 3 or more[4] (% popln)**	**Satisfy 4 or more[2] (% popln)**	**Elsewhere 4 or more[3] (% popln)**	**Achieve 4 or more[4] (% popln)**
**Length of Stay (months)**								
2017/18	<1	4,474	<10 [6]	<10 [6]	11 (0.2)	<10 [6]	<10 [6]	<10 [6]
	1-<6	8,075	37 (0.5)	<10 [6]	45 (0.6)	12 (0.1)	<10 [6]	14 (0.2)
	6-<12	3,672	20 (0.5)	<10 [6]	23 (0.6)	<10 [6]	<10 [6]	<10 [6]
	12-<24	2,832	17 (0.6)	<10 [6]	17 (0.6)	<10 [6]	<10 [6]	<10 [6]
	24+	2,624	17 (0.6)	<10 [6]	18 (0.7)	<10 [6]	<10 [6]	<10 [6]
2018/19	<1	4,801	10 (0.2)	<10 [6]	13 (0.3)	<10 [6]	<10 [6]	<10 [6]
	1-<6	7,742	48 (0.6)	<10 [6]	52 (0.7)	18 (0.2)	<10 [6]	20 (0.3)
	6-<12	3,616	25 (0.7)	<10 [6]	27 (0.7)	11 (0.3)	<10 [6]	12 (0.3)
	12-<24	3,752	48 (1.3)	<10 [6]	52 (1.4)	16 (0.4)	<10 [6]	18 (0.5)
	24+	2,188	14 (0.6)	<10 [6]	14 (0.6)	<10 [6]	<10 [6]	<10 [6]
2019/20	<1	5,745	20 (0.3)	<10 [6]	23 (0.4)	<10 [6]	<10 [6]	10 (0.2)
	1-<6	9,697	61 (0.6)	14 (0.1)	75 (0.8)	31 (0.3)	<10 [6]	35 (0.4)
	6-<12	5,090	62 (1.2)	<10 [6]	64 (1.3)	31 (0.6)	<10 [6]	32 (0.6)
	12-<24	3,244	38 (1.2)	<10 [6]	38 (1.2)	19 (0.6)	<10 [6]	19 (0.6)
	24+	2,035	15 (0.7)	<10 [6]	16 (0.8)	<10 [6]	<10 [6]	<10 [6]
**Ethnic Group**								
2017/18	White	15,638	83 (0.5)	16 (0.1)	99 (0.6)	29 (0.2)	<10 [6]	35 (0.2)
	Mixed	431	<10 [6]	<10 [6]	<10 [6]	<10 [6]	<10 [6]	<10 [6]
	Asian or Asian British	813	<10 [6]	<10 [6]	<10 [6]	<10 [6]	<10 [6]	<10 [6]
	Black or Black British	404	<10 [6]	<10 [6]	<10 [6]	<10 [6]	<10 [6]	<10 [6]
	Chinese and Other	214	<10 [6]	<10 [6]	<10 [6]	<10 [6]	<10 [6]	<10 [6]
	Unclassified	372	<10 [6]	<10 [6]	<10 [6]	<10 [6]	<10 [6]	<10 [6]
2018/19	White	14,911	122 (0.8)	11 (0.1)	133 (0.9)	46 (0.3)	<10 [6]	50 (0.3)
	Mixed	371	<10 [6]	<10 [6]	<10 [6]	<10 [6]	<10 [6]	<10 [6]
	Asian or Asian British	726	<10 [6]	<10 [6]	<10 [6]	<10 [6]	<10 [6]	<10 [6]
	Black or Black British	364	<10 [6]	<10 [6]	<10 [6]	<10 [6]	<10 [6]	<10 [6]
	Chinese and Other	167	<10 [6]	<10 [6]	<10 [6]	<10 [6]	<10 [6]	<10 [6]
	Unclassified	409	<10 [6]	<10 [6]	<10 [6]	<10 [6]	<10 [6]	<10 [6]
2019/20	White	16,606	161 (1.0)	20 (0.1)	180 (1.1)	82 (0.5)	<10 [6]	87 (0.5)
	Mixed	409	<10 [6]	<10 [6]	<10 [6]	<10 [6]	<10 [6]	<10 [6]
	Asian or Asian British	755	<10 [6]	<10 [6]	<10 [6]	<10 [6]	<10 [6]	<10 [6]
	Black or Black British	451	<10 [6]	<10 [6]	<10 [6]	<10 [6]	<10 [6]	<10 [6]
	Chinese and Other	163	<10 [6]	<10 [6]	<10 [6]	<10 [6]	<10 [6]	<10 [6]
	Unclassified	387	<10 [6]	<10 [6]	<10 [6]	<10 [6]	<10 [6]	<10 [6]

[1]Eligible for indicator; [2]Achieve in current prison; [3]Achieve in previous prison; [4]Overall achievement - either current or previous prison; [5]Declined indicator; [6]Suppressed (<10) to avoid disclosure

Indicator	*The proportion of people aged over 55 years in the prison population who have a record of one of the following assessments in the preceding 12 months; Mini Mental State Examination, 6 item Cognitive Impairment Test, General practitioner Assessment of Cognition*						*Community achievement 2019/20: no comparable data in QOF 2019/20*
	**Group: Mental health**						
	**Variable**	**Population**	**Eligible[1] (% popln)**	**Satisfy[2] (% eligible)**	**Elsewhere[3] (% eligible)**	**Achieve[4] (% eligible)**	
**Year**							
	2017/18	21,677	1,746 (8.1)	23 (1.3)	<10 [6]	23 (1.3)	
	2018/19	22,099	1,729 (7.8)	125 (7.2)	<10 [6]	125 (7.2)	
	2019/20	25,811	1,884 (7.3)	370 (19.6)	<10 [6]	370 (19.6)	
**Prison**							
2017/18	Prison 1	1,323	40 (3.0)	<10 [6]	<10 [6]	<10 [6]	
	Prison 2	3,261	241 (7.4)	<10 [6]	<10 [6]	<10 [6]	
	Prison 3	2,623	189 (7.2)	<10 [6]	<10 [6]	<10 [6]	
	Prison 4	2,089	223 (10.7)	<10 [6]	<10 [6]	<10 [6]	
	Prison 5	637	56 (8.8)	<10 [6]	<10 [6]	<10 [6]	
	Prison 6	1,552	130 (8.4)	<10 [6]	<10 [6]	<10 [6]	
	Prison 7	635	40 (6.3)	<10 [6]	<10 [6]	<10 [6]	
	Prison 8	1,085	70 (6.5)	<10 [6]	<10 [6]	<10 [6]	
	Prison 9	981	224 (22.8)	17 (7.6)	<10 [6]	17 (7.6)	
	Prison 10	2,523	178 (7.1)	<10 [6]	<10 [6]	<10 [6]	
	Prison 11	3,470	230 (6.6)	<10 [6]	<10 [6]	<10 [6]	
	Prison 12	815	<10 [6]	<10 [6]	<10 [6]	<10 [6]	
	Prison 13	683	124 (18.2)	<10 [6]	<10 [6]	<10 [6]	
2018/19	Prison 1	1,333	37 (2.8)	<10 [6]	<10 [6]	<10 [6]	
	Prison 2	2,705	170 (6.3)	65 (38.2)	<10 [6]	65 (38.2)	
	Prison 3	2,522	163 (6.5)	<10 [6]	<10 [6]	<10 [6]	
	Prison 4	2,349	240 (10.2)	<10 [6]	<10 [6]	<10 [6]	
	Prison 5	676	62 (9.2)	<10 [6]	<10 [6]	<10 [6]	
	Prison 6	1,513	121 (8.0)	<10 [6]	<10 [6]	<10 [6]	
	Prison 7	654	41 (6.3)	<10 [6]	<10 [6]	<10 [6]	
	Prison 8	1,148	77 (6.7)	<10 [6]	<10 [6]	<10 [6]	
	Prison 9	996	239 (24.0)	<10 [6]	<10 [6]	<10 [6]	
	Prison 10	2,717	189 (7.0)	36 (19.0)	<10 [6]	36 (19.0)	
	Prison 11	4,020	252 (6.3)	<10 [6]	<10 [6]	<10 [6]	
	Prison 12	792	<10 [6]	<10 [6]	<10 [6]	<10 [6]	
	Prison 13	674	138 (20.5)	<10 [6]	<10 [6]	<10 [6]	
2019/20	Prison 1	1,410	46 (3.3)	21 (45.7)	<10 [6]	21 (45.7)	
	Prison 2	2,979	196 (6.6)	126 (64.3)	<10 [6]	126 (64.3)	
	Prison 3	2,809	172 (6.1)	<10 [6]	<10 [6]	<10 [6]	
	Prison 4	2,651	270 (10.2)	<10 [6]	<10 [6]	<10 [6]	
	Prison 5	616	55 (8.9)	<10 [6]	<10 [6]	<10 [6]	
	Prison 6	1,533	104 (6.8)	64 (61.5)	<10 [6]	64 (61.5)	
	Prison 7	860	60 (7.0)	<10 [6]	<10 [6]	<10 [6]	
	Prison 8	1,385	84 (6.1)	26 (31.0)	<10 [6]	26 (31.0)	
	Prison 9	1,092	260 (23.8)	<10 [6]	<10 [6]	<10 [6]	
	Prison 10	3,577	203 (5.7)	118 (58.1)	<10 [6]	118 (58.1)	
	Prison 11	5,348	295 (5.5)	<10 [6]	<10 [6]	<10 [6]	
	Prison 12	805	<10 [6]	<10 [6]	<10 [6]	<10 [6]	
	Prison 13	746	138 (18.5)	<10 [6]	<10 [6]	<10 [6]	
**Prison category**							
2017/18	A	1,664	348 (20.9)	19 (5.5)	<10 [6]	19 (5.5)	
	B	9,254	649 (7.0)	<10 [6]	<10 [6]	<10 [6]	
	C	6,035	452 (7.5)	<10 [6]	<10 [6]	<10 [6]	
	Closed	1,720	110 (6.4)	<10 [6]	<10 [6]	<10 [6]	
	D	2,189	186 (8.5)	<10 [6]	<10 [6]	<10 [6]	
	YOI	815	<10 [6]	<10 [6]	<10 [6]	<10 [6]	
2018/19	A	1,670	377 (22.6)	<10 [6]	<10 [6]	<10 [6]	
	B	9,442	611 (6.5)	103 (16.9)	<10 [6]	103 (16.9)	
	C	6,204	440 (7.1)	<10 [6]	<10 [6]	<10 [6]	
	Closed	1,802	118 (6.5)	<10 [6]	<10 [6]	<10 [6]	
	D	2,189	183 (8.4)	<10 [6]	<10 [6]	<10 [6]	
	YOI	792	<10 [6]	<10 [6]	<10 [6]	<10 [6]	
2019/20	A	1,838	398 (21.7)	<10 [6]	<10 [6]	<10 [6]	
	B	11,904	694 (5.8)	245 (35.3)	<10 [6]	245 (35.3)	
	C	6,870	488 (7.1)	27 (5.5)	<10 [6]	27 (5.5)	
	Closed	2,245	144 (6.4)	27 (18.8)	<10 [6]	27 (18.8)	
	D	2,149	159 (7.4)	65 (40.9)	<10 [6]	65 (40.9)	
	YOI	805	<10 [6]	<10 [6]	<10 [6]	<10 [6]	
**Gender**							
2017/18	F	1,699	109 (6.4)	<10 [6]	<10 [6]	<10 [6]	
	M	19,977	1,637 (8.2)	23 (1.4)	<10 [6]	23 (1.4)	
2018/19	F	1,802	119 (6.6)	<10 [6]	<10 [6]	<10 [6]	
	M	20,295	1,610 (7.9)	125 (7.8)	<10 [6]	125 (7.8)	
2019/20	F	1,376	83 (6.0)	26 (31.3)	<10 [6]	26 (31.3)	
	M	23,570	1,741 (7.4)	343 (19.7)	<10 [6]	343 (19.7)	

[1]Eligible for indicator; [2]Achieve in current prison; [3]Achieve in previous prison; [4]Overall achievement - either current or previous prison; [5]Declined indicator; [6]Suppressed (<10) to avoid disclosure

Indicator	The proportion of people aged over 55 years in the prison population who have a record of one of the following assessments in the preceding 12 months; Mini Mental State Examination, 6 item Cognitive Impairment Test, General practitioner Assessment of Cognition						Community achievement 2019/20: no comparable data in QOF 2019/20
	Group: Mental health						
	Variable	Population	Eligible[1] (% popln)	Satisfy[2] (% eligible)	Elsewhere[3] (% eligible)	Achieve[4] (% eligible)	
**Sentence Status**							
2017/18	.	81	11 (13.6)	<10 [6]	<10 [6]	<10 [6]	
	Absconded	<10 [6]	<10 [6]	<10 [6]	<10 [6]	<10 [6]	
	Active In	15,872	1,316 (8.3)	22 (1.7)	<10 [6]	22 (1.7)	
	Active Out	1,052	82 (7.8)	<10 [6]	<10 [6]	<10 [6]	
	Convicted Sentence	2,125	169 (8.0)	<10 [6]	<10 [6]	<10 [6]	
	Downgrade in security category	<10 [6]	<10 [6]	<10 [6]	<10 [6]	<10 [6]	
	Internal Cell Move	<10 [6]	<10 [6]	<10 [6]	<10 [6]	<10 [6]	
	Judges Remand	92	<10 [6]	<10 [6]	<10 [6]	<10 [6]	
	Licence Revoke	52	<10 [6]	<10 [6]	<10 [6]	<10 [6]	
	On Remand	1,492	86 (5.8)	<10 [6]	<10 [6]	<10 [6]	
	Transfer	910	76 (8.4)	<10 [6]	<10 [6]	<10 [6]	
	Upgrade in security category	<10 [6]	<10 [6]	<10 [6]	<10 [6]	<10 [6]	
2018/19	.	88	<10 [6]	<10 [6]	<10 [6]	<10 [6]	
	Absconded	<10 [6]	<10 [6]	<10 [6]	<10 [6]	<10 [6]	
	Active In	18,145	1,440 (7.9)	74 (5.1)	<10 [6]	74 (5.1)	
	Active Out	835	72 (8.6)	<10 [6]	<10 [6]	<10 [6]	
	Convicted Sentence	1,320	112 (8.5)	30 (26.8)	<10 [6]	30 (26.8)	
	Downgrade in security category	<10 [6]	<10 [6]	<10 [6]	<10 [6]	<10 [6]	
	Internal Cell Move	<10 [6]	<10 [6]	<10 [6]	<10 [6]	<10 [6]	
	Judges Remand	<10 [6]	<10 [6]	<10 [6]	<10 [6]	<10 [6]	
	Licence Revoke	125	<10 [6]	<10 [6]	<10 [6]	<10 [6]	
	On Remand	1,059	53 (5.0)	17 (32.1)	<10 [6]	17 (32.1)	
	Transfer	518	39 (7.5)	<10 [6]	<10 [6]	<10 [6]	
	Upgrade in security category	<10 [6]	<10 [6]	<10 [6]	<10 [6]	<10 [6]	
2019/20	.	69	<10 [6]	<10 [6]	<10 [6]	<10 [6]	
	Absconded	<10 [6]	<10 [6]	<10 [6]	<10 [6]	<10 [6]	
	Active In	22,424	1,636 (7.3)	247 (15.1)	<10 [6]	247 (15.1)	
	Active Out	625	54 (8.6)	18 (33.3)	<10 [6]	18 (33.3)	
	Convicted Sentence	1,361	109 (8.0)	60 (55.0)	<10 [6]	60 (55.0)	
	Downgrade in security category	<10 [6]	<10 [6]	<10 [6]	<10 [6]	<10 [6]	
	Internal Cell Move	<10 [6]	<10 [6]	<10 [6]	<10 [6]	<10 [6]	
	Judges Remand	19	<10 [6]	<10 [6]	<10 [6]	<10 [6]	
	Licence Revoke	178	10 (5.6)	<10 [6]	<10 [6]	<10 [6]	
	On Remand	1,031	59 (5.7)	37 (62.7)	<10 [6]	37 (62.7)	
	Transfer	101	<10 [6]	<10 [6]	<10 [6]	<10 [6]	
	Upgrade in security category	<10 [6]	<10 [6]	<10 [6]	<10 [6]	<10 [6]	
**Age - years**							
2017/18	10 - <20	468	<10 [6]	<10 [6]	<10 [6]	<10 [6]	
	20 - <30	6,994	<10 [6]	<10 [6]	<10 [6]	<10 [6]	
	30 - <40	7,051	<10 [6]	<10 [6]	<10 [6]	<10 [6]	
	40 - <50	4,114	<10 [6]	<10 [6]	<10 [6]	<10 [6]	
	50 - <60	2,107	805 (38.2)	<10 [6]	<10 [6]	<10 [6]	
	60 - <70	684	684 (100.0)	14 (2.0)	<10 [6]	14 (2.0)	
	70 - <80	213	213 (100.0)	<10 [6]	<10 [6]	<10 [6]	
	80 - <90	40	40 (100.0)	<10 [6]	<10 [6]	<10 [6]	
	90 - <100	<10 [6]	<10 [6]	<10 [6]	<10 [6]	<10 [6]	
	100 - <110	<10 [6]	<10 [6]	<10 [6]	<10 [6]	<10 [6]	
2018/19	10 - <20	436	<10 [6]	<10 [6]	<10 [6]	<10 [6]	
	20 - <30	7,163	<10 [6]	<10 [6]	<10 [6]	<10 [6]	
	30 - <40	7,381	<10 [6]	<10 [6]	<10 [6]	<10 [6]	
	40 - <50	4,180	<10 [6]	<10 [6]	<10 [6]	<10 [6]	
	50 - <60	1,978	771 (39.0)	44 (5.7)	<10 [6]	44 (5.7)	
	60 - <70	701	701 (100.0)	55 (7.8)	<10 [6]	55 (7.8)	
	70 - <80	209	209 (100.0)	19 (9.1)	<10 [6]	19 (9.1)	
	80 - <90	45	45 (100.0)	<10 [6]	<10 [6]	<10 [6]	
	90 - <100	<10 [6]	<10 [6]	<10 [6]	<10 [6]	<10 [6]	
	100 - <110	<10 [6]	<10 [6]	<10 [6]	<10 [6]	<10 [6]	
2019/20	10 - <20	404	<10 [6]	<10 [6]	<10 [6]	<10 [6]	
	20 - <30	8,064	<10 [6]	<10 [6]	<10 [6]	<10 [6]	
	30 - <40	9,125	<10 [6]	<10 [6]	<10 [6]	<10 [6]	
	40 - <50	4,948	<10 [6]	<10 [6]	<10 [6]	<10 [6]	
	50 - <60	2,224	840 (37.8)	192 (22.9)	<10 [6]	192 (22.9)	
	60 - <70	751	751 (100.0)	126 (16.8)	<10 [6]	126 (16.8)	
	70 - <80	238	238 (100.0)	41 (17.2)	<10 [6]	41 (17.2)	
	80 - <90	53	53 (100.0)	10 (18.9)	<10 [6]	10 (18.9)	
	90 - <100	<10 [6]	<10 [6]	<10 [6]	<10 [6]	<10 [6]	
	100 - <110	<10 [6]	<10 [6]	<10 [6]	<10 [6]	<10 [6]	

[1]Eligible for indicator; [2]Achieve in current prison; [3]Achieve in previous prison; [4]Overall achievement - either current or previous prison; [5]Declined indicator; [6]Suppressed (<10) to avoid disclosure

Indicator ***The proportion of people aged over 55 years in the prison population who have a record of one of the following assessments in the preceding 12 months; Mini Mental State Examination, 6 item Cognitive Impairment Test, General practitioner Assessment of Cognition***

Community achievement 2019/20: no comparable data in QOF 2019/20

Group: Mental health

	Variable	Population	Eligible[1] (% popln)	Satisfy[2] (% eligible)	Elsewhere[3] (% eligible)	Achieve[4] (% eligible)
**Length of Stay (months)**						
2017/18	<1	4,474	267 (6.0)	<10 [6]	<10 [6]	<10 [6]
	1-<6	8,075	424 (5.3)	<10 [6]	<10 [6]	<10 [6]
	6-<12	3,672	242 (6.6)	<10 [6]	<10 [6]	<10 [6]
	12-<24	2,832	228 (8.1)	<10 [6]	<10 [6]	<10 [6]
	24+	2,624	585 (22.3)	19 (3.2)	<10 [6]	19 (3.2)
2018/19	<1	4,801	273 (5.7)	17 (6.2)	<10 [6]	17 (6.2)
	1-<6	7,742	365 (4.7)	34 (9.3)	<10 [6]	34 (9.3)
	6-<12	3,616	217 (6.0)	17 (7.8)	<10 [6]	17 (7.8)
	12-<24	3,447	301 (8.7)	37 (12.3)	<10 [6]	37 (12.3)
	24+	2,493	573 (23.0)	20 (3.5)	<10 [6]	20 (3.5)
2019/20	<1	5,745	333 (5.8)	69 (20.7)	<10 [6]	69 (20.7)
	1-<6	9,697	482 (5.0)	151 (31.3)	<10 [6]	151 (31.3)
	6-<12	5,090	273 (5.4)	79 (28.9)	<10 [6]	79 (28.9)
	12-<24	3,244	293 (9.0)	52 (17.7)	<10 [6]	52 (17.7)
	24+	2,035	503 (24.7)	19 (3.8)	<10 [6]	19 (3.8)
Ethnic Group						
2017/18	White	15,638	1,407 (9.0)	21 (1.5)	<10 [6]	21 (1.5)
	Mixed	431	21 (4.9)	<10 [6]	<10 [6]	<10 [6]
	Asian or Asian British	813	27 (3.3)	<10 [6]	<10 [6]	<10 [6]
	Black or Black British	404	26 (6.4)	<10 [6]	<10 [6]	<10 [6]
	Chinese and Other	214	11 (5.1)	<10 [6]	<10 [6]	<10 [6]
	Unclassified	372	33 (8.9)	<10 [6]	<10 [6]	<10 [6]
2018/19	White	14,911	1,312 (8.8)	114 (8.7)	<10 [6]	114 (8.7)
	Mixed	371	24 (6.5)	<10 [6]	<10 [6]	<10 [6]
	Asian or Asian British	726	23 (3.2)	<10 [6]	<10 [6]	<10 [6]
	Black or Black British	364	27 (7.4)	<10 [6]	<10 [6]	<10 [6]
	Chinese and Other	167	<10 [6]	<10 [6]	<10 [6]	<10 [6]
	Unclassified	409	24 (5.9)	<10 [6]	<10 [6]	<10 [6]
2019/20	White	16,606	1,309 (7.9)	287 (21.9)	<10 [6]	287 (21.9)
	Mixed	409	24 (5.9)	<10 [6]	<10 [6]	<10 [6]
	Asian or Asian British	755	21 (2.8)	<10 [6]	<10 [6]	<10 [6]
	Black or Black British	451	29 (6.4)	<10 [6]	<10 [6]	<10 [6]
	Chinese and Other	163	<10 [6]	<10 [6]	<10 [6]	<10 [6]
	Unclassified	387	24 (6.2)	<10 [6]	<10 [6]	<10 [6]

[1]Eligible for indicator; [2]Achieve in current prison; [3]Achieve in previous prison; [4]Overall achievement - either current or previous prison; [5]Declined indicator; [6]Suppressed (<10) to avoid disclosure

Indicator	***The proportion of people with a new diagnosis of any form of dementia in the reporting year, and who have a record of the following blood tests between 12 months before and 6 months after the date of diagnosis: full blood count, calcium, glucose and/or HbA1C, urea and electrolytes, liver function, thyroid function, serum vitamin B12, folate levels***	*no comparable data in QOF 2019/20. Most recent QOF data was from 2018/19 and achievement was 70.01%*
	**Group: Mental health**	

	Variable	Population	Eligible[1] (% popln)	FBC (% eligible)		Calcium (% eligible)		Hb1Ac (% eligible)		Renal (% eligible)	
				Satisfy[2]	Elsewhere[3]	Satisfy[2]	Elsewhere[3]	Satisfy[2]	Elsewhere[3]	Satisfy[2]	Elsewhere[3]
**Year**											
	2017/18	21,677	10 (0.0)	<10 [6]	<10 [6]	<10 [6]	<10 [6]	<10 [6]	<10 [6]	<10 [6]	<10 [6]
	2018/19	22,099	10 (0.0)	<10 [6]	<10 [6]	<10 [6]	<10 [6]	<10 [6]	<10 [6]	<10 [6]	<10 [6]
	2019/20	25,811	13 (0.1)	<10 [6]	<10 [6]	<10 [6]	<10 [6]	<10 [6]	<10 [6]	<10 [6]	<10 [6]
**Prison**											
2017/18	Prison 1	1,323	<10 [6]	<10 [6]	<10 [6]	<10 [6]	<10 [6]	<10 [6]	<10 [6]	<10 [6]	<10 [6]
	Prison 2	3,261	<10 [6]	<10 [6]	<10 [6]	<10 [6]	<10 [6]	<10 [6]	<10 [6]	<10 [6]	<10 [6]
	Prison 3	2,623	<10 [6]	<10 [6]	<10 [6]	<10 [6]	<10 [6]	<10 [6]	<10 [6]	<10 [6]	<10 [6]
	Prison 4	2,089	<10 [6]	<10 [6]	<10 [6]	<10 [6]	<10 [6]	<10 [6]	<10 [6]	<10 [6]	<10 [6]
	Prison 5	637	<10 [6]	<10 [6]	<10 [6]	<10 [6]	<10 [6]	<10 [6]	<10 [6]	<10 [6]	<10 [6]
	Prison 6	1,552	<10 [6]	<10 [6]	<10 [6]	<10 [6]	<10 [6]	<10 [6]	<10 [6]	<10 [6]	<10 [6]
	Prison 7	635	<10 [6]	<10 [6]	<10 [6]	<10 [6]	<10 [6]	<10 [6]	<10 [6]	<10 [6]	<10 [6]
	Prison 8	1,085	<10 [6]	<10 [6]	<10 [6]	<10 [6]	<10 [6]	<10 [6]	<10 [6]	<10 [6]	<10 [6]
	Prison 9	981	<10 [6]	<10 [6]	<10 [6]	<10 [6]	<10 [6]	<10 [6]	<10 [6]	<10 [6]	<10 [6]
	Prison 10	2,523	<10 [6]	<10 [6]	<10 [6]	<10 [6]	<10 [6]	<10 [6]	<10 [6]	<10 [6]	<10 [6]
	Prison 11	3,470	<10 [6]	<10 [6]	<10 [6]	<10 [6]	<10 [6]	<10 [6]	<10 [6]	<10 [6]	<10 [6]
	Prison 12	815	<10 [6]	<10 [6]	<10 [6]	<10 [6]	<10 [6]	<10 [6]	<10 [6]	<10 [6]	<10 [6]
	Prison 13	683	<10 [6]	<10 [6]	<10 [6]	<10 [6]	<10 [6]	<10 [6]	<10 [6]	<10 [6]	<10 [6]
2018/19	Prison 1	1,333	<10 [6]	<10 [6]	<10 [6]	<10 [6]	<10 [6]	<10 [6]	<10 [6]	<10 [6]	<10 [6]
	Prison 2	2,705	<10 [6]	<10 [6]	<10 [6]	<10 [6]	<10 [6]	<10 [6]	<10 [6]	<10 [6]	<10 [6]
	Prison 3	2,522	<10 [6]	<10 [6]	<10 [6]	<10 [6]	<10 [6]	<10 [6]	<10 [6]	<10 [6]	<10 [6]
	Prison 4	2,349	<10 [6]	<10 [6]	<10 [6]	<10 [6]	<10 [6]	<10 [6]	<10 [6]	<10 [6]	<10 [6]
	Prison 5	676	<10 [6]	<10 [6]	<10 [6]	<10 [6]	<10 [6]	<10 [6]	<10 [6]	<10 [6]	<10 [6]
	Prison 6	1,513	<10 [6]	<10 [6]	<10 [6]	<10 [6]	<10 [6]	<10 [6]	<10 [6]	<10 [6]	<10 [6]
	Prison 7	654	<10 [6]	<10 [6]	<10 [6]	<10 [6]	<10 [6]	<10 [6]	<10 [6]	<10 [6]	<10 [6]
	Prison 8	1,148	<10 [6]	<10 [6]	<10 [6]	<10 [6]	<10 [6]	<10 [6]	<10 [6]	<10 [6]	<10 [6]
	Prison 9	996	<10 [6]	<10 [6]	<10 [6]	<10 [6]	<10 [6]	<10 [6]	<10 [6]	<10 [6]	<10 [6]
	Prison 10	2,717	<10 [6]	<10 [6]	<10 [6]	<10 [6]	<10 [6]	<10 [6]	<10 [6]	<10 [6]	<10 [6]
	Prison 11	4,020	<10 [6]	<10 [6]	<10 [6]	<10 [6]	<10 [6]	<10 [6]	<10 [6]	<10 [6]	<10 [6]
	Prison 12	792	<10 [6]	<10 [6]	<10 [6]	<10 [6]	<10 [6]	<10 [6]	<10 [6]	<10 [6]	<10 [6]
	Prison 13	674	<10 [6]	<10 [6]	<10 [6]	<10 [6]	<10 [6]	<10 [6]	<10 [6]	<10 [6]	<10 [6]
2019/20	Prison 1	1,410	<10 [6]	<10 [6]	<10 [6]	<10 [6]	<10 [6]	<10 [6]	<10 [6]	<10 [6]	<10 [6]
	Prison 2	2,979	<10 [6]	<10 [6]	<10 [6]	<10 [6]	<10 [6]	<10 [6]	<10 [6]	<10 [6]	<10 [6]
	Prison 3	2,809	<10 [6]	<10 [6]	<10 [6]	<10 [6]	<10 [6]	<10 [6]	<10 [6]	<10 [6]	<10 [6]
	Prison 4	2,651	<10 [6]	<10 [6]	<10 [6]	<10 [6]	<10 [6]	<10 [6]	<10 [6]	<10 [6]	<10 [6]
	Prison 5	616	<10 [6]	<10 [6]	<10 [6]	<10 [6]	<10 [6]	<10 [6]	<10 [6]	<10 [6]	<10 [6]
	Prison 6	1,533	<10 [6]	<10 [6]	<10 [6]	<10 [6]	<10 [6]	<10 [6]	<10 [6]	<10 [6]	<10 [6]
	Prison 7	860	<10 [6]	<10 [6]	<10 [6]	<10 [6]	<10 [6]	<10 [6]	<10 [6]	<10 [6]	<10 [6]
	Prison 8	1,385	<10 [6]	<10 [6]	<10 [6]	<10 [6]	<10 [6]	<10 [6]	<10 [6]	<10 [6]	<10 [6]
	Prison 9	1,092	<10 [6]	<10 [6]	<10 [6]	<10 [6]	<10 [6]	<10 [6]	<10 [6]	<10 [6]	<10 [6]
	Prison 10	3,577	<10 [6]	<10 [6]	<10 [6]	<10 [6]	<10 [6]	<10 [6]	<10 [6]	<10 [6]	<10 [6]
	Prison 11	5,348	<10 [6]	<10 [6]	<10 [6]	<10 [6]	<10 [6]	<10 [6]	<10 [6]	<10 [6]	<10 [6]
	Prison 12	805	<10 [6]	<10 [6]	<10 [6]	<10 [6]	<10 [6]	<10 [6]	<10 [6]	<10 [6]	<10 [6]
	Prison 13	746	<10 [6]	<10 [6]	<10 [6]	<10 [6]	<10 [6]	<10 [6]	<10 [6]	<10 [6]	<10 [6]
**Prison category**											
2017/18	A	1,664	<10 [6]	<10 [6]	<10 [6]	<10 [6]	<10 [6]	<10 [6]	<10 [6]	<10 [6]	<10 [6]
	B	9,254	<10 [6]	<10 [6]	<10 [6]	<10 [6]	<10 [6]	<10 [6]	<10 [6]	<10 [6]	<10 [6]
	C	6,035	<10 [6]	<10 [6]	<10 [6]	<10 [6]	<10 [6]	<10 [6]	<10 [6]	<10 [6]	<10 [6]
	Closed	1,720	<10 [6]	<10 [6]	<10 [6]	<10 [6]	<10 [6]	<10 [6]	<10 [6]	<10 [6]	<10 [6]
	D	2,189	<10 [6]	<10 [6]	<10 [6]	<10 [6]	<10 [6]	<10 [6]	<10 [6]	<10 [6]	<10 [6]
	YOI	815	<10 [6]	<10 [6]	<10 [6]	<10 [6]	<10 [6]	<10 [6]	<10 [6]	<10 [6]	<10 [6]
2018/19	A	1,670	<10 [6]	<10 [6]	<10 [6]	<10 [6]	<10 [6]	<10 [6]	<10 [6]	<10 [6]	<10 [6]
	B	9,442	<10 [6]	<10 [6]	<10 [6]	<10 [6]	<10 [6]	<10 [6]	<10 [6]	<10 [6]	<10 [6]
	C	6,204	<10 [6]	<10 [6]	<10 [6]	<10 [6]	<10 [6]	<10 [6]	<10 [6]	<10 [6]	<10 [6]
	Closed	1,802	<10 [6]	<10 [6]	<10 [6]	<10 [6]	<10 [6]	<10 [6]	<10 [6]	<10 [6]	<10 [6]
	D	2,189	<10 [6]	<10 [6]	<10 [6]	<10 [6]	<10 [6]	<10 [6]	<10 [6]	<10 [6]	<10 [6]
	YOI	792	<10 [6]	<10 [6]	<10 [6]	<10 [6]	<10 [6]	<10 [6]	<10 [6]	<10 [6]	<10 [6]

[1]Eligible for indicator; [2]Achieve in current prison; [3]Achieve in previous prison; [4]Overall achievement - either current or previous prison; [5]Declined indicator; [6]Suppressed (<10) to avoid disclosure

Indicator	*The proportion of people with a new diagnosis of any form of dementia in the reporting year, and who have a record of the following blood tests between 12 months before and 6 months after the date of diagnosis: full blood count, calcium, glucose and/or HbA1C, urea and electrolytes, liver function, thyroid function, serum vitamin B12, folate levels*								*no comparable data in QOF 2019/20. Most recent QOF data was from 2018/19 and achievement was 70.01%*
	**Group: Mental health**								
		**Eligible[1]**	**Thyorid (% eligible)**		**Serum (% eligible)**		**Folate (% eligible)**		
	**Variable**	**(% popln)**	**Satisfy[2]**	**Elsewhere[3]**	**Satisfy[2]**	**Elsewhere[3]**	**Satisfy[2]**	**Elsewhere[3]**	
Year									
	2017/18	10	<10 [6]	<10 [6]	<10 [6]	<10 [6]	<10 [6]	<10 [6]	
	2018/19	10	<10 [6]	<10 [6]	<10 [6]	<10 [6]	<10 [6]	<10 [6]	
	2019/20	13	<10 [6]	<10 [6]	<10 [6]	<10 [6]	<10 [6]	<10 [6]	
**Prison**									
2017/18	Prison 1	<10 [6]	<10 [6]	<10 [6]	<10 [6]	0 (0.0)	<10 [6]	<10 [6]	
	Prison 2	<10 [6]	<10 [6]	<10 [6]	<10 [6]	<10 [6]	<10 [6]	<10 [6]	
	Prison 3	<10 [6]	<10 [6]	<10 [6]	<10 [6]	<10 [6]	<10 [6]	<10 [6]	
	Prison 4	<10 [6]	<10 [6]	<10 [6]	<10 [6]	<10 [6]	<10 [6]	<10 [6]	
	Prison 5	<10 [6]	<10 [6]	<10 [6]	<10 [6]	<10 [6]	<10 [6]	<10 [6]	
	Prison 6	<10 [6]	<10 [6]	<10 [6]	<10 [6]	<10 [6]	<10 [6]	<10 [6]	
	Prison 7	<10 [6]	<10 [6]	<10 [6]	<10 [6]	<10 [6]	<10 [6]	<10 [6]	
	Prison 8	<10 [6]	<10 [6]	<10 [6]	<10 [6]	<10 [6]	<10 [6]	<10 [6]	
	Prison 9	<10 [6]	<10 [6]	<10 [6]	<10 [6]	<10 [6]	<10 [6]	<10 [6]	
	Prison 10	<10 [6]	<10 [6]	<10 [6]	<10 [6]	<10 [6]	<10 [6]	<10 [6]	
	Prison 11	<10 [6]	<10 [6]	<10 [6]	<10 [6]	<10 [6]	<10 [6]	<10 [6]	
	Prison 12	<10 [6]	<10 [6]	<10 [6]	<10 [6]	<10 [6]	<10 [6]	<10 [6]	
	Prison 13	<10 [6]	<10 [6]	<10 [6]	<10 [6]	<10 [6]	<10 [6]	<10 [6]	
2018/19	Prison 1	<10 [6]	<10 [6]	<10 [6]	<10 [6]	<10 [6]	<10 [6]	<10 [6]	
	Prison 2	<10 [6]	<10 [6]	<10 [6]	<10 [6]	<10 [6]	<10 [6]	<10 [6]	
	Prison 3	<10 [6]	<10 [6]	<10 [6]	<10 [6]	<10 [6]	<10 [6]	<10 [6]	
	Prison 4	<10 [6]	<10 [6]	<10 [6]	<10 [6]	<10 [6]	<10 [6]	<10 [6]	
	Prison 5	<10 [6]	<10 [6]	<10 [6]	<10 [6]	<10 [6]	<10 [6]	<10 [6]	
	Prison 6	<10 [6]	<10 [6]	<10 [6]	<10 [6]	<10 [6]	<10 [6]	<10 [6]	
	Prison 7	<10 [6]	<10 [6]	<10 [6]	<10 [6]	<10 [6]	<10 [6]	<10 [6]	
	Prison 8	<10 [6]	<10 [6]	<10 [6]	<10 [6]	<10 [6]	<10 [6]	<10 [6]	
	Prison 9	<10 [6]	<10 [6]	<10 [6]	<10 [6]	<10 [6]	<10 [6]	<10 [6]	
	Prison 10	<10 [6]	<10 [6]	<10 [6]	<10 [6]	<10 [6]	<10 [6]	<10 [6]	
	Prison 11	<10 [6]	<10 [6]	<10 [6]	<10 [6]	<10 [6]	<10 [6]	<10 [6]	
	Prison 12	<10 [6]	<10 [6]	<10 [6]	<10 [6]	<10 [6]	<10 [6]	<10 [6]	
	Prison 13	<10 [6]	<10 [6]	<10 [6]	<10 [6]	<10 [6]	<10 [6]	<10 [6]	
2019/20	Prison 1	<10 [6]	<10 [6]	<10 [6]	<10 [6]	<10 [6]	<10 [6]	<10 [6]	
	Prison 2	<10 [6]	<10 [6]	<10 [6]	<10 [6]	<10 [6]	<10 [6]	<10 [6]	
	Prison 3	<10 [6]	<10 [6]	<10 [6]	<10 [6]	<10 [6]	<10 [6]	<10 [6]	
	Prison 4	<10 [6]	<10 [6]	<10 [6]	<10 [6]	<10 [6]	<10 [6]	<10 [6]	
	Prison 5	<10 [6]	<10 [6]	<10 [6]	<10 [6]	<10 [6]	<10 [6]	<10 [6]	
	Prison 6	<10 [6]	<10 [6]	<10 [6]	<10 [6]	<10 [6]	<10 [6]	<10 [6]	
	Prison 7	<10 [6]	<10 [6]	<10 [6]	<10 [6]	<10 [6]	<10 [6]	<10 [6]	
	Prison 8	<10 [6]	<10 [6]	<10 [6]	<10 [6]	<10 [6]	<10 [6]	<10 [6]	
	Prison 9	<10 [6]	<10 [6]	<10 [6]	<10 [6]	<10 [6]	<10 [6]	<10 [6]	
	Prison 10	<10 [6]	<10 [6]	<10 [6]	<10 [6]	<10 [6]	<10 [6]	<10 [6]	
	Prison 11	<10 [6]	<10 [6]	<10 [6]	<10 [6]	<10 [6]	<10 [6]	<10 [6]	
	Prison 12	<10 [6]	<10 [6]	<10 [6]	<10 [6]	<10 [6]	<10 [6]	<10 [6]	
	Prison 13	<10 [6]	<10 [6]	<10 [6]	<10 [6]	<10 [6]	<10 [6]	<10 [6]	
Prison category									
2017/18	A	<10 [6]	<10 [6]	<10 [6]	<10 [6]	<10 [6]	<10 [6]	<10 [6]	
	B	<10 [6]	<10 [6]	<10 [6]	<10 [6]	<10 [6]	<10 [6]	<10 [6]	
	C	<10 [6]	<10 [6]	<10 [6]	<10 [6]	<10 [6]	<10 [6]	<10 [6]	
	Closed	<10 [6]	<10 [6]	<10 [6]	<10 [6]	<10 [6]	<10 [6]	<10 [6]	
	D	<10 [6]	<10 [6]	<10 [6]	<10 [6]	<10 [6]	<10 [6]	<10 [6]	
	YOI	<10 [6]	<10 [6]	<10 [6]	<10 [6]	<10 [6]	<10 [6]	<10 [6]	
2018/19	A	<10 [6]	<10 [6]	<10 [6]	<10 [6]	<10 [6]	<10 [6]	<10 [6]	
	B	<10 [6]	<10 [6]	<10 [6]	<10 [6]	<10 [6]	<10 [6]	<10 [6]	
	C	<10 [6]	<10 [6]	<10 [6]	<10 [6]	<10 [6]	<10 [6]	<10 [6]	
	Closed	<10 [6]	<10 [6]	<10 [6]	<10 [6]	<10 [6]	<10 [6]	<10 [6]	
	D	<10 [6]	<10 [6]	<10 [6]	<10 [6]	<10 [6]	<10 [6]	<10 [6]	
	YOI	<10 [6]	<10 [6]	<10 [6]	<10 [6]	<10 [6]	<10 [6]	<10 [6]	

[1]Eligible for indicator; [2]Achieve in current prison; [3]Achieve in previous prison; [4]Overall achievement - either current or previous prison; [5]Declined indicator; [6]Suppressed (<10) to avoid disclosure

Indicator

The proportion of people with a new diagnosis of any form of dementia in the reporting year, and who have a record of the following blood tests between 12 months before and 6 months after the date of diagnosis: full blood count, calcium, glucose and/or HbA1C, urea and electrolytes, liver function, thyroid function, serum vitamin B12, folate levels

no comparable data in QOF 2019/20. Most recent QOF data was from 2018/19 and achievement was 70.01%

Group: Mental health

	Variable	Population	Eligible[1] (% popln)	FBC (% eligible) Satisfy[2]	FBC (% eligible) Elsewhere[3]	Calcium (% eligible) Satisfy[2]	Calcium (% eligible) Elsewhere[3]	Hb1Ac (% eligible) Satisfy[2]	Hb1Ac (% eligible) Elsewhere[3]	Renal (% eligible) Satisfy[2]	Renal (% eligible) Elsewhere[3]
2019/20	A	1,838	<10 [6]	<10 [6]	<10 [6]	<10 [6]	<10 [6]	<10 [6]	<10 [6]	<10 [6]	<10 [6]
	B	11,904	<10 [6]	<10 [6]	<10 [6]	<10 [6]	<10 [6]	<10 [6]	<10 [6]	<10 [6]	<10 [6]
	C	6,870	<10 [6]	<10 [6]	<10 [6]	<10 [6]	<10 [6]	<10 [6]	<10 [6]	<10 [6]	<10 [6]
	Closed	2,245	<10 [6]	<10 [6]	<10 [6]	<10 [6]	<10 [6]	<10 [6]	<10 [6]	<10 [6]	<10 [6]
	D	2,149	<10 [6]	<10 [6]	<10 [6]	<10 [6]	<10 [6]	<10 [6]	<10 [6]	<10 [6]	<10 [6]
	YOI	805	<10 [6]	<10 [6]	<10 [6]	<10 [6]	<10 [6]	<10 [6]	<10 [6]	<10 [6]	<10 [6]
**Gender**											
2017/18	F	1,699	<10 [6]	<10 [6]	<10 [6]	<10 [6]	<10 [6]	<10 [6]	<10 [6]	<10 [6]	<10 [6]
	M	19,977	10 (0.1)	<10 [6]	<10 [6]	<10 [6]	<10 [6]	<10 [6]	<10 [6]	<10 [6]	<10 [6]
2018/19	F	1,802	<10 [6]	<10 [6]	<10 [6]	<10 [6]	<10 [6]	<10 [6]	<10 [6]	<10 [6]	<10 [6]
	M	20,295	10 (0.0)	<10 [6]	<10 [6]	<10 [6]	<10 [6]	<10 [6]	<10 [6]	<10 [6]	<10 [6]
2019/20	F	1,376	<10 [6]	<10 [6]	<10 [6]	<10 [6]	<10 [6]	<10 [6]	<10 [6]	<10 [6]	<10 [6]
	M	23,570	12 (0.1)	<10 [6]	<10 [6]	<10 [6]	<10 [6]	<10 [6]	<10 [6]	<10 [6]	<10 [6]
**Sentence Status**											
2017/18	.	81	<10 [6]	<10 [6]	<10 [6]	<10 [6]	<10 [6]	<10 [6]	<10 [6]	<10 [6]	<10 [6]
	Absconded	<10 [6]	<10 [6]	<10 [6]	<10 [6]	<10 [6]	<10 [6]	<10 [6]	<10 [6]	<10 [6]	<10 [6]
	Active In	15,872	<10 [6]	<10 [6]	<10 [6]	<10 [6]	<10 [6]	<10 [6]	<10 [6]	<10 [6]	<10 [6]
	Active Out	1,052	<10 [6]	<10 [6]	<10 [6]	<10 [6]	<10 [6]	<10 [6]	<10 [6]	<10 [6]	<10 [6]
	Convicted Sentence	2,125	<10 [6]	<10 [6]	<10 [6]	<10 [6]	<10 [6]	<10 [6]	<10 [6]	<10 [6]	<10 [6]
	Downgrade in security category	<10 [6]	<10 [6]	<10 [6]	<10 [6]	<10 [6]	<10 [6]	<10 [6]	<10 [6]	<10 [6]	<10 [6]
	Internal Cell Move	<10 [6]	<10 [6]	<10 [6]	<10 [6]	<10 [6]	<10 [6]	<10 [6]	<10 [6]	<10 [6]	<10 [6]
	Judges Remand	92	<10 [6]	<10 [6]	<10 [6]	<10 [6]	<10 [6]	<10 [6]	<10 [6]	<10 [6]	<10 [6]
	Licence Revoke	52	<10 [6]	<10 [6]	<10 [6]	<10 [6]	<10 [6]	<10 [6]	<10 [6]	<10 [6]	<10 [6]
	On Remand	1,492	<10 [6]	<10 [6]	<10 [6]	<10 [6]	<10 [6]	<10 [6]	<10 [6]	<10 [6]	<10 [6]
	Transfer	910	<10 [6]	<10 [6]	<10 [6]	<10 [6]	<10 [6]	<10 [6]	<10 [6]	<10 [6]	<10 [6]
	Upgrade in security category	<10 [6]	<10 [6]	<10 [6]	<10 [6]	<10 [6]	<10 [6]	<10 [6]	<10 [6]	<10 [6]	<10 [6]
2018/19	.	88	<10 [6]	<10 [6]	<10 [6]	<10 [6]	<10 [6]	<10 [6]	<10 [6]	<10 [6]	<10 [6]
	Absconded	<10 [6]	<10 [6]	<10 [6]	<10 [6]	<10 [6]	<10 [6]	<10 [6]	<10 [6]	<10 [6]	<10 [6]
	Active In	18,145	<10 [6]	<10 [6]	<10 [6]	<10 [6]	<10 [6]	<10 [6]	<10 [6]	<10 [6]	<10 [6]
	Active Out	835	<10 [6]	<10 [6]	<10 [6]	<10 [6]	<10 [6]	<10 [6]	<10 [6]	<10 [6]	<10 [6]
	Convicted Sentence	1,320	<10 [6]	<10 [6]	<10 [6]	<10 [6]	<10 [6]	<10 [6]	<10 [6]	<10 [6]	<10 [6]
	Downgrade in security category	<10 [6]	<10 [6]	<10 [6]	<10 [6]	<10 [6]	<10 [6]	<10 [6]	<10 [6]	<10 [6]	<10 [6]
	Internal Cell Move	<10 [6]	<10 [6]	<10 [6]	<10 [6]	<10 [6]	<10 [6]	<10 [6]	<10 [6]	<10 [6]	<10 [6]
	Judges Remand	<10 [6]	<10 [6]	<10 [6]	<10 [6]	<10 [6]	<10 [6]	<10 [6]	<10 [6]	<10 [6]	<10 [6]
	Licence Revoke	125	<10 [6]	<10 [6]	<10 [6]	<10 [6]	<10 [6]	<10 [6]	<10 [6]	<10 [6]	<10 [6]
	On Remand	1,059	<10 [6]	<10 [6]	<10 [6]	<10 [6]	<10 [6]	<10 [6]	<10 [6]	<10 [6]	<10 [6]
	Transfer	518	<10 [6]	<10 [6]	<10 [6]	<10 [6]	<10 [6]	<10 [6]	<10 [6]	<10 [6]	<10 [6]
	Upgrade in security category	<10 [6]	<10 [6]	<10 [6]	<10 [6]	<10 [6]	<10 [6]	<10 [6]	<10 [6]	<10 [6]	<10 [6]
2019/20	.	69	<10 [6]	<10 [6]	<10 [6]	<10 [6]	<10 [6]	<10 [6]	<10 [6]	<10 [6]	<10 [6]
	Absconded	<10 [6]	<10 [6]	<10 [6]	<10 [6]	<10 [6]	<10 [6]	<10 [6]	<10 [6]	<10 [6]	<10 [6]
	Active In	22,424	10 (0.0)	<10 [6]	<10 [6]	<10 [6]	<10 [6]	<10 [6]	<10 [6]	<10 [6]	<10 [6]
	Active Out	625	<10 [6]	<10 [6]	<10 [6]	<10 [6]	<10 [6]	<10 [6]	<10 [6]	<10 [6]	<10 [6]
	Convicted Sentence	1,361	<10 [6]	<10 [6]	<10 [6]	<10 [6]	<10 [6]	<10 [6]	<10 [6]	<10 [6]	<10 [6]
	Downgrade in security category	<10 [6]	<10 [6]	<10 [6]	<10 [6]	<10 [6]	<10 [6]	<10 [6]	<10 [6]	<10 [6]	<10 [6]
	Internal Cell Move	<10 [6]	<10 [6]	<10 [6]	<10 [6]	<10 [6]	<10 [6]	<10 [6]	<10 [6]	<10 [6]	<10 [6]
	Judges Remand	19	<10 [6]	<10 [6]	<10 [6]	<10 [6]	<10 [6]	<10 [6]	<10 [6]	<10 [6]	<10 [6]
	Licence Revoke	178	<10 [6]	<10 [6]	<10 [6]	<10 [6]	<10 [6]	<10 [6]	<10 [6]	<10 [6]	<10 [6]
	On Remand	1,031	<10 [6]	<10 [6]	<10 [6]	<10 [6]	<10 [6]	<10 [6]	<10 [6]	<10 [6]	<10 [6]
	Transfer	101	<10 [6]	<10 [6]	<10 [6]	<10 [6]	<10 [6]	<10 [6]	<10 [6]	<10 [6]	<10 [6]
	Upgrade in security category	<10 [6]	<10 [6]	<10 [6]	<10 [6]	<10 [6]	<10 [6]	<10 [6]	<10 [6]	<10 [6]	<10 [6]
**Age - years**											
2017/18	10 - <20	468	<10 [6]	<10 [6]	<10 [6]	<10 [6]	<10 [6]	<10 [6]	<10 [6]	<10 [6]	<10 [6]
	20 - <30	6,994	<10 [6]	<10 [6]	<10 [6]	<10 [6]	<10 [6]	<10 [6]	<10 [6]	<10 [6]	<10 [6]
	30 - <40	7,051	<10 [6]	<10 [6]	<10 [6]	<10 [6]	<10 [6]	<10 [6]	<10 [6]	<10 [6]	<10 [6]
	40 - <50	4,114	<10 [6]	<10 [6]	<10 [6]	<10 [6]	<10 [6]	<10 [6]	<10 [6]	<10 [6]	<10 [6]
	50 - <60	2,107	<10 [6]	<10 [6]	<10 [6]	<10 [6]	<10 [6]	<10 [6]	<10 [6]	<10 [6]	<10 [6]
	60 - <70	684	<10 [6]	<10 [6]	<10 [6]	<10 [6]	<10 [6]	<10 [6]	<10 [6]	<10 [6]	<10 [6]
	70 - <80	213	<10 [6]	<10 [6]	<10 [6]	<10 [6]	<10 [6]	<10 [6]	<10 [6]	<10 [6]	<10 [6]
	80 - <90	40	<10 [6]	<10 [6]	<10 [6]	<10 [6]	<10 [6]	<10 [6]	<10 [6]	<10 [6]	<10 [6]
	90 - <100	<10 [6]	<10 [6]	<10 [6]	<10 [6]	<10 [6]	<10 [6]	<10 [6]	<10 [6]	<10 [6]	<10 [6]
	100 - <110	<10 [6]	<10 [6]	<10 [6]	<10 [6]	<10 [6]	<10 [6]	<10 [6]	<10 [6]	<10 [6]	<10 [6]

[1]Eligible for indicator; [2]Achieve in current prison; [3]Achieve in previous prison; [4]Overall achievement - either current or previous prison; [5]Declined indicator; [6]Suppressed (<10) to avoid disclosure

Indicator *The proportion of people with a new diagnosis of any form of dementia in the reporting year, and who have a record of the following blood tests between 12 months before and 6 months after the date of diagnosis: full blood count, calcium, glucose and/or HbA1C, urea and electrolytes, liver function, thyroid function, serum vitamin B12, folate levels*

Group: Mental health

no comparable data in QOF 2019/20. Most recent QOF data was from 2018/19 and achievement was 70.01%

	Variable	Eligible[1] (% popln)	Thyorid (% eligible) Satisfy[2]	Thyorid (% eligible) Elsewhere[3]	Serum (% eligible) Satisfy[2]	Serum (% eligible) Elsewhere[3]	Folate (% eligible) Satisfy[2]	Folate (% eligible) Elsewhere[3]
2019/20	A	<10 [6]	<10 [6]	<10 [6]	<10 [6]	<10 [6]	<10 [6]	<10 [6]
	B	<10 [6]	<10 [6]	<10 [6]	<10 [6]	<10 [6]	<10 [6]	<10 [6]
	C	<10 [6]	<10 [6]	<10 [6]	<10 [6]	<10 [6]	<10 [6]	<10 [6]
	Closed	<10 [6]	<10 [6]	<10 [6]	<10 [6]	<10 [6]	<10 [6]	<10 [6]
	D	<10 [6]	<10 [6]	<10 [6]	<10 [6]	<10 [6]	<10 [6]	<10 [6]
	YOI	<10 [6]	<10 [6]	<10 [6]	<10 [6]	<10 [6]	<10 [6]	<10 [6]
**Gender**								
2017/18	F	<10 [6]	<10 [6]	<10 [6]	<10 [6]	<10 [6]	<10 [6]	<10 [6]
	M	10	<10 [6]	<10 [6]	<10 [6]	<10 [6]	<10 [6]	<10 [6]
2018/19	F	<10 [6]	<10 [6]	<10 [6]	<10 [6]	<10 [6]	<10 [6]	<10 [6]
	M	10	<10 [6]	<10 [6]	<10 [6]	<10 [6]	<10 [6]	<10 [6]
2019/20	F	<10 [6]	<10 [6]	<10 [6]	<10 [6]	<10 [6]	<10 [6]	<10 [6]
	M	12	<10 [6]	<10 [6]	<10 [6]	<10 [6]	<10 [6]	<10 [6]
**Sentence Status**								
2017/18	.	<10 [6]	<10 [6]	<10 [6]	<10 [6]	<10 [6]	<10 [6]	<10 [6]
	Absconded	<10 [6]	<10 [6]	<10 [6]	<10 [6]	<10 [6]	<10 [6]	<10 [6]
	Active In	<10 [6]	<10 [6]	<10 [6]	<10 [6]	<10 [6]	<10 [6]	<10 [6]
	Active Out	<10 [6]	<10 [6]	<10 [6]	<10 [6]	<10 [6]	<10 [6]	<10 [6]
	Convicted Sentence	<10 [6]	<10 [6]	<10 [6]	<10 [6]	<10 [6]	<10 [6]	<10 [6]
	Downgrade in security category	<10 [6]	<10 [6]	<10 [6]	<10 [6]	<10 [6]	<10 [6]	<10 [6]
	Internal Cell Move	<10 [6]	<10 [6]	<10 [6]	<10 [6]	<10 [6]	<10 [6]	<10 [6]
	Judges Remand	<10 [6]	<10 [6]	<10 [6]	<10 [6]	<10 [6]	<10 [6]	<10 [6]
	Licence Revoke	<10 [6]	<10 [6]	<10 [6]	<10 [6]	<10 [6]	<10 [6]	<10 [6]
	On Remand	<10 [6]	<10 [6]	<10 [6]	<10 [6]	<10 [6]	<10 [6]	<10 [6]
	Transfer	<10 [6]	<10 [6]	<10 [6]	<10 [6]	<10 [6]	<10 [6]	<10 [6]
	Upgrade in security category	<10 [6]	<10 [6]	<10 [6]	<10 [6]	<10 [6]	<10 [6]	<10 [6]
2018/19	.	<10 [6]	<10 [6]	<10 [6]	<10 [6]	<10 [6]	<10 [6]	<10 [6]
	Absconded	<10 [6]	<10 [6]	<10 [6]	<10 [6]	<10 [6]	<10 [6]	<10 [6]
	Active In	<10 [6]	<10 [6]	<10 [6]	<10 [6]	<10 [6]	<10 [6]	<10 [6]
	Active Out	<10 [6]	<10 [6]	<10 [6]	<10 [6]	<10 [6]	<10 [6]	<10 [6]
	Convicted Sentence	<10 [6]	<10 [6]	<10 [6]	<10 [6]	<10 [6]	<10 [6]	<10 [6]
	Downgrade in security category	<10 [6]	<10 [6]	<10 [6]	<10 [6]	<10 [6]	<10 [6]	<10 [6]
	Internal Cell Move	<10 [6]	<10 [6]	<10 [6]	<10 [6]	<10 [6]	<10 [6]	<10 [6]
	Judges Remand	<10 [6]	<10 [6]	<10 [6]	<10 [6]	<10 [6]	<10 [6]	<10 [6]
	Licence Revoke	<10 [6]	<10 [6]	<10 [6]	<10 [6]	<10 [6]	<10 [6]	<10 [6]
	On Remand	<10 [6]	<10 [6]	<10 [6]	<10 [6]	<10 [6]	<10 [6]	<10 [6]
	Transfer	<10 [6]	<10 [6]	<10 [6]	<10 [6]	<10 [6]	<10 [6]	<10 [6]
	Upgrade in security category	<10 [6]	<10 [6]	<10 [6]	<10 [6]	<10 [6]	<10 [6]	<10 [6]
2019/20	.	<10 [6]	<10 [6]	<10 [6]	<10 [6]	<10 [6]	<10 [6]	<10 [6]
	Absconded	<10 [6]	<10 [6]	<10 [6]	<10 [6]	<10 [6]	<10 [6]	<10 [6]
	Active In	<10 [6]	<10 [6]	<10 [6]	<10 [6]	<10 [6]	<10 [6]	<10 [6]
	Active Out	<10 [6]	<10 [6]	<10 [6]	<10 [6]	<10 [6]	<10 [6]	<10 [6]
	Convicted Sentence	<10 [6]	<10 [6]	<10 [6]	<10 [6]	<10 [6]	<10 [6]	<10 [6]
	Downgrade in security category	<10 [6]	<10 [6]	<10 [6]	<10 [6]	<10 [6]	<10 [6]	<10 [6]
	Internal Cell Move	<10 [6]	<10 [6]	<10 [6]	<10 [6]	<10 [6]	<10 [6]	<10 [6]
	Judges Remand	<10 [6]	<10 [6]	<10 [6]	<10 [6]	<10 [6]	<10 [6]	<10 [6]
	Licence Revoke	<10 [6]	<10 [6]	<10 [6]	<10 [6]	<10 [6]	<10 [6]	<10 [6]
	On Remand	<10 [6]	<10 [6]	<10 [6]	<10 [6]	<10 [6]	<10 [6]	<10 [6]
	Transfer	<10 [6]	<10 [6]	<10 [6]	<10 [6]	<10 [6]	<10 [6]	<10 [6]
	Upgrade in security category	<10 [6]	<10 [6]	<10 [6]	<10 [6]	<10 [6]	<10 [6]	<10 [6]
**Age - years**								
2017/18	10 - <20	<10 [6]	<10 [6]	<10 [6]	<10 [6]	<10 [6]	<10 [6]	<10 [6]
	20 - <30	<10 [6]	<10 [6]	<10 [6]	<10 [6]	<10 [6]	<10 [6]	<10 [6]
	30 - <40	<10 [6]	<10 [6]	<10 [6]	<10 [6]	<10 [6]	<10 [6]	<10 [6]
	40 - <50	<10 [6]	<10 [6]	<10 [6]	<10 [6]	<10 [6]	<10 [6]	<10 [6]
	50 - <60	<10 [6]	<10 [6]	<10 [6]	<10 [6]	<10 [6]	<10 [6]	<10 [6]
	60 - <70	<10 [6]	<10 [6]	<10 [6]	<10 [6]	<10 [6]	<10 [6]	<10 [6]
	70 - <80	<10 [6]	<10 [6]	<10 [6]	<10 [6]	<10 [6]	<10 [6]	<10 [6]
	80 - <90	<10 [6]	<10 [6]	<10 [6]	<10 [6]	<10 [6]	<10 [6]	<10 [6]
	90 - <100	<10 [6]	<10 [6]	<10 [6]	<10 [6]	<10 [6]	<10 [6]	<10 [6]
	100 - <110	<10 [6]	<10 [6]	<10 [6]	<10 [6]	<10 [6]	<10 [6]	<10 [6]

[1]Eligible for indicator; [2]Achieve in current prison; [3]Achieve in previous prison; [4]Overall achievement - either current or previous prison; [5]Declined indicator; [6]Suppressed (<10) to avoid disclosure

Indicator ***The proportion of people with a new diagnosis of any form of dementia in the reporting year, and who have a record of the following blood tests between 12 months before and 6 months after the date of diagnosis: full blood count, calcium, glucose and/or HbA1C, urea and electrolytes, liver function, thyroid function, serum vitamin B12, folate levels***

no comparable data in QOF 2019/20. Most recent QOF data was from 2018/19 and achievement was 70.01%

Group: Mental health

				FBC (% eligible)		Calcium (% eligible)		Hb1Ac (% eligible)		Renal (% eligible)	
	**Variable**	**Population**	**Eligible[1] (% popln)**	**Satisfy[2]**	**Elsewhere[3]**	**Satisfy[2]**	**Elsewhere[3]**	**Satisfy[2]**	**Elsewhere[3]**	**Satisfy[2]**	**Elsewhere[3]**
2018/19	10 - <20	436	<10 [6]	<10 [6]	<10 [6]	<10 [6]	<10 [6]	<10 [6]	<10 [6]	<10 [6]	<10 [6]
	20 - <30	7,163	<10 [6]	<10 [6]	<10 [6]	<10 [6]	<10 [6]	<10 [6]	<10 [6]	<10 [6]	<10 [6]
	30 - <40	7,381	<10 [6]	<10 [6]	<10 [6]	<10 [6]	<10 [6]	<10 [6]	<10 [6]	<10 [6]	<10 [6]
	40 - <50	4,180	<10 [6]	<10 [6]	<10 [6]	<10 [6]	<10 [6]	<10 [6]	<10 [6]	<10 [6]	<10 [6]
	50 - <60	1,978	<10 [6]	<10 [6]	<10 [6]	<10 [6]	<10 [6]	<10 [6]	<10 [6]	<10 [6]	<10 [6]
	60 - <70	701	<10 [6]	<10 [6]	<10 [6]	<10 [6]	<10 [6]	<10 [6]	<10 [6]	<10 [6]	<10 [6]
	70 - <80	209	<10 [6]	<10 [6]	<10 [6]	<10 [6]	<10 [6]	<10 [6]	<10 [6]	<10 [6]	<10 [6]
	80 - <90	45	<10 [6]	<10 [6]	<10 [6]	<10 [6]	<10 [6]	<10 [6]	<10 [6]	<10 [6]	<10 [6]
	90 - <100	<10 [6]	<10 [6]	<10 [6]	<10 [6]	<10 [6]	<10 [6]	<10 [6]	<10 [6]	<10 [6]	<10 [6]
	100 - <110	<10 [6]	<10 [6]	<10 [6]	<10 [6]	<10 [6]	<10 [6]	<10 [6]	<10 [6]	<10 [6]	<10 [6]
2019/20	10 - <20	404	<10 [6]	<10 [6]	<10 [6]	<10 [6]	<10 [6]	<10 [6]	<10 [6]	<10 [6]	<10 [6]
	20 - <30	8,064	<10 [6]	<10 [6]	<10 [6]	<10 [6]	<10 [6]	<10 [6]	<10 [6]	<10 [6]	<10 [6]
	30 - <40	9,125	<10 [6]	<10 [6]	<10 [6]	<10 [6]	<10 [6]	<10 [6]	<10 [6]	<10 [6]	<10 [6]
	40 - <50	4,948	<10 [6]	<10 [6]	<10 [6]	<10 [6]	<10 [6]	<10 [6]	<10 [6]	<10 [6]	<10 [6]
	50 - <60	2,224	<10 [6]	<10 [6]	<10 [6]	<10 [6]	<10 [6]	<10 [6]	<10 [6]	<10 [6]	<10 [6]
	60 - <70	751	<10 [6]	<10 [6]	<10 [6]	<10 [6]	<10 [6]	<10 [6]	<10 [6]	<10 [6]	<10 [6]
	70 - <80	238	<10 [6]	<10 [6]	<10 [6]	<10 [6]	<10 [6]	<10 [6]	<10 [6]	<10 [6]	<10 [6]
	80 - <90	53	<10 [6]	<10 [6]	<10 [6]	<10 [6]	<10 [6]	<10 [6]	<10 [6]	<10 [6]	<10 [6]
	90 - <100	<10 [6]	<10 [6]	<10 [6]	<10 [6]	<10 [6]	<10 [6]	<10 [6]	<10 [6]	<10 [6]	<10 [6]
	100 - <110	<10 [6]	<10 [6]	<10 [6]	<10 [6]	<10 [6]	<10 [6]	<10 [6]	<10 [6]	<10 [6]	<10 [6]
**Length of Stay (months)**											
2017/18	<1	4,474	<10 [6]	<10 [6]	<10 [6]	<10 [6]	<10 [6]	<10 [6]	<10 [6]	<10 [6]	<10 [6]
	1-<6	8,075	<10 [6]	<10 [6]	<10 [6]	<10 [6]	<10 [6]	<10 [6]	<10 [6]	<10 [6]	<10 [6]
	6-<12	3,672	<10 [6]	<10 [6]	<10 [6]	<10 [6]	<10 [6]	<10 [6]	<10 [6]	<10 [6]	<10 [6]
	12-<24	2,832	<10 [6]	<10 [6]	<10 [6]	<10 [6]	<10 [6]	<10 [6]	<10 [6]	<10 [6]	<10 [6]
	24+	2,624	<10 [6]	<10 [6]	<10 [6]	<10 [6]	<10 [6]	<10 [6]	<10 [6]	<10 [6]	<10 [6]
2018/19	<1	4,801	<10 [6]	<10 [6]	<10 [6]	<10 [6]	<10 [6]	<10 [6]	<10 [6]	<10 [6]	<10 [6]
	1-<6	7,742	<10 [6]	<10 [6]	<10 [6]	<10 [6]	<10 [6]	<10 [6]	<10 [6]	<10 [6]	<10 [6]
	6-<12	3,616	<10 [6]	<10 [6]	<10 [6]	<10 [6]	<10 [6]	<10 [6]	<10 [6]	<10 [6]	<10 [6]
	12-<24	3,447	<10 [6]	<10 [6]	<10 [6]	<10 [6]	<10 [6]	<10 [6]	<10 [6]	<10 [6]	<10 [6]
	24+	2,493	<10 [6]	<10 [6]	<10 [6]	<10 [6]	<10 [6]	<10 [6]	<10 [6]	<10 [6]	<10 [6]
2019/20	<1	5,745	<10 [6]	<10 [6]	<10 [6]	<10 [6]	<10 [6]	<10 [6]	<10 [6]	<10 [6]	<10 [6]
	1-<6	9,697	<10 [6]	<10 [6]	<10 [6]	<10 [6]	<10 [6]	<10 [6]	<10 [6]	<10 [6]	<10 [6]
	6-<12	5,090	<10 [6]	<10 [6]	<10 [6]	<10 [6]	<10 [6]	<10 [6]	<10 [6]	<10 [6]	<10 [6]
	12-<24	3,244	<10 [6]	<10 [6]	<10 [6]	<10 [6]	<10 [6]	<10 [6]	<10 [6]	<10 [6]	<10 [6]
	24+	2,035	<10 [6]	<10 [6]	<10 [6]	<10 [6]	<10 [6]	<10 [6]	<10 [6]	<10 [6]	<10 [6]
**Ethnic Group**											
2017/18	White	15,638	<10 [6]	<10 [6]	<10 [6]	<10 [6]	<10 [6]	<10 [6]	<10 [6]	<10 [6]	<10 [6]
	Mixed	431	<10 [6]	<10 [6]	<10 [6]	<10 [6]	<10 [6]	<10 [6]	<10 [6]	<10 [6]	<10 [6]
	Asian or Asian British	813	<10 [6]	<10 [6]	<10 [6]	<10 [6]	<10 [6]	<10 [6]	<10 [6]	<10 [6]	<10 [6]
	Black or Black British	404	<10 [6]	<10 [6]	<10 [6]	<10 [6]	<10 [6]	<10 [6]	<10 [6]	<10 [6]	<10 [6]
	Chinese and Other	214	<10 [6]	<10 [6]	<10 [6]	<10 [6]	<10 [6]	<10 [6]	<10 [6]	<10 [6]	<10 [6]
	Unclassified	372	<10 [6]	<10 [6]	<10 [6]	<10 [6]	<10 [6]	<10 [6]	<10 [6]	<10 [6]	<10 [6]
2018/19	White	14,911	<10 [6]	<10 [6]	<10 [6]	<10 [6]	<10 [6]	<10 [6]	<10 [6]	<10 [6]	<10 [6]
	Mixed	371	<10 [6]	<10 [6]	<10 [6]	<10 [6]	<10 [6]	<10 [6]	<10 [6]	<10 [6]	<10 [6]
	Asian or Asian British	726	<10 [6]	<10 [6]	<10 [6]	<10 [6]	<10 [6]	<10 [6]	<10 [6]	<10 [6]	<10 [6]
	Black or Black British	364	<10 [6]	<10 [6]	<10 [6]	<10 [6]	<10 [6]	<10 [6]	<10 [6]	<10 [6]	<10 [6]
	Chinese and Other	167	<10 [6]	<10 [6]	<10 [6]	<10 [6]	<10 [6]	<10 [6]	<10 [6]	<10 [6]	<10 [6]
	Unclassified	409	<10 [6]	<10 [6]	<10 [6]	<10 [6]	<10 [6]	<10 [6]	<10 [6]	<10 [6]	<10 [6]
2019/20	White	16,606	<10 [6]	<10 [6]	<10 [6]	<10 [6]	<10 [6]	<10 [6]	<10 [6]	<10 [6]	<10 [6]
	Mixed	409	<10 [6]	<10 [6]	<10 [6]	<10 [6]	<10 [6]	<10 [6]	<10 [6]	<10 [6]	<10 [6]
	Asian or Asian British	755	<10 [6]	<10 [6]	<10 [6]	<10 [6]	<10 [6]	<10 [6]	<10 [6]	<10 [6]	<10 [6]
	Black or Black British	451	<10 [6]	<10 [6]	<10 [6]	<10 [6]	<10 [6]	<10 [6]	<10 [6]	<10 [6]	<10 [6]
	Chinese and Other	163	<10 [6]	<10 [6]	<10 [6]	<10 [6]	<10 [6]	<10 [6]	<10 [6]	<10 [6]	<10 [6]
	Unclassified	387	<10 [6]	<10 [6]	<10 [6]	<10 [6]	<10 [6]	<10 [6]	<10 [6]	<10 [6]	<10 [6]

[1]Eligible for indicator; [2]Achieve in current prison; [3]Achieve in previous prison; [4]Overall achievement - either current or previous prison; [5]Declined indicator; [6]Suppressed (<10) to avoid disclosure

Indicator

The proportion of people with a new diagnosis of any form of dementia in the reporting year, and who have a record of the following blood tests between 12 months before and 6 months after the date of diagnosis: full blood count, calcium, glucose and/or HbA1C, urea and electrolytes, liver function, thyroid function, serum vitamin B12, folate levels

no comparable data in QOF 2019/20. Most recent QOF data was from 2018/19 and achievement was 70.01%

Group: Mental health

	**Variable**	**Eligible[1] (% popln)**	**Thyorid (% eligible) Satisfy[2]**	**Thyorid (% eligible) Elsewhere[3]**	**Serum (% eligible) Satisfy[2]**	**Serum (% eligible) Elsewhere[3]**	**Folate (% eligible) Satisfy[2]**	**Folate (% eligible) Elsewhere[3]**
2018/19	10 - <20	<10 [6]	<10 [6]	<10 [6]	<10 [6]	<10 [6]	<10 [6]	<10 [6]
	20 - <30	<10 [6]	<10 [6]	<10 [6]	<10 [6]	<10 [6]	<10 [6]	<10 [6]
	30 - <40	<10 [6]	<10 [6]	<10 [6]	<10 [6]	<10 [6]	<10 [6]	<10 [6]
	40 - <50	<10 [6]	<10 [6]	<10 [6]	<10 [6]	<10 [6]	<10 [6]	<10 [6]
	50 - <60	<10 [6]	<10 [6]	<10 [6]	<10 [6]	<10 [6]	<10 [6]	<10 [6]
	60 - <70	<10 [6]	<10 [6]	<10 [6]	<10 [6]	<10 [6]	<10 [6]	<10 [6]
	70 - <80	<10 [6]	<10 [6]	<10 [6]	<10 [6]	<10 [6]	<10 [6]	<10 [6]
	80 - <90	<10 [6]	<10 [6]	<10 [6]	<10 [6]	<10 [6]	<10 [6]	<10 [6]
	90 - <100	<10 [6]	<10 [6]	<10 [6]	<10 [6]	<10 [6]	<10 [6]	<10 [6]
	100 - <110	<10 [6]	<10 [6]	<10 [6]	<10 [6]	<10 [6]	<10 [6]	<10 [6]
2019/20	10 - <20	<10 [6]	<10 [6]	<10 [6]	<10 [6]	<10 [6]	<10 [6]	<10 [6]
	20 - <30	<10 [6]	<10 [6]	<10 [6]	<10 [6]	<10 [6]	<10 [6]	<10 [6]
	30 - <40	<10 [6]	<10 [6]	<10 [6]	<10 [6]	<10 [6]	<10 [6]	<10 [6]
	40 - <50	<10 [6]	<10 [6]	<10 [6]	<10 [6]	<10 [6]	<10 [6]	<10 [6]
	50 - <60	<10 [6]	<10 [6]	<10 [6]	<10 [6]	<10 [6]	<10 [6]	<10 [6]
	60 - <70	<10 [6]	<10 [6]	<10 [6]	<10 [6]	<10 [6]	<10 [6]	<10 [6]
	70 - <80	<10 [6]	<10 [6]	<10 [6]	<10 [6]	<10 [6]	<10 [6]	<10 [6]
	80 - <90	<10 [6]	<10 [6]	<10 [6]	<10 [6]	<10 [6]	<10 [6]	<10 [6]
	90 - <100	<10 [6]	<10 [6]	<10 [6]	<10 [6]	<10 [6]	<10 [6]	<10 [6]
	100 - <110	<10 [6]	<10 [6]	<10 [6]	<10 [6]	<10 [6]	<10 [6]	<10 [6]
**Length of Stay (months)**								
2017/18	<1	<10 [6]	<10 [6]	<10 [6]	<10 [6]	<10 [6]	<10 [6]	<10 [6]
	1-<6	<10 [6]	<10 [6]	<10 [6]	<10 [6]	<10 [6]	<10 [6]	<10 [6]
	6-<12	<10 [6]	<10 [6]	<10 [6]	<10 [6]	<10 [6]	<10 [6]	<10 [6]
	12-<24	<10 [6]	<10 [6]	<10 [6]	<10 [6]	<10 [6]	<10 [6]	<10 [6]
	24+	<10 [6]	<10 [6]	<10 [6]	<10 [6]	<10 [6]	<10 [6]	<10 [6]
2018/19	<1	<10 [6]	<10 [6]	<10 [6]	<10 [6]	<10 [6]	<10 [6]	<10 [6]
	1-<6	<10 [6]	<10 [6]	<10 [6]	<10 [6]	<10 [6]	<10 [6]	<10 [6]
	6-<12	<10 [6]	<10 [6]	<10 [6]	<10 [6]	<10 [6]	<10 [6]	<10 [6]
	12-<24	<10 [6]	<10 [6]	<10 [6]	<10 [6]	<10 [6]	<10 [6]	<10 [6]
	24+	<10 [6]	<10 [6]	<10 [6]	<10 [6]	<10 [6]	<10 [6]	<10 [6]
2019/20	<1	<10 [6]	<10 [6]	<10 [6]	<10 [6]	<10 [6]	<10 [6]	<10 [6]
	1-<6	<10 [6]	<10 [6]	<10 [6]	<10 [6]	<10 [6]	<10 [6]	<10 [6]
	6-<12	<10 [6]	<10 [6]	<10 [6]	<10 [6]	<10 [6]	<10 [6]	<10 [6]
	12-<24	<10 [6]	<10 [6]	<10 [6]	<10 [6]	<10 [6]	<10 [6]	<10 [6]
	24+	<10 [6]	<10 [6]	<10 [6]	<10 [6]	<10 [6]	<10 [6]	<10 [6]
**Ethnic Group**								
2017/18	White	<10 [6]	<10 [6]	<10 [6]	<10 [6]	<10 [6]	<10 [6]	<10 [6]
	Mixed	<10 [6]	<10 [6]	<10 [6]	<10 [6]	<10 [6]	<10 [6]	<10 [6]
	Asian or Asian British	<10 [6]	<10 [6]	<10 [6]	<10 [6]	<10 [6]	<10 [6]	<10 [6]
	Black or Black British	<10 [6]	<10 [6]	<10 [6]	<10 [6]	<10 [6]	<10 [6]	<10 [6]
	Chinese and Other	<10 [6]	<10 [6]	<10 [6]	<10 [6]	<10 [6]	<10 [6]	<10 [6]
	Unclassified	<10 [6]	<10 [6]	<10 [6]	<10 [6]	<10 [6]	<10 [6]	<10 [6]
2018/19	White	<10 [6]	<10 [6]	<10 [6]	<10 [6]	<10 [6]	<10 [6]	<10 [6]
	Mixed	<10 [6]	<10 [6]	<10 [6]	<10 [6]	<10 [6]	<10 [6]	<10 [6]
	Asian or Asian British	<10 [6]	<10 [6]	<10 [6]	<10 [6]	<10 [6]	<10 [6]	<10 [6]
	Black or Black British	<10 [6]	<10 [6]	<10 [6]	<10 [6]	<10 [6]	<10 [6]	<10 [6]
	Chinese and Other	<10 [6]	<10 [6]	<10 [6]	<10 [6]	<10 [6]	<10 [6]	<10 [6]
	Unclassified	<10 [6]	<10 [6]	<10 [6]	<10 [6]	<10 [6]	<10 [6]	<10 [6]
2019/20	White	<10 [6]	<10 [6]	<10 [6]	<10 [6]	<10 [6]	<10 [6]	<10 [6]
	Mixed	<10 [6]	<10 [6]	<10 [6]	<10 [6]	<10 [6]	<10 [6]	<10 [6]
	Asian or Asian British	<10 [6]	<10 [6]	<10 [6]	<10 [6]	<10 [6]	<10 [6]	<10 [6]
	Black or Black British	<10 [6]	<10 [6]	<10 [6]	<10 [6]	<10 [6]	<10 [6]	<10 [6]
	Chinese and Other	<10 [6]	<10 [6]	<10 [6]	<10 [6]	<10 [6]	<10 [6]	<10 [6]
	Unclassified	<10 [6]	<10 [6]	<10 [6]	<10 [6]	<10 [6]	<10 [6]	<10 [6]

[1]Eligible for indicator; [2]Achieve in current prison; [3]Achieve in previous prison; [4]Overall achievement - either current or previous prison; [5]Declined indicator; [6]Suppressed (<10) to avoid disclosure

Indicator	The proportion of people with a new diagnosis of any form of dementia in the reporting year, and who have a record of the following blood tests between 12 months before and 6 months after the date of diagnosis: full blood count, calcium, glucose and/or HbA1C, urea and electrolytes, liver function, thyroid function, serum vitamin B12, folate levels<br>Group: Mental health			*Community achievement 2019/20 (%): no comparable data in QOF 2019/20. Most recent QOF data was from 2018/19 and achievement was 70.01%*
	**Variable**	**Population**	**Eligible[1] (% population)**	**Achieved[4] 5 of the 8 blood tests (% eligible)**
**Year**				
	2017/18	21,677	10 (0.0)	<10 [6]
	2018/19	22,099	10 (0.0)	<10 [6]
	2019/20	25,811	13 (0.1)	<10 [6]
**Prison**				
2017/18	Prison 1	1,323	<10 [6]	<10 [6]
	Prison 2	3,261	<10 [6]	<10 [6]
	Prison 3	2,623	<10 [6]	<10 [6]
	Prison 4	2,089	<10 [6]	<10 [6]
	Prison 5	637	<10 [6]	<10 [6]
	Prison 6	1,552	<10 [6]	<10 [6]
	Prison 7	635	<10 [6]	<10 [6]
	Prison 8	1,085	<10 [6]	<10 [6]
	Prison 9	981	<10 [6]	<10 [6]
	Prison 10	2,523	<10 [6]	<10 [6]
	Prison 11	3,470	<10 [6]	<10 [6]
	Prison 12	815	<10 [6]	<10 [6]
	Prison 13	683	<10 [6]	<10 [6]
2018/19	Prison 1	1,333	<10 [6]	<10 [6]
	Prison 2	2,705	<10 [6]	<10 [6]
	Prison 3	2,522	<10 [6]	<10 [6]
	Prison 4	2,349	<10 [6]	<10 [6]
	Prison 5	676	<10 [6]	<10 [6]
	Prison 6	1,513	<10 [6]	<10 [6]
	Prison 7	654	<10 [6]	<10 [6]
	Prison 8	1,148	<10 [6]	<10 [6]
	Prison 9	996	<10 [6]	<10 [6]
	Prison 10	2,717	<10 [6]	<10 [6]
	Prison 11	4,020	<10 [6]	<10 [6]
	Prison 12	792	<10 [6]	<10 [6]
	Prison 13	674	<10 [6]	<10 [6]
2019/20	Prison 1	1,410	<10 [6]	<10 [6]
	Prison 2	2,979	<10 [6]	<10 [6]
	Prison 3	2,809	<10 [6]	<10 [6]
	Prison 4	2,651	<10 [6]	<10 [6]
	Prison 5	616	<10 [6]	<10 [6]
	Prison 6	1,533	<10 [6]	<10 [6]
	Prison 7	860	<10 [6]	<10 [6]
	Prison 8	1,385	<10 [6]	<10 [6]
	Prison 9	1,092	<10 [6]	<10 [6]
	Prison 10	3,577	<10 [6]	<10 [6]
	Prison 11	5,348	<10 [6]	<10 [6]
	Prison 12	805	<10 [6]	<10 [6]
	Prison 13	746	<10 [6]	<10 [6]
**Prison category**				
2017/18	A	1,664	<10 [6]	<10 [6]
	B	9,254	<10 [6]	<10 [6]
	C	6,035	<10 [6]	<10 [6]
	Closed	1,720	<10 [6]	<10 [6]
	D	2,189	<10 [6]	<10 [6]
	YOI	815	<10 [6]	<10 [6]
2018/19	A	1,670	<10 [6]	<10 [6]
	B	9,442	<10 [6]	<10 [6]
	C	6,204	<10 [6]	<10 [6]
	Closed	1,802	<10 [6]	<10 [6]
	D	2,189	<10 [6]	<10 [6]
	YOI	792	<10 [6]	<10 [6]
2019/20	A	1,838	<10 [6]	<10 [6]
	B	11,904	<10 [6]	<10 [6]
	C	6,870	<10 [6]	<10 [6]
	Closed	2,245	<10 [6]	<10 [6]
	D	2,149	<10 [6]	<10 [6]
	YOI	805	<10 [6]	<10 [6]
**Gender**				
2017/18	F	1,699	<10 [6]	<10 [6]
	M	19,977	10 (0.1)	<10 [6]
2018/19	F	1,802	<10 [6]	<10 [6]
	M	20,295	10 (0.0)	<10 [6]
2019/20	F	1,376	<10 [6]	<10 [6]
	M	23,570	12 (0.1)	<10 [6]

[1]Eligible for indicator; [2]Achieve in current prison; [3]Achieve in previous prison; [4]Overall achievement - either current or previous prison; [5]Declined indicator; [6]Suppressed (<10) to avoid disclosure

Indicator	*The proportion of people with a new diagnosis of any form of dementia in the reporting year, and who have a record of the following blood tests between 12 months before and 6 months after the date of diagnosis: full blood count, calcium, glucose and/or HbA1C, urea and electrolytes, liver function, thyroid function, serum vitamin B12, folate levels*			***Community achievement 2019/20 (%): no comparable data in QOF 2019/20. Most recent QOF data was from 2018/19 and achievement was 70.01%***
	**Group: Mental health**			
	**Variable**	**Population**	**Eligible[1] (% population)**	**Achieved[4] 5 of the 8 blood tests (% eligible)**
**Sentence Status**				
2017/18	.	81	<10 [6]	<10 [6]
	Absconded	<10 [6]	<10 [6]	<10 [6]
	Active In	15,872	<10 [6]	<10 [6]
	Active Out	1,052	<10 [6]	<10 [6]
	Convicted Sentence	2,125	<10 [6]	<10 [6]
	Downgrade in security category	<10 [6]	<10 [6]	<10 [6]
	Internal Cell Move	<10 [6]	<10 [6]	<10 [6]
	Judges Remand	92	<10 [6]	<10 [6]
	Licence Revoke	52	<10 [6]	<10 [6]
	On Remand	1,492	<10 [6]	<10 [6]
	Transfer	910	<10 [6]	<10 [6]
	Upgrade in security category	<10 [6]	<10 [6]	<10 [6]
2018/19	.	88	<10 [6]	<10 [6]
	Absconded	<10 [6]	<10 [6]	<10 [6]
	Active In	18,145	<10 [6]	<10 [6]
	Active Out	835	<10 [6]	<10 [6]
	Convicted Sentence	1,320	<10 [6]	<10 [6]
	Downgrade in security category	<10 [6]	<10 [6]	<10 [6]
	Internal Cell Move	<10 [6]	<10 [6]	<10 [6]
	Judges Remand	<10 [6]	<10 [6]	<10 [6]
	Licence Revoke	125	<10 [6]	<10 [6]
	On Remand	1,059	<10 [6]	<10 [6]
	Transfer	518	<10 [6]	<10 [6]
	Upgrade in security category	<10 [6]	<10 [6]	<10 [6]
2019/20	.	69	<10 [6]	<10 [6]
	Absconded	<10 [6]	<10 [6]	<10 [6]
	Active In	22,424	10 (0.0)	<10 [6]
	Active Out	625	<10 [6]	<10 [6]
	Convicted Sentence	1,361	<10 [6]	<10 [6]
	Downgrade in security category	<10 [6]	<10 [6]	<10 [6]
	Internal Cell Move	<10 [6]	<10 [6]	<10 [6]
	Judges Remand	19	<10 [6]	<10 [6]
	Licence Revoke	178	<10 [6]	<10 [6]
	On Remand	1,031	<10 [6]	<10 [6]
	Transfer	101	<10 [6]	<10 [6]
	Upgrade in security category	<10 [6]	<10 [6]	<10 [6]
**Age - years**				
2017/18	10 - <20	468	<10 [6]	<10 [6]
	20 - <30	6,994	<10 [6]	<10 [6]
	30 - <40	7,051	<10 [6]	<10 [6]
	40 - <50	4,114	<10 [6]	<10 [6]
	50 - <60	2,107	<10 [6]	<10 [6]
	60 - <70	684	<10 [6]	<10 [6]
	70 - <80	213	<10 [6]	<10 [6]
	80 - <90	40	<10 [6]	<10 [6]
	90 - <100	<10 [6]	<10 [6]	<10 [6]
	100 - <110	<10 [6]	<10 [6]	<10 [6]
2018/19	10 - <20	436	<10 [6]	<10 [6]
	20 - <30	7,163	<10 [6]	<10 [6]
	30 - <40	7,381	<10 [6]	<10 [6]
	40 - <50	4,180	<10 [6]	<10 [6]
	50 - <60	1,978	<10 [6]	<10 [6]
	60 - <70	701	<10 [6]	<10 [6]
	70 - <80	209	<10 [6]	<10 [6]
	80 - <90	45	<10 [6]	<10 [6]
	90 - <100	<10 [6]	<10 [6]	<10 [6]
	100 - <110	<10 [6]	<10 [6]	<10 [6]
2019/20	10 - <20	404	<10 [6]	<10 [6]
	20 - <30	8,064	<10 [6]	<10 [6]
	30 - <40	9,125	<10 [6]	<10 [6]
	40 - <50	4,948	<10 [6]	<10 [6]
	50 - <60	2,224	<10 [6]	<10 [6]
	60 - <70	751	<10 [6]	<10 [6]
	70 - <80	238	<10 [6]	<10 [6]
	80 - <90	53	<10 [6]	<10 [6]
	90 - <100	<10 [6]	<10 [6]	<10 [6]
	100 - <110	<10 [6]	<10 [6]	<10 [6]

[1]Eligible for indicator; [2]Achieve in current prison; [3]Achieve in previous prison; [4]Overall achievement - either current or previous prison; [5]Declined indicator; [6]Suppressed (<10) to avoid disclosure

Indicator	*The proportion of people with a new diagnosis of any form of dementia in the reporting year, and who have a record of the following blood tests between 12 months before and 6 months after the date of diagnosis: full blood count, calcium, glucose and/or HbA1C, urea and electrolytes, liver function, thyroid function, serum vitamin B12, folate levels*			*Community achievement 2019/20 (%): no comparable data in QOF 2019/20. Most recent QOF data was from 2018/19 and achievement was 70.01%*
	**Group: Mental health**			
	**Variable**	**Population**	**Eligible[1] (% population)**	**Achieved[4] 5 of the 8 blood tests (% eligible)**
**Length of Stay (months)**				
2017/18	<1	4,474	<10 [6]	<10 [6]
	1-<6	8,075	<10 [6]	<10 [6]
	6-<12	3,672	<10 [6]	<10 [6]
	12-<24	2,832	<10 [6]	<10 [6]
	24+	2,624	<10 [6]	<10 [6]
2018/19	<1	4,801	<10 [6]	<10 [6]
	1-<6	7,742	<10 [6]	<10 [6]
	6-<12	3,616	<10 [6]	<10 [6]
	12-<24	3,447	<10 [6]	<10 [6]
	24+	2,493	<10 [6]	<10 [6]
2019/20	<1	5,745	<10 [6]	<10 [6]
	1-<6	9,697	<10 [6]	<10 [6]
	6-<12	5,090	<10 [6]	<10 [6]
	12-<24	3,244	<10 [6]	<10 [6]
	24+	2,035	<10 [6]	<10 [6]
**Ethnic Group**				
2017/18	White	15,638	<10 [6]	<10 [6]
	Mixed	431	<10 [6]	<10 [6]
	Asian or Asian British	813	<10 [6]	<10 [6]
	Black or Black British	404	<10 [6]	<10 [6]
	Chinese and Other	214	<10 [6]	<10 [6]
	Unclassified	372	<10 [6]	<10 [6]
2018/19	White	14,911	<10 [6]	<10 [6]
	Mixed	371	<10 [6]	<10 [6]
	Asian or Asian British	726	<10 [6]	<10 [6]
	Black or Black British	364	<10 [6]	<10 [6]
	Chinese and Other	167	<10 [6]	<10 [6]
	Unclassified	409	<10 [6]	<10 [6]
2019/20	White	16,606	10 (0.1)	<10 [6]
	Mixed	409	<10 [6]	<10 [6]
	Asian or Asian British	755	<10 [6]	<10 [6]
	Black or Black British	451	<10 [6]	<10 [6]
	Chinese and Other	163	<10 [6]	<10 [6]
	Unclassified	387	<10 [6]	<10 [6]

[1]Eligible for indicator; [2]Achieve in current prison; [3]Achieve in previous prison; [4]Overall achievement - either current or previous prison; [5]Declined indicator; [6]Suppressed (<10) to avoid disclosure

Indicator		The proportion of new receptions to prison accepting opt out dry blood spot testing					
	Group: Communicable Disease						
	Variable	Population	Eligible[1] (% popln)	Satisfy[2] (% eligible)	Elsewhere[3] (% eligible)	Achieve[4] (% eligible)	*Community achievement 2019/20 (%): NA*
**Year**							
	2017/18	21,677	13,072 (60.3)	81 (0.6)	64 (0.5)	145 (1.1)	
	2018/19	22,099	12,401 (56.1)	3,436 (27.7)	337 (2.7)	3,773 (30.4)	
	2019/20	25,811	12,606 (48.8)	4,711 (37.4)	942 (7.5)	5,653 (44.8)	
**Prison**							
2017/18	Prison 1	1,323	832 (62.9)	<10 [6]	<10 [6]	<10 [6]	
	Prison 2	3,261	2,313 (70.9)	<10 [6]	<10 [6]	<10 [6]	
	Prison 3	2,623	1,785 (68.1)	<10 [6]	16 (0.9)	22 (1.2)	
	Prison 4	2,089	1,049 (50.2)	<10 [6]	<10 [6]	10 (1.0)	
	Prison 5	637	370 (58.1)	<10 [6]	<10 [6]	<10 [6]	
	Prison 6	1,552	968 (62.4)	<10 [6]	<10 [6]	<10 [6]	
	Prison 7	635	410 (64.6)	17 (4.1)	<10 [6]	18 (4.4)	
	Prison 8	1,085	477 (44.0)	<10 [6]	<10 [6]	<10 [6]	
	Prison 9	981	206 (21.0)	27 (13.1)	<10 [6]	28 (13.6)	
	Prison 10	2,523	1,505 (59.7)	<10 [6]	<10 [6]	<10 [6]	
	Prison 11	3,470	2,578 (74.3)	29 (1.1)	<10 [6]	37 (1.4)	
	Prison 12	815	441 (54.1)	<10 [6]	<10 [6]	<10 [6]	
	Prison 13	683	138 (20.2)	<10 [6]	<10 [6]	<10 [6]	
2018/19	Prison 1	1,333	814 (61.1)	470 (57.7)	26 (3.2)	496 (60.9)	
	Prison 2	2,705	1,693 (62.6)	965 (57.0)	<10 [6]	967 (57.1)	
	Prison 3	2,522	1,340 (53.1)	167 (12.5)	76 (5.7)	243 (18.1)	
	Prison 4	2,349	1,139 (48.5)	207 (18.2)	64 (5.6)	271 (23.8)	
	Prison 5	676	397 (58.7)	93 (23.4)	21 (5.3)	114 (28.7)	
	Prison 6	1,513	926 (61.2)	302 (32.6)	64 (6.9)	366 (39.5)	
	Prison 7	654	322 (49.2)	168 (52.2)	<10 [6]	175 (54.3)	
	Prison 8	1,148	646 (56.3)	438 (67.8)	<10 [6]	438 (67.8)	
	Prison 9	996	191 (19.2)	25 (13.1)	10 (5.2)	35 (18.3)	
	Prison 10	2,717	1,939 (71.4)	<10 [6]	25 (1.3)	25 (1.3)	
	Prison 11	4,020	2,465 (61.3)	494 (20.0)	21 (0.9)	515 (20.9)	
	Prison 12	792	377 (47.6)	107 (28.4)	19 (5.0)	126 (33.4)	
	Prison 13	674	152 (22.6)	<10 [6]	<10 [6]	<10 [6]	
2019/20	Prison 1	1,410	779 (55.2)	690 (88.6)	30 (3.9)	720 (92.4)	
	Prison 2	2,979	1,693 (56.8)	1,066 (63.0)	119 (7.0)	1,185 (70.0)	
	Prison 3	2,809	1,266 (45.1)	181 (14.3)	312 (24.6)	493 (38.9)	
	Prison 4	2,651	1,108 (41.8)	550 (49.6)	150 (13.5)	700 (63.2)	
	Prison 5	616	334 (54.2)	187 (56.0)	47 (14.1)	234 (70.1)	
	Prison 6	1,533	856 (55.8)	340 (39.7)	84 (9.8)	424 (49.5)	
	Prison 7	860	376 (43.7)	214 (56.9)	17 (4.5)	231 (61.4)	
	Prison 8	1,385	658 (47.5)	547 (83.1)	<10 [6]	552 (83.9)	
	Prison 9	1,092	234 (21.4)	53 (22.6)	43 (18.4)	96 (41.0)	
	Prison 10	3,577	1,947 (54.4)	<10 [6]	42 (2.2)	42 (2.2)	
	Prison 11	5,348	2,798 (52.3)	656 (23.4)	53 (1.9)	709 (25.3)	
	Prison 12	805	347 (43.1)	212 (61.1)	34 (9.8)	246 (70.9)	
	Prison 13	746	210 (28.2)	15 (7.1)	<10 [6]	21 (10.0)	
**Prison category**							
2017/18	A	1,664	344 (20.7)	27 (7.8)	<10 [6]	30 (8.7)	
	B	9,254	6,396 (69.1)	29 (0.5)	15 (0.2)	44 (0.7)	
	C	6,035	3,666 (60.7)	<10 [6]	28 (0.8)	36 (1.0)	
	Closed	1,720	887 (51.6)	17 (1.9)	<10 [6]	19 (2.1)	
	D	2,189	1,338 (61.1)	<10 [6]	<10 [6]	<10 [6]	
	YOI	815	441 (54.1)	<10 [6]	<10 [6]	<10 [6]	
2018/19	A	1,670	343 (20.5)	25 (7.3)	12 (3.5)	37 (10.8)	
	B	9,442	6,097 (64.6)	1,459 (23.9)	48 (0.8)	1,507 (24.7)	
	C	6,204	3,293 (53.1)	844 (25.6)	166 (5.0)	1,010 (30.7)	
	Closed	1,802	968 (53.7)	606 (62.6)	<10 [6]	613 (63.3)	
	D	2,189	1,323 (60.4)	395 (29.9)	85 (6.4)	480 (36.3)	
	YOI	792	377 (47.6)	107 (28.4)	19 (5.0)	126 (33.4)	
2019/20	A	1,838	444 (24.2)	68 (15.3)	49 (11.0)	117 (26.4)	
	B	11,904	6,438 (54.1)	1,722 (26.7)	214 (3.3)	1,936 (30.1)	
	C	6,870	3,153 (45.9)	1,421 (45.1)	492 (15.6)	1,913 (60.7)	
	Closed	2,245	1,034 (46.1)	761 (73.6)	22 (2.1)	783 (75.7)	
	D	2,149	1,190 (55.4)	527 (44.3)	131 (11.0)	658 (55.3)	
	YOI	805	347 (43.1)	212 (61.1)	34 (9.8)	246 (70.9)	
**Gender**							
2017/18	F	1,699	884 (52.0)	17 (1.9)	<10 [6]	19 (2.1)	
	M	19,977	12,187 (61.0)	64 (0.5)	62 (0.5)	126 (1.0)	
2018/19	F	1,802	967 (53.7)	605 (62.6)	<10 [6]	612 (63.3)	
	M	20,295	11,432 (56.3)	2,830 (24.8)	330 (2.9)	3,160 (27.6)	
2019/20	F	1,376	657 (47.7)	547 (83.3)	<10 [6]	552 (84.0)	
	M	23,570	11,574 (49.1)	3,951 (34.1)	920 (7.9)	4,871 (42.1)	

[1]Eligible for indicator; [2]Achieve in current prison; [3]Achieve in previous prison; [4]Overall achievement - either current or previous prison; [5]Declined indicator; [6]Suppressed (<10) to avoid disclosure

Indicator		The proportion of new receptions to prison accepting opt out dry blood spot testing					
	Group: Communicable Disease						
	Variable	Population	Eligible[1] (% popln)	Satisfy[2] (% eligible)	Elsewhere[3] (% eligible)	Achieve[4] (% eligible)	*Community achievement 2019/20 (%): NA*
**Sentence Status**							
2017/18	.	81	<10 [6]	<10 [6]	<10 [6]	<10 [6]	
	Absconded	<10 [6]	<10 [6]	<10 [6]	<10 [6]	<10 [6]	
	Active In	15,872	9,626 (60.6)	76 (0.8)	59 (0.6)	135 (1.4)	
	Active Out	1,052	551 (52.4)	<10 [6]	<10 [6]	<10 [6]	
	Convicted Sentence	2,125	1,355 (63.8)	<10 [6]	<10 [6]	<10 [6]	
	Downgrade in security category	<10 [6]	<10 [6]	<10 [6]	<10 [6]	<10 [6]	
	Internal Cell Move	<10 [6]	<10 [6]	<10 [6]	<10 [6]	<10 [6]	
	Judges Remand	92	23 (25.0)	<10 [6]	<10 [6]	<10 [6]	
	Licence Revoke	52	50 (96.2)	<10 [6]	<10 [6]	<10 [6]	
	On Remand	1,492	964 (64.6)	<10 [6]	<10 [6]	<10 [6]	
	Transfer	910	499 (54.8)	<10 [6]	<10 [6]	<10 [6]	
	Upgrade in security category	<10 [6]	<10 [6]	<10 [6]	<10 [6]	<10 [6]	
2018/19	.	88	<10 [6]	<10 [6]	<10 [6]	<10 [6]	
	Absconded	<10 [6]	<10 [6]	<10 [6]	<10 [6]	<10 [6]	
	Active In	18,145	10,090 (55.6)	2,433 (24.1)	321 (3.2)	2,754 (27.3)	
	Active Out	835	499 (59.8)	81 (16.2)	<10 [6]	87 (17.4)	
	Convicted Sentence	1,320	831 (63.0)	427 (51.4)	<10 [6]	431 (51.9)	
	Downgrade in security category	<10 [6]	<10 [6]	<10 [6]	<10 [6]	<10 [6]	
	Internal Cell Move	<10 [6]	<10 [6]	<10 [6]	<10 [6]	<10 [6]	
	Judges Remand	<10 [6]	<10 [6]	<10 [6]	<10 [6]	<10 [6]	
	Licence Revoke	125	67 (53.6)	32 (47.8)	<10 [6]	32 (47.8)	
	On Remand	1,059	651 (61.5)	384 (59.0)	<10 [6]	384 (59.0)	
	Transfer	518	254 (49.0)	77 (30.3)	<10 [6]	83 (32.7)	
	Upgrade in security category	<10 [6]	<10 [6]	<10 [6]	<10 [6]	<10 [6]	
2019/20	.	69	<10 [6]	<10 [6]	<10 [6]	<10 [6]	
	Absconded	<10 [6]	<10 [6]	<10 [6]	<10 [6]	<10 [6]	
	Active In	22,424	10,778 (48.1)	3,676 (34.1)	852 (7.9)	4,528 (42.0)	
	Active Out	625	351 (56.2)	106 (30.2)	<10 [6]	114 (32.5)	
	Convicted Sentence	1,361	803 (59.0)	520 (64.8)	40 (5.0)	560 (69.7)	
	Downgrade in security category	<10 [6]	<10 [6]	<10 [6]	<10 [6]	<10 [6]	
	Internal Cell Move	<10 [6]	<10 [6]	<10 [6]	<10 [6]	<10 [6]	
	Judges Remand	19	11 (57.9)	<10 [6]	<10 [6]	<10 [6]	
	Licence Revoke	178	82 (46.1)	50 (61.0)	<10 [6]	55 (67.1)	
	On Remand	1,031	559 (54.2)	350 (62.6)	35 (6.3)	385 (68.9)	
	Transfer	101	20 (19.8)	<10 [6]	<10 [6]	<10 [6]	
	Upgrade in security category	<10 [6]	<10 [6]	<10 [6]	<10 [6]	<10 [6]	
**Age - years**							
2017/18	10 - <20	468	357 (76.3)	<10 [6]	<10 [6]	<10 [6]	
	20 - <30	6,994	4,575 (65.4)	25 (0.5)	21 (0.5)	46 (1.0)	
	30 - <40	7,051	4,315 (61.2)	26 (0.6)	21 (0.5)	47 (1.1)	
	40 - <50	4,114	2,381 (57.9)	13 (0.5)	10 (0.4)	23 (1.0)	
	50 - <60	2,107	1,061 (50.4)	<10 [6]	<10 [6]	13 (1.2)	
	60 - <70	684	284 (41.5)	<10 [6]	<10 [6]	<10 [6]	
	70 - <80	213	78 (36.6)	<10 [6]	<10 [6]	<10 [6]	
	80 - <90	40	20 (50.0)	<10 [6]	<10 [6]	<10 [6]	
	90 - <100	<10 [6]	<10 [6]	<10 [6]	<10 [6]	<10 [6]	
	100 - <110	<10 [6]	<10 [6]	<10 [6]	<10 [6]	<10 [6]	
2018/19	10 - <20	436	319 (73.2)	71 (22.3)	13 (4.1)	84 (26.3)	
	20 - <30	7,163	4,259 (59.5)	1,186 (27.8)	116 (2.7)	1,302 (30.6)	
	30 - <40	7,381	4,202 (56.9)	1,168 (27.8)	99 (2.4)	1,267 (30.2)	
	40 - <50	4,180	2,232 (53.4)	639 (28.6)	62 (2.8)	701 (31.4)	
	50 - <60	1,978	977 (49.4)	283 (29.0)	31 (3.2)	314 (32.1)	
	60 - <70	701	319 (45.5)	71 (22.3)	14 (4.4)	85 (26.6)	
	70 - <80	209	76 (36.4)	12 (15.8)	<10 [6]	13 (17.1)	
	80 - <90	45	14 (31.1)	<10 [6]	<10 [6]	<10 [6]	
	90 - <100	<10 [6]	<10 [6]	<10 [6]	<10 [6]	<10 [6]	
	100 - <110	<10 [6]	<10 [6]	<10 [6]	<10 [6]	<10 [6]	
2019/20	10 - <20	404	300 (74.3)	102 (34.0)	22 (7.3)	124 (41.3)	
	20 - <30	8,064	4,125 (51.2)	1,557 (37.7)	342 (8.3)	1,899 (46.0)	
	30 - <40	9,125	4,460 (48.9)	1,709 (38.3)	303 (6.8)	2,012 (45.1)	
	40 - <50	4,948	2,319 (46.9)	862 (37.2)	193 (8.3)	1,055 (45.5)	
	50 - <60	2,224	999 (44.9)	355 (35.5)	54 (5.4)	409 (40.9)	
	60 - <70	751	289 (38.5)	90 (31.1)	21 (7.3)	111 (38.4)	
	70 - <80	238	94 (39.5)	31 (33.0)	<10 [6]	37 (39.4)	
	80 - <90	53	20 (37.7)	<10 [6]	<10 [6]	<10 [6]	
	90 - <100	<10 [6]	<10 [6]	<10 [6]	<10 [6]	<10 [6]	
	100 - <110	<10 [6]	<10 [6]	<10 [6]	<10 [6]	<10 [6]	

[1]Eligible for indicator; [2]Achieve in current prison; [3]Achieve in previous prison; [4]Overall achievement - either current or previous prison; [5]Declined indicator; [6]Suppressed (<10) to avoid disclosure

Indicator ***The proportion of new receptions to prison accepting opt out dry blood spot testing***

Group: Communicable Disease

	**Variable**	**Population**	**Eligible[1] (% popln)**	**Satisfy[2] (% eligible)**	**Elsewhere[3] (% eligible)**	**Achieve[4] (% eligible)**	*Community achievement 2019/20 (%): NA*
**Length of Stay (months)**							
2017/18	<1	4,474	3,510 (78.5)	<10 [6]	<10 [6]	14 (0.4)	
	1-<6	8,075	5,864 (72.6)	36 (0.6)	20 (0.3)	56 (1.0)	
	6-<12	3,672	1,967 (53.6)	<10 [6]	14 (0.7)	21 (1.1)	
	12-<24	2,832	1,126 (39.8)	10 (0.9)	<10 [6]	17 (1.5)	
	24+	2,624	605 (23.1)	23 (3.8)	14 (2.3)	37 (6.1)	
2018/19	<1	4,801	3,468 (72.2)	707 (20.4)	37 (1.1)	744 (21.5)	
	1-<6	7,742	5,253 (67.9)	1,558 (29.7)	113 (2.2)	1,671 (31.8)	
	6-<12	3,616	1,807 (50.0)	611 (33.8)	63 (3.5)	674 (37.3)	
	12-<24	3,752	1,872 (49.9)	560 (29.9)	124 (6.6)	684 (36.5)	
	24+	2,188	<10 [6]	<10 [6]	<10 [6]	<10 [6]	
2019/20	<1	6,764	4,081 (60.3)	1,058 (25.9)	207 (5.1)	1,265 (31.0)	
	1-<6	10,802	6,697 (62.0)	2,723 (40.7)	586 (8.8)	3,309 (49.4)	
	6-<12	3,893	1,813 (46.6)	922 (50.9)	149 (8.2)	1,071 (59.1)	
	12-<24	2,600	15 (0.6)	<10 [6]	<10 [6]	<10 [6]	
	24+	1,752	<10 [6]	<10 [6]	<10 [6]	<10 [6]	
**Ethnic Group**							
2017/18	White	15,638	9,346 (59.8)	59 (0.6)	52 (0.6)	111 (1.2)	
	Mixed	431	223 (51.7)	<10 [6]	<10 [6]	<10 [6]	
	Asian or Asian British	813	472 (58.1)	<10 [6]	<10 [6]	<10 [6]	
	Black or Black British	404	203 (50.2)	<10 [6]	<10 [6]	<10 [6]	
	Chinese and Other	214	118 (55.1)	<10 [6]	<10 [6]	<10 [6]	
	Unclassified	372	190 (51.1)	<10 [6]	<10 [6]	<10 [6]	
2018/19	White	14,911	7,650 (51.3)	2,235 (29.2)	224 (2.9)	2,459 (32.1)	
	Mixed	371	186 (50.1)	62 (33.3)	<10 [6]	66 (35.5)	
	Asian or Asian British	726	363 (50.0)	98 (27.0)	20 (5.5)	118 (32.5)	
	Black or Black British	364	173 (47.5)	62 (35.8)	<10 [6]	68 (39.3)	
	Chinese and Other	167	95 (56.9)	27 (28.4)	<10 [6]	27 (28.4)	
	Unclassified	409	202 (49.4)	47 (23.3)	<10 [6]	54 (26.7)	
2019/20	White	16,606	7,016 (42.2)	2,849 (40.6)	615 (8.8)	3,464 (49.4)	
	Mixed	409	186 (45.5)	84 (45.2)	16 (8.6)	100 (53.8)	
	Asian or Asian British	755	334 (44.2)	127 (38.0)	23 (6.9)	150 (44.9)	
	Black or Black British	451	213 (47.2)	89 (41.8)	21 (9.9)	110 (51.6)	
	Chinese and Other	163	82 (50.3)	34 (41.5)	<10 [6]	41 (50.0)	
	Unclassified	387	153 (39.5)	44 (28.8)	12 (7.8)	56 (36.6)	

[1]Eligible for indicator; [2]Achieve in current prison; [3]Achieve in previous prison; [4]Overall achievement - either current or previous prison; [5]Declined indicator; [6]Suppressed (<10) to avoid disclosure

Indicator	*The proportion of the prison population who have received one, two, three or four (course of three plus booster) Hepatitis B vaccinations in the preceding 12 months*						*Community achievement 2019/20 (%): NA*
	**Group: Communicable Disease**						
				**Hepatitis B vaccines of the population who entered in the last 12 months**			
				**1st Hep B**	**1st and 2nd Hep B**	**1st, 2nd and 3rd Hep B**	**1st, 2nd, 3rd and 4th Hep B**
	**Variable**	**Eligible[1]**	**Declined[5] (%)**	**Satisfy[2] (% eligible)**	**Satisfy[2] (% eligible)**	**Satisfy[2] (% eligible)**	**Satisfy[2] (% eligible)**
**Year**							
	2017/18	14,804	217 (1.5)	4,417 (29.8)	3,274 (22.1)	2,514 (17.0)	863 (5.8)
	2018/19	15,187	275 (1.8)	4,165 (27.4)	3,325 (21.9)	1,211 (8.0)	1,211 (8.0)
	2019/20	17,957	410 (2.3)	8,210 (45.7)	6,676 (37.2)	5,437 (30.3)	2,212 (12.3)
**Prison**							
2017/18	Prison 1	853	<10 [6]	227 (26.6)	182 (21.3)	148 (17.4)	59 (6.9)
	Prison 2	2,520	59 (2.3)	520 (20.6)	382 (15.2)	274 (10.9)	68 (2.7)
	Prison 3	1,834	26 (1.4)	581 (31.7)	444 (24.2)	343 (18.7)	153 (8.3)
	Prison 4	1,092	19 (1.7)	412 (37.7)	329 (30.1)	278 (25.5)	106 (9.7)
	Prison 5	379	<10 [6]	135 (35.6)	114 (30.1)	98 (25.9)	27 (7.1)
	Prison 6	990	<10 [6]	365 (36.9)	308 (31.1)	257 (26.0)	71 (7.2)
	Prison 7	426	<10 [6]	225 (52.8)	166 (39.0)	145 (34.0)	57 (13.4)
	Prison 8	803	21 (2.6)	319 (39.7)	244 (30.4)	178 (22.2)	46 (5.7)
	Prison 9	241	<10 [6]	86 (35.7)	72 (29.9)	60 (24.9)	28 (11.6)
	Prison 10	2,070	32 (1.5)	553 (26.7)	327 (15.8)	208 (10.0)	67 (3.2)
	Prison 11	2,980	26 (0.9)	809 (27.1)	558 (18.7)	407 (13.7)	156 (5.2)
	Prison 12	449	10 (2.2)	134 (29.8)	109 (24.3)	87 (19.4)	16 (3.6)
	Prison 13	167	<10 [6]	51 (30.5)	39 (23.4)	31 (18.6)	9 (5.4)
2018/19	Prison 1	868	<10 [6]	222 (25.6)	181 (20.9)	76 (8.8)	76 (8.8)
	Prison 2	2,087	57 (2.7)	392 (18.8)	306 (14.7)	108 (5.2)	108 (5.2)
	Prison 3	1,640	23 (1.4)	463 (28.2)	367 (22.4)	177 (10.8)	177 (10.8)
	Prison 4	1,322	25 (1.9)	481 (36.4)	414 (31.3)	154 (11.6)	154 (11.6)
	Prison 5	420	<10 [6]	145 (34.5)	120 (28.6)	43 (10.2)	43 (10.2)
	Prison 6	977	12 (1.2)	357 (36.5)	317 (32.4)	90 (9.2)	90 (9.2)
	Prison 7	431	11 (2.6)	231 (53.6)	190 (44.1)	79 (18.3)	79 (18.3)
	Prison 8	844	31 (3.7)	277 (32.8)	217 (25.7)	61 (7.2)	61 (7.2)
	Prison 9	250	<10 [6]	86 (34.4)	69 (27.6)	38 (15.2)	38 (15.2)
	Prison 10	2,266	70 (3.1)	434 (19.2)	300 (13.2)	136 (6.0)	136 (6.0)
	Prison 11	3,447	35 (1.0)	806 (23.4)	606 (17.6)	205 (5.9)	205 (5.9)
	Prison 12	450	<10 [6]	220 (48.9)	196 (43.6)	25 (5.6)	25 (5.6)
	Prison 13	185	<10 [6]	51 (27.6)	42 (22.7)	19 (10.3)	19 (10.3)
2019/20	Prison 1	927	<10 [6]	533 (57.5)	472 (50.9)	414 (44.7)	159 (17.2)
	Prison 2	2,419	62 (2.6)	748 (30.9)	574 (23.7)	445 (18.4)	178 (7.4)
	Prison 3	1,780	50 (2.8)	784 (44.0)	657 (36.9)	537 (30.2)	295 (16.6)
	Prison 4	1,522	50 (3.3)	847 (55.7)	723 (47.5)	615 (40.4)	274 (18.0)
	Prison 5	354	<10 [6]	182 (51.4)	160 (45.2)	143 (40.4)	51 (14.4)
	Prison 6	904	<10 [6]	504 (55.8)	441 (48.8)	373 (41.3)	135 (14.9)
	Prison 7	603	19 (3.2)	467 (77.4)	415 (68.8)	352 (58.4)	150 (24.9)
	Prison 8	1,048	42 (4.0)	495 (47.2)	375 (35.8)	292 (27.9)	115 (11.0)
	Prison 9	276	<10 [6]	147 (53.3)	122 (44.2)	106 (38.4)	56 (20.3)
	Prison 10	2,760	111 (4.0)	1,518 (55.0)	1,198 (43.4)	936 (33.9)	306 (11.1)
	Prison 11	4,676	60 (1.3)	1,560 (33.4)	1,156 (24.7)	889 (19.0)	408 (8.7)
	Prison 12	441	<10 [6]	312 (70.7)	291 (66.0)	267 (60.5)	57 (12.9)
	Prison 13	247	<10 [6]	113 (45.7)	92 (37.2)	68 (27.5)	28 (11.3)
**Prison category**							
2017/18	A	408	<10 [6]	137 (33.6)	111 (27.2)	91 (22.3)	37 (9.1)
	B	7,570	117 (1.5)	1,882 (24.9)	1,267 (16.7)	889 (11.7)	291 (3.8)
	C	3,779	45 (1.2)	1,220 (32.3)	955 (25.3)	769 (20.3)	318 (8.4)
	Closed	1,229	30 (2.4)	544 (44.3)	410 (33.4)	323 (26.3)	103 (8.4)
	YOI	449	10 (2.2)	134 (29.8)	109 (24.3)	87 (19.4)	16 (3.6)
2018/19	A	435	<10 [6]	137 (31.5)	111 (25.5)	57 (13.1)	57 (13.1)
	B	7,800	162 (2.1)	1,632 (20.9)	1,212 (15.5)	449 (5.8)	449 (5.8)
	C	3,830	48 (1.3)	1,166 (30.4)	962 (25.1)	407 (10.6)	407 (10.6)
	Closed	1,275	42 (3.3)	508 (39.8)	407 (31.9)	140 (11.0)	140 (11.0)
	YOI	450	<10 [6]	220 (48.9)	196 (43.6)	25 (5.6)	25 (5.6)
2019/20	A	523	<10 [6]	260 (49.7)	214 (40.9)	174 (33.3)	84 (16.1)
	B	9,855	233 (2.4)	3,826 (38.8)	2,928 (29.7)	2,270 (23.0)	892 (9.1)
	C	4,229	100 (2.4)	2,164 (51.2)	1,852 (43.8)	1,566 (37.0)	728 (17.2)
	Closed	1,651	61 (3.7)	962 (58.3)	790 (47.8)	644 (39.0)	265 (16.1)
	D	1,258	<10 [6]	686 (54.5)	601 (47.8)	516 (41.0)	186 (14.8)
	YOI	441	<10 [6]	312 (70.7)	291 (66.0)	267 (60.5)	57 (12.9)
**Gender**							
2017/18	F	1,208	30 (2.5)	539 (44.6)	406 (33.6)	321 (26.6)	103 (8.5)
	M	13,595	187 (1.4)	3,878 (28.5)	2,868 (21.1)	2,193 (16.1)	760 (5.6)
2018/19	F	1,275	42 (3.3)	508 (39.8)	407 (31.9)	140 (11.0)	140 (11.0)
	M	13,910	233 (1.7)	3,657 (26.3)	2,918 (21.0)	1,071 (7.7)	1,071 (7.7)
2019/20	F	1,042	42 (4.0)	495 (47.5)	375 (36.0)	292 (28.0)	115 (11.0)
	M	16,307	349 (2.1)	7,246 (44.4)	5,885 (36.1)	4,793 (29.4)	1,946 (11.9)

[1]Eligible for indicator; [2]Achieve in current prison; [3]Achieve in previous prison; [4]Overall achievement - either current or previous prison; [5]Declined indicator; [6]Suppressed (<10) to avoid disclosure

Indicator *The proportion of the prison population who have received one, two, three or four (course of three plus booster) Hepatitis B vaccinations in the preceding 12 months*

Group: Communicable Disease

Community achievement 2019/20 (%): NA

				Hepatitis B vaccines of the population who entered in the last 12 months			
				1st Hep B	1st and 2nd Hep B	1st, 2nd and 3rd Hep B	1st, 2nd, 3rd and 4th Hep B
	**Variable**	**Eligible[1]**	**Declined[5] (%)**	**Satisfy[2] (% eligible)**	**Satisfy[2] (% eligible)**	**Satisfy[2] (% eligible)**	**Satisfy[2] (% eligible)**
**Sentence Status**							
2017/18	.	75	<10 [6]	18 (24.0)	17 (22.7)	11 (14.7)	<10 [6]
	Absconded	<10 [o]	<10 [6]	<10 [6]	<10 [6]	<10 [6]	<10 [6]
	Active In	10,623	146 (1.4)	3,326 (31.3)	2,494 (23.5)	1,938 (18.2)	692 (6.5)
	Active Out	816	<10 [6]	231 (28.3)	171 (21.0)	124 (15.2)	41 (5.0)
	Convicted Sentence	1,562	42 (2.7)	367 (23.5)	256 (16.4)	180 (11.5)	56 (3.6)
	Downgrade in security category	<10 [o]	<10 [6]	<10 [6]	<10 [6]	<10 [6]	<10 [6]
	Internal Cell Move	<10 [o]	<10 [6]	<10 [6]	<10 [6]	<10 [6]	<10 [6]
	Judges Remand	52	<10 [6]	22 (42.3)	<10 [6]	<10 [6]	<10 [6]
	Licence Revoke	51	<10 [6]	12 (23.5)	<10 [6]	<10 [6]	<10 [6]
	On Remand	1,067	18 (1.7)	246 (23.1)	168 (15.7)	124 (11.6)	31 (2.9)
	Transfer	558	<10 [6]	195 (34.9)	156 (28.0)	127 (22.8)	37 (6.6)
	Upgrade in security category	<10 [o]	<10 [6]	<10 [6]	<10 [6]	<10 [6]	<10 [6]
2018/19	.	84	<10 [6]	13 (15.5)	<10 [6]	<10 [6]	<10 [6]
	Absconded	<10 [o]	<10 [6]	<10 [6]	<10 [6]	<10 [6]	<10 [6]
	Active In	12,307	216 (1.8)	3,551 (28.9)	2,857 (23.2)	1,048 (8.5)	1,048 (8.5)
	Active Out	580	<10 [6]	134 (23.1)	99 (17.1)	38 (6.6)	38 (6.6)
	Convicted Sentence	1,050	29 (2.8)	217 (20.7)	175 (16.7)	65 (6.2)	65 (6.2)
	Downgrade in security category	<10 [o]	<10 [6]	<10 [6]	<10 [6]	<10 [6]	<10 [6]
	Internal Cell Move	<10 [o]	<10 [6]	<10 [6]	<10 [6]	<10 [6]	<10 [6]
	Judges Remand	<10 [o]	<10 [6]	<10 [6]	<10 [6]	<10 [6]	<10 [6]
	Licence Revoke	108	<10 [6]	28 (25.9)	22 (20.4)	<10 [6]	<10 [6]
	On Remand	768	16 (2.1)	145 (18.9)	105 (13.7)	39 (5.1)	39 (5.1)
	Transfer	281	<10 [6]	76 (27.0)	57 (20.3)	<10 [6]	<10 [6]
	Upgrade in security category	<10 [o]	<10 [6]	<10 [6]	<10 [6]	<10 [6]	<10 [6]
2019/20	.	61	<10 [6]	10 (16.4)	<10 [6]	<10 [6]	<10 [6]
	Absconded	<10 [o]	<10 [6]	<10 [6]	<10 [6]	<10 [6]	<10 [6]
	Active In	15,308	353 (2.3)	7,374 (48.2)	6,022 (39.3)	4,935 (32.2)	2,029 (13.3)
	Active Out	448	<10 [6]	183 (40.8)	148 (33.0)	116 (25.9)	40 (8.9)
	Convicted Sentence	1,148	24 (2.1)	337 (29.4)	267 (23.3)	200 (17.4)	74 (6.4)
	Downgrade in security category	<10 [o]	<10 [6]	<10 [6]	<10 [6]	<10 [6]	<10 [6]
	Internal Cell Move	<10 [o]	<10 [6]	<10 [6]	<10 [6]	<10 [6]	<10 [6]
	Judges Remand	15	<10 [6]	<10 [6]	<10 [6]	<10 [6]	<10 [6]
	Licence Revoke	156	<10 [6]	64 (41.0)	50 (32.1)	37 (23.7)	16 (10.3)
	On Remand	781	20 (2.6)	232 (29.7)	176 (22.5)	141 (18.1)	52 (6.7)
	Transfer	37	<10 [6]	<10 [6]	<10 [6]	<10 [6]	<10 [6]
	Upgrade in security category	<10 [o]	<10 [6]	<10 [6]	<10 [6]	<10 [6]	<10 [6]
**Age - years**							
2017/18	10 - <20	391	<10 [6]	69 (17.6)	44 (11.3)	35 (9.0)	<10 [6]
	20 - <30	5,022	61 (1.2)	1,523 (30.3)	1,134 (22.6)	872 (17.4)	292 (5.8)
	30 - <40	4,888	87 (1.8)	1,495 (30.6)	1,101 (22.5)	833 (17.0)	292 (6.0)
	40 - <50	2,744	43 (1.6)	805 (29.3)	610 (22.2)	470 (17.1)	170 (6.2)
	50 - <60	1,268	17 (1.3)	400 (31.5)	295 (23.3)	234 (18.5)	76 (6.0)
	60 - <70	362	<10 [6]	96 (26.5)	69 (19.1)	51 (14.1)	18 (5.0)
	70 - <80	98	<10 [6]	24 (24.5)	18 (18.4)	16 (16.3)	<10 [6]
	80 - <90	26	<10 [6]	<10 [6]	<10 [6]	<10 [6]	<10 [6]
	90 - <100	<10 [o]	<10 [6]	<10 [6]	<10 [6]	<10 [6]	<10 [6]
	100 - <110	<10 [o]	<10 [6]	<10 [6]	<10 [6]	<10 [6]	<10 [6]
2018/19	10 - <20	375	<10 [6]	110 (29.3)	89 (23.7)	10 (2.7)	10 (2.7)
	20 - <30	5,241	75 (1.4)	1,496 (28.5)	1,188 (22.7)	433 (8.3)	433 (8.3)
	30 - <40	5,204	113 (2.2)	1,412 (27.1)	1,125 (21.6)	418 (8.0)	418 (8.0)
	40 - <50	2,769	64 (2.3)	750 (27.1)	600 (21.7)	239 (8.6)	239 (8.6)
	50 - <60	1,133	12 (1.1)	296 (26.1)	243 (21.4)	90 (7.9)	90 (7.9)
	60 - <70	364	<10 [6]	85 (23.4)	68 (18.7)	17 (4.7)	17 (4.7)
	70 - <80	79	<10 [6]	13 (16.5)	10 (12.7)	<10 [6]	<10 [6]
	80 - <90	18	<10 [6]	<10 [6]	<10 [6]	<10 [6]	<10 [6]
	90 - <100	<10 [o]	<10 [6]	<10 [6]	<10 [6]	<10 [6]	<10 [6]
	100 - <110	<10 [o]	<10 [6]	<10 [6]	<10 [6]	<10 [6]	<10 [6]
2019/20	10 - <20	342	<10 [6]	182 (53.2)	158 (46.2)	134 (39.2)	16 (4.7)
	20 - <30	5,819	91 (1.6)	2,631 (45.2)	2,185 (37.5)	1,755 (30.2)	718 (12.3)
	30 - <40	6,583	197 (3.0)	3,113 (47.3)	2,508 (38.1)	2,048 (31.1)	916 (13.9)
	40 - <50	3,395	92 (2.7)	1,552 (45.7)	1,226 (36.1)	1,001 (29.5)	408 (12.0)
	50 - <60	1,323	24 (1.8)	545 (41.2)	449 (33.9)	371 (28.0)	126 (9.5)
	60 - <70	365	<10 [6]	143 (39.2)	116 (31.8)	99 (27.1)	19 (5.2)
	70 - <80	104	<10 [6]	36 (34.6)	27 (26.0)	24 (23.1)	<10 [6]
	80 - <90	25	<10 [6]	<10 [6]	<10 [6]	<10 [6]	<10 [6]
	90 - <100	<10 [o]	<10 [6]	<10 [6]	<10 [6]	<10 [6]	<10 [6]
	100 - <110	<10 [o]	<10 [6]	<10 [6]	<10 [6]	<10 [6]	<10 [6]

[1]Eligible for indicator; [2]Achieve in current prison; [3]Achieve in previous prison; [4]Overall achievement - either current or previous prison; [5]Declined indicator; [6]Suppressed (<10) to avoid disclosure

Indicator ***The proportion of the prison population who have received one, two, three or four (course of three plus booster) Hepatitis B vaccinations in the preceding 12 months***

Group: Communicable Disease

Community achievement 2019/20 (%): NA

	Variable	Eligible[1]	Declined[5] (%)	Hepatitis B vaccines of the population who entered in the last 12 months: 1st Hep B Satisfy[2] (% eligible)	1st and 2nd Hep B Satisfy[2] (% eligible)	1st, 2nd and 3rd Hep B Satisfy[2] (% eligible)	1st, 2nd, 3rd and 4th Hep B Satisfy[2] (% eligible)
**Length of Stay (months)**							
2017/18	<1	4,305	39 (0.9)	1,031 (23.9)	686 (15.9)	493 (11.5)	182 (4.2)
	1-<6	6,393	99 (1.5)	1,901 (29.7)	1,397 (21.9)	1,056 (16.5)	327 (5.1)
	6-<12	2,119	31 (1.5)	668 (31.5)	520 (24.5)	409 (19.3)	128 (6.0)
	12-<24	1,247	22 (1.8)	477 (38.3)	392 (31.4)	322 (25.8)	123 (9.9)
	24+	740	26 (3.5)	340 (45.9)	279 (37.7)	234 (31.6)	103 (13.9)
2018/19	<1	4,620	60 (1.3)	878 (19.0)	638 (13.8)	229 (5.0)	229 (5.0)
	1-<6	6,311	126 (2.0)	1,626 (25.8)	1,250 (19.8)	426 (6.8)	426 (6.8)
	6-<12	2,127	44 (2.1)	670 (31.5)	559 (26.3)	183 (8.6)	183 (8.6)
	12-<24	2,128	45 (2.1)	990 (46.5)	877 (41.2)	373 (17.5)	373 (17.5)
	24+	<10 [6]	<10 [6]	<10 [6]	<10 [6]	<10 [6]	<10 [6]
2019/20	<1	6,326	132 (2.1)	2,218 (35.1)	1,610 (25.5)	1,183 (18.7)	565 (8.9)
	1-<6	9,250	234 (2.5)	4,614 (49.9)	3,848 (41.6)	3,154 (34.1)	1,218 (13.2)
	6-<12	2,363	44 (1.9)	1,368 (57.9)	1,209 (51.2)	1,091 (46.2)	428 (18.1)
	12-<24	18	<10 [6]	10 (55.6)	<10 [6]	<10 [6]	<10 [6]
	24+	<10 [6]	<10 [6]	<10 [6]	<10 [6]	<10 [6]	<10 [6]
**Ethnic Group**							
2017/18	White	10,520	182 (1.7)	3,595 (34.2)	2,714 (25.8)	2,123 (20.2)	766 (7.3)
	Mixed	259	<10 [6]	77 (29.7)	52 (20.1)	35 (13.5)	<10 [6]
	Asian or Asian British	542	<10 [6]	189 (34.9)	134 (24.7)	106 (19.6)	31 (5.7)
	Black or Black British	249	<10 [6]	62 (24.9)	42 (16.9)	34 (13.7)	<10 [6]
	Chinese and Other	138	<10 [6]	34 (24.6)	26 (18.8)	18 (13.0)	<10 [6]
	Unclassified	216	<10 [6]	56 (25.9)	41 (19.0)	24 (11.1)	10 (4.6)
2018/19	White	9,734	227 (2.3)	3,184 (32.7)	2,571 (26.4)	1,041 (10.7)	1,041 (10.7)
	Mixed	217	<10 [6]	68 (31.3)	57 (26.3)	23 (10.6)	23 (10.6)
	Asian or Asian British	439	<10 [6]	140 (31.9)	114 (26.0)	43 (9.8)	43 (9.8)
	Black or Black British	227	<10 [6]	63 (27.8)	56 (24.7)	15 (6.6)	15 (6.6)
	Chinese and Other	111	<10 [6]	28 (25.2)	23 (20.7)	6 (5.4)	<10 [6]
	Unclassified	256	<10 [6]	60 (23.4)	44 (17.2)	12 (4.7)	12 (4.7)
2019/20	White	11,251	345 (3.1)	6,011 (53.4)	5,002 (44.5)	4,148 (36.9)	1,912 (17.0)
	Mixed	250	<10 [6]	136 (54.4)	112 (44.8)	90 (36.0)	44 (17.6)
	Asian or Asian British	429	<10 [6]	223 (52.0)	186 (43.4)	156 (36.4)	69 (16.1)
	Black or Black British	285	<10 [6]	142 (49.8)	119 (41.8)	96 (33.7)	36 (12.6)
	Chinese and Other	105	<10 [6]	45 (42.9)	35 (33.3)	28 (26.7)	<10 [6]
	Unclassified	228	<10 [6]	93 (40.8)	76 (33.3)	57 (25.0)	20 (8.8)

[1]Eligible for indicator; [2]Achieve in current prison; [3]Achieve in previous prison; [4]Overall achievement - either current or previous prison; [5]Declined indicator; [6]Suppressed (<10) to avoid disclosure

Indicator *The proportion of the prison population who have received one, two, three or four (course of three plus booster) Hepatitis B vaccinations in the preceding 12 months*
Group: Communicable Disease

Community achievement 2019/20 (%): NA

Patients resident in the study prison in the last 12 months with a clinical code added at any time indicating a history of any illicit drug use

	Variable	Eligible[1]	Declined (%)[5]	1st Hep B Satisfy[2] (% eligible)	1st and 2nd Hep B Satisfy[2] (% eligible)	1st, 2nd and 3rd Hep B Satisfy[2] (% eligible)	1st, 2nd, 3rd and 4th Hep B Satisfy[2] (% eligible)
**Year**							
	2017/18	7,755	1,215 (15.7)	1,904 (24.6)	1,904 (24.6)	1,473 (19.0)	563 (7.3)
	2018/19	8,168	1,264 (15.5)	3,247 (39.8)	2,471 (30.3)	1,972 (24.1)	784 (9.6)
	2019/20	10,207	2,038 (20.0)	5,117 (50.1)	4,176 (40.9)	3,387 (33.2)	1,493 (14.6)
**Prison**							
2017/18	Prison 1	710	76 (10.7)	157 (22.1)	157 (22.1)	127 (17.9)	54 (7.6)
	Prison 2	1,188	478 (40.2)	194 (16.3)	194 (16.3)	138 (11.6)	30 (2.5)
	Prison 3	970	175 (18.0)	279 (28.8)	279 (28.8)	224 (23.1)	115 (11.9)
	Prison 4	684	78 (11.4)	208 (30.4)	208 (30.4)	173 (25.3)	77 (11.3)
	Prison 5	73	0 (0.0)	24 (32.9)	24 (32.9)	22 (30.1)	<10 [6]
	Prison 6	592	43 (7.3)	185 (31.3)	185 (31.3)	156 (26.4)	46 (7.8)
	Prison 7	286	41 (14.3)	121 (42.3)	121 (42.3)	108 (37.8)	51 (17.8)
	Prison 8	382	71 (18.6)	130 (34.0)	130 (34.0)	94 (24.6)	30 (7.9)
	Prison 9	65	<10 [6]	19 (29.2)	19 (29.2)	14 (21.5)	<10 [6]
	Prison 10	1,322	<10 [6]	251 (19.0)	251 (19.0)	167 (12.6)	60 (4.5)
	Prison 11	1,062	144 (13.6)	231 (21.8)	231 (21.8)	165 (15.5)	66 (6.2)
	Prison 12	313	54 (17.3)	80 (25.6)	80 (25.6)	62 (19.8)	13 (4.2)
	Prison 13	108	52 (48.1)	25 (23.1)	25 (23.1)	23 (21.3)	<10 [6]
2018/19	Prison 1	708	59 (8.3)	234 (33.1)	184 (26.0)	154 (21.8)	64 (9.0)
	Prison 2	988	472 (47.8)	286 (28.9)	204 (20.6)	149 (15.1)	52 (5.3)
	Prison 3	940	93 (9.9)	401 (42.7)	323 (34.4)	257 (27.3)	144 (15.3)
	Prison 4	857	145 (16.9)	388 (45.3)	318 (37.1)	277 (32.3)	121 (14.1)
	Prison 5	47	7 (14.9)	20 (42.6)	18 (38.3)	12 (25.5)	<10 [6]
	Prison 6	471	75 (15.9)	194 (41.2)	145 (30.8)	132 (28.0)	41 (8.7)
	Prison 7	311	39 (12.5)	205 (65.9)	176 (56.6)	150 (48.2)	67 (21.5)
	Prison 8	457	102 (22.3)	238 (52.1)	177 (38.7)	137 (30.0)	49 (10.7)
	Prison 9	81	<10 [6]	44 (54.3)	35 (43.2)	29 (35.8)	17 (21.0)
	Prison 10	1,451	<10 [6]	536 (36.9)	328 (22.6)	232 (16.0)	107 (7.4)
	Prison 11	1,449	206 (14.2)	498 (34.4)	381 (26.3)	279 (19.3)	86 (5.9)
	Prison 12	321	52 (16.2)	177 (55.1)	161 (50.2)	145 (45.2)	23 (7.2)
	Prison 13	87	13 (14.9)	26 (29.9)	21 (24.1)	19 (21.8)	<10 [6]
2019/20	Prison 1	715	77 (10.8)	408 (57.1)	363 (50.8)	315 (44.1)	132 (18.5)
	Prison 2	1,161	503 (43.3)	374 (32.2)	282 (24.3)	210 (18.1)	68 (5.9)
	Prison 3	995	193 (19.4)	508 (51.1)	434 (43.6)	365 (36.7)	216 (21.7)
	Prison 4	969	190 (19.6)	555 (57.3)	471 (48.6)	401 (41.4)	204 (21.1)
	Prison 5	77	10 (13.0)	39 (50.6)	35 (45.5)	31 (40.3)	12 (15.6)
	Prison 6	477	65 (13.6)	260 (54.5)	224 (47.0)	191 (40.0)	80 (16.8)
	Prison 7	481	31 (6.4)	379 (78.8)	335 (69.6)	284 (59.0)	139 (28.9)
	Prison 8	652	152 (23.3)	371 (56.9)	276 (42.3)	218 (33.4)	103 (15.8)
	Prison 9	137	<10 [6]	77 (56.2)	59 (43.1)	50 (36.5)	34 (24.8)
	Prison 10	1,912	371 (19.4)	1,136 (59.4)	913 (47.8)	716 (37.4)	265 (13.9)
	Prison 11	2,275	407 (17.9)	781 (34.3)	579 (25.5)	422 (18.5)	175 (7.7)
	Prison 12	228	22 (9.6)	163 (71.5)	153 (67.1)	145 (63.6)	47 (20.6)
	Prison 13	128	10 (7.8)	66 (51.6)	52 (40.6)	39 (30.5)	18 (14.1)
**Prison category**							
2017/18	A	173	55 (31.8)	44 (25.4)	44 (25.4)	37 (21.4)	14 (8.1)
	B	3,572	622 (17.4)	676 (18.9)	676 (18.9)	470 (13.2)	156 (4.4)
	C	2,364	329 (13.9)	644 (27.2)	644 (27.2)	524 (22.2)	246 (10.4)
	Closed	668	112 (16.8)	251 (37.6)	251 (37.6)	202 (30.2)	81 (12.1)
	YOI	313	54 (17.3)	80 (25.6)	80 (25.6)	62 (19.8)	13 (4.2)
2018/19	A	168	14 (8.3)	70 (41.7)	56 (33.3)	48 (28.6)	25 (14.9)
	B	3,888	678 (17.4)	1,320 (34.0)	913 (23.5)	660 (17.0)	245 (6.3)
	C	2,505	297 (11.9)	1,023 (40.8)	825 (32.9)	688 (27.5)	329 (13.1)
	Closed	768	141 (18.4)	443 (57.7)	353 (46.0)	287 (37.4)	116 (15.1)
	YOI	321	52 (16.2)	177 (55.1)	161 (50.2)	145 (45.2)	23 (7.2)
2019/20	A	265	17 (6.4)	143 (54.0)	111 (41.9)	89 (33.6)	52 (19.6)
	B	5,348	1,281 (24.0)	2,291 (42.8)	1,774 (33.2)	1,348 (25.2)	508 (9.5)
	C	2,679	460 (17.2)	1,471 (54.9)	1,268 (47.3)	1,081 (40.4)	552 (20.6)
	Closed	1,133	183 (16.2)	750 (66.2)	611 (53.9)	502 (44.3)	242 (21.4)
	D	554	75 (13.5)	299 (54.0)	259 (46.8)	222 (40.1)	92 (16.6)
	YOI	228	22 (9.6)	163 (71.5)	153 (67.1)	145 (63.6)	47 (20.6)
**Gender**							
2017/18	F	667	112 (16.8)	250 (37.5)	250 (37.5)	202 (30.3)	81 (12.1)
	M	7,088	1,103 (15.6)	1,654 (23.3)	1,654 (23.3)	1,271 (17.9)	482 (6.8)
2018/19	F	768	141 (18.4)	443 (57.7)	353 (46.0)	287 (37.4)	116 (15.1)
	M	7,399	1,123 (15.2)	2,804 (37.9)	2,118 (28.6)	1,685 (22.8)	668 (9.0)
2019/20	F	651	152 (23.3)	371 (57.0)	276 (42.4)	218 (33.5)	103 (15.8)
	M	9,075	1,855 (20.4)	4,367 (48.1)	3,566 (39.3)	2,886 (31.8)	1,251 (13.8)

[1]Eligible for indicator; [2]Achieve in current prison; [3]Achieve in previous prison; [4]Overall achievement - either current or previous prison; [5]Declined indicator; [6]Suppressed (<10) to avoid disclosure

Indicator *The proportion of the prison population who have received one, two, three or four (course of three plus booster) Hepatitis B vaccinations in the preceding 12 months*
Group: Communicable Disease

Community achievement 2019/20 (%): NA

Patients resident in the study prison in the last 12 months with a clinical code added at any time indicating a history of any illicit drug use

	Variable	Eligible[1]	Declined (%)[5]	1st Hep B Satisfy[2] (% eligible)	1st and 2nd Hep B Satisfy[2] (% eligible)	1st, 2nd and 3rd Hep B Satisfy[2] (% eligible)	1st, 2nd, 3rd and 4th Hep B Satisfy[2] (% eligible)
**Sentence Status**							
2017/18	.	<10 [6]	<10 [6]	<10 [6]	<10 [6]	<10 [6]	<10 [6]
	Absconded	<10 [6]	<10 [6]	<10 [6]	<10 [6]	<10 [6]	<10 [6]
	Active In	5,671	730 (12.9)	1,481 (26.1)	1,481 (26.1)	1,165 (20.5)	473 (8.3)
	Active Out	371	42 (11.3)	87 (23.5)	87 (23.5)	60 (16.2)	19 (5.1)
	Convicted Sentence	804	234 (29.1)	150 (18.7)	150 (18.7)	100 (12.4)	31 (3.9)
	Downgrade in security category	<10 [6]	<10 [6]	<10 [6]	<10 [6]	<10 [6]	<10 [6]
	Internal Cell Move	<10 [6]	<10 [6]	<10 [6]	<10 [6]	<10 [6]	<10 [6]
	Judges Remand	32	<10 [6]	<10 [6]	<10 [6]	<10 [6]	<10 [6]
	Licence Revoke	29	14 (48.3)	<10 [6]	<10 [6]	<10 [6]	<10 [6]
	On Remand	507	172 (33.9)	89 (17.6)	89 (17.6)	69 (13.6)	16 (3.2)
	Transfer	340	23 (6.8)	90 (26.5)	90 (26.5)	73 (21.5)	22 (6.5)
	Upgrade in security category	<10 [6]	<10 [6]	<10 [6]	<10 [6]	<10 [6]	<10 [6]
2018/19	.	<10 [6]	<10 [6]	<10 [6]	<10 [6]	<10 [6]	<10 [6]
	Absconded	<10 [6]	<10 [6]	<10 [6]	<10 [6]	<10 [6]	<10 [6]
	Active In	6,753	786 (11.6)	2,793 (41.4)	2,141 (31.7)	1,733 (25.7)	699 (10.4)
	Active Out	302	24 (7.9)	98 (32.5)	72 (23.8)	49 (16.2)	22 (7.3)
	Convicted Sentence	518	221 (42.7)	176 (34.0)	130 (25.1)	104 (20.1)	42 (8.1)
	Downgrade in security category	<10 [6]	<10 [6]	<10 [6]	<10 [6]	<10 [6]	<10 [6]
	Internal Cell Move	<10 [6]	<10 [6]	<10 [6]	<10 [6]	<10 [6]	<10 [6]
	Judges Remand	<10 [6]	<10 [6]	<10 [6]	<10 [6]	<10 [6]	<10 [6]
	Licence Revoke	60	23 (38.3)	19 (31.7)	14 (23.3)	<10 [6]	<10 [6]
	On Remand	374	179 (47.9)	103 (27.5)	72 (19.3)	45 (12.0)	15 (4.0)
	Transfer	151	27 (17.9)	55 (36.4)	41 (27.2)	31 (20.5)	<10 [6]
	Upgrade in security category	<10 [6]	<10 [6]	<10 [6]	<10 [6]	<10 [6]	<10 [6]
2019/20	.	<10 [6]	<10 [6]	<10 [6]	<10 [6]	<10 [6]	<10 [6]
	Absconded	<10 [6]	<10 [6]	<10 [6]	<10 [6]	<10 [6]	<10 [6]
	Active In	8,900	1,548 (17.4)	4,652 (52.3)	3,812 (42.8)	3,113 (35.0)	1,400 (15.7)
	Active Out	223	47 (21.1)	105 (47.1)	88 (39.5)	66 (29.6)	24 (10.8)
	Convicted Sentence	594	257 (43.3)	189 (31.8)	148 (24.9)	112 (18.9)	43 (7.2)
	Downgrade in security category	<10 [6]	<10 [6]	<10 [6]	<10 [6]	<10 [6]	<10 [6]
	Internal Cell Move	<10 [6]	<10 [6]	<10 [6]	<10 [6]	<10 [6]	<10 [6]
	Judges Remand	10	<10 [6]	<10 [6]	<10 [6]	<10 [6]	<10 [6]
	Licence Revoke	88	27 (30.7)	37 (42.0)	26 (29.5)	17 (19.3)	<10 [6]
	On Remand	374	155 (41.4)	127 (34.0)	99 (26.5)	77 (20.6)	23 (6.1)
	Transfer	13	<10 [6]	<10 [6]	<10 [6]	<10 [6]	<10 [6]
	Upgrade in security category	<10 [6]	<10 [6]	<10 [6]	<10 [6]	<10 [6]	<10 [6]
**Age - years**							
2017/18	10 - <20	213	39 (18.3)	25 (11.7)	25 (11.7)	19 (8.9)	<10 [6]
	20 - <30	2,800	429 (15.3)	723 (25.8)	723 (25.8)	563 (20.1)	209 (7.5)
	30 - <40	2,735	407 (14.9)	675 (24.7)	675 (24.7)	517 (18.9)	205 (7.5)
	40 - <50	1,412	231 (16.4)	338 (23.9)	338 (23.9)	260 (18.4)	102 (7.2)
	50 - <60	479	78 (16.3)	124 (25.9)	124 (25.9)	100 (20.9)	35 (7.3)
	60 - <70	92	25 (27.2)	13 (14.1)	13 (14.1)	<10 [6]	<10 [6]
	70 - <80	16	<10 [6]	<10 [6]	<10 [6]	<10 [6]	<10 [6]
	80 - <90	<10 [6]	<10 [6]	<10 [6]	<10 [6]	<10 [6]	<10 [6]
	90 - <100	<10 [6]	<10 [6]	<10 [6]	<10 [6]	<10 [6]	<10 [6]
	100 - <110	<10 [6]	<10 [6]	<10 [6]	<10 [6]	<10 [6]	<10 [6]
2018/19	10 - <20	203	32 (15.8)	94 (46.3)	70 (34.5)	61 (30.0)	<10 [6]
	20 - <30	2,892	448 (15.5)	1,163 (40.2)	910 (31.5)	728 (25.2)	288 (10.0)
	30 - <40	3,095	439 (14.2)	1,256 (40.6)	938 (30.3)	740 (23.9)	298 (9.6)
	40 - <50	1,495	262 (17.5)	556 (37.2)	420 (28.1)	334 (22.3)	146 (9.8)
	50 - <60	405	66 (16.3)	157 (38.8)	117 (28.9)	98 (24.2)	40 (9.9)
	60 - <70	72	16 (22.2)	18 (25.0)	13 (18.1)	10 (13.9)	<10 [6]
	70 - <80	<10 [6]	<10 [6]	<10 [6]	<10 [6]	<10 [6]	<10 [6]
	80 - <90	<10 [6]	<10 [6]	<10 [6]	<10 [6]	<10 [6]	<10 [6]
	90 - <100	<10 [6]	<10 [6]	<10 [6]	<10 [6]	<10 [6]	<10 [6]
	100 - <110	<10 [6]	<10 [6]	<10 [6]	<10 [6]	<10 [6]	<10 [6]
2019/20	10 - <20	155	31 (20.0)	94 (60.6)	80 (51.6)	69 (44.5)	12 (7.7)
	20 - <30	3,280	578 (17.6)	1,646 (50.2)	1,371 (41.8)	1,098 (33.5)	464 (14.1)
	30 - <40	4,078	810 (19.9)	2,072 (50.8)	1,693 (41.5)	1,387 (34.0)	668 (16.4)
	40 - <50	2,076	447 (21.5)	1,028 (49.5)	808 (38.9)	649 (31.3)	277 (13.3)
	50 - <60	550	148 (26.9)	251 (45.6)	204 (37.1)	167 (30.4)	64 (11.6)
	60 - <70	60	20 (33.3)	24 (40.0)	19 (31.7)	16 (26.7)	<10 [6]
	70 - <80	<10 [6]	<10 [6]	<10 [6]	<10 [6]	<10 [6]	<10 [6]
	80 - <90	<10 [6]	<10 [6]	<10 [6]	<10 [6]	<10 [6]	<10 [6]
	90 - <100	<10 [6]	<10 [6]	<10 [6]	<10 [6]	<10 [6]	<10 [6]
	100 - <110	<10 [6]	<10 [6]	<10 [6]	<10 [6]	<10 [6]	<10 [6]

[1]Eligible for indicator; [2]Achieve in current prison; [3]Achieve in previous prison; [4]Overall achievement - either current or previous prison; [5]Declined indicator; [6]Suppressed (<10) to avoid disclosure

Indicator *The proportion of the prison population who have received one, two, three or four (course of three plus booster) Hepatitis B vaccinations in the preceding 12 months*

Group: Communicable Disease

Community achievement 2019/20 (%): NA

Patients resident in the study prison in the last 12 months with a clinical code added at any time indicating a history of any illicit drug use

	Variable	Eligible[1]	Declined (%)[5]	1st Hep B Satisfy[2] (% eligible)	1st and 2nd Hep B Satisfy[2] (% eligible)	1st, 2nd and 3rd Hep B Satisfy[2] (% eligible)	1st, 2nd, 3rd and 4th Hep B Satisfy[2] (% eligible)
**Length of Stay (months)**							
2017/18	<1	1,984	329 (16.6)	376 (19.0)	376 (19.0)	273 (13.8)	104 (5.2)
	1-<6	3,475	570 (16.4)	830 (23.9)	830 (23.9)	638 (18.4)	221 (6.4)
	6-<12	1,206	166 (13.8)	323 (26.8)	323 (26.8)	248 (20.6)	91 (7.5)
	12-<24	731	101 (13.8)	245 (33.5)	245 (33.5)	202 (27.6)	90 (12.3)
	24+	359	49 (13.6)	130 (36.2)	130 (36.2)	112 (31.2)	57 (15.9)
2018/19	<1	2,232	406 (18.2)	734 (32.9)	510 (22.8)	361 (16.2)	123 (5.5)
	1-<6	3,622	565 (15.6)	1,375 (38.0)	1,018 (28.1)	789 (21.8)	306 (8.4)
	6-<12	1,217	175 (14.4)	508 (41.7)	399 (32.8)	340 (27.9)	128 (10.5)
	12-<24	1,097	118 (10.8)	630 (57.4)	544 (49.6)	482 (43.9)	227 (20.7)
	24+	<10 [6]	<10 [6]	<10 [6]	<10 [6]	<10 [6]	<10 [6]
2019/20	<1	3,467	738 (21.3)	1,445 (41.7)	1,077 (31.1)	791 (22.8)	375 (10.8)
	1-<6	5,427	1,095 (20.2)	2,891 (53.3)	2,418 (44.6)	1,982 (36.5)	845 (15.6)
	6-<12	1,301	202 (15.5)	774 (59.5)	674 (51.8)	607 (46.7)	272 (20.9)
	12-<24	12	<10 [6]	<10 [6]	<10 [6]	<10 [6]	<10 [6]
	24+	<10 [6]	<10 [6]	<10 [6]	<10 [6]	<10 [6]	<10 [6]
**Ethnic Group**							
2017/18	White	5,797	896 (15.5)	1,616 (27.9)	1,616 (27.9)	1,268 (21.9)	506 (8.7)
	Mixed	129	23 (17.8)	34 (26.4)	34 (26.4)	23 (17.8)	<10 [6]
	Asian or Asian British	224	30 (13.4)	65 (29.0)	65 (29.0)	47 (21.0)	16 (7.1)
	Black or Black British	119	24 (20.2)	19 (16.0)	19 (16.0)	16 (13.4)	<10 [6]
	Chinese and Other	43	11 (25.6)	12 (27.9)	12 (27.9)	<10 [6]	<10 [6]
	Unclassified	113	24 (21.2)	25 (22.1)	25 (22.1)	19 (16.8)	<10 [6]
2018/19	White	5,696	977 (17.2)	2,599 (45.6)	1,997 (35.1)	1,603 (28.1)	683 (12.0)
	Mixed	110	19 (17.3)	45 (40.9)	37 (33.6)	30 (27.3)	15 (13.6)
	Asian or Asian British	173	21 (12.1)	84 (48.6)	65 (37.6)	55 (31.8)	22 (12.7)
	Black or Black British	84	23 (27.4)	35 (41.7)	26 (31.0)	23 (27.4)	<10 [6]
	Chinese and Other	44	10 (22.7)	19 (43.2)	14 (31.8)	13 (29.5)	<10 [6]
	Unclassified	143	15 (10.5)	40 (28.0)	34 (23.8)	25 (17.5)	<10 [6]
2019/20	White	7,158	1,513 (21.1)	3,990 (55.7)	3,294 (46.0)	2,713 (37.9)	1,318 (18.4)
	Mixed	122	25 (20.5)	70 (57.4)	59 (48.4)	48 (39.3)	27 (22.1)
	Asian or Asian British	182	30 (16.5)	110 (60.4)	97 (53.3)	82 (45.1)	40 (22.0)
	Black or Black British	124	29 (23.4)	66 (53.2)	57 (46.0)	49 (39.5)	14 (11.3)
	Chinese and Other	34	<10 [6]	13 (38.2)	12 (35.3)	10 (29.4)	<10 [6]
	Unclassified	125	18 (14.4)	61 (48.8)	50 (40.0)	40 (32.0)	11 (8.8)

[1]Eligible for indicator; [2]Achieve in current prison; [3]Achieve in previous prison; [4]Overall achievement - either current or previous prison; [5]Declined indicator; [6]Suppressed (<10) to avoid disclosure

Indicator *The proportion of people with one or more of coronary heart disease, stroke or transient ischemic attack, diabetes or chronic obstructive pulmonary disease who have received a flu vaccination between August and March*

Group: Communicable Disease

Community achievement 2019/20: CHD: 77.33%, Stroke/TIA: 74.22%, Diabetes: 72.22%, COPD 77.44% (QOF 19/20)

	Variable	Population	Eligible[1] (% popln)	Declined[5] (% eligible)	Satisfy[2] (% eligible)	Elsewhere[3] (% eligible)	Achieve[4] (% eligible)
**Year**							
	2017/18	21,677	1,300 (6.0)	179 (13.8)	570 (43.8)	4 (0.3)	574 (44.2)
	2018/19	22,099	1,534 (6.9)	237 (15.4)	613 (40.0)	9 (0.6)	622 (40.5)
	2019/20	25,811	1,752 (6.8)	252 (14.4)	786 (44.9)	1 (0.1)	787 (44.9)
**Prison**							
2017/18	Prison 1	1,323	53 (4.0)	2 (3.8)	17 (32.1)	0 (0.0)	17 (32.1)
	Prison 2	3,261	198 (6.1)	33 (16.7)	62 (31.3)	0 (0.0)	62 (31.3)
	Prison 3	2,623	134 (5.1)	2 (1.5)	87 (64.9)	0 (0.0)	87 (64.9)
	Prison 4	2,089	147 (7.0)	25 (17.0)	78 (53.1)	0 (0.0)	78 (53.1)
	Prison 5	637	38 (6.0)	10 (26.3)	18 (47.4)	0 (0.0)	18 (47.4)
	Prison 6	1,552	92 (5.9)	4 (4.3)	48 (52.2)	4 (4.3)	52 (56.5)
	Prison 7	635	48 (7.6)	14 (29.2)	22 (45.8)	0 (0.0)	22 (45.8)
	Prison 8	1,085	81 (7.5)	27 (33.3)	29 (35.8)	0 (0.0)	29 (35.8)
	Prison 9	981	150 (15.3)	36 (24.0)	87 (58.0)	0 (0.0)	87 (58.0)
	Prison 10	2,523	115 (4.6)	0 (0.0)	32 (27.8)	0 (0.0)	32 (27.8)
	Prison 11	3,470	158 (4.6)	7 (4.4)	33 (20.9)	0 (0.0)	33 (20.9)
	Prison 12	815	4 (0.5)	1 (25.0)	0 (0.0)	0 (0.0)	0 (0.0)
	Prison 13	683	82 (12.0)	18 (22.0)	57 (69.5)	0 (0.0)	57 (69.5)
2018/19	Prison 1	1,333	49 (3.7)	4 (8.2)	18 (36.7)	2 (4.1)	20 (40.8)
	Prison 2	2,705	156 (5.8)	33 (21.2)	47 (30.1)	0 (0.0)	47 (30.1)
	Prison 3	2,522	159 (6.3)	3 (1.9)	90 (56.6)	0 (0.0)	90 (56.6)
	Prison 4	2,349	178 (7.6)	21 (11.8)	93 (52.2)	0 (0.0)	93 (52.2)
	Prison 5	676	45 (6.7)	7 (15.6)	0 (0.0)	7 (15.6)	7 (15.6)
	Prison 6	1,513	95 (6.3)	15 (15.8)	45 (47.4)	0 (0.0)	45 (47.4)
	Prison 7	654	68 (10.4)	15 (22.1)	32 (47.1)	0 (0.0)	32 (47.1)
	Prison 8	1,148	129 (11.2)	30 (23.3)	48 (37.2)	0 (0.0)	48 (37.2)
	Prison 9	996	175 (17.6)	65 (37.1)	89 (50.9)	0 (0.0)	89 (50.9)
	Prison 10	2,717	171 (6.3)	12 (7.0)	49 (28.7)	0 (0.0)	49 (28.7)
	Prison 11	4,020	222 (5.5)	13 (5.9)	48 (21.6)	0 (0.0)	48 (21.6)
	Prison 12	792	5 (0.6)	1 (20.0)	1 (20.0)	0 (0.0)	1 (20.0)
	Prison 13	674	82 (12.2)	18 (22.0)	53 (64.6)	0 (0.0)	53 (64.6)
2019/20	Prison 1	1,410	75 (5.3)	5 (6.7)	37 (49.3)	0 (0.0)	37 (49.3)
	Prison 2	2,979	174 (5.8)	27 (15.5)	76 (43.7)	0 (0.0)	76 (43.7)
	Prison 3	2,809	166 (5.9)	7 (4.2)	108 (65.1)	0 (0.0)	108 (65.1)
	Prison 4	2,651	154 (5.8)	28 (18.2)	84 (54.5)	0 (0.0)	84 (54.5)
	Prison 5	616	42 (6.8)	5 (11.9)	26 (61.9)	0 (0.0)	26 (61.9)
	Prison 6	1,533	100 (6.5)	14 (14.0)	46 (46.0)	0 (0.0)	46 (46.0)
	Prison 7	860	100 (11.6)	12 (12.0)	43 (43.0)	0 (0.0)	43 (43.0)
	Prison 8	1,385	165 (11.9)	55 (33.3)	54 (32.7)	0 (0.0)	54 (32.7)
	Prison 9	1,092	208 (19.0)	46 (22.1)	127 (61.1)	1 (0.5)	128 (61.5)
	Prison 10	3,577	202 (5.6)	17 (8.4)	64 (31.7)	0 (0.0)	64 (31.7)
	Prison 11	5,348	267 (5.0)	17 (6.4)	59 (22.1)	0 (0.0)	59 (22.1)
	Prison 12	805	4 (0.5)	1 (25.0)	1 (25.0)	0 (0.0)	1 (25.0)
	Prison 13	746	95 (12.7)	18 (18.9)	61 (64.2)	0 (0.0)	61 (64.2)
**Prison category**							
2017/18	A	1,664	232 (13.9)	54 (23.3)	144 (62.1)	0 (0.0)	144 (62.1)
	B	9,254	471 (5.1)	40 (8.5)	127 (27.0)	0 (0.0)	127 (27.0)
	C	6,035	334 (5.5)	29 (8.7)	182 (54.5)	0 (0.0)	182 (54.5)
	Closed	1,720	129 (7.5)	41 (31.8)	51 (39.5)	0 (0.0)	51 (39.5)
	D	2,189	130 (5.9)	14 (10.8)	66 (50.8)	4 (3.1)	70 (53.8)
	YOI	815	4 (0.5)	1 (25.0)	0 (0.0)	0 (0.0)	0 (0.0)
2018/19	A	1,670	257 (15.4)	83 (32.3)	142 (55.3)	0 (0.0)	142 (55.3)
	B	9,442	549 (5.8)	58 (10.6)	144 (26.2)	0 (0.0)	144 (26.2)
	C	6,204	386 (6.2)	28 (7.3)	201 (52.1)	2 (0.5)	203 (52.6)
	Closed	1,802	197 (10.9)	45 (22.8)	80 (40.6)	0 (0.0)	80 (40.6)
	D	2,189	140 (6.4)	22 (15.7)	45 (32.1)	7 (5.0)	52 (37.1)
	YOI	792	5 (0.6)	1 (20.0)	1 (20.0)	0 (0.0)	1 (20.0)
2019/20	A	1,838	303 (16.5)	64 (21.1)	188 (62.0)	1 (0.3)	189 (62.4)
	B	11,904	643 (5.4)	61 (9.5)	199 (30.9)	0 (0.0)	199 (30.9)
	C	6,870	395 (5.7)	40 (10.1)	229 (58.0)	0 (0.0)	229 (58.0)
	Closed	2,245	265 (11.8)	67 (25.3)	97 (36.6)	0 (0.0)	97 (36.6)
	D	2,149	142 (6.6)	19 (13.4)	72 (50.7)	0 (0.0)	72 (50.7)
	YOI	805	4 (0.5)	1 (25.0)	1 (25.0)	0 (0.0)	1 (25.0)
**Gender**							
2017/18	F	1,699	129 (7.6)	41 (31.8)	51 (39.5)	0 (0.0)	51 (39.5)
	M	19,977	1,171 (5.9)	138 (11.8)	519 (44.3)	4 (0.3)	523 (44.7)
2018/19	F	1,802	196 (10.9)	45 (23.0)	80 (40.8)	0 (0.0)	80 (40.8)
	M	20,295	1,338 (6.6)	192 (14.3)	533 (39.8)	9 (0.7)	542 (40.5)
2019/20	F	1,376	165 (12.0)	55 (33.3)	54 (32.7)	0 (0.0)	54 (32.7)
	M	23,570	1,487 (6.3)	185 (12.4)	689 (46.3)	1 (0.1)	690 (46.4)

[1]Eligible for indicator; [2]Achieve in current prison; [3]Achieve in previous prison; [4]Overall achievement - either current or previous prison; [5]Declined indicator; [6]Suppressed (<10) to avoid disclosure

Indicator	The proportion of people with one or more of coronary heart disease, stroke or transient ischemic attack, diabetes or chronic obstructive pulmonary disease who have received a flu vaccination between August and March
	Group: Communicable Disease

Community achievement 2019/20: CHD: 77.33%, Stroke/TIA: 74.22%, Diabetes: 72.22%, COPD 77.44% (QOF 19/20)

	Variable	Population	Eligible[1] (% popln)	Declined[5] (% eligible)	Satisfy[2] (% eligible)	Elsewhere[3] (% eligible)	Achieve[4] (% eligible)
**Sentence Status**							
2017/18	.	81	<10 [6]	<10 [6]	<10 [6]	<10 [6]	<10 [6]
	Absconded	<10 [6]	<10 [6]	<10 [6]	<10 [6]	<10 [6]	<10 [6]
	Active In	15,872	964 (6.1)	138 (14.3)	461 (47.8)	<10 [6]	462 (47.9)
	Active Out	1,052	62 (5.9)	<10 [6]	23 (37.1)	<10 [6]	24 (38.7)
	Convicted Sentence	2,125	125 (5.9)	12 (9.6)	35 (28.0)	<10 [6]	35 (28.0)
	Downgrade in security category	<10 [6]	<10 [6]	<10 [6]	<10 [6]	<10 [6]	<10 [6]
	Internal Cell Move	<10 [6]	<10 [6]	<10 [6]	<10 [6]	<10 [6]	<10 [6]
	Judges Remand	92	<10 [6]	<10 [6]	<10 [6]	<10 [6]	<10 [6]
	Licence Revoke	52	<10 [6]	<10 [6]	<10 [6]	<10 [6]	<10 [6]
	On Remand	1,492	82 (5.5)	18 (22.0)	25 (30.5)	<10 [6]	25 (30.5)
	Transfer	910	50 (5.5)	<10 [6]	24 (48.0)	<10 [6]	26 (52.0)
	Upgrade in security category	<10 [6]	<10 [6]	<10 [6]	<10 [6]	<10 [6]	<10 [6]
2018/19	.	88	<10 [6]	<10 [6]	<10 [6]	<10 [6]	<10 [6]
	Absconded	<10 [6]	<10 [6]	<10 [6]	<10 [6]	<10 [6]	<10 [6]
	Active In	18,145	1,277 (7.0)	187 (14.6)	542 (42.4)	<10 [6]	550 (43.1)
	Active Out	835	70 (8.4)	11 (15.7)	18 (25.7)	<10 [6]	19 (27.1)
	Convicted Sentence	1,320	80 (6.1)	18 (22.5)	23 (28.8)	<10 [6]	23 (28.8)
	Downgrade in security category	<10 [6]	<10 [6]	<10 [6]	<10 [6]	<10 [6]	<10 [6]
	Internal Cell Move	<10 [6]	<10 [6]	<10 [6]	<10 [6]	<10 [6]	<10 [6]
	Judges Remand	<10 [6]	<10 [6]	<10 [6]	<10 [6]	<10 [6]	<10 [6]
	Licence Revoke	125	<10 [6]	<10 [6]	<10 [6]	<10 [6]	<10 [6]
	On Remand	1,059	65 (6.1)	16 (24.6)	19 (29.2)	<10 [6]	19 (29.2)
	Transfer	518	32 (6.2)	<10 [6]	<10 [6]	<10 [6]	<10 [6]
	Upgrade in security category	<10 [6]	<10 [6]	<10 [6]	<10 [6]	<10 [6]	<10 [6]
2019/20	.	69	<10 [6]	<10 [6]	<10 [6]	<10 [6]	<10 [6]
	Absconded	<10 [6]	<10 [6]	<10 [6]	<10 [6]	<10 [6]	<10 [6]
	Active In	22,424	1,533 (6.8)	225 (14.7)	708 (46.2)	<10 [6]	709 (46.2)
	Active Out	625	54 (8.6)	<10 [6]	12 (22.2)	<10 [6]	12 (22.2)
	Convicted Sentence	1,361	81 (6.0)	14 (17.3)	34 (42.0)	<10 [6]	34 (42.0)
	Downgrade in security category	<10 [6]	<10 [6]	<10 [6]	<10 [6]	<10 [6]	<10 [6]
	Internal Cell Move	<10 [6]	<10 [6]	<10 [6]	<10 [6]	<10 [6]	<10 [6]
	Judges Remand	19	<10 [6]	<10 [6]	<10 [6]	<10 [6]	<10 [6]
	Licence Revoke	178	13 (7.3)	<10 [6]	<10 [6]	<10 [6]	<10 [6]
	On Remand	1,031	61 (5.9)	<10 [6]	27 (44.3)	<10 [6]	27 (44.3)
	Transfer	101	<10 [6]	<10 [6]	<10 [6]	<10 [6]	<10 [6]
	Upgrade in security category	<10 [6]	<10 [6]	<10 [6]	<10 [6]	<10 [6]	<10 [6]
**Age - years**							
2017/18	10 - <20	468	<10 [6]	<10 [6]	<10 [6]	<10 [6]	<10 [6]
	20 - <30	6,994	49 (0.7)	<10 [6]	15 (30.6)	<10 [6]	15 (30.6)
	30 - <40	7,051	133 (1.9)	18 (13.5)	49 (36.8)	<10 [6]	49 (36.8)
	40 - <50	4,114	317 (7.7)	42 (13.2)	123 (38.8)	<10 [6]	123 (38.8)
	50 - <60	2,107	419 (19.9)	59 (14.1)	183 (43.7)	<10 [6]	185 (44.2)
	60 - <70	684	257 (37.6)	38 (14.8)	133 (51.8)	<10 [6]	134 (52.1)
	70 - <80	213	100 (46.9)	14 (14.0)	54 (54.0)	<10 [6]	55 (55.0)
	80 - <90	40	21 (52.5)	<10 [6]	13 (61.9)	<10 [6]	13 (61.9)
	90 - <100	<10 [6]	<10 [6]	<10 [6]	<10 [6]	<10 [6]	<10 [6]
	100 - <110	<10 [6]	<10 [6]	<10 [6]	<10 [6]	<10 [6]	<10 [6]
2018/19	10 - <20	436	<10 [6]	<10 [6]	<10 [6]	<10 [6]	<10 [6]
	20 - <30	7,163	70 (1.0)	<10 [6]	19 (27.1)	<10 [6]	19 (27.1)
	30 - <40	7,381	200 (2.7)	23 (11.5)	71 (35.5)	<10 [6]	74 (37.0)
	40 - <50	4,180	379 (9.1)	63 (16.6)	130 (34.3)	<10 [6]	133 (35.1)
	50 - <60	1,978	468 (23.7)	82 (17.5)	186 (39.7)	<10 [6]	188 (40.2)
	60 - <70	701	286 (40.8)	43 (15.0)	137 (47.9)	<10 [6]	137 (47.9)
	70 - <80	209	101 (48.3)	13 (12.9)	55 (54.5)	<10 [6]	56 (55.4)
	80 - <90	45	28 (62.2)	<10 [6]	14 (50.0)	<10 [6]	14 (50.0)
	90 - <100	<10 [6]	<10 [6]	<10 [6]	<10 [6]	<10 [6]	<10 [6]
	100 - <110	<10 [6]	<10 [6]	<10 [6]	<10 [6]	<10 [6]	<10 [6]
2019/20	10 - <20	404	<10 [6]	<10 [6]	<10 [6]	<10 [6]	<10 [6]
	20 - <30	8,064	81 (1.0)	<10 [6]	35 (43.2)	<10 [6]	35 (43.2)
	30 - <40	9,125	237 (2.6)	35 (14.8)	86 (36.3)	<10 [6]	86 (36.3)
	40 - <50	4,948	478 (9.7)	81 (16.9)	199 (41.6)	<10 [6]	199 (41.6)
	50 - <60	2,224	500 (22.5)	76 (15.2)	225 (45.0)	<10 [6]	225 (45.0)
	60 - <70	751	308 (41.0)	37 (12.0)	159 (51.6)	<10 [6]	160 (51.9)
	70 - <80	238	116 (48.7)	15 (12.9)	62 (53.4)	<10 [6]	62 (53.4)
	80 - <90	53	30 (56.6)	<10 [6]	19 (63.3)	<10 [6]	19 (63.3)
	90 - <100	<10 [6]	<10 [6]	<10 [6]	<10 [6]	<10 [6]	<10 [6]
	100 - <110	<10 [6]	<10 [6]	<10 [6]	<10 [6]	<10 [6]	<10 [6]

[1]Eligible for indicator; [2]Achieve in current prison; [3]Achieve in previous prison; [4]Overall achievement - either current or previous prison; [5]Declined indicator; [6]Suppressed (<10) to avoid disclosure

Indicator	The proportion of people with one or more of coronary heart disease, stroke or transient ischemic attack, diabetes or chronic obstructive pulmonary disease who have received a flu vaccination between August and March						Community achievement 2019/20: CHD: 77.33%, Stroke/TIA: 74.22%, Diabetes: 72.22%, COPD 77.44% (QOF 19/20)
	**Group: Communicable Disease**						
	**Variable**	**Population**	**Eligible[1] (% popln)**	**Declined[5] (% eligible)**	**Satisfy[2] (% eligible)**	**Elsewhere[3] (% eligible)**	**Achieve[4] (% eligible)**
**Length of Stay (months)**							
2017/18	<1	4,474	173 (3.9)	18 (10.4)	23 (13.3)	<10 [6]	23 (13.3)
	1-<6	8,075	367 (4.5)	38 (10.4)	104 (28.3)	<10 [6]	105 (28.6)
	6-<12	3,672	193 (5.3)	23 (11.9)	80 (41.5)	<10 [6]	82 (42.5)
	12-<24	2,832	180 (6.4)	27 (15.0)	104 (57.8)	<10 [6]	105 (58.3)
	24+	2,624	387 (14.7)	73 (18.9)	259 (66.9)	<10 [6]	259 (66.9)
2018/19	<1	4,801	249 (5.2)	25 (10.0)	40 (16.1)	<10 [6]	40 (16.1)
	1-<6	7,742	435 (5.6)	58 (13.3)	120 (27.6)	<10 [6]	123 (28.3)
	6-<12	3,616	208 (5.8)	27 (13.0)	82 (39.4)	<10 [6]	84 (40.4)
	12-<24	3,447	237 (6.9)	36 (15.2)	118 (49.8)	<10 [6]	122 (51.5)
	24+	2,493	405 (16.2)	91 (22.5)	253 (62.5)	<10 [6]	253 (62.5)
2019/20	<1	5,745	293 (5.1)	34 (11.6)	43 (14.7)	<10 [6]	43 (14.7)
	1-<6	9,697	534 (5.5)	83 (15.5)	172 (32.2)	<10 [6]	172 (32.2)
	6-<12	5,090	327 (6.4)	32 (9.8)	186 (56.9)	<10 [6]	186 (56.9)
	12-<24	3,244	250 (7.7)	35 (14.0)	157 (62.8)	<10 [6]	158 (63.2)
	24+	2,035	348 (17.1)	68 (19.5)	228 (65.5)	<10 [6]	228 (65.5)
**Ethnic Group**							
2017/18	White	15,638	1,075 (6.9)	156 (14.5)	487 (45.3)	<10 [6]	488 (45.4)
	Mixed	431	22 (5.1)	<10 [6]	<10 [6]	<10 [6]	<10 [6]
	Asian or Asian British	813	35 (4.3)	<10 [6]	12 (34.3)	<10 [6]	12 (34.3)
	Black or Black British	404	20 (5.0)	<10 [6]	<10 [6]	<10 [6]	<10 [6]
	Chinese and Other	214	16 (7.5)	<10 [6]	<10 [6]	<10 [6]	<10 [6]
	Unclassified	372	28 (7.5)	<10 [6]	18 (64.3)	<10 [6]	18 (64.3)
2018/19	White	14,911	1,189 (8.0)	189 (15.9)	491 (41.3)	<10 [6]	494 (41.5)
	Mixed	371	25 (6.7)	10 (40.0)	10 (40.0)	<10 [6]	10 (40.0)
	Asian or Asian British	726	42 (5.8)	<10 [6]	15 (35.7)	<10 [6]	16 (38.1)
	Black or Black British	364	22 (6.0)	<10 [6]	<10 [6]	<10 [6]	<10 [6]
	Chinese and Other	167	<10 [6]	<10 [6]	<10 [6]	<10 [6]	<10 [6]
	Unclassified	409	18 (4.4)	<10 [6]	<10 [6]	<10 [6]	<10 [6]
2019/20	White	16,606	1,272 (7.7)	192 (15.1)	592 (46.5)	<10 [6]	593 (46.6)
	Mixed	409	24 (5.9)	<10 [6]	19 (79.2)	<10 [6]	19 (79.2)
	Asian or Asian British	755	69 (9.1)	10 (14.5)	33 (47.8)	<10 [6]	33 (47.8)
	Black or Black British	451	29 (6.4)	<10 [6]	<10 [6]	<10 [6]	<10 [6]
	Chinese and Other	163	10 (6.1)	<10 [6]	<10 [6]	<10 [6]	<10 [6]
	Unclassified	387	21 (5.4)	<10 [6]	12 (57.1)	<10 [6]	12 (57.1)

[1]Eligible for indicator; [2]Achieve in current prison; [3]Achieve in previous prison; [4]Overall achievement - either current or previous prison; [5]Declined indicator; [6]Suppressed (<10) to avoid disclosure

Indicator		The proportion of prison population prescribed opioids in the preceding 8 weeks from 31st March
	Group: Prison specific	

no comparable data in QOF 2019/20 data. Foy et al: 12.8% of all adult patients prescribed an opioid at least once; 6.6% of the sample were prescribed stronger opioids, or 0.85% of adults; For patients prescribed any opioids, the proportion prescribed stronger opioids was 13.7%.

			All prison population			Any mental health diagnosis		All prison population AND NO coded diagnosis of neuropathic pain	
	Variable	Population	Prescribed any opioid in the preceding 8 weeks from 31st March (% popln)	Prescribed any strong opioid in the preceding 8 weeks from 31st March (% popln)	Prescribed benzodiazepines AND any opioid in preceding 8 weeks from 31st March (% popln)	Eligible (% popln)	Prescribed any opioid in the preceding 8 weeks from 31st March (% eligible)	Eligible (% popln)	Prescribed pregabalin OR gabapentin in the preceding 8 weeks from 31st March (% eligible)
**Year**									
	2017/18	21,677	1,834 (8.5)	1,834 (8.5)	1,834 (8.5)	2,466 (11.4)	337 (13.7)	20,772 (95.8)	218 (1.0)
	2018/19	22,099	2,496 (11.3)	2,294 (10.4)	4,882 (22.1)	2,398 (10.9)	435 (18.1)	20,915 (94.6)	190 (0.9)
	2019/20	25,811	2,957 (11.5)	2,257 (8.7)	2,257 (8.7)	4,054 (15.7)	770 (19.0)	24,388 (94.5)	204 (0.8)
**Prison**									
2017/18	Prison 1	1,323	98 (7.4)	98 (7.4)	98 (7.4)	255 (19.3)	23 (9.0)	1,284 (97.1)	23 (1.8)
	Prison 2	3,261	214 (6.6)	214 (6.6)	214 (6.6)	687 (21.1)	92 (13.4)	3,171 (97.2)	29 (0.9)
	Prison 3	2,623	317 (12.1)	317 (12.1)	317 (12.1)	207 (7.9)	49 (23.7)	2,564 (97.8)	<10 [6]
	Prison 4	2,089	329 (15.7)	329 (15.7)	329 (15.7)	78 (3.7)	17 (21.8)	1,944 (93.1)	33 (1.7)
	Prison 5	637	37 (5.8)	37 (5.8)	37 (5.8)	55 (8.6)	<10 [6]	613 (96.2)	13 (2.1)
	Prison 6	1,552	<10 [6]	<10 [6]	<10 [6]	<10 [6]	<10 [6]	1,552 (100.0)	<10 [6]
	Prison 7	635	104 (16.4)	104 (16.4)	104 (16.4)	31 (4.9)	<10 [6]	575 (90.6)	14 (2.4)
	Prison 8	1,085	132 (12.2)	132 (12.2)	132 (12.2)	543 (50.0)	75 (13.8)	1,006 (92.7)	12 (1.2)
	Prison 9	981	157 (16.0)	157 (16.0)	157 (16.0)	60 (6.1)	17 (28.3)	869 (88.6)	22 (2.5)
	Prison 10	2,523	194 (7.7)	194 (7.7)	194 (7.7)	304 (12.0)	36 (11.8)	2,473 (98.0)	29 (1.2)
	Prison 11	3,470	211 (6.1)	211 (6.1)	211 (6.1)	97 (2.8)	10 (10.3)	3,319 (95.6)	26 (0.8)
	Prison 12	815	<10 [6]	<10 [6]	<10 [6]	<10 [6]	<10 [6]	815 (100.0)	<10 [6]
	Prison 13	683	41 (6.0)	41 (6.0)	41 (6.0)	149 (21.8)	13 (8.7)	587 (85.9)	17 (2.9)
2018/19	Prison 1	1,333	111 (8.3)	126 (9.5)	126 (9.5)	247 (18.5)	37 (15.0)	1,286 (96.5)	12 (0.9)
	Prison 2	2,705	223 (8.2)	219 (8.1)	219 (8.1)	761 (28.1)	126 (16.6)	2,608 (96.4)	32 (1.2)
	Prison 3	2,522	457 (18.1)	457 (18.1)	457 (18.1)	185 (7.3)	77 (41.6)	2,420 (96.0)	<10 [6]
	Prison 4	2,349	396 (16.9)	414 (17.6)	414 (17.6)	132 (5.6)	33 (25.0)	2,143 (91.2)	19 (0.9)
	Prison 5	676	35 (5.2)	54 (8.0)	54 (8.0)	57 (8.4)	<10 [6]	649 (96.0)	<10 [6]
	Prison 6	1,513	<10 [6]	<10 [6]	<10 [6]	<10 [6]	<10 [6]	1,513 (100.0)	<10 [6]
	Prison 7	654	136 (20.8)	157 (24.0)	157 (24.0)	60 (9.2)	15 (25.0)	581 (88.8)	15 (2.6)
	Prison 8	1,148	152 (13.2)	188 (16.4)	188 (16.4)	522 (45.5)	86 (16.5)	1,053 (91.7)	14 (1.3)
	Prison 9	996	152 (15.3)	185 (18.6)	185 (18.6)	79 (7.9)	20 (25.3)	869 (87.2)	19 (2.2)
	Prison 10	2,717	436 (16.0)	58 (2.1)	2,646 (97.4)	26 (1.0)	<10 [6]	2,646 (97.4)	26 (1.0)
	Prison 11	4,020	351 (8.7)	381 (9.5)	381 (9.5)	167 (4.2)	17 (10.2)	3,788 (94.2)	30 (0.8)
	Prison 12	792	<10 [6]	<10 [6]	<10 [6]	<10 [6]	<10 [6]	792 (100.0)	<10 [6]
	Prison 13	674	47 (7.0)	55 (8.2)	55 (8.2)	162 (24.0)	13 (8.0)	567 (84.1)	17 (3.0)
2019/20	Prison 1	1,410	128 (9.1)	103 (7.3)	103 (7.3)	394 (27.9)	70 (17.8)	1,340 (95.0)	12 (0.9)
	Prison 2	2,979	250 (8.4)	155 (5.2)	155 (5.2)	1,009 (33.9)	156 (15.5)	2,845 (95.5)	43 (1.5)
	Prison 3	2,809	527 (18.8)	437 (15.6)	437 (15.6)	260 (9.3)	90 (34.6)	2,748 (97.8)	<10 [6]
	Prison 4	2,651	491 (18.5)	367 (13.8)	367 (13.8)	184 (6.9)	59 (32.1)	2,411 (90.9)	14 (0.6)
	Prison 5	616	48 (7.8)	42 (6.8)	42 (6.8)	77 (12.5)	12 (15.6)	571 (92.7)	<10 [6]
	Prison 6	1,533	<10 [6]	<10 [6]	<10 [6]	<10 [6]	<10 [6]	1,533 (100.0)	<10 [6]
	Prison 7	860	211 (24.5)	176 (20.5)	176 (20.5)	98 (11.4)	22 (22.4)	747 (86.9)	17 (2.3)
	Prison 8	1,385	222 (16.0)	137 (9.9)	137 (9.9)	546 (39.4)	141 (25.8)	1,290 (93.1)	28 (2.2)
	Prison 9	1,092	175 (16.0)	182 (16.7)	182 (16.7)	94 (8.6)	21 (22.3)	948 (86.8)	23 (2.4)
	Prison 10	3,577	295 (8.2)	178 (5.0)	178 (5.0)	872 (24.4)	137 (15.7)	3,463 (96.8)	24 (0.7)
	Prison 11	5,348	569 (10.6)	420 (7.9)	420 (7.9)	291 (5.4)	45 (15.5)	5,053 (94.5)	31 (0.6)
	Prison 12	805	<10 [6]	<10 [6]	<10 [6]	<10 [6]	<10 [6]	805 (100.0)	<10 [6]
	Prison 13	746	41 (5.5)	60 (8.0)	60 (8.0)	229 (30.7)	17 (7.4)	634 (85.0)	<10 [6]
**Prison category**									
2017/18	A	1,664	198 (11.9)	198 (11.9)	198 (11.9)	209 (12.6)	30 (14.4)	1,456 (87.5)	39 (2.7)
	B	9,254	619 (6.7)	619 (6.7)	619 (6.7)	1,088 (11.8)	138 (12.7)	8,963 (96.9)	84 (0.9)
	C	6,035	744 (12.3)	744 (12.3)	744 (12.3)	540 (8.9)	89 (16.5)	5,792 (96.0)	56 (1.0)
	Closed	1,720	236 (13.7)	236 (13.7)	236 (13.7)	574 (33.4)	78 (13.6)	1,581 (91.9)	26 (1.6)
	D	2,189	37 (1.7)	37 (1.7)	37 (1.7)	55 (2.5)	<10 [6]	2,165 (98.9)	13 (0.6)
	YOI	815	<10 [6]	<10 [6]	<10 [6]	<10 [6]	<10 [6]	815 (100.0)	<10 [6]
2018/19	A	1,670	199 (11.9)	240 (14.4)	240 (14.4)	241 (14.4)	33 (13.7)	1,436 (86.0)	36 (2.5)
	B	9,442	1,010 (10.7)	658 (7.0)	3,246 (34.4)	954 (10.1)	150 (15.7)	9,042 (95.8)	88 (1.0)
	C	6,204	964 (15.5)	997 (16.1)	997 (16.1)	564 (9.1)	147 (26.1)	5,849 (94.3)	31 (0.5)
	Closed	1,802	288 (16.0)	345 (19.1)	345 (19.1)	582 (32.3)	101 (17.4)	1,634 (90.7)	29 (1.8)
	D	2,189	35 (1.6)	54 (2.5)	54 (2.5)	57 (2.6)	<10 [6]	2,162 (98.8)	<10 [6]
	YOI	792	<10 [6]	<10 [6]	<10 [6]	<10 [6]	<10 [6]	792 (100.0)	<10 [6]
2019/20	A	1,838	216 (11.8)	242 (13.2)	242 (13.2)	323 (17.6)	38 (11.8)	1,582 (86.1)	27 (1.7)
	B	11,904	1,114 (9.4)	753 (6.3)	753 (6.3)	2,172 (18.2)	338 (15.6)	11,361 (95.4)	98 (0.9)
	C	6,870	1,146 (16.7)	907 (13.2)	907 (13.2)	838 (12.2)	219 (26.1)	6,499 (94.6)	26 (0.4)
	Closed	2,245	433 (19.3)	313 (13.9)	313 (13.9)	644 (28.7)	163 (25.3)	2,037 (90.7)	45 (2.2)
	D	2,149	48 (2.2)	42 (2.0)	42 (2.0)	77 (3.6)	12 (15.6)	2,104 (97.9)	<10 [6]
	YOI	805	<10 [6]	<10 [6]	<10 [6]	<10 [6]	<10 [6]	805 (100.0)	<10 [6]
**Gender**									
2017/18	F	1,699	236 (13.9)	236 (13.9)	236 (13.9)	572 (33.7)	78 (13.6)	1,561 (91.9)	26 (1.7)
	M	19,977	1,598 (8.0)	1,598 (8.0)	1,598 (8.0)	1,894 (9.5)	259 (13.7)	19,210 (96.2)	192 (1.0)
2018/19	F	1,802	288 (16.0)	345 (19.1)	347 (19.3)	581 (32.2)	101 (17.4)	1,633 (90.6)	29 (1.8)
	M	20,295	2,208 (10.9)	1,949 (9.6)	4,535 (22.3)	1,817 (9.0)	334 (18.4)	19,280 (95.0)	161 (0.8)
2019/20	F	1,376	222 (16.1)	137 (10.0)	137 (10.0)	547 (39.8)	141 (25.8)	1,281 (93.1)	28 (2.2)
	M	23,570	2,525 (10.7)	1,944 (8.2)	1,944 (8.2)	3,410 (14.5)	608 (17.8)	22,355 (94.8)	159 (0.7)

[1]Eligible for indicator; [2]Achieve in current prison; [3]Achieve in previous prison; [4]Overall achievement - either current or previous prison; [5]Declined indicator; [6]Suppressed (<10) to avoid disclosure

Indicator		The proportion of prison population prescribed opioids in the preceding 8 weeks from 31st March							no comparable data in QOF 2019/20 data. Foy et al: 12.8% of all adult patients prescribed an opioid at least once; 6.6% of the sample were prescribed stronger opioids, or 0.85% of adults; For patients prescribed any opioids, the proportion prescribed stronger opioids was 13.7%.
	Group: Prison specific								
			All prison population			Any mental health diagnosis		All prison population AND NO coded diagnosis of neuropathic pain	
	Variable	Population	Prescribed any opioid in the preceding 8 weeks from 31st March (% popln)	Prescribed any strong opioid in the preceding 8 weeks from 31st March (% popln)	Prescribed benzodiazepines AND any opioid in preceding 8 weeks from 31st March (% popln)	Eligible (% popln)	Prescribed any opioid in the preceding 8 weeks from 31st March (% eligible)	Eligible (% popln)	Prescribed pregabalin OR gabapentin in the preceding 8 weeks from 31st March (% eligible)
**Sentence Status**									
2017/18	.	81	<10 [6]	<10 [6]	<10 [6]	<10 [6]	<10 [6]	80 (98.8)	<10 [6]
	Absconded	<10 [6]	<10 [6]	<10 [6]	<10 [6]	<10 [6]	<10 [6]	<10 [6]	<10 [6]
	Active In	15,872	1,584 (10.0)	1,584 (10.0)	1,584 (10.0)	1,586 (10.0)	244 (15.4)	15,090 (95.1)	178 (1.2)
	Active Out	1,052	61 (5.8)	61 (5.8)	61 (5.8)	134 (12.7)	11 (8.2)	1,026 (97.5)	13 (1.3)
	Convicted Sentence	2,125	91 (4.3)	91 (4.3)	91 (4.3)	367 (17.3)	35 (9.5)	2,072 (97.5)	14 (0.7)
	Downgrade in security category	<10 [6]	<10 [6]	<10 [6]	<10 [6]	<10 [6]	<10 [6]	<10 [6]	<10 [6]
	Internal Cell Move	<10 [6]	<10 [6]	<10 [6]	<10 [6]	<10 [6]	<10 [6]	<10 [6]	<10 [6]
	Judges Remand	92	<10 [6]	<10 [6]	<10 [6]	10 (10.9)	<10 [6]	91 (98.9)	<10 [6]
	Licence Revoke	52	<10 [6]	<10 [6]	<10 [6]	17 (32.7)	<10 [6]	51 (98.1)	<10 [6]
	On Remand	1,492	86 (5.8)	86 (5.8)	86 (5.8)	316 (21.2)	43 (13.6)	1,454 (97.5)	12 (0.8)
	Transfer	910	<10 [6]	<10 [6]	<10 [6]	32 (3.5)	<10 [6]	907 (99.7)	<10 [6]
	Upgrade in security category	<10 [6]	<10 [6]	<10 [6]	<10 [6]	<10 [6]	<10 [6]	<10 [6]	<10 [6]
2018/19	.	88	<10 [6]	<10 [6]	<10 [6]	<10 [6]	<10 [6]	85 (96.6)	<10 [6]
	Absconded	<10 [6]	<10 [6]	<10 [6]	<10 [6]	<10 [6]	<10 [6]	<10 [6]	<10 [6]
	Active In	18,145	2,214 (12.2)	2,052 (11.3)	4,276 (23.6)	1,635 (9.0)	319 (19.5)	17,095 (94.2)	152 (0.9)
	Active Out	835	80 (9.6)	40 (4.8)	376 (45.0)	72 (8.6)	<10 [6]	800 (95.8)	<10 [6]
	Convicted Sentence	1,320	109 (8.3)	113 (8.6)	116 (8.8)	326 (24.7)	59 (18.1)	1,275 (96.6)	16 (1.3)
	Downgrade in security category	<10 [6]	<10 [6]	<10 [6]	<10 [6]	<10 [6]	<10 [6]	<10 [6]	<10 [6]
	Internal Cell Move	<10 [6]	<10 [6]	<10 [6]	<10 [6]	<10 [6]	<10 [6]	<10 [6]	<10 [6]
	Judges Remand	<10 [6]	<10 [6]	<10 [6]	<10 [6]	<10 [6]	<10 [6]	<10 [6]	<10 [6]
	Licence Revoke	125	<10 [6]	14 (11.2)	14 (11.2)	44 (35.2)	<10 [6]	117 (93.6)	<10 [6]
	On Remand	1,059	77 (7.3)	70 (6.6)	78 (7.4)	311 (29.4)	45 (14.5)	1,020 (96.3)	13 (1.3)
	Transfer	518	<10 [6]	<10 [6]	<10 [6]	<10 [6]	<10 [6]	516 (99.6)	<10 [6]
	Upgrade in security category	<10 [6]	<10 [6]	<10 [6]	<10 [6]	<10 [6]	<10 [6]	<10 [6]	<10 [6]
2019/20	.	69	<10 [6]	<10 [6]	<10 [6]	<10 [6]	<10 [6]	68 (98.6)	<10 [6]
	Absconded	<10 [6]	<10 [6]	<10 [6]	<10 [6]	<10 [6]	<10 [6]	<10 [6]	<10 [6]
	Active In	22,424	2,709 (12.1)	2,087 (9.3)	2,087 (9.3)	3,114 (13.9)	634 (20.4)	21,137 (94.3)	167 (0.8)
	Active Out	625	29 (4.6)	35 (5.6)	35 (5.6)	97 (15.5)	<10 [6]	602 (96.3)	<10 [6]
	Convicted Sentence	1,361	124 (9.1)	72 (5.3)	72 (5.3)	389 (28.6)	70 (18.0)	1,300 (95.5)	19 (1.5)
	Downgrade in security category	<10 [6]	<10 [6]	<10 [6]	<10 [6]	<10 [6]	<10 [6]	<10 [6]	<10 [6]
	Internal Cell Move	<10 [6]	<10 [6]	<10 [6]	<10 [6]	<10 [6]	<10 [6]	<10 [6]	<10 [6]
	Judges Remand	19	<10 [6]	<10 [6]	<10 [6]	<10 [6]	<10 [6]	18 (94.7)	<10 [6]
	Licence Revoke	178	12 (6.7)	18 (10.1)	18 (10.1)	78 (43.8)	<10 [6]	166 (93.3)	<10 [6]
	On Remand	1,031	81 (7.9)	41 (4.0)	41 (4.0)	367 (35.6)	48 (13.1)	994 (96.4)	14 (1.4)
	Transfer	101	<10 [6]	<10 [6]	<10 [6]	<10 [6]	<10 [6]	100 (99.0)	<10 [6]
	Upgrade in security category	<10 [6]	<10 [6]	<10 [6]	<10 [6]	<10 [6]	<10 [6]	<10 [6]	<10 [6]
**Age - years**									
2017/18	10 - <20	468	<10 [6]	<10 [6]	<10 [6]	<10 [6]	<10 [6]	467 (99.8)	<10 [6]
	20 - <30	6,994	246 (3.5)	246 (3.5)	246 (3.5)	538 (7.7)	28 (5.2)	6,908 (98.8)	16 (0.2)
	30 - <40	7,051	700 (9.9)	700 (9.9)	700 (9.9)	846 (12.0)	115 (13.6)	6,804 (96.5)	75 (1.1)
	40 - <50	4,114	540 (13.1)	540 (13.1)	540 (13.1)	667 (16.2)	123 (18.4)	3,844 (93.4)	74 (1.9)
	50 - <60	2,107	229 (10.9)	229 (10.9)	229 (10.9)	297 (14.1)	52 (17.5)	1,918 (91.0)	42 (2.2)
	60 - <70	684	75 (11.0)	75 (11.0)	75 (11.0)	86 (12.6)	13 (15.1)	598 (87.4)	<10 [6]
	70 - <80	213	35 (16.4)	35 (16.4)	35 (16.4)	20 (9.4)	<10 [6]	189 (88.7)	<10 [6]
	80 - <90	40	<10 [6]	<10 [6]	<10 [6]	<10 [6]	<10 [6]	38 (95.0)	<10 [6]
	90 - <100	<10 [6]	<10 [6]	<10 [6]	<10 [6]	<10 [6]	<10 [6]	<10 [6]	<10 [6]
	100 - <110	<10 [6]	<10 [6]	<10 [6]	<10 [6]	<10 [6]	<10 [6]	<10 [6]	<10 [6]
2018/19	10 - <20	436	<10 [6]	<10 [6]	72 (16.5)	<10 [6]	<10 [6]	436 (100.0)	<10 [6]
	20 - <30	7,163	419 (5.8)	299 (4.2)	1,225 (17.1)	528 (7.4)	46 (8.7)	7,039 (98.3)	24 (0.3)
	30 - <40	7,381	985 (13.3)	872 (11.8)	1,735 (23.5)	864 (11.7)	165 (19.1)	7,015 (95.0)	66 (0.9)
	40 - <50	4,180	680 (16.3)	682 (16.3)	1,110 (26.6)	624 (14.9)	138 (22.1)	3,812 (91.2)	46 (1.2)
	50 - <60	1,978	283 (14.3)	303 (15.3)	508 (25.7)	270 (13.7)	72 (26.7)	1,772 (89.6)	40 (2.3)
	60 - <70	701	87 (12.4)	94 (13.4)	165 (23.5)	81 (11.6)	10 (12.3)	609 (86.9)	12 (2.0)
	70 - <80	209	28 (13.4)	35 (16.7)	52 (24.9)	21 (10.0)	<10 [6]	186 (89.0)	<10 [6]
	80 - <90	45	<10 [6]	<10 [6]	15 (33.3)	<10 [6]	<10 [6]	40 (88.9)	<10 [6]
	90 - <100	<10 [6]	<10 [6]	<10 [6]	<10 [6]	<10 [6]	<10 [6]	<10 [6]	<10 [6]
	100 - <110	<10 [6]	<10 [6]	<10 [6]	<10 [6]	<10 [6]	<10 [6]	<10 [6]	<10 [6]
2019/20	10 - <20	404	<10 [6]	<10 [6]	<10 [6]	14 (3.5)	<10 [6]	404 (100.0)	<10 [6]
	20 - <30	8,064	445 (5.5)	273 (3.4)	273 (3.4)	941 (11.7)	94 (10.0)	7,919 (98.2)	<10 [6]
	30 - <40	9,125	1,216 (13.3)	933 (10.2)	933 (10.2)	1,600 (17.5)	301 (18.8)	8,635 (94.6)	73 (0.8)
	40 - <50	4,948	905 (18.3)	648 (13.1)	648 (13.1)	994 (20.1)	263 (26.5)	4,525 (91.5)	82 (1.8)
	50 - <60	2,224	271 (12.2)	278 (12.5)	278 (12.5)	371 (16.7)	92 (24.8)	1,987 (89.3)	24 (1.2)
	60 - <70	751	79 (10.5)	85 (11.3)	85 (11.3)	108 (14.4)	17 (15.7)	658 (87.6)	13 (2.0)
	70 - <80	238	31 (13.0)	33 (13.9)	33 (13.9)	23 (9.7)	<10 [6]	209 (87.8)	<10 [6]
	80 - <90	53	<10 [6]	<10 [6]	<10 [6]	<10 [6]	<10 [6]	47 (88.7)	<10 [6]
	90 - <100	<10 [6]	<10 [6]	<10 [6]	<10 [6]	<10 [6]	<10 [6]	<10 [6]	<10 [6]
	100 - <110	<10 [6]	<10 [6]	<10 [6]	<10 [6]	<10 [6]	<10 [6]	<10 [6]	<10 [6]

[1]Eligible for indicator; [2]Achieve in current prison; [3]Achieve in previous prison; [4]Overall achievement - either current or previous prison; [5]Declined indicator; [6]Suppressed (<10) to avoid disclosure

Indicator *The proportion of prison population prescribed opioids in the preceding 8 weeks from 31st March*

Group: Prison specific

no comparable data in QOF 2019/20 data. Foy et al: 12.8% of all adult patients prescribed an opioid at least once; 6.6% of the sample were prescribed stronger opioids, or 0.85% of adults; For patients prescribed any opioids, the proportion prescribed stronger opioids was 13.7%.

			All prison population			Any mental health diagnosis		All prison population AND NO coded diagnosis of neuropathic pain	
	**Variable**	**Population**	**Prescribed any opioid in the preceding 8 weeks from 31st March (% popln)**	**Prescribed any strong opioid in the preceding 8 weeks from 31st March (% popln)**	**Prescribed benzodiazepines AND any opioid in preceding 8 weeks from 31st March (% popln)**	**Eligible (% popln)**	**Prescribed any opioid in the preceding 8 weeks from 31st March (% eligible)**	**Eligible (% popln)**	**Prescribed pregabalin OR gabapentin in the preceding 8 weeks from 31st March (% eligible)**
**Length of Stay (months)**									
2017/18	<1	4,474	190 (4.2)	190 (4.2)	190 (4.2)	416 (9.3)	33 (7.9)	4,369 (97.7)	22 (0.5)
	1-<6	8,075	527 (6.5)	527 (6.5)	527 (6.5)	930 (11.5)	98 (10.5)	7,839 (97.1)	62 (0.8)
	6-<12	3,672	361 (9.8)	361 (9.8)	361 (9.8)	463 (12.6)	76 (16.4)	3,548 (96.6)	45 (1.3)
	12-<24	2,832	316 (11.2)	316 (11.2)	316 (11.2)	301 (10.6)	58 (19.3)	2,710 (95.7)	33 (1.2)
	24+	2,624	440 (16.8)	440 (16.8)	440 (16.8)	356 (13.6)	72 (20.2)	2,306 (87.9)	56 (2.4)
2018/19	<1	4,801	312 (6.5)	299 (6.2)	1,189 (24.8)	400 (8.3)	45 (11.3)	4,632 (96.5)	19 (0.4)
	1-<6	7,742	837 (10.8)	656 (8.5)	1,935 (25.0)	838 (10.8)	129 (15.4)	7,447 (96.2)	53 (0.7)
	6-<12	3,616	426 (11.8)	401 (11.1)	730 (20.2)	403 (11.1)	74 (18.4)	3,441 (95.2)	40 (1.2)
	12-<24	3,447	501 (14.5)	472 (13.7)	557 (16.2)	379 (11.0)	107 (28.2)	3,241 (94.0)	42 (1.3)
	24+	2,493	420 (16.8)	466 (18.7)	471 (18.9)	378 (15.2)	80 (21.2)	2,154 (86.4)	36 (1.7)
2019/20	<1	5,745	359 (6.2)	345 (6.0)	345 (6.0)	742 (12.9)	85 (11.5)	5,520 (96.1)	26 (0.5)
	1-<6	9,697	1,033 (10.7)	672 (6.9)	672 (6.9)	1,621 (16.7)	272 (16.8)	9,304 (95.9)	79 (0.8)
	6-<12	5,090	792 (15.6)	451 (8.9)	451 (8.9)	863 (17.0)	221 (25.6)	4,803 (94.4)	47 (1.0)
	12-<24	3,244	455 (14.0)	399 (12.3)	399 (12.3)	474 (14.6)	118 (24.9)	3,024 (93.2)	32 (1.1)
	24+	2,035	318 (15.6)	390 (19.2)	390 (19.2)	354 (17.4)	74 (20.9)	1,737 (85.4)	20 (1.2)
**Ethnic Group**									
2017/18	White	15,638	1,558 (10.0)	1,558 (10.0)	1,558 (10.0)	2,120 (13.6)	303 (14.3)	14,854 (95.0)	184 (1.2)
	Mixed	431	19 (4.4)	19 (4.4)	19 (4.4)	44 (10.2)	<10 [6]	415 (96.3)	<10 [6]
	Asian or Asian British	813	19 (2.3)	19 (2.3)	19 (2.3)	47 (5.8)	<10 [6]	803 (98.8)	<10 [6]
	Black or Black British	404	13 (3.2)	13 (3.2)	13 (3.2)	41 (10.1)	<10 [6]	390 (96.5)	<10 [6]
	Chinese and Other	214	<10 [6]	<10 [6]	<10 [6]	26 (12.1)	<10 [6]	210 (98.1)	<10 [6]
	Unclassified	372	17 (4.6)	17 (4.6)	17 (4.6)	36 (9.7)	<10 [6]	347 (93.3)	<10 [6]
2018/19	White	14,911	2,062 (13.8)	2,010 (13.5)	3,779 (25.3)	2,043 (13.7)	395 (19.3)	13,898 (93.2)	148 (1.1)
	Mixed	371	31 (8.4)	21 (5.7)	60 (16.2)	41 (11.1)	<10 [6]	350 (94.3)	<10 [6]
	Asian or Asian British	726	30 (4.1)	26 (3.6)	163 (22.5)	48 (6.6)	<10 [6]	708 (97.5)	<10 [6]
	Black or Black British	364	19 (5.2)	11 (3.0)	45 (12.4)	31 (8.5)	<10 [6]	347 (95.3)	<10 [6]
	Chinese and Other	167	10 (6.0)	<10 [6]	31 (18.6)	21 (12.6)	<10 [6]	161 (96.4)	<10 [6]
	Unclassified	409	33 (8.1)	18 (4.4)	49 (12.0)	25 (6.1)	<10 [6]	384 (93.9)	<10 [6]
2019/20	White	16,606	2,442 (14.7)	2,042 (12.3)	2,042 (12.3)	3,395 (20.4)	694 (20.4)	15,401 (92.7)	173 (1.1)
	Mixed	409	20 (4.9)	22 (5.4)	22 (5.4)	67 (16.4)	<10 [6]	377 (92.2)	<10 [6]
	Asian or Asian British	755	29 (3.8)	28 (3.7)	28 (3.7)	91 (12.1)	<10 [6]	733 (97.1)	<10 [6]
	Black or Black British	451	25 (5.5)	24 (5.3)	24 (5.3)	58 (12.9)	<10 [6]	432 (95.8)	<10 [6]
	Chinese and Other	163	<10 [6]	<10 [6]	<10 [6]	21 (12.9)	<10 [6]	157 (96.3)	<10 [6]
	Unclassified	387	32 (8.3)	26 (6.7)	26 (6.7)	49 (12.7)	<10 [6]	364 (94.1)	<10 [6]

[1]Eligible for indicator; [2]Achieve in current prison; [3]Achieve in previous prison; [4]Overall achievement - either current or previous prison; [5]Declined indicator; [6]Suppressed (<10) to avoid disclosure

Indicator	The proportion of new receptions to prison who have been asked for consent to transfer medical records from GP to prison healthcare						Community achievement 2019/20 (%): NA
	**Group: Prison specific**						
	**Variable**	**Population**	**Eligible[1] (% popln)**	**Satisfy[2] (% eligible)**	**Elsewhere[3] (% eligible)**	**Achieve[4] (% eligible)**	
**Year**							
	2017/18	21,677	14,248 (65.7)	7,118 (50.0)	526 (3.7)	7,644 (53.6)	
	2018/19	22,099	14,907 (67.5)	10,661 (71.5)	477 (3.2)	11,138 (74.7)	
	2019/20	25,811	17,957 (69.6)	11,519 (64.1)	1,120 (6.2)	12,639 (70.4)	
**Prison**							
2017/18	Prison 1	1,323	818 (61.8)	41 (5.0)	99 (12.1)	140 (17.1)	
	Prison 2	3,261	2,502 (76.7)	20 (0.8)	60 (2.4)	80 (3.2)	
	Prison 3	2,623	1,834 (69.9)	1,702 (92.8)	23 (1.3)	1,725 (94.1)	
	Prison 4	2,089	1,091 (52.2)	1,014 (92.9)	20 (1.8)	1,034 (94.8)	
	Prison 5	637	366 (57.5)	<10 [6]	114 (31.1)	117 (32.0)	
	Prison 6	1,552	555 (35.8)	173 (31.2)	72 (13.0)	245 (44.1)	
	Prison 7	635	426 (67.1)	378 (88.7)	<10 [6]	380 (89.2)	
	Prison 8	1,085	802 (73.9)	683 (85.2)	<10 [6]	683 (85.2)	
	Prison 9	981	240 (24.5)	168 (70.0)	25 (10.4)	193 (80.4)	
	Prison 10	2,523	2,030 (80.5)	130 (6.4)	59 (2.9)	189 (9.3)	
	Prison 11	3,470	2,978 (85.8)	2,367 (79.5)	30 (1.0)	2,397 (80.5)	
	Prison 12	815	440 (54.0)	401 (91.1)	<10 [6]	407 (92.5)	
	Prison 13	683	166 (24.3)	38 (22.9)	16 (9.6)	54 (32.5)	
2018/19	Prison 1	1,333	856 (64.2)	125 (14.6)	176 (20.6)	301 (35.2)	
	Prison 2	2,705	2,076 (76.7)	22 (1.1)	103 (5.0)	125 (6.0)	
	Prison 3	2,522	1,635 (64.8)	1,558 (95.3)	<10 [6]	1,567 (95.8)	
	Prison 4	2,349	1,320 (56.2)	1,174 (88.9)	55 (4.2)	1,229 (93.1)	
	Prison 5	676	419 (62.0)	346 (82.6)	11 (2.6)	357 (85.2)	
	Prison 6	1,513	741 (49.0)	659 (88.9)	12 (1.6)	671 (90.6)	
	Prison 7	654	431 (65.9)	414 (96.1)	<10 [6]	415 (96.3)	
	Prison 8	1,148	844 (73.5)	771 (91.4)	<10 [6]	771 (91.4)	
	Prison 9	996	248 (24.9)	170 (68.5)	49 (19.8)	219 (88.3)	
	Prison 10	2,717	2,265 (83.4)	1,923 (84.9)	<10 [6]	1,928 (85.1)	
	Prison 11	4,020	3,441 (85.6)	2,938 (85.4)	41 (1.2)	2,979 (86.6)	
	Prison 12	792	447 (56.4)	418 (93.5)	<10 [6]	424 (94.9)	
	Prison 13	674	184 (27.3)	143 (77.7)	<10 [6]	152 (82.6)	
2019/20	Prison 1	1,410	927 (65.7)	349 (37.6)	267 (28.8)	616 (66.5)	
	Prison 2	2,979	2,419 (81.2)	49 (2.0)	174 (7.2)	223 (9.2)	
	Prison 3	2,809	1,780 (63.4)	1,430 (80.3)	141 (7.9)	1,571 (88.3)	
	Prison 4	2,651	1,522 (57.4)	1,061 (69.7)	220 (14.5)	1,281 (84.2)	
	Prison 5	616	354 (57.5)	262 (74.0)	30 (8.5)	292 (82.5)	
	Prison 6	1,533	904 (59.0)	837 (92.6)	18 (2.0)	855 (94.6)	
	Prison 7	860	603 (70.1)	481 (79.8)	17 (2.8)	498 (82.6)	
	Prison 8	1,385	1,048 (75.7)	952 (90.8)	<10 [6]	952 (90.8)	
	Prison 9	1,092	276 (25.3)	142 (51.4)	62 (22.5)	204 (73.9)	
	Prison 10	3,577	2,760 (77.2)	2,013 (72.9)	37 (1.3)	2,050 (74.3)	
	Prison 11	5,348	4,676 (87.4)	3,437 (73.5)	84 (1.8)	3,521 (75.3)	
	Prison 12	805	441 (54.8)	333 (75.5)	50 (11.3)	383 (86.8)	
	Prison 13	746	247 (33.1)	173 (70.0)	20 (8.1)	193 (78.1)	
**Prison category**							
2017/18	A	1,664	406 (24.4)	206 (50.7)	41 (10.1)	247 (60.8)	
	B	9,254	7,510 (81.2)	2,517 (33.5)	149 (2.0)	2,666 (35.5)	
	C	6,035	3,743 (62.0)	2,757 (73.7)	142 (3.8)	2,899 (77.5)	
	Closed	1,720	1,228 (71.4)	1,061 (86.4)	<10 [6]	1,063 (86.6)	
	D	2,189	921 (42.1)	176 (19.1)	186 (20.2)	362 (39.3)	
	YOI	815	440 (54.0)	401 (91.1)	6 (1.4)	407 (92.5)	
2018/19	A	1,670	432 (25.9)	313 (72.5)	58 (13.4)	371 (85.9)	
	B	9,442	7,782 (82.4)	4,883 (62.7)	149 (1.9)	5,032 (64.7)	
	C	6,204	3,811 (61.4)	2,857 (75.0)	240 (6.3)	3,097 (81.3)	
	Closed	1,802	1,275 (70.8)	1,185 (92.9)	<10 [6]	1,186 (93.0)	
	D	2,189	1,160 (53.0)	1,005 (86.6)	23 (2.0)	1,028 (88.6)	
	YOI	792	447 (56.4)	418 (93.5)	6 (1.3)	424 (94.9)	
2019/20	A	1,838	523 (28.5)	315 (60.2)	82 (15.7)	397 (75.9)	
	B	11,904	9,855 (82.8)	5,499 (55.8)	295 (3.0)	5,794 (58.8)	
	C	6,870	4,229 (61.6)	2,840 (67.2)	628 (14.8)	3,468 (82.0)	
	Closed	2,245	1,651 (73.5)	1,433 (86.8)	17 (1.0)	1,450 (87.8)	
	D	2,149	1,258 (58.5)	1,099 (87.4)	48 (3.8)	1,147 (91.2)	
	YOI	805	441 (54.8)	333 (75.5)	50 (11.3)	383 (86.8)	
**Gender**							
2017/18	F	1,699	1,206 (71.0)	1,058 (87.7)	<10 [6]	1,060 (87.9)	
	M	19,977	13,041 (65.3)	6,059 (46.5)	524 (4.0)	6,583 (50.5)	
2018/19	F	1,802	1,274 (70.7)	1,184 (92.9)	<10 [6]	1,187 (93.2)	
	M	20,295	13,631 (67.2)	9,476 (69.5)	474 (3.5)	9,950 (73.0)	
2019/20	F	1,376	1,042 (75.7)	952 (91.4)	<10 [6]	952 (91.4)	
	M	23,570	16,307 (69.2)	10,087 (61.9)	1,104 (6.8)	11,191 (68.6)	

[1]Eligible for indicator; [2]Achieve in current prison; [3]Achieve in previous prison; [4]Overall achievement - either current or previous prison; [5]Declined indicator; [6]Suppressed (<10) to avoid disclosure

Indicator	*The proportion of new receptions to prison who have been asked for consent to transfer medical records from GP to prison healthcare*						*Community achievement 2019/20 (%): NA*
	**Group: Prison specific**						
	**Variable**	**Population**	**Eligible[1] (% popln)**	**Satisfy[2] (% eligible)**	**Elsewhere[3] (% eligible)**	**Achieve[4] (% eligible)**	
**Sentence Status**							
2017/18	.	81	77 (95.1)	<10 [6]	<10 [6]	<10 [6]	
	Absconded	<10 [6]	<10 [6]	<10 [6]	<10 [6]	<10 [6]	
	Active In	15,872	10,624 (66.9)	6,717 (63.2)	438 (4.1)	7,155 (67.3)	
	Active Out	1,052	809 (76.9)	254 (31.4)	34 (4.2)	288 (35.6)	
	Convicted Sentence	2,125	1,567 (73.7)	80 (5.1)	35 (2.2)	115 (7.3)	
	Downgrade in security category	<10 [6]	<10 [6]	<10 [6]	<10 [6]	<10 [6]	
	Internal Cell Move	<10 [6]	<10 [6]	<10 [6]	<10 [6]	<10 [6]	
	Judges Remand	92	53 (57.6)	<10 [6]	<10 [6]	<10 [6]	
	Licence Revoke	52	51 (98.1)	<10 [6]	<10 [6]	<10 [6]	
	On Remand	1,492	1,067 (71.5)	58 (5.4)	12 (1.1)	70 (6.6)	
	Transfer	910	<10 [6]	<10 [6]	<10 [6]	<10 [6]	
	Upgrade in security category	<10 [6]	<10 [6]	<10 [6]	<10 [6]	<10 [6]	
2018/19	.	88	84 (95.5)	<10 [6]	<10 [6]	<10 [6]	
	Absconded	<10 [6]	<10 [6]	<10 [6]	<10 [6]	<10 [6]	
	Active In	18,145	12,308 (67.8)	10,028 (81.5)	395 (3.2)	10,423 (84.7)	
	Active Out	835	580 (69.5)	450 (77.6)	<10 [6]	459 (79.1)	
	Convicted Sentence	1,320	1,050 (79.5)	125 (11.9)	46 (4.4)	171 (16.3)	
	Downgrade in security category	<10 [6]	<10 [6]	<10 [6]	<10 [6]	<10 [6]	
	Internal Cell Move	<10 [6]	<10 [6]	<10 [6]	<10 [6]	<10 [6]	
	Judges Remand	<10 [6]	<10 [6]	<10 [6]	<10 [6]	<10 [6]	
	Licence Revoke	125	108 (86.4)	<10 [6]	<10 [6]	10 (9.3)	
	On Remand	1,059	768 (72.5)	46 (6.0)	17 (2.2)	63 (8.2)	
	Transfer	518	<10 [6]	<10 [6]	<10 [6]	<10 [6]	
	Upgrade in security category	<10 [6]	<10 [6]	<10 [6]	<10 [6]	<10 [6]	
2019/20	.	69	61 (88.4)	<10 [6]	<10 [6]	<10 [6]	
	Absconded	<10 [6]	<10 [6]	<10 [6]	<10 [6]	<10 [6]	
	Active In	22,424	15,308 (68.3)	11,023 (72.0)	1,008 (6.6)	12,031 (78.6)	
	Active Out	625	448 (71.7)	320 (71.4)	13 (2.9)	333 (74.3)	
	Convicted Sentence	1,361	1,148 (84.3)	85 (7.4)	60 (5.2)	145 (12.6)	
	Downgrade in security category	<10 [6]	<10 [6]	<10 [6]	<10 [6]	<10 [6]	
	Internal Cell Move	<10 [6]	<10 [6]	<10 [6]	<10 [6]	<10 [6]	
	Judges Remand	19	15 (78.9)	<10 [6]	<10 [6]	<10 [6]	
	Licence Revoke	178	156 (87.6)	19 (12.2)	11 (7.1)	30 (19.2)	
	On Remand	1,031	781 (75.8)	52 (6.7)	21 (2.7)	73 (9.3)	
	Transfer	101	37 (36.6)	12 (32.4)	<10 [6]	16 (43.2)	
	Upgrade in security category	<10 [6]	<10 [6]	<10 [6]	<10 [6]	<10 [6]	
**Age - years**							
2017/18	10 - <20	468	392 (83.8)	287 (73.2)	<10 [6]	295 (75.3)	
	20 - <30	6,994	4,871 (69.6)	2,450 (50.3)	183 (3.8)	2,633 (54.1)	
	30 - <40	7,051	4,712 (66.8)	2,369 (50.3)	158 (3.4)	2,527 (53.6)	
	40 - <50	4,114	2,638 (64.1)	1,263 (47.9)	102 (3.9)	1,365 (51.7)	
	50 - <60	2,107	1,195 (56.7)	542 (45.4)	59 (4.9)	601 (50.3)	
	60 - <70	684	328 (48.0)	147 (44.8)	12 (3.7)	159 (48.5)	
	70 - <80	213	84 (39.4)	52 (61.9)	<10 [6]	56 (66.7)	
	80 - <90	40	24 (60.0)	<10 [6]	<10 [6]	<10 [6]	
	90 - <100	<10 [6]	<10 [6]	<10 [6]	<10 [6]	<10 [6]	
	100 - <110	<10 [6]	<10 [6]	<10 [6]	<10 [6]	<10 [6]	
2018/19	10 - <20	436	375 (86.0)	334 (89.1)	<10 [6]	336 (89.6)	
	20 - <30	7,163	5,152 (71.9)	3,639 (70.6)	177 (3.4)	3,816 (74.1)	
	30 - <40	7,381	5,098 (69.1)	3,652 (71.6)	145 (2.8)	3,797 (74.5)	
	40 - <50	4,180	2,713 (64.9)	1,952 (71.9)	80 (2.9)	2,032 (74.9)	
	50 - <60	1,978	1,110 (56.1)	767 (69.1)	52 (4.7)	819 (73.8)	
	60 - <70	701	358 (51.1)	251 (70.1)	16 (4.5)	267 (74.6)	
	70 - <80	209	79 (37.8)	57 (72.2)	<10 [6]	62 (78.5)	
	80 - <90	45	18 (40.0)	<10 [6]	<10 [6]	<10 [6]	
	90 - <100	<10 [6]	<10 [6]	<10 [6]	<10 [6]	<10 [6]	
	100 - <110	<10 [6]	<10 [6]	<10 [6]	<10 [6]	<10 [6]	
2019/20	10 - <20	404	342 (84.7)	226 (66.1)	31 (9.1)	257 (75.1)	
	20 - <30	8,064	5,819 (72.2)	3,642 (62.6)	385 (6.6)	4,027 (69.2)	
	30 - <40	9,125	6,583 (72.1)	4,278 (65.0)	412 (6.3)	4,690 (71.2)	
	40 - <50	4,948	3,395 (68.6)	2,275 (67.0)	190 (5.6)	2,465 (72.6)	
	50 - <60	2,224	1,323 (59.5)	821 (62.1)	70 (5.3)	891 (67.3)	
	60 - <70	751	365 (48.6)	205 (56.2)	23 (6.3)	228 (62.5)	
	70 - <80	238	104 (43.7)	57 (54.8)	<10 [6]	64 (61.5)	
	80 - <90	53	25 (47.2)	15 (60.0)	<10 [6]	17 (68.0)	
	90 - <100	<10 [6]	<10 [6]	<10 [6]	<10 [6]	<10 [6]	
	100 - <110	<10 [6]	<10 [6]	<10 [6]	<10 [6]	<10 [6]	

[1]Eligible for indicator; [2]Achieve in current prison; [3]Achieve in previous prison; [4]Overall achievement - either current or previous prison; [5]Declined indicator; [6]Suppressed (<10) to avoid disclosure

Indicator		The proportion of new receptions to prison who have been asked for consent to transfer medical records from GP to prison healthcare					Community achievement 2019/20 (%): NA
	Group: Prison specific						
	Variable	Population	Eligible[1] (% popln)	Satisfy[2] (% eligible)	Elsewhere[3] (% eligible)	Achieve[4] (% eligible)	
**Length of Stay (months)**							
2017/18	<1	4,474	4,209 (94.1)	1,877 (44.6)	79 (1.9)	1,956 (46.5)	
	1-<6	8,075	6,274 (77.7)	3,061 (48.8)	240 (3.8)	3,301 (52.6)	
	6-<12	3,672	2,008 (54.7)	1,055 (52.5)	106 (5.3)	1,161 (57.8)	
	12-<24	2,832	1,113 (39.3)	674 (60.6)	51 (4.6)	725 (65.1)	
	24+	2,624	644 (24.5)	451 (70.0)	50 (7.8)	501 (77.8)	
2018/19	<1	4,801	4,551 (94.8)	3,000 (65.9)	81 (1.8)	3,081 (67.7)	
	1-<6	7,742	6,147 (79.4)	4,436 (72.2)	181 (2.9)	4,617 (75.1)	
	6-<12	3,616	2,082 (57.6)	1,576 (75.7)	63 (3.0)	1,639 (78.7)	
	12-<24	3,447	1,821 (52.8)	1,403 (77.0)	131 (7.2)	1,534 (84.2)	
	24+	2,493	306 (12.3)	246 (80.4)	21 (6.9)	267 (87.3)	
2019/20	<1	5,745	5,530 (96.3)	3,276 (59.2)	118 (2.1)	3,394 (61.4)	
	1-<6	9,697	7,922 (81.7)	4,994 (63.0)	565 (7.1)	5,559 (70.2)	
	6-<12	5,090	3,560 (69.9)	2,451 (68.8)	400 (11.2)	2,851 (80.1)	
	12-<24	3,244	945 (29.1)	798 (84.4)	37 (3.9)	835 (88.4)	
	24+	2,035	<10 [6]	<10 [6]	<10 [6]	<10 [6]	
**Ethnic Group**							
2017/18	White	15,638	10,182 (65.1)	4,945 (48.6)	424 (4.2)	5,369 (52.7)	
	Mixed	431	247 (57.3)	120 (48.6)	10 (4.0)	130 (52.6)	
	Asian or Asian British	813	514 (63.2)	188 (36.6)	28 (5.4)	216 (42.0)	
	Black or Black British	404	247 (61.1)	99 (40.1)	<10 [6]	108 (43.7)	
	Chinese and Other	214	137 (64.0)	42 (30.7)	<10 [6]	49 (35.8)	
	Unclassified	372	207 (55.6)	115 (55.6)	<10 [6]	117 (56.5)	
2018/19	White	14,911	9,618 (64.5)	6,697 (69.6)	357 (3.7)	7,054 (73.3)	
	Mixed	371	212 (57.1)	132 (62.3)	14 (6.6)	146 (68.9)	
	Asian or Asian British	726	424 (58.4)	289 (68.2)	20 (4.7)	309 (72.9)	
	Black or Black British	364	224 (61.5)	122 (54.5)	13 (5.8)	135 (60.3)	
	Chinese and Other	167	110 (65.9)	71 (64.5)	<10 [6]	74 (67.3)	
	Unclassified	409	252 (61.6)	210 (83.3)	<10 [6]	212 (84.1)	
2019/20	White	16,606	11,251 (67.8)	7,218 (64.2)	797 (7.1)	8,015 (71.2)	
	Mixed	409	250 (61.1)	127 (50.8)	28 (11.2)	155 (62.0)	
	Asian or Asian British	755	429 (56.8)	273 (63.6)	36 (8.4)	309 (72.0)	
	Black or Black British	451	285 (63.2)	149 (52.3)	41 (14.4)	190 (66.7)	
	Chinese and Other	163	105 (64.4)	54 (51.4)	<10 [6]	62 (59.0)	
	Unclassified	387	228 (58.9)	169 (74.1)	12 (5.3)	181 (79.4)	

[1]Eligible for indicator; [2]Achieve in current prison; [3]Achieve in previous prison; [4]Overall achievement - either current or previous prison; [5]Declined indicator; [6]Suppressed (<10) to avoid disclosure

Indicator ***The proportion of new receptions to prison with a code for a second-stage health assessment in the past 12 months, plus clinical codes for in possession risk assessment and medicines reconciliation added in the week before the second-stage assessment***

Group: Prison specific

Community achievement 2019/20 (%): NA

	Variable	Population	Eligible - had a 2nd screen assessment (% popln)	Both Medicines Reconciliation (MR) and In-Possession (IP) assessment within 7 days (% eligible)	Had MR during stay, but did not satisfy both within 7 days (% eligible)	Had IP during stay, but did not satisfy both within 7 days (% eligible)
**Year**						
	2019/20	8,245	6,173 (74.9)	2,359 (38.2)	319 (5.2)	3,810 (61.7)
**Prison**						
2019/20	Prison 1	486	410 (84.4)	408 (99.5)	<10 [6]	<10 [6]
	Prison 2	764	704 (92.1)	602 (85.5)	101 (14.3)	102 (14.5)
	Prison 3	1,137	1,014 (89.2)	24 (2.4)	14 (1.4)	986 (97.2)
	Prison 4	1,309	1,049 (80.1)	<10 [6]	<10 [6]	1,049 (100.0)
	Prison 5	208	190 (91.3)	<10 [6]	<10 [6]	185 (97.4)
	Prison 6	465	358 (77.0)	246 (68.7)	55 (15.4)	112 (31.3)
	Prison 7	261	251 (96.2)	<10 [6]	<10 [6]	246 (98.0)
	Prison 8	350	340 (97.1)	221 (65.0)	119 (35.0)	119 (35.0)
	Prison 9	827	295 (35.7)	13 (4.4)	10 (3.4)	282 (95.6)
	Prison 10	666	655 (98.3)	653 (99.7)	<10 [6]	<10 [6]
	Prison 11	890	323 (36.3)	<10 [6]	<10 [6]	323 (100.0)
	Prison 12	308	301 (97.7)	<10 [6]	<10 [6]	298 (99.0)
	Prison 13	574	283 (49.3)	179 (63.3)	10 (3.5)	104 (36.7)
2019/20	A	1,401	578 (41.3)	192 (33.2)	20 (3.5)	386 (66.8)
	B	2,320	1,682 (72.5)	1,255 (74.6)	104 (6.2)	427 (25.4)
	C	2,932	2,473 (84.3)	432 (17.5)	16 (0.6)	2,037 (82.4)
	Closed	611	591 (96.7)	226 (38.2)	122 (20.6)	365 (61.8)
	D	673	548 (81.4)	251 (45.8)	55 (10.0)	297 (54.2)
	YOI	308	301 (97.7)	<10 [6]	<10 [6]	298 (99.0)
**Gender**						
2019/20	F	611	591 (96.7)	227 (38.4)	122 (20.6)	364 (61.6)
	M	7,634	5,582 (73.1)	2,132 (38.2)	197 (3.5)	3,446 (61.7)
**Sentence Status**						
2019/20	.	<10 [6]	<10 [6]	<10 [6]	<10 [6]	<10 [6]
	Active In	7,542	5,545 (73.5)	1,889 (34.1)	247 (4.5)	3,652 (65.9)
	Active Out	68	56 (82.4)	24 (42.9)	<10 [6]	32 (57.1)
	Convicted Sentence	304	270 (88.8)	197 (73.0)	25 (9.3)	73 (27.0)
	Judges Remand	<10 [6]	<10 [6]	<10 [6]	<10 [6]	<10 [6]
	Licence Revoke	33	30 (90.9)	27 (90.0)	<10 [6]	<10 [6]
	On Remand	291	267 (91.8)	219 (82.0)	40 (15.0)	48 (18.0)
	Transfer	<10 [6]	<10 [6]	<10 [6]	<10 [6]	<10 [6]
**Age - years**						
2019/20	10 - <20	147	116 (78.9)	30 (25.9)	<10 [6]	86 (74.1)
	20 - <30	2,325	1,833 (78.8)	719 (39.2)	80 (4.4)	1,113 (60.7)
	30 - <40	2,799	2,191 (78.3)	848 (38.7)	112 (5.1)	1,340 (61.2)
	40 - <50	1,687	1,257 (74.5)	484 (38.5)	74 (5.9)	773 (61.5)
	50 - <60	816	530 (65.0)	207 (39.1)	35 (6.6)	323 (60.9)
	60 - <70	337	188 (55.8)	51 (27.1)	<10 [6]	137 (72.9)
	70 - <80	114	48 (42.1)	14 (29.2)	<10 [6]	34 (70.8)
	80 - <90	20	10 (50.0)	<10 [6]	<10 [6]	<10 [6]
**Length of Stay (months)**						
	1-<6	2,713	2,230 (82.2)	913 (40.9)	160 (7.2)	1,315 (59.0)
	6-<12	1,696	1,487 (87.7)	604 (40.6)	45 (3.0)	882 (59.3)
	12-<24	1,416	1,173 (82.8)	364 (31.0)	47 (4.0)	808 (68.9)
	24+	1,352	492 (36.4)	123 (25.0)	29 (5.9)	369 (75.0)
**Ethnic Group**						
	Mixed	187	139 (74.3)	72 (51.8)	13 (9.4)	66 (47.5)
	Asian or Asian British	313	241 (77.0)	137 (56.8)	10 (4.1)	104 (43.2)
	Black or Black British	211	150 (71.1)	65 (43.3)	<10 [6]	85 (56.7)
	Chinese and Other	83	63 (75.9)	44 (69.8)	<10 [6]	18 (28.6)
	Unclassified	128	74 (57.8)	37 (50.0)	<10 [6]	37 (50.0)

[1]Eligible for indicator; [2]Achieve in current prison; [3]Achieve in previous prison; [4]Overall achievement - either current or previous prison; [5]Declined indicator; [6]Suppressed (<10) to avoid disclosure

Supplementary section 3: Multi-level mixed effects logistic regression model results for each indicator

Indicator ***The proportion of people with diabetes in whom the last blood pressure reading (measured in the preceding 12 months) is 140/80 mmHg or less***

Group: Diabetes, Asthma & Epilepsy Care

		**Univariate**		**Multivariable**			
	**Variable**	**Odds Ratio (OR) of achieving indicator**	**(95% CI) of OR**	**OR**	**(95% CI)**	**Probability of achieving indicator**	**(95% CI) of probability**
**Year**							
	2017/18 (comparator)	1.0	-	1.0	-	0.33	-
	2018/19	0.97	(0.78, 1.21)	0.92	(0.71, 1.19)	0.32	-
	2019/20	0.98	(0.79, 1.22)	1.17	(0.9, 1.51)	0.37	-
**Prison**							
	Prison 1	2.41	(1.44, 4.02)	2.69	(1.34, 5.38)	0.47	-
	Prison 2	1.29	(0.88, 1.9)	1.30	(0.81, 2.09)	0.33	-
	Prison 3	2.18	(1.52, 3.15)	2.18	(1.31, 3.63)	0.42	-
	Prison 4	3.14	(2.07, 4.77)	3.42	(2.01, 5.82)	0.52	-
	Prison 5	2.13	(1.23, 3.69)	2.86	(1.42, 5.77)	0.48	-
	Prison 6	2.03	(1.34, 3.09)	2.89	(1.63, 5.12)	0.48	-
	Prison 7	1.99	(1.14, 3.47)	0.00	(0, 0)	0.03	-
	Prison 8	5.66	(3.54, 9.03)	0.00	(0, 0)	0.05	-
	Prison 9	1.10	(0.76, 1.58)	0.95	(0.55, 1.63)	0.28	-
	Prison 10	2.36	(1.64, 3.39)	2.63	(1.65, 4.16)	0.46	-
	Prison 11 (comparator)	1.0	-	1.0	-	0.28	-
	Prison 12	2.93	(0.87, 9.88)	2.52	(0.58, 11.05)	0.45	-
	Prison 13	1.80	(1.15, 2.82)	1.41	(0.75, 2.67)	0.34	-
**Prison category**							
	A (comparator)	1.0	-				
	B	1.12	(0.86, 1.45)				
	C	1.94	(1.47, 2.57)				
	Closed	2.94	(2.05, 4.22)				
	D	1.61	(1.14, 2.27)				
	YOI	2.29	(0.69, 7.63)				
**Gender**							
	Female (comparator)	1.0	-	1.0	-	0.96	-
	Male	0.44	(0.32, 0.6)	-	-	0.29	-
**Age - years**							
	10 - <20	2.22	(0.31, 15.95)	1.79	(0.24, 13.51)	0.43	-
	20 - <30	2.48	(1.766, 3.47)	2.82	(1.87, 4.3)	0.53	-
	30 - <40	1.47	(1.103, 1.95)	1.59	(1.13, 2.2)	0.40	-
	40 - <50	0.95	(0.733, 1.23)	0.98	(0.72, 1.3)	0.30	-
	50 - <60 (comparator)	1.0	-	1.0	-	0.30	-
	60 - <70	0.88	(0.662, 1.16)	0.88	(0.63, 1.2)	0.28	-
	70 - <80	1.16	(0.798, 1.7)	1.17	(0.76, 1.8)	0.34	-
	80 - <90	1.97	(0.985, 3.96)	2.15	(1.003, 4.6)	0.47	-
	90 - <100	2.21	(0.137, 35.58)	1.76	(0.09, 33.25)	0.43	-
	100 - <110	-	-	-	-	-	-
**Length of Stay (months)**							
	<1	0.97	(0.73, 1.27)	1.23	(0.87, 1.74)	0.33	-
	1-<6 (comparator)	1.0	-	1.0	-	0.29	-
	6-<12	0.96	(0.72, 1.29)	1.03	(0.73, 1.45)	0.30	-
	12-<24	0.97	(0.73, 1.28)	1.12	(0.78, 1.61)	0.31	-
	24+	1.08	(0.84, 1.38)	1.95	(1.34, 2.85)	0.43	-
**Ethnic Group**							
	White (comparator)	1.0	-	1.0	-	0.34	-
	Mixed	1.36	(0.7, 2.67)	1.36	(0.65, 2.81)	0.41	-
	Asian or Asian British	0.63	(0.4, 0.99)	0.58	(0.36, 0.95)	0.24	-
	Black or Black British	0.65	(0.34, 1.24)	0.76	(0.39, 1.48)	0.29	-
	Chinese and Other	0.87	(0.3, 2.52)	0.49	(0.15, 1.61)	0.21	-
	Unclassified	1.77	(0.85, 3.7)	1.80	(0.83, 3.95)	0.47	-

Indicator		The percentage of patients with diabetes who have had the following care processes performed in the preceding 12 months: BMI measurement; BP measurement; HbA1c measurement; Cholesterol measurement; Record of smoking status; Foot examination; Albumin: creatinine ratio; Serum creatinine measurement															
	Group: Diabetes, Asthma & Epilepsy Care																
		Univariate: Odds ratios (95% CI)															
	Variable	BMI		Hb1Ac		Cholesterol		Smoking Status		Albumin Creatinine Ratio		Serum Creatinine		BP		Foot Exam	
**Year**																	
	2017/18 (comparator)	1.0	-	1.0	-	1.0	-	1.0	-	1.0	-	1.0	-	1.0	-	1.0	-
	2018/19	1.84	(1.42, 2.4)	1.02	(0.82, 1.27)	1.13	(0.91, 1.39)	1.44	(1.09, 1.92)	1.18	(0.93, 1.49)	0.27	(0.09, 0.81)	1.47	(1.07, 2.02)	1.02	(0.82, 1.27)
	2019/20	2.29	(1.75, 3)	1.06	(0.85, 1.32)	1.18	(0.96, 1.45)	1.21	(0.92, 1.58)	0.98	(0.77, 1.24)	0.25	(0.08, 0.75)	2.68	(1.87, 3.84)	1.10	(0.89, 1.36)
**Prison**																	
	Prison 1	1.43	(0.69, 2.97)	4.97	(2.86, 8.63)	6.28	(3.72, 10.62)	1.97	(1.02, 3.83)	3.15	(1.71, 5.81)	9.60	(0.98, 94.25)	1.22	(0.55, 2.75)	11.28	(6.38, 19.94)
	Prison 2	0.89	(0.58, 1.38)	2.09	(1.49, 2.92)	2.73	(1.91, 3.91)	1.94	(1.26, 2.99)	2.60	(1.63, 4.15)	0.90	(0.06, 14.46)	1.31	(0.77, 2.25)	6.27	(4.01, 9.81)
	Prison 3	0.79	(0.51, 1.22)	3.83	(2.69, 5.45)	4.05	(2.83, 5.8)	1.22	(0.82, 1.81)	3.66	(2.34, 5.75)	1.04	(0.06, 16.82)	1.21	(0.71, 2.06)	5.60	(3.57, 8.76)
	Prison 4	0.80	(0.49, 1.31)	5.44	(3.5, 8.47)	9.03	(5.79, 14.07)	1.13	(0.71, 1.8)	5.80	(3.55, 9.47)	2.74	(0.24, 30.84)	0.57	(0.34, 0.96)	4.50	(2.71, 7.48)
	Prison 5	0.46	(0.25, 0.83)	4.04	(2.29, 7.11)	4.94	(2.86, 8.51)	1.32	(0.7, 2.51)	4.47	(2.42, 8.26)	-	-	0.66	(0.32, 1.35)	4.17	(2.22, 7.83)
	Prison 6	1.96	(1.06, 3.62)	4.52	(2.97, 6.89)	6.07	(4, 9.23)	2.72	(1.55, 4.78)	7.59	(4.71, 12.24)	20.24	(2.59, 157.98)	22.26	(3.02, 163.87)	20.38	(12.37, 33.57)
	Prison 7	1.54	(0.69, 3.43)	2.88	(1.68, 4.93)	4.88	(2.83, 8.41)	1.79	(0.9, 3.58)	0.41	(0.12, 1.36)	-	-	2.22	(0.76, 6.48)	6.10	(3.33, 11.19)
	Prison 8	0.52	(0.31, 0.88)	1.79	(1.16, 2.78)	1.35	(0.82, 2.2)	1.77	(1, 3.15)	0.91	(0.44, 1.92)	-	-	1.30	(0.62, 2.72)	4.60	(2.67, 7.93)
	Prison 9	1.55	(0.98, 2.45)	12.87	(8.53, 19.43)	17.94	(12.09, 26.64)	4.30	(2.63, 7.03)	4.61	(3.02, 7.04)	0.82	(0.05, 13.16)	2.27	(1.26, 4.07)	15.94	(10.37, 24.49)
	Prison 10	0.42	(0.28, 0.62)	1.38	(0.99, 1.93)	1.82	(1.27, 2.62)	1.49	(0.99, 2.23)	0.61	(0.33, 1.14)	-	-	0.65	(0.41, 1.05)	2.39	(1.48, 3.85)
	Prison 11 (comparator)	1.0	-	1.0	-	1.0	-	1.0	-	1.0	-	1.0	-	1.0	-	1.0	-
	Prison 12	2.14	(0.26, 17.3)	4.40	(1.15, 16.92)	2.82	(0.84, 9.53)	1.54	(0.33, 7.32)	5.17	(1.44, 18.59)	-	-	1.44	(0.18, 11.69)	11.51	(3.32, 39.86)
	Prison 13	1.83	(0.96, 3.5)	19.30	(9.75, 38.22)	32.55	(17.03, 62.22)	2.98	(1.59, 5.57)	30.43	(17.62, 52.57)	1.89	(0.12, 30.58)	2.44	(1.06, 5.63)	26.17	(15.3, 44.75)
**Prison category**																	
	A (comparator)	1.0	-	1.0	-	1.0	-	1.0	-	1.0	-	1.0	-	1.0	-	1.0	-
	B	0.44	(0.31, 0.62)	0.10	(0.07, 0.13)	0.08	(0.06, 0.11)	0.35	(0.24, 0.51)	0.17	(0.13, 0.22)	0.52	(0.07, 3.72)	0.40	(0.26, 0.63)	0.14	(0.11, 0.19)
	C	0.53	(0.36, 0.77)	0.31	(0.22, 0.45)	0.27	(0.19, 0.37)	0.34	(0.22, 0.5)	0.53	(0.41, 0.7)	2.63	(0.53, 13.16)	0.39	(0.24, 0.62)	0.33	(0.25, 0.43)
	Closed	0.45	(0.28, 0.73)	0.15	(0.1, 0.23)	0.11	(0.07, 0.17)	0.47	(0.27, 0.79)	0.09	(0.05, 0.17)	-	-	0.68	(0.34, 1.34)	0.28	(0.2, 0.41)
	D	0.66	(0.42, 1.06)	0.30	(0.2, 0.47)	0.27	(0.19, 0.4)	0.55	(0.33, 0.91)	0.83	(0.6, 1.15)	11.97	(2.64, 54.17)	1.00	(0.5, 2)	0.68	(0.49, 0.94)
	YOI	1.31	(0.16, 10.6)	0.31	(0.08, 1.2)	0.14	(0.04, 0.46)	0.40	(0.08, 1.94)	0.67	(0.19, 2.31)	-	-	0.62	(0.08, 5.11)	0.63	(0.19, 2.09)
**Gender**																	
	Female (comparator)	1.0	-	1.0	-	1.0	-	1.0	-	1.0	-	1.0	-	1.0	-	1.0	-
	Male	1.23	(0.81, 1.86)	1.54	(1.1, 2.16)	2.23	(1.57, 3.16)	0.87	(0.55, 1.4)	4.86	(2.61, 9.05)	-	-	0.71	(0.39, 1.31)	1.21	(0.85, 1.72)
**Age - years**																	
	10 - <20	0.55	(0.05, 6.39)	0.21	(0.02, 2.32)	-	-	0.42	(0.04, 4.66)	-	-	-	-	0.25	(0.02, 2.98)	0.67	(0.06, 7.45)
	20 - <30	0.83	(0.53, 1.31)	0.44	(0.31, 0.64)	0.29	(0.19, 0.42)	1.05	(0.63, 1.73)	0.48	(0.3, 0.75)	0.69	(0.08, 5.7)	0.87	(0.49, 1.57)	0.49	(0.33, 0.72)
	30 - <40	0.82	(0.58, 1.17)	0.43	(0.32, 0.57)	0.50	(0.37, 0.66)	0.87	(0.6, 1.27)	0.40	(0.28, 0.57)	0.70	(0.14, 3.43)	0.96	(0.6, 1.53)	0.61	(0.46, 0.83)
	40 - <50	1.06	(0.78, 1.45)	0.54	(0.42, 0.69)	0.61	(0.48, 0.78)	0.92	(0.67, 1.26)	0.52	(0.39, 0.69)	0.97	(0.3, 3.08)	0.77	(0.53, 1.12)	0.62	(0.48, 0.79)
	50 - <60 (comparator)	1.0	-	1.0	-	1.0	-	1.0	-	1.0	-	1.0	-	1.0	-	1.0	-
	60 - <70	1.05	(0.76, 1.46)	1.07	(0.81, 1.41)	1.23	(0.95, 1.59)	0.88	(0.63, 1.23)	1.11	(0.85, 1.45)	0.93	(0.27, 3.22)	1.08	(0.71, 1.65)	1.24	(0.96, 1.59)
	70 - <80	0.89	(0.57, 1.38)	1.17	(0.79, 1.74)	1.11	(0.78, 1.6)	0.83	(0.52, 1.31)	1.11	(0.77, 1.61)	1.77	(0.45, 6.95)	1.00	(0.56, 1.78)	1.11	(0.78, 1.57)
	80 - <90	2.66	(0.8, 8.88)	1.77	(0.76, 4.1)	0.98	(0.5, 1.93)	0.80	(0.34, 1.87)	0.90	(0.44, 1.86)	-	-	4.26	(0.57, 31.69)	1.13	(0.58, 2.2)
	90 - <100	-	-	-	-	-	-	0.37	(0.03, 4.15)	1.07	(0.1, 11.89)	-	-	-	-	2.65	(0.24, 29.42)
	100 - <110	-	-	-	-	-	-	-	-	-	-	-	-	-	-	-	-
**Length of Stay (months)**																	
	<1	1.67	(1.14, 2.46)	0.28	(0.21, 0.39)	0.34	(0.25, 0.48)	1.01	(0.7, 1.44)	0.34	(0.2, 0.57)	0.92	(0.17, 5.06)	1.81	(1.12, 2.91)	0.31	(0.2, 0.48)
	1-<6 (comparator)	1.0	-	1.0	-	1.0	-	1.0	-	1.0	-	1.0	-	1.0	-	1.0	-
	6-<12	0.90	(0.65, 1.25)	2.80	(2.09, 3.73)	2.46	(1.88, 3.23)	0.79	(0.56, 1.1)	1.80	(1.29, 2.51)	2.54	(0.71, 9.09)	0.91	(0.62, 1.34)	3.04	(2.28, 4.05)
	12-<24	1.00	(0.71, 1.4)	3.68	(2.71, 5.02)	3.84	(2.88, 5.12)	1.02	(0.71, 1.46)	3.37	(2.46, 4.62)	3.72	(1.1, 12.52)	1.23	(0.81, 1.86)	4.12	(3.08, 5.51)
	24+	1.26	(0.93, 1.7)	8.28	(6.06, 11.32)	8.52	(6.45, 11.25)	1.36	(0.98, 1.89)	4.46	(3.37, 5.91)	0.47	(0.09, 2.61)	2.33	(1.54, 3.52)	5.72	(4.41, 7.43)
**Ethnic Group**																	
	White (comparator)	1.0	-	1.0	-	1.0	-	1.0	-	1.0	-	1.0	-	1.0	-	1.0	-
	Mixed	1.94	(0.68, 5.58)	0.94	(0.47, 1.91)	1.52	(0.75, 3.08)	1.43	(0.5, 4.1)	1.42	(0.7, 2.89)	6.61	(1.4, 31.19)	2.04	(0.48, 8.63)	1.24	(0.63, 2.43)
	Asian or Asian British	0.80	(0.5, 1.29)	0.94	(0.62, 1.42)	0.98	(0.66, 1.45)	0.74	(0.45, 1.22)	1.03	(0.67, 1.59)	1.17	(0.15, 9.11)	0.89	(0.48, 1.67)	1.05	(0.71, 1.56)
	Black or Black British	1.64	(0.69, 3.92)	1.24	(0.66, 2.33)	1.25	(0.7, 2.23)	1.08	(0.48, 2.43)	0.94	(0.5, 1.8)	5.78	(1.24, 27.05)	1.72	(0.53, 5.63)	0.96	(0.54, 1.7)
	Chinese and Other	0.46	(0.18, 1.19)	1.09	(0.41, 2.88)	0.91	(0.37, 2.25)	0.72	(0.24, 2.21)	3.07	(1.24, 7.63)	-	-	0.29	(0.11, 0.78)	2.59	(1.01, 6.63)
	Unclassified	0.47	(0.22, 0.99)	1.70	(0.73, 3.98)	1.28	(0.61, 2.65)	0.54	(0.24, 1.23)	1.32	(0.62, 2.83)	-	-	1.20	(0.36, 4.03)	1.59	(0.78, 3.24)

Indicator	*The percentage of patients with diabetes who have had the following care processes performed in the preceding 12 months: BMI measurement; BP measurement; HbA1c measurement; Cholesterol measurement; Record of smoking status; Foot examination; Albumin: creatinine ratio; Serum creatinine measurement*															
**Domain: Routine primary care**																
	**Multivariable**															
**Variable**	**BMI**		**Hb1Ac**		**Cholesterol**		**Smoking Status**		**Albumin Creatinine Ratio**		**Serum Creatinine**		**BP**		**Foot Exam**	
	**OR (95% CI)**	**Probability of achieving indicator (95% CI)**	**OR (95% CI)**	**Probability of achieving indicator (95% CI)**	**OR (95% CI)**	**Probability of achieving indicator (95% CI)**	**OR (95% CI)**	**Probability of achieving indicator (95% CI)**	**OR (95% CI)**	**Probability of achieving indicator (95% CI)**	**OR (95% CI)**	**Probability of achieving indicator (95% CI)**	**OR (95% CI)**	**Probability of achieving indicator (95% CI)**	**OR (95% CI)**	**Probability of achieving indicator (95% CI)**
**Year**																
2017/18 (comparator)	1.0	0.73 (-9.4, 10.86)	1.0	0.65 (0.05, 1.26)	1.0	0.53 (-7.66, 8.71)	1.0	0.82 (-7.83, 9.46)	1.0	0.26 (-1.99, 2.51)	1.0	0.04 (-7.15, 7.22)	1.0	0.84 (0.77, 0.91)	1.0	0.39 (-1.32, 2.09)
2018/19	1.8 (1.35, 2.42)	0.82 (-10.6, 12.24)	1.05 (0.79, 1.38)	0.66 (0.05, 1.28)	1.19 (0.91, 1.56)	0.56 (-8.13, 9.24)	1.37 (1, 1.88)	0.86 (-8.22, 9.94)	1.36 (1.02, 1.8)	0.31 (-2.38, 2.99)	0.16 (0.03, 0.75)	0.01 (-1.35, 1.37)	1.52 (1.06, 2.17)	0.89 (0.82, 0.96)	1.12 (0.86, 1.46)	0.41 (-1.39, 2.21)
2019/20	2.33 (1.7, 3.19)	0.86 (-11.01, 12.72)	1.32 (0.99, 1.77)	0.7 (0.05, 1.35)	1.6 (1.21, 2.11)	0.61 (-8.91, 10.13)	1.28 (0.92, 1.77)	0.85 (-8.14, 9.84)	1.23 (0.91, 1.66)	0.29 (-2.25, 2.83)	0.22 (0.06, 0.86)	0.01 (-1.83, 1.85)	2.6 (1.7, 4)	0.93 (0.86, 1)	1.31 (1, 1.71)	0.44 (-1.49, 2.37)
**Prison**																
Prison 1	2.05 (0.81, 5.19)	0.87 (-11.24, 12.98)	1.57 (0.73, 3.41)	0.78 (0.05, 1.51)	3.14 (1.54, 6.39)	0.71 (-10.41, 11.83)	4.13 (1.36, 12.51)	0.93 (-8.95, 10.82)	1.53 (0.71, 3.28)	0.37 (-2.84, 3.57)	12.13 (1, 146.7)	0.04 (-8.85, 8.94)	1.48 (0.51, 4.3)	0.92 (0.83, 1.02)	4 (1.95, 8.24)	0.58 (-1.96, 3.11)
Prison 2	1.11 (0.66, 1.87)	0.8 (-10.23, 11.83)	0.82 (0.54, 1.26)	0.68 (0.05, 1.31)	1.48 (0.96, 2.28)	0.57 (-8.35, 9.5)	1.61 (0.96, 2.69)	0.85 (-8.14, 9.84)	1.21 (0.7, 2.09)	0.32 (-2.52, 3.16)	-	-	1.44 (0.76, 2.7)	0.92 (0.84, 0.99)	2.95 (1.75, 4.98)	0.52 (-1.75, 2.78)
Prison 3	1.08 (0.61, 1.91)	0.79 (-10.18, 11.76)	0.94 (0.57, 1.55)	0.7 (0.05, 1.36)	1.27 (0.78, 2.06)	0.54 (-7.91, 8.99)	1.15 (0.66, 2)	0.8 (-7.69, 9.3)	1.24 (0.69, 2.23)	0.33 (-2.55, 3.21)	1.59 (0.09, 28.64)	0.01 (-1.31, 1.32)	1.08 (0.54, 2.14)	0.9 (0.82, 0.97)	1.48 (0.84, 2.61)	0.38 (-1.29, 2.06)
Prison 4	1.27 (0.69, 2.32)	0.81 (-10.48, 12.11)	1.08 (0.62, 1.88)	0.73 (0.05, 1.4)	2.9 (1.69, 4.97)	0.7 (-10.2, 11.6)	1.09 (0.6, 1.98)	0.8 (-7.62, 9.21)	1.57 (0.85, 2.88)	0.37 (-2.88, 3.62)	3.4 (0.26, 44.64)	0.01 (-2.73, 2.76)	0.66 (0.34, 1.29)	0.84 (0.76, 0.93)	1.23 (0.67, 2.26)	0.35 (-1.18, 1.88)
Prison 5	0.59 (0.29, 1.22)	0.69 (-8.84, 10.22)	1.05 (0.5, 2.21)	0.72 (0.05, 1.4)	1.62 (0.81, 3.21)	0.59 (-8.61, 9.79)	0.9 (0.42, 1.92)	0.76 (-7.31, 8.84)	1.19 (0.55, 2.6)	0.32 (-2.49, 3.13)	-	-	0.7 (0.29, 1.64)	0.85 (0.75, 0.96)	1.01 (0.46, 2.2)	0.32 (-1.07, 1.7)
Prison 6	3.78 (1.69, 8.48)	0.93 (-11.9, 13.75)	1.07 (0.6, 1.93)	0.72 (0.05, 1.4)	2.06 (1.17, 3.6)	0.64 (-9.29, 10.56)	2.78 (1.31, 5.89)	0.91 (-8.68, 10.49)	2.55 (1.37, 4.74)	0.47 (-3.62, 4.56)	18.36 (1.94, 174.02)	0.06 (-12.68, 12.8)	-	-	6.82 (3.62, 12.85)	0.68 (-2.29, 3.65)
Prison 7	-	0.07 (-0.89, 1.02)	-	0.04 (0, 0.08)	-	0.04 (-0.62, 0.71)	-	0.06 (-0.6, 0.73)	-	-	-	-	-	0.07 (0.06, 0.08)	-	0.03 (-0.1, 0.16)
Prison 8	-	0.05 (-0.69, 0.79)	-	0.04 (0, 0.08)	-	0.02 (-0.33, 0.38)	-	0.06 (-0.61, 0.73)	-	0.01 (-0.05, 0.06)	-	-	-	0.07 (0.06, 0.08)	-	0.03 (-0.09, 0.14)
Prison 9	2.86 (1.52, 5.39)	0.9 (-11.63, 13.44)	1.39 (0.77, 2.51)	0.76 (0.06, 1.47)	3.51 (2.02, 6.08)	0.73 (-10.67, 12.14)	4.39 (2.23, 8.66)	0.94 (-8.98, 10.86)	0.92 (0.51, 1.67)	0.28 (-2.16, 2.72)	1.11 (0.05, 26.73)	0.005 (-0.92, 0.93)	1.55 (0.7, 3.42)	0.92 (0.85, 1)	2.77 (1.56, 4.92)	0.5 (-1.71, 2.71)
Prison 10	0.44 (0.27, 0.73)	0.63 (-8.09, 9.35)	0.58 (0.38, 0.9)	0.62 (0.04, 1.2)	1.05 (0.67, 1.65)	0.5 (-7.37, 8.37)	1.1 (0.67, 1.82)	0.8 (-7.63, 9.23)	0.35 (0.17, 0.7)	0.16 (-1.25, 1.57)	-	0.004 (-0.83, 0.84)	0.68 (0.38, 1.22)	0.85 (0.77, 0.93)	1.3 (0.74, 2.29)	0.36 (-1.22, 1.93)
Prison 11 (comparator)	1.0	0.78 (-10.02, 11.58)	1.0	0.71 (0.05, 1.37)	1.0	0.49 (-7.22, 8.21)	1.0	0.78 (-7.48, 9.05)	1.0	0.29 (-2.27, 2.85)	1.0	-	1.0	0.89 (0.81, 0.97)	1.0	0.31 (-1.06, 1.69)
Prison 12	3.18 (0.33, 30.72)	0.91 (-11.74, 13.57)	1.63 (0.27, 9.78)	0.79 (0.02, 1.55)	2.66 (0.53, 13.4)	0.68 (-9.98, 11.35)	1.05 (0.17, 6.35)	0.79 (-7.56, 9.14)	1.49 (0.28, 7.96)	0.36 (-2.82, 3.54)	-	-	1.68 (0.15, 18.66)	0.93 (0.77, 1.09)	3.55 (0.75, 16.77)	0.55 (-1.89, 3)
Prison 13	3.94 (1.72, 9)	0.93 (-11.93, 13.79)	1.73 (0.76, 3.94)	0.79 (0.05, 1.53)	5.53 (2.55, 11.96)	0.8 (-11.68, 13.28)	3.28 (1.44, 7.44)	0.92 (-8.8, 10.64)	7.15 (3.45, 14.82)	0.68 (-5.24, 6.6)	-	-	1.67 (0.59, 4.72)	0.93 (0.84, 1.02)	4.68 (2.36, 9.32)	0.61 (-2.06, 3.28)
**Prison category**																
A (comparator)	-		-		-		-		-		-		-		-	
B	-		-		-		-		-		-		-		-	
C	-		-		-		-		-		-		-		-	
Closed	-		-		-		-		-		-		-		-	
D	-		-		-		-		-		-		-		-	
YOI	-		-		-		-		-		-		-		-	
**Gender**																
Female (comparator)	-	0.98 (-12.63, 14.6)	-	0.97 (0.07, 1.86)	-	0.95 (-13.89, 15.8)	-	0.99 (-9.46, 11.44)	-	0.95 (-7.34, 9.23)	-		-	0.99 (0.92, 1.07)	-	0.95 (-3.22, 5.12)
Male	-	0.74 (-9.57, 11.06)	-	0.63 (0.05, 1.22)	-	0.54 (-7.81, 8.88)	-	0.78 (-7.45, 9)	-	0.28 (-2.16, 2.71)	-	0.02 (-3.65, 3.68)	-	0.81 (0.75, 0.87)	-	0.39 (-1.3, 2.08)
**Age - years**																
10 - <20	0.72 (0.06, 9.04)	0.75 (-9.68, 11.19)	0.2 (0.01, 3.33)	0.42 (-0.25, 1.1)	-		0.56 (0.05, 6.93)	0.77 (-7.37, 8.9)	-		-		0.33 (0.03, 4.32)	0.74 (0.3, 1.19)	0.84 (0.05, 13.83)	0.4 (-1.42, 2.22)
20 - <30	0.93 (0.54, 1.6)	0.79 (-10.21, 11.8)	0.68 (0.42, 1.1)	0.66 (0.05, 1.27)	0.46 (0.28, 0.76)	0.47 (-6.85, 7.79)	1.14 (0.62, 2.11)	0.87 (-8.3, 10.04)	0.74 (0.41, 1.33)	0.27 (-2.12, 2.67)	0.62 (0.04, 9.11)	0.01 (-2.3, 2.33)	1.02 (0.52, 2.01)	0.89 (0.81, 0.98)	0.8 (0.47, 1.34)	0.39 (-1.31, 2.08)
30 - <40	0.81 (0.54, 1.22)	0.77 (-9.94, 11.49)	0.5 (0.35, 0.73)	0.6 (0.04, 1.17)	0.59 (0.41, 0.85)	0.52 (-7.53, 8.56)	0.97 (0.62, 1.52)	0.85 (-8.12, 9.81)	0.57 (0.37, 0.86)	0.23 (-1.8, 2.26)	0.75 (0.12, 4.79)	0.01 (-2.72, 2.75)	0.91 (0.54, 1.53)	0.88 (0.8, 0.96)	0.85 (0.59, 1.24)	0.4 (-1.35, 2.15)
40 - <50	1.08 (0.75, 1.54)	0.82 (-10.48, 12.11)	0.62 (0.45, 0.86)	0.64 (0.05, 1.24)	0.7 (0.51, 0.95)	0.55 (-7.99, 9.09)	0.92 (0.63, 1.34)	0.84 (-8.06, 9.74)	0.54 (0.38, 0.77)	0.23 (-1.75, 2.2)	0.76 (0.16, 3.73)	0.01 (-2.77, 2.8)	0.8 (0.52, 1.24)	0.87 (0.8, 0.94)	0.79 (0.58, 1.07)	0.38 (-1.3, 2.07)
50 - <60 (comparator)	1.0	0.8 (-10.34, 11.95)	1.0	0.72 (0.05, 1.39)	1.0	0.61 (-8.96, 10.19)	1.0	0.85 (-8.16, 9.86)	1.0	0.33 (-2.53, 3.18)	1.0	0.02 (-3.5, 3.54)	1.0	0.89 (0.82, 0.96)	1.0	0.43 (-1.46, 2.32)
60 - <70	1 (0.69, 1.46)	0.8 (-10.34, 11.95)	0.76 (0.53, 1.1)	0.68 (0.05, 1.31)	0.79 (0.56, 1.11)	0.57 (-8.34, 9.48)	0.85 (0.57, 1.27)	0.83 (-7.96, 9.62)	0.9 (0.65, 1.25)	0.31 (-2.38, 3)	1.51 (0.37, 6.11)	0.03 (-4.96, 5.01)	1.01 (0.61, 1.67)	0.89 (0.82, 0.97)	0.99 (0.72, 1.35)	0.43 (-1.45, 2.3)
70 - <80	0.76 (0.47, 1.23)	0.76 (-9.8, 11.32)	0.85 (0.51, 1.42)	0.7 (0.05, 1.35)	0.78 (0.49, 1.23)	0.57 (-8.28, 9.42)	0.65 (0.39, 1.09)	0.79 (-7.59, 9.18)	0.8 (0.52, 1.24)	0.29 (-2.24, 2.81)	2.96 (0.48, 18.19)	0.04 (-8.48, 8.56)	0.82 (0.43, 1.54)	0.87 (0.78, 0.96)	0.89 (0.58, 1.35)	0.41 (-1.38, 2.2)
80 - <90	3.83 (1.08, 13.58)	0.94 (-12.02, 13.89)	1.17 (0.43, 3.12)	0.75 (0.04, 1.45)	0.73 (0.33, 1.62)	0.55 (-8.1, 9.21)	0.84 (0.34, 2.09)	0.83 (-7.94, 9.6)	0.81 (0.36, 1.85)	0.29 (-2.25, 2.83)	-		4.16 (0.54, 31.98)	0.97 (0.88, 1.06)	1.12 (0.52, 2.4)	0.45 (-1.53, 2.44)
90 - <100	-		-		-		0.48 (0.04, 5.54)	0.74 (-7.09, 8.56)	0.54 (0.05, 6.16)	0.22 (-1.77, 2.22)	-		-		2.04 (0.17, 24.05)	0.57 (-1.97, 3.1)
100 - <110	-		-		-		-		-		-		-		-	
**Length of Stay (months)**																
<1	1.62 (1.04, 2.53)	0.88 (-11.37, 13.14)	0.32 (0.22, 0.46)	0.3 (0.01, 0.6)	0.44 (0.29, 0.66)	0.28 (-4.08, 4.64)	0.95 (0.61, 1.48)	0.86 (-8.26, 9.99)	0.45 (0.25, 0.82)	0.11 (-0.82, 1.03)	1.19 (0.07, 20.67)	0.02 (-3.99, 4.03)	1.82 (1.04, 3.18)	0.92 (0.84, 1)	0.38 (0.23, 0.63)	0.13 (-0.44, 0.7)
1-<6 (comparator)	1.0	0.83 (-10.68, 12.34)	1.0	0.57 (0.04, 1.09)	1.0	0.45 (-6.56, 7.46)	1.0	0.87 (-8.32, 10.05)	1.0	0.2 (-1.51, 1.9)	1.0	0.02 (-3.41, 3.45)	1.0	0.86 (0.79, 0.94)	1.0	0.27 (-0.91, 1.45)
6-<12	0.73 (0.5, 1.08)	0.79 (-10.13, 11.7)	2.47 (1.76, 3.47)	0.76 (0.06, 1.45)	2.06 (1.49, 2.86)	0.61 (-8.91, 10.13)	0.62 (0.41, 0.94)	0.81 (-7.73, 9.35)	1.63 (1.08, 2.44)	0.27 (-2.11, 2.65)	1.53 (0.33, 7.16)	0.03 (-4.96, 5.01)	0.94 (0.59, 1.5)	0.86 (0.78, 0.93)	2.43 (1.72, 3.43)	0.46 (-1.54, 2.45)
12-<24	0.56 (0.37, 0.85)	0.74 (-9.56, 11.05)	2.76 (1.88, 4.05)	0.77 (0.06, 1.49)	2.46 (1.72, 3.53)	0.65 (-9.46, 10.75)	0.73 (0.46, 1.16)	0.83 (-7.96, 9.62)	2.29 (1.51, 3.45)	0.34 (-2.6, 3.27)	1.47 (0.29, 7.43)	0.02 (-4.79, 4.84)	0.85 (0.51, 1.42)	0.85 (0.77, 0.92)	3.07 (2.12, 4.46)	0.51 (-1.72, 2.74)
24+	0.58 (0.37, 0.9)	0.75 (-9.64, 11.14)	4.91 (3.15, 7.63)	0.86 (0.06, 1.65)	3.79 (2.55, 5.64)	0.73 (-10.69, 12.15)	0.7 (0.43, 1.13)	0.82 (-7.89, 9.54)	2.88 (1.87, 4.43)	0.38 (-2.97, 3.73)	0.42 (0.05, 3.59)	0.01 (-1.53, 1.54)	1.89 (1.05, 3.4)	0.92 (0.85, 1)	3.7 (2.5, 5.47)	0.55 (-1.87, 2.97)
**Ethnic Group**																
White (comparator)	1.0	0.81 (-10.38, 11.99)	1.0	0.67 (0.05, 1.29)	1.0	0.56 (-8.15, 9.27)	1.0	0.85 (-8.11, 9.8)	1.0	0.28 (-2.19, 2.75)	1.0	0.02 (-3.18, 3.21)	1.0	0.89 (0.82, 0.95)	1.0	0.41 (-1.39, 2.21)
Mixed	1.36 (0.45, 4.14)	0.85 (-10.9, 12.6)	0.77 (0.33, 1.77)	0.62 (0.03, 1.22)	1.4 (0.6, 3.26)	0.62 (-9.04, 10.28)	1.08 (0.36, 3.24)	0.86 (-8.19, 9.9)	1.53 (0.68, 3.44)	0.36 (-2.76, 3.47)	4.17 (0.68, 25.71)	0.05 (-10.48, 10.58)	1.61 (0.36, 7.3)	0.92 (0.8, 1.05)	0.93 (0.42, 2.08)	0.4 (-1.35, 2.14)
Asian or Asian British	0.82 (0.48, 1.38)	0.78 (-9.98, 11.54)	1.3 (0.81, 2.08)	0.71 (0.05, 1.37)	1.28 (0.81, 2.02)	0.6 (-8.81, 10.02)	0.77 (0.45, 1.31)	0.81 (-7.76, 9.38)	1.16 (0.7, 1.93)	0.31 (-2.38, 3)	0.55 (0.06, 4.95)	0.01 (-1.84, 1.86)	1.11 (0.56, 2.21)	0.89 (0.81, 0.98)	1.22 (0.76, 1.96)	0.45 (-1.52, 2.42)
Black or Black British	1.4 (0.57, 3.46)	0.85 (-10.94, 12.64)	1.51 (0.73, 3.15)	0.73 (0.05, 1.42)	1.3 (0.66, 2.59)	0.61 (-8.85, 10.06)	0.78 (0.34, 1.81)	0.81 (-7.79, 9.42)	1.13 (0.57, 2.25)	0.3 (-2.35, 2.95)	7.9 (1.07, 58.26)	0.08 (-16.65, 16.82)	1.5 (0.45, 5.04)	0.92 (0.81, 1.03)	0.87 (0.45, 1.66)	0.38 (-1.3, 2.07)
Chinese and Other	0.44 (0.16, 1.21)	0.66 (-8.53, 9.86)	0.93 (0.3, 2.86)	0.66 (0.02, 1.29)	0.88 (0.28, 2.73)	0.54 (-7.81, 8.88)	0.6 (0.19, 1.92)	0.77 (-7.41, 8.95)	2.77 (0.87, 8.85)	0.47 (-3.64, 4.58)	-		0.23 (0.08, 0.66)	0.66 (0.44, 0.88)	2.25 (0.78, 6.43)	0.57 (-1.92, 3.05)
Unclassified	0.3 (0.13, 0.69)	0.58 (-7.49, 8.65)	0.84 (0.32, 2.21)	0.64 (0.03, 1.25)	0.65 (0.27, 1.56)	0.48 (-7.01, 7.97)	0.48 (0.2, 1.17)	0.73 (-7.02, 8.49)	0.36 (0.14, 0.92)	0.15 (-1.13, 1.42)	-		0.86 (0.24, 3.12)	0.87 (0.72, 1.02)	0.9 (0.4, 2.03)	0.39 (-1.32, 2.1)

Indicator	*The proportion of people with diabetes who have had the following in the preceding 12 months: body mass index, blood pressure, record of smoking status, foot examination, urine albumin: creatinine ratio blood tests for HbA1c, cholesterol and serum creatinine*				
	**Group: Diabetes, Asthma & Epilepsy Care**				
		**Univariate**		**Multivariable**	
	**Variable**	**Odds Ratio (OR) of achieving 5 of the 8 items/tests (95% CI)**		**Odds Ratio (OR) of achieving 5 of the 8 items/tests (95% CI)**	**Probability of achieving 5 of the 8 items/tests (95% CI)**
**Year**					
	2017/18 (comparator)	1.0	-	1.0	0.52 (-0.53, 1.57)
	2018/19	1.12	(0.91, 1.39)	1.21 (0.93, 1.58)	0.55 (-0.57, 1.68)
	2019/20	1.21	(0.99, 1.49)	1.51 (1.15, 1.99)	0.6 (-0.61, 1.8)
**Prison**					
	Prison 1	6.65	(3.92, 11.27)	2.82 (1.38, 5.76)	0.71 (-0.73, 2.14)
	Prison 2	3.16	(2.21, 4.53)	1.58 (1.02, 2.44)	0.6 (-0.61, 1.81)
	Prison 3	4.63	(3.23, 6.64)	1.43 (0.88, 2.32)	0.58 (-0.59, 1.75)
	Prison 4	5.13	(3.37, 7.82)	1.38 (0.82, 2.34)	0.57 (-0.59, 1.74)
	Prison 5	4.13	(2.41, 7.08)	1.04 (0.53, 2.06)	0.52 (-0.54, 1.57)
	Prison 6	7.94	(5.16, 12.21)	2.67 (1.5, 4.75)	0.7 (-0.72, 2.11)
	Prison 7	3.63	(2.12, 6.22)	-	0.04 (-0.04, 0.12)
	Prison 8	1.35	(0.82, 2.2)	-	0.02 (-0.02, 0.07)
	Prison 9	15.32	(10.44, 22.48)	2.8 (1.64, 4.79)	0.71 (-0.72, 2.13)
	Prison 10	1.49	(1.03, 2.16)	0.78 (0.5, 1.24)	0.46 (-0.47, 1.39)
	Prison 11 (comparator)	1.0	-	1.0	0.51 (-0.52, 1.54)
	Prison 12	5.95	(1.69, 20.93)	3.04 (0.62, 15)	0.72 (-0.76, 2.2)
	Prison 13	23.55	(13.14, 42.22)	4.46 (2.13, 9.31)	0.78 (-0.8, 2.36)
**Prison category**					
	A (comparator)	1.0	-	-	-
	B	0.10	(0.07, 0.13)	-	-
	C	0.30	(0.22, 0.4)	-	-
	Closed	0.12	(0.08, 0.18)	-	-
	D	0.37	(0.26, 0.54)	-	-
	YOI	0.35	(0.1, 1.21)	-	-
**Gender**					
	Female (comparator)	1.0	-	-	-
	Male	2.15	(1.52, 3.06)	-	-
**Age - years**					
	10 - <20	0.77	(0.07, 8.58)	0.66 (0.04, 11.11)	0.42 (-0.58, 1.42)
	20 - <30	0.75	(0.5, 1.14)	0.92 (0.54, 1.57)	0.48 (-0.5, 1.47)
	30 - <40 (comparator)	1.0	-	1.0	0.5 (-0.51, 1.51)
	40 - <50	1.24	(0.92, 1.67)	1.19 (0.82, 1.73)	0.53 (-0.55, 1.61)
	50 - <60	2.14	(1.6, 2.84)	1.76 (1.23, 2.54)	0.61 (-0.62, 1.83)
	60 - <70	2.45	(1.79, 3.34)	1.39 (0.93, 2.09)	0.56 (-0.58, 1.7)
	70 - <80	2.31	(1.55, 3.45)	1.26 (0.76, 2.07)	0.54 (-0.56, 1.65)
	80 - <90	3.06	(1.48, 6.35)	2.02 (0.85, 4.79)	0.63 (-0.65, 1.92)
	90 - <100	-	-	-	-
	100 - <110	-	-	-	-
**Length of Stay (months)**					
	<1	0.33	(0.23, 0.46)	0.38 (0.25, 0.58)	0.25 (-0.26, 0.75)
	1-<6 (comparator)	1.0	-	1.0	0.44 (-0.45, 1.34)
	6-<12	2.87	(2.18, 3.77)	2.29 (1.65, 3.19)	0.63 (-0.64, 1.9)
	12-<24	3.98	(2.98, 5.31)	2.39 (1.67, 3.42)	0.64 (-0.65, 1.93)
	24+	7.26	(5.55, 9.49)	3.41 (2.32, 5.03)	0.71 (-0.73, 2.14)
**Ethnic Group**					
	White (comparator)	1.0	-	1.0	0.55 (-0.57, 1.68)
	Mixed	1.08	(0.55, 2.14)	0.8 (0.36, 1.81)	0.51 (-0.54, 1.56)
	Asian or Asian British	1.08	(0.73, 1.6)	1.41 (0.89, 2.24)	0.62 (-0.63, 1.87)
	Black or Black British	1.00	(0.57, 1.77)	0.93 (0.47, 1.82)	0.54 (-0.56, 1.64)
	Chinese and Other	0.93	(0.38, 2.31)	0.83 (0.27, 2.52)	0.52 (-0.55, 1.59)
	Unclassified	1.00	(0.49, 2.05)	0.43 (0.18, 0.99)	0.39 (-0.42, 1.21)

Indicator ***The percentage of patients with diabetes, on the register, without moderate or severe frailty, in whom the last HbA1c is 58 mmol/mol or less in the preceding 12 months***

Group: Diabetes, Asthma & Epilepsy Care

		Univariate		Multivariable			
	**Variable**	**Odds Ratio (OR) of achieving indicator**	**(95% CI) of OR**	**OR**	**(95% CI)**	**Probability of achieving indicator**	**(95% CI) of probability**
**Year**							
	2017/18 (comparator)	1.0	-	1.0	-	0.35	-
	2018/19	1.03	(0.83, 1.29)	1.02	(0.79, 1.33)	0.36	-
	2019/20	0.99	(0.79, 1.23)	1.15	(0.88, 1.51)	0.38	-
**Prison**							
	Prison 1	3.99	(2.36, 6.72)	1.95	(0.96, 3.97)	0.57	-
	Prison 2	2.12	(1.43, 3.15)	0.92	(0.56, 1.51)	0.42	-
	Prison 3	3.06	(2.07, 4.52)	0.80	(0.47, 1.37)	0.40	-
	Prison 4	3.06	(1.96, 4.76)	0.91	(0.52, 1.61)	0.42	-
	Prison 5	3.23	(1.85, 5.66)	1.02	(0.5, 2.06)	0.44	-
	Prison 6	6.09	(3.96, 9.35)	2.13	(1.18, 3.85)	0.58	-
	Prison 7	3.23	(1.85, 5.66)	-	-	0.03	-
	Prison 8	1.76	(1.05, 2.94)	-	-	0.02	-
	Prison 9	4.61	(3.21, 6.61)	0.84	(0.49, 1.45)	0.41	-
	Prison 10	1.25	(0.82, 1.9)	0.63	(0.37, 1.07)	0.35	-
	Prison 11 (comparator)	1.0	-	1.0	-	0.44	-
	Prison 12	2.71	(0.77, 9.58)	2.39	(0.49, 11.59)	0.60	-
	Prison 13	2.26	(1.41, 3.61)	0.34	(0.17, 0.65)	0.26	-
**Prison category**							
	A (comparator)	1.0	-				
	B	0.36	(0.28, 0.47)				
	C	0.85	(0.65, 1.1)				
	Closed	0.60	(0.41, 0.86)				
	D	1.32	(0.96, 1.82)				
	YOI	0.72	(0.21, 2.48)				
**Gender**							
	Female (comparator)	1.0	-	1.0	-	0.95	-
	Male	1.21	(0.84, 1.74)	-	-	0.34	-
**Age - years**							
	10 - <20	0.81	(0.07, 8.96)	0.75	(0.04, 13.04)	0.32	-
	20 - <30	0.29	(0.18, 0.46)	0.31	(0.16, 0.58)	0.18	-
	30 - <40	0.45	(0.33, 0.62)	0.52	(0.35, 0.76)	0.26	-
	40 - <50	0.73	(0.56, 0.93)	0.84	(0.62, 1.14)	0.34	-
	50 - <60 (comparator)	1.0	-	1.0	-	0.38	-
	60 - <70	1.20	(0.93, 1.54)	1.25	(0.92, 1.69)	0.43	-
	70 - <80	1.36	(0.95, 1.95)	1.33	(0.88, 2)	0.44	-
	80 - <90	1.97	(0.91, 4.29)	2.01	(0.83, 4.86)	0.53	-
	90 - <100	-	-	-	-	-	-
	100 - <110	-	-	-	-	-	-
**Length of Stay (months)**							
	<1	0.18	(0.11, 0.3)	0.21	(0.12, 0.38)	0.08	-
	1-<6 (comparator)	1.0	-	1.0	-	0.27	-
	6-<12	2.71	(2.05, 3.6)	2.58	(1.83, 3.64)	0.47	-
	12-<24	2.69	(2.01, 3.61)	2.07	(1.42, 3.01)	0.42	-
	24+	2.68	(2.09, 3.45)	2.47	(1.68, 3.65)	0.46	-
**Ethnic Group**							
	White (comparator)	1.0	-	1.0	-	0.35	-
	Mixed	1.22	(0.62, 2.42)	1.14	(0.52, 2.51)	0.38	-
	Asian or Asian British	1.23	(0.82, 1.83)	1.39	(0.87, 2.21)	0.42	-
	Black or Black British	2.52	(1.42, 4.46)	3.08	(1.6, 5.91)	0.58	-
	Chinese and Other	0.35	(0.1, 1.19)	0.49	(0.14, 1.76)	0.23	-
	Unclassified	0.92	(0.43, 1.98)	0.84	(0.36, 1.97)	0.32	-

Indicator	*The percentage of patients with asthma, on the register, who have had an asthma review in the preceding 12 months that includes an assessment of asthma control using the 3 RCP questions*						
	**Group: Diabetes, Asthma & Epilepsy Care**						
		**Univariate**		**Multivariable**			
	**Variable**	**Odds Ratio (OR) of achieving indicator**	**(95% CI) of OR**	**OR**	**(95% CI)**	**Probability of achieving indicator**	**(95% CI) of probability**
**Year**							
	2017/18 (comparator)	1.0	-	1.0	-	0.18	(-1.1, 1.47)
	2018/19	0.26	(0.22, 0.31)	0.24	(0.2, 0.29)	0.06	(-0.37, 0.49)
	2019/20	0.14	(0.11, 0.17)	0.14	(0.11, 0.17)	0.04	(-0.23, 0.3)
**Prison**							
	Prison 1	4.82	(3.14, 7.4)	2.72	(1.68, 4.39)	0.11	(-0.64, 0.86)
	Prison 2	4.23	(2.84, 6.29)	2.38	(1.56, 3.62)	0.10	(-0.58, 0.77)
	Prison 3	1.87	(1.17, 2.99)	0.77	(0.46, 1.27)	0.04	(-0.22, 0.3)
	Prison 4	7.48	(5.03, 11.14)	2.25	(1.45, 3.47)	0.09	(-0.55, 0.74)
	Prison 5	1.80	(0.81, 3.98)	0.68	(0.3, 1.56)	0.03	(-0.2, 0.27)
	Prison 6	4.64	(3.04, 7.09)	2.04	(1.29, 3.24)	0.09	(-0.51, 0.69)
	Prison 7	8.26	(5.43, 12.57)	7.62	(0.76, 76.72)	0.22	(-1.34, 1.77)
	Prison 8	3.78	(2.45, 5.84)	4.54	(0.45, 46)	0.16	(-0.96, 1.27)
	Prison 9	18.58	(12.22, 28.25)	3.59	(2.23, 5.78)	0.13	(-0.79, 1.05)
	Prison 10	1.26	(0.8, 2.01)	0.95	(0.59, 1.53)	0.05	(-0.27, 0.36)
	Prison 11 (comparator)	1.0	-	1.0	-	0.05	(-0.28, 0.38)
	Prison 12	6.74	(4.3, 10.57)	3.27	(1.93, 5.53)	0.12	(-0.74, 0.98)
	Prison 13	28.34	(18.24, 44.04)	5.01	(3, 8.37)	0.17	(-1, 1.33)
**Prison category**							
	A (comparator)	1.0	-				
	B	0.09	(0.07, 0.12)				
	C	0.20	(0.16, 0.25)				
	Closed	0.25	(0.19, 0.32)				
	D	0.18	(0.14, 0.24)				
	YOI	0.31	(0.22, 0.43)				
**Gender**							
	Female (comparator)	1.0	-	1.0	-	0.06	(-0.34, 0.46)
	Male	0.79	(0.65, 0.96)	2.2	(0.23, 21.47)	0.10	(-0.61, 0.82)
**Age - years**							
	10 - <20	1.51	(0.94, 2.45)	1.05	(0.57, 1.93)	0.09	(-0.54, 0.72)
	20 - <30	0.75	(0.62, 0.91)	0.79	(0.63, 1)	0.07	(-0.43, 0.58)
	30 - <40	1.0	-	1.0	-	0.09	(-0.52, 0.69)
	40 - <50	1.31	(1.08, 1.58)	1.13	(0.91, 1.39)	0.10	(-0.57, 0.76)
	50 - <60 (comparator)	1.86	(1.49, 2.34)	1.48	(1.15, 1.9)	0.12	(-0.7, 0.93)
	60 - <70	3.67	(2.68, 5.04)	2.26	(1.57, 3.25)	0.16	(-0.93, 1.25)
	70 - <80	4.81	(2.84, 8.17)	4.11	(2.26, 7.46)	0.23	(-1.36, 1.81)
	80 - <90	2.30	(0.5, 10.44)	1.97	(0.41, 9.42)	0.14	(-0.86, 1.15)
	90 - <100	-	-	-	-		
	100 - <110	-	-	-	-		
**Length of Stay (months)**							
	<1	0.35	(0.24, 0.51)	0.36	(0.24, 0.53)	0.02	(-0.14, 0.18)
	1-<6 (comparator)	1.0	-	1.0	-	0.06	(-0.35, 0.47)
	6-<12	2.06	(1.65, 2.57)	1.78	(1.4, 2.25)	0.10	(-0.57, 0.76)
	12-<24	3.52	(2.85, 4.35)	2.58	(2.04, 3.27)	0.13	(-0.77, 1.02)
	24+	8.30	(6.78, 10.17)	4.06	(3.14, 5.24)	0.18	(-1.07, 1.42)
**Ethnic Group**							
	White (comparator)	1.0	-	1.0	-	0.09	(-0.54, 0.72)
	Mixed	1.50	(1.01, 2.23)	1.05	(0.68, 1.62)	0.09	(-0.56, 0.75)
	Asian or Asian British	1.55	(1.12, 2.15)	1.49	(1.04, 2.14)	0.12	(-0.73, 0.97)
	Black or Black British	0.98	(0.58, 1.63)	0.72	(0.41, 1.24)	0.07	(-0.42, 0.56)
	Chinese and Other	2.85	(1.43, 5.69)	2.21	(1.03, 4.75)	0.16	(-0.95, 1.26)
	Unclassified	2.39	(1.57, 3.65)	1.37	(0.82, 2.29)	0.11	(-0.68, 0.91)

Indicator *The percentage of patients with a stroke shown to be non-haemorrhagic, or a history of TIA, who have a record in the preceding 12 months that an anti-platelet agent, or an anti-coagulant is being taken*

Group: Cardiovascular Disease

	**Univariate**		**Multivariable**			
**Variable**	**Odds Ratio (OR) of achieving indicator**	**(95% CI) of OR**	**OR**	**(95% CI)**	**Probability of achieving indicator**	**(95% CI) of probability**
**Year**						
2017/18 (comparator)	1.0	-	1.0	-	0.79	(0.69, 0.89)
2018/19	0.90	(0.44, 1.82)	1.23	(0.34, 4.43)	0.82	(0.73, 0.9)
2019/20	0.60	(0.31, 1.17)	0.84	(0.22, 3.16)	0.77	(0.67, 0.88)
**Prison**						
Prison 1	0.43	(0.052, 3.55)	0.51	(0.01, 19.5)		
Prison 2	0.28	(0.04, 2)	0.08	(0, 1.33)		
Prison 3	0.23	(0.043, 1.17)	0.04	(0, 0.51)		
Prison 4	0.42	(0.081, 2.19)	0.11	(0.01, 1.3)		
Prison 5	-	-	-	-		
Prison 6	0.89	(0.114, 7)	0.03	(0, 0.97)		
Prison 7	0.03	(0.002, 0.47)	-	-		
Prison 8	0.28	(0.032, 2.42)	0.53	(0.02, 12.82)		
Prison 9	-	-	-	-		
Prison 10	0.73	(0.057, 9.23)	1.29	(0.06, 28.6)		
Prison 11 (comparator)	1.0	-	1.0	-		
Prison 12	-	-	-	-		
Prison 13	0.31	(0.056, 1.73)	0.20	(0.01, 3.23)		
**Prison category**						
A (comparator)	1.0	-				
B	14.65	(5.32, 40.38)				
C	8.23	(3.94, 17.2)				
Closed	3.29	(0.93, 11.66)				
D	23.96	(5.08, 113.02)				
YOI	-	-				
**Gender**						
Female (comparator)	1.0	-	-	-		
Male	1.35	(0.37, 4.96)	-	-		
**Age - years**						
10 - <20	-	-	-	-		
20 - <30	-	-	-	-		
30 - <40	0.16	(0.02, 1.71)	-	-	0.01	(-0.01, 0.04)
40 - <50	0.57	(0.22, 1.47)	0.54	(0.09, 3.26)	0.81	(0.64, 0.99)
50 - <60	0.45	(0.22, 0.91)	0.14	(0.04, 0.53)	0.61	(0.47, 0.75)
60 - <70 (comparator)	1.0	-	1.0	-	0.88	(0.8, 0.96)
70 - <80	0.99	(0.44, 2.24)	2.72	(0.26, 28.05)	0.94	(0.84, 1.04)
80 - <90	1.60	(0.41, 6.3)	0.58	(0.08, 4.22)	0.82	(0.62, 1.02)
90 - <100	-	-	-	-		
100 - <110	-	-	-	-	-	-
**Length of Stay (months)**						
<1	0.82	(0.21, 3.23)	0.21	(0.02, 2.02)	0.46	(0.09, 0.84)
1-<6 (comparator)	1.0	-	1.0	-	0.71	(0.52, 0.91)
6-<12	4.97	(1.01, 24.57)	-	-		
12-<24	1.71	(0.61, 4.8)	32.66	(1.39, 769.61)	0.96	(0.92, 1.01)
24+	0.42	(0.19, 0.92)	1.40	(0.3, 6.6)	0.76	(0.67, 0.85)
**Ethnic Group**						
White (comparator)	1.0	-	1.0	-	0.80	(0.75, 0.86)
Mixed	-	-	-	-		
Asian or Asian British	1.84	(0.19, 18.34)	1,281,697.43	-	0.98	(0.96, 1)
Black or Black British	-	-	-	-		
Chinese and Other	-	-	-	-		
Unclassified	0.15	(0.02, 1.54)	0.04	(0.002, 0.59)	0.31	(-0.07, 0.7)

Indicator	In those patients with atrial fibrillation with a record of a CHA2DS2-VASc score of 2 or more, the percentage of patients who are currently treated with anticoagulation drug therapy.						
	Group: Cardiovascular Disease						
		Univariate		Multivariable			
	Variable	Odds Ratio (OR) of achieving indicator	(95% CI) of OR	OR	(95% CI)	Probability of achieving indicator	(95% CI) of probability
**Year**							
	2017/18 (comparator)	1.0	-	-	-	-	-
	2018/19	0.60	(0.2, 1.78)	-	-	-	-
	2019/20	0.88	(0.29, 2.72)	-	-	-	-
**Prison**							
	Prison 1	-	-	-	-	-	-
	Prison 2	1.36	(0.22, 8.52)	-	-	-	-
	Prison 3	-	-	-	-	-	-
	Prison 4	1.95	(0.35, 10.95)	-	-	-	-
	Prison 5	2.71	(0.21, 34.5)	-	-	-	-
	Prison 6	-	-	-	-	-	-
	Prison 7	-	-	-	-	-	-
	Prison 8	0.66	(0.07, 6.38)	-	-	-	-
	Prison 9	1.11	(0.21, 5.77)	-	-	-	-
	Prison 10	-	-	-	-	-	-
	Prison 11 (comparator)	1.0	-	-	-	-	-
	Prison 12	-	-	-	-	-	-
	Prison 13	2.21	(0.34, 14.44)	-	-	-	-
**Prison category**							
	A (comparator)	1.0	-	-	-	-	-
	B	1.15	(0.39, 3.34)	-	-	-	-
	C	2.40	(0.77, 7.55)	-	-	-	-
	Closed	0.75	(0.13, 4.5)	-	-	-	-
	D	4.09	(0.47, 35.18)	-	-	-	-
	YOI	-	-	-	-	-	-
**Gender**							
	Female (comparator)	1.0	-	-	-	-	-
	Male	2.46	(0.41, 14.62)	-	-	-	-
**Age - years**							
	10 - <20	-	-	-	-	-	-
	20 - <30	-	-	-	-	-	-
	30 - <40	-	-	-	-	-	-
	40 - <50	-	-	-	-	-	-
	50 - <60 (comparator)	1.0	-	-	-	-	-
	60 - <70	2.09	(0.7, 6.27)	-	-	-	-
	70 - <80	2.16	(0.65, 7.18)	-	-	-	-
	80 - <90	3.66	(0.39, 34.14)	-	-	-	-
	90 - <100	0.40	(0.05, 3.41)	-	-	-	-
	100 - <110	-	-	-	-	-	-
**Length of Stay (months)**							
	<1	0.75	(0.06, 9.53)	-	-	-	-
	1-<6 (comparator)	1.0	-	-	-	-	-
	6-<12	3.04	(0.28, 33.3)	-	-	-	-
	12-<24	3.62	(0.54, 24.5)	-	-	-	-
	24+	0.52	(0.13, 2.05)	-	-	-	-
**Ethnic Group**							
	White (comparator)	-	-	-	-	-	-
	Mixed	-	-	-	-	-	-
	Asian or Asian British	-	-	-	-	-	-
	Black or Black British	-	-	-	-	-	-
	Chinese and Other	-	-	-	-	-	-
	Unclassified	-	-	-	-	-	-

Indicator	*The percentage of patients with atrial fibrillation in whom stroke risk has been assessed using the CHA2DS2-VASc score risk stratification scoring system in the preceding 12 months (excluding those patients with a previous CHADS2 or CHA2DS2-VASc score of 2 or more)*						
	**Group: Cardiovascular Disease**						
		**Univariate**		**Multivariable**			
	**Variable**	**Odds Ratio (OR) of achieving indicator**	**(95% CI) of OR**	**OR**	**(95% CI)**	**Probability of achieving indicator**	**(95% CI) of probability**
**Year**							
	2017/18 (comparator)	1.0	-	1.0	-	0.36	(-28.31, 29.03)
	2018/19	2.34	(0.69, 7.87)	2.58	(0.57, 11.57)	0.54	(-43.09, 44.17)
	2019/20	3.21	(0.97, 10.69)	5.17	(1.02, 26.2)	0.67	(-53.46, 54.8)
**Prison**							
	Prison 1	0.67	(0.02, 28.58)	2.77	(0.01, 620.43)		
	Prison 2	0.63	(0.03, 11.5)	1.11	(0.02, 55.53)		
	Prison 3	-	-	-	-		
	Prison 4	0.17	(0.01, 2.71)	0.15	(0.01, 4.33)		
	Prison 5	-	-	-	-		
	Prison 6	0.26	(0.02, 4.62)	0.21	(0, 14.91)		
	Prison 7	-	-	-	-		
	Prison 8	-	-	-	-		
	Prison 9	0.87	(0.06, 12.79)	1.80	(0.05, 58.91)		
	Prison 10	0.67	(0.02, 28.58)	1.77	(0.01, 465.58)		
	Prison 11 (comparator)	1.0	-	1.0	-		
	Prison 12	-	-	-	-		
	Prison 13	1.55	(0.08, 29.79)	5.14	(0.1, 276.4)		
**Prison category**							
	A (comparator)	1.0	-	-	-	-	-
	B	0.29	(0.07, 1.17)	-	-	-	-
	C	0.19	(0.05, 0.74)	-	-	-	-
	Closed	0.95	(0.07, 12.97)	-	-	-	-
	D	0.22	(0.04, 1.15)	-	-	-	-
	YOI	-	-	-	-	-	-
**Gender**							
	Female (comparator)	1.0	-	-	-	-	-
	Male	0.42	(0.03, 5.29)	-	-	-	-
**Age - years**							
	10 - <20	-	-	-	-	-	-
	20 - <30	0.54	(0.03, 10.66)	-	-	-	-
	30 - <40	0.51	(0.11, 2.31)	2.25	(0.09, 57.94)	0.52	(-41.79, 42.84)
	40 - <50	0.53	(0.07, 4.19)	13.68	(0.24, 787.79)	0.80	(-63.94, 65.55)
	50 - <60	0.63	(0.15, 2.7)	3.45	(0.28, 42.73)	0.60	(-47.64, 48.83)
	60 - <70 (comparator)	1.0	-	1.0	-	0.39	(-30.72, 31.49)
	70 - <80	0.99	(0.26, 3.78)	3.52	(0.4, 31.05)	0.60	(-47.89, 49.09)
	80 - <90	-	-	-	-	-	-
	90 - <100	-	-	-	-	-	-
	100 - <110	-	-	-	-	-	-
**Length of Stay (months)**							
	<1	-	-	-	-	-	-
	1-<6 (comparator)	-	-	-	-	0.36	(-29.06, 29.79)
	6-<12	1.79	(0.24, 13.23)	6.15	(0.07, 531.88)	0.68	(-54.14, 55.5)
	12-<24	2.84	(0.54, 14.99)	2.84	(0.12, 67.51)	0.55	(-43.97, 45.07)
	24+	2.42	(0.58, 10.06)	2.62	(0.12, 58.02)	0.54	(-42.83, 43.9)
**Ethnic Group**							
	White (comparator)	-					
	Mixed	-					
	Asian or Asian British	-					
	Black or Black British	-					
	Chinese and Other	-					
	Unclassified	-					

Indicator	*The proportion of people aged 79 years or under, with coronary heart disease, hypertension, stroke, transient ischaemic attack or peripheral arterial disease, who have had a blood pressure of 140/90mmHg or less in the preceding 12 months*					
	**Group: Cardiovascular Disease**					
	**Univariate**		**Multivariable**			
**Variable**	**Odds Ratio (OR) of achieving indicator**	**(95% CI) of OR**	**OR**	**(95% CI)**	**Probability of achieving indicator**	**(95% CI) of probability**
**Year**						
2017/18 (comparator)	1.0	-	1.0	-	0.48	(-0.68, 1.63)
2018/19	0.97	(0.82, 1.15)	0.90	(0.75, 1.09)	0.45	(-0.64, 1.55)
2019/20	0.91	(0.77, 1.07)	0.94	(0.78, 1.13)	0.46	(-0.66, 1.58)
**Prison**						
Prison 1	2.57	(1.81, 3.66)	2.72	(1.76, 4.22)	0.58	(-0.82, 1.98)
Prison 2	1.20	(0.87, 1.64)	1.09	(0.76, 1.56)	0.36	(-0.51, 1.23)
Prison 3	1.56	(1.14, 2.15)	1.46	(0.99, 2.15)	0.43	(-0.61, 1.46)
Prison 4	2.45	(1.86, 3.23)	1.98	(1.39, 2.83)	0.50	(-0.71, 1.71)
Prison 5	3.18	(2.08, 4.87)	3.25	(1.94, 5.45)	0.62	(-0.88, 2.12)
Prison 6	3.49	(2.51, 4.86)	3.42	(2.28, 5.12)	0.63	(-0.9, 2.16)
Prison 7	2.56	(1.71, 3.85)	1.08	(0.15, 7.98)	0.36	(-0.6, 1.31)
Prison 8	5.20	(3.54, 7.64)	3.00	(0.39, 22.95)	0.60	(-0.92, 2.12)
Prison 9	1.55	(1.16, 2.07)	1.09	(0.73, 1.63)	0.36	(-0.51, 1.23)
Prison 10	2.81	(2.12, 3.74)	2.48	(1.79, 3.44)	0.56	(-0.79, 1.9)
Prison 11 (comparator)	1.0	-	1.0	-	0.34	(-0.48, 1.16)
Prison 12	2.41	(0.33, 17.29)	0.52	(0.04, 6.2)	0.21	(-0.44, 0.87)
Prison 13	1.83	(1.31, 2.56)	1.38	(0.89, 2.16)	0.41	(-0.59, 1.42)
**Prison category**						
A (comparator)	1.0	-				
B	0.95	(0.78, 1.15)				
C	1.32	(1.08, 1.62)				
Closed	2.31	(1.74, 3.06)				
D	2.06	(1.58, 2.68)				
YOI	1.47	(0.21, 10.5)				
**Gender**						
Female (comparator)	1.0	-	1.0	-	0.56	(-0.86, 1.98)
Male	0.50	(0.39, 0.66)	0.6	(0.09, 4.79)	0.46	(-0.65, 1.56)
**Age - years**						
10 - <20	-	-	-	-	-	-
20 - <30	1.45	(1.01, 2.09)	1.88	(1.23, 2.86)	0.60	(-0.86, 2.06)
30 - <40	1.26	(1.02, 1.56)	1.36	(1.06, 1.74)	0.53	(-0.75, 1.8)
40 - <50	0.98	(0.82, 1.17)	1.00	(0.82, 1.23)	0.45	(-0.65, 1.56)
50 - <60 (comparator)	1.0	-	1.0	-	0.45	(-0.65, 1.55)
60 - <70	0.92	(0.75, 1.12)	0.91	(0.73, 1.15)	0.43	(-0.61, 1.48)
70 - <80	0.87	(0.66, 1.16)	0.87	(0.63, 1.2)	0.42	(-0.6, 1.44)
80 - <90	-	-	-	-	-	-
90 - <100	-	-	-	-	-	-
100 - <110	-	-	-	-	-	-
**Length of Stay (months)**						
<1	0.78	(0.62, 0.98)	0.97	(0.73, 1.27)	0.44	(-0.63, 1.51)
1-<6 (comparator)	1.0	-	1.0	-	0.45	(-0.64, 1.53)
6-<12	0.96	(0.78, 1.19)	0.88	(0.68, 1.13)	0.42	(-0.6, 1.43)
12-<24	1.02	(0.83, 1.26)	0.93	(0.72, 1.2)	0.43	(-0.61, 1.47)
24+	1.01	(0.84, 1.22)	1.42	(1.08, 1.87)	0.53	(-0.75, 1.81)
**Ethnic Group**						
White (comparator)	1.0	-	1.0	-	0.46	(-0.66, 1.59)
Mixed	0.63	(0.31, 1.27)	0.56	(0.27, 1.17)	0.33	(-0.49, 1.16)
Asian or Asian British	2.05	(1.3, 3.26)	1.76	(1.09, 2.84)	0.60	(-0.85, 2.04)
Black or Black British	0.70	(0.4, 1.25)	0.75	(0.41, 1.37)	0.40	(-0.57, 1.37)
Chinese and Other	0.90	(0.42, 1.94)	0.96	(0.43, 2.11)	0.45	(-0.66, 1.57)
Unclassified	0.71	(0.41, 1.22)	0.80	(0.44, 1.46)	0.41	(-0.59, 1.42)

Indicator		The percentage of patients who had a myocardial infarction who are currently being treated with ACE-I (or ARB if ACE-I intolerant), anti-platelet therapy, beta-blocker and a statin							
	Group: Cardiovascular Disease								
		Univariate: Odds ratios (95% CI)							
	Variable	ACEI		Anti Platelet		Beta Blocker		Statin	
**Year**									
	2017/18 (comparator)	1.0	-	1.0	-	1.0	-	1.0	-
	2018/19	1.10	(0.82, 1.47)	1.01	(0.76, 1.35)	1.00	(0.75, 1.33)	0.75	(0.54, 1.02)
	2019/20	1.19	(0.9, 1.58)	1.09	(0.82, 1.44)	1.04	(0.79, 1.37)	0.83	(0.61, 1.13)
**Prison**									
	Prison 1	0.19	(0.07, 0.57)	1.12	(0.56, 2.25)	0.75	(0.38, 1.49)	0.81	(0.4, 1.63)
	Prison 2	1.44	(0.92, 2.27)	1.68	(1.06, 2.65)	0.84	(0.54, 1.31)	1.05	(0.66, 1.67)
	Prison 3	0.83	(0.5, 1.38)	1.08	(0.67, 1.74)	1.04	(0.64, 1.68)	1.57	(0.93, 2.68)
	Prison 4	1.70	(1.12, 2.59)	2.05	(1.33, 3.15)	1.39	(0.91, 2.12)	2.14	(1.33, 3.44)
	Prison 5	0.81	(0.36, 1.82)	1.41	(0.65, 3.04)	1.44	(0.66, 3.17)	1.19	(0.53, 2.68)
	Prison 6	1.28	(0.77, 2.13)	0.53	(0.32, 0.87)	0.73	(0.44, 1.19)	1.32	(0.77, 2.26)
	Prison 7	0.72	(0.28, 1.83)	0.60	(0.25, 1.41)	0.54	(0.23, 1.27)	0.45	(0.19, 1.06)
	Prison 8	0.79	(0.41, 1.51)	0.87	(0.48, 1.59)	0.34	(0.18, 0.64)	0.65	(0.35, 1.2)
	Prison 9	1.64	(1.08, 2.51)	2.29	(1.47, 3.58)	1.14	(0.75, 1.74)	2.51	(1.53, 4.12)
	Prison 10	1.25	(0.79, 1.99)	0.79	(0.51, 1.25)	0.92	(0.58, 1.45)	0.76	(0.48, 1.22)
	Prison 11 (comparator)	1.0	-	1.0	-	1.0	-	1.0	-
	Prison 12	-	-	0.84	(0.05, 13.74)	0.76	(0.05, 12.4)	-	-
	Prison 13	1.33	(0.82, 2.16)	1.62	(0.99, 2.65)	1.24	(0.76, 2.01)	2.21	(1.26, 3.89)
**Prison category**									
	A (comparator)	1.0	-	1.0	-	1.0	-	1.0	-
	B	0.79	(0.58, 1.07)	0.55	(0.4, 0.75)	0.78	(0.58, 1.06)	0.39	(0.27, 0.56)
	C	0.73	(0.53, 1.02)	0.76	(0.54, 1.07)	0.99	(0.71, 1.37)	0.70	(0.47, 1.04)
	Closed	0.51	(0.3, 0.87)	0.39	(0.23, 0.65)	0.33	(0.2, 0.56)	0.24	(0.14, 0.42)
	D	0.76	(0.49, 1.16)	0.34	(0.22, 0.53)	0.73	(0.48, 1.12)	0.54	(0.33, 0.88)
	YOI	-	-	0.42	(0.03, 6.9)	0.64	(0.04, 10.41)	-	-
**Gender**									
	Female (comparator)	1.0	-	1.0	-	1.0	-	1.0	-
	Male	1.48	(0.87, 2.51)	1.62	(0.99, 2.67)	2.73	(1.62, 4.62)	2.24	(1.36, 3.7)
**Age - years**									
	10 - <20	-	-	-	-	-	-	-	-
	20 - <30	0.11	(0.02, 0.46)	0.16	(0.06, 0.42)	0.27	(0.11, 0.67)	0.04	(0.01, 0.15)
	30 - <40	0.34	(0.2, 0.58)	0.11	(0.06, 0.19)	0.33	(0.21, 0.53)	0.09	(0.06, 0.16)
	40 - <50	0.59	(0.42, 0.84)	0.49	(0.35, 0.69)	0.63	(0.45, 0.88)	0.46	(0.32, 0.66)
	50 - <60	1.0	-	1.0	-	1.0	-	1.0	-
	60 - <70 (comparator)	0.97	(0.73, 1.29)	1.16	(0.85, 1.57)	1.05	(0.78, 1.4)	1.26	(0.89, 1.79)
	70 - <80	0.89	(0.6, 1.33)	0.82	(0.55, 1.23)	1.13	(0.75, 1.69)	1.32	(0.8, 2.16)
	80 - <90	0.51	(0.24, 1.11)	0.87	(0.41, 1.81)	1.33	(0.63, 2.81)	1.38	(0.55, 3.43)
	90 - <100	-	-	-	-	-	-	-	-
	100 - <110	-	-	-	-	-	-	-	-
**Length of Stay (months)**									
	<1	0.55	(0.36, 0.83)	0.54	(0.37, 0.79)	0.54	(0.37, 0.79)	0.63	(0.43, 0.93)
	1-<6 (comparator)	1.0	-	1.0	-	1.0	-	1.0	-
	6-<12	0.89	(0.62, 1.29)	1.08	(0.75, 1.54)	1.03	(0.72, 1.47)	1.43	(0.98, 2.09)
	12-<24	1.24	(0.87, 1.78)	0.94	(0.65, 1.34)	1.03	(0.72, 1.48)	1.28	(0.88, 1.88)
	24+	1.35	(0.99, 1.83)	1.93	(1.4, 2.65)	1.02	(0.75, 1.38)	3.02	(2.1, 4.33)
**Ethnic Group**									
	White (comparator)	1.0	-	1.0	-	1.0	-	1.0	-
	Mixed	1.49	(0.55, 4.01)	0.49	(0.18, 1.34)	0.27	(0.09, 0.84)	0.43	(0.16, 1.16)
	Asian or Asian British	0.59	(0.29, 1.21)	0.69	(0.36, 1.33)	1.22	(0.63, 2.38)	1.39	(0.65, 2.99)
	Black or Black British	2.10	(0.84, 5.28)	0.71	(0.28, 1.75)	1.74	(0.65, 4.6)	0.72	(0.28, 1.84)
	Chinese and Other	0.23	(0.03, 1.85)	1.07	(0.25, 4.51)	1.34	(0.32, 5.63)	0.40	(0.1, 1.62)
	Unclassified	2.39	(1.06, 5.39)	0.51	(0.23, 1.13)	0.87	(0.39, 1.93)	2.13	(0.72, 6.28)

Indicator	*The percentage of patients who had a myocardial infarction who are currently being treated with ACE-I (or ARB if ACE-I intolerant), anti-platelet therapy, beta-blocker and a statin*							
**Domain: Routine primary care**								
	**Multivariable**							
**Variable**	**ACEI**		**Anti Platelet**		**Beta Blocker**		**Statin**	
	**OR (95% CI)**	**Probability of achieving indicator (95% CI)**	**OR (95% CI)**	**Probability of achieving indicator (95% CI)**	**OR (95% CI)**	**Probability of achieving indicator (95% CI)**	**OR (95% CI)**	**Probability of achieving indicator (95% CI)**
**Year**								
2017/18 (comparator)	1.0	0.35 (-1.25, 1.96)	1.0	0.58 (-0.64, 1.8)	1.0	0.55 (-2.15, 3.25)	1.0	0.71 (-15.5, 16.92)
2018/19	1.32 (0.95, 1.82)	0.41 (-1.47, 2.29)	1.15 (0.82, 1.61)	0.61 (-0.67, 1.89)	1.04 (0.76, 1.43)	0.56 (-2.19, 3.3)	0.97 (0.67, 1.41)	0.7 (-15.4, 16.8)
2019/20	1.47 (1.07, 2.02)	0.44 (-1.55, 2.43)	1.24 (0.89, 1.74)	0.62 (-0.69, 1.94)	1.09 (0.8, 1.49)	0.57 (-2.23, 3.36)	1.04 (0.72, 1.51)	0.71 (-15.66, 17.09)
**Prison**								
Prison 1	0.17 (0.05, 0.53)	0.1 (-0.35, 0.55)	0.76 (0.33, 1.76)	0.56 (-0.63, 1.76)	0.63 (0.28, 1.38)	0.45 (-1.79, 2.7)	0.66 (0.27, 1.57)	0.62 (-13.55, 14.78)
Prison 2	1.39 (0.81, 2.39)	0.44 (-1.57, 2.45)	1.13 (0.65, 1.98)	0.64 (-0.71, 1.99)	0.71 (0.42, 1.2)	0.48 (-1.9, 2.87)	0.83 (0.47, 1.49)	0.66 (-14.46, 15.78)
Prison 3	0.75 (0.4, 1.4)	0.31 (-1.1, 1.72)	0.62 (0.34, 1.15)	0.52 (-0.58, 1.63)	0.79 (0.43, 1.43)	0.51 (-1.99, 3)	1.09 (0.55, 2.16)	0.7 (-15.37, 16.78)
Prison 4	1.47 (0.83, 2.6)	0.45 (-1.61, 2.51)	1.13 (0.62, 2.07)	0.64 (-0.71, 1.99)	1.14 (0.65, 2.02)	0.59 (-2.31, 3.49)	1.17 (0.61, 2.24)	0.71 (-15.61, 17.03)
Prison 5	0.56 (0.22, 1.45)	0.26 (-0.92, 1.43)	0.61 (0.24, 1.53)	0.52 (-0.59, 1.62)	1.25 (0.48, 3.25)	0.61 (-2.39, 3.6)	0.64 (0.24, 1.67)	0.61 (-13.43, 14.65)
Prison 6	1.07 (0.55, 2.05)	0.38 (-1.36, 2.13)	0.25 (0.13, 0.49)	0.33 (-0.37, 1.03)	0.59 (0.31, 1.12)	0.44 (-1.73, 2.61)	1.12 (0.54, 2.32)	0.71 (-15.47, 16.89)
Prison 7	2270222.54 (0, 0)	0.97 (-3.43, 5.37)	190089.57 (0, 0)	0.96 (-1.07, 3)	575519.7 (0, 0)	0.97 (-3.81, 5.75)	22348.3 (0, 0)	0.97 (-21.17, 23.1)
Prison 8	1228954.86 (0, 0)	0.96 (-3.41, 5.33)	420240.76 (0, 0)	0.97 (-1.08, 3.02)	320671.23 (0, 0)	0.96 (-3.78, 5.71)	46751.94 (0, 0)	0.97 (-21.34, 23.29)
Prison 9	1.26 (0.68, 2.34)	0.42 (-1.49, 2.33)	0.99 (0.52, 1.9)	0.61 (-0.68, 1.91)	1.12 (0.61, 2.07)	0.58 (-2.3, 3.46)	1.2 (0.58, 2.47)	0.72 (-15.69, 17.13)
Prison 10	1.42 (0.82, 2.48)	0.44 (-1.58, 2.47)	0.63 (0.36, 1.1)	0.52 (-0.58, 1.63)	0.81 (0.47, 1.39)	0.51 (-2.02, 3.04)	0.63 (0.35, 1.14)	0.61 (-13.41, 14.64)
Prison 11 (comparator)	1.0	0.37 (-1.31, 2.05)	1.0	0.62 (-0.69, 1.92)	1.0	0.56 (-2.2, 3.32)	1.0	0.69 (-15.1, 16.48)
Prison 12	-		3.21 (0.16, 65.61)	0.79 (-0.91, 2.49)	1.92 (0.1, 38.08)	0.69 (-2.75, 4.13)	-	
Prison 13	0.79 (0.4, 1.57)	0.32 (-1.14, 1.78)	0.95 (0.47, 1.93)	0.61 (-0.68, 1.89)	1.02 (0.53, 1.98)	0.56 (-2.22, 3.35)	0.84 (0.38, 1.81)	0.66 (-14.47, 15.79)
**Prison category**								
A (comparator)								
A/B								
B								
C								
Closed								
D								
YOI								
**Gender**								
Female (comparator)	1.0	0.02 (-0.06, 0.1)	1.0	0.03 (-0.03, 0.09)	1.0	0.02 (-0.08, 0.12)	1.0	0.03 (-0.68, 0.74)
Male	1721535.63 (0, 0)	0.44 (-1.58, 2.46)	602493.15 (0, 0)	0.63 (-0.7, 1.97)	829669.15 (0, 0)	0.59 (-2.34, 3.53)	68865.26 (0, 0)	0.74 (-16.12, 17.59)
**Age - years**								
10 - <20	-		-		-		-	
20 - <30	0.15 (0.03, 0.66)	0.12 (-0.44, 0.68)	0.1 (0.03, 0.33)	0.21 (-0.27, 0.69)	0.2 (0.07, 0.57)	0.26 (-1.03, 1.55)	0.03 (0.01, 0.16)	0.13 (-2.83, 3.09)
30 - <40	0.36 (0.2, 0.65)	0.24 (-0.87, 1.35)	0.09 (0.05, 0.17)	0.19 (-0.22, 0.59)	0.27 (0.16, 0.47)	0.32 (-1.28, 1.92)	0.09 (0.05, 0.17)	0.27 (-5.81, 6.34)
40 - <50	0.64 (0.44, 0.96)	0.36 (-1.28, 1.99)	0.5 (0.33, 0.73)	0.54 (-0.6, 1.68)	0.55 (0.38, 0.8)	0.48 (-1.91, 2.87)	0.52 (0.34, 0.78)	0.65 (-14.24, 15.54)
50 - <60 (comparator)	1.0	0.46 (-1.63, 2.54)	1.0	0.69 (-0.77, 2.15)	1.0	0.63 (-2.46, 3.71)	1.0	0.77 (-16.97, 18.51)
60 - <70	0.88 (0.64, 1.23)	0.43 (-1.53, 2.39)	0.96 (0.67, 1.36)	0.68 (-0.76, 2.12)	0.79 (0.57, 1.09)	0.57 (-2.25, 3.39)	1.04 (0.7, 1.54)	0.78 (-17.11, 18.67)
70 - <80	0.78 (0.5, 1.2)	0.4 (-1.43, 2.23)	0.63 (0.4, 0.99)	0.59 (-0.66, 1.84)	0.9 (0.58, 1.39)	0.6 (-2.36, 3.56)	1 (0.58, 1.72)	0.77 (-16.97, 18.52)
80 - <90	0.5 (0.22, 1.13)	0.3 (-1.09, 1.7)	0.61 (0.28, 1.35)	0.59 (-0.66, 1.83)	1.05 (0.48, 2.29)	0.64 (-2.5, 3.78)	1.07 (0.41, 2.79)	0.79 (-17.21, 18.78)
90 - <100	-		-		-		-	
100 - <110	-		-		-		-	
**Length of Stay (months)**								
<1	0.45 (0.27, 0.75)	0.25 (-0.91, 1.42)	0.42 (0.26, 0.68)	0.42 (-0.47, 1.31)	0.41 (0.26, 0.66)	0.43 (-1.68, 2.53)	0.48 (0.3, 0.79)	0.54 (-11.84, 12.92)
1-<6 (comparator)	1.0	0.42 (-1.49, 2.32)	1.0	0.61 (-0.68, 1.89)	1.0	0.63 (-2.48, 3.74)	1.0	0.69 (-15.1, 16.48)
6-<12	0.96 (0.62, 1.48)	0.41 (-1.45, 2.27)	1.38 (0.88, 2.17)	0.67 (-0.75, 2.09)	0.95 (0.62, 1.45)	0.62 (-2.44, 3.67)	1.76 (1.07, 2.89)	0.78 (-17.12, 18.69)
12-<24	1.24 (0.79, 1.93)	0.47 (-1.66, 2.59)	0.78 (0.49, 1.24)	0.56 (-0.62, 1.73)	0.74 (0.48, 1.16)	0.56 (-2.22, 3.35)	0.81 (0.5, 1.32)	0.65 (-14.23, 15.52)
24+	0.98 (0.63, 1.53)	0.41 (-1.47, 2.29)	1.28 (0.8, 2.06)	0.66 (-0.73, 2.05)	0.62 (0.4, 0.97)	0.52 (-2.05, 3.09)	1.75 (1.03, 2.98)	0.78 (-17.11, 18.67)
**Ethnic Group**								
White (comparator)	1.0	0.4 (-1.41, 2.21)	1.0	0.61 (-0.68, 1.9)	1.0	0.55 (-2.18, 3.29)	1.0	0.71 (-15.5, 16.91)
Mixed	1.38 (0.47, 4.06)	0.47 (-1.69, 2.63)	0.96 (0.29, 3.11)	0.6 (-0.69, 1.89)	0.41 (0.12, 1.37)	0.35 (-1.39, 2.09)	0.35 (0.1, 1.2)	0.5 (-11, 12)
Asian or Asian British	0.61 (0.28, 1.34)	0.3 (-1.06, 1.65)	1.14 (0.54, 2.42)	0.64 (-0.71, 1.99)	1.54 (0.76, 3.13)	0.65 (-2.56, 3.87)	2.56 (1.05, 6.25)	0.84 (-18.33, 20.01)
Black or Black British	2.5 (0.94, 6.63)	0.6 (-2.16, 3.37)	0.53 (0.2, 1.4)	0.48 (-0.55, 1.5)	1.68 (0.61, 4.62)	0.67 (-2.64, 3.98)	0.59 (0.21, 1.67)	0.61 (-13.35, 14.57)
Chinese and Other	0.35 (0.04, 2.93)	0.2 (-0.75, 1.14)	2.86 (0.43, 18.89)	0.79 (-0.9, 2.48)	2.35 (0.48, 11.47)	0.73 (-2.9, 4.37)	0.84 (0.16, 4.26)	0.68 (-14.82, 16.17)
Unclassified	2.88 (1.21, 6.88)	0.64 (-2.27, 3.54)	0.29 (0.12, 0.71)	0.35 (-0.41, 1.12)	0.75 (0.32, 1.77)	0.49 (-1.93, 2.91)	1.09 (0.34, 3.42)	0.72 (-15.8, 17.24)

Indicator		The proportion of people who have had a myocardial infarction and are have received a prescription for an ACE-inhibitor or angiotensin receptor blocker, anti-platelet therapy, beta-blocker and a statin in the preceding 12 months			
	Group: Cardiovascular Disease				
		Univariate		Multivariable	
	Variable	Odds Ratio (OR) of satisying 3 out of 4 classes of drugs (95% CI)		Odds Ratio (OR) of satisying 3 out of 4 classes of drugs (95% CI)	Probability of satisying 3 out of 4 classes of drugs (95% CI)
**Year**					
	2017/18 (comparator)	1.0	-	1.0	0.48 (-7.32, 8.28)
	2018/19	1.04	(0.78, 1.38)	1.25 (0.9, 1.72)	0.53 (-8.07, 9.13)
	2019/20	1.04	(0.79, 1.37)	1.32 (0.96, 1.81)	0.54 (-8.25, 9.34)
**Prison**					
	Prison 1	0.73	(0.36, 1.47)	0.58 (0.25, 1.33)	0.38 (-5.82, 6.59)
	Prison 2	1.66	(1.07, 2.6)	1.33 (0.77, 2.27)	0.56 (-8.52, 9.64)
	Prison 3	1.11	(0.69, 1.79)	0.72 (0.39, 1.32)	0.43 (-6.55, 7.41)
	Prison 4	1.98	(1.3, 3.01)	1.29 (0.73, 2.29)	0.56 (-8.43, 9.54)
	Prison 5	1.21	(0.57, 2.57)	0.85 (0.35, 2.08)	0.47 (-7.08, 8.01)
	Prison 6	0.95	(0.57, 1.56)	0.68 (0.35, 1.3)	0.42 (-6.34, 7.17)
	Prison 7	0.73	(0.3, 1.75)	299317.88 (0, 0)	0.97 (-14.71, 16.65)
	Prison 8	0.59	(0.31, 1.11)	145066.69 (0, 0)	0.96 (-14.58, 16.5)
	Prison 9	1.99	(1.3, 3.03)	1.15 (0.62, 2.13)	0.53 (-8.07, 9.13)
	Prison 10	1.01	(0.64, 1.59)	0.91 (0.52, 1.58)	0.48 (-7.29, 8.25)
	Prison 11 (comparator)	1.0	-	1.0	0.5 (-7.62, 8.62)
	Prison 12	-	-	-	
	Prison 13	1.84	(1.14, 2.98)	0.97 (0.49, 1.89)	0.49 (-7.5, 8.49)
**Prison category**					
	A (comparator)	1.0	-	-	-
	B	0.61	(0.45, 0.82)	-	-
	C	0.75	(0.54, 1.04)	-	-
	Closed	0.33	(0.19, 0.55)	-	-
	D	0.52	(0.34, 0.8)	-	-
	YOI	-	-	-	-
**Gender**					
	Female (comparator)	1.0	-	-	-
	Male	2.10	(1.25, 3.53)	-	-
**Age - years**					
	10 - <20	-	-	-	-
	20 - <30	0.44	(0.09, 2.04)	0.28 (0.03, 2.33)	0.06 (-0.89, 1.01)
	30 - <40 (comparator)	1.0	-	1.0	0.18 (-2.69, 3.04)
	40 - <50	3.67	(2, 6.72)	4.41 (2.21, 8.79)	0.47 (-7.17, 8.12)
	50 - <60	6.77	(3.82, 12)	7.58 (3.91, 14.72)	0.6 (-9.11, 10.31)
	60 - <70	7.66	(4.28, 13.7)	6.87 (3.52, 13.43)	0.58 (-8.77, 9.92)
	70 - <80	5.29	(2.79, 10.04)	5.03 (2.41, 10.46)	0.5 (-7.65, 8.65)
	80 - <90	5.79	(2.42, 13.85)	4.87 (1.87, 12.68)	0.5 (-7.53, 8.52)
	90 - <100	-	-	-	-
	100 - <110	-	-	-	-
**Length of Stay (months)**					
	<1	0.55	(0.37, 0.81)	0.43 (0.27, 0.7)	0.35 (-5.34, 6.05)
	1-<6 (comparator)	1.0	-	1.0	0.54 (-8.22, 9.31)
	6-<12	0.90	(0.63, 1.28)	1.03 (0.67, 1.58)	0.55 (-8.31, 9.41)
	12-<24	0.99	(0.69, 1.41)	0.75 (0.48, 1.17)	0.48 (-7.24, 8.19)
	24+	1.76	(1.29, 2.39)	1.15 (0.74, 1.8)	0.57 (-8.7, 9.85)
**Ethnic Group**					
	White (comparator)	1.0	-	1.0	0.52 (-7.85, 8.88)
	Mixed	0.73	(0.27, 1.96)	1.14 (0.36, 3.6)	0.55 (-8.3, 9.4)
	Asian or Asian British	0.83	(0.43, 1.59)	1.18 (0.57, 2.44)	0.55 (-8.42, 9.53)
	Black or Black British	1.62	(0.63, 4.14)	1.46 (0.54, 3.93)	0.6 (-9.09, 10.29)
	Chinese and Other	0.96	(0.24, 3.87)	1.69 (0.34, 8.37)	0.63 (-9.56, 10.82)
	Unclassified	1.44	(0.64, 3.24)	1.08 (0.46, 2.58)	0.53 (-8.12, 9.19)

Indicator ***The proportion of people with coronary heart disease who have received a prescription for anti-platelet or anti-coagulant therapy in the preceding 12 months***

Group: Cardiovascular Disease

		Univariate		Multivariable			
	**Variable**	**Odds Ratio (OR) of achieving indicator**	**(95% CI) of OR**	**OR**	**(95% CI)**	**Probability of achieving indicator**	**(95% CI) of probability**
**Year**							
	2017/18 (comparator)	1.0	-	1.0	-	0.73	(0.58, 0.87)
	2018/19	0.79	(0.57, 1.08)	0.94	(0.65, 1.36)	0.72	(0.57, 0.86)
	2019/20	0.91	(0.67, 1.24)	1.13	(0.78, 1.64)	0.74	(0.6, 0.89)
**Prison**							
	Prison 1	1.16	(0.56, 2.39)	0.86	(0.35, 2.08)	0.70	(0.51, 0.89)
	Prison 2	1.31	(0.81, 2.1)	0.97	(0.54, 1.73)	0.72	(0.57, 0.88)
	Prison 3	1.54	(0.91, 2.59)	1.10	(0.56, 2.16)	0.75	(0.58, 0.91)
	Prison 4	2.96	(1.82, 4.79)	2.10	(1.07, 4.1)	0.84	(0.67, 1.003)
	Prison 5	2.48	(0.97, 6.33)	1.14	(0.37, 3.46)	0.75	(0.54, 0.97)
	Prison 6	1.32	(0.77, 2.26)	0.88	(0.43, 1.79)	0.71	(0.54, 0.87)
	Prison 7	0.55	(0.24, 1.3)	0.00	(0, 0)	0.02	(0.01, 0.04)
	Prison 8	0.55	(0.3, 1.01)	0.00	(0, 0)	0.03	(0.02, 0.04)
	Prison 9	2.43	(1.49, 3.96)	1.38	(0.68, 2.81)	0.78	(0.62, 0.94)
	Prison 10	1.03	(0.65, 1.66)	0.93	(0.51, 1.69)	0.72	(0.56, 0.88)
	Prison 11 (comparator)	1.0	-	1.0	-	0.73	(0.57, 0.89)
	Prison 12	0.63	(0.04, 10.24)	1.86	(0.1, 36.06)	0.82	(0.42, 1.22)
	Prison 13	2.27	(1.31, 3.96)	1.48	(0.67, 3.23)	0.79	(0.62, 0.96)
**Prison category**							
	A (comparator)	1.0	-				
	B	0.46	(0.32, 0.66)				
	C	0.88	(0.59, 1.3)				
	Closed	0.23	(0.14, 0.4)				
	D	0.64	(0.39, 1.05)				
	YOI	0.26	(0.02, 4.29)				
**Gender**							
	Female (comparator)	1.0	-	1.0	-	0.97	(0.79, 1.16)
	Male	2.78	(1.69, 4.58)	-	-	0.70	(0.57, 0.83)
**Age - years**							
	10 - <20	-	-	-	-	-	-
	20 - <30	0.17	(0.07, 0.4)	0.15	(0.05, 0.4)	0.38	(0.16, 0.61)
	30 - <40	0.11	(0.07, 0.18)	0.11	(0.06, 0.19)	0.32	(0.19, 0.44)
	40 - <50	0.36	(0.25, 0.51)	0.40	(0.26, 0.6)	0.62	(0.48, 0.75)
	50 - <60 (comparator)	1.0	-	1.0	-	0.79	(0.64, 0.95)
	60 - <70	1.27	(0.89, 1.8)	1.06	(0.71, 1.58)	0.80	(0.64, 0.96)
	70 - <80	1.66	(0.97, 2.84)	1.14	(0.64, 2.01)	0.81	(0.64, 0.98)
	80 - <90	2.82	(0.84, 9.46)	2.33	(0.67, 8.09)	0.90	(0.69, 1.1)
	90 - <100	-	-	-	-	-	
	100 - <110	-	-	-	-	-	-
**Length of Stay (months)**							
	<1	0.50	(0.34, 0.74)	0.41	(0.25, 0.67)	0.57	(0.43, 0.72)
	1-<6 (comparator)	1.0	-	1.0	-	0.74	(0.59, 0.89)
	6-<12	1.36	(0.92, 2)	1.47	(0.89, 2.44)	0.80	(0.64, 0.96)
	12-<24	1.69	(1.13, 2.54)	1.01	(0.61, 1.68)	0.75	(0.59, 0.9)
	24+	2.03	(1.43, 2.87)	0.92	(0.54, 1.55)	0.73	(0.58, 0.88)
**Ethnic Group**							
	White (comparator)	1.0	-	1.0	-	0.74	(0.6, 0.88)
	Mixed	0.36	(0.13, 0.96)	0.63	(0.19, 2.09)	0.66	(0.4, 0.91)
	Asian or Asian British	0.41	(0.21, 0.78)	0.61	(0.29, 1.27)	0.65	(0.47, 0.84)
	Black or Black British	0.60	(0.23, 1.53)	0.56	(0.2, 1.58)	0.64	(0.4, 0.87)
	Chinese and Other	0.57	(0.13, 2.41)	1.40	(0.26, 7.58)	0.79	(0.51, 1.07)
	Unclassified	0.73	(0.31, 1.72)	0.39	(0.15, 1.02)	0.56	(0.34, 0.78)

Indicator ***In those patients with a current diagnosis of heart failure due to left ventricular systolic dysfunction, the percentage of patients who are currently treated with an ACE-I or ARB (Inherited)***

Group: Cardiovascular Disease

	**Univariate**		**Multivariable**			
**Variable**	**Odds Ratio (OR) of achieving indicator**	**(95% CI) of OR**	**OR**	**(95% CI)**	**Probability of achieving indicator**	**(95% CI) of probability**
**Year**						
2017/18 (comparator)	1.0	-	1.0	-	0.82	(-123, 124.64)
2018/19	0.74	(0.34, 1.58)	0.57	(0.21, 1.52)	0.73	(-109.72, 111.18)
2019/20	0.56	(0.28, 1.15)	0.32	(0.12, 0.82)	0.62	(-93.07, 94.31)
**Prison**						
Prison 1	-	-	-	-	-	-
Prison 2	1.78	(0.5, 6.34)	1.53	(0.33, 7.13)	-	-
Prison 3	2.57	(0.91, 7.26)	1.58	(0.4, 6.24)	-	-
Prison 4	1.85	(0.7, 4.89)	1.39	(0.31, 6.21)	-	-
Prison 5	6.55	(0.74, 58.08)	3.46	(0.31, 38.63)	-	-
Prison 6	2.56	(0.45, 14.58)	1.23	(0.15, 10.07)	-	-
Prison 7	0.22	(0.02, 2.24)	-	-	-	-
Prison 8	0.35	(0.07, 1.7)	0.60	(0.05, 6.99)	-	-
Prison 9	2.09	(0.64, 6.84)	1.24	(0.22, 6.82)	-	-
Prison 10	3.75	(1.01, 13.88)	3.94	(0.78, 19.86)	-	-
Prison 11 (comparator)	1.0	-	1.0	-	-	-
Prison 12	-	-	-	-	-	-
Prison 13	-	-	-	-	-	-
**Prison category**						
A (comparator)	1.0	-	-	-	-	-
B	2.36	(1.02, 5.49)	-	-	-	-
C	3.21	(1.4, 7.36)	-	-	-	-
Closed	0.42	(0.11, 1.63)	-	-	-	-
D	5.74	(1.42, 23.28)	-	-	-	-
YOI	-	-	-	-	-	-
**Gender**						
Female (comparator)	1.0	-	-	-	-	-
Male	3.01	(0.65, 13.94)	-	-	-	-
**Age - years**						
10 - <20	-	-	-	-		
20 - <30	0.43	(0.11, 1.77)	0.79	(0.13, 4.78)	0.74	(-110.66, 112.13)
30 - <40	0.54	(0.17, 1.72)	0.63	(0.16, 2.53)	0.70	(-104.79, 106.19)
40 - <50	0.39	(0.16, 0.96)	0.47	(0.13, 1.68)	0.64	(-96.48, 97.77)
50 - <60 (comparator)	1.0	-	1.0	-	0.78	(-116.6, 118.15)
60 - <70	0.53	(0.23, 1.22)	0.82	(0.26, 2.54)	0.74	(-111.71, 113.19)
70 - <80	0.59	(0.24, 1.43)	0.51	(0.17, 1.53)	0.66	(-98.85, 100.17)
80 - <90	0.72	(0.16, 3.18)	0.68	(0.12, 3.78)	0.71	(-107.01, 108.43)
90 - <100	-	-	-	-		
100 - <110	-	-	-	-		
**Length of Stay (months)**						
<1	3.20	(0.94, 10.87)	3.80	(0.91, 15.79)	0.80	(-119.59, 121.19)
1-<6 (comparator)	1.0	-	1.0	-	0.53	(-80.28, 81.34)
6-<12	3.06	(1.22, 7.65)	2.29	(0.65, 8.08)	0.71	(-106.57, 107.99)
12-<24	3.87	(1.57, 9.55)	4.72	(1.33, 16.78)	0.83	(-124.31, 125.96)
24+	1.29	(0.63, 2.67)	2.64	(0.68, 10.21)	0.74	(-110.52, 111.99)

Indicator *In those patients with a current diagnosis of heart failure due to left ventricular systolic dysfunction who are currently treated with an ACE-I or ARB, the percentage of patients who are additionally currently treated with a beta-blocker licensed for heart failure*

Group: Cardiovascular Disease

	Univariate		Multivariable			
**Variable**	**Odds Ratio (OR) of achieving indicator**	**(95% CI) of OR**	**OR**	**(95% CI)**	**Probability of achieving indicator**	**(95% CI) of probability**
**Year**						
2017/18 (comparator)	1.0	-	1.0	-	0.79	(0.65, 0.94)
2018/19	1.17	(0.4, 3.42)	1.20	(0.35, 4.07)	0.82	(0.68, 0.95)
2019/20	0.91	(0.34, 2.45)	0.87	(0.27, 2.76)	0.77	(0.64, 0.9)
**Prison**						
Prison 1	-	-	-	-		
Prison 2	1.91	(0.3, 12.04)	3.21	(0.36, 28.6)	0.75	(0.44, 1.05)
Prison 3	0.61	(0.17, 2.17)	1.91	(0.32, 11.43)	0.65	(0.47, 0.83)
Prison 4	2.71	(0.62, 11.83)	10.02	(1.29, 77.82)	0.89	(0.76, 1.03)
Prison 5	3.39	(0.34, 34.08)	7.82	(0.58, 106.26)	0.87	(0.62, 1.13)
Prison 6	-	-	-	-		
Prison 7	-	-	-	-		
Prison 8	-	-	-	-		
Prison 9	2.70	(0.44, 16.4)	15.51	(1.36, 176.33)	0.93	(0.8, 1.06)
Prison 10	1.96	(0.39, 9.92)	4.41	(0.66, 29.35)	0.80	(0.56, 1.03)
Prison 11 (comparator)	1.0	-	1.0	-	0.51	(0.2, 0.82)
Prison 12	-	-	-	-		
Prison 13	-	-	-	-		
**Prison category**						
A (comparator)	1.0	-				
B	0.55	(0.11, 2.79)				
C	0.45	(0.09, 2.21)				
Closed	-	-				
D	1.94	(0.16, 23.93)				
YOI	-	-				
**Gender**						
Female (comparator)	-	-	-	-		
Male	-	-	-	-	0.79	(0.69, 0.89)
**Age - years**						
10 - <20	-	-	-	-		
20 - <30	0.53	(0.08, 3.35)	0.93	(0.11, 7.91)	0.73	(0.38, 1.08)
30 - <40	-	-	-	-		
40 - <50	1.57	(0.39, 6.38)	2.21	(0.42, 11.49)	0.85	(0.67, 1.03)
50 - <60 (comparator)	1.0	-	1.0	-	0.74	(0.59, 0.89)
60 - <70	1.50	(0.46, 4.86)	1.55	(0.37, 6.39)	0.81	(0.63, 0.98)
70 - <80	1.07	(0.35, 3.3)	1.67	(0.44, 6.35)	0.82	(0.66, 0.97)
80 - <90	1.39	(0.14, 13.53)	2.05	(0.18, 23.38)	0.84	(0.56, 1.12)
90 - <100	-	-	-	-		
100 - <110	-	-	-	-	-	-
**Length of Stay (months)**						
<1	0.28	(0.05, 1.48)	0.27	(0.03, 2.1)	0.77	(0.52, 1.02)
1-<6 (comparator)	1.0	-	1.0	-	0.92	(0.78, 1.05)
6-<12	0.72	(0.15, 3.57)	0.40	(0.06, 2.8)	0.82	(0.65, 1)
12-<24	0.60	(0.14, 2.65)	0.33	(0.05, 2.19)	0.80	(0.64, 0.96)
24+	0.32	(0.08, 1.25)	0.14	(0.02, 1.06)	0.65	(0.45, 0.86)
**Ethnic Group**						
White (comparator)	-	-				
Mixed	-	-				
Asian or Asian British	-	-				
Black or Black British	-	-				
Chinese and Other	-	-				
Unclassified	-	-				

Indicator ***The proportion of women eligible for screening and aged 25-49 years who have had cervical screening in the preceding five years and six months***

Group: Screening

	Univariate		Multivariable			
**Variable**	**Odds Ratio (OR) of achieving indicator**	**(95% CI) of OR**	**OR**	**(95% CI)**	**Probability of achieving indicator**	**(95% CI) of probability**
**Year**						
2017/18 (comparator)	1.0	-	1.0	-	0.56	(-2.31, 3.43)
2018/19	1.50	(1.28, 1.75)	1.61	(1.36, 1.91)	0.67	(-2.74, 4.08)
2019/20	1.41	(1.22, 1.63)	1.61	(1.37, 1.89)	0.67	(-2.74, 4.08)
**Prison**						
Prison 1	-	-	-	-	-	-
Prison 2	-	-	-	-	-	-
Prison 3	-	-	-	-	-	-
Prison 4	-	-	-	-	-	-
Prison 5	-	-	-	-	-	-
Prison 6	-	-	-	-	-	-
Prison 7 (comparator)	1.0	-	1.0	-	0.61	(-2.51, 3.73)
Prison 8	1.08	(0.95, 1.22)	1.20	(1.05, 1.38)	0.65	(-2.68, 3.98)
Prison 9	-	-	-	-	-	-
Prison 10	-	-	-	-	-	-
Prison 11	-	-	-	-	-	-
Prison 12	-	-	-	-	-	-
Prison 13	-	-	-	-	-	-
**Prison category**						
A (comparator)	-	-				
B	-	-				
C	-	-				
Closed	-	-				
D	-	-				
YOI	-	-				
**Gender**						
Female (comparator)	-	-	-	-	-	-
Male	-	-				
**Age - years**						
10 - <20	-	-	-	-		
20 - <30	0.61	(0.52, 0.73)	0.6	(0.47, 0.68)	0.55	(-2.24, 3.33)
30 - <40 (comparator)	1.0	-	1.0	-	0.67	(-2.76, 4.1)
40 - <50	0.92	(0.8, 1.06)	0.8	(0.72, 0.97)	0.63	(-2.6, 3.87)
50 - <60	0.76	(0.5, 1.17)	0.8	(0.47, 1.2)	0.61	(-2.5, 3.72)
60 - <70	-	-	-	-		
70 - <80	-	-	-	-		
80 - <90	-	-	-	-		
90 - <100	-	-	-	-		
100 - <110	-	-	-	-		
**Length of Stay (months)**						
<1	0.67	(0.58, 0.78)	0.7	(0.56, 0.78)	0.51	(-2.07, 3.08)
1-<6 (comparator)	1.0	-	1.0	-	0.60	(-2.47, 3.68)
6-<12	1.84	(1.52, 2.22)	1.9	(1.52, 2.29)	0.74	(-3.02, 4.49)
12-<24	2.21	(1.77, 2.77)	2.0	(1.61, 2.58)	0.75	(-3.09, 4.6)
24+	2.62	(2.02, 3.4)	2.6	(1.97, 3.43)	0.79	(-3.26, 4.85)
**Ethnic Group**						
White (comparator)	1.0	-	1.0	-	0.64	(-2.62, 3.9)
Mixed	1.21	(0.78, 1.88)	1.1	(0.72, 1.8)	0.67	(-2.74, 4.07)
Asian or Asian British	0.70	(0.43, 1.16)	0.6	(0.36, 1.02)	0.53	(-2.16, 3.22)
Black or Black British	1.22	(0.76, 1.96)	1.4	(0.84, 2.28)	0.71	(-2.9, 4.31)
Chinese and Other	0.53	(0.32, 0.9)	0.6	(0.33, 0.95)	0.51	(-2.09, 3.1)
Unclassified	0.52	(0.23, 1.14)	0.7	(0.29, 1.46)	0.54	(-2.23, 3.32)

Indicator ***The proportion of women eligible for screening and aged 50-64 years who have had cervical screening in the preceding five years and six months***

Group: Screening

		Univariate		Multivariable			
	**Variable**	**Odds Ratio (OR) of achieving indicator**	**(95% CI) of OR**	**OR**	**(95% CI)**	**Probability of achieving indicator**	**(95% CI) of probability**
**Year**							
	2017/18 (comparator)	1.0	-	1.0	-	0.55	(-20.54, 21.64)
	2018/19	1.10	(0.76, 1.59)	1.18	(0.8, 1.74)	0.59	(-21.99, 23.17)
	2019/20	1.33	(0.92, 1.92)	1.50	(1.01, 2.24)	0.65	(-24.11, 25.4)
**Prison**							
	Prison 1	-	-	-	-		
	Prison 2	-	-	-	-		
	Prison 3	-	-	-	-		
	Prison 4	-	-	-	-		
	Prison 5	-	-	-	-		
	Prison 6	-	-	-	-		
	Prison 7	1.0	-	1.0	-	0.60	(-22.53, 23.74)
	Prison 8	1.04	(0.76, 1.42)	0.96	(0.68, 1.35)	0.59	(-22.16, 23.35)
	Prison 9	-	-	-	-		
	Prison 10	-	-	-	-		
	Prison 11 (comparator)	-	-	-	-		
	Prison 12	-	-	-	-		
	Prison 13						
**Prison category**							
	A (comparator)	-	-				
	B	-	-				
	C	-	-				
	Closed	-	-				
	D	-	-				
	YOI	-	-				
**Gender**							
	Female (comparator)	-	-				
	Male	-	-				
**Age - years**							
	10 - <20	-	-	-	-		
	20 - <30	-	-	-	-		
	30 - <40	-	-	-	-		
	40 - <50	-	-	-	-		
	50 - <60 (comparator)	1.0	-	1.0	-	0.61	(-22.76, 23.98)
	60 - <70	0.75	(0.5, 1.14)	0.7	(0.45, 1.1)	0.53	(-19.64, 20.69)
	70 - <80	-	-	-	-		
	80 - <90	-	-	-	-		
	90 - <100	-	-	-	-		
	100 - <110	-	-	-	-		
**Length of Stay (months)**							
	<1	0.70	(0.46, 1.07)	0.7	(0.42, 1.04)	0.50	(-18.68, 19.68)
	1-<6 (comparator)	1.0	-	1.0	-	0.60	(-22.47, 23.68)
	6-<12	1.08	(0.71, 1.65)	1.0	(0.66, 1.64)	0.61	(-22.8, 24.03)
	12-<24	1.05	(0.57, 1.95)	0.9	(0.45, 1.64)	0.56	(-21.06, 22.19)
	24+	1.46	(0.94, 2.25)	1.4	(0.85, 2.14)	0.67	(-25.01, 26.35)
**Ethnic Group**							
	White (comparator)	1.0	-	1.0	-	0.60	(-22.26, 23.45)
	Mixed	0.53	(0.16, 1.76)	0.6	(0.17, 2.2)	0.48	(-17.87, 18.83)
	Asian or Asian British	2.76	(0.58, 13.17)	2.6	(0.53, 12.4)	0.79	(-29.42, 31)
	Black or Black British	0.78	(0.16, 3.93)	0.9	(0.17, 4.53)	0.57	(-21.15, 22.28)
	Chinese and Other	-	-	-	-		
	Unclassified	-	-	-	-		

Indicator ***The proportion of women eligible for screening and aged 50-70 years who have had breast screening in the preceding three years***

Group: Screening

Variable	Univariate: Odds Ratio (OR) of achieving indicator	Univariate: (95% CI) of OR	Multivariable: OR	Multivariable: (95% CI)	Multivariable: Probability of achieving indicator	Multivariable: (95% CI) of probability
**Year**						
2017/18 (comparator)	1.0	-	1.0	-	0.27	(-1.42, 1.96)
2018/19	1.14	(0.71, 1.85)	1.16	(0.68, 1.98)	0.30	(-1.56, 2.15)
2019/20	1.22	(0.77, 1.95)	1.69	(0.88, 3.21)	0.37	(-1.94, 2.68)
**Prison**						
Prison 1	-	-	-	-	-	-
Prison 2	-	-	-	-	-	-
Prison 3	-	-	-	-	-	-
Prison 4	-	-	-	-	-	-
Prison 5	-	-	-	-	-	-
Prison 6	-	-	-	-	-	-
Prison 7	1.0	-	1.0	-	0.51	(-2.71, 3.74)
Prison 8	0.40	(0.27, 0.59)	0.27	(0.15, 0.47)	0.24	(-1.24, 1.71)
Prison 9	-	-	-	-	-	-
Prison 10	-	-	-	-		
Prison 11 (comparator)	-	-	-	-	-	-
Prison 12	-	-	-	-	-	-
Prison 13	-	-	-	-	-	-
**Prison category**						
A (comparator)	-	-	-	-	-	-
B	-	-	-	-	-	-
C	-	-	-	-	-	-
Closed	-	-	-	-	-	-
D	-	-	-	-	-	-
YOI	-	-	-	-	-	-
**Gender**						
Female (comparator)	-	-	-	-	0.30	(-1.59, 2.19)
Male	-	-	-	-	-	-
**Age - years**						
10 - <20	-	-	-	-	-	-
20 - <30	-	-	-	-	-	-
30 - <40	-	-	-	-	-	-
40 - <50	-	-	-	-	-	-
50 - <60 (comparator)	1.0	-	1.0	-	0.34	(-1.79, 2.47)
60 - <70	0.58	(0.36, 0.92)	0.43	(0.23, 0.78)	0.19	(-1.02, 1.41)
70 - <80	0.39	(0.04, 3.34)	0.57	(0.06, 5.38)	0.24	(-1.29, 1.76)
80 - <90	-	-	-	-	-	-
90 - <100	-	-	-	-	-	-
100 - <110	-	-	-	-	-	-
**Length of Stay (months)**						
<1	0.63	(0.33, 1.19)	0.69	(0.32, 1.47)	0.21	(-1.11, 1.53)
1-<6 (comparator)	1.0	-	1.0	-	0.27	(-1.43, 1.98)
6-<12	1.46	(0.86, 2.5)	1.23	(0.63, 2.38)	0.31	(-1.64, 2.26)
12-<24	1.38	(0.64, 2.98)	1.48	(0.6, 3.66)	0.35	(-1.84, 2.54)
24+	2.05	(1.23, 3.42)	1.84	(0.98, 3.47)	0.40	(-2.08, 2.87)
**Ethnic Group**						
White (comparator)	1.0	-	1.0	-	0.30	(-1.56, 2.15)
Mixed	0.43	(0.05, 3.75)	-	-	-	-
Asian or Asian British	0.56	(0.06, 5.11)	0.62	(0.06, 6.13)	0.22	(-1.18, 1.61)
Black or Black British	3.57	(0.58, 21.79)	8.72	(1.33, 57.19)	0.74	(-3.92, 5.41)
Chinese and Other	2.22	(0.14, 36.08)	4.67	(0.25, 88.15)	0.62	(-3.32, 4.57)
Unclassified	-	-	-	-	-	-

Indicator *Offer people equivalent health checks to those offered in the community e.g. relevant NHS screening programmes, such as those for abdominal aortic aneurysm (AAA screening is offered to men during the screening year (1 April to 31 March) that they turn 65)*

Group: Screening

	Univariate		Multivariable			
**Variable**	**Odds Ratio (OR) of achieving indicator**	**(95% CI) of OR**	**OR**	**(95% CI)**	**Probability of achieving indicator**	**(95% CI) of probability**
**Year**						
2017/18 (comparator)	1.0	-	1.0	-	0.54	(0.5, 0.59)
2018/19	1.14	(0.89, 1.47)	1.14	(0.81, 1.59)	0.57	(0.52, 0.61)
2019/20	1.05	(0.82, 1.34)	1.32	(0.94, 1.85)	0.59	(0.55, 0.64)
**Prison**						
Prison 1	19.21	(5.08, 72.66)	9.12	(1.6, 52.04)	0.83	(0.65, 1.01)
Prison 2	5.28	(3.29, 8.5)	2.83	(1.53, 5.25)	0.67	(0.61, 0.73)
Prison 3	-	-	-	-		
Prison 4	16.66	(10.37, 26.75)	2.80	(1.42, 5.53)	0.67	(0.61, 0.73)
Prison 5	2.98	(1.47, 6.04)	0.63	(0.25, 1.54)	0.38	(0.24, 0.53)
Prison 6	7.62	(4.23, 13.73)	4.52	(1.9, 10.75)	0.74	(0.64, 0.84)
Prison 7	-	-	-	-		
Prison 8	-	-	-	-		
Prison 9	7.84	(5, 12.3)	1.00	(0.48, 2.05)	0.48	(0.41, 0.54)
Prison 10	2.26	(1.38, 3.72)	1.22	(0.64, 2.29)	0.52	(0.43, 0.6)
Prison 11 (comparator)	1.0	-	1.0	-	0.48	(0.36, 0.59)
Prison 12	-	-	-	-		
Prison 13	8.88	(5.38, 14.66)	1.26	(0.58, 2.73)	0.52	(0.44, 0.61)
**Prison category**						
A (comparator)	1.0	-				
B	0.29	(0.22, 0.38)				
C	0.51	(0.39, 0.68)				
Closed	-	-				
D	0.66	(0.44, 0.99)				
YOI	-	-				
**Gender**						
Female (comparator)	-	-	-	-		
Male	-	-	-	-	0.57	(0.54, 0.59)
**Age - years**						
10 - <20	-	-	-	-		
20 - <30	-	-	-	-		
30 - <40	-	-	-	-		
40 - <50	-	-	-	-		
50 - <60 (comparator)	1.0	-	1.0	-		
60 - <70	0.32	(0.06, 1.65)	0.34	(0.03, 3.48)	0.54	(0.5, 0.58)
70 - <80	0.36	(0.07, 1.86)	0.44	(0.04, 4.49)	0.59	(0.55, 0.63)
80 - <90	0.27	(0.05, 1.44)	0.55	(0.05, 5.93)	0.63	(0.54, 0.72)
90 - <100	-	-	-	-	0.72	(0.39, 1.06)
100 - <110	-	-	-	-		
**Length of Stay (months)**						
<1	0.33	(0.18, 0.58)	0.27	(0.14, 0.54)	0.11	(0.06, 0.17)
1-<6 (comparator)	1.0	-	1.0	-	0.30	(0.22, 0.38)
6-<12	3.53	(2.37, 5.27)	3.27	(1.96, 5.47)	0.56	(0.47, 0.64)
12-<24	3.82	(2.62, 5.57)	5.04	(2.98, 8.54)	0.65	(0.58, 0.73)
24+	6.11	(4.4, 8.48)	8.04	(4.53, 14.26)	0.74	(0.69, 0.79)
**Ethnic Group**						
White (comparator)	1.0	-	-	-	0.57	(0.55, 0.6)
Mixed	0.47	(0.16, 1.39)	0.26	(0.08, 0.81)	0.32	(0.11, 0.52)
Asian or Asian British	1.74	(0.77, 3.95)	3.59	(1, 12.89)	0.77	(0.61, 0.93)
Black or Black British	0.54	(0.16, 1.86)	0.34	(0.08, 1.43)	0.37	(0.1, 0.64)
Chinese and Other	1.02	(0.06, 16.35)	1.52	(0.09, 25.27)	0.65	(0.18, 1.11)
Unclassified	1.30	(0.65, 2.63)	0.68	(0.31, 1.52)	0.50	(0.35, 0.65)

Indicator	*The proportion of people aged 45 to 74 years with a recorded cardiovascular disease risk assessment in the preceding 5 years, and who do NOT have a diagnosis of: coronary heart disease, chronic kidney disease, diabetes, hypertension, atrial fibrillation, transient ischaemic attack, familial hypercholesterolemia, heart failure, peripheral arterial disease, stroke or previously documented 20% or higher 10-year cardiovascular disease risk BEFORE the last 5 years*						
	**Group: Screening**						
		**Univariate**		**Multivariable**			
	**Variable**	**Odds Ratio (OR) of achieving indicator**	**(95% CI) of OR**	**OR**	**(95% CI)**	**Probability of achieving indicator**	**(95% CI) of probability**
**Year**							
	2019/20						
**Prison**							
	Prison 1	11.45	(4.53, 28.96)	6.25	(2.34, 16.69)	0.57	(0.41, 0.73)
	Prison 2	3.75	(1.62, 8.66)	2.25	(0.94, 5.37)	0.33	(0.21, 0.44)
	Prison 3	4.53	(2.01, 10.21)	2.67	(1.12, 6.36)	0.37	(0.25, 0.48)
	Prison 4	2.05	(0.9, 4.64)	1.41	(0.59, 3.39)	0.24	(0.14, 0.33)
	Prison 5	3.75	(1.35, 10.43)	2.40	(0.78, 7.37)	0.34	(0.15, 0.53)
	Prison 6	21.00	(8.75, 50.39)	10.04	(3.94, 25.56)	0.67	(0.55, 0.8)
	Prison 7	3.39	(1.23, 9.36)	0.69	(0.03, 13.91)	0.13	(-0.15, 0.42)
	Prison 8	2.88	(1.13, 7.35)	0.89	(0.05, 17.28)	0.16	(-0.17, 0.5)
	Prison 9	4.54	(2.05, 10.06)	2.06	(0.84, 5.06)	0.31	(0.21, 0.41)
	Prison 10	3.94	(1.66, 9.34)	2.65	(1.07, 6.52)	0.36	(0.23, 0.5)
	Prison 11 (comparator)	1.0	-	1.0	-	0.18	(0.06, 0.31)
	Prison 12	-	-	-	-		
	Prison 13	9.85	(4.41, 22.04)	4.62	(1.87, 11.4)	0.49	(0.38, 0.61)
**Prison category**							
	A (comparator)	1.0	-	-	-	-	-
	B	0.44	(0.3, 0.63)	-	-	-	-
	C	0.56	(0.41, 0.78)	-	-	-	-
	Closed	0.48	(0.29, 0.8)	-	-	-	-
	D	1.82	(1.17, 2.84)	-	-	-	-
	YOI	-	-	-	-	-	-
**Gender**							
	Female (comparator)	1.0	-	1.0	-	0.50	(-0.06, 1.07)
	Male	1.46	(0.92, 2.32)	0.45	(0.03, 7.39)	0.33	(0.29, 0.38)
**Age - years**							
	10 - <20	-	-	-	-	-	-
	20 - <30	-	-	-	-	-	-
	30 - <40	-	-	-	-	-	-
	40 - <50	0.84	(0.65, 1.1)	0.85	(0.63, 1.15)	0.33	(0.29, 0.38)
	50 - <60 (comparator)	1.0	-	1.0	-	0.37	(0.32, 0.41)
	60 - <70	0.71	(0.45, 1.12)	0.80	(0.48, 1.34)	0.32	(0.23, 0.41)
	70 - <80	0.32	(0.07, 1.44)	0.50	(0.1, 2.4)	0.24	(-0.02, 0.49)
	80 - <90	-	-	-	-	-	-
	90 - <100	-	-	-	-	-	-
	100 - <110	-	-	-	-	-	-
**Length of Stay (months)**							
	<1	0.72	(0.42, 1.24)	0.88	(0.49, 1.59)	0.25	(0.16, 0.34)
	1-<6 (comparator)	1.0	-	1.0	-	0.27	(0.21, 0.34)
	6-<12	2.33	(1.56, 3.48)	1.88	(1.19, 2.97)	0.40	(0.33, 0.48)
	12-<24	2.23	(1.5, 3.32)	1.78	(1.12, 2.82)	0.39	(0.32, 0.46)
	24+	2.22	(1.54, 3.2)	1.60	(0.97, 2.65)	0.37	(0.3, 0.43)
**Ethnic Group**							
	White (comparator)	1.0	-	1.0	-	0.35	(0.31, 0.38)
	Mixed	0.92	(0.44, 1.92)	0.93	(0.43, 2)	0.33	(0.18, 0.48)
	Asian or Asian British	1.18	(0.6, 2.32)	1.07	(0.52, 2.23)	0.36	(0.21, 0.51)
	Black or Black British	1.93	(0.83, 4.51)	1.82	(0.74, 4.53)	0.48	(0.27, 0.68)
	Chinese and Other	0.43	(0.09, 2)	0.47	(0.1, 2.32)	0.21	(-0.03, 0.45)
	Unclassified	1.29	(0.61, 2.71)	0.82	(0.37, 1.85)	0.31	(0.15, 0.46)

Indicator ***The proportion of people who have received three or more prescriptions for an antipsychotic in the preceding 12 months, and who have a record of blood testing (fasting glucose, HbA1c, lipid profile, urea and electrolytes, full blood count, liver function tests), body mass index and blood pressure***

Group: Mental health

Univariate: Odds ratios (95% CI)

**Variable**	**Fasting glucose**		**HbA1c**		**Lipid profile**		**Urea and electrolytes**		**Full blood count**		**Liver function**		**BMI**		**Blood Pressure**	
**Year**																
2017/18 (comparator)	1.0	-	1.0	-	1.0	-	1.0	-	1.0	-	1.0	-	1.0	-	1.0	-
2018/19	0.18	(0.04, 0.87)	0.30	(0.2, 0.46)	0.43	(0.28, 0.67)	0.25	(0.17, 0.36)	0.26	(0.18, 0.37)	0.22	(0.15, 0.32)	0.18	(0.12, 0.26)	0.10	(0.06, 0.15)
2019/20	-	-	0.15	(0.09, 0.23)	0.17	(0.1, 0.28)	0.10	(0.06, 0.14)	0.10	(0.06, 0.14)	0.06	(0.03, 0.09)	0.02	(0.01, 0.03)	0.03	(0.02, 0.05)
**Prison**																
Prison 1	2.67	(0.21, 33.8)	3.82	(1.02, 14.26)	1.81	(0.42, 7.69)	2.83	(1.06, 7.56)	4.43	(1.74, 11.25)	4.40	(1.55, 12.51)	2.87	(1.16, 7.1)	2.20	(0.93, 5.2)
Prison 2	-	-	5.46	(1.95, 15.28)	0.56	(0.14, 2.31)	2.51	(1.18, 5.32)	2.50	(1.18, 5.31)	1.95	(0.82, 4.63)	1.82	(0.94, 3.54)	2.07	(1.12, 3.83)
Prison 3	0.39	(0.02, 6.73)	5.00	(1.78, 14.08)	4.96	(1.9, 12.93)	5.06	(2.47, 10.37)	5.55	(2.71, 11.36)	1.78	(0.74, 4.26)	9.05	(4.6, 17.82)	6.11	(3.34, 11.16)
Prison 4	-	-	1.62	(0.45, 5.85)	3.00	(1.04, 8.67)	2.02	(0.89, 4.62)	1.84	(0.8, 4.25)	2.36	(0.95, 5.87)	2.68	(1.33, 5.41)	1.23	(0.62, 2.45)
Prison 5	-	-	-	-	4.49	(0.39, 51.48)	1.49	(0.14, 16.41)	1.48	(0.13, 16.33)	1.63	(0.14, 19.02)	1.64	(0.16, 17.05)	0.67	(0.07, 6.61)
Prison 6	3.07	(0.17, 57.07)	11.87	(2.83, 49.72)	3.03	(0.54, 17.13)	5.87	(1.67, 20.58)	5.90	(1.67, 20.77)	8.35	(2.1, 33.18)	6.84	(1.67, 27.98)	4.23	(1.2, 14.88)
Prison 7	-	-	4.02	(1.36, 11.88)	3.63	(1.33, 9.95)	3.56	(1.68, 7.53)	3.79	(1.8, 8)	4.48	(1.92, 10.46)	1.53	(0.75, 3.12)	2.19	(1.2, 4.02)
Prison 8	-	-	6.90	(2.42, 19.68)	2.51	(0.85, 7.41)	4.16	(1.94, 8.92)	3.64	(1.68, 7.89)	3.85	(1.62, 9.15)	3.01	(1.48, 6.11)	2.30	(1.19, 4.47)
Prison 9	2.91	(0.31, 27.69)	30.88	(11.31, 84.33)	25.73	(10.09, 65.57)	13.49	(6.39, 28.47)	19.14	(9.02, 40.62)	23.68	(10.2, 54.97)	8.51	(4.09, 17.73)	10.57	(5.37, 20.81)
Prison 10	-	-	1.52	(0.34, 6.83)	0.45	(0.05, 3.9)	1.97	(0.74, 5.28)	1.97	(0.74, 5.3)	1.69	(0.55, 5.16)	1.53	(0.57, 4.12)	2.19	(0.9, 5.31)
Prison 11 (comparator)	1.0	-	1.0	-	1.0	-	1.0	-	1.0	-	1.0	-	1.0	-	1.0	-
Prison 12	-	-	-	-	-	-	-	-	-	-	-	-	-	-	-	-
Prison 13	-	-	18.53	(6.29, 54.57)	11.62	(4.15, 32.51)	8.17	(3.52, 19.01)	8.21	(3.52, 19.12)	10.79	(4.22, 27.62)	3.90	(1.67, 9.11)	3.38	(1.54, 7.41)
**Prison category**																
A (comparator)	1.0	-	1.0	-	1.0	-	1.0	-	1.0	-	1.0	-	1.0	-	1.0	-
B	0.09	(0.01, 0.83)	0.10	(0.06, 0.17)	0.04	(0.02, 0.08)	0.15	(0.09, 0.25)	0.12	(0.07, 0.2)	0.08	(0.05, 0.15)	0.21	(0.13, 0.37)	0.22	(0.13, 0.36)
C	0.33	(0.08, 1.44)	0.14	(0.08, 0.23)	0.19	(0.11, 0.32)	0.31	(0.19, 0.5)	0.27	(0.17, 0.44)	0.13	(0.08, 0.23)	0.75	(0.44, 1.27)	0.44	(0.27, 0.73)
Closed	-	-	0.21	(0.12, 0.35)	0.16	(0.09, 0.28)	0.34	(0.21, 0.56)	0.27	(0.16, 0.44)	0.24	(0.14, 0.41)	0.34	(0.19, 0.6)	0.33	(0.2, 0.56)
D	1.12	(0.12, 10.79)	0.28	(0.09, 0.87)	0.18	(0.05, 0.66)	0.38	(0.13, 1.12)	0.31	(0.1, 0.91)	0.32	(0.1, 1.02)	0.77	(0.22, 2.7)	0.43	(0.13, 1.37)
YOI	-	-	-	-	-	-	-	-	-	-	-	-	-	-	-	-
**Gender**																
Female (comparator)	1.0	-	1.0	-	1.0	-	1.0	-	1.0	-	1.0	-	1.0	-	1.0	-
Male	-	-	0.86	(0.54, 1.37)	1.09	(0.65, 1.83)	0.81	(0.53, 1.23)	0.91	(0.6, 1.39)	0.71	(0.46, 1.1)	1.20	(0.78, 1.83)	1.11	(0.73, 1.68)
**Age - years**																
10 - <20	-	-	-	-	-	-	-	-	-	-	-	-	0.50	(0.07, 3.75)	0.28	(0.04, 2.12)
20 - <30	-	-	0.68	(0.37, 1.22)	0.60	(0.31, 1.16)	0.61	(0.37, 0.99)	0.58	(0.35, 0.95)	0.57	(0.33, 0.98)	1.02	(0.64, 1.63)	1.01	(0.65, 1.56)
30 - <40 (comparator)	-	-	-	-	-	-	-	-	-	-	-	-	-	-	-	-
40 - <50	0.77	(0.14, 4.27)	1.32	(0.84, 2.09)	1.24	(0.76, 2.01)	0.90	(0.6, 1.35)	1.02	(0.68, 1.52)	0.76	(0.48, 1.2)	1.08	(0.71, 1.64)	1.40	(0.95, 2.06)
50 - <60	1.46	(0.26, 8.27)	2.27	(1.31, 3.95)	2.50	(1.42, 4.4)	1.61	(0.96, 2.7)	1.98	(1.19, 3.3)	1.53	(0.88, 2.67)	1.83	(1.03, 3.23)	2.13	(1.24, 3.67)
60 - <70	3.22	(0.33, 31.44)	3.73	(1.47, 9.47)	2.33	(0.85, 6.42)	7.39	(2.84, 19.21)	7.36	(2.83, 19.13)	6.72	(2.54, 17.77)	8.98	(2.86, 28.18)	12.78	(3.91, 41.8)
70 - <80	5.38	(0.52, 55.05)	2.00	(0.58, 6.87)	1.63	(0.43, 6.19)	0.59	(0.15, 2.32)	0.91	(0.26, 3.16)	1.65	(0.48, 5.62)	0.48	(0.13, 1.83)	2.44	(0.74, 8.1)
80 - <90	-	-	-	-	-	-	2.37	(0.26, 21.46)	2.37	(0.26, 21.42)	-	-	8.57	(0.45, 164.44)	-	-
90 - <100	-	-	-	-	-	-	-	-	-	-	-	-	-	-	-	-
100 - <110	-	-	-	-	-	-	-	-	-	-	-	-	-	-	-	-
**Length of Stay (months)**																
<1	-	-	0.73	(0.32, 1.66)	0.57	(0.19, 1.74)	0.70	(0.35, 1.37)	0.70	(0.35, 1.37)	0.93	(0.44, 1.98)	1.45	(0.82, 2.55)	1.23	(0.73, 2.06)
1-<6 (comparator)	-	-	-	-	-	-	-	-	-	-	-	-	-	-	-	-
6-<12	2.86	(0.25, 32.21)	1.45	(0.82, 2.56)	1.66	(0.85, 3.27)	1.55	(0.96, 2.5)	1.76	(1.1, 2.83)	1.92	(1.11, 3.32)	1.69	(1.04, 2.74)	1.26	(0.81, 1.97)
12-<24	2.97	(0.26, 33.45)	1.86	(1.04, 3.33)	3.21	(1.69, 6.09)	2.11	(1.29, 3.46)	2.11	(1.29, 3.45)	2.13	(1.21, 3.74)	2.41	(1.46, 3.98)	2.20	(1.37, 3.52)
24+	6.26	(0.72, 54.75)	5.34	(3.2, 8.93)	8.31	(4.64, 14.88)	4.94	(3.11, 7.87)	5.70	(3.58, 9.07)	6.95	(4.13, 11.69)	3.22	(1.97, 5.26)	4.62	(2.88, 7.4)
**Ethnic Group**																
White (comparator)	-	-	-	-	-	-	-	-	-	-	-	-	-	-	-	-
Mixed	-	-	0.45	(0.06, 3.63)	-	-	0.54	(0.11, 2.65)	0.50	(0.1, 2.48)	0.73	(0.14, 3.76)	0.49	(0.11, 2.24)	0.29	(0.06, 1.36)
Asian or Asian British	9.62	(0.99, 93.54)	8.07	(3.05, 21.34)	4.65	(1.72, 12.58)	4.66	(1.74, 12.48)	4.34	(1.62, 11.6)	4.28	(1.51, 12.17)	3.42	(1.12, 10.46)	2.54	(0.91, 7.11)
Black or Black British	16.40	(2.7, 99.6)	0.90	(0.24, 3.33)	2.46	(0.83, 7.31)	1.30	(0.44, 3.87)	1.23	(0.41, 3.64)	1.80	(0.58, 5.64)	0.66	(0.19, 2.24)	0.73	(0.22, 2.39)
Chinese and Other	-	-	2.69	(0.81, 8.95)	1.60	(0.42, 6.1)	2.81	(0.84, 9.36)	2.63	(0.79, 8.73)	1.72	(0.49, 6.04)	3.37	(0.81, 14)	3.61	(0.84, 15.52)
Unclassified	-	-	2.45	(0.65, 9.26)	1.76	(0.38, 8.21)	1.51	(0.39, 5.79)	1.41	(0.37, 5.41)	2.60	(0.64, 10.54)	0.73	(0.13, 4.21)	0.66	(0.16, 2.73)

Indicator: *The proportion of people who have received three or more prescriptions for an antipsychotic in the preceding 12 months, and who have a record of blood testing (fasting glucose, HbA1c, lipid profile, urea and electrolytes, full blood count, liver function tests), body mass index and blood pressure*

Domain: Mental health

Multivariable

Variable	Fasting glucose		HbA1c		Lipid profile		Urea and electrolytes		Full blood count		Liver function		BMI		Blood Pressure	
	OR (95% CI)	Probability of achieving indicator (95% CI)	OR (95% CI)	Probability of achieving indicator (95% CI)	OR (95% CI)	Probability of achieving indicator (95% CI)	OR (95% CI)	Probability of achieving indicator (95% CI)	OR (95% CI)	Probability of achieving indicator (95% CI)	OR (95% CI)	Probability of achieving indicator (95% CI)	OR (95% CI)	Probability of achieving indicator (95% CI)	OR (95% CI)	Probability of achieving indicator (95% CI)
**Year**																
2017/18 (comparator)	1.0	0.1 (-77.25, 77.44)	1.0	0.29 (-11.38, 11.96)	1.0	0.21 (-14.26, 14.67)	1.0	0.47 (-11.46, 12.4)	1.0	0.48 (-10.43, 11.38)	1.0	0.44 (-10.65, 11.53)	1.0	0.72 (-11.28, 12.71)	1.0	0.83 (0.78, 0.89)
2018/19	0.25 (0.03, 2.39)	0.04 (-28.63, 28.7)	0.31 (0.18, 0.51)	0.14 (-5.34, 5.62)	0.49 (0.28, 0.85)	0.13 (-9.04, 9.3)	0.24 (0.16, 0.38)	0.21 (-5.22, 5.64)	0.25 (0.16, 0.39)	0.23 (-4.99, 5.44)	0.18 (0.11, 0.29)	0.17 (-4, 4.33)	0.17 (0.1, 0.26)	0.37 (-5.75, 6.48)	0.09 (0.05, 0.15)	0.38 (0.33, 0.43)
2019/20	-		0.22 (0.13, 0.39)	0.11 (-4.24, 4.45)	0.31 (0.17, 0.57)	0.09 (-6.54, 6.73)	0.13 (0.08, 0.21)	0.14 (-3.34, 3.61)	0.12 (0.08, 0.2)	0.14 (-3.09, 3.37)	0.06 (0.03, 0.11)	0.08 (-1.85, 2)	0.02 (0.01, 0.03)	0.08 (-1.25, 1.41)	0.03 (0.02, 0.06)	0.21 (0.16, 0.25)
**Prison**																
Prison 1	3.44 (0.03, 439.92)	0.11 (-84.41, 84.62)	2.03 (0.4, 10.43)	-	0.55 (0.05, 5.56)	-	1.61 (0.47, 5.49)	-	2.48 (0.78, 7.88)	-	3.19 (0.86, 11.84)	-	4.12 (1.3, 13.06)	-	2.24 (0.77, 6.48)	-
Prison 2	-		5.23 (1.66, 16.41)	-	0.6 (0.13, 2.9)	-	2.32 (1, 5.39)	-	2.3 (0.99, 5.33)	-	1.99 (0.73, 5.44)	-	2.49 (1.15, 5.37)	-	2.14 (1.1, 4.16)	-
Prison 3	0.08 (0, 28.17)	0.01 (-8.66, 8.68)	3.59 (1.07, 12.06)	-	4.33 (1.28, 14.58)	-	4.01 (1.7, 9.5)	-	4.51 (1.92, 10.62)	-	1.43 (0.49, 4.2)	-	13.26 (5.66, 31.09)	-	5.22 (2.51, 10.88)	-
Prison 4	-		1.18 (0.28, 4.92)	-	2.52 (0.69, 9.21)	-	1.44 (0.55, 3.78)	-	1.35 (0.51, 3.55)	-	2.02 (0.68, 6.07)	-	4.87 (2.04, 11.6)	-	1.24 (0.56, 2.76)	-
Prison 5	-		-	-	4.31 (0.3, 62.02)	-	1.41 (0.1, 19.53)	-	1.48 (0.11, 20.48)	-	2.37 (0.15, 37.67)	-	1.65 (0.13, 21.22)	-	0.47 (0.04, 5.49)	-
Prison 6	-	-	8.81 (1.66, 46.66)	-	1.38 (0.13, 15.09)	-	3.5 (0.78, 15.79)	-	3.34 (0.74, 15.02)	-	6.52 (1.28, 33.14)	-	7.64 (1.52, 38.32)	-	3.47 (0.83, 14.49)	-
Prison 7	-	-	5.04 (1.41, 17.98)	-	5.13 (1.44, 18.23)	-	3.32 (1.3, 8.49)	-	3.64 (1.44, 9.22)	-	5.88 (2.07, 16.76)	-	2.03 (0.86, 4.8)	-	1.7 (0.77, 3.77)	-
Prison 8	-	-	5.73 (1.75, 18.72)	-	2.52 (0.69, 9.13)	-	3.51 (1.47, 8.39)	-	3.09 (1.28, 7.44)	-	4.06 (1.46, 11.25)	-	4.33 (1.88, 9.95)	-	2.22 (1.06, 4.65)	-
Prison 9	10.18 (0.02, 5175.59)	0.19 (-153.34, 153.73)	12.11 (3.44, 42.58)	-	12.22 (3.38, 44.09)	-	4.48 (1.66, 12.12)	-	6.93 (2.58, 18.62)	-	9.63 (3.14, 29.56)	-	13.17 (4.72, 36.74)	-	5.23 (2.06, 13.23)	-
Prison 10	-	0.05 (-40.89, 40.99)	1.65 (0.33, 8.27)	-	0.61 (0.06, 5.95)	-	1.71 (0.56, 5.26)	-	1.77 (0.58, 5.42)	-	1.7 (0.46, 6.24)	-	2.17 (0.67, 7.02)	-	2.64 (0.94, 7.39)	-
Prison 11 (comparator)	1.0	-	1.0	-	1.0	-	1.0	-	1.0	-	1.0	-	1.0	-	1.0	-
Prison 12	-	-	-	-	-	-	-	-	-	-	-	-	-	-	-	-
Prison 13	-	-	7 (1.82, 26.96)	-	5.09 (1.29, 20.05)	-	2.99 (0.99, 8.97)	-	3.26 (1.09, 9.81)	-	4.18 (1.22, 14.33)	-	6.85 (2.13, 22.08)	-	2.82 (0.98, 8.15)	-
**Prison category**																
A (comparator)	-		-		-		-		-		-		-		-	
B	-		-		-		-		-		-		-		-	
C	-		-		-		-		-		-		-		-	
Closed	-		-		-		-		-		-		-		-	
D	-		-		-		-		-		-		-		-	
YOI	-		-		-		-		-		-		-		-	
**Gender**																
Female (comparator)	1.0	-	1.0	-	1.0	-	1.0	-	1.0	-	1.0	-	1.0	-	1.0	-
Male	-	0.07 (-55.32, 55.46)	-	-	-	-	-	-	-	-	-	-	-	-	-	-
**Age - years**																
10 - <20	-	-	-	-	-	-	-	-	-	-	-	-	2.55 (0.13, 49.03)	0.47 (-7.37, 8.31)	1.87 (0.13, 26.91)	0.49 (0.07, 0.92)
20 - <30	-	-	0.7 (0.36, 1.37)	0.12 (-4.9, 5.15)	0.57 (0.26, 1.24)	0.1 (-6.72, 6.91)	0.61 (0.35, 1.07)	0.2 (-4.91, 5.31)	0.59 (0.33, 1.03)	0.2 (-4.3, 4.69)	0.64 (0.35, 1.19)	0.17 (-4.23, 4.58)	1.05 (0.61, 1.79)	0.34 (-5.42, 6.11)	1.08 (0.65, 1.8)	0.41 (0.35, 0.47)
30 - <40 (comparator)	1.0	0.15 (-119.04, 119.34)	1.0	0.16 (-6.27, 6.59)	1.0	0.14 (-9.85, 10.13)	1.0	0.27 (-6.53, 7.06)	1.0	0.27 (-5.84, 6.38)	1.0	0.22 (-5.41, 5.86)	1.0	0.34 (-5.32, 6)	1.0	0.39 (0.35, 0.44)
40 - <50	0.02 (0, 1.63)	0.01 (-9.42, 9.44)	1.27 (0.74, 2.18)	0.19 (-7.32, 7.69)	1.07 (0.59, 1.94)	0.15 (-10.3, 10.6)	0.89 (0.56, 1.43)	0.25 (-6.12, 6.61)	0.98 (0.61, 1.56)	0.26 (-5.77, 6.3)	0.69 (0.4, 1.18)	0.18 (-4.39, 4.76)	0.97 (0.59, 1.57)	0.33 (-5.25, 5.92)	1.36 (0.87, 2.14)	0.44 (0.39, 0.49)
50 - <60	0.11 (0, 2.81)	0.04 (-33.75, 33.83)	1.59 (0.83, 3.07)	0.21 (-8.4, 8.83)	1.57 (0.77, 3.18)	0.19 (-13.04, 13.41)	1.1 (0.6, 2.03)	0.28 (-6.88, 7.43)	1.42 (0.78, 2.59)	0.32 (-7, 7.64)	1.01 (0.52, 1.96)	0.23 (-5.44, 5.89)	1.34 (0.7, 2.58)	0.38 (-5.94, 6.7)	1.38 (0.74, 2.6)	0.44 (0.36, 0.53)
60 - <70	0.74 (0.04, 15.77)	0.13 (-101.8, 102.05)	1.55 (0.49, 4.91)	0.21 (-8.27, 8.69)	0.64 (0.17, 2.47)	0.11 (-7.3, 7.51)	4.39 (1.49, 12.97)	0.52 (-12.73, 13.76)	3.96 (1.34, 11.74)	0.5 (-10.89, 11.89)	4.09 (1.35, 12.38)	0.42 (-10.25, 11.09)	8.46 (2.26, 31.75)	0.64 (-10.06, 11.33)	9.33 (2.17, 40.07)	0.75 (0.54, 0.96)
70 - <80	2.54 (0.14, 45.65)	0.24 (-189.21, 189.68)	1.3 (0.3, 5.68)	0.19 (-7.43, 7.81)	0.67 (0.14, 3.26)	0.11 (-7.49, 7.71)	0.38 (0.08, 1.77)	0.15 (-3.6, 3.9)	0.53 (0.12, 2.31)	0.19 (-4.05, 4.42)	1.36 (0.31, 6.05)	0.26 (-6.34, 6.87)	0.1 (0.02, 0.62)	0.1 (-1.54, 1.74)	1.02 (0.23, 4.39)	0.4 (0.19, 0.61)
80 - <90	-	-	-	-	-	-	0.78 (0.09, 7.04)	0.23 (-5.69, 6.15)	0.86 (0.1, 7.76)	0.25 (-5.39, 5.88)	-	-	3.84 (0.25, 59.97)	0.53 (-8.3, 9.35)	-	-
90 - <100	-	-	-	-	-	-	-	-	-	-	-	-	-	-	-	-
100 - <110	-	-	-	-	-	-	-	-	-	-	-	-	-	-	-	-
**Length of Stay (months)**																
<1	1.0		1.0	0.13 (-5.24, 5.51)	1.0	0.09 (-6.3, 6.48)	1.0	0.19 (-4.69, 5.07)	1.0	0.2 (-4.42, 4.82)	1.0	0.18 (-4.36, 4.73)	1.0	0.44 (-6.88, 7.75)	1.0	0.46 (0.37, 0.54)
1-<6 (comparator)	-	0.07 (-57.62, 57.76)	-	0.13 (-5.02, 5.27)	-	0.09 (-6.19, 6.36)	-	0.2 (-4.81, 5.2)	-	0.21 (-4.57, 4.99)	-	0.15 (-3.73, 4.04)	-	0.31 (-4.86, 5.48)	-	0.37 (0.32, 0.42)
6-<12	4.37 (0.03, 611.38)	0.16 (-131.19, 131.52)	1.19 (0.61, 2.31)	0.14 (-5.67, 5.96)	1.21 (0.54, 2.72)	0.1 (-7.16, 7.37)	1.15 (0.66, 2.01)	0.21 (-5.26, 5.69)	1.24 (0.72, 2.16)	0.24 (-5.22, 5.7)	1.53 (0.81, 2.88)	0.2 (-4.83, 5.23)	1.38 (0.78, 2.43)	0.35 (-5.5, 6.2)	0.92 (0.55, 1.56)	0.36 (0.31, 0.42)
12-<24	4.77 (0.01, 1526.23)	0.17 (-136.62, 136.96)	1.81 (0.9, 3.63)	0.19 (-7.57, 7.95)	2.45 (1.12, 5.38)	0.17 (-11.89, 12.23)	1.92 (1.07, 3.45)	0.29 (-7.13, 7.71)	1.78 (0.99, 3.19)	0.29 (-6.39, 6.98)	1.79 (0.91, 3.5)	0.22 (-5.29, 5.73)	1.66 (0.91, 3.03)	0.37 (-5.89, 6.64)	1.85 (1.04, 3.29)	0.47 (0.39, 0.54)
24+	0.51 (0, 199.54)	0.04 (-35.84, 35.93)	2.53 (1.21, 5.31)	0.24 (-9.35, 9.82)	3.13 (1.39, 7.01)	0.2 (-13.92, 14.32)	3.35 (1.73, 6.49)	0.39 (-9.52, 10.3)	2.95 (1.53, 5.68)	0.38 (-8.27, 9.03)	3.16 (1.54, 6.5)	0.3 (-7.14, 7.73)	1.17 (0.58, 2.37)	0.33 (-5.17, 5.83)	2.63 (1.32, 5.26)	0.53 (0.43, 0.62)
**Ethnic Group**																
White (comparator)	1.0	0.04 (-35.29, 35.38)	1.0	0.16 (-6.44, 6.77)	1.0	0.13 (-9.29, 9.55)	1.0	0.25 (-6.19, 6.69)	1.0	0.26 (-5.74, 6.27)	1.0	0.21 (-4.98, 5.39)	1.0	0.35 (-5.48, 6.18)	1.0	0.43 (0.4, 0.46)
Mixed	-		0.27 (0.03, 2.29)	0.06 (-2.48, 2.61)	-		0.41 (0.08, 2.05)	0.15 (-3.57, 3.87)	0.37 (0.07, 1.88)	0.14 (-3.17, 3.45)	0.53 (0.1, 2.83)	0.14 (-3.45, 3.74)	0.51 (0.12, 2.25)	0.26 (-4.15, 4.68)	0.22 (0.05, 1.02)	0.23 (0.07, 0.4)
Asian or Asian British	196.3 (1.32, 29283.52)	0.53 (-422.77, 423.82)	6.69 (2.2, 20.32)	0.45 (-17.61, 18.51)	3.41 (1.06, 11.02)	0.27 (-18.9, 19.45)	3.83 (1.28, 11.46)	0.47 (-11.59, 12.53)	3.42 (1.13, 10.38)	0.46 (-10.08, 11)	3.66 (1.2, 11.16)	0.38 (-9.14, 9.9)	2.21 (0.67, 7.31)	0.46 (-7.19, 8.1)	1.88 (0.6, 5.86)	0.53 (0.35, 0.71)
Black or Black British	40.4 (2.09, 781.43)	0.34 (-269.48, 270.15)	0.87 (0.2, 3.81)	0.15 (-5.87, 6.17)	3.8 (0.92, 15.63)	0.29 (-19.94, 20.51)	1.46 (0.44, 4.88)	0.31 (-7.56, 8.18)	1.26 (0.36, 4.34)	0.3 (-6.48, 7.07)	2.45 (0.68, 8.78)	0.32 (-7.73, 8.37)	0.45 (0.12, 1.72)	0.25 (-3.9, 4.4)	0.64 (0.17, 2.39)	0.36 (0.18, 0.54)
Chinese and Other	-		1.45 (0.38, 5.48)	0.21 (-8.15, 8.57)	0.73 (0.15, 3.51)	0.11 (-7.49, 7.7)	1.1 (0.3, 4.07)	0.26 (-6.52, 7.05)	1.06 (0.28, 3.99)	0.27 (-5.92, 6.47)	0.74 (0.18, 2.97)	0.17 (-4.21, 4.55)	3.46 (0.7, 17.05)	0.52 (-8.19, 9.23)	1.62 (0.34, 7.83)	0.5 (0.25, 0.75)
Unclassified	-		1.44 (0.29, 7.23)	0.21 (-8.11, 8.52)	1.13 (0.2, 6.48)	0.14 (-10.02, 10.31)	0.66 (0.12, 3.77)	0.2 (-4.87, 5.27)	0.67 (0.12, 3.62)	0.21 (-4.59, 5.01)	1.2 (0.2, 7.22)	0.23 (-5.5, 5.95)	0.27 (0.03, 2.23)	0.19 (-3.02, 3.41)	0.24 (0.04, 1.45)	0.24 (0.04, 0.44)

Indicator	The proportion of people who have received three or more prescriptions for an antipsychotic in the preceding 12 months, and who have a record of blood testing (fasting glucose, HbA1c, lipid profile, urea and electrolytes, full blood count, liver function tests), body mass index and blood pressure			
	Group: Mental health			
	Univariate		Multivariable	
Variable	Odds Ratio (OR) of achieving 4 of the 6 blood tests (95% CI)		Odds Ratio (OR) of achieving 4 of the 6 blood tests (95% CI)	Probability of achieving 4 of the 6 blood tests (95% CI)
**Year**				
2017/18 (comparator)	1.0	-	1.0	0.31 (-9.66, 10.29)
2018/19	0.28	(0.19, 0.43)	0.29 (0.17, 0.48)	0.15 (-4.51, 4.81)
2019/20	0.10	(0.06, 0.16)	0.13 (0.07, 0.24)	0.08 (-2.57, 2.74)
**Prison**				
Prison 1	1.94	(0.52, 7.26)	0.81 (0.13, 4.91)	-
Prison 2	2.30	(0.87, 6.05)	2.33 (0.76, 7.1)	-
Prison 3	3.37	(1.32, 8.55)	2.47 (0.78, 7.8)	-
Prison 4	2.38	(0.85, 6.69)	1.83 (0.54, 6.2)	-
Prison 5	2.98	(0.26, 34.77)	3.57 (0.24, 53.28)	-
Prison 6	11.85	(3.05, 46.12)	7.26 (1.41, 37.34)	-
Prison 7	3.23	(1.22, 8.58)	3.91 (1.19, 12.82)	-
Prison 8	5.08	(1.98, 13.04)	4.81 (1.58, 14.65)	-
Prison 9	24.13	(9.77, 59.59)	8.55 (2.59, 28.19)	-
Prison 10	0.65	(0.13, 3.36)	0.68 (0.11, 4.09)	-
Prison 11 (comparator)	1.0	-	1.0	
Prison 12	-	-	-	
Prison 13	13.40	(4.97, 36.12)	4.53 (1.25, 16.37)	
**Prison category**				
A (comparator)	1.0	-	-	-
B	0.07	(0.04, 0.13)	-	-
C	0.14	(0.08, 0.24)	-	-
Closed	0.21	(0.12, 0.36)	-	-
D	0.45	(0.15, 1.32)	-	-
YOI	-	-	-	-
**Gender**				
Female (comparator)	1.0	-	-	-
Male	0.83	(0.52, 1.32)	-	-
**Age - years**				
10 - <20	-	-	-	-
20 - <30	0.60	(0.33, 1.09)	0.6 (0.3, 1.18)	0.13 (-3.87, 4.12)
30 - <40 (comparator)	1.0	-	1.0	0.17 (-5.39, 5.74)
40 - <50	1.08	(0.68, 1.72)	0.96 (0.55, 1.67)	0.17 (-5.25, 5.59)
50 - <60	2.24	(1.29, 3.88)	1.41 (0.72, 2.75)	0.21 (-6.6, 7.03)
60 - <70	3.31	(1.28, 8.55)	1.52 (0.46, 5.04)	0.22 (-6.89, 7.34)
70 - <80	1.74	(0.5, 6.1)	1.12 (0.25, 5.04)	0.19 (-5.78, 6.15)
80 - <90	-	-	-	-
90 - <100	-	-	-	-
100 - <110	-	-	-	-
**Length of Stay (months)**				
<1	0.72	(0.3, 1.73)	1.15 (0.44, 3.01)	0.13 (-3.93, 4.18)
1-<6 (comparator)	1.0	-	1.0	0.11 (-3.54, 3.77)
6-<12	1.73	(0.96, 3.11)	1.44 (0.73, 2.87)	0.15 (-4.6, 4.9)
12-<24	2.23	(1.23, 4.03)	1.98 (0.97, 4.03)	0.18 (-5.67, 6.04)
24+	6.85	(4.02, 11.67)	3.48 (1.66, 7.31)	0.26 (-7.99, 8.51)
**Ethnic Group**				
White (comparator)	1.0	-	1.0	0.16 (-5.01, 5.34)
Mixed	0.44	(0.05, 3.62)	0.29 (0.03, 2.52)	0.07 (-2.13, 2.27)
Asian or Asian British	6.74	(2.49, 18.28)	5.67 (1.84, 17.46)	0.4 (-12.37, 13.18)
Black or Black British	2.52	(0.85, 7.48)	4.04 (1.12, 14.54)	0.35 (-10.68, 11.37)
Chinese and Other	2.55	(0.76, 8.59)	1.18 (0.3, 4.61)	0.18 (-5.56, 5.92)
Unclassified	2.87	(0.74, 11.16)	1.85 (0.35, 9.85)	0.23 (-7.2, 7.67)

Indicator *The proportion of the prison population prescribed three or more psychotropic drugs (antipsychotics, sedative anti-depressants, hypnotics and anxiolytics, pregabalin or gabapentin, opioids) at the same time during an eight-week period*

Group: Mental health

	Univariate				Multivariable							
	3 or more Drugs		4 or more Drugs		3 or more Drugs				4 or more Drugs			
**Variable**	**Odds Ratio (OR) of achieving indicator**	**(95% CI) of OR**	**Odds Ratio (OR) of achieving indicator**	**(95% CI) of OR**	**OR**	**(95% CI)**	**Probability of achieving indicator**	**(95% CI) of probability**	**OR**	**(95% CI)**	**Probability of achieving indicator**	**(95% CI) of probability**
**Year**												
2017/18 (comparator)	1.0	-	1.0	-	1.0	-	0.006	(-0.046, 0.058)	1.0	-	0.002	(0.001, 0.003)
2018/19	1.36	(1.07, 1.73)	1.46	(0.97, 2.19)	1.38	(1.07, 1.79)	0.008	(-0.063, 0.079)	1.45	(0.95, 2.2)	0.003	(0.002, 0.005)
2019/20	1.60	(1.27, 2)	2.22	(1.54, 3.21)	1.76	(1.37, 2.25)	0.011	(-0.079, 0.1)	2.30	(1.56, 3.39)	0.005	(0.003, 0.008)
**Prison**												
Prison 1	1.34	(0.79, 2.3)	0.78	(0.26, 2.32)	0.89	(0.48, 1.64)	0.007	(-0.051, 0.064)	0.41	(0.12, 1.47)	0.001	(-0.001, 0.004)
Prison 2	4.42	(3.16, 6.19)	5.00	(2.9, 8.63)	2.94	(2.02, 4.3)	0.022	(-0.163, 0.206)	3.30	(1.79, 6.1)	0.01	(-0.007, 0.028)
Prison 3	1.67	(1.11, 2.51)	0.70	(0.29, 1.68)	0.93	(0.58, 1.49)	0.007	(-0.053, 0.067)	0.34	(0.13, 0.91)	0.001	(-0.001, 0.003)
Prison 4	1.56	(1.02, 2.4)	1.53	(0.76, 3.11)	0.71	(0.44, 1.17)	0.005	(-0.041, 0.052)	0.58	(0.26, 1.31)	0.002	(-0.002, 0.005)
Prison 5	0.60	(0.22, 1.67)	-	-	0.25	(0.08, 0.83)	0.002	(-0.015, 0.018)	-	-	-	-
Prison 6	0.75	(0.4, 1.42)	0.17	(0.02, 1.31)	0.27	(0.12, 0.62)	0.002	(-0.016, 0.02)	0.10	(0.01, 0.75)	0.0003	(-0.001, 0.001)
Prison 7	4.25	(2.7, 6.69)	2.86	(1.23, 6.64)	0.85	(0.01, 74.82)	0.006	(-0.052, 0.065)	0.48	(0, 882.55)	0.001	(-0.007, 0.01)
Prison 8	8.11	(5.71, 11.52)	15.06	(8.84, 25.64)	1.37	(0.02, 119.2)	0.01	(-0.083, 0.104)	2.31	(0.001, 4060)	0.007	(-0.033, 0.047)
Prison 9	1.87	(1.11, 3.17)	1.54	(0.61, 3.92)	0.85	(0.46, 1.6)	0.006	(-0.049, 0.062)	0.62	(0.22, 1.76)	0.002	(-0.002, 0.006)
Prison 10	0.60	(0.35, 1.03)	1.21	(0.6, 2.46)	0.52	(0.29, 0.92)	0.004	(-0.03, 0.038)	1.10	(0.52, 2.36)	0.003	(-0.003, 0.01)
Prison 11 (comparator)	1.0	-	1.0	-	1.0	-	0.008	(-0.057, 0.072)	1.0	-	0.003	(-0.003, 0.009)
Prison 12	-	-	-	-	-	-	-	-	-	-	-	-
Prison 13	2.19	(1.24, 3.88)	1.88	(0.69, 5.11)	0.97	(0.46, 2.01)	0.007	(-0.055, 0.07)	0.50	(0.13, 1.89)	0.002	(-0.002, 0.005)
**Prison category**												
A (comparator)	1.0	-	1.0	-								
B	0.92	(0.65, 1.32)	1.30	(0.69, 2.43)								
C	0.78	(0.53, 1.14)	0.61	(0.3, 1.25)								
Closed	3.33	(2.3, 4.82)	6.22	(3.3, 11.72)								
D	0.35	(0.2, 0.64)	0.07	(0.01, 0.57)								
YOI	-	-	-	-								
**Gender**												
Female (comparator)	1.0	-	1.0	-	1.0	-	0.023	(-0.189, 0.235)	1.0	-	0.009	(-0.049, 0.068)
Male	0.21	(0.17, 0.26)	0.12	(0.09, 0.16)	0.29	(0, 24.78)	0.007	(-0.051, 0.064)	0.29	(0, 492.45)	0.003	(0, 0.006)
**Age - years**												
10 - <20	-	-	-	-	-	-			-	-		
20 - <30	0.40	(0.3, 0.52)	0.41	(0.27, 0.62)	0.51	(0.38, 0.69)	0.005	(-0.035, 0.044)	0.56	(0.36, 0.87)	0.002	(0.001, 0.003)
30 - <40 (comparator)	1.0	-	1.0	-	1.0	-	0.009	(-0.068, 0.086)	1.0	-	0.004	(0.002, 0.006)
40 - <50	1.31	(1.05, 1.62)	1.47	(1.06, 2.03)	1.26	(0.99, 1.59)	0.011	(-0.085, 0.107)	1.41	(0.99, 2)	0.006	(0.003, 0.008)
50 - <60	1.15	(0.86, 1.54)	0.71	(0.41, 1.23)	1.07	(0.78, 1.48)	0.01	(-0.072, 0.092)	0.67	(0.38, 1.21)	0.003	(0.001, 0.004)
60 - <70	0.80	(0.46, 1.38)	0.70	(0.28, 1.72)	0.78	(0.42, 1.45)	0.007	(-0.053, 0.067)	0.87	(0.35, 2.2)	0.003	(0.0002, 0.007)
70 - <80	0.74	(0.27, 1.99)	0.90	(0.22, 3.66)	0.88	(0.32, 2.41)	0.008	(-0.0603, 0.076)	1.09	(0.26, 4.54)	0.004	(-0.002, 0.01)
80 - <90	0.87	(0.12, 6.27)	-	-	0.74	(0.1, 5.38)	0.007	(-0.052, 0.065)	-	-	-	-
90 - <100	-	-	-	-	-	-	-	-	-	-	-	-
100 - <110	-	-	-	-	-	-	-	-	-	-	-	-
**Length of Stay (months)**												
<1	0.46	(0.33, 0.64)	0.42	(0.24, 0.71)	0.43	(0.31, 0.62)	0.003	(-0.025, 0.031)	0.38	(0.21, 0.67)	0.001	(0.0004, 0.002)
1-<6 (comparator)	1.0	-	1.0	-	1.0	-	0.007	(-0.056, 0.071)	1.0	-	0.003	(0.002, 0.004)
6-<12	1.35	(1.07, 1.72)	1.52	(1.06, 2.19)	1.64	(1.26, 2.14)	0.012	(-0.091, 0.115)	2.19	(1.48, 3.24)	0.006	(0.004, 0.009)
12-<24	1.63	(1.28, 2.07)	1.61	(1.1, 2.37)	2.16	(1.64, 2.85)	0.016	(-0.119, 0.151)	2.82	(1.84, 4.32)	0.008	(0.004, 0.012)
24+	1.08	(0.78, 1.48)	1.15	(0.7, 1.89)	1.22	(0.83, 1.8)	0.009	(-0.068, 0.086)	1.92	(1.07, 3.42)	0.006	(0.002, 0.009)
**Ethnic Group**												
White (comparator)	1.0	-	1.0	-	1.0	-	0.009	(-0.066, 0.084)	1.0	-	0.004	(0.002, 0.005)
Mixed	0.38	(0.14, 1.02)	0.46	(0.11, 1.85)	0.40	(0.15, 1.07)	0.004	(-0.027, 0.034)	0.44	(0.11, 1.78)	0.002	(-0.001, 0.004)
Asian or Asian British	0.15	(0.05, 0.47)	0.12	(0.02, 0.87)	0.22	(0.07, 0.69)	0.002	(-0.015, 0.019)	0.18	(0.03, 1.29)	0.001	(-0.001, 0.002)
Black or Black British	0.47	(0.19, 1.13)	-	-	0.40	(0.15, 1.07)	0.004	(-0.027, 0.034)	-	-		
Chinese and Other	0.65	(0.21, 2.03)	1.59	(0.5, 4.98)	0.57	(0.18, 1.78)	0.005	(-0.038, 0.048)	1.29	(0.41, 4.08)	0.005	(-0.001, 0.011)
Unclassified	0.49	(0.2, 1.18)	0.71	(0.23, 2.22)	0.72	(0.29, 1.79)	0.006	(-0.049, 0.061)	1.41	(0.43, 4.61)	0.005	(-0.001, 0.012)

Indicator ***The proportion of people aged over 55 years in the prison population who have a record of one of the following assessments in the preceding 12 months; Mini Mental State Examination, 6 item Cognitive Impairment Test, General practitioner Assessment of Cognition***

Group: Mental health

	Univariate		Multivariable			
**Variable**	**Odds Ratio (OR) of achieving indicator**	**(95% CI) of OR**	**OR**	**(95% CI)**	**Probability of achieving indicator**	**(95% CI) of probability**
**Year**						
2017/18 (comparator)	1.0	-	1.0	-	0.01	(0.01, 0.02)
2018/19	5.84	(3.72, 9.15)	8.51	(5.29, 13.72)	0.09	(0.08, 0.1)
2019/20	18.31	(11.95, 28.05)	40.47	(25.34, 64.64)	0.23	(0.22, 0.25)
**Prison**						
Prison 1	85.17	(24.74, 293.23)	34.95	(9.78, 124.91)	-	-
Prison 2	232.22	(72.98, 738.93)	109.76	(34.23, 351.92)	-	-
Prison 3	1.12	(0.19, 6.74)	0.65	(0.11, 3.97)	-	-
Prison 4	2.19	(0.54, 8.81)	0.86	(0.2, 3.74)	-	-
Prison 5	1.71	(0.18, 16.61)	0.87	(0.09, 8.63)	-	-
Prison 6	111.62	(34.51, 360.98)	44.33	(13.3, 147.78)	-	-
Prison 7	1.70	(0.17, 16.58)	-	-	-	-
Prison 8	37.88	(11.24, 127.67)	17.79	(5.16, 61.39)	-	-
Prison 9	9.09	(2.71, 30.52)	3.30	(0.92, 11.88)	-	-
Prison 10	142.14	(44.7, 451.95)	70.34	(21.83, 226.7)	-	-
Prison 11 (comparator)	1.0	-	1.0	-	-	-
Prison 12	-	-	-	-	-	-
Prison 13	7.24	(1.97, 26.61)	2.30	(0.55, 9.63)	-	-
**Prison category**						
A (comparator)	1.0	-				
B	8.41	(5.79, 12.2)				
C	0.81	(0.5, 1.34)				
Closed	2.52	(1.48, 4.3)				
D	6.87	(4.44, 10.64)				
YOI	-	-				
**Gender**						
Female (comparator)	1.0	-	-	-		
Male	1.04	(0.68, 1.59)	-	-		
**Age - years**						
10 - <20	-	-	-	-	-	-
20 - <30	-	-	-	-	-	-
30 - <40	-	-	-	-	-	-
40 - <50	-	-	-	-	-	-
50 - <60 (comparator)	1.0	-	1.0	-	0.10	(0.09, 0.11)
60 - <70	0.90	(0.73, 1.11)	1.30	(0.99, 1.71)	0.11	(0.1, 0.13)
70 - <80	0.99	(0.74, 1.33)	1.60	(1.07, 2.41)	0.13	(0.1, 0.15)
80 - <90	1.20	(0.7, 2.08)	0.99	(0.49, 1.98)	0.10	(0.06, 0.14)
90 - <100	4.58	(0.76, 27.58)	6.53	(0.39, 108.71)	0.24	(-0.03, 0.51)
100 - <110	-	-	-	-		
**Length of Stay (months)**						
<1	0.60	(0.45, 0.79)	0.65	(0.45, 0.94)	0.08	(0.07, 0.1)
1-<6 (comparator)	1.0	-	1.0	-	0.11	(0.09, 0.12)
6-<12	0.89	(0.67, 1.17)	1.29	(0.89, 1.86)	0.12	(0.1, 0.14)
12-<24	0.70	(0.53, 0.92)	1.32	(0.89, 1.97)	0.12	(0.1, 0.14)
24+	0.22	(0.16, 0.3)	1.09	(0.67, 1.77)	0.11	(0.09, 0.14)
**Ethnic Group**						
White (comparator)	1.0	-	1.0	-	0.11	(0.1, 0.12)
Mixed	1.05	(0.49, 2.29)	1.11	(0.43, 2.88)	0.11	(0.05, 0.17)
Asian or Asian British	1.57	(0.76, 3.21)	1.07	(0.46, 2.53)	0.11	(0.06, 0.17)
Black or Black British	1.12	(0.56, 2.26)	0.89	(0.39, 2.03)	0.10	(0.05, 0.15)
Chinese and Other	-	-	-	-	-	-
Unclassified	0.22	(0.05, 0.91)	0.68	(0.15, 3.2)	0.09	(0.003, 0.17)

Indicator ***The proportion of new receptions to prison accepting opt out dry blood spot testing***

Group: Communicable Disease

	Univariate		Multivariable			
**Variable**	**Odds Ratio (OR) of achieving indicator**	**(95% CI) of OR**	**OR**	**(95% CI)**	**Probability of achieving indicator**	**(95% CI) of probability**
**Year**						
2017/18 (comparator)	1.0	-	1.0	-	0.01	(-0.01, 0.03)
2018/19	38.99	(32.95, 46.12)	75.98	(61.25, 94.25)	0.33	(-0.3, 0.96)
2019/20	72.48	(61.31, 85.69)	212.13	(170.37, 264.13)	0.51	(-0.46, 1.48)
**Prison**						
Prison 1	10.77	(9.47, 12.25)	8.45	(7.03, 10.14)	0.47	(-0.42, 1.37)
Prison 2	5.86	(5.33, 6.45)	5.45	(4.84, 6.14)	0.42	(-0.37, 1.21)
Prison 3	1.36	(1.22, 1.51)	0.97	(0.83, 1.12)	0.18	(-0.16, 0.52)
Prison 4	2.61	(2.35, 2.9)	1.45	(1.25, 1.68)	0.23	(-0.21, 0.67)
Prison 5	3.19	(2.71, 3.74)	1.44	(1.13, 1.84)	0.23	(-0.21, 0.67)
Prison 6	2.75	(2.45, 3.08)	1.84	(1.57, 2.16)	0.26	(-0.24, 0.77)
Prison 7	5.04	(4.27, 5.94)	3.00	(0.27, 33.3)	0.33	(-0.37, 1.04)
Prison 8	10.62	(9.23, 12.21)	4.53	(0.41, 50.69)	0.39	(-0.41, 1.2)
Prison 9	1.90	(1.54, 2.35)	1.34	(1.04, 1.71)	0.22	(-0.2, 0.64)
Prison 10	0.06	(0.04, 0.07)	0.05	(0.04, 0.07)	0.01	(-0.01, 0.04)
Prison 11 (comparator)	1.0	-	1.0	-	0.18	(-0.17, 0.53)
Prison 12	3.79	(3.23, 4.45)	2.10	(1.64, 2.7)	0.28	(-0.26, 0.82)
Prison 13	0.22	(0.15, 0.33)	0.14	(0.09, 0.22)	0.04	(-0.04, 0.11)
**Prison category**						
A (comparator)	1.0	-				
B	1.31	(1.1, 1.55)				
C	2.95	(2.48, 3.51)				
Closed	8.09	(6.68, 9.79)				
D	2.94	(2.45, 3.54)				
YOI	3.87	(3.1, 4.83)				
**Gender**						
Female (comparator)	1.0	-	1.0	-	0.31	(-0.33, 0.95)
Male	0.20	(0.18, 0.23)	0.5	(0.05, 5.77)	0.24	(-0.22, 0.69)
**Age - years**						
10 - <20	0.87	(0.73, 1.03)	1.31	(0.96, 1.78)	0.28	(-0.25, 0.8)
20 - <30	1.03	(0.96, 1.09)	1.04	(0.95, 1.14)	0.25	(-0.23, 0.73)
30 - <40 (comparator)	1.0	-	1.0	-	0.25	(-0.22, 0.71)
40 - <50	1.03	(0.96, 1.11)	0.95	(0.86, 1.06)	0.24	(-0.22, 0.7)
50 - <60	0.96	(0.86, 1.06)	0.90	(0.78, 1.04)	0.24	(-0.21, 0.68)
60 - <70	0.81	(0.68, 0.97)	1.03	(0.8, 1.32)	0.25	(-0.22, 0.72)
70 - <80	0.73	(0.53, 1.01)	0.82	(0.52, 1.29)	0.23	(-0.2, 0.66)
80 - <90	0.83	(0.41, 1.69)	0.84	(0.31, 2.25)	0.23	(-0.22, 0.67)
90 - <100	2.18	(0.15, 32.8)	1.84	(0.07, 50.53)	0.31	(-0.38, 1.01)
100 - <110	-	-	-	-	-	-
**Length of Stay (months)**						
<1	0.50	(0.47, 0.54)	0.53	(0.48, 0.58)	0.19	(-0.17, 0.55)
1-<6 (comparator)	1.0	-	1.0	-	0.26	(-0.23, 0.74)
6-<12	1.36	(1.26, 1.47)	1.36	(1.22, 1.51)	0.29	(-0.26, 0.83)
12-<24	1.22	(1.1, 1.35)	1.30	(1.14, 1.5)	0.28	(-0.25, 0.82)
24+	6.78	(4.61, 9.96)	10.15	(6.73, 15.31)	0.49	(-0.44, 1.42)
**Ethnic Group**						
White (comparator)	1.0	-	1.0	-	0.25	(-0.22, 0.72)
Mixed	1.16	(0.95, 1.43)	0.85	(0.66, 1.08)	0.23	(-0.21, 0.66)
Asian or Asian British	0.91	(0.78, 1.07)	1.10	(0.9, 1.33)	0.26	(-0.23, 0.74)
Black or Black British	1.21	(0.99, 1.48)	0.94	(0.75, 1.2)	0.24	(-0.22, 0.7)
Chinese and Other	0.92	(0.68, 1.25)	0.76	(0.53, 1.09)	0.22	(-0.2, 0.63)
Unclassified	0.71	(0.57, 0.89)	1.06	(0.81, 1.38)	0.25	(-0.23, 0.74)

Indicator		*The proportion of the prison population who have received one, two, three or four (course of three plus booster) Hepatitis B vaccinations in the preceding 12 months*							
	**Group: Communicable Disease**								
		**Univariate**							
		**Hepatitis B vaccines of the population who entered in the last 12 months - Odds Ratio (OR) of achieving indicator (95% CI)**							
	**Variable**	**1st Hep B**		**1st and 2nd Hep B**		**1st, 2nd and 3rd Hep B**		**1st, 2nd, 3rd and 4th Hep B**	
**Year**									
	2017/18 (comparator)	1.0	(0.85, 0.93)	1.0	-	1.0	-	1.0	-
	2018/19	0.89	(1.89, 2.07)	0.99	(0.93, 1.04)	0.42	(0.39, 0.46)	1.40	(1.28, 1.53)
	2019/20	1.98	(0.41, 0.44)	2.08	(1.98, 2.19)	2.12	(2.01, 2.24)	2.27	(2.09, 2.46)
**Prison**									
	Prison 1	1.58	(1.44, 1.73)	1.88	(1.71, 2.07)	2.30	(2.06, 2.56)	1.78	(1.54, 2.05)
	Prison 2	0.81	(0.76, 0.87)	0.88	(0.81, 0.95)	0.91	(0.83, 0.99)	0.76	(0.67, 0.87)
	Prison 3	1.43	(1.33, 1.54)	1.59	(1.47, 1.72)	1.79	(1.64, 1.96)	1.95	(1.75, 2.18)
	Prison 4	2.09	(1.93, 2.25)	2.37	(2.19, 2.57)	2.58	(2.35, 2.83)	2.17	(1.93, 2.44)
	Prison 5	1.86	(1.64, 2.12)	2.21	(1.93, 2.52)	2.54	(2.19, 2.95)	1.71	(1.4, 2.1)
	Prison 6	2.07	(1.9, 2.25)	2.50	(2.29, 2.74)	2.54	(2.29, 2.82)	1.68	(1.46, 1.94)
	Prison 7	4.54	(4.05, 5.1)	4.48	(4, 5.03)	4.65	(4.11, 5.27)	3.36	(2.89, 3.9)
	Prison 8	1.77	(1.62, 1.94)	1.78	(1.62, 1.95)	1.66	(1.49, 1.86)	1.24	(1.06, 1.45)
	Prison 9	1.90	(1.63, 2.22)	2.12	(1.81, 2.49)	2.63	(2.2, 3.13)	2.69	(2.18, 3.31)
	Prison 10	1.41	(1.32, 1.51)	1.36	(1.26, 1.46)	1.49	(1.37, 1.62)	1.06	(0.95, 1.2)
	Prison 11 (comparator)	1.0	-	1.0	-	1.0	-	1.0	-
	Prison 12	2.74	(2.43, 3.08)	3.39	(3.01, 3.82)	2.99	(2.61, 3.42)	1.14	(0.91, 1.42)
	Prison 13	1.42	(1.19, 1.7)	1.57	(1.3, 1.89)	1.62	(1.31, 2.01)	1.40	(1.05, 1.86)
**Prison category**									
	A (comparator)	1.0	-	1.0	-	1.0	-	1.0	-
	B	0.63	(0.56, 0.7)	0.57	(0.5, 0.64)	0.51	(0.45, 0.59)	0.46	(0.39, 0.54)
	C	0.99	(0.88, 1.11)	1.02	(0.9, 1.15)	1.00	(0.88, 1.15)	0.95	(0.8, 1.13)
	Closed	1.47	(1.29, 1.67)	1.34	(1.18, 1.53)	1.17	(1.01, 1.35)	0.92	(0.77, 1.11)
	D	1.20	(1.05, 1.36)	1.30	(1.14, 1.48)	1.19	(1.02, 1.38)	0.81	(0.67, 0.98)
	YOI	1.63	(1.4, 1.91)	1.82	(1.55, 2.14)	1.39	(1.16, 1.67)	0.54	(0.42, 0.71)
**Gender**									
	Female (comparator)	1.0	-	-	-	-	-	-	-
	Male	0.61	(0.57, 0.66)	0.7	(0.62, 0.72)	0.7	(0.68, 0.81)	0.8	(0.71, 0.89)
**Age - years**									
	10 - <20	0.91	(0.8, 1.04)	1.0	(0.83, 1.1)	0.9	(0.72, 1)	0.3	(0.22, 0.44)
	20 - <30	0.98	(0.94, 1.03)	1.0	(0.96, 1.06)	1.0	(0.93, 1.04)	0.9	(0.87, 1)
	30 - <40 (comparator)	1.0	-	1.0	-	1.0	-	1.0	-
	40 - <50	0.96	(0.9, 1.01)	1.0	(0.9, 1.02)	1.0	(0.91, 1.04)	0.9	(0.86, 1.03)
	50 - <60	0.91	(0.84, 0.98)	0.9	(0.86, 1.01)	1.0	(0.87, 1.05)	0.8	(0.71, 0.93)
	60 - <70	0.78	(0.68, 0.89)	0.8	(0.68, 0.92)	0.8	(0.65, 0.92)	0.5	(0.38, 0.66)
	70 - <80	0.62	(0.47, 0.82)	0.6	(0.46, 0.83)	0.7	(0.52, 1.01)	0.5	(0.32, 0.9)
	80 - <90	0.49	(0.27, 0.88)	0.5	(0.25, 0.92)	0.5	(0.24, 1.09)	0.8	(0.3, 1.87)
	90 - <100	0.80	(0.08, 7.72)	1.2	(0.12, 11.13)	2.3	(0.23, 23.28)	-	-
	100 - <110	-	-	-	-	-	-	-	-
**Length of Stay (months)**									
	<1	0.62	(0.59, 0.65)	0.6	(0.53, 0.59)	0.5	(0.49, 0.55)	0.7	(0.64, 0.75)
	1-<6 (comparator)	1.0	-	1.0	-	1.0	-	1.0	-
	6-<12	1.26	(1.19, 1.33)	1.4	(1.28, 1.44)	1.4	(1.34, 1.53)	1.4	(1.24, 1.49)
	12-<24	1.97	(1.82, 2.12)	2.2	(2.04, 2.39)	2.2	(1.98, 2.4)	2.6	(2.29, 2.87)
	24+	2.03	(1.75, 2.36)	2.2	(1.89, 2.58)	2.4	(2.01, 2.78)	3.1	(2.45, 3.84)
**Ethnic Group**									
	White (comparator)	1.0	-	1.0	-	1.0	-	1.0	-
	Mixed	0.93	(0.8, 1.09)	0.9	(0.77, 1.07)	0.8	(0.7, 1.02)	0.9	(0.7, 1.13)
	Asian or Asian British	0.98	(0.88, 1.1)	1.0	(0.85, 1.08)	1.0	(0.84, 1.1)	0.9	(0.74, 1.06)
	Black or Black British	0.77	(0.66, 0.9)	0.8	(0.68, 0.95)	0.8	(0.62, 0.9)	0.6	(0.45, 0.78)
	Chinese and Other	0.66	(0.52, 0.83)	0.7	(0.52, 0.86)	0.6	(0.43, 0.8)	0.5	(0.3, 0.74)
	Unclassified	0.63	(0.53, 0.74)	0.6	(0.52, 0.75)	0.5	(0.41, 0.65)	0.5	(0.35, 0.65)

Indicator	*The proportion of the prison population who have received one, two, three or four (course of three plus booster) Hepatitis B vaccinations in the preceding 12 months*							
**Domain: Communicable Disease**								
	**Multivariable**							
	**Hepatitis B vaccines of the population who entered in the last 12 months**							
**Variable**	**1st Hep B**		**1st and 2nd Hep B**		**1st, 2nd and 3rd Hep B**		**1st, 2nd, 3rd and 4th Hep B**	
	**OR (95% CI)**	**Probability of achieving indicator (95% CI)**	**OR (95% CI)**	**Probability of achieving indicator (95% CI)**	**OR (95% CI)**	**Probability of achieving indicator (95% CI)**	**OR (95% CI)**	**Probability of achieving indicator (95% CI)**
**Year**								
2017/18 (comparator)	1.0	0.33 (0.26, 0.39)	1.0	0.24 (0.24, 0.25)	1.0	0.19 (0.04, 0.34)	1.0	0.07 (-0.03, 0.16)
2018/19	0.93 (0.88, 0.98)	0.31 (0.25, 0.38)	1.05 (0.98, 1.11)	0.25 (0.24, 0.26)	0.46 (0.43, 0.5)	0.1 (0.02, 0.18)	1.56 (1.41, 1.72)	0.1 (-0.04, 0.23)
2019/20	2.45 (2.32, 2.6)	0.53 (0.43, 0.64)	2.67 (2.52, 2.84)	0.45 (0.44, 0.46)	2.73 (2.56, 2.91)	0.37 (0.08, 0.67)	3.18 (2.89, 3.49)	0.18 (-0.07, 0.42)
**Prison**								
Prison 1	1.53 (1.36, 1.72)	0.49 (0.39, 0.59)	1.64 (1.45, 1.85)	0.41 (0.38, 0.44)	1.88 (1.64, 2.14)	0.32 (0.07, 0.57)	1.59 (1.35, 1.86)	0.17 (-0.07, 0.4)
Prison 2	0.52 (0.48, 0.56)	0.26 (0.2, 0.31)	0.55 (0.5, 0.6)	0.2 (0.18, 0.21)	0.55 (0.5, 0.61)	0.13 (0.03, 0.24)	0.48 (0.42, 0.54)	0.06 (-0.02, 0.14)
Prison 3	1.15 (1.05, 1.25)	0.42 (0.34, 0.51)	1.2 (1.1, 1.32)	0.34 (0.32, 0.37)	1.32 (1.19, 1.47)	0.25 (0.05, 0.45)	1.53 (1.35, 1.73)	0.16 (-0.06, 0.39)
Prison 4	1.2 (1.09, 1.32)	0.43 (0.34, 0.52)	1.29 (1.17, 1.42)	0.36 (0.33, 0.38)	1.38 (1.24, 1.54)	0.26 (0.06, 0.47)	1.45 (1.27, 1.66)	0.16 (-0.06, 0.38)
Prison 5	1.26 (1.07, 1.48)	0.45 (0.35, 0.54)	1.5 (1.27, 1.77)	0.39 (0.35, 0.43)	1.67 (1.39, 2)	0.29 (0.06, 0.53)	1.37 (1.08, 1.72)	0.15 (-0.06, 0.36)
Prison 6	1.83 (1.63, 2.04)	0.53 (0.42, 0.64)	2.03 (1.81, 2.27)	0.46 (0.43, 0.49)	1.93 (1.7, 2.19)	0.32 (0.07, 0.58)	1.31 (1.12, 1.54)	0.14 (-0.06, 0.35)
Prison 7	2.04 (0.97, 4.28)	0.56 (0.36, 0.75)	2.03 (0.92, 4.52)	0.46 (0.29, 0.63)	2.14 (0.83, 5.49)	0.34 (0.02, 0.66)	1.29 (0.35, 4.66)	0.14 (-0.1, 0.38)
Prison 8	0.88 (0.42, 1.83)	0.36 (0.2, 0.53)	0.95 (0.43, 2.09)	0.29 (0.15, 0.43)	0.84 (0.33, 2.15)	0.18 (0, 0.37)	0.44 (0.12, 1.61)	0.06 (-0.04, 0.15)
Prison 9	1.08 (0.9, 1.28)	0.41 (0.32, 0.5)	1.12 (0.93, 1.34)	0.33 (0.29, 0.37)	1.31 (1.08, 1.59)	0.25 (0.05, 0.45)	1.5 (1.19, 1.88)	0.16 (-0.06, 0.39)
Prison 10	1.05 (0.97, 1.13)	0.4 (0.32, 0.49)	0.97 (0.89, 1.05)	0.3 (0.28, 0.32)	1 (0.91, 1.09)	0.21 (0.04, 0.37)	0.81 (0.72, 0.92)	0.1 (-0.04, 0.23)
Prison 11 (comparator)	1.0	0.39 (0.31, 0.47)	1.0	0.3 (0.29, 0.32)	1.0	0.21 (0.04, 0.37)	1.0	0.12 (-0.05, 0.28)
Prison 12	1.66 (1.42, 1.94)	0.51 (0.4, 0.62)	1.91 (1.63, 2.24)	0.45 (0.41, 0.49)	1.56 (1.3, 1.87)	0.28 (0.06, 0.51)	0.95 (0.74, 1.23)	0.11 (-0.04, 0.27)
Prison 13	1.15 (0.93, 1.42)	0.42 (0.33, 0.52)	1.16 (0.93, 1.44)	0.33 (0.29, 0.38)	1.12 (0.87, 1.42)	0.23 (0.04, 0.41)	0.99 (0.73, 1.35)	0.11 (-0.05, 0.28)
**Prison category**								
A (comparator)	-		-		-		-	
B	-		-		-		-	
C	-		-		-		-	
Closed	-		-		-		-	
D	-		-		-		-	
YOI	-		-		-		-	
**Gender**								
Female (comparator)	1.0	0.46 (0.29, 0.64)	1.0	0.36 (0.21, 0.51)	1.0	0.26 (0.01, 0.51)	1.0	0.18 (-0.12, 0.48)
Male	0.71 (0.34, 1.48)	0.39 (0.31, 0.47)	0.79 (0.36, 1.73)	0.31 (0.3, 0.33)	0.78 (0.3, 1.98)	0.22 (0.05, 0.39)	0.52 (0.15, 1.88)	0.11 (-0.04, 0.26)
**Age - years**								
10 - <20	0.55 (0.46, 0.66)	0.28 (0.22, 0.35)	0.58 (0.48, 0.71)	0.22 (0.19, 0.26)	0.6 (0.47, 0.75)	0.15 (0.03, 0.28)	0.35 (0.23, 0.52)	0.04 (-0.02, 0.11)
20 - <30	1.03 (0.98, 1.09)	0.41 (0.33, 0.49)	1.05 (0.99, 1.11)	0.33 (0.32, 0.34)	1.07 (1, 1.14)	0.23 (0.05, 0.42)	1.07 (0.99, 1.16)	0.12 (-0.05, 0.29)
30 - <40 (comparator)	1.0	0.4 (0.32, 0.49)	1.0	0.32 (0.31, 0.33)	1.0	0.22 (0.05, 0.4)	1.0	0.12 (-0.04, 0.28)
40 - <50	0.92 (0.86, 0.98)	0.39 (0.31, 0.46)	0.9 (0.84, 0.96)	0.3 (0.29, 0.31)	0.93 (0.86, 1.01)	0.21 (0.05, 0.38)	0.9 (0.82, 0.99)	0.11 (-0.04, 0.25)
50 - <60	0.84 (0.76, 0.91)	0.37 (0.29, 0.44)	0.84 (0.77, 0.92)	0.29 (0.27, 0.31)	0.91 (0.82, 1.01)	0.21 (0.04, 0.37)	0.83 (0.72, 0.95)	0.1 (-0.04, 0.23)
60 - <70	0.75 (0.64, 0.88)	0.34 (0.27, 0.42)	0.77 (0.65, 0.91)	0.27 (0.24, 0.3)	0.72 (0.59, 0.89)	0.18 (0.04, 0.32)	0.55 (0.41, 0.74)	0.07 (-0.03, 0.17)
70 - <80	0.58 (0.42, 0.79)	0.29 (0.21, 0.37)	0.55 (0.39, 0.78)	0.22 (0.16, 0.27)	0.64 (0.44, 0.95)	0.16 (0.03, 0.3)	0.43 (0.24, 0.78)	0.05 (-0.03, 0.14)
80 - <90	0.48 (0.25, 0.92)	0.26 (0.13, 0.38)	0.43 (0.21, 0.9)	0.18 (0.08, 0.28)	0.41 (0.17, 0.99)	0.11 (-0.01, 0.24)	0.82 (0.32, 2.11)	0.1 (-0.06, 0.26)
90 - <100	0.82 (0.08, 8.61)	0.36 (-0.15, 0.87)	1.16 (0.11, 12.41)	0.35 (-0.14, 0.85)	2.34 (0.19, 28.24)	0.38 (-0.21, 0.96)	-	-
100 - <110	-	-	-	-	-	-	-	-
**Length of Stay (months)**								
<1	0.76 (0.72, 0.81)	0.34 (0.27, 0.41)	0.72 (0.68, 0.76)	0.25 (0.24, 0.26)	0.69 (0.65, 0.74)	0.17 (0.04, 0.3)	0.84 (0.77, 0.92)	0.09 (-0.04, 0.22)
1-<6 (comparator)	1.0	0.4 (0.32, 0.48)	1.0	0.32 (0.31, 0.32)	1.0	0.22 (0.05, 0.39)	1.0	0.11 (-0.04, 0.25)
6-<12	1.14 (1.06, 1.22)	0.42 (0.34, 0.51)	1.18 (1.1, 1.27)	0.35 (0.34, 0.36)	1.26 (1.16, 1.36)	0.26 (0.06, 0.46)	1.19 (1.07, 1.31)	0.12 (-0.05, 0.29)
12-<24	1.65 (1.5, 1.8)	0.51 (0.41, 0.61)	1.78 (1.63, 1.95)	0.44 (0.42, 0.46)	1.75 (1.57, 1.95)	0.32 (0.07, 0.57)	2.02 (1.78, 2.29)	0.19 (-0.07, 0.45)
24+	1.68 (1.43, 1.98)	0.51 (0.41, 0.62)	1.81 (1.53, 2.14)	0.44 (0.41, 0.48)	1.86 (1.56, 2.21)	0.33 (0.07, 0.59)	2.13 (1.68, 2.7)	0.2 (-0.08, 0.47)
**Ethnic Group**								
White (comparator)	1.0	0.4 (0.32, 0.48)	1.0	0.32 (0.32, 0.33)	1.0	0.23 (0.05, 0.4)	1.0	0.12 (-0.04, 0.28)
Mixed	0.87 (0.74, 1.02)	0.37 (0.29, 0.45)	0.83 (0.7, 0.98)	0.28 (0.25, 0.32)	0.79 (0.65, 0.96)	0.19 (0.04, 0.34)	0.85 (0.66, 1.1)	0.1 (-0.04, 0.24)
Asian or Asian British	0.86 (0.77, 0.97)	0.37 (0.29, 0.45)	0.82 (0.73, 0.93)	0.28 (0.26, 0.31)	0.84 (0.73, 0.97)	0.2 (0.04, 0.36)	0.84 (0.7, 1.01)	0.1 (-0.04, 0.24)
Black or Black British	0.76 (0.65, 0.9)	0.34 (0.27, 0.42)	0.77 (0.65, 0.92)	0.27 (0.24, 0.3)	0.73 (0.6, 0.89)	0.18 (0.04, 0.33)	0.6 (0.45, 0.8)	0.07 (-0.03, 0.18)
Chinese and Other	0.72 (0.57, 0.92)	0.33 (0.25, 0.41)	0.74 (0.57, 0.95)	0.26 (0.22, 0.31)	0.66 (0.48, 0.9)	0.17 (0.03, 0.3)	0.55 (0.34, 0.86)	0.07 (-0.03, 0.17)
Unclassified	0.56 (0.47, 0.66)	0.28 (0.22, 0.35)	0.55 (0.45, 0.66)	0.21 (0.18, 0.24)	0.45 (0.36, 0.57)	0.12 (0.02, 0.22)	0.42 (0.3, 0.58)	0.05 (-0.02, 0.13)

Indicator *The proportion of the prison population who have received one, two, three or four (course of three plus booster) Hepatitis B vaccinations in the preceding 12 months*

Group: Communicable Disease

Univariate

Patients resident in the study prison in the last 12 months with a clinical code added at any time indicating a history of any illicit drug use
Odds Ratio (OR) of achieving indicator (95% CI)

Variable	1st Hep B		1st and 2nd Hep B		1st, 2nd and 3rd Hep B		1st, 2nd, 3rd and 4th Hep B	
**Year**								
2017/18 (comparator)	1.0	-	1.0	-	1.0	-	1.0	-
2018/19	2.03	(1.89, 2.17)	1.33	(1.24, 1.43)	1.36	(1.26, 1.46)	1.36	(1.21, 1.52)
2019/20	3.09	(2.9, 3.3)	2.13	(1.99, 2.27)	2.12	(1.97, 2.27)	2.19	(1.98, 2.42)
**Prison**								
Prison 1	1.52	(1.36, 1.69)	1.67	(1.49, 1.87)	1.97	(1.75, 2.23)	2.02	(1.7, 2.41)
Prison 2	0.85	(0.77, 0.95)	0.85	(0.77, 0.95)	0.88	(0.78, 0.99)	0.71	(0.58, 0.87)
Prison 3	1.76	(1.59, 1.94)	1.87	(1.69, 2.08)	2.09	(1.87, 2.33)	2.98	(2.56, 3.46)
Prison 4	2.04	(1.84, 2.26)	2.15	(1.94, 2.39)	2.51	(2.25, 2.81)	2.79	(2.39, 3.26)
Prison 5	1.86	(1.38, 2.51)	2.14	(1.59, 2.89)	2.47	(1.81, 3.37)	2.07	(1.32, 3.22)
Prison 6	1.90	(1.68, 2.15)	1.96	(1.73, 2.22)	2.37	(2.08, 2.71)	1.90	(1.56, 2.32)
Prison 7	4.61	(4, 5.33)	4.57	(3.97, 5.25)	4.88	(4.23, 5.63)	4.46	(3.73, 5.35)
Prison 8	2.30	(2.04, 2.6)	2.03	(1.79, 2.3)	2.04	(1.78, 2.33)	1.96	(1.62, 2.38)
Prison 9	2.20	(1.72, 2.82)	2.03	(1.58, 2.61)	2.25	(1.73, 2.92)	3.72	(2.73, 5.06)
Prison 10	1.66	(1.52, 1.8)	1.50	(1.37, 1.64)	1.50	(1.35, 1.66)	1.46	(1.26, 1.7)
Prison 11 (comparator)	1.0	-	1.0	-	1.0	-	1.0	-
Prison 12	2.61	(2.24, 3.04)	3.03	(2.6, 3.52)	3.73	(3.19, 4.37)	1.70	(1.32, 2.19)
Prison 13	1.39	(1.09, 1.77)	1.42	(1.11, 1.82)	1.64	(1.26, 2.14)	1.56	(1.06, 2.3)
**Prison category**								
A (comparator)	1.0	-	1.0	-	1.0	-	1.0	-
B	0.67	(0.57, 0.8)	0.67	(0.56, 0.79)	0.59	(0.49, 0.71)	0.43	(0.34, 0.55)
C	1.02	(0.86, 1.22)	1.12	(0.94, 1.34)	1.14	(0.95, 1.38)	1.05	(0.83, 1.32)
Closed	1.76	(1.47, 2.12)	1.69	(1.4, 2.04)	1.56	(1.29, 1.9)	1.16	(0.91, 1.48)
D	1.09	(0.9, 1.33)	1.17	(0.96, 1.43)	1.25	(1.01, 1.53)	0.76	(0.58, 1)
YOI	1.50	(1.21, 1.86)	1.79	(1.44, 2.22)	1.95	(1.56, 2.44)	0.68	(0.49, 0.93)
**Gender**								
Female (comparator)	1.0	-	1.0	-	1.0	-	1.0	-
Male	0.54	(0.49, 0.59)	0.6	(0.54, 0.65)	0.6	(0.56, 0.68)	0.7	(0.57, 0.74)
**Age - years**								
10 - <20	0.99	(0.83, 1.19)	1.0	(0.81, 1.17)	1.1	(0.88, 1.29)	0.4	(0.26, 0.58)
20 - <30	1.00	(0.94, 1.06)	1.0	(0.98, 1.1)	1.0	(0.96, 1.1)	0.9	(0.84, 1.01)
30 - <40 (comparator)	1.0	-	1.0	-	1.0	-	1.0	-
40 - <50	0.93	(0.86, 0.99)	0.9	(0.85, 0.98)	0.9	(0.84, 0.99)	0.9	(0.79, 0.98)
50 - <60	0.91	(0.81, 1.02)	0.9	(0.82, 1.04)	1.0	(0.85, 1.1)	0.8	(0.68, 0.99)
60 - <70	0.54	(0.4, 0.74)	0.6	(0.4, 0.77)	0.5	(0.37, 0.78)	0.6	(0.32, 0.95)
70 - <80	0.99	(0.43, 2.25)	1.0	(0.44, 2.32)	0.9	(0.38, 2.35)	1.1	(0.33, 3.76)
80 - <90	0.23	(0.03, 1.83)	0.3	(0.03, 2.16)	0.4	(0.05, 3)	1.0	(0.13, 8.31)
90 - <100	-	-	-	-	-	-	-	-
100 - <110	-	-	-	-	-	-	-	-
**Length of Stay (months)**								
<1	0.70	(0.66, 0.74)	0.6	(0.6, 0.69)	0.6	(0.55, 0.63)	0.7	(0.61, 0.75)
1-<6 (comparator)	1.0	-	1.0	-	1.0	-	1.0	-
6-<12	1.21	(1.12, 1.3)	1.3	(1.16, 1.35)	1.4	(1.26, 1.48)	1.3	(1.2, 1.49)
12-<24	1.90	(1.71, 2.1)	2.0	(1.84, 2.26)	2.3	(2.03, 2.52)	2.5	(2.19, 2.92)
24+	1.83	(1.46, 2.29)	1.8	(1.46, 2.29)	2.1	(1.66, 2.64)	2.8	(2.1, 3.83)
**Ethnic Group**								
White (comparator)	1.0	-	1.0	-	1.0	-	1.0	-
Mixed	0.94	(0.76, 1.17)	1.0	(0.8, 1.24)	0.9	(0.74, 1.19)	1.1	(0.8, 1.46)
Asian or Asian British	1.13	(0.95, 1.34)	1.2	(0.98, 1.39)	1.2	(0.97, 1.39)	1.1	(0.84, 1.36)
Black or Black British	0.76	(0.6, 0.95)	0.8	(0.61, 0.99)	0.9	(0.68, 1.12)	0.5	(0.36, 0.81)
Chinese and Other	0.77	(0.53, 1.13)	0.8	(0.56, 1.22)	0.9	(0.57, 1.29)	0.8	(0.47, 1.49)
Unclassified	0.62	(0.5, 0.78)	0.7	(0.55, 0.87)	0.7	(0.52, 0.86)	0.5	(0.35, 0.77)

Indicator	The proportion of the prison population who have received one, two, three or four (course of three plus booster) Hepatitis B vaccinations in the preceding 12 months							
Domain: Communicable Disease								
	Multivariable							
	Patients resident in the study prison in the last 12 months with a clinical code added at any time indicating a history of any illicit drug use							
Variable	1st Hep B		1st and 2nd Hep B		1st, 2nd and 3rd Hep B		1st, 2nd, 3rd and 4th Hep B	
	OR (95% CI)	Probability of achieving indicator (95% CI)	OR (95% CI)	Probability of achieving indicator (95% CI)	OR (95% CI)	Probability of achieving indicator (95% CI)	OR (95% CI)	Probability of achieving indicator (95% CI)
**Year**								
2017/18 (comparator)	1.0	0.27 (0.02, 0.52)	1.0	0.27 (0.09, 0.44)	1.0	0.2 (0.03, 0.38)	1.0	0.08 (0.07, 0.09)
2018/19	2.25 (2.08, 2.44)	0.44 (0.03, 0.86)	1.42 (1.31, 1.54)	0.34 (0.12, 0.56)	1.46 (1.34, 1.6)	0.27 (0.04, 0.5)	1.46 (1.29, 1.66)	0.11 (0.1, 0.12)
2019/20	3.64 (3.37, 3.93)	0.56 (0.04, 1.07)	2.5 (2.31, 2.7)	0.46 (0.16, 0.77)	2.62 (2.41, 2.85)	0.39 (0.06, 0.72)	2.89 (2.57, 3.25)	0.19 (0.18, 0.2)
**Prison**								
Prison 1	1.79 (1.55, 2.06)	0.49 (0.03, 0.94)	1.81 (1.57, 2.09)	0.43 (0.14, 0.71)	2.04 (1.76, 2.36)	0.38 (0.05, 0.71)	2.1 (1.72, 2.55)	0.19 (0.12, 0.26)
Prison 2	0.64 (0.57, 0.71)	0.27 (0.02, 0.52)	0.62 (0.55, 0.7)	0.21 (0.07, 0.35)	0.64 (0.56, 0.73)	0.17 (0.02, 0.31)	0.52 (0.42, 0.64)	0.06 (0.02, 0.09)
Prison 3	1.48 (1.31, 1.66)	0.44 (0.03, 0.86)	1.51 (1.34, 1.7)	0.38 (0.13, 0.64)	1.63 (1.43, 1.85)	0.33 (0.05, 0.62)	2.34 (1.97, 2.76)	0.21 (0.13, 0.28)
Prison 4	1.37 (1.21, 1.55)	0.43 (0.03, 0.83)	1.41 (1.24, 1.59)	0.37 (0.13, 0.61)	1.62 (1.42, 1.85)	0.33 (0.05, 0.61)	2.05 (1.72, 2.45)	0.19 (0.12, 0.26)
Prison 5	1.27 (0.88, 1.84)	0.41 (0.02, 0.8)	1.38 (0.95, 1.99)	0.36 (0.11, 0.61)	1.74 (1.19, 2.54)	0.35 (0.04, 0.65)	1.77 (1.07, 2.96)	0.17 (0.07, 0.26)
Prison 6	2.13 (1.82, 2.49)	0.53 (0.03, 1.02)	2.03 (1.74, 2.38)	0.45 (0.15, 0.75)	2.39 (2.03, 2.81)	0.42 (0.06, 0.77)	1.79 (1.44, 2.24)	0.17 (0.1, 0.24)
Prison 7	6.99 (0.72, 68.36)	0.77 (-0.02, 1.56)	7.86 (0.79, 78.66)	0.75 (0.14, 1.35)	3.11 (0.38, 25.12)	0.48 (-0.13, 1.08)	1.32 (0.05, 37.59)	0.13 (-0.18, 0.44)
Prison 8	3.93 (0.4, 38.46)	0.66 (-0.08, 1.41)	3.81 (0.38, 38.22)	0.6 (-0.01, 1.21)	1.3 (0.16, 10.57)	0.29 (-0.16, 0.73)	0.52 (0.02, 14.9)	0.06 (-0.09, 0.2)
Prison 9	1.52 (1.15, 2)	0.45 (0.02, 0.87)	1.34 (1.02, 1.77)	0.36 (0.12, 0.6)	1.4 (1.05, 1.87)	0.3 (0.04, 0.56)	2.34 (1.68, 3.26)	0.21 (0.12, 0.29)
Prison 10	1.37 (1.24, 1.51)	0.42 (0.03, 0.82)	1.2 (1.09, 1.34)	0.33 (0.11, 0.55)	1.19 (1.07, 1.34)	0.27 (0.04, 0.5)	1.23 (1.05, 1.45)	0.12 (0.07, 0.18)
Prison 11 (comparator)	1.0	0.36 (0.02, 0.69)	1.0	0.3 (0.1, 0.49)	1.0	0.24 (0.03, 0.44)	1.0	0.1 (0.05, 0.16)
Prison 12	1.92 (1.57, 2.34)	0.5 (0.03, 0.97)	2.11 (1.73, 2.57)	0.46 (0.16, 0.77)	2.42 (1.97, 2.98)	0.42 (0.06, 0.78)	1.6 (1.19, 2.15)	0.15 (0.08, 0.22)
Prison 13	1.5 (1.11, 2.02)	0.45 (0.02, 0.87)	1.39 (1.03, 1.87)	0.37 (0.12, 0.61)	1.52 (1.12, 2.06)	0.32 (0.04, 0.59)	1.38 (0.92, 2.1)	0.14 (0.06, 0.21)
**Prison category**								
A (comparator)	-		-		-		-	
B	-		-		-		-	
C	-		-		-		-	
Closed	-		-		-		-	
D	-		-		-		-	
YOI	-		-		-		-	
**Gender**								
Female (comparator)	1.0	0.28 (-0.17, 0.73)	1.0	0.21 (-0.12, 0.54)	1.0	0.31 (-0.14, 0.77)	1.0	0.26 (-0.28, 0.81)
Male	2.26 (0.23, 22.07)	0.44 (0.03, 0.86)	2.51 (0.25, 25.14)	0.38 (0.13, 0.63)	0.87 (0.11, 7.02)	0.29 (0.04, 0.53)	0.35 (0.01, 10)	0.12 (0.1, 0.14)
**Age - years**								
10 - <20	0.6 (0.47, 0.76)	0.33 (0.02, 0.63)	0.54 (0.42, 0.7)	0.25 (0.08, 0.41)	0.62 (0.47, 0.8)	0.21 (0.03, 0.39)	0.37 (0.23, 0.6)	0.06 (0.03, 0.08)
20 - <30	1.01 (0.94, 1.09)	0.44 (0.03, 0.85)	1.06 (0.98, 1.13)	0.38 (0.13, 0.63)	1.06 (0.98, 1.15)	0.31 (0.05, 0.57)	1.04 (0.94, 1.15)	0.14 (0.13, 0.15)
30 - <40 (comparator)	1.0	0.44 (0.03, 0.85)	1.0	0.37 (0.13, 0.61)	1.0	0.29 (0.05, 0.54)	1.0	0.13 (0.13, 0.14)
40 - <50	0.89 (0.82, 0.96)	0.41 (0.03, 0.79)	0.88 (0.81, 0.95)	0.34 (0.12, 0.56)	0.88 (0.8, 0.96)	0.27 (0.04, 0.5)	0.84 (0.75, 0.95)	0.12 (0.11, 0.13)
50 - <60	0.86 (0.76, 0.99)	0.41 (0.03, 0.78)	0.88 (0.77, 1.01)	0.34 (0.12, 0.56)	0.95 (0.83, 1.1)	0.29 (0.04, 0.53)	0.86 (0.71, 1.06)	0.12 (0.1, 0.14)
60 - <70	0.55 (0.39, 0.78)	0.31 (0.01, 0.61)	0.57 (0.4, 0.81)	0.25 (0.08, 0.43)	0.56 (0.37, 0.83)	0.19 (0.02, 0.37)	0.62 (0.36, 1.09)	0.09 (0.05, 0.13)
70 - <80	0.78 (0.33, 1.82)	0.38 (-0.02, 0.78)	0.79 (0.33, 1.86)	0.32 (0.05, 0.59)	0.71 (0.28, 1.81)	0.23 (-0.02, 0.48)	0.8 (0.23, 2.72)	0.11 (0, 0.22)
80 - <90	0.21 (0.03, 1.73)	0.15 (-0.15, 0.45)	0.25 (0.03, 2.04)	0.13 (-0.12, 0.39)	0.34 (0.04, 2.79)	0.13 (-0.12, 0.39)	0.91 (0.11, 7.63)	0.12 (-0.1, 0.34)
90 - <100	-	-	-	-	-	-	-	-
100 - <110	-	-	-	-	-	-	-	-
**Length of Stay (months)**								
<1	0.83 (0.77, 0.89)	0.38 (0.03, 0.74)	0.8 (0.74, 0.86)	0.31 (0.11, 0.51)	0.76 (0.7, 0.83)	0.24 (0.04, 0.44)	0.84 (0.75, 0.94)	0.11 (0.1, 0.11)
1-<6 (comparator)	1.0	0.43 (0.03, 0.82)	1.0	0.36 (0.13, 0.59)	1.0	0.29 (0.04, 0.53)	1.0	0.12 (0.12, 0.13)
6-<12	1.12 (1.03, 1.23)	0.45 (0.03, 0.88)	1.15 (1.05, 1.26)	0.39 (0.14, 0.64)	1.21 (1.1, 1.33)	0.32 (0.05, 0.6)	1.19 (1.05, 1.35)	0.14 (0.13, 0.15)
12-<24	1.53 (1.36, 1.73)	0.52 (0.03, 1.01)	1.63 (1.44, 1.83)	0.47 (0.16, 0.77)	1.73 (1.53, 1.95)	0.4 (0.06, 0.74)	1.92 (1.64, 2.24)	0.2 (0.18, 0.23)
24+	1.46 (1.15, 1.85)	0.51 (0.03, 0.99)	1.44 (1.13, 1.82)	0.44 (0.15, 0.73)	1.61 (1.25, 2.06)	0.39 (0.06, 0.72)	1.87 (1.37, 2.56)	0.2 (0.16, 0.25)
**Ethnic Group**								
White (comparator)	1.0	0.43 (0.03, 0.83)	1.0	0.36 (0.13, 0.6)	1.0	0.29 (0.05, 0.54)	1.0	0.13 (0.13, 0.13)
Mixed	0.88 (0.7, 1.11)	0.4 (0.02, 0.78)	0.94 (0.75, 1.18)	0.35 (0.12, 0.58)	0.86 (0.68, 1.1)	0.27 (0.04, 0.5)	1.07 (0.78, 1.46)	0.14 (0.1, 0.17)
Asian or Asian British	1 (0.83, 1.19)	0.43 (0.03, 0.83)	1.04 (0.87, 1.25)	0.37 (0.13, 0.62)	1.02 (0.85, 1.23)	0.3 (0.04, 0.55)	1.06 (0.83, 1.37)	0.14 (0.11, 0.16)
Black or Black British	0.75 (0.59, 0.96)	0.37 (0.02, 0.72)	0.77 (0.6, 0.99)	0.31 (0.1, 0.52)	0.84 (0.65, 1.1)	0.26 (0.04, 0.49)	0.59 (0.38, 0.9)	0.08 (0.05, 0.11)
Chinese and Other	0.85 (0.57, 1.27)	0.4 (0.02, 0.78)	0.91 (0.61, 1.37)	0.34 (0.1, 0.58)	0.94 (0.61, 1.44)	0.28 (0.03, 0.53)	0.99 (0.55, 1.8)	0.13 (0.07, 0.19)
Unclassified	0.55 (0.44, 0.7)	0.31 (0.02, 0.6)	0.6 (0.48, 0.76)	0.26 (0.09, 0.44)	0.57 (0.44, 0.74)	0.2 (0.03, 0.37)	0.46 (0.31, 0.69)	0.07 (0.04, 0.09)

Indicator	*The percentage of patients with coronary heart disease, stroke or transient ischemic attack, diabetes and/or chronic obstructive pulmonary disease who have influenza immunisation in the preceding 1 August and 31 March*						
	**Group: Communicable Disease**						
		**Univariate**		**Multivariable**			
	**Variable**	**Odds Ratio (OR) of achieving indicator**	**(95% CI) of OR**	**OR**	**(95% CI)**	**Probability of achieving indicator**	**(95% CI) of probability**
**Year**							
	2017/18 (comparator)	1.0	-	1.0	-	0.44	(-2.36, 3.24)
	2018/19	0.86	(0.74, 1)	0.86	(0.73, 1.03)	0.41	(-2.2, 3.02)
	2019/20	1.03	(0.89, 1.19)	1.22	(1.02, 1.45)	0.48	(-2.58, 3.55)
**Prison**							
	Prison 1	2.58	(1.81, 3.68)	1.18	(0.77, 1.82)	0.51	(-2.71, 3.72)
	Prison 2	1.96	(1.51, 2.54)	1.19	(0.87, 1.62)	0.51	(-2.71, 3.72)
	Prison 3	5.99	(4.59, 7.81)	2.36	(1.68, 3.31)	0.64	(-3.41, 4.68)
	Prison 4	4.19	(3.23, 5.43)	1.28	(0.92, 1.79)	0.52	(-2.79, 3.83)
	Prison 5	2.52	(1.69, 3.78)	0.92	(0.56, 1.51)	0.46	(-2.45, 3.37)
	Prison 6	3.62	(2.69, 4.88)	1.47	(1, 2.16)	0.55	(-2.93, 4.03)
	Prison 7	2.93	(2.11, 4.07)	-	-	0.05	(-0.28, 0.38)
	Prison 8	1.94	(1.46, 2.58)	-	-	0.04	(-0.24, 0.33)
	Prison 9	4.83	(3.74, 6.22)	1.04	(0.73, 1.49)	0.48	(-2.58, 3.54)
	Prison 10	1.53	(1.17, 2.01)	1.09	(0.79, 1.5)	0.49	(-2.62, 3.6)
	Prison 11 (comparator)	1.0	-	1.0	-	0.47	(-2.54, 3.48)
	Prison 12	0.67	(0.15, 3.05)	0.13	(0.02, 1.14)	0.20	(-1.09, 1.5)
	Prison 13	7.07	(5.14, 9.73)	1.70	(1.11, 2.6)	0.57	(-3.08, 4.23)
**Prison category**							
	A (comparator)	1.0	-				
	B	0.26	(0.22, 0.31)				
	C	0.82	(0.68, 0.99)				
	Closed	0.42	(0.34, 0.52)				
	D	0.60	(0.47, 0.76)				
	YOI	0.12	(0.03, 0.56)				
**Gender**							
	Female (comparator)	1.0	-	1.0	-	0.93	(-4.96, 6.82)
	Male	1.28	(1.05, 1.55)	-	-	0.40	(-2.14, 2.93)
**Age - years**							
	10 - <20	-	-	-	-	-	-
	20 - <30	0.69	(0.51, 0.95)	1.07	(0.73, 1.57)	0.46	(-2.45, 3.36)
	30 - <40	0.76	(0.62, 0.93)	0.95	(0.75, 1.21)	0.43	(-2.31, 3.17)
	40 - <50	0.83	(0.71, 0.97)	0.96	(0.79, 1.16)	0.43	(-2.32, 3.19)
	50 - <60 (comparator)	1.0	-	1.0	-	0.44	(-2.37, 3.25)
	60 - <70	1.35	(1.14, 1.61)	1.06	(0.87, 1.3)	0.45	(-2.44, 3.35)
	70 - <80	1.58	(1.24, 2.02)	1.14	(0.86, 1.52)	0.47	(-2.52, 3.46)
	80 - <90	1.84	(1.16, 2.92)	1.28	(0.76, 2.16)	0.49	(-2.65, 3.64)
	90 - <100	1.36	(0.19, 9.69)	0.78	(0.11, 5.72)	0.39	(-2.12, 2.9)
	100 - <110	-	-	-	-	-	-
**Length of Stay (months)**							
	<1	0.41	(0.32, 0.52)	0.46	(0.35, 0.6)	0.17	(-0.92, 1.26)
	1-<6 (comparator)	1.0	-	1.0	-	0.31	(-1.65, 2.27)
	6-<12	2.17	(1.8, 2.61)	2.16	(1.74, 2.69)	0.49	(-2.6, 3.58)
	12-<24	3.23	(2.66, 3.93)	2.89	(2.29, 3.65)	0.56	(-2.98, 4.09)
	24+	4.44	(3.75, 5.27)	3.96	(3.09, 5.07)	0.63	(-3.38, 4.64)
**Ethnic Group**							
	White (comparator)	1.0	-	1.0	-	0.45	(-2.4, 3.29)
	Mixed	1.44	(0.9, 2.31)	1.34	(0.8, 2.26)	0.51	(-2.74, 3.76)
	Asian or Asian British	0.88	(0.63, 1.23)	0.98	(0.68, 1.43)	0.44	(-2.38, 3.27)
	Black or Black British	0.55	(0.33, 0.92)	0.58	(0.34, 0.99)	0.34	(-1.8, 2.47)
	Chinese and Other	0.46	(0.21, 0.99)	0.40	(0.17, 0.92)	0.27	(-1.44, 1.98)
	Unclassified	1.62	(0.99, 2.64)	0.84	(0.48, 1.45)	0.41	(-2.2, 3.02)

Indicator		*The proportion of prison population prescribed opioids in the preceding 8 weeks from 31st March*									
	**Group: Prison specific**	**Univariate: Odds ratios (95% CI)**									
		**All prison population**						**Any mental health diagnosis**		**All prison population AND NO coded diagnosis of neuropathic pain**	
	**Variable**	**Prescribed any opioid in the preceding 8 weeks from 31st March**		**Prescribed any strong opioid in the preceding 8 weeks from 31st March**		**Prescribed benzodiazepines AND any opioid in preceding 8 weeks from 31st March**		**Prescribed any opioid in the preceding 8 weeks from 31st March**		**Prescribed pregabalin OR gabapentin in the preceding 8 weeks from 31st March**	
**Year**											
	2017/18 (comparator)	1.0	-	1.0	-	1.0	-	1.0	-	1.0	-
	2018/19	1.38	(1.29, 1.47)	1.25	(1.17, 1.34)	3.07	(2.9, 3.25)	1.40	(1.2, 1.63)	0.86	(0.71, 1.05)
	2019/20	1.40	(1.32, 1.49)	1.04	(0.97, 1.11)	1.04	(0.97, 1.11)	1.48	(1.29, 1.7)	0.80	(0.66, 0.96)
**Prison**											
	Prison 1	0.95	(0.84, 1.08)	1.02	(0.9, 1.16)	1.00	(0.88, 1.14)	1.19	(0.87, 1.62)	1.66	(1.16, 2.38)
	Prison 2	0.89	(0.8, 0.98)	0.83	(0.74, 0.92)	0.83	(0.74, 0.92)	1.26	(0.96, 1.65)	1.66	(1.24, 2.21)
	Prison 3	2.06	(1.89, 2.25)	2.10	(1.92, 2.3)	2.15	(1.96, 2.35)	3.55	(2.63, 4.78)	-	-
	Prison 4	2.16	(1.98, 2.36)	2.16	(1.98, 2.37)	2.18	(1.99, 2.39)	2.61	(1.87, 3.64)	1.41	(1.02, 1.95)
	Prison 5	0.70	(0.58, 0.85)	0.86	(0.71, 1.04)	0.82	(0.68, 0.99)	0.74	(0.43, 1.28)	2.04	(1.32, 3.15)
	Prison 6	-	-	-	-	-	-	-	-	-	-
	Prison 7	2.78	(2.46, 3.13)	2.99	(2.65, 3.39)	3.18	(2.8, 3.61)	1.80	(1.17, 2.76)	3.42	(2.38, 4.9)
	Prison 8	1.70	(1.52, 1.9)	1.69	(1.5, 1.9)	1.71	(1.52, 1.93)	1.67	(1.26, 2.2)	2.26	(1.61, 3.18)
	Prison 9	1.97	(1.76, 2.21)	2.40	(2.15, 2.7)	2.46	(2.18, 2.76)	2.32	(1.57, 3.42)	3.34	(2.41, 4.63)
	Prison 10	1.22	(1.11, 1.34)	0.60	(0.53, 0.67)	6.80	(6.28, 7.37)	1.18	(0.88, 1.59)	1.28	(0.95, 1.74)
	Prison 11 (comparator)	1.0	-	1.0	-	1.0	-	1.0	-	1.0	-
	Prison 12	-	-	-	-	-	-	-	-	-	-
	Prison 13	0.69	(0.57, 0.83)	0.94	(0.79, 1.12)	0.92	(0.77, 1.1)	0.60	(0.41, 0.9)	2.97	(2.02, 4.37)
**Prison category**											
	A (comparator)	1.0	-	1.0	-	1.0	-	1.0	-	1.0	-
	B	0.73	(0.66, 0.8)	0.47	(0.43, 0.52)	1.20	(1.1, 1.32)	1.15	(0.91, 1.44)	0.40	(0.32, 0.5)
	C	1.30	(1.19, 1.43)	1.06	(0.97, 1.16)	1.06	(0.97, 1.17)	2.04	(1.62, 2.58)	0.27	(0.21, 0.35)
	Closed	1.47	(1.32, 1.64)	1.21	(1.09, 1.35)	1.24	(1.11, 1.38)	1.60	(1.26, 2.03)	0.84	(0.63, 1.11)
	D	0.14	(0.11, 0.17)	0.14	(0.11, 0.17)	0.13	(0.11, 0.16)	0.70	(0.42, 1.2)	0.18	(0.12, 0.28)
	YOI	-	-	-	-	-	-	-	-	-	-
**Gender**											
	Female (comparator)	1.0	-	1.0	-	1.0	-	1.0	-	1.0	-
	Male	0.60	(0.56, 0.65)	0.55	(0.51, 0.6)	0.89	(0.81, 0.96)	0.85	(0.74, 0.97)	0.46	(0.36, 0.58)
**Age - years**											
	10 - <20	0.06	(0.03, 0.11)	0.03	(0.01, 0.07)	0.35	(0.27, 0.44)	0.17	(0.02, 1.28)	-	-
	20 - <30	0.38	(0.35, 0.4)	0.32	(0.3, 0.35)	0.49	(0.46, 0.53)	0.43	(0.36, 0.52)	0.23	(0.17, 0.32)
	30 - <40 (comparator)	1.0	-	1.0	-	1.0	-	1.0	-	1.0	-
	40 - <50	1.37	(1.29, 1.45)	1.38	(1.3, 1.47)	1.27	(1.2, 1.35)	1.42	(1.25, 1.62)	1.75	(1.44, 2.12)
	50 - <60	1.02	(0.94, 1.11)	1.24	(1.14, 1.35)	1.16	(1.07, 1.25)	1.45	(1.21, 1.73)	1.96	(1.55, 2.48)
	60 - <70	0.91	(0.79, 1.05)	1.13	(0.99, 1.3)	1.06	(0.93, 1.2)	0.82	(0.58, 1.16)	1.92	(1.33, 2.77)
	70 - <80	1.19	(0.95, 1.49)	1.55	(1.25, 1.93)	1.34	(1.09, 1.65)	0.89	(0.45, 1.77)	0.89	(0.37, 2.18)
	80 - <90	1.35	(0.85, 2.13)	1.50	(0.94, 2.4)	1.53	(1, 2.35)	1.65	(0.33, 8.23)	0.84	(0.12, 6.02)
	90 - <100	-	-	-	-	-	-	-	-	17.23	(2.06, 143.89)
	100 - <110	-	-	-	-	-	-	-	-	-	-
**Length of Stay (months)**											
	<1	0.58	(0.54, 0.63)	0.75	(0.69, 0.81)	0.90	(0.85, 0.96)	0.67	(0.56, 0.81)	0.58	(0.44, 0.77)
	1-<6 (comparator)	1.0	-	1.0	-	1.0	-	1.0	-	1.0	-
	6-<12	1.40	(1.31, 1.5)	1.39	(1.28, 1.5)	1.03	(0.96, 1.1)	1.58	(1.36, 1.83)	1.43	(1.14, 1.78)
	12-<24	1.48	(1.38, 1.59)	1.80	(1.67, 1.94)	1.03	(0.96, 1.1)	1.89	(1.61, 2.23)	1.52	(1.2, 1.93)
	24+	1.94	(1.8, 2.1)	2.82	(2.61, 3.04)	1.54	(1.43, 1.65)	1.57	(1.32, 1.87)	2.29	(1.81, 2.89)
**Ethnic Group**											
	White (comparator)	1.0	-	1.0	-	1.0	-	1.0	-	1.0	-
	Mixed	0.42	(0.33, 0.53)	0.40	(0.31, 0.52)	0.49	(0.4, 0.6)	0.67	(0.42, 1.08)	0.30	(0.11, 0.81)
	Asian or Asian British	0.24	(0.19, 0.3)	0.24	(0.19, 0.31)	0.54	(0.46, 0.62)	0.25	(0.13, 0.47)	0.15	(0.06, 0.41)
	Black or Black British	0.33	(0.25, 0.43)	0.30	(0.23, 0.41)	0.39	(0.31, 0.48)	0.63	(0.37, 1.06)	0.22	(0.07, 0.69)
	Chinese and Other	0.29	(0.19, 0.45)	0.32	(0.21, 0.49)	0.50	(0.37, 0.68)	0.45	(0.19, 1.04)	0.66	(0.24, 1.76)
	Unclassified	0.51	(0.41, 0.64)	0.41	(0.31, 0.52)	0.43	(0.35, 0.54)	0.55	(0.3, 1)	0.40	(0.16, 0.96)

Indicator	*The proportion of prison population prescribed opioids in the preceding 8 weeks from 31st March*									
**Domain: Prison specific**	**Multivariable**									
	**All prison population**						**Any mental health diagnosis**		**All prison population AND NO coded diagnosis of neuropathic pain**	
**Variable**	**Prescribed any opioid in the preceding 8 weeks from 31st March**		**Prescribed any strong opioid in the preceding 8 weeks from 31st March**		**Prescribed benzodiazepines AND any opioid in preceding 8 weeks from 31st March**		**Prescribed any opioid in the preceding 8 weeks from 31st March**		**Prescribed pregabalin OR gabapentin in the preceding 8 weeks from 31st March**	
	**OR (95% CI)**	**Probability of achieving indicator (95% CI)**	**OR (95% CI)**	**Probability of achieving indicator (95% CI)**	**OR (95% CI)**	**Probability of achieving indicator (95% CI)**	**OR (95% CI)**	**Probability of achieving indicator (95% CI)**	**OR (95% CI)**	**Probability of achieving indicator (95% CI)**
**Year**										
2017/18 (comparator)	1.0	0.1 (0.07, 0.13)	1.0	0.1 (0.05, 0.16)	1.0	0.1 (0, 0.2)	1.0	0.14 (-0.13, 0.41)	1.0	0.01 (-0.05, 0.08)
2018/19	1.44 (1.35, 1.55)	0.14 (0.1, 0.18)	1.36 (1.27, 1.46)	0.13 (0.06, 0.2)	3.65 (3.42, 3.9)	0.27 (0.01, 0.53)	1.4 (1.18, 1.65)	0.18 (-0.17, 0.53)	0.85 (0.69, 1.06)	0.01 (-0.05, 0.07)
2019/20	1.47 (1.38, 1.58)	0.14 (0.1, 0.18)	1.22 (1.14, 1.32)	0.12 (0.06, 0.19)	1.18 (1.1, 1.27)	0.12 (0.01, 0.23)	1.64 (1.41, 1.91)	0.2 (-0.2, 0.6)	0.89 (0.71, 1.1)	0.01 (-0.05, 0.07)
**Prison**										
Prison 1	0.58 (0.49, 0.68)	0.09 (0.06, 0.12)	0.64 (0.55, 0.76)	0.1 (0.05, 0.16)	0.7 (0.59, 0.82)	0.11 (0, 0.21)	0.76 (0.54, 1.09)	0.13 (-0.13, 0.39)	1.22 (0.79, 1.9)	0.02 (-0.06, 0.09)
Prison 2	0.56 (0.5, 0.63)	0.09 (0.06, 0.12)	0.5 (0.44, 0.56)	0.08 (0.04, 0.13)	0.51 (0.46, 0.58)	0.08 (0, 0.16)	0.89 (0.66, 1.2)	0.15 (-0.15, 0.44)	1.2 (0.86, 1.67)	0.02 (-0.06, 0.09)
Prison 3	1.31 (1.18, 1.45)	0.18 (0.13, 0.24)	1.36 (1.23, 1.52)	0.19 (0.09, 0.29)	1.5 (1.35, 1.67)	0.19 (0.01, 0.38)	1.9 (1.36, 2.65)	0.26 (-0.26, 0.78)	-	-
Prison 4	1.02 (0.91, 1.13)	0.15 (0.1, 0.2)	1.09 (0.98, 1.22)	0.16 (0.07, 0.25)	1.2 (1.08, 1.34)	0.16 (0.01, 0.32)	1.64 (1.13, 2.38)	0.24 (-0.23, 0.7)	0.67 (0.46, 1)	0.01 (-0.03, 0.05)
Prison 5	0.28 (0.22, 0.36)	0.05 (0.03, 0.07)	0.46 (0.38, 0.57)	0.08 (0.03, 0.12)	0.49 (0.4, 0.61)	0.08 (0, 0.16)	0.39 (0.21, 0.71)	0.07 (-0.08, 0.22)	1.03 (0.62, 1.7)	0.01 (-0.05, 0.08)
Prison 6	-	-	-	-	-	-	-	-	-	-
Prison 7	1.14 (0.38, 3.43)	0.17 (0.03, 0.3)	0.84 (0.27, 2.59)	0.13 (0, 0.25)	0.79 (0.28, 2.24)	0.12 (-0.02, 0.26)	3 (0.28, 31.97)	0.35 (-0.45, 1.14)	1.29 (0.06, 29.76)	0.02 (-0.07, 0.11)
Prison 8	0.76 (0.25, 2.28)	0.12 (0.01, 0.22)	0.52 (0.17, 1.58)	0.08 (0, 0.17)	0.59 (0.21, 1.67)	0.09 (-0.02, 0.2)	2.48 (0.23, 27.15)	0.31 (-0.41, 1.03)	0.83 (0.04, 19.22)	0.01 (-0.05, 0.07)
Prison 9	0.75 (0.65, 0.87)	0.12 (0.08, 0.15)	0.84 (0.73, 0.97)	0.13 (0.06, 0.2)	0.94 (0.81, 1.09)	0.13 (0, 0.26)	1 (0.64, 1.56)	0.16 (-0.16, 0.49)	1.22 (0.78, 1.9)	0.02 (-0.06, 0.09)
Prison 10	1.01 (0.91, 1.12)	0.15 (0.1, 0.2)	0.47 (0.41, 0.53)	0.08 (0.03, 0.12)	4.68 (4.28, 5.13)	0.39 (0.02, 0.77)	1.04 (0.75, 1.43)	0.17 (-0.17, 0.5)	1.15 (0.81, 1.64)	0.01 (-0.06, 0.09)
Prison 11 (comparator)	1.0	0.15 (0.1, 0.19)	1.0	0.15 (0.07, 0.23)	1.0	0.14 (0.01, 0.28)	1.0	0.16 (-0.16, 0.49)	1.0	0.01 (-0.05, 0.07)
Prison 12	-	-	-	-	-	-	-	-	-	-
Prison 13	0.26 (0.21, 0.33)	0.05 (0.03, 0.06)	0.36 (0.29, 0.45)	0.06 (0.03, 0.1)	0.39 (0.31, 0.48)	0.06 (0, 0.13)	0.27 (0.17, 0.42)	0.05 (-0.05, 0.15)	1.02 (0.58, 1.77)	0.01 (-0.05, 0.08)
**Gender**										
Female (comparator)	1.0	0.15 (0.02, 0.28)	1.0	0.18 (0.01, 0.35)	1.0	0.23 (-0.03, 0.48)	1.0	0.11 (-0.15, 0.37)	1.0	0.02 (-0.09, 0.12)
Male	0.8 (0.27, 2.41)	0.13 (0.09, 0.17)	0.55 (0.18, 1.69)	0.11 (0.05, 0.17)	0.6 (0.21, 1.67)	0.16 (0.01, 0.31)	2.28 (0.21, 24.64)	0.21 (-0.21, 0.63)	0.64 (0.03, 14.53)	0.01 (-0.05, 0.07)
**Age - years**										
10 - <20	0.14 (0.07, 0.27)	0.02 (0.01, 0.04)	0.06 (0.02, 0.18)	0.01 (0, 0.02)	0.46 (0.34, 0.61)	0.09 (0, 0.19)	0.24 (0.03, 1.89)	0.05 (-0.09, 0.2)	-	-
20 - <30	0.44 (0.41, 0.48)	0.07 (0.05, 0.09)	0.38 (0.34, 0.41)	0.06 (0.03, 0.08)	0.5 (0.47, 0.54)	0.1 (0, 0.2)	0.42 (0.35, 0.51)	0.09 (-0.09, 0.26)	0.25 (0.17, 0.36)	0.003 (-0.01, 0.02)
30 - <40 (comparator)	1.0	0.14 (0.1, 0.18)	1.0	0.13 (0.06, 0.2)	1.0	0.17 (0.01, 0.34)	1.0	0.18 (-0.18, 0.54)	1.0	0.01 (-0.05, 0.07)
40 - <50	1.38 (1.29, 1.48)	0.19 (0.13, 0.24)	1.39 (1.3, 1.49)	0.17 (0.08, 0.26)	1.32 (1.24, 1.42)	0.21 (0.01, 0.42)	1.4 (1.21, 1.61)	0.23 (-0.23, 0.69)	1.77 (1.43, 2.19)	0.02 (-0.08, 0.13)
50 - <60	1.03 (0.94, 1.14)	0.15 (0.1, 0.19)	1.22 (1.11, 1.34)	0.15 (0.07, 0.24)	1.19 (1.09, 1.31)	0.2 (0.01, 0.39)	1.35 (1.11, 1.63)	0.23 (-0.22, 0.67)	1.7 (1.31, 2.21)	0.02 (-0.08, 0.12)
60 - <70	0.83 (0.71, 0.97)	0.12 (0.08, 0.16)	0.92 (0.79, 1.07)	0.12 (0.06, 0.19)	0.96 (0.83, 1.11)	0.17 (0.01, 0.33)	0.74 (0.51, 1.08)	0.14 (-0.14, 0.42)	1.54 (1.02, 2.31)	0.02 (-0.07, 0.11)
70 - <80	0.85 (0.66, 1.08)	0.12 (0.08, 0.17)	1.06 (0.84, 1.33)	0.14 (0.06, 0.21)	1.07 (0.85, 1.34)	0.18 (0.01, 0.36)	0.47 (0.23, 0.95)	0.1 (-0.1, 0.29)	0.61 (0.22, 1.65)	0.01 (-0.03, 0.05)
80 - <90	0.97 (0.6, 1.57)	0.14 (0.07, 0.21)	1.11 (0.68, 1.82)	0.14 (0.05, 0.24)	1.12 (0.7, 1.78)	0.19 (0, 0.38)	0.61 (0.12, 3.12)	0.12 (-0.17, 0.41)	0.81 (0.11, 5.9)	0.01 (-0.04, 0.06)
90 - <100	-	-	-	-	-	-	-	-	12.22 (1.41, 105.56)	0.13 (-0.54, 0.8)
100 - <110	-	-	-	-	-	-	-	-	-	-
**Length of Stay (months)**										
<1	0.58 (0.53, 0.64)	0.07 (0.05, 0.09)	0.79 (0.72, 0.87)	0.08 (0.04, 0.12)	0.85 (0.78, 0.92)	0.13 (0.01, 0.25)	0.66 (0.54, 0.81)	0.1 (-0.1, 0.31)	0.51 (0.37, 0.7)	0.006 (-0.02, 0.03)
1-<6 (comparator)	1.0	0.11 (0.08, 0.14)	1.0	0.1 (0.05, 0.15)	1.0	0.14 (0.01, 0.28)	1.0	0.15 (-0.14, 0.43)	1.0	0.01 (-0.04, 0.06)
6-<12	1.59 (1.47, 1.72)	0.16 (0.12, 0.21)	1.44 (1.32, 1.57)	0.13 (0.06, 0.21)	1.36 (1.26, 1.47)	0.18 (0.01, 0.35)	1.71 (1.46, 2.02)	0.22 (-0.22, 0.66)	1.77 (1.38, 2.29)	0.02 (-0.07, 0.11)
12-<24	1.82 (1.67, 1.99)	0.18 (0.13, 0.24)	1.75 (1.59, 1.91)	0.16 (0.07, 0.24)	1.6 (1.47, 1.75)	0.2 (0.01, 0.39)	2.01 (1.67, 2.42)	0.25 (-0.24, 0.74)	1.91 (1.43, 2.54)	0.02 (-0.08, 0.12)
24+	1.85 (1.67, 2.05)	0.18 (0.13, 0.24)	1.92 (1.73, 2.13)	0.17 (0.08, 0.26)	1.82 (1.64, 2.02)	0.22 (0.01, 0.43)	1.95 (1.56, 2.43)	0.24 (-0.24, 0.72)	1.91 (1.36, 2.67)	0.02 (-0.08, 0.12)
**Ethnic Group**										
White (comparator)	1.0	0.14 (0.1, 0.18)	1.0	0.13 (0.06, 0.19)	1.0	0.17 (0.01, 0.33)	1.0	0.18 (-0.18, 0.54)	1.0	0.01 (-0.05, 0.08)
Mixed	0.55 (0.43, 0.71)	0.08 (0.05, 0.11)	0.51 (0.39, 0.67)	0.07 (0.03, 0.11)	0.62 (0.5, 0.78)	0.12 (0.003, 0.23)	0.58 (0.36, 0.95)	0.12 (-0.12, 0.36)	0.33 (0.12, 0.9)	0.005 (-0.02, 0.03)
Asian or Asian British	0.32 (0.25, 0.4)	0.05 (0.03, 0.07)	0.34 (0.26, 0.43)	0.05 (0.02, 0.08)	0.56 (0.48, 0.65)	0.11 (0.004, 0.21)	0.25 (0.13, 0.48)	0.06 (-0.06, 0.17)	0.18 (0.07, 0.48)	0.003 (-0.01, 0.02)
Black or Black British	0.41 (0.31, 0.54)	0.06 (0.04, 0.09)	0.37 (0.28, 0.5)	0.05 (0.02, 0.08)	0.47 (0.37, 0.6)	0.09 (0.002, 0.19)	0.58 (0.33, 1.03)	0.12 (-0.12, 0.36)	0.22 (0.07, 0.69)	0.003 (-0.01, 0.02)
Chinese and Other	0.31 (0.2, 0.48)	0.05 (0.02, 0.07)	0.33 (0.21, 0.51)	0.05 (0.02, 0.08)	0.48 (0.35, 0.66)	0.1 (0.001, 0.19)	0.48 (0.2, 1.12)	0.1 (-0.11, 0.31)	0.61 (0.23, 1.66)	0.009 (-0.03, 0.05)
Unclassified	0.69 (0.54, 0.87)	0.1 (0.06, 0.13)	0.51 (0.39, 0.67)	0.07 (0.03, 0.11)	0.59 (0.47, 0.74)	0.11 (0.003, 0.22)	0.82 (0.44, 1.54)	0.16 (-0.16, 0.48)	0.43 (0.17, 1.08)	0.006 (-0.02, 0.04)

Indicator		*The proportion of new receptions to prison who have been asked for consent to transfer medical records from GP to prison healthcare*					
	**Group: Prison specific**						
		**Univariate**		**Multivariable**			
	**Variable**	**Odds Ratio (OR) of achieving indicator**	**(95% CI) of OR**	**OR**	**(95% CI)**	**Probability of achieving indicator**	**(95% CI) of probability**
**Year**							
	2017/18 (comparator)	1.0	-	1.0	-	0.52	(-0.02, 1.07)
	2018/19	2.55	(2.43, 2.68)	5.04	(4.64, 5.47)	0.72	(-0.03, 1.47)
	2019/20	2.05	(1.96, 2.15)	4.28	(3.96, 4.62)	0.70	(-0.03, 1.44)
**Prison**							
	Prison 1	0.16	(0.14, 0.17)	0.12	(0.11, 0.14)	0.54	(-0.02, 1.11)
	Prison 2	0.01	(0.01, 0.02)	0.01	(0.01, 0.01)	0.11	(-0.01, 0.23)
	Prison 3	3.57	(3.18, 4.01)	2.09	(1.78, 2.44)	0.93	(-0.04, 1.91)
	Prison 4	2.34	(2.08, 2.63)	1.68	(1.43, 1.97)	0.92	(-0.04, 1.88)
	Prison 5	0.50	(0.44, 0.58)	0.17	(0.14, 0.21)	0.61	(-0.03, 1.25)
	Prison 6	0.99	(0.88, 1.12)	0.50	(0.42, 0.59)	0.79	(-0.03, 1.62)
	Prison 7	2.03	(1.71, 2.41)	0.20	(0.08, 0.51)	0.64	(-0.05, 1.34)
	Prison 8	2.20	(1.93, 2.52)	0.09	(0.03, 0.21)	0.47	(-0.05, 0.99)
	Prison 9	1.08	(0.89, 1.31)	0.55	(0.43, 0.71)	0.81	(-0.04, 1.65)
	Prison 10	0.34	(0.32, 0.36)	0.18	(0.16, 0.19)	0.61	(-0.03, 1.25)
	Prison 11 (comparator)	1.0	-	1.0	-	0.88	(-0.04, 1.79)
	Prison 12	2.93	(2.39, 3.58)	2.36	(1.78, 3.13)	0.94	(-0.04, 1.92)
	Prison 13	0.48	(0.4, 0.58)	0.25	(0.19, 0.32)	0.68	(-0.03, 1.39)
**Prison category**							
	A (comparator)	1.0	-				
	B	0.38	(0.33, 0.43)				
	C	1.43	(1.26, 1.64)				
	Closed	2.86	(2.44, 3.35)				
	D	1.05	(0.91, 1.22)				
	YOI	3.88	(3.08, 4.89)				
**Gender**							
	Female (comparator)	1.0	-	1.0	-	0.88	(-0.04, 1.8)
	Male	0.17	(0.15, 0.19)	0.1	(0.02, 0.14)	0.61	(-0.03, 1.25)
**Age - years**							
	10 - <20	2.10	(1.81, 2.45)	0.91	(0.72, 1.16)	0.64	(-0.03, 1.32)
	20 - <30	0.96	(0.92, 1.01)	0.96	(0.88, 1.03)	0.65	(-0.03, 1.33)
	30 - <40 (comparator)	1.0	-	1.0	-	0.65	(-0.03, 1.34)
	40 - <50	1.00	(0.95, 1.06)	1.03	(0.94, 1.13)	0.66	(-0.03, 1.35)
	50 - <60	0.88	(0.82, 0.95)	0.86	(0.76, 0.97)	0.64	(-0.03, 1.3)
	60 - <70	0.81	(0.71, 0.92)	0.72	(0.58, 0.89)	0.62	(-0.03, 1.26)
	70 - <80	1.07	(0.82, 1.4)	0.70	(0.46, 1.05)	0.61	(-0.03, 1.25)
	80 - <90	0.49	(0.3, 0.8)	0.62	(0.28, 1.36)	0.60	(-0.03, 1.23)
	90 - <100	0.22	(0.02, 2.55)	0.72	(0.01, 65.55)	0.62	(-0.23, 1.46)
	100 - <110	-	-	-	-	-	-
**Length of Stay (months)**							
	<1	0.71	(0.68, 0.75)	0.77	(0.71, 0.83)	0.62	(-0.03, 1.27)
	1-<6 (comparator)	1.0	-	1.0	-	0.65	(-0.03, 1.33)
	6-<12	1.41	(1.33, 1.5)	1.22	(1.11, 1.34)	0.67	(-0.03, 1.37)
	12-<24	1.97	(1.81, 2.14)	1.43	(1.25, 1.63)	0.69	(-0.03, 1.41)
	24+	2.96	(2.51, 3.5)	1.85	(1.47, 2.31)	0.72	(-0.03, 1.46)
**Ethnic Group**							
	White (comparator)	1.0	-	1.0	-	0.65	(-0.03, 1.34)
	Mixed	0.81	(0.7, 0.95)	0.80	(0.65, 0.99)	0.63	(-0.03, 1.29)
	Asian or Asian British	0.84	(0.75, 0.94)	0.80	(0.69, 0.92)	0.63	(-0.03, 1.29)
	Black or Black British	0.69	(0.59, 0.8)	0.75	(0.61, 0.93)	0.62	(-0.03, 1.27)
	Chinese and Other	0.59	(0.48, 0.73)	0.70	(0.52, 0.96)	0.61	(-0.03, 1.26)
	Unclassified	1.48	(1.24, 1.76)	0.71	(0.57, 0.87)	0.61	(-0.03, 1.26)

Indicator	*The proportion of new receptions to prison with a code for a second-stage health assessment in the past 12 months, plus clinical codes for in possession risk assessment and medicines reconciliation added in the week before the second-stage assessment*					
	**Group: Prison specific**					
			**Univariate: Odds Ratio (95 %CI)**			
**Variable**	**Both Medicines Reconciliation (MR) and In-Possession (IP) assessment within 7 days**		**Had MR during stay, but did not satisfy both within 7 days**		**Had IP during stay, but did not satisfy both within 7 days**	
**Year**						
2019/20 only						
**Prison**						
Prison 1	10,036.80	(1932.51, 52127.7)	1.58	(0.14, 17.48)	0.00	(0, 0)
Prison 2	290.38	(116.89, 721.33)	53.93	(7.49, 388.4)	0.00	(0, 0.01)
Prison 3	1.19	(0.45, 3.16)	4.51	(0.59, 34.41)	0.72	(0.274, 1.87)
Prison 4	-	-	-	-	-	-
Prison 5	1.33	(0.38, 4.66)	-	-	0.752	(0.215, 2.64)
Prison 6	108.06	(43.36, 269.31)	58.45	(8.04, 424.99)	0.0093	(0.00371, 0.0231)
Prison 7	-	-	3.90	(0.4, 37.67)	-	-
Prison 8	91.37	(36.67, 227.68)	173.38	(24.04, 1250.28)	0.01	(0, 0.03)
Prison 9	2.27	(0.8, 6.45)	11.30	(1.44, 88.81)	0.44090	(0.15499, 1.25426)
Prison 10	16,063.80	(3096.28, 83340.43)	0.99	(0.09, 10.92)	0.00	(0, 0)
Prison 11 (comparator)	1.0	-	1.0	-	1.0	-
Prison 12	0.50	(0.12, 2.09)	2.15	(0.19, 23.88)	2.02	(0.48, 8.53)
Prison 13	84.68	(33.82, 212.02)	11.79	(1.5, 92.72)	0.01	(0, 0.03)
**Prison category**						
A (comparator)	1.0	-	1.0	-	1.0	-
B	5.91	(4.81, 7.25)	1.84	(1.13, 3)	0.17	(0.14, 0.21)
C	0.43	(0.35, 0.52)	0.18	(0.09, 0.35)	2.32	(1.9, 2.84)
Closed	1.24	(0.98, 1.58)	7.26	(4.45, 11.83)	0.80	(0.63, 1.02)
D	1.70	(1.33, 2.16)	3.11	(1.84, 5.27)	0.59	(0.46, 0.75)
YOI	0.02	(0.01, 0.06)	0.19	(0.04, 0.8)	49.41	(15.64, 156.1)
**Gender**						
Female (comparator)	1.0	-	1.0	-	1.0	-
Male	0.99	(0.83, 1.18)	0.14	(0.11, 0.18)	1.01	(0.85, 1.2)
**Age - years**						
10 - <20	0.55	(0.36, 0.84)	1.01	(0.44, 2.35)	1.82	(1.19, 2.78)
20 - <30	1.02	(0.9, 1.16)	0.85	(0.63, 1.14)	0.98	(0.86, 1.11)
30 - <40 (comparator)	1.0	-	1.0	-	1.0	-
40 - <50	0.99	(0.86, 1.14)	1.16	(0.86, 1.57)	1.01	(0.88, 1.17)
50 - <60	1.01	(0.84, 1.23)	1.31	(0.89, 1.94)	0.99	(0.82, 1.2)
60 - <70	0.59	(0.42, 0.82)	0.83	(0.4, 1.72)	1.71	(1.22, 2.38)
70 - <80	0.65	(0.35, 1.22)	1.69	(0.6, 4.78)	1.54	(0.82, 2.89)
80 - <90	2.38	(0.67, 8.44)	-	-	0.42	(0.12, 1.5)
90 - <100	-	-	-	-	-	-
100 - <110	-	-	-	-	-	-
**Length of Stay (months)**						
<1	1.17	(1, 1.38)	0.65	(0.45, 0.94)	0.85	(0.73, 1.01)
1-<6 (comparator)	1.0	-	1.0	-	1.0	-
6-<12	0.99	(0.86, 1.13)	0.40	(0.29, 0.57)	1.01	(0.89, 1.16)
12-<24	0.65	(0.56, 0.75)	0.54	(0.39, 0.75)	1.54	(1.33, 1.79)
24+	0.48	(0.39, 0.6)	0.81	(0.54, 1.22)	2.09	(1.67, 2.6)
**Ethnic Group**						
White (comparator)	1.0	-	1.0	-	1.0	-
Mixed	1.68	(1.2, 2.36)	1.73	(0.96, 3.1)	0.58	(0.41, 0.81)
Asian or Asian British	2.06	(1.59, 2.68)	0.72	(0.38, 1.38)	0.49	(0.37, 0.63)
Black or Black British	1.20	(0.86, 1.66)	0.58	(0.23, 1.42)	0.84	(0.6, 1.16)
Chinese and Other	3.63	(2.11, 6.23)	0.55	(0.13, 2.26)	0.26	(0.15, 0.44)
Unclassified	1.57	(0.99, 2.48)	0.71	(0.22, 2.26)	0.64	(0.4, 1.01)

Indicator		*The proportion of new receptions to prison with a code for a second-stage health assessment in the past 12 months, plus clinical codes for in possession risk assessment and medicines reconciliation added in the week before the second-stage assessment*					
	**Domain: Prison specific**						
		**Multivariable**					
	**Variable**	**Both Medicines Reconciliation (MR) and In-Possession (IP) assessment within 7 days**		**Had MR during stay, but did not satisfy both within 7 days**		**Had IP during stay, but did not satisfy both within 7 days**	
		**OR (95% CI)**	**Probability of achieving indicator (95% CI)**	**OR (95% CI)**	**Probability of achieving indicator (95% CI)**	**OR (95% CI)**	**Probability of achieving indicator (95% CI)**
**Year**							
	2019/20 only						
**Prison**							
	Prison 1	9724.53 (35.71, 2648315.74)	0.99 (0.99, 1)	1.54 (0.14, 17.11)	0.005 (-0.004, 0.013)	0.0001 (0.0000004, 0.03)	0.005 (-0.003, 0.01)
	Prison 2	267.54 (1.17, 61384.49)	0.84 (0.74, 0.95)	52.86 (7.3, 382.56)	0.139 (0.006, 0.271)	0.004 (0.00002, 0.9)	0.16 (0.05, 0.26)
	Prison 3	0.86 (0, 201.45)	0.02 (0.01, 0.04)	4.93 (0.64, 38)	0.015 (-0.003, 0.034)	0.97 (0.004, 235.07)	0.97 (0.96, 0.99)
	Prison 4	-	-	-	-	-	-
	Prison 5	1.24 (0, 309.4)	0.03 (0, 0.06)	-	-	0.8 (0.003, 209.08)	0.97 (0.94, 1)
	Prison 6	91.96 (0.4, 21160.98)	0.66 (0.49, 0.82)	90.55 (12.19, 672.89)	0.212 (0.02, 0.405)	0.01 (0.00005, 2.6)	0.34 (0.18, 0.51)
	Prison 7	-	0.02 (-0.09, 0.14)	5.03 (0, 109922.9)	0.016 (-0.118, 0.15)	-	0.98 (0.86, 1.09)
	Prison 8	98.17 (38.68, 249.11)	0.67 (-0.31, 1.65)	221.59 (0.01, 4859750.31)	0.385 (-1.501, 2.27)	0.01 (0.004, 0.03)	0.33 (-0.66, 1.32)
	Prison 9	1.98 (0.01, 452.5)	0.05 (0.01, 0.08)	15.11 (1.89, 121.16)	0.045 (-0.008, 0.099)	0.5 (0.002, 118.81)	0.95 (0.92, 0.99)
	Prison 10	14745.1 (54.91, 3959521.92)	1 (0.99, 1)	0.86 (0.08, 9.58)	0.003 (-0.002, 0.007)	0.00007 (0.0000002, 0.02)	0.004 (-0.002, 0.01)
	Prison 11 (comparator)	1.0	-	1.0	0.003 (-0.004, 0.01)	1.0	-
	Prison 12	0.45 (0, 124.06)	0.01 (-0.01, 0.03)	-	-	2.23 (0.008, 633.11)	0.99 (0.97, 1.01)
	Prison 13	80.87 (0.35, 18582.79)	0.63 (0.46, 0.8)	19.16 (2.4, 153.18)	0.057 (-0.01, 0.123)	0.01 (0.00005, 2.97)	0.37 (0.2, 0.54)
**Prison category**							
	A (comparator)						
	B						
	C						
	Closed						
	D						
	YOI						
**Gender**							
	Female (comparator)	1.0	0.5 (0.17, 0.83)	1.0	0.06 (-0.25, 0.38)	1.0	0.5 (0.17, 0.84)
	Male	1.21 (0.01, 274.94)	0.51 (0.43, 0.6)	1.26 (0, 22564.91)	0.07 (-0.09, 0.24)	0.83 (0.004, 197.17)	0.49 (0.4, 0.57)
**Age - years**							
	10 - <20	0.53 (0.12, 2.46)	0.46 (0.34, 0.59)	2.83 (0.58, 13.68)	0.14 (0, 0.27)	1.88 (0.41, 8.64)	0.54 (0.41, 0.66)
	20 - <30	0.94 (0.7, 1.28)	0.51 (0.49, 0.52)	1.19 (0.84, 1.68)	0.08 (0.06, 0.09)	1.06 (0.78, 1.43)	0.49 (0.48, 0.51)
	30 - <40 (comparator)	1.0	0.51 (0.5, 0.52)	1.0	0.07 (0.05, 0.08)	1.0	0.49 (0.47, 0.5)
	40 - <50	0.98 (0.73, 1.33)	0.51 (0.49, 0.53)	1.07 (0.75, 1.52)	0.07 (0.05, 0.08)	1.03 (0.76, 1.39)	0.49 (0.47, 0.51)
	50 - <60	0.99 (0.67, 1.45)	0.51 (0.48, 0.53)	1.07 (0.68, 1.7)	0.07 (0.05, 0.09)	1.02 (0.69, 1.49)	0.49 (0.46, 0.52)
	60 - <70	0.7 (0.38, 1.32)	0.48 (0.44, 0.53)	0.87 (0.36, 2.15)	0.06 (0.02, 0.1)	1.43 (0.76, 2.66)	0.52 (0.47, 0.56)
	70 - <80	1.52 (0.36, 6.44)	0.54 (0.44, 0.64)	2.35 (0.68, 8.14)	0.12 (0.02, 0.22)	0.67 (0.16, 2.78)	0.46 (0.36, 0.56)
	80 - <90	-	-	-	-	-	-
	90 - <100	-	-	-	-	-	-
	100 - <110	-	-	-	-	-	-
**Length of Stay (months)**							
	<1	3.02 (1.86, 4.89)	0.55 (0.53, 0.58)	0.66 (0.43, 1.02)	0.08 (0.06, 0.1)	0.35 (0.21, 0.56)	0.45 (0.42, 0.47)
	1-<6 (comparator)	1.0	0.47 (0.46, 0.49)	1.0	0.11 (0.09, 0.12)	1.0	0.52 (0.51, 0.54)
	6-<12	3.17 (2.26, 4.44)	0.56 (0.54, 0.57)	0.27 (0.18, 0.4)	0.04 (0.03, 0.05)	0.33 (0.23, 0.46)	0.44 (0.43, 0.46)
	12-<24	1.04 (0.76, 1.43)	0.48 (0.46, 0.5)	0.41 (0.28, 0.62)	0.05 (0.04, 0.07)	0.97 (0.71, 1.33)	0.52 (0.5, 0.54)
	24+	1.54 (1.01, 2.33)	0.51 (0.48, 0.53)	0.51 (0.31, 0.83)	0.06 (0.04, 0.09)	0.67 (0.44, 1.01)	0.49 (0.47, 0.52)
**Ethnic Group**							
	White (comparator)	1.0	0.51 (0.5, 0.52)	1.0	0.07 (0.07, 0.08)	1.0	0.49 (0.48, 0.5)
	Mixed	0.89 (0.5, 1.59)	0.5 (0.45, 0.54)	1.22 (0.63, 2.36)	0.09 (0.05, 0.13)	1.02 (0.57, 1.83)	0.5 (0.45, 0.54)
	Asian or Asian British	1.01 (0.64, 1.59)	0.51 (0.47, 0.54)	0.59 (0.29, 1.2)	0.05 (0.02, 0.08)	0.98 (0.63, 1.54)	0.49 (0.46, 0.52)
	Black or Black British	1.17 (0.65, 2.11)	0.52 (0.48, 0.56)	0.52 (0.21, 1.34)	0.04 (0.01, 0.08)	0.85 (0.47, 1.53)	0.48 (0.44, 0.52)
	Chinese and Other	2.33 (0.94, 5.81)	0.56 (0.51, 0.62)	0.25 (0.06, 1.12)	0.02 (-0.01, 0.05)	0.34 (0.13, 0.89)	0.42 (0.36, 0.48)
	Unclassified	2.11 (0.77, 5.8)	0.56 (0.49, 0.62)	0.8 (0.22, 2.86)	0.06 (0, 0.12)	0.48 (0.18, 1.3)	0.44 (0.38, 0.51)

Supplementary section 4. Comparisons with achievement in community settings

Study indicator	Study achievement 2019-20 (%)	Community indicator [a]	Community achievement (England) (%)
**Long-term conditions**			
Blood pressure control in diabetes: proportion of the prison population with diabetes whose most recent blood pressure was ≤ 140/80 mmHg in the last 12 months	261 of 770 eligible people (33.9%)	Quality and Outcomes Framework, 2019-20: The percentage of patients with diabetes, on the register, without moderate or severe frailty in whom the last blood pressure reading (measured in the preceding 12 months) is 140/80 mmHg or less	68.01%(1)
Processes of care for diabetes: proportion of the prison population with diabetes who have had ≥ five of these measurements in the preceding 12 months: body mass index, blood pressure, record of smoking status, foot examination, urine albumin: creatinine ratio, blood tests for HbA1c, cholesterol, creatinine	426 of 770 eligible people (55.3%)	Quality and Outcomes Framework, 2019-20: The percentage of patients with diabetes, on the register, with a record of a foot examination and risk classification within the preceding 12 months	78.04%(1)
Glycaemic control for diabetes: proportion of the prison population with diabetes and without moderate or severe frailty, whose most recent HbA1c was ≤ 58 mmol/mol in the last 12 months	260 of 756 eligible people (34.4%)	Quality and Outcomes Framework, 2019-20: The percentage of patients with diabetes, on the register, without moderate or severe frailty in whom the last IFCC-HbA1c is 58 mmol/mol or less in the preceding 12 months	56.66%(1)
Glycaemic control for diabetes with frailty: proportion of the prison population with diabetes and moderate or severe frailty, whose most recent HbA1c was ≤ 75 mmol/mol in the last 12 months	9 of 14 eligible people (64.3%)	Quality and Outcomes Framework, 2019-20: The percentage of patients with diabetes, on the register, with moderate or severe frailty in whom the last IFCC-HbA1c is 75 mmol/mol or less in the preceding 12 months	83.34%(1)
Asthma review: proportion of the prison population with asthma who had an asthma review in the last 12 months that included an assessment of asthma control	136 of 4,459 eligible people (3.1%)	Quality and Outcomes Framework, 2019-20: The percentage of patients with asthma, on the register, who have had an asthma review in the preceding 12 months that includes an assessment of asthma control using the 3 Royal College of Physicians questions	67.14%(1)
Epilepsy review and control: proportion of the prison population with epilepsy and prescribed antiepileptic drug(s), that had an annual review and were seizure free in the last 12 months	Annual review: 5 of 419 eligible people (1.2%)<br><br>Seizure free: 1 of 419 eligible people (<1.00%)	Quality and Outcomes Framework, 2013-14: The percentage of patients aged 18 or over on drug treatment for epilepsy who have been seizure free for the last 12 months, recorded in the preceding 12 months	57.50%(2)
Secondary prevention of stroke: proportion of the prison population with transient ischaemic attack or stroke (excluding haemorrhagic stroke), prescribed an antiplatelet/anticoagulant in the last 12 months	51 of 86 eligible people (59.3%)	Quality and Outcomes Framework, 2019-20: The percentage of patients with a stroke shown to be non-haemorrhagic, or a history of transient ischaemic attack, who have a record in the preceding 12 months that an antiplatelet agent, or an anti-coagulant is being taken	91.01%(1)
Anticoagulation for atrial fibrillation: proportion of the prison population with persistent/ paroxysmal atrial fibrillation and CHA2DS2-VASc score ≥ two, with a prescription for warfarin/direct-acting oral anticoagulant in the last 12 months	45 of 54 eligible people (83.3%)	Quality and Outcomes Framework, 2019-20: In those patients with atrial fibrillation with a record of a CHA2DS2-VASc score of 2 or more, the percentage of patients who are currently treated with anti-coagulation drug therapy	87.26%(1)
Stroke risk assessment in atrial fibrillation: proportion of the prison population with persistent/ paroxysmal atrial fibrillation, and a CHA2DS2-VASc score in the last 12 months	15 of 25 eligible people (60%)	Quality and Outcomes Framework, 2019-20: The percentage of patients with atrial fibrillation in whom stroke risk has been assessed using the CHA2DS2-VASc score risk stratification scoring system in the preceding 12 months (excluding those patients with a previous CHADS2 or CHA2DS2-VASc score of 2 or more)	93.35%(1)

Study indicator	Study achievement 2019-20 (%)	Community indicator [a]	Community achievement (England) (%)
**Long-term conditions**			
Blood pressure control in people 79 years or under with cardiovascular disease: proportion of the prison population aged ≤ 79 years with coronary heart disease, hypertension, stroke, transient ischaemic attack or peripheral arterial disease, and without diabetes or chronic kidney disease, with blood pressure ≤ 140/90mmHg in the last 12 months	586 of 1,311 eligible people (44.7%)	Quality and Outcomes Framework, 2019-20: The percentage of patients aged 79 years or under, with coronary heart disease, in whom the last blood pressure reading (measured in the preceding 12 months) is 140/90 mmHg or less	77.77%(1)
Blood pressure control in people 80 years or over with cardiovascular disease: proportion of the prison population aged ≥ 80 years with coronary heart disease, hypertension, stroke, transient ischaemic attack or peripheral arterial disease, and without diabetes or chronic kidney disease, with blood pressure ≤ 150/90mmHg in the last 12 months	10 of 19 eligible people (52.6%)	Quality and Outcomes Framework, 2019-20: The percentage of patients aged 80 years or over, with coronary heart disease, in whom the last blood pressure reading (measured in the preceding 12 months) is 150/90 mmHg or less	86.50%(1)
Secondary prevention of myocardial infarction: proportion of the prison population with myocardial infarction, prescribed all of the following in the last 12 months: ACE-inhibitor/ angiotensin receptor blocker, anti-platelet, beta-blocker and statin	0 of 492 eligible people (0%)	Quality and Outcomes Framework, 2014-15: The percentage of patients with a history of myocardial infarction (on or after 1 April 2011) currently treated with an ACE-inhibitor (or angiotensin receptor blocker if ACE-inhibitor intolerant), aspirin or an alternative anti-platelet therapy, beta-blocker and statin	69.06%(3)
Treatment of coronary heart disease: proportion of the prison population with coronary heart disease prescribed anti-platelet/ anti-coagulant therapy in the last 12 months	360 of 494 eligible people (72.9%)	Quality and Outcomes Framework, 2019-20: The percentage of patients with coronary heart disease with a record in the preceding 12 months that aspirin, an alternative anti-platelet therapy, or an anti-coagulant is being taken	90.87%(1)
Treatment of heart failure: proportion of the prison population with heart failure, prescribed an ACE-inhibitor/ angiotensin receptor blocker in the last 12 months	62 of 98 eligible people (63.3%)	Quality and Outcomes Framework, 2019-20: In those patients with a current diagnosis of heart failure due to left ventricular systolic dysfunction, the percentage of patients who are currently treated with an ACE-inhibitor or angiotensin receptor blocker	81.95%(1)
Treatment of heart failure: proportion of the prison population with heart failure, prescribed an ACE-inhibitor/ angiotensin receptor blocker and beta-blocker, in the last 12 months	49 of 61 eligible people (80.3%)	Quality and Outcomes Framework, 2019-20: In those patients with a current diagnosis of heart failure due to left ventricular systolic dysfunction who are currently treated with an ACE-inhibitor or angiotensin receptor blocker, the percentage of patients who are additionally currently treated with a beta-blocker licensed for heart failure	82.01%(1)

Study indicator	Study achievement 2019-20 (%)	Community indicator [a]	Community achievement (England) (%)
**Screening**			
Cervical screening age 25-49 years: proportion of women in prison eligible for cervical screening, aged 25-49 years, who had the test in the last three years and six months	1,145 of 1,796 eligible people (63.8%)	Quality and Outcomes Framework, 2019-20: The percentage of women eligible for screening and aged 25-49 years whose notes record that an adequate cervical screening test has been performed in the preceding 3 years and 6 months	71.48%(1)
Cervical screening age 50-64 years: proportion of women in prison eligible for cervical screening, aged 50-64 years, who had the test in the last five years and six months	159 of 259 eligible people (61.4%)	Quality and Outcomes Framework, 2019-20: The percentage of women eligible for screening and aged 50-64 years whose notes record that an adequate cervical screening test has been performed in the preceding 5 years and 6 months	77.64%(1)
Breast screening: proportion of women in prison eligible for breast screening, aged 50-70 years, who had the test in the last three years	59 of 179 eligible people (33%)	Breast Screening Programme 2017-18: uptake of invitations to screen for women age 50-70 years	70.5%(4)
Abdominal aortic aneurysm screening: proportion of men in prison eligible for abdominal aortic aneurysm screening, aged ≥ 65 years, who have had the test	241 of 540 eligible people (44.6%)	Abdominal aortic aneurysm screening 2014-15: uptake for initial screens. Data are for men turning 65 in the screening year.	79.5%(5)
Cardiovascular disease risk assessment: proportion of the prison population aged 45 to 74 years with cardiovascular disease risk assessment in the last 5 years· People with diagnoses of: coronary heart disease, chronic kidney disease, diabetes, hypertension, atrial fibrillation, transient ischaemic attack, familial hypercholesterolemia, heart failure, peripheral arterial disease, stroke or ≥ 20% 10-year cardiovascular disease risk before the last 5 years, were excluded	353 of 1,183 eligible people (29.8%)	People taking up an NHS Health Check invite per year, England, 2019-20. The NHS Health Check programme aims to help prevent heart disease, stroke, diabetes and kidney disease. Everyone between the ages of 40 and 74, who has not already been diagnosed with one of these conditions, will be invited (once every five years) to have a check to assess their risk of heart disease, stroke, kidney disease and diabetes and will be given support and advice to help them reduce or manage that risk.	43.7%(6)
**Mental illness**			
Antipsychotic monitoring: proportion of the prison population with ≥ three prescriptions for a first or second generation antipsychotic drug, and ≥ five of these measurements in the preceding 12 months: body mass index, blood pressure, blood tests for fasting glucose and/or HbA1c, lipid profile, renal and liver function, full blood count	24 of 463 eligible people (5.2%)	Quality and Outcomes Framework, 2019-20: The percentage of patients with schizophrenia, bipolar affective disorder and other psychoses who have a record or blood pressure and body mass index in the preceding 12 months	80.28% blood pressure<br>74.40% body mass index(1)
Diagnosis of dementia: proportion of the prison population with a new diagnosis of any form of dementia, and a record of ≥ five of these blood tests 12 months before to 6 months after date of diagnosis: full blood count, calcium, glucose and/or HbA1C, renal and liver function, thyroid function, vitamin B12, folate	6 of 13 eligible people (46.2%)	Quality and Outcomes Framework, 2018-19: The percentage of patients with a new diagnosis of dementia recorded in the preceding 1 April to 31 March with a record of full blood count, calcium, glucose, renal and liver function, thyroid function tests, serum vitamin B12 and folate levels recorded between 12 months before or 6 months after entering on to the register	70.01%(7)
**Communicable disease**			
Influenza immunisation: proportion of the prison population with one or more of: coronary heart disease, stroke, transient ischemic attack, diabetes or chronic obstructive pulmonary disease, who received an influenza vaccination August to March	787 of 1,752 eligible people (44.9%)	Quality and Outcomes Framework, 2019-20: The percentage of patients who have had influenza immunisation in the preceding 1 August to 31 March	72.22% diabetes<br>74.22 transient ischaemic attack<br>77.33% coronary heart disease<br>77.44% chronic obstructive pulmonary disease(1)

Study indicator	Study achievement 2019-20 (%)	Community indicator [a]	Community achievement (England) (%)
**Opioid and gabapentinoid prescribing**			
Prescribed any opioid: proportion of the prison population prescribed any opioid during an eight-week period	2,957 of 25,811 eligible people (11.5%)	A record of four or more opioid prescriptions 2011-12	12.4%(8)[b]<br><br>Long-term prescribing was associated with mental health problems (odds ratio 1.12, 1.03 to 1.23)
Prescribed any strong opioid: proportion of the prison population prescribed any strong opioid during an eight-week period	2,257 of 25,811 eligible people (8.7%)	A record of four or more opioid prescriptions 2011-12, and at least one prescription of a strong opioid at any time point (diamorphine, morphine, oxycodone, fentanyl, hydromorphone, buprenorphine (excluding preparations used for substance misuse), pentazocine, dipipanone and papaveretum)	0.85%(8)[b]<br><br>Strong opioid prescribing was associated with mental health problems (odds ratio 1.23, 1.06 to 1.23)

[a] We used results from comparable quality indicators employed in the community and public health data. Data from 2019-20 were used where available, corresponding to the final year of data collection in the study.

[b] Data from West Yorkshire, England.

1. NHS Digital. Quality and Outcomes Framework 2019-20: Prevalence, achievement and personalised care adjustments at regional and national level, 2020 [26 January 2023]. Available from: https://digital.nhs.uk/data-and-information/publications/statistical/quality-and-outcomes-framework-achievement-prevalence-and-exceptions-data/2019-20.

2. NHS Digital. Quality and Outcomes Framework (QOF) - 2013-14: Prevalence, achievements and exceptions at region and nation level for England, 2014 [26 January 2023]. Available from: https://digital.nhs.uk/data-and-information/publications/statistical/quality-and-outcomes-framework-achievement-prevalence-and-exceptions-data/quality-and-outcomes-framework-qof-2013-14.

3. NHS Digital. Quality and Outcomes Framework (QOF) - 2014-15: Prevalence, achievements and exceptions at region and nation level V1.1, 2015 [26 January 2023]. Available from: https://digital.nhs.uk/data-and-information/publications/statistical/quality-and-outcomes-framework-achievement-prevalence-and-exceptions-data/quality-and-outcomes-framework-qof-2014-15.

4. NHS Digital. Breast Screening Programme, England 2017-18: Data tables, 2018 [26 January 2023]. Available from: https://digital.nhs.uk/data-and-information/publications/statistical/breast-screening-programme/england-2017-18.

5. Public Health England. Research and analysis. Abdominal aortic aneurysm screening: 2014 to 2015 data tables, 2015 [26 January 2023]. Available from: https://www.gov.uk/government/publications/abdominal-aortic-aneurysm-screening-2014-to-2015-data.

6. Office for Health Improvement & Disparities. Finger Tips Public Health Data. NHS Health Check: People taking up an NHS Health Check invite per year, 2020 [26 January 2023]. Available from: https://fingertips.phe.org.uk/profile/nhs-health-check-detailed/data#page/4/gid/1938132726/pat/159/par/K02000001/ati/15/are/E92000001/iid/91112/age/219/sex/4/cat/-1/ctp/-1/yrr/5/cid/4/tbm/1.

7. NHS Digital. Quality and Outcomes Framework 2018-19: Prevalence, achievements and exceptions at regional and national level, 2019 [26 January 2023]. Available from: https://digital.nhs.uk/data-and-information/publications/statistical/quality-and-outcomes-framework-achievement-prevalence-and-exceptions-data/2018-19-pas.

8. Foy R, Leaman B, McCrorie C, et al. Prescribed opioids in primary care: cross-sectional and longitudinal analyses of influence of patient and practice characteristics. BMJ Open. 2016;6(5):e010276.
