# Supplementary material for: The quality of prison primary care: cross-sectional cluster-level analyses of prison healthcare data in the North of England
Source: eClinicalMedicine. 2023 Aug 31;63:102171. doi: 10.1016/j.eclinm.2023.102171 (PMC10484963; doi:10.1016/j.eclinm.2023.102171)
Supplement: HSDR170226_Full_20180912_clean [file mmc2.pdf]

Understanding and improving the quality of primary care for prisoners: a mixed-methods study

Summary of Research

Background: Compared to community populations, prisoners have significantly poorer health, with higher levels of long-term illness and disability, and mortality. Prisoners need and are entitled to appropriate health care, especially primary care for long-term conditions, mental illness and primary prevention. However, relatively little is known about their current quality of primary care.

Aim: Our mixed-methods study will explore gaps and variations in the quality of primary care for prisoners and identify quality improvement interventions to promote high quality prison care.

Research objectives:

1. To identify candidate quality indicators based on current national guidance which can be assessed using routinely collected data through a panel of recent prisoners, prison primary care providers and commissioners.

2. To explore perceptions of quality of care, including barriers to and enablers of recommended care and quality indicators, through qualitative interviews involving prisoners and prison primary health care providers.

3. To assess the current quality of primary care provided to prisoners through analysis of routinely held prison primary care records.

4. To integrate the above findings within a stakeholder consensus process (including prisoners) to prioritise and enhance quality improvement interventions which can be monitored by our set of quality indicators.

This programme of work will address a major gap in knowledge about the primary healthcare of a significant but marginalised population. By highlighting variations in primary care, providing insights into their likely causes and working with stakeholders, we will be able to generate a suite of recommended quality improvement interventions that can be realistically applied within the prison healthcare system.

Methods:
Close collaborative links between Leeds Institute of Health Sciences (LIHS), prison health care practitioners and providers (Spectrum Community Health Community Interest Company (CIC)) and commissioners, place us in a unique position to undertake this research. The study will involve four related work packages (WPs).

WP1: We will convene a Stakeholder Panel of 11 participants to consider a list of indicators based on the Quality and Outcomes Framework (QoF), the National Institute for Health and Care Excellence (NICE) quality standards related to prisoners and the Health and Justice Indicators of Performance (HJIPs), and indicators we have previously developed for general practice. The Panel will select quality indicators based upon criteria including the feasibility of assessment using routinely collected data.

WP2: Qualitative interviews with around 15 ex-prisoners and 15 prison healthcare providers will explore attitudes, perceptions and experiences concerning current quality of care provided in prison settings, along with perceived barriers and enablers to optimising care delivery, including the wider contextual factors that influence primary care delivery.

WP3: We will use the proposed quality indicators to analyse data from prison-held primary care records to explore quality of care and variations in care according to particular prisoner groups, conditions and prison primary care. Data extraction will occur in 11 adult prisons served by Spectrum CIC; three category A (high security); four category B (remand); four category C (training); and one category D (open). Their collective total capacity is approximately 8,560 prisoners (range 280 to 1,350)

WP4: Integration and sharing of findings from WPs 1-3 through a structured consensus development process, enabling initial elicitation of all views, social interaction and transparent decision-making. We will identify ways of enhancing existing quality improvement systems or developing new interventions to improve quality, and provide guidance on their implementation. Participants will consist of commissioners of health services, care providers, and prisoners.

Outputs:
Having identified appropriate quality indicators (WP1), gained insights into barriers to and enablers of change (WP2), and analysed inappropriate variations in practice within prison (WP3), we will identify a package of quality indicators and a suite of improvement interventions that can be sustainably embedded within available systems and resources (WP4). We anticipate that our recommended interventions will target individual, team, organisational and wider system levels. Whilst this bid is primarily geared to provide practical insights and guidance for prisoner health care, this project will also underpin longer term work to evaluate the effects, costs and sustainability of interventions to improve quality of prisoner care.

Background and Rationale

The prison population experiences a disproportionately higher burden of illness and poorer access to treatment and prevention programmes compared to community populations. Prisoners consult general practitioners three times more frequently, consult other primary care professionals 80 times more frequently, and receive inpatient care at least 10 times as frequently [1]. They have significant levels of long-term illness and disability [2, 3] and premature mortality [4]. In addition, prison populations have higher rates of communicable disease (including HIV and hepatitis B and C) [5], mental health issues, and drug and alcohol problems [6]. There is clearly a need to ensure that appropriate care of long-term conditions, mental illness and primary prevention is provided to prisoners both during and following their prison sentence. However, relatively little research has examined the quality of primary care provided in prisons, and hence allow comparisons to the general population.

Even in the face of continuing pressures [7], United Kingdom primary care is consistently highly ranked in international comparisons [8]. This standing builds upon the recognised value of strong primary care systems [9] with organised preventive and long term condition care underpinned by an information technology infrastructure, the legacy of National Service Frameworks and (to varying extents) by the Quality and Outcomes Framework (QoF), which linked remuneration to the achievement of evidence-based quality indicators [10]. While significant advances have been made in improving care for the population as a whole, variations still exist, not least in relation to those patients with the most complex health needs or marginalised communities [11], such as prisoners.

Most research with prison populations has understandably prioritised drug misuse, mental health and communicable disease. Recent examples include the implementation of indoor smoke free prison facilities [12]; drug treatment of young male prisoners with attention-deficit hyperactivity disorder [13]; care pathways for older prisoners [14], particularly those with cognitive impairment [15]; outcomes for forensic services for people with intellectual and/or developmental disabilities [16]; organisation of care for those, near to and after release, with common mental health problems [17] or with serious mental illness [18]; and, peer-based interventions to maintain and improve offender mental health [19].

However, relatively little attention has been given to common (or even ‘routine’) conditions which affect the quality and length of life, including cardiovascular and respiratory health (e.g. hypertension, asthma), and which are amenable to evidence-based treatments [20, 21]. There has been little or no exploration of variations in the quality of care across prisons and between particular prisoner groups in the UK, nor work to explain any variations. Furthermore, providing routine health care is highly challenging within the prison environment; any improvement initiatives need to be realistically based on an in-depth understanding of constraints and norms within prisons. For example, a recent randomised controlled trial found that an Older prisoner Health and Social Care Assessment and Plan (OHSCAP) did not improve the primary outcome, the mean number of unmet health and social care needs, compared to usual care [22]. Process evaluation data suggested that the intervention was not implemented as planned, partly attributable to wider challenges in the prison context, including staffing shortages, the loss of specialist support roles for such initiatives, and regime disruption.

We have previously developed and applied a set of ‘high impact’ quality indicators for primary care, based on criteria including: burden of illness (e.g. prevalence, severity), potential for significant patient benefit (e.g. longevity, quality of life), scope for improvement upon current levels of achievement, and the feasibility of measurement using routinely collected data [23]. Other indicators are available but not yet routinely applied to prison populations, including primary care quality indicators for people with serious mental illness [24]. We are now in a position to build upon these, understand variations in prison primary care, and initiate strategies to improve prisoner healthcare and outcomes. For example, the detection and management of hypertension

reduces avoidable mortality and morbidity [25]; there is scope for improving upon current management in primary care, whereby just over two-thirds of people with hypertension are achieving treatment goals [26]. The detection and treatment gaps in the prison population are unknown, thereby undermining priority setting and planning to reduce avoidable cardiovascular events in this population.

From April 2013, NHS England became responsible for commissioning all health services (with the exception of some emergency care, ambulance services, out of hours and 111 services) for people in prisons in England through 'Health and Justice' commissioning teams, supported by a national Health and Justice team. NHS England has set a key commissioning strategic goal to reduce the respective gaps in healthcare and health outcomes between those in criminal justice and the rest of the population [27]. Whilst steps have already been taken to bring about equity of care for prisoners, most significantly by integrating prison healthcare into the wider NHS, these steps focus on equality in relation to service configuration, rather than receipt and outcomes of care.

In summary, the prison population is broadly recognised as having greater healthcare needs and poorer outcomes than the general population. Whilst most research in this population has focused on specific health needs (e.g. mental health), less is known about the extent to which prisoners receive recommended routine primary care.

Evidence explaining why this research is needed now

Our proposal aims to drive a new improvement agenda for the primary care of prisoners, which will address inappropriate variations between and within prisons as well as inform strategies to close the likely gaps in health care and outcomes between prison and community populations. We are ideally placed to build upon our links with commissioners and providers of prison healthcare, and work through how to integrate our research findings with service planning, delivery and monitoring. This proposal addresses current NHS England commissioning priorities, aligns with the drive to develop Health and Justice Indicators of Performance (HJIPs), and draws upon new national guidelines.

Commissioning priorities: The recent NHS England commissioning strategy to improve outcomes for prisoners outlines the current scale of the problems for prisoners highlighting poor health and poorer access to treatment and programmes [27]. It highlights the wider impact of socioeconomic influences on prisoner health, including homelessness, unemployment, and poorer education attainment. These health and social issues both contribute to and follow patterns of offending and re-offending. Therefore health and care services can have impacts on both the individual and wider society. Our planned work therefore fits with the commissioning strategy in aiming to inform and guide quality improvement for the prison population.

HJIP development: There are opportunities to build upon existing data sources and analyses. For example, Health Needs Assessments (HNAs) are produced for each prison to support the commissioning process. These HNAs provide a summary of current health needs and practice, based upon QoF, local and national quality frameworks, and the HJIPs. The HJIPs have been developed over recent years to support key strategic programmes and commissioning. Whilst the quality of data and reporting of the HJIPs has continued to improve since their inception, there is scope to enhance their breadth and reach [28]. Limited prison level comparison of these indicators has been carried out, however as of yet, prison level comparison has not accounted for factors that might explain any variation or across prisoner groups.

National guidelines: The National Institute for Health and Care Excellence (NICE) has issued new guidelines on the identification and treatment of physical ill health in prisons (November 2016) and mental illness (March 2017) in the criminal justice system [29, 30]. Active strategies are generally needed to support the implementation of guidelines [31], and any such strategies need to be informed by data on patterns of uptake, including variations between prisoner groups and between prisons. Our study will be novel in assessing variations in adherence to these guidelines, exploring reasons for variations and hence informing the targeting of interventions to improve the quality of primary care for prisoners.

Aims and objectives

Our mixed-methods study will explore gaps and variations in the quality of primary care for prisoners and identify quality improvement interventions to promote high quality prison care.

Research objectives:
1. To identify candidate quality indicators based on current national guidance which can be assessed using routinely collected data through a panel of recent prisoners, prison primary care providers and commissioners.

2. To explore perceptions of quality of care, including barriers to and enablers of recommended care and quality indicators, through qualitative interviews involving prisoners and prison primary health care providers.

3. To assess the current quality of primary care provided to prisoners through analysis of routinely held prison primary care records.

4. To integrate the above findings within a stakeholder consensus process (including prisoners) to prioritise and enhance quality improvement interventions which can be monitored by our set of quality indicators.

Close collaborative links between Leeds Institute of Health Sciences (LIHS) at the University of Leeds, prison health care practitioners and providers (Spectrum Community Health Community Interest Company (CIC)) and commissioners, places us in a unique and strong position to undertake this research.

This programme of work will address a major gap in knowledge about the primary healthcare of a significant but marginalised population. Using evidence-based indicators we will evaluate the current quality of care and highlight variations in primary care provided to prisoners, determine providers' and users' perceptions of barriers and enablers to quality and then generate a suite of recommended quality improvement interventions that can be realistically applied within the prison healthcare system.

Research Plan

Research design and setting

This mixed-methods study will identify a set of quality indicators for prison primary care, explore the behavioural and contextual factors that influence primary care delivery, identify gaps and variations in the quality of care, and prioritise interventions to improve prisoner healthcare and outcomes.

Close collaborative links between LIHS, prison health care practitioners, commissioners and a major provider, Spectrum Community Health Community Interest Company (CIC), place us in a unique position to undertake this research. The study will involve four related Work packages (WP) corresponding to each research objective.

Immediately after study commencement, we will conduct a focused scoping review to identify any recent qualitative and quantitative research on prisoner quality of care. This will further inform identification of indicators (WP1) and interview planning (WP2).

WP1: Identification of quality indicators (Objective 1)

DESIGN: We will use a consensus development process to identify and select quality indicators for the prison population which can be assessed using routinely collected data. A structured deliberation enable initial elicitation of all views, social interaction and transparent decision-making.

PARTICIPANTS: We will convene a stakeholder panel to consider the list of the indicators and identify quality indicators which can be assessed using routinely collected data. The panel will comprise 11 people drawn from prison health practitioners, prison officers, probation workers, commissioners and prisoner representatives. We will ensure that each of these groups is represented. At least two panel places will be reserved for participants who have a specific commissioning, practitioner or policy role regarding female prisoner health. We will ensure that they include at least one prisoner representative and one prison practitioner/policy maker/commissioner who works in or is responsible for the female estate. This will allow strategic consideration of the needs of the female prison population. Consensus groups gain relatively little in reliability exceeding 11 participants [32].

METHODS: We will use a modified RAND process, which is suitable for judgements requiring some degree of deliberation and discussion [32]. We will initially ask panellists to rate a list of candidate indicators independently and online. The candidate indictors will be drawn from the Quality and Outcomes Framework

(QoF), the National Institute for Health and Care Excellence (NICE) prison health guidelines and quality standards, the Health and Justice Indicators of Performance (HJIPs) via the Public Health England (PHE), the Health and Justice health needs assessment toolkit and Care Quality Commission (CQC) inspection standards. We will also include a set of indicators for community general practice developed in an earlier NIHR-funded programme [23]. The panellists will rate indicators on a 1-9 scale according to: burden of illness, potential for significant patient benefit, anticipated scope for improvement upon current levels of achievement, and (critically) feasibility of measurement using routinely collected data [23].

Aggregate rankings will be fed back at a face-to-face meeting of panel members. Structured discussion will then centre on the recommendation rankings over which there is maximal discordance (provisionally defined as at least three panellists scoring 1–3 and at least three scoring 7–9). Panellists will have the opportunity to clarify aspects of indicators and discuss reasons for low or high rankings. Immediately after this discussion, panellists will again independently rate each indicator.

ANTICIPATED OUTPUTS: We will develop a set of quality indicators relevant to the prison population, and take those with the highest rankings (particularly feasibility) forward to Work Packages 2 and 3. However, some of these will cover specific conditions or needs strongly associated with prisoner status, e.g. substance misuse, mental illness. Others will cover conditions or needs commonly managed in community general practice, e.g. asthma, cervical screening. We recognise that recent or existing research studies are addressing and aiming to improve quality of care for the former [13, 15-19, 22]. We will not attempt to duplicate such work unnecessarily. We will therefore focus our efforts around indicators relatively neglected by current prisoner research i.e. common (or even ‘routine’) conditions. Furthermore, we do not envisage separate indicators for each health condition, e.g., a quality indicator for monitoring blood pressure will be required for patients with either diabetes or hypertension. Cross-condition indicators will be particularly relevant for those with mental illnesses (schizophrenia, bipolar affective disorder and other psychoses). Evaluating physical care indicators (e.g. blood pressure recording) for those patients identified with one of the mental illness will assess whether these prisoners are also being treated for their physical health, an important aspect for quality care of prisoners with mental health problems in general. To determine whether prisoners are routinely included among patients targeted for primary prevention, we will assess recorded smoking status and smoking cessation advice or therapy (increasingly relevant with the phasing in of smoke-free prisons), and if cervical screening was performed for women prisoners. To assess quality and continuity in medicines management, we will review relevant repeat medications related to particular health conditions and determine whether prisoners were provided with necessary treatment (e.g. an anti-hypertensive drug if BP ≥150/90 or a statin if cholesterol ≥5 mmol/l).

We will also explore the feasibility of developing prison-specific indicators from routinely recorded data for substance misuse, sexually transmitted infections (STIs), health assessments, medicines reconciliation and prescriptions on discharge, based on the NICE quality indicators, HJIPs via PHE Health and Justice HNA toolkit and CQC inspection standards.

WP2: Perceptions of quality of care and levers for change (Objective 2)

DESIGN: We will use qualitative interviews to explore attitudes, perceptions and experiences concerning the quality of care currently provided in the prison setting, along with perceived barriers and enablers to achieving optimal care delivery.

PARTICIPANTS: We will recruit around 30 participants. The exact number will depend on achieving both coding and meaning saturation [33]. Participants will be split between two groups: ex-prisoners (approximately 15 participants) and providers of prison based healthcare (15 participants). Recent ex-prisoners will be involved at this stage, rather than current prisoners, as it is unlikely that permissions and access will be in place at the start of the study period (current prisoners will be involved in WP4). Our definition of a ‘recent ex-prisoner’ will be a person who has been incarcerated and then released within a maximum of the past 18 months. We will not set a minimum limit on how long the ex-prisoner was incarcerated for as it is known that remand prisoners – who are typically sent to prison for shorter periods of time – often receive worse continuity of care.

Purposive sampling will be employed. Ex-prisoners will be sampled on age, gender, ethnicity, length of sentence and health status. We will sample prison based care providers from several establishments in the North of England which provide for a mixture of male, female and remand/sentenced prisoner types. Due to

the participants consisting of two distinct groups, our sample size of 30 participants is justified to ensure heterogeneity within each sub sample.

INTERVIEW CONDUCT: We will consider and select two quality indicators from WP1 in order to anchor the interviews. Interviews will explore current need for care and screening related to these two quality indicators, prisoner access to care in a general sense and perceptions of the current quality of care provision more widely. We will also concentrate on how quality of care could be improved. Therefore, the dialogue during the interview will be focused broadly on quality of care but also contain concrete foci of the quality indicators. Topic guides will be tailored appropriately for use with the different groups of participants. Interviews will be recorded and fully transcribed.

ANALYSIS: Analysis will proceed on two levels. Firstly, an inductive thematic analysis [34] will take place which will focus on answering the research objectives. That is, the barriers and levers of quality of prison healthcare will be explicitly drawn out alongside an understanding of what is important to the participants themselves regarding this issue. This approach will be iterative as preliminary insights gathered during fieldwork will then assist in partially shaping the resultant coding framework. The data arising from the different participants groups will be compared and contrasted, with discordant cases actively sought. We may find that the different groups of participants are in broad agreement or that their views contrast with each other. This thematic analysis will involve a process of organising the data, descriptive coding, interpretive coding, writing and theorising. *NVivo* software will be used to aid sorting and categorisation of the data.

Secondly, a conceptual analysis will be undertaken based on an existing theoretical framework pertaining to improving the quality of healthcare [35]. This framework purports that change in quality of care is dependent on a multi-level approach, consisting of: the individual (attitudinal), the group/ team (clinical microsystems and team culture), the organisation (staffing/ resource allocation), the larger system (policy/commissioning decisions). Conducting such an analysis will allow us to understand factors that are operating at the micro, meso, macro and super macro levels. This style of analysis will allow for conceptual understandings of the data to be generated and will move beyond the descriptive approach which will be undertaken in the traditional thematic analysis stated above. The thematic analysis allows the participants voices to be heard and gives to credence to their stated perceptions and experiences. The conceptual analysis moves beyond 'what the participants' said' to attempt understanding how quality of care can be improved with reference to the theoretical change management literature, and what the levers for change are. An abductive approach to analysis will be taken [36]. This involves iterative cycles of analytical interpretation between the theoretical literature and empirical data.

OUTPUT: We will develop an understanding of the individual, team, organisational and system-level factors that influence the provision of care. We will particularly focus subsequent work on those which appear relatively amenable to change.

WP3: Quality of primary care in the prison setting (Objective 3)

DESIGN: We will analyse routinely collected data to investigate the care currently provided to prisoners from a prison primary care perspective based on the quality indicators identified in WP1. We will explore variations in the quality of care according to particular prisoner groups, conditions and prison primary care.

SAMPLING and SETTING CONTEXT: The data will be extracted for those prisons where Spectrum CIC provide primary care services via SystmOne Prison, the IT system that currently holds the data for prison based health care for all prisons in England (new IT system Health & Justice Information System (HJIS) in place in next 18 months). Spectrum CIC are currently responsible for primary healthcare in 11 adult prisons: three category A (high security); four category B (remand); four category C (training); and one category D (open). These collectively have a capacity of approximately 8,560 prisoners in total (range 280 to 1,350).

DATA EXTRACTION: SystmOne Prison contains prisoner demographics (automatically updated from the Prison National Offender Management System), health screening data from a prisoner's first reception in prison, and data related to ongoing care including morbidity data (Read codes), pathology results and prescribing. It allows transfer of records between prisons, ensuring a complete patient health record regardless of where a term is served.

For those quality indicators identified from the stakeholder panel (WP1), algorithms will already have been developed to examine the receipt of recommended care and achievement of targets. As these indicators will be largely based on national guidance (i.e. QoF, NICE, HJIPs and CQC) that are now being collated by

commissioners and commissioning support units (CSUs) for monitoring purposes from providers via guidance and templates, there are coding algorithms in place. Whilst the future of QoF is uncertain, it has established standardised approaches to the routine collection of clinical data in general practice. Individual-level data including achievement of the various indicators, demographic, screening and clinical information will be obtained for the period 1 April 2015 to 31 March 2018. Relevant prison-level data (e.g. prison category) will also be included for each individual. This 36 month timeframe will ensure that we encapsulate the various potential sentences of prisoners (long term through to a number of short sentences – exemplars in flowchart below) and also include sufficient retrospective follow-up to monitor achievement of some of the indicators (e.g. QoF period). We will also account for amendments, removals and additions to quality indicators over the time frame.

We already have an initial agreement with Spectrum Community Health CIC to extract the quality indicators for the prisons where they are responsible for primary healthcare from SystmOne Prison and its replacement (see attached letter). Following the standard Her Majesty's Prison and Probation Service (HMPPS)/Health Research Authority(HRA) approvals required as part of governance procedures, the data made available to staff at LIHS will be anonymised (e.g. age groups provided rather than date of birth, Index of Multiple Deprivation score rather than addresses in community). While the current study involves a data snapshot for a specific time period, the products of this process – permissions, techniques to generate relevant quality indicator codes, algorithms for interrogating data, determining completeness and comprehensiveness of the indicators – allow these methods to be translated to subsequent research and monitoring.

Flowchart: Exemplars of records of prisoner health data – shaded sections represent data available for study

Figure 1: Data for those imprisoned and released once within a selected timeframe (1 April 2015– 31 March 2018)

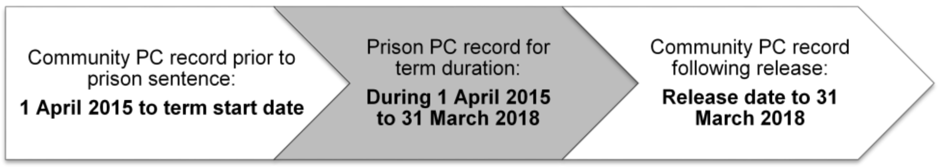

Figure 2: Data for those imprisoned and released more than once within a selected timeframe (1 April 2015– 31 March 2018)

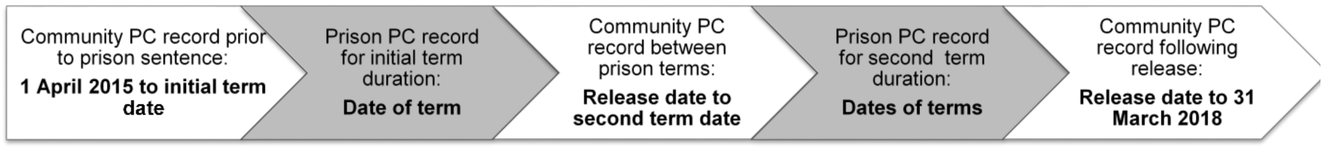

Figure 3: Data for those serving longer terms and/or imprisoned prior to the selected timeframe (1 April 2015– 31 March 2018)

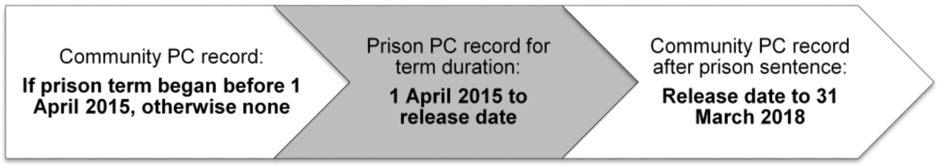

The extraction of the anonymised quality indicator data will be carried out by the Research Assistant employed by and based at Spectrum CIC. As a staff member of the healthcare provider organisation, the research assistant based at Spectrum will have the necessary permissions to have direct access to the records; under our guidance, they will develop and apply the algorithms and anonymise the data for subsequent analysis in Leeds Institute of Health Sciences (LIHS). A Service Level Agreement will be in place between Spectrum CIC and LIHS to transfer the anonymised data in compliance with requirements outlined in the Her Majesty's Prison and Probation Service (HMPPS)/Health Research Authority (HRA) approval.

DATA ANALYSIS: This WP involves analysing data from prison-held primary care records to explore quality of care of prisoners, and the variation and factors that are associated with any variation for particular prisoner groups, prisons or conditions. These data will be analysed to determine the quality of care that prisoners received across the years for each of the quality indicators, along with the use and uptake of preventive services.

The potential validity of the data to be analysed will be integral to the selection of the quality indicators (WP1). The consensus panel criteria for identifying quality indicators will include an assessment of whether the indicators can be collated from routine data. Exploration of perceptions of quality of care (WP2) will also shed further light on the potential completeness and accuracy of the indicator data, and factors that influence variability in their completeness and accuracy. Furthermore, as part of the contract management process between commissioners and prison healthcare providers, Spectrum CIC extract data quarterly for the North of England Commissioning Support Unit (NECSU). NECSU is then responsible for processing these data to provide a dashboard for providers and commissioners. The research team will have access to these anonymised dashboards for Spectrum, via NS and an appropriate Service Level Agreement. These dashboards are likely to include some of our planned quality indicators, thereby allowing a quality control comparison with data extracted and processed by the research assistant at Spectrum.

We share considerable experience of using or adapting clinical data routinely collected for QoF in research and service evaluation. In addition to determining percentage achievement of quality indicators for the prisoner population as a whole, comparisons by prisoner type, sentence duration and relevant prisoner characteristics (age, gender etc.) will determine whether there are systematic differences in quality for these factors; one hypothesis being that long stay prisoners are likely to have relevant quality indicators recorded in prison, whilst those serving shorter sentences (e.g. <3 months) may be overlooked or even not apply in the time they are in prison. Exploring prisoner characteristics, such as age, is particular pertinent with the growing number of elderly prisoners (both in absolute terms and as a proportion of the prison population); and the accompanying poorer health of aging prisoners [37].

We will compare the initial summary and descriptive analysis of the attainment rates of the indicators by prison and prisoner groups with the contextual background elicited in WP1 and 2 to check the credibility of the indicators. We will also be able to triangulate and 'sense check' the data completeness and validity of our proposed indicators and findings with existing prison Health Needs Assessments (HNAs) via our links with the Health and Justice commissioning teams and the prison health care provider. Furthermore, using a Bayesian analysis framework we can estimate the false negative and positive rates of the attainment rates for the prisons and prisoner groups, which would provide further information on the completeness and reliability of the data [38].

As illustrated in the flowchart (above), the length of sentence and the potential number of sentences over the timeframe needs to be considered when assessing achievement of the quality indicators. For example, if some is diagnosed with hypertension and their blood pressure is measured and treated whilst in the community, they might not require monitoring while in prison for a short sentence (e.g. couple of months). Therefore, the timing, duration and number of sentences for each person will be important to consider when assessing achievement of particular quality indicators.

The initial descriptive and Bayesian analysis will provide the necessary information (i.e. achievement numbers and levels) to assess whether further analysis is appropriate. Specifically, multi-level logistic regression models will identify the factors that are associated with achievement of the quality indicators. These types of models will be employed as they can appropriately account for the potential correlations between outcomes at multiple time-points and between-prison variation. WP1 and WP2 will provide contextual background to understand achievement levels for the quality indicators across prisons. These between-prison differences will need to be accounted in exploring variations in attainment. With likely strong associations between the different quality indicators, latent variable models will be developed to identify factors common across all indicators. This unifying model will explore which factors are associated with quality in care in prisons as measured by all these indicators. We will also compare the QoF-based quality indicator achievement rates for these prisons with published QoF rates to assess the quality of healthcare of prisoners relative to current community primary care.

SAMPLE SIZE CONSIDERATIONS: This WP aims to explore variations in the quality of primary care provision for prisoners. As such a formal sample size calculation is not appropriate as the aim is to describe the current provision. However a consideration of the characteristics of the study population shows the

potential to explore the variation in prisoner groups and prisons. Taking a potential health condition with a relatively low prevalence - mental illnesses (schizophrenia, bipolar affective disorder and other psychoses), we would expect 77 prisoners to be diagnosed from the 11 prisons (0.9% prevalence based on QoF for England 2015-16 [37]). This expected number of prisoners diagnosed does not take into account the probable higher prevalence in prisoners nor the throughput of prisoners which would increase the sample population over the 3 years and so those diagnosed. For example, as a remand prison Durham has on average 60% of prisoners transferred within 50 days. If we use the lowest achievement (49%) of the QoF indicator 'The percentage of patients with a comprehensive care plan documented in the record, in the preceding 12 months', we would expect 38 of the 77 prisoners to achieve the QoF indicator. If we use the rule of thumb that logistic models should be used with a minimum of 10 events per factor/predictor variable, based on simulation studies [39], then we would be able to explore approximately 3 factors simultaneously as to whether they are associated with achievement of the indicator. Therefore even with the lowest prevalence of both the condition and achievement of indicator, based on community data, we have potentially a more than sufficient study population size to explore the factors associated with variation in quality indicators.

The initial descriptive analysis will provide the necessary information (i.e. achievement numbers and levels) to assess whether further analysis (i.e. multi-level and latent models) are appropriate. The Bayesian analysis approaches will reveal understanding of the impact of false negatives and positives on the subsequent multi-level models. Furthermore, considering indicators within a unifying model i.e. latent models, will likely result in the number of records required to be reduced and allow more factors to be explored with the same number of records.

OUTPUT: We will understand the current quality of care and the variations in care according to particular prisoner groups, conditions and prison primary care based on appropriate quality indicators.

WP4: Identification of interventions to improve prisoner health (Objective 4)

We will integrate multiple perspectives from WPs 1-3 within a structured deliberation to prioritise quality improvement interventions for prison primary care. As for WP1, the consensus panel of 11 members will be drawn from key stakeholders involved in commissioning and delivering primary care, along with prisoners and their advocates. We will seek consensus on key areas for intervention, and on what kinds of quality improvement interventions need to be enhanced or adopted to improve quality of care.

METHOD: We will share our findings from WPs1-3, with the panel and take them through the following steps:

a. Reviewing findings from WP3 to identify priorities for improvement based on scope for improvement for each indicator, i.e. low performance; groups of prisoners associated with lower achievement of indicators, e.g. older people, longer or shorter term prisoners.
b. Considering findings from WP2 to understand barriers to and enablers of good quality of care, considering levers for change at individual, team, organisational and wider system levels.
c. A briefing summarising the range of approaches (interventions) potentially available to support the implementation of quality indicators, drawn from a broad overview of systematic reviews (or most recent updates then available) [37].
d. Mapping of implementation interventions to identified barriers and enablers. For example: (i) if clinical staff or teams are unaware of their poorer performance relative to other clinical staff and teams, then audit and feedback comparing data on performance can help identify erroneous perceptions and use social comparisons and goal setting to motivate change; (ii) if clinical staff under pressure within time-limited consultations are unable to recall key information about patients or knowledge of care pathways, then specifically targeted computerised prompts and templates can support decision-making and action; or (iii) if prisoners with long term conditions, such as asthma, do not consistently understand the importance and consequences of requesting or taking preventive treatment (or how to do so), targeted patient information and instruction from clinical staff can support treatment adherence. These examples illustrate types of approaches requiring planning and action at individual, team, organisational and system levels.
e. Application of APEASE criteria (affordability, practicability, effectiveness, acceptability, safety and equity) [40]. For each intervention, we need to consider likely adaptability to and sustainability within the prison healthcare environment.

We plan three consecutive panel meetings to allow sufficient time and reflection to work through these five sets of considerations.

ANALYSIS: We will use ratings by panellists for steps 1 (priority setting) and 5 (appraising applicability of candidate interventions). As for WP1, for each of these panellists will independently rate each priority or feature of an intervention (e.g. affordability) on a 1-9 scale, where scores of '1' indicates the strongest disagreement and scores of '9' indicate strongest agreement. We will collate the scores for each and feedback the median and range scores to all participants for a face-to-face discussion. We will discuss ratings, focusing on those with maximal discordance, defined as at least three panellists rating a priority or intervention feature 1–3 and at least three rating 7–9. Participants will then independently rate each item again. This process offers a relatively transparent and inclusive approach to select priorities and interventions, so that those with the highest aggregate scores are carried forward. Written notes will be taken by participating members of the research team during and after workshops and these, together with any materials developed by participants as part of their evaluation, will be included in the analysis. Some sections of the workshops (with participants' permission) may be recorded and transcribed.

Using the findings from the workshops a prioritised list of quality indicators and a suite of implementation interventions relevant to the prison healthcare setting will be identified, likely to be based around the commissioning of prison healthcare, development and sharing of patient records, and delivery of prison-based primary care. Whilst we do not want to pre-judge the findings of the proposed research, or the inductive process that development of interventions will entail, we anticipate that these may fall into three broad categories (further details in Projected Outputs section): individual/team (e.g. educational workshops), organisations (e.g. feedback of performance data, commissioning specifications), and systems (e.g. shared community-prison records).

Dissemination and projected outputs

Commissioners, providers and policy makers' dissemination:

We shall use an explicit framework developed within the Leeds Institute of Health Sciences (LIHS) to guide our knowledge transfer strategy [40, 41]. The most frequently reported barrier to the use of research by decision-makers is the lack of personal contact between researchers and decision-makers [42]. We shall build upon strong existing links between the research, practice and policy communities.

The networks and positions of team members will not only enable identification of participants for interviews, workshops and panels, but will also ensure that the findings of the study feed into the relevant commissioning and provider bodies. For example, NW and KM have direct experience in the organisation and delivery of care through Spectrum Community Health CIC and Care UK, which provide health care to over 50 prisons and are at the forefront of a range of initiatives in the sector; while NS is Lead for Health and Justice Commissioning Support at NECS (North of England Commissioning Support) – embedded within NHS England, the lead commissioner of Health and Justice related services for patients in a prison setting.

RF shares research findings with and advises NICE, particularly via its Implementation Strategy Group, the Healthcare Quality Improvement Partnership and a number of national clinical audits.

We will contact *Inside Time* to elicit interest in our work and its dissemination. We will also enquire as to whether *Inside Time* would be interested in an article explaining our project and informing readers about our planned data collection shortly after requisite ethical and governance approvals are in place.

Dissemination Workshop:

During the last phase of the study, a workshop will provide a forum for engaging with key stakeholders, including prisoners and their advocates (e.g. Howard League), and will include those who have not participated in the study, thereby widening dissemination.

Academic Dissemination:

We aim to publish four papers (one with the main findings of the study and one for each WP 2-4) in relevant target journals, (e.g. BMJ for findings of interest to a general readership and International Journal of Prisoner Health or BJGP for findings of specific interest to primary and prison healthcare professionals). We will prioritise open access publishing as a means of enabling wider access to our findings and therefore potentially faster uptake and implementation. We will also disseminate our findings at two relevant national conferences, most likely the Society for Academic Primary Care and Offender Health Research Network Annual Conferences.

Projected Outputs:

These will directly guide initiatives to improve quality of care for prisoners, and establish a foundation for further intervention development and evaluation.

GUIDING IMPROVEMENT: Having identified existing quality improvement interventions, gained insights into barriers to and enablers of change (WP2), and explored the quality of care and inappropriate variations in practice within prisons (WP3) based on appropriate quality indicators (WP1), we will develop a suite of improvement interventions that can be sustainably embedded within available systems and resources (WP4). We anticipate that our recommended interventions will operate across different levels:

- Individual/team level, e.g. educational workshops, targeted computerised prompts and decision support. There is a substantial evidence-base underpinning such interventions, and this research will help adapt that evidence to the challenging context of prisoner care.
- Health provider organization, e.g. feedback of comparative data on performance, commissioning specifications. We will synthesise findings to include in the commissioning guide for prisoner healthcare, specifying the roles and expectations of prison and community services.
- System level, e.g. enhancements to record keeping, summary shared record. We can establish the importance of enhanced record keeping, and potentially for shared information across the prison/community interface.

FURTHER EVALUATION: Whilst this bid is primarily geared to provide practical insights and guidance for prisoner health care, our longer term goal is to further develop and evaluate interventions to improve quality of prisoner care using rigorous methods, with embedded process and economic evaluations. We will have demonstrated the feasibility of gaining permissions, techniques to generate relevant quality indicator codes, algorithms for interrogating data, determining completeness and comprehensiveness of the indicators which can be used as outcome measures.

Plan of investigation and timetable

The majority of activities in the project are sequential, but there will be some overlap in relation to the setup of individual work packages and the analysis of qualitative and quantitative data streams (see Gantt chart).

GANTT CHART OF QUALITY OF PRISONER CARE PROJECT MILESTONES

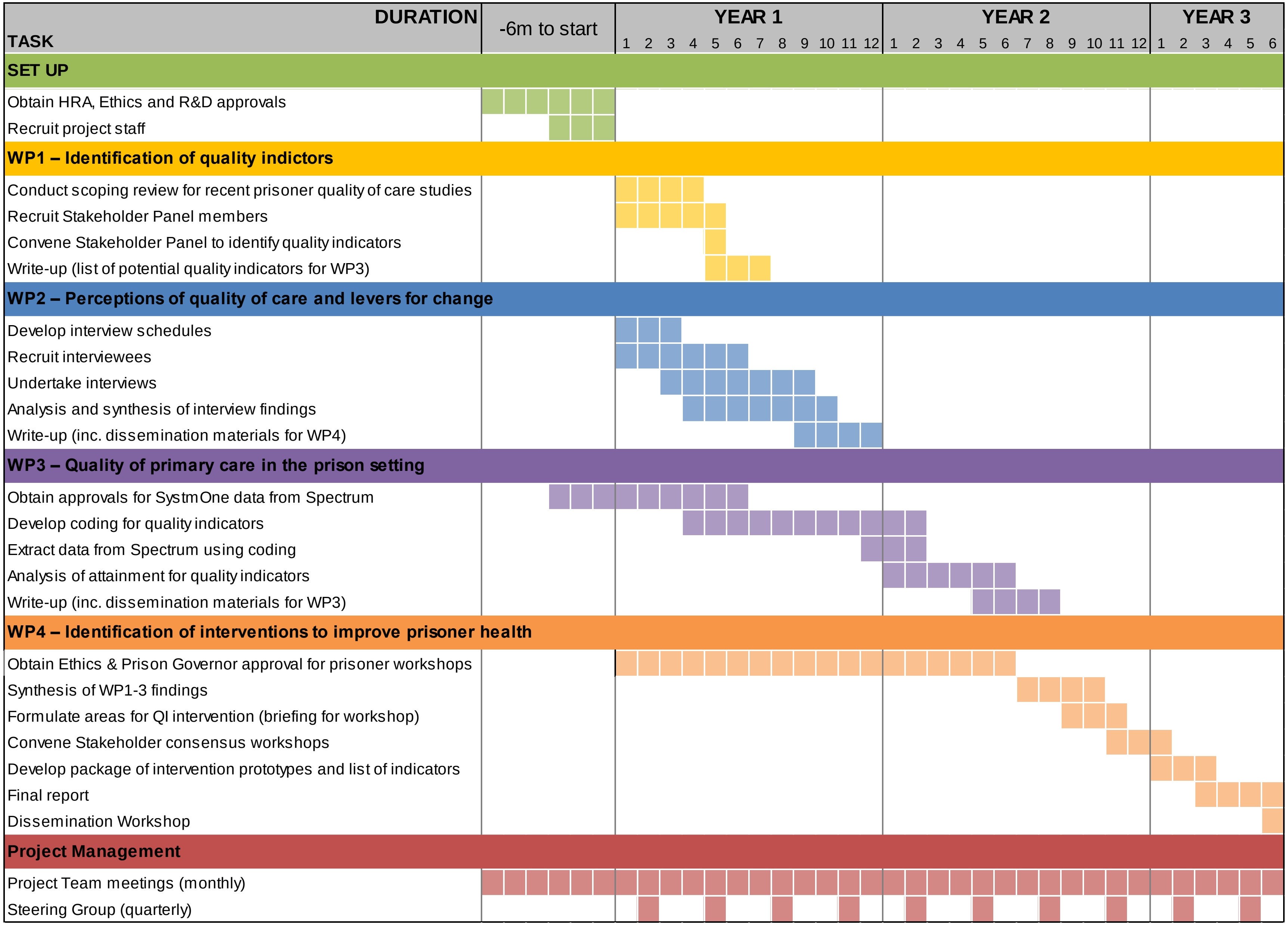

Project management

The named Principal Investigator (LS) will be overall strategic lead but will also work closely with an acting Co-Principal Investigator (RF). Through this joint responsibility and mentoring, we will contribute to the development of LS's leadership abilities and capitalise upon RF's experience. LS and RF will gain the required ethical and research governance approvals prior to the project start and then LS will line manage all three research staff employed on this study. Administrative and communications support will be provided within the Leeds Institute of Health Sciences (costs as outlined).

Individual team members will lead on specific work packages according to their skills and experience:

WP1: identification of quality indicators (RF and TF as lead, NW, KM)

WP2: qualitative work (LS as lead, NW, EM, NS),

WP3: analysis of routine data (TF as lead, EM, RF),

WP4: identification of interventions (RF as lead, NW, KM, EM, LS, NS).

A Project Management Team will meet monthly, with individual leads convening WP specific meetings as required. The Research Programme Manager and Fergus will be responsible for PPI liaison and recruitment.

A Steering Group will meet quarterly to oversee progress and assist in interpretation of results, with the research team in attendance. Other collaborators, as Steering Group members, will provide advice from a range of perspectives: e.g. Kate Davies is Director of Health & Justice, Armed Forces and Sexual Assault Services Commissioning, NHS England, (which has overall responsibility for commissioning prison health services); Dr Linda Harris is Chief Executive of Spectrum CIC. Additional, ad-hoc meetings may be arranged depending on the advisory needs of the Research Team.

Achievement of the milestones and deliverables outlined in the Gantt chart is the basis on which success criteria will be measured. These will be monitored regularly via monthly research team meetings and the quarterly Steering Groups.

We propose inclusion of the following triggers for a formal review by the HS&DR Programme:

1. Failure to agree and implement service level agreements for data sharing between the Leeds Institute of Health Science, University of Leeds and Spectrum CIC (within the first 12 months),
2. Failure to secure or demonstrate significant progress with seeking Her Majesty's Prison & Probation Service approval to conduct WP4 (within the first 18 months as WP4 does not start until months 23 and 24),
3. Loss of four or more (out of 11) contracts for Spectrum CIC to provide prison primary care (within the first 18 months).

Approval by ethics committees

Standard Health Research Authority/NHS Research Ethics and Her Majesty's Prison & Probation Service (previously National Offender Management Service) approvals for WP1-3 will be sought prior to project commencement. We will seek approval for WP4 during the project as this work will involve serving prisoners and will need to meet more exacting specifications.

The ethical issues in this project relate to obtaining and analysing prisoner health data (both quantitative and qualitative). We have had several discussions with colleagues at Spectrum CIC about this research, and while specific service level agreements will be required between Spectrum and LIHS to share the quality indicator data, we already have initial agreement to extract the quality indicators for the prisons where they are responsible for primary healthcare from SystmOne Prison and its replacement (see attached letter).

All prisoner quality of care indicator data provided by Spectrum CIC will be anonymised, and the research team is experienced in handling these sorts of data and in maintaining appropriate levels of security and confidentiality, with standard operating procedures already in place within LIHS.

We will work with our provider partners and PPI Panel to develop a proposal on how best to inform prisoners about the use of their data for research and then seek further advice from Her Majesty's

Prison & Probation /Health Research Authority during the approval process e.g. the provision of posters in easy to understand format in healthcare waiting rooms (also known as holding cells). One issue is that we will be collecting retrospective data covering the period 2015-18, including for prisoners who will have been released. We intend to apply the same standards that we have already applied to collecting and analysing data to assess the quality of care for community general practice populations. Data extraction will occur with the provider organisation and only anonymised data stripped of strong identifiers will be released outside of this setting for analysis. As for other quality of care studies and clinical audits, we recognise that seeking individual consent for access is impractical and risk selection bias that will invalidate the findings.

In addition, the research team comprises members who are familiar with the intricacies involved in conducting research within the prison sector, and in obtaining the necessary permissions from the Her Majesty's Prison & Probation Service and individual Prison Governors approval for prisoner interviews/workshops.

Patient and Public Involvement

Since the outline our PPI Co-I has unfortunately had to step down. As a co-applicant, LMW had reviewed the proposal, and from her experience as a prisoner supported the development of interventions to improve the quality of care in prisoners, and had made sure that the prisoner perspective is gathered at each stage of the project. However, we are delighted that Fergus (surname not provided for confidentiality reasons) has agreed to be our PPI rep on the research team. He is an ex service user and a volunteer and peer mentor at Spectrum People and with WYFI (West Yorkshire Finding Independence) two independent charities that promote social inclusion, including with those with previous criminal convictions. Spectrum People is a charity and a subsidiary of Spectrum CIC. Its remit is to work with vulnerable people in order to aid their better integration into society. We have already approached Spectrum People which has agreed that their service users are willing to support this research study in an advisory capacity.

Fergus does not wish to be a Co-I but will be directly involved in making sure that the prisoner perspective is gathered at each stage of the project and will harness his links with these two organisations and receive support from them to further develop the project through workshops, interviews and discussion groups. Therefore he and the groups are ideally placed to provide ex-offenders along with the research teams own contacts and experience of the inclusion of offenders.

By involving current and ex-prisoners throughout the project, we can draw on their unique perspectives to identify pertinent research questions, outcomes relevant to them, and appropriate strategies to improve the quality and continuity of care they receive. Prisoners will be crucial to the identification and development of interventions through their involvement in the interviews and stakeholder panels (WP1&2) and consensus workshops (WP4). Fergus and the 'experts by experience' signposted will be responsible for identifying prisoner participants and liaising with them throughout the process, which they have done in a number of other projects. They will also be involved in co-designing and facilitating workshops and co-producing project materials for offenders and appropriate support/funding is provided for them. The 'experts by experience' panel comprises of representatives from Spectrum People who have previously been prisoners within a UK prison. Provision will be made for gender balance throughout the duration of the PPI input.

Fergus's role will be crucial in liaising with prisoner participants and will be supported by the Research Programme Manager, the prison charities and further prisoner representation on the steering committee. Through the skills and experience of PPI held at LIHS, the Research Programme Manager will be fully supported to facilitate his meaningful involvement within project meetings (e.g. by providing a glossary of terms and by acting as his mentor).

Expertise and Justification of Support required

Relevant expertise

This proposal builds upon a unique collaboration between Leeds Institute of Health Sciences (LIHS), prison health care practitioners and providers (Spectrum Community Health CIC and Care UK) and commissioners responsible for prisoner healthcare. LIHS brings methodological rigour and innovation to problem-driven, applied research; we bring particular skills in quality improvement research and analysis of large datasets (TF, RF, KM). Spectrum Community Health CIC and Care UK provides health care to over 50 prisons and is at the forefront of a range of initiatives in the sector, including clinical academics with direct experience in the organisation and delivery of care (NW, KM).

This proposal is also underpinned by our existing strong links with North of England Commissioning Support (NECS), which works across the UK to support health and social care customers (including STPs, CCGs, Trusts and Accountable Care Systems (ACS)) in meeting strategic and operational challenges, to improve outcomes and increase efficiency. The NECSU (and its forerunners) has been a critical partner in a number of past and present NIHR-funded studies, for example, by ensuring timely research governance permissions, directly supporting research, and promoting dialogue with commissioners. NS, Health & Justice Lead for NECSU, brings considerable knowledge of the complexities of healthcare provision for offenders.

Our team includes topic experts, methodologists and clinicians. The co-investigators combine track records in prisoner research (NW, LS), primary care research (RF, TF, EM, KM), quality improvement (LS, EM, RF, NW), handling and analysis of large datasets (TF, RF, EM, KM), and qualitative research (LS, EM). Fergus has experienced life as a prisoner, and brings knowledge that is valuable to many aspects of this project. Other collaborators, as Steering Group members, will provide advice from a range of perspectives: Kate Davies is Head of Public Health, Armed Forces and their Families and Health & Justice, NHS England, (which has overall responsibility for commissioning prison health services); Dr Linda Harris is Chief Executive of Spectrum Community Health (currently providing primary healthcare in 11 adult prisons).

As outlined below research posts have been costed only for the time-periods required during the study and with time-appropriate costing for applicants this provides value for money by achieving the study objectives without excessive resource use. Having identified existing quality improvement interventions, gained insights into barriers to and enablers of change (WP2), and analysed inappropriate variations in practice within prison (WP3) based on identified quality indicators (WP1), we will identify a list of quality indicators and a suite of improvement interventions that can be sustainably embedded within available systems and resources (WP4).

Justification of support

This study will provide a significant resource from which to bring about benefits to patients. While the current study involves a data snapshot for a specific time period, the products of this process – permissions, techniques to generate relevant quality indicator codes, algorithms for interrogating data, determining completeness and comprehensiveness of the resource – allow these methods to be translated to subsequent research.

Our longer term goal is to further develop and evaluate interventions to improve quality of prisoner care using rigorous methods, with embedded process and economic evaluations. This study will demonstrate the feasibility of accessing and interrogating prisoner health data across boundaries and prisons in developing indicators which can be used as outcome measures. Furthermore, this study will further embed existing collaborations of academic researchers, providers and commissioners of prisoners' health services to provide a sustainable basis to develop further research with the aim to improve the quality of care and outcomes for prisoners.

CALCULATION OF RESEARCH COSTS:

Staff

LS (30%) as PI will have overall responsibility for achieving objectives and milestones, with specific lead responsibility for the qualitative interviews to explore attitudes, perceptions and experiences concerning the quality of care for prisoners (WP2);

RF (10%) as Co-PI will have joint responsibility for the study and provide mentoring support to LS, with specific lead responsibility for the identification of indicators (WP1 – co-lead) and consensus panel development of interventions (WP4);

TF (10%) has lead responsibility for the analysis of the prisoner primary care records (WP3) and co-lead on identifying quality indicators (WP1);

NW (8%), EM (5%) and KM (5%), will provide clinical and methodological input, conduct consensus panels as well as identifying appropriate participants for the stakeholder panel (WP1), interviews (WP2) and intervention development (WP4). Furthermore NW will provide liaison with Spectrum Community Health CIC in accessing the primary care prisoner data.

NS (10%) as Health & Justice Lead for North of England Commissioning Support (NECS) will provide expertise on commissioning offender health services, including strategic and operational level knowledge and identify appropriate participants for WP1-4s with access to her team and resources (e.g. HNAs).

Fergus will liaise with prisoner participants as well as providing insight into the experiences of prisoners, reflecting his contribution (44 days) at recommended INVOLVE rates.

Research programme manager (50%) to manage the day-to-day aspects of the project, runs to timescale, gaining ethical and research governance approvals for WP4, organise and convene the quarterly steering committee, take a lead for PPI management and work closely with Fergus and other key PPI reps, primary stakeholder engagement, agree and implement all SLAs and collaboration agreements, manage all invoicing and payments. Appropriate admin and clerical support (20%), will be provided, with interview transcription.

Qualitative researcher (100%) will be involved throughout the study but in a more focused manner in WP2 where they will lead the qualitative fieldwork and analysis. This person will assist in both the preparation and delivery of the stakeholder and consensus workshops in WP1 and WP4. They will be responsible for delivery of the scoping review which now occurs prior to WP1.

Research Assistant (100%) will be based in Spectrum CIC and will primarily be responsible for extracting the primary care including development of algorithms for the selected quality indicators, but will also be involved in interviews and both stakeholder and consensus workshops.

Consumables

Payment required for participants to attend stakeholder, consensus and dissemination workshops (£350 + NI per GP session, £175 + NI per nurse session, £150 + NI per PPI).

General study costs

Costs required to run the steering groups, stakeholder panels, consensus workshops and the dissemination workshop, including, room hire (£120 per meeting), refreshments (£5 pp half day, £15 pp full day), and printing (£500 per meeting) of the appropriate materials.

Travel Costs

Travel and subsistence for the qualitative researcher to undertake the interviews (£70 per interview); participants travel to stakeholder panels, consensus panels and dissemination workshop (£60 per participant from across Y&H); collaborators travel to Steering Groups(£60 per collaborator); and for Co-Is out with Leeds to attend meetings (£200-£320 per year).

Equipment

Encrypted laptop (£650) and audio recorder (£250) required for interviews; with secure archiving of interviews and the primary care prison data (£200). Appropriate statistical software (STATA 15) for analysis of primary care data (£600).

Dissemination

Two members of the team to attend 2 appropriate conferences for wider dissemination (£780 pp). We will prioritise open access journals (4 papers £8000 total).

NHS SUPPORT AND TREATMENT COSTS: Discussions with the NIHR Clinical Research Network: Yorkshire and Humber confirmed that none of the activities within the interviews, stakeholder panels, consensus panels and analysis of primary care data would directly contribute to NHS service provision, and so were not to be attributed as Research Costs in line with AcoRD.

REFERENCES

1. Marshall T, Simpson S, Stevens A. Use of health services by prison inmates: comparisons with the community. *Journal of Epidemiology and Community Health* 2001; 55: 364-365.
2. Bridgwood A, Malbon G. *Survey of the physical health of prisoners 1994*. London: Office of Population Censuses and Surveys, 2005.
3. Plugge E, Douglas N, Fitzpatrick R. *The health of women in prison: study findings*. Oxford: The University of Oxford, 2006.
4. Condon L, Hek G, Harris F. Public health, health promotion and the health of people in prison. *Community Practitioner* 2006; 79(1): 19-22.
5. Weild A, Gill ON, Bennett D, Livingstone S, Parry J, Curran L. *The prevalence of anti-HIV, antihepatitis B core, anti-hepatitis C antibodies and associated risk factors in prisoners: England and Wales, 1997-1998*. London: Public Health Laboratory Service, 1998.
6. Rennie C, Senior J, Shaw J. The future of offender health: evidencing mainstream health services through the offender pathway. *Criminal Behaviour and Mental Health* 2009; 19: 1-8.
7. Hobbs FDR, Bankhead C, Mukhtar T, Stevens S, Perera-Salazar R, Holt T, Salisbury C; National Institute for Health Research School for Primary Care Research. *Clinical workload in UK primary care: a retrospective analysis of 100 million consultations in England, 2007-14*. Lancet. 2016 Jun 4; 387(10035): 2323-2330.
8. Schneider EC, Sarnak DO, Squires D, Shah A, and Doty MM. *Mirror, Mirror 2017: International Comparison Reflects Flaws and Opportunities for Better U.S. Health Care*, The Commonwealth Fund, July 2017.
9. Starfield B, Shi L, Macinko J. *Contribution of primary care to health systems and health.* Milbank Q. 2005;83(3):457-502.
10. Dawda P, Jenkins R, Varnam R. *Quality improvement in general practice.* London: The King's Fund; 2010.
11. Kings Fund. *Improving the quality of care in general practice: Report of an independent inquiry commissioned by The King's Fund*, March 2011
12. Hunt K, Demou E, Sweeting H, Boyd K, Craig P, Conaglen P, Semple S, Eadie D, Leyland A, Pell J, Bauld L. Evaluating graduated progress towards and impacts of the implementation of indoor smoke free prison facilities in Scotland. NIHR Public Health Research Programme – 15/55/44.
13. Asherson P, Forrester A, Howitt S, Young S, Thomson L, Landau S, Strang J, Lawrie S, Fahy T. Randomised controlled trial of the short term effects of OROS-methylphenidate on ADHD symptoms and behavioral outcomes in young male prisoners with attention-deficit/hyperactivity disorder. *NIHR Efficacy and Mechanism Evaluation Programme - 14/23/17*
14. Senior J, Forsyth K, Walsh E, O'Hara K, Stevenson C, Hayes A, et al. *Health and social care services for older male adults in prison: the identification of current service provision and piloting of an assessment and care planning model*. Health Service Delivery Research 2013;1(5)
15. Shaw J, Hayes A, Sanders C, Senior J, Perryman K, Piper M, Domone R, Meacock R, Webb R, Karim S, Ware S, Forsyth K, Burns A, Challis D, Emsley R, Fazel S. Dementia and Cognitive Impairment in the Older Prison Population of England and Wales: Identifying Individual Need and Developing a skilled, Multi-Agency Workforce to Deliver Targeted and Responsive Services. *Health Services and Delivery Research programme – 14/197/65*
16. Morrissey C, Geach N, Alexander R, Chester V, Devapriam J, Duggan C, Langdon PE, Lindsay B, McCarthy J, Walker DM. *Researching outcomes from forensic services for people with intellectual or developmental disabilities: a systematic review, evidence synthesis and expert and patient/carer consultation.* Southampton (UK): NIHR Journals Library; 2017 Jan.
17. Byng R, et al. Developing and evaluating a collaborative care intervention for prisoners, with common mental health problems, near to and after release (Engager 2). *Programme Grants for Applied Research - RP-PG-1210-12011*
18. Shaw J, Conover S, Herman D, Jarrett M, Leese M, McCrone P, et al. *Critical time Intervention for Severely mentally ill Prisoners (CrISP): a randomised controlled trial.* Health Service Delivery Research 2017;5(8)

19. Shaw J, Herman D, Edge D, Senior J, Leese M, Wright N, Jarrett M, Murphy C, Conover S, Susser E, Thornicroft G, Marshall M, McCrone P. Critical Time Intervention for Severely Mentally Ill Released Prisoners: A Randomised Control Trial (CrISP). *Health Services and Delivery Research programme – 09/1004/15*
20. National Institute for Health and Care Excellence. Hypertension in adults: diagnosis and management (NICE Clinical guideline 127). NICE, 2016. www.nice.org.uk/guidance/cg127 (accessed 13-2-18)
21. National Institute for Health and Care Excellence. Asthma (NICE Quality standard 25). NICE, 2017. www.nice.org.uk/guidance/qs25 (accessed 13-2-18)
22. Forsyth K, Archer-Power L, Senior J, Meacock R, Webb R, Emsley R, et al. *The effectiveness of the Older prisoner Health and Social Care Assessment and Plan (OHSCAP): a randomised controlled trial*. Health Services Delivery Research 2018;5(31)
23. Rushforth B, Stokes T, Andrews E, Willis TA, McEachan R, Faulkner S, Foy R. Developing 'high impact' guideline-based quality indicators for UK primary care: a multi-stage consensus process. *BMC Family Practice* 2015;16:156.
24. Kronenberg C, Doran T, Goddard M, Kendrick T, Gilbody S, Dare CR, Aylott L, Jacobs R. *Identifying primary care quality indicators for people with serious mental illness: a systematic review.* Br J Gen Pract. 2017 Aug;67(661)
25. Blood Pressure Lowering Treatment Trialists' Collaboration. *Blood pressure-lowering treatment based on cardiovascular risk: a meta-analysis of individual patient data.* Lancet. 2014 Aug 16;384(9943):591-598.
26. Willis TA, West R, Rushforth B, Stokes T, Glidewell L, Carder P, Faulkner S, Foy R; ASPIRE programme team. *Variations in achievement of evidence-based, high-impact quality indicators in general practice: An observational study.* PLoS One. 2017 Jul 13;12(7)
27. NHS England. Strategic direction for health services in the justice system: 2016 – 2020. NHS England, 2016. https://www.england.nhs.uk/wp-content/uploads/2016/10/hlth-justice-directions-v11.pdf (accessed 19-4-17)
28. NHS England. Health and Justice Indicators of Performance (HJIPs) Adult Secure Estate User Guide 2017-18, 2017. https://supplierlive.proactisp2p.com/PublicDocument/Get?d=l5dgapako5621843v2kb35xot3 (accessed 13-2-18)
29. National Institute for Health and Care Excellence. Physical health of people in prison (NICE guideline 57). NICE, 2016. www.nice.org.uk/guidance/ng57 (accessed 19-4-17)
30. National Institute for Health and Care Excellence. Mental health of adults in contact with the criminal justice system (NICE guideline 66). NICE, 2017. www.nice.org.uk/guidance/ng66 (accessed 19-4-17)
31. Grimshaw JM, Eccles MP, Lavis JN, Hill SJ, Squires JE. Knowledge translation of research findings. *Implementation Science* : IS. 2012;7:50.
32. Murphy MK, Black NA, Lamping DL, McKee CM, Sanderson CFB, Askham J, Marteau T. Consensus development methods, and their use in clinical guideline development. *Health Technology Assessment*. 1998;2(3):i-iv, 1-88.
33. Hennink M, Kaiser B, Marconi V (2016) Code saturation versus meaning saturation. How many interviews are enough? *Qualitative Health Research* 27 (4) 591-608.
34. Guest G, Macqueen K, Namey E. *Applied Thematic Analysis*. Sage, London. 2012
35. Ferlie EB, Shortell SM: Improving the Quality of Health Care in the United Kingdom and the United States: A Framework for Change. *Milbank Q 2001, 79:281-315*.
36. Timmermans, S., Tavory, I., 2012. Theory construction in qualitative research: from grounded theory to abductive analysis. *Sociological Theory* 30 (3), 167e186
37. Ministry of Justice. Prison Population Projections 2017 to 2022, England and Wales. London, 2017.
38. Lawrence Joseph, Theresa W. Gyorkos, Louis Coupal; Bayesian Estimation of Disease Prevalence and the Parameters of Diagnostic Tests in the Absence of a Gold Standard, *American Journal of Epidemiology* 1995;141(3): 263–272
39. Peduzzi P, Concato J, Kemper E, Holford TR, Feinstein AR. A simulation study of the number of events per variable in logistic regression analysis, *Journal of Clinical Epidemiology*, 1996 Dec;49(12):1373-9

40. Ruppertsberg AI, Ward V, Ridout A, Foy R. The development and application of audit criteria for assessing knowledge exchange plans in health research grant applications. *Implementation Science* 2014; 9:93
41. Ward V, Smith S, Foy R, House A, Hamer S. Planning for knowledge translation: a researchers' guide. *Evidence and Policy* 2010;6: 527-541
42. Innvaer S, Vist G, Trommald M, Oxman A: Health policy-makers' perceptions of their use of evidence: a systematic review. *Journal of Health Services Research Policy* 2002, 7:239-244
